# Supplementary material for: [1,n]-Metal migrations for directional translational motion at the molecular level
Source: Nat Commun. 2025 Jun 5;16:5232. doi: 10.1038/s41467-025-60383-3 (PMC12141569; doi:10.1038/s41467-025-60383-3)
Supplement: Supplementary file 1 — Supplementary Information [file 41467_2025_60383_MOESM1_ESM.pdf]

[1,n]-Metal migrations for directional translational motion at the molecular level

Supplementary Information

Emma L. Hollis,[1] Michael N. Chronias,[1] Carlijn L. F. van Beek,[1,2] Paul J. Gates[1] & Beatrice S. L. Collins*[1]

[1]School of Chemistry, University of Bristol, Cantock's Close, Bristol, BS8 1TS, UK.

[2]Stratingh Institute for Chemistry, University of Groningen, Groningen 9747 AG, The Netherlands.

Contents

1) General Experimental Details 3
2) Synthesis of Polyaromatic Tracks 5
2.1) Synthesis of Biaryl Halide Precursors to Tracks 1–8 5
2.2) Synthesis of Biaryl Boronic Acid Pinacol Ester Tracks 1–8 13
2.3) Synthesis of Bis-Ortho-Deutero Biaryl Boronic Acid Pinacol Ester Track D_2-1 21
2.4) Synthesis of Triaryl Boronic Acid Pinacol Ester Track 9 26
2.5) Synthesis of Bridged Biaryl Boronic Acid Pinacol Ester Tracks 10–15 30
2.6) Synthesis of Polyaniline Tracks 16 and 17 46
3) Rhodium-Catalysed Norbornane Incorporation for Controlled Translational Motion Along Polyaromatic Tracks 53
3.1) General Procedures 53
3.2) Analysis of Reactions using ^{1}H NMR Spectroscopy 55
3.3) Details on Characterisation and Representation of Polysubstituted Polyaromatic Tracks 57
3.4) Selected Optimisation for the Rhodium-Catalysed Incorporation of Norbornene into 2-(4'-Fluoro-[1,1'-biphenyl]-4-yl)-4,4,5,5-tetramethyl-1,3,2-dioxaborolane (1) 58
3.5) Biaryl Boronic Acid Pinacol Ester Tracks 1–8 as Substrates for Rh-Catalysed Norbornane Incorporation 60
3.6) Triaryl Boronic Acid Pinacol Ester Track 9 as Substrate for Rh-Catalysed Norbornane Incorporation 74
3.7) Bridged Biaryl Boronic Acid Pinacol Ester Tracks 10–15 as Substrates for Rh-Catalysed Norbornane Incorporation 76
3.8) Mechanistic Studies to Investigate Directionally Sequential Processive Incorporation of Norbornane Units 87
3.8.1) Deuterium oxide quenching experiment 87
3.8.2) Deuterated isopropyl alcohol quenching experiment 89
3.8.3) Study of increasing equivalents of H_2O on optimised reaction conditions for track 1 90
3.8.4) Di-1 subjected to optimised reaction conditions 92
3.8.5) Bis-deutero track D_2-1 subjected to optimised reaction conditions 93
3.9) Proposed Mechanism for Directional Motion of the Rhodium Centre 94
3.10) Polyaniline Boronic Acid Pinacol Ester Tracks 16 and 17 as Substrates for Rh-Catalysed Norbornane Incorporation 95
3.10.1) *N^1,N^4*-dimethyl-*N^1*-phenyl-*N^4*-(4-(4,4,5,5-tetramethyl-1,3,2-dioxaborolan-2-yl)phenyl)benzene-1,4-diamine (16) 95
3.10.2) *N^1,N^4*-dimethyl-*N^1*-(4-(methyl(4-(4,4,5,5-tetramethyl-1,3,2-dioxaborolan-2-yl)phenyl)amino)phenyl)-*N^4*-(4-(methyl(phenyl)amino)phenyl)benzene-1,4-diamine (17) 97
4) NMR and Selected HRMS Spectra 99
5) References 233

1) General Experimental Details

Reagents and solvents. Reactions with air- or moisture-sensitive materials were carried out in oven- or flame-dried glassware under a nitrogen atmosphere using standard Schlenk line techniques. Bulk solutions were evaporated under reduced pressure using a Büchi or IKA rotary evaporator. Unless otherwise stated, all reagents, catalysts, and solvents were obtained from commercial sources and were used without further purification. All anhydrous solvents were commercially supplied or, in the case of toluene, DCM, Et_2O, THF, MeCN or hexane, provided by the communal stills of the School of Chemistry, University of Bristol (Anhydrous Engineering Ltd. modified Grubbs system of double alumina and alumina-copper catalysed drying columns). H_2O used is deionised using a Veolia laboratory water purification system.

Chromatography. Flash column chromatography was carried out using VWR silica gel 40–63 µm or using a Biotage Isolera Four, Biotage SNAP Ultra 10 g or 5 g columns. Thin-layer chromatography (TLC) was performed using Merck aluminium-backed plates (Silica gel 60 F_{254}). Compounds were visualized under UV light, at either 254 nm or 365 nm, by staining with aqueous basic potassium permanganate, aqueous acidic 2,4-dinitrophenylhydrazine (DNP) solution, ethanolic phosphomolybdic acid (PMA), ceric ammonium molybdate (CAM), or ethanolic acidic *p*-anisaldehyde. Normal phase preparatory High Performance Liquid Chromatography (HPLC) was performed on an ACCQPrep HP125 system. Preparatory Column: Kromasil 60-5SIL 250 mm x 21.2 mm. Or Phenomonex Luna-2 250 mm x 21.2 mm.

Nuclear magnetic resonance spectroscopy. ^{1}H, $^{13}C\{^{1}H\}$, $^{13}C\{^{19}F\}$ ^{19}F, $^{19}F\{^{1}H\}$ nuclear magnetic resonance (NMR) spectra were recorded using Jeol ECS 400 MHz, Jeol JNM-ECZ 400 MHz, Varian ^{13}C NMR 400 MHz, Bruker Ascend^{TM} DPX 400 MHz, Bruker Avance III HD 500 MHz (TCI Prodigy probe), Bruker Avance III HD 500 MHz (equipped with Cryo Probe), and Bruker Neo 600 MHz (equipped with Cryo Probe) spectrometers. ^{1}H and ^{13}C chemical shifts (δ) are referenced to $CDCl_3$ (^{1}H: 7.26 ppm, ^{13}C: 77.16 ppm), $(CD_3)_2CO$ (^{1}H: 2.05 ppm, ^{13}C: 29.84 ppm), C_6D_6 (^{1}H: 7.16 ppm, ^{13}C: 128.06 ppm) or $(CD_3)_2SO$ (^{1}H: 2.50 ppm, ^{13}C: 39.52 ppm) and are given in parts per million (ppm). Coupling constants (*J*) are given in Hertz (Hz) and reported as observed. The ^{1}H NMR data are reported as follows: chemical shift (multiplicity, coupling constants, integral). The $^{13}C\{^{1}H\}$ and ^{19}F NMR data are reported as follows: chemical shift (multiplicity, coupling constants). Splitting patterns are abbreviated to: singlet (s), doublet (d), triplet (t), quartet (q), multiplet (m), broad (br.) or some combination thereof.

The baselines of ^{13}C NMR spectra acquired from spectrometers equipped with a Cryo Probe were corrected with a polynomial fit method (polynomial order 15).

Infrared spectroscopy. Infrared (IR) spectra were recorded on a Perkin Elmer Spectrum Two FT-IR spectrophotometer as a thin film with selected absorption maxima (ν) reported in wavenumbers (cm^{-1}).

Mass spectrometry. High resolution mass spectrometry (HRMS) spectra were recorded on a Thermo Scientific QExactive by Electron Ionisation (EI), on a Bruker Daltonics micrOTOF II by Electrospray Ionisation (ESI), on a Waters Synapt G2S by Nanospray, or on a Thermo Scientific Orbitrap Elite by Atmospheric Pressure Chemical Ionisation (APCI). In the cases where the reported compound contains boron, silicon, chlorine or bromine the masses are given for the ^{11}B, ^{28}Si ^{35}Cl or ^{79}Br isotopes respectively.

Melting points. Melting points were recorded in degrees Celsius (°C), using a Gallenkamp or a Cole-Parmer SMP50 melting point apparatus and are reported uncorrected.

Naming of Compounds. Compound names are those generated by ChemBioDraw 20.0 software (PerkinElmer), following IUPAC nomenclature.

2) Synthesis of Polyaromatic Tracks

2.1) Synthesis of Biaryl Halide Precursors to Tracks 1–8

Biaryl bromide precursors **S1**–**S8** and **D_2-S1** were synthesised via a palladium-catalysed Suzuki-Miyaura cross-coupling reaction between 1-bromo-4-iodobenzene and the appropriate phenyl boronic acid coupling partner. In some cases, unreacted 1-bromo-4-iodobenzene (**^{1}H NMR** (400 MHz, $CDCl_3$) δ 7.59 – 7.50 (m, 2H), 7.25 – 7.19 (m, 2H). **^{13}C NMR** (101 MHz, $CDCl_3$) δ 139.2, 133.6, 122.3, 92.1) could not be separated from the desired product and the mixture was carried forward into the following borylation, at which point the tracks **1**–**8** and D_2-**1** could be isolated in analytically pure form (> 97% purity by ^{1}H NMR spectroscopy).

4-Bromo-4'-fluoro-1,1'-biphenyl (S1)

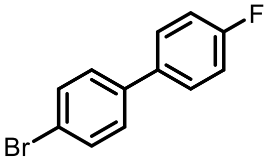

A solution of 1-bromo-4-iodobenzene (11.3 g, 40.0 mmol, 1.00 eq), 4-fluorophenylboronic acid (6.72 g, 48.0 mmol, 1.20 eq), tetrakis(triphenylphosphine)palladium(0) (1.15 g, 2.50 mol%) and potassium carbonate (11.1 g, 80.0 mmol, 2.00 eq) in toluene: ethanol: water (300 mL, 8:1:1) was heated to reflux and stirred at this temperature for 12 h. After this time the reaction mixture was cooled to room temperature and poured over water (30 mL). The aqueous phase was then extracted with ether (3 × 50 mL) and the combined organic phases were washed with brine (3 × 30 mL), dried over $MgSO_4$, filtered, and concentrated under reduced pressure. The resulting residue was purified by flash column chromatography on silica gel (hexane, isocratic) to afford the title compound (6.56 g, 65%) as a white solid.

R_f (hexane): 0.33. **^{1}H NMR** (400 MHz, $CDCl_3$): δ 7.60 – 7.46 (m, 4H), 7.44 – 7.36 (m, 4H), 7.18 – 7.08 (m, 2H). **^{13}C NMR** (101 MHz, $CDCl_3$) δ 163.1 (d, $^1J_{C\text{–}F}$ = 247.1 Hz), 139.6, 136.6 (d, $^4J_{C\text{–}F}$ = 3.3 Hz), 132.4, 129.0 (d, $^2J_{C\text{–}F}$ = 9.6 Hz), 122.0, 116.3 (d, $^2J_{C\text{–}F}$ = 21.3 Hz). **^{19}F NMR** (376 MHz, $CDCl_3$): δ −114.7–−115.1 (m). **IR** ν (cm^{-1}): 1585, 1596, 1514, 1457, 1387, 1300. **HRMS** (EI) *m/z*: Calculated for $C_{12}H_8BrF$ 249.9793. Found 249.9788 $[M]^+$. **mp**: 95–98 °C.

4-Bromo-4'-(trifluoromethyl)-1,1'-biphenyl (S2)

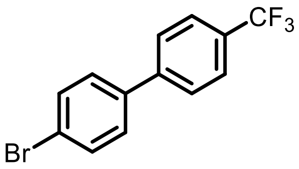

A solution of 1-bromo-4-iodobenzene (1.87 g, 6.61 mmol, 1.10 eq), (4-(trifluoromethyl)phenyl)boronic acid (1.14 g, 6.00 mmol, 1.00 eq), tetrakis(triphenylphosphine)palladium(0) (0.208 g, 3.00 mol%) and potassium carbonate (1.66 g, 12.0 mmol, 2.00 eq) in toluene: ethanol: water (80 mL, 8:1:1) was heated to reflux and stirred at this temperature for 12 h. After this time the reaction mixture was cooled to room temperature and poured over water (30 mL). The aqueous phase was then extracted with ethyl acetate (3 × 30 mL) and the combined organic phases were washed with brine (3 × 20 mL), dried over $MgSO_4$, filtered, and concentrated under reduced pressure. The resulting residue was purified by flash column chromatography on silica gel (hexane, isocratic) to afford the title compound (1.67 g, 92%) as a white powder. Note that the sample contains 1-bromo-4-iodobenzene as an impurity (approx. 13%) and the mixture was carried through to the subsequent borylation reaction without further purification.

***R*$_f$** (hexane): 0.30. **^{1}H NMR** (400 MHz, $CDCl_3$) δ 7.74 – 7.63 (m, 4H), 7.60 (d, *J* = 8.7 Hz, 2H), 7.46 (d, *J* = 8.7 Hz, 2H). **^{13}C{F^{19}} NMR** (101 MHz, $CDCl_3$) δ 143.6, 138.8, 132.3, 129.9, 129.0, 127.4, 126.0, 124.3, 122.8. **^{19}F NMR** (376 MHz, $CDCl_3$): δ −62.37. **IR** ν (cm^{-1}): 2956, 2928, 2855, 1391, 1339, 1166. **HRMS** (EI) *m/z*: Calculated for $C_{13}H_8FBr$ 299.9761. Found 299.9752 $[M]^+$. **mp**: 101–107 °C.

4-Bromo-4'-(methoxy)-1,1'-biphenyl (S3)

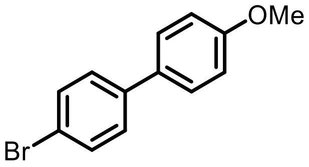

A solution of 1-bromo-4-iodobenzene (1.9 g, 6.6 mmol, 1.1 eq), (4-(methoxy)phenyl)boronic acid (0.91 g, 6.0 mmol, 1.0 eq), tetrakis(triphenylphosphine)palladium(0) (0.21 g, 3.0 mol%) and potassium carbonate (1.7 g, 12 mmol, 2.0 eq) in toluene: ethanol: water (80 mL, 8:1:1) was heated to reflux and stirred at this temperature for 12 h. After this time the reaction mixture was cooled to room temperature and poured over water (30 mL). The aqueous phase was then extracted with ethyl acetate (3 × 30 mL) and the combined organic phases were washed with brine (3 × 20 mL), dried over $MgSO_4$, filtered, and concentrated under reduced pressure. The resulting residue was purified by flash column chromatography on silica gel (hexane, isocratic) to afford the title compound (1.3 g, 82%) as a white powder. Note that the sample contains 1-bromo-4-iodobenzene as an impurity (approx. 9%) and the mixture was carried through to the subsequent borylation reaction without further purification.

***R*f** (hexane): 0.35. ^{1}H NMR (400 MHz, $CDCl_3$) δ 7.53 (d, *J* = 8.7 Hz, 2H), 7.49 (d, *J* = 8.9 Hz, 2H), 7.41 (d, *J* = 8.7 Hz, 2H), 6.98 (d, *J* = 8.8 Hz, 12), 3.85 (s, 3H). **^{13}C NMR** (101 MHz, $CDCl_3$) δ 159.5, 139.9, 132.6, 131.9, 128.4, 128.1, 120.9, 114.4, 55.5. **IR** ν (cm^{-1}): 2964, 2924, 2855, 1483. **HRMS** (EI) *m/z*: Calculated for $C_{13}H_{11}OBr$ 261.9993. Found 261.9984 $[M]^+$. **mp:** 130–137 °C.

4-Bromo-3'-(methoxy)-1,1'-biphenyl (S4)

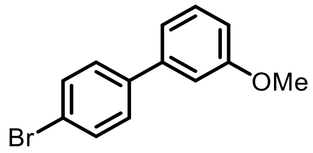

A solution of 1-bromo-4-iodobenzene (1.9 g, 6.6 mmol, 1.1 eq), (3-methoxyphenyl)boronic acid (0.91 g, 6.0 mmol, 1.0 eq), tetrakis(triphenylphosphine)palladium(0) (0.21 g, 3.0 mol%) and potassium carbonate (1.7 g, 12 mmol, 2.0 eq) in toluene: ethanol: water (80 mL, 8:1:1) was heated to reflux and stirred at this temperature for 12 h. After this time the reaction mixture was cooled to room temperature and poured over water (30 mL). The aqueous phase was then extracted with ethyl acetate (3 × 30 mL) and the combined organic phases were washed with brine (3 × 20 mL), dried over $MgSO_4$, filtered, and concentrated under reduced pressure. The resulting residue was purified by flash column chromatography on silica gel (hexane, isocratic) to afford the title compound (1.6 g, quant.) as a colourless oil.

***R*f** (hexane): 0.30. **^{1}H NMR** (400 MHz, $CDCl_3$) δ 7.56 (d, *J* = 8.7 Hz, 2H), 7.45 (d, *J* = 8.7 Hz, 2H), 7.36 (t, *J* = 7.9 Hz, 1H), 7.17 – 7.12 (m, 1H), 7.10 – 7.06 (m, 1H), 6.92 (dd, *J* = 8.1, 2.6 Hz, 1H), 3.87 (s, 3H). **^{13}C NMR** (101 MHz, $CDCl_3$) δ 160.2, 141.7, 140.1, 132.0, 130.1, 128.9, 121.8, 119.6, 113.1, 112.9, 55.5. **IR** ν (cm^{-1}): 2960, 2923, 2859, 1607, 1561, 1476. **HRMS** (EI) *m/z*: Calculated for $C_{13}H_{11}OBr$ 261.9993. Found 261.9983 $[M]^+$.

4-Bromo-4'-methyl-1,1'-biphenyl (S5)

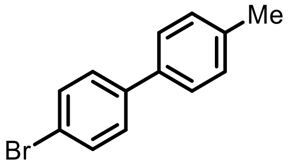

A solution of 1-bromo-4-iodobenzene (2.83 g, 10.0 mmol, 1.00 eq), *p*-tolylboronic acid (1.63 g, 12.0 mmol, 1.20 eq), tetrakis(triphenylphosphine)palladium(0) (0.347 g, 3.00 mol%) and potassium carbonate (2.76 g, 20.0 mmol, 2.00 eq) in toluene: ethanol: water (100 mL, 8:1:1) was heated to reflux and stirred at this temperature for 12 h. After this time the reaction mixture was cooled to room temperature and poured over water (30 mL). The aqueous phase was then extracted with ethyl acetate (3 × 30 mL) and the combined organic phases were washed with brine (3 × 30 mL), dried over $MgSO_4$, filtered, and concentrated under reduced pressure. The resulting residue was purified by flash column chromatography on silica gel (hexane, isocratic) to afford the title compound (2.35 g, 95%) as a white solid.

***R*f** (hexane): 0.25. **^{1}H NMR** (400 MHz, $CDCl_3$): δ 7.58 – 7.50 (m, 2H), 7.49 – 7.39 (m, 4H,), 7.30 – 7.21 (m, 2H), 2.40 (s, 3H).**^{13}C NMR** (101 MHz, $CDCl_3$) δ 140.2, 137.7, 137.3, 131.9, 129.8, 128.7, 126.9, 121.3, 21.3. **IR** ν (cm^{-1}): 3044, 2980, 2917, 2844, 1611, 1507. **HRMS** (EI) *m/z*: Calculated for $C_{13}H_{11}Br$ 246.0044. Found 246.0039 $[M]^+$. **mp**: 123–127 °C.

4-Chloro-4'-fluoro-2-methyl-1,1'-biphenyl (S6)

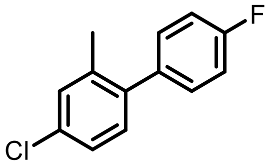

A solution of 4-chloro-1-iodo-2-methylbenzene (0.75 g, 3.0 mmol, 1.0 eq), (4-fluorophenyl)boronic acid (0.45 g, 3.3 mmol, 1.1 eq), tetrakis(triphenylphosphine)palladium(0) (0.10 g, 3.0 mol%) and potassium carbonate (0.83 g, 6.0 mmol, 2.0 eq) in toluene: ethanol: water (40 mL, 8:1:1) was heated to reflux and stirred at this temperature for 12 h. After this time the reaction mixture was cooled to room temperature and poured over water (30 mL). The aqueous phase was then extracted with ethyl acetate (3 × 30 mL) and the combined organic phases were washed with brine (3 × 20 mL), dried over $MgSO_4$, filtered, and concentrated under reduced pressure. The resulting residue was purified by flash column chromatography on silica gel (hexane, isocratic) to afford the title compound (0.40 g, 61%) as a colourless oil.

$\boldsymbol{R}_{\mathbf{f}}$ (hexane): 0.30. **^{1}H NMR** (400 MHz, $CDCl_3$) δ 7.59 – 7.50 (m, 2H), 7.26 (s, 1H), 7.19 – 7.11 (m, 2H), 7.00 – 6.90 (m, 2H), 2.24 (s, 3H). **^{13}C NMR** (126 MHz, $CDCl_3$) δ 162.2 (d, $^{1}J_{C\text{–}F}$ = 246.0 Hz), 139.5, 137.5, 136.8 (d, $^{4}J_{C\text{–}F}$ = 3.3 Hz), 133.2, 131.1, 130.8 (d, $^{3}J_{C\text{–}F}$ = 8.0 Hz), 126.0, 115.3 (d, $^{2}J_{C\text{–}F}$ = 21.5 Hz), 20.5. **^{19}F NMR** (376 MHz, $CDCl_3$) δ −115.24 – −115.53 (m). **IR** ν (cm^{-1}): 3042, 2957, 2925, 2857, 1728, 1594, 1513, 1478. **HRMS** (EI) *m/z*: Calculated for $C_{13}H_{10}ClF$ 220.0455 Found 220.0443 $[M]^+$.

4'-Bromo-4-fluoro-2-methyl-1,1'-biphenyl (S7)

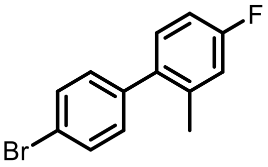

A solution of 1-bromo-4-iodobenzene (0.85 g, 3.0 mmol, 1.0 eq), (4-fluoro-2-methylphenyl)boronic acid (0.51 g, 3.3 mmol, 1.1 eq), tetrakis(triphenylphosphine)palladium(0) (0.10 g, 3.0 mol%) and potassium carbonate (0.83 g, 6.0 mmol, 2.0 eq) in toluene: ethanol: water (40 mL, 8:1:1) was heated to reflux and stirred at this temperature for 12 h. After this time the reaction mixture was cooled to room temperature and poured over water (30 mL). The aqueous phase was then extracted with ethyl acetate (3 × 30 mL) and the combined organic phases were washed with brine (3 × 20 mL), dried over $MgSO_4$, filtered, and concentrated under reduced pressure. The resulting residue was purified by flash column chromatography on silica gel (hexane, isocratic) to afford the title compound (0.71 g, 89%) as a colourless oil. Note that the sample contains 1-bromo-4-iodobenzene as an impurity (approx. 28%) and the mixture was carried through to the subsequent borylation reaction without further purification.

***R*f** (hexane): 0.30. **^{1}H NMR** (400 MHz, $CDCl_3$) δ 7.52 (d, J = 8.6 Hz, 2H), 7.19 – 7.09 (m, 3H), 7.00 – 6.87 (m, 2H), 2.22 (s, 3H). **^{13}C NMR** (126 MHz, $CDCl_3$) δ 162.3 (d, $^1J_{C\text{–}F}$ = 246.0 Hz), 140.0, 137.8 (d, $^3J_{C\text{–}F}$ = 7.8 Hz), 136.8 (d, $^4J_{C\text{–}F}$ = 2.8 Hz), 131.5, 131.2 (d, $^3J_{C\text{–}F}$ = 8.6 Hz), 131.1, 121.3, 117.1 (d, $^2J_{C\text{–}F}$ = 21.0 Hz), 112.8 (d, $^2J_{C\text{–}F}$ = 21.0 Hz), 20.7. **^{19}F NMR** (376 MHz, $CDCl_3$) δ −115.46 – −115.57 (m). **IR** ν (cm^{-1}): 3030, 2957, 2924, 2856, 1727, 1610, 1586, 1479. **HRMS** (EI) *m/z*: Calculated for $C_{13}H_{10}BrF$ 263.9950. Found 263.9944 $[M]^+$.

4-Chloro-4'-fluoro-2,2'-dimethyl-1,1'-biphenyl (S8)

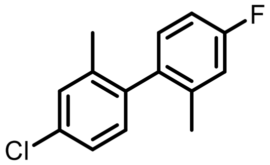

A solution of 4-chloro-1-iodo-2-methylbenzene (0.75 g, 3.0 mmol, 1.0 eq), (4-fluoro-2-methylphenyl)boronic acid (0.51 g, 3.3 mmol, 1.1 eq), tetrakis(triphenylphosphine)palladium(0) (0.10 g, 3.0 mol%) and potassium carbonate (0.83 g, 6.0 mmol, 2.0 eq) in toluene: ethanol: water (40 mL, 8:1:1) was heated to reflux and stirred at this temperature for 12 h. After this time the reaction mixture was cooled to room temperature and poured over water (30 mL). The aqueous phase was then extracted with ether (3 × 30 mL) and the combined organic phases were washed with brine (3 × 20 mL), dried over $MgSO_4$, filtered, and concentrated under reduced pressure. The resulting residue was purified by flash column chromatography on silica gel (hexane, isocratic) to afford the title compound (0.53 g, 75%) as a colourless oil.

***R*f** (hexane): 0.30. **^{1}H NMR** (400 MHz, $CDCl_3$) δ 7.29 – 7.26 (m, 1H), 7.20 (dd, J = 8.1, 2.2 Hz, 1H), 7.05 – 6.85 (m, 4H), 2.03 (s, 3H), 2.02 (s, 3H). **^{13}C NMR** (101 MHz, $CDCl_3$) δ 162.2 (d, $^1J_{C-F}$ = 245.3 Hz), 139.1, 138.3 (d, $^3J_{C-F}$ = 7.8 Hz), 138.2, 136.3 (d, $^4J_{C-F}$ = 3.2 Hz), 133.0, 130.9, 130.7 (d, $^3J_{C-F}$ = 8.2 Hz), 129.9, 125.9, 116.6 (d, $^2J_{C-F}$ = 20.8 Hz), 112.6 (d, $^2J_{C-F}$ = 20.9 Hz), 20.0 (d, $^4J_{C-F}$ = 1.6 Hz), 19.8. **IR** ν (cm^{-1}): 3027, 2955, 2923, 1729, 1614, 1586, 1476. **^{19}F NMR** (376 MHz, $CDCl_3$) δ −115.67 – −115.79 (m). **HRMS** (EI) *m/z*: Calculated for $C_{14}H_{12}ClF$ 234.0612 Found 234.0602 $[M]^+$.

2.2) Synthesis of Biaryl Boronic Acid Pinacol Ester Tracks 1–8

2-(4'-Fluoro-[1,1'-biphenyl]-4-yl)-4,4,5,5-tetramethyl-1,3,2-dioxaborolane (1)

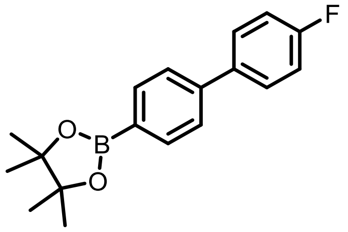

To a solution of **4-bromo-4'-fluoro-1,1'-biphenyl (S1)** (5.02 g, 20.0 mmol, 1.00 eq) in THF (100 mL) at −78 °C was added *n*-BuLi (17.4 mL of 1.26 M in hexanes, 22.2 mmol, 1.10 eq) dropwise, after which the reaction mixture was stirred for 1 h. After this time, 2-isopropoxy-4,4,5,5-tetramethyl-1,3,2-dioxaborolane (4.70 mL, 24.0 mmol, 1.20 eq) was added dropwise and the reaction mixture was allowed to warm to room temperature and stirred for a further 2 h. After this time, the reaction was quenched by the careful addition of saturated aqueous KH_2PO_4 (20 mL). The aqueous phase was extracted with ethyl acetate (3 × 30 mL) and the combined organic extracts were washed with brine (3 × 20 mL), dried over $MgSO_4$, filtered, and concentrated under reduced pressure. The resulting residue was recrystalised from hexane to afford the title compound (5.20 g, 87%) as a white solid.

^{1}H NMR (400 MHz, $CDCl_3$): δ 7.88 (d, *J* = 8.3 Hz, 2H), 7.62 – 7.51 (m, 4H), 7.18 – 7.09 (m, 2H), 1.38 (s, 12H). **^{13}C NMR** (101 MHz, $CDCl_3$) *Note: ^{13}C NMR signals for the aromatic carbon atom adjacent to the boron atom are not observed due to rapid quadrupolar relaxation.* δ 162.7 (d, $^1J_{C–F}$ = 246.7 Hz), 143.0, 137.2 (d, $^4J_{C–F}$ = 3.2 Hz), 135.4, 128.9 (d, $^3J_{C–F}$ = 7.9 Hz), 126.4, 115.8 (d, $^2J_{C–F}$ = 21.3 Hz), 84.0, 25.0. **^{19}F {^{1}H} NMR** (377 MHz, $CDCl_3$) δ −115.31 (s). **IR** ν (cm^{-1}): 3377, 2983, 2924, 1600, 1527, 1356. **HRMS** (EI) *m/z*: Calculated for $C_{18}H_{20}BFO_2$ 298.1540. Found 298.1535 $[M]^+$. **mp**: 105–107 °C.

4,4,5,5-Tetramethyl-2-(4'-(trifluoromethyl)-[1,1'-biphenyl]-4-yl)-1,3,2-dioxaborolane (2)

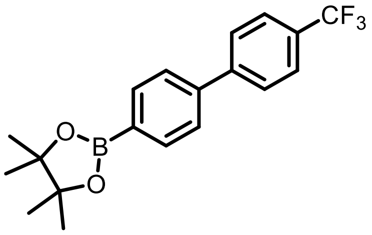

A solution of **4-bromo-4'-(trifluoromethyl)-1,1'-biphenyl (S2)** (1.67 g, 5.55 mmol, 1.00 eq), bis(pinacolato)-diboron (1.68 g, 6.66 mmol, 1.20 eq), potassium acetate (1.09 g, 11.1 mmol, 2.00 eq) and Pd(dppf)Cl_2•CH_2Cl_2 (0.226 g, 5.00 mol%) in 1,4-dioxane (30 mL) was heated to reflux and stirred at this temperature for 16 h. After this time the reaction was allowed to cool to room temperature and diluted with ethyl acetate (20 mL). The mixture was then filtered over Celite®, washing with ethyl acetate (20 mL) and the filtrate was concentrated under reduced pressure. The resulting residue was purified by flash column chromatography on silica gel (hexane, isocratic) to afford the title compound (0.93 g, 48%) as a white solid.

***R*$_f$** (hexane): 0.30. **^{1}H NMR** (400 MHz, $CDCl_3$) δ 7.92 (d, *J* = 8.3 Hz, 2H), 7.74 – 7.67 (m, 4H), 7.61 (d, *J* = 8.3 Hz, 2H), 1.37 (s, 12H). **^{13}C{^{19}F} NMR** (101 MHz, $CDCl_3$) *Note: ^{13}C NMR signals for the aromatic carbon atom adjacent to the boron atom are not observed due to rapid quadrupolar relaxation.* δ 144.7, 142.5, 135.6, 129.7, 127.7, 126.7, 125.9, 124.4, 84.1, 25.0. **^{19}F NMR** (376 MHz, $CDCl_3$) δ −62.30 (s). **IR** ν (cm^{-1}): 2979, 2933, 1611, 1589, 1380. **HRMS** (EI) *m/z*: Calculated for $C_{19}H_{20}BF_3O_2$ 348.1508. Found 348.1496 [M]$^+$. **mp:** 104–109 °C.

2-(4'-Methoxy-[1,1'-biphenyl]-4-yl)-4,4,5,5-tetramethyl-1,3,2-dioxaborolane (3)

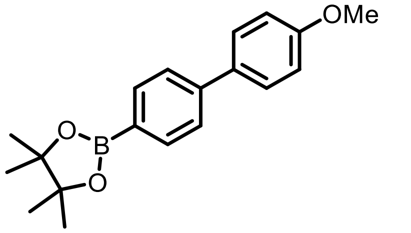

A solution of **4-bromo-4'-(methoxy)-1,1'-biphenyl (S3)** (1.30 g, 4.94 mmol, 1.00 eq), bis(pinacolato)-diboron (1.54 g, 5.95 mmol, 1.20 eq), potassium acetate (0.968 g, 9.88 mmol, 2.00 eq) and Pd(dppf)Cl_2•CH_2Cl_2 (0.202 g, 5.00 mol%) in 1,4-dioxane (30 mL) was heated to reflux and stirred at this temperature for 16 h. After this time the reaction was allowed to cool to room temperature and diluted with ethyl acetate (20 mL). The mixture was then filtered over Celite®, washing with ethyl acetate (20 mL) and the filtrate was concentrated under reduced pressure. The resulting residue was purified by flash column chromatography on silica gel (hexane:EtOAc, 95:5) to afford the title compound (1.22 g, 80%) as a white solid.

***R*f** (hexane): 0.25. **^{1}H NMR** (400 MHz, $CDCl_3$) δ 7.88 – 7.83 (m, 2H), 7.60 – 7.54 (m, 4H), 7.01 – 6.94 (m, 2H), 3.85 (s, 3H), 1.36 (s, 12H). **^{13}C NMR** (126 MHz, $CDCl_3$) *Note: ^{13}C NMR signals for the aromatic carbon atom adjacent to the boron atom are not observed due to rapid quadrupolar relaxation.* δ 159.5, 143.6, 135.4, 133.7, 128.4, 126.1, 114.4, 83.9, 55.5, 25.0. **IR** ν (cm^{-1}): 2976, 2928, 1606, 1583, 1399. **HRMS** (EI) *m/z*: Calculated for $C_{19}H_{23}BO_3$ 310.1740. Found 310.1729 $[M]^+$. **mp:** 128–134 °C.

2-(3'-Methoxy-[1,1'-biphenyl]-4-yl)-4,4,5,5-tetramethyl-1,3,2-dioxaborolane (4)

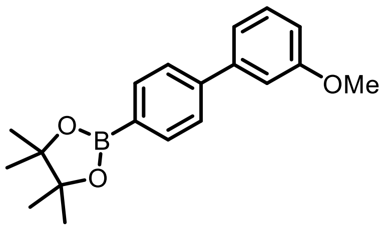

A solution of **4-bromo-3'-(methoxy)-1,1'-biphenyl (S4)** (1.6 g, 6.0 mmol, 1.0 eq), bis(pinacolato)-diboron (1.8 g, 7.2 mmol, 1.2 eq), caesium carbonate (4.2 g, 12 mmol, 2.0 eq), $Pd(OAc)_2$ (0.067 g, 5.0 mol%) and XPhos (0.29 g, 10 mol%) in anhydrous toluene (60 mL) was heated to reflux and stirred at this temperature for 16 h. After this time the reaction was allowed to cool to room temperature and treated with water (20 mL). The aqueous phase was extracted with DCM (3 × 20 mL) and the combined organic phases were washed with brine (3 × 20 mL), dried over $MgSO_4$, filtered, and concentrated under reduced pressure. The resulting residue was purified by flash column chromatography on silica gel (hexane:EtOAc, 95:5) to afford the title compound (0.42 g, 23%) as a colourless oil.

***R*f** (hexane): 0.15. **^{1}H NMR** (400 MHz, $CDCl_3$) δ 7.87 (d, *J* = 8.3 Hz, 2H), 7.59 (d, *J* = 8.3 Hz, 2H), 7.35 (t, *J* = 7.9 Hz, 1H), 7.22 – 7.17 (m, 1H), 7.15 – 7.11 (m, 1H), 6.94 – 6.86 (m, 1H), 3.86 (s, 3H), 1.35 (s, 12H). **^{13}C NMR** (101 MHz, $CDCl_3$) *Note: ^{13}C NMR signals for the aromatic carbon atom adjacent to the boron atom are not observed due to rapid quadrupolar relaxation.* δ 160.0, 143.8, 142.6, 135.3, 129.9, 126.6, 119.9, 113.1, 113.0, 83.9, 55.4, 25.0. **IR** ν (cm^{-1}): 2979, 2865, 2844, 1607, 1587, 1396, 1360. **HRMS** (EI) *m/z*: Calculated for $C_{19}H_{23}BO_3$ 310.1740. Found 310.1727 $[M]^+$.

4,4,5,5-Tetramethyl-2-(4'-methyl-[1,1'-biphenyl]-4-yl)-1,3,2-dioxaborolane (5)

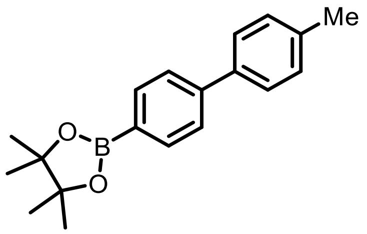

To a solution of **4-bromo-4'-methyl-1,1'-biphenyl (S5)** (1.86 g, 7.56 mmol, 1.00 eq) in THF (80 mL) at −78 °C was added *n*-BuLi (8.07 mL of 1.03 M in hexanes, 8.31 mmol, 1.10 eq) dropwise, after which the reaction mixture was stirred for 30 min. After this time, 2-isopropoxy-4,4,5,5-tetramethyl-1,3,2-dioxaborolane (1.80 mL, 9.07 mmol, 1.20 eq) was added dropwise and the reaction mixture was allowed to warm to room temperature and stirred for a further 2 h. After this time, the reaction was quenched by the careful addition of saturated aqueous KH_2PO_4 (30 mL). The aqueous phase was extracted with ether (3 × 30 mL) and the combined organic extracts were washed with brine (3 × 30 mL), dried over $MgSO_4$, filtered, and concentrated under reduced pressure. The resulting residue was recrystalised from hexane to afford the title compound (1.29 g, 59%) as a white solid.

^{1}H NMR (400 MHz, $CDCl_3$): δ 7.88 (d, *J* = 8.0 Hz, 2H), 7.60 (d, *J* = 8.0 Hz, 2H), 7.53 (d, *J* = 8.0 Hz, 2H), 7.26 (d, *J* = 8.0 Hz, 2H), 2.40 (s, 3H), 1.37 (s, 12H). **^{13}C NMR** (101 MHz, $CDCl_3$) *Note: ^{13}C NMR signals for the aromatic carbon atom adjacent to the boron atom are not observed due to rapid quadrupolar relaxation.* δ 143.8, 138.1, 137.4, 135.2, 129.5, 127.1, 126.3, 83.8, 24.9, 21.1. **IR** ν (cm^{-1}): 2979, 2920, 2844, 1609, 1507. **HRMS** (EI) *m/z*: Calculated for $C_{18}H_{21}BO_2$ 294.1791. Found 294.1778 $[M]^+$. **mp**: 75–77 °C.

2-(4'-Fluoro-2-methyl-[1,1'-biphenyl]-4-yl)-4,4,5,5-tetramethyl-1,3,2-dioxaborolane (6)

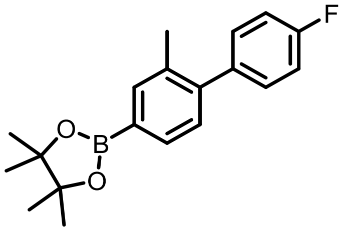

A solution of **4-chloro-4'-fluoro-2-methyl-1,1'-biphenyl (S6)** (0.50 g, 2.3 mmol, 1.0 eq), bis(pinacolato)-diboron (0.70 g, 2.8 mmol, 1.2 eq), potassium acetate (0.45 g, 4.6 mmol, 2.0 eq), $Pd(OAc)_2$ (0.030 g, 5.0 mol%) and XPhos (0.11 g, 10 mol%) in 1,4-dioxane (30 mL) was heated to reflux and stirred at this temperature for 16 h. After this time the reaction was allowed to cool to room temperature and treated with water (20 mL). The aqueous phase was extracted with DCM (3 × 20 mL) and the combined organic phases were washed with brine (3 × 20 mL), dried over $MgSO_4$, filtered, and concentrated under reduced pressure. The resulting residue was purified by flash column chromatography on silica gel (hexane, isocratic) to afford the title compound (0.71 g, 71%) as a white solid.

***R*$_f$** (hexane): 0.20. **^{1}H NMR** (400 MHz, $CDCl_3$) δ 7.73 (s, 1H), 7.68 (d, *J* = 7.5 Hz, 1H), 7.31 – 7.24 (m, 2H), 7.22 (d, *J* = 7.5 Hz, 1H), 7.13 – 7.05 (m, 2H), 2.26 (s, 3H), 1.36 (s, 12H). **^{13}C NMR** (101 MHz, $CDCl_3$) *Note: ^{13}C NMR signals for the aromatic carbon atom adjacent to the boron atom are not observed due to rapid quadrupolar relaxation.* δ 162.1 (d, $^1J_{C\text{–}F}$ = 245.7 Hz), 143.9, 137.8 (d, $^4J_{C\text{–}F}$ = 3.4 Hz), 136.9, 134.8, 132.4, 130.7 (d, $^3J_{C\text{–}F}$ = 7.8 Hz), 129.4, 115.1 (d, $^2J_{C\text{–}F}$ = 21.5 Hz), 83.9, 25.0, 20.3. **^{19}F NMR** (377 MHz, $CDCl_3$) δ −115.67 – −115.86 (m). **IR** ν (cm^{-1}): 2978, 2926, 1611, 1518, 1391, 1358 **HRMS** (EI) *m/z*: Calculated for $C_{19}H_{22}O_2BF$ 312.1697. Found 312.1685 [M^+]. **mp:** 76–79 °C.

2-(4'-Fluoro-2'-methyl-[1,1'-biphenyl]-4-yl)-4,4,5,5-tetramethyl-1,3,2-dioxaborolane (7)

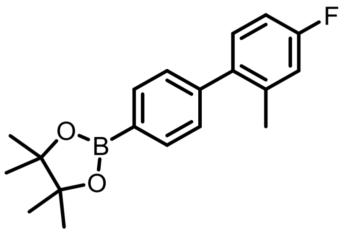

A solution of **4'-bromo-4-fluoro-2-methyl-1,1'-biphenyl (S7)** (0.40 g, 1.5 mmol, 1.0 eq), bis(pinacolato)-diboron (0.45 g, 1.8 mmol, 1.2 eq), potassium acetate (0.30 g, 3.0 mmol, 2.0 eq) and Pd(dppf)Cl_2•CH_2Cl_2 (0.075 g, 5.0 mol%) in 1,4-dioxane (30 mL) was heated to reflux and stirred at this temperature for 16 h. After this time the reaction was allowed to cool to room temperature and treated with water (20 mL). The aqueous phase was extracted with DCM (3 × 20 mL) and the combined organic phases were washed with brine (3 × 20 mL), dried over $MgSO_4$, filtered, and concentrated under reduced pressure. The resulting residue was purified by flash column chromatography on silica gel (hexane, isocratic) to afford the title compound (0.39 g, 83%) as a white solid.

***R*f** (hexane): 0.20. **^{1}H NMR** (400 MHz, $CDCl_3$) δ 7.86 (d, *J* = 8.2 Hz, 2H), 7.30 (d, *J* = 8.2 Hz, 2H), 7.20 – 7.14 (m, 1H), 7.01 – 6.88 (m, 2H), 2.25 (s, 3H), 1.37 (s, 12H). **^{13}C NMR** (101 MHz, $CDCl_3$) *Note: ^{13}C NMR signals for the aromatic carbon atom adjacent to the boron atom are not observed due to rapid quadrupolar relaxation.* δ 162.1 (d, $^1J_{C–F}$ = 245.2 Hz), 144.0, 137.9, 137.7 (d, $^3J_{C–F}$ = 8.1 Hz), 134.7, 131.2 (d, $^3J_{C–F}$ = 8.2 Hz), 128.8, 116.9 (d, $^2J_{C–F}$ = 21.2 Hz), 112.6 (d, $^2J_{C–F}$ = 21.2 Hz), 84.0, 25.0, 20.7. **^{19}F NMR** (377 MHz, $CDCl_3$) δ -115.87 – -116.19 (m). **IR** ν (cm^{-1}): 2977, 2950, 1522, 1352, 1276, 1260. **HRMS** (EI) *m/z*: calculated for $C_{19}H_{22}O_2BF$ 312.1697. Found 312.1686 [M^+]. **mp:** 79–81 °C.

2-(4'-Fluoro-2,2'-dimethyl-[1,1'-biphenyl]-4-yl)-4,4,5,5-tetramethyl-1,3,2-dioxaborolane (8)

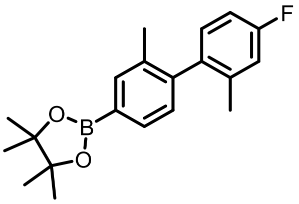

A solution of **4-chloro-4'-fluoro-2,2'-dimethyl-1,1'-biphenyl (S8)** (0.70 g, 3.0 mmol, 1.0 eq), bis(pinacolato)-diboron (0.91 g, 3.6 mmol, 1.2 eq), potassium acetate (0.59 g, 6.0 mmol, 2.0 eq), $Pd(OAc)_2$ (0.035 g, 5.0 mol%) and Xphos (0.14 g, 10 mol%) in 1,4-dioxane (30 mL) was heated to reflux and stirred at this temperature for 16 h. After this time the reaction was allowed to cool to room temperature and treated with water (20 mL). The aqueous phase was extracted with DCM (3 × 20 mL) and the combined organic phases were washed with brine (3 × 20 mL), dried over $MgSO_4$, filtered, and concentrated under reduced pressure. The resulting residue was purified by flash column chromatography on silica gel (hexane, isocratic) to afford the title compound (0.51 g, 52%) as a white solid.

***R*f** (hexane): 0.15. **^{1}H NMR** (400 MHz, $CDCl_3$) δ 7.74 (s, 1H), 7.68 (d, *J* = 7.4 Hz, 1H), 7.10 (d, *J* = 7.5 Hz, 1H), 7.04 (dd, *J* = 8.3, 6.1 Hz, 1H), 6.97 (dd, *J* = 9.8, 2.8 Hz, 1H), 6.94 – 6.88 (m, 1H), 2.06 (s, 3H), 2.03 (s, 3H), 1.37 (s, 12H). **^{13}C NMR** (101 MHz, $CDCl_3$) *Note: ^{13}C NMR signals for the aromatic carbon atom adjacent to the boron atom are not observed due to rapid quadrupolar relaxation.* δ 162.1 (d, $^1J_{C–F}$ = 244.8 Hz), 143.8, 138.2 (d, $^3J_{C–F}$ = 7.9 Hz), 137.4 (d, $^4J_{C–F}$ = 3.1 Hz), 136.4, 135.5, 132.2, 130.5 (d, $^3J_{C–F}$ = 8.2 Hz), 129.2, 116.5 (d, $^2J_{C–F}$ = 20.9 Hz), 112.5 (d, $^2J_{C–F}$ = 20.9 Hz), 83.9, 25.0, 20.0 (d, $^4J_{C–F}$ = 1.6 Hz), 19.7. **^{19}F NMR {^{1}H}** (377 MHz, $CDCl_3$) δ −116.31 (s). **IR** ν (cm^{-1}): 2980, 2895, 1359, 1274, 1146, 1065. **HRMS** (EI) *m/z*: Calculated for $C_{20}H_{24}O_2BF$ 326.1853. Found 326.1848 [M^+]. **mp:** 96–100 °C.

2.3) Synthesis of Bis-Ortho-Deutero Biaryl Boronic Acid Pinacol Ester Track D_2-1

4-Fluorobenzen-2,6-d$_2$-amine (D_2-S1a)

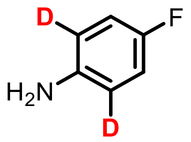

A solution of HCl (4.0 M in dioxane, 7.5 mL, 30 mmol, 1.0 eq) was added slowly to a stirred solution of 4-fluoroaniline (2.8 mL, 30 mmol, 1.0 eq) in anhydrous ether (30 mL). After stirring for a further 15 minutes the volatiles were removed under reduced pressure. The resulting ammonium salt was transferred to a thick-walled pressure tube and deuterium oxide (15 mL) was added. The tube was sealed and stirred at 135 °C for 24 h. After this time, the reaction was allowed to cool to room temperature and was treated with NaOH solution (2 M, 20 mL). The reaction mixture was then extracted with ether (3 × 30 mL) and the combined organic phases were dried over $MgSO_4$, filtered, and concentrated under reduced pressure. 1H NMR analysis of the crude reaction mixture indicated incomplete deuterium incorporation. The reaction mixture was thus then re-subjected to the reaction conditions for a further After repeating the procedure for a second time, leaving under reaction conditions for 48 h, the crude 48 h. After this time the resulting residue was purified by distillation under reduced pressure (heating at 100 °C) to afford the title compound (0.97 g, 29%, >97% D incorporation by ^{1}H NMR spectroscopic analysis) as a colourless oil.

^{1}H NMR (400 MHz, $CDCl_3$): δ 6.85 (d, J = 8.6 Hz, 2H), 6.61 (m, 0.06H), 3.31 (br. s, 2H). **^{13}C NMR** (101 MHz, $CDCl_3$) δ 156.5 (d, $^1J_{C–F}$ = 235.2 Hz), 142.3 (d, $^4J_{C–F}$ = 2.1 Hz), 116.3 – 115.8 (m), 115.6 (d, $^2J_{C–F}$ = 22.4 Hz). **^{19}F NMR {^{1}H}** (376 MHz, $CDCl_3$): δ −126.83 (s). **HRMS** (ESI) *m/z*: Calculated for $C_6H_4D_2FN$ 113.0610. Found 114.0678 $[M + H]^+$. Data in accordance with the literature.[1]

1-Bromo-4-fluorobenzene-2,6-d$_2$ (D$_2$-S1b)

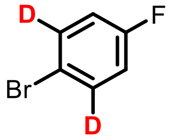

A solution of HBr (48 wt% in water, 5 mL) was added dropwise to a stirred suspension of **4-fluorobenzen-2,6-d$_2$-amine (D$_2$-S1a)** (0.950 g, 8.40 mmol, 1.00 eq) in water (4 mL) at room temperature. After stirring for a further 15 minutes at room temperature, the reaction was cooled to –5 °C using an ice/salt bath and a solution of sodium nitrite (0.607 g, 8.82 mmol, 1.05 eq) in water (4 mL) was added dropwise, with the internal reaction temperature maintained below 0 °C. After stirring at < 0 °C for 30 min, the reaction mixture was added dropwise to a rapidly stirred suspension of CuBr (1.59 g, 11.0 mmol, 1.30 eq) in HBr (48 wt% in water, 10 mL) at room temperature. The reaction was then heated to 50 °C and stirred at this temperature until no further effervescence was observed. After this the reaction was cooled to room temperature and extracted with pentane (3 × 50 mL). The combined organic phases were washed with saturated aqueous $NaHCO_3$ (30 mL), aqueous sodium thiosulfate solution (10% wt., 30 mL), dried over $MgSO_4$, filtered, and concentrated under reduced pressure. The resulting pentane solution was not reduced to dryness due to the volatility of the compound and the title compound was identified by ^{1}H NMR and HRMS as a solution in pentane.

^{1}H NMR (400 MHz, $CDCl_3$): δ 7.51 – 7.43 (m, 0.06H), 6.98 (d, *J* = 7.1 Hz, 2H). **^{19}F NMR {^{1}H}** (376 MHz, $CDCl_3$): δ −115.34 (s). **HRMS** (EI) *m/z*: Calculated for $C_6H_2D_2FBr$ 175.9606. Found 175.9599 $[M]^+$. Data in accordance with the literature.[1]

(4-Fluorophenyl-2,6-d$_2$)boronic acid (D$_2$-S1c)

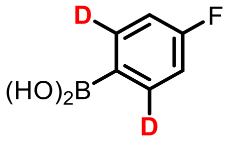

To a solution of **1-bromo-4-fluorobenzene-2,6-d$_2$ (D$_2$-S1b)** (1.2 g, 6.8 mmol, 1.0 eq) in THF (60 mL) at −78 °C was added *n*-BuLi (5.6 mL of 1.32 M in hexanes, 7.5 mmol, 1.1 eq) dropwise and the reaction was stirred at −78 °C for 2 h. After this time, triisopropyl borate (1.9 mL, 8.2 mmol, 1.2 eq) was added dropwise and the reaction mixture was allowed to warm to room temperature and stirred for a further 16 h. After this time, the reaction was quenched by the careful addition of 1M HCl (30 mL). The aqueous phase was extracted with ethyl acetate (3 × 30 mL) and the combined organic phases were washed with brine (3 × 20 mL), dried over $MgSO_4$, filtered, and concentrated under reduced pressure. The resulting off white solid was treated with 1M NaOH (20 mL) and the resulting aqueous solution was washed with chloroform (20 mL) and then acidified with 1M HCl (30 mL). The aqueous phase was then extracted with ethyl acetate (3 × 20 mL) and the combined organic phases were washed with brine (3 × 30 mL), dried over $MgSO_4$, filtered, and concentrated under reduced pressure to afford the title compound (0.68 g, 68%, >97% D incorporation by ^{1}H NMR spectroscopic analysis) as a white solid.

^{1}H NMR (400 MHz, $(CD_3)_2SO$) δ 8.11 (s, 2H), 7.88 – 7.79 (m, 0.05H), 7.13 (d, J = 9.6 Hz, 2H). **^{13}C NMR** (101 MHz, $(CD_3)_2SO$) *Note: ^{13}C NMR signals for the aromatic carbon atom adjacent to the boron atom are not observed due to rapid quadrupolar relaxation.* δ 163.8 (d, $^{1}J_{C\text{–}F}$ = 245.6 Hz), 137.0 – 136.5 (m), 114.2 (d, $^{2}J_{C\text{–}F}$ = 19.7 Hz). **^{19}F NMR** (376 MHz, $CDCl_3$): δ −110.8 (t). **IR** ν (cm^{-1}): 3205, 2927, 1579, 1469. **HRMS** (Nanospray, negative ion) *m/z*: Calculated for $C_6H_4D_2BFO_2$ 142.0570. Found 177.0262 $[M + Cl]^-$. **mp**: >250 °C (max apparatus).

4-Bromo-4'-fluoro-1,1'-biphenyl-2',6'-d$_2$ (D$_2$-S1)

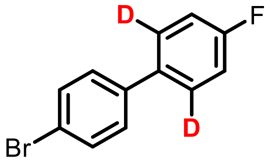

A solution of 1-bromo-4-iodobenzene (0.85 g, 3.0 mmol, 1.0 eq), **(4-fluorophenyl-2,6-d$_2$)boronic acid (D$_2$-S1c)** (0.54 g, 3.6 mmol, 1.2 eq), tetrakis(triphenylphosphine)palladium(0) (0.10 g, 3.0 mol%) and potassium carbonate (0.83 g, 6.0 mmol, 2.0 eq) in toluene: ethanol: water (40 mL, 8:1:1) was heated to reflux and stirred at this temperature for 5 h. After this time the reaction mixture was cooled to room temperature and poured over water (30 mL). The aqueous phase was then extracted with ether (3 × 30 mL) and the combined organic phases were washed with brine (3 × 20 mL), dried over $MgSO_4$, filtered, and concentrated under reduced pressure. The resulting residue was purified by flash column chromatography on silica gel (hexane, isocratic) to afford the title compound (0.18 g, 23%, >97% D incorporation by ^{1}H NMR spectroscopic analysis) as a white solid.

***R*$_f$** (hexane): 0.33. **^{1}H NMR** (400 MHz, $CDCl_3$) δ 7.59 – 7.51 (m, 2H), 7.44 – 7.37 (m, 2H), 7.13 (d, *J* = 8.6 Hz, 2H). **^{13}C NMR** (101 MHz, $CDCl_3$) δ 162.9 (d, $^1J_{C–F}$ = 246.9 Hz), 139.3, 136.2 (d, $^4J_{C–F}$ = 3.3 Hz), 132.2, 128.8 (m), 128.5 (m), 121.7, 115.9 (d, $^2J_{C–F}$ = 21.5 Hz). **^{19}F NMR {^{1}H}** (376 MHz, $CDCl_3$): δ −115.03 (s). **IR** ν (cm^{-1}): 2921, 2851, 1583, 1453. **HRMS** (EI) *m/z*: Calculated for $C_{12}H_6D_2BrF$ 251.9919. Found 251.9914 $[M]^+$. **mp**: 96–98 °C.

2-(4'-Fluoro-[1,1'-biphenyl]-4-yl-2',6'-d$_2$)-4,4,5,5-tetramethyl-1,3,2-dioxaborolane (D$_2$-1)

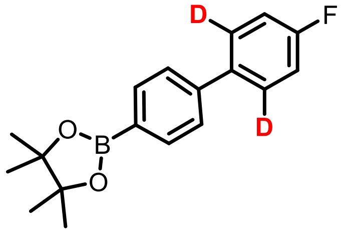

To a solution of **4-bromo-4'-fluoro-1,1'-biphenyl-2',6'-d$_2$ (D$_2$-S1)** (0.11 g, 0.43 mmol, 1.0 eq) in THF (15 mL) at −78 °C was added *n*-BuLi (0.44 mL of 1.06 M in hexanes, 0.47 mmol, 1.1 eq) dropwise, after which the reaction mixture was stirred for 30 min at −78 °C. After this time, 2-isopropoxy-4,4,5,5-tetramethyl-1,3,2- dioxaborolane (0.10 mL, 0.51 mmol, 1.2 eq) was added dropwise and the reaction mixture was allowed to warm to room temperature and stirred for a further 2 h. After this time, the reaction was quenched by the careful addition of saturated aqueous KH_2PO_4 (30 mL). The aqueous phase was extracted with ether (3 × 10 mL) and the combined organic extracts were washed with brine (3 × 10 mL), dried over $MgSO_4$, filtered, and concentrated under reduced pressure. The resulting residue was purified by flash column chromatography on silica gel (hexane:EtOAc, gradient 100:0–97:3) to afford the title compound (0.090 g, 67%, >97% D incorporation by ^{1}H NMR spectroscopic analysis) as a white solid.

***R*$_f$** (hexane): 0.10. **^{1}H NMR** (400 MHz, $CDCl_3$) δ 7.89 (d, *J* = 8.3 Hz, 2H), 7.56 (d, *J* = 8.3 Hz, 2H), 7.13 (d, *J* = 8.7 Hz, 2H), 1.37 (s, 12H). **^{13}C NMR** (101 MHz, $CDCl_3$) *Note: ^{13}C NMR signals for the aromatic carbon atom adjacent to the boron atom are not observed due to rapid quadrupolar relaxation.* δ 162.8 (d, $^{1}J_{C\text{–}F}$ = 247.4 Hz), 142.9, 137.1 (d, $^{4}J_{C\text{–}F}$ = 3.2 Hz), 135.4, 128.7 (m), 126.4, 115.7 (d, $^{2}J_{C\text{–}F}$ = 21.3 Hz), 84.0, 25.0. **^{19}F{^{1}H} NMR** (376 MHz, $CDCl_3$) δ −115.34 (s). **IR** ν (cm^{-1}): 2979, 2925, 1609, 1585, 1459. **HRMS** (EI) *m/z*: Calculated for $C_{18}H_{18}D_2BFO_2$ 300.1666. Found 300.1650 $[M]^+$. **mp**: 105–108 °C.

2.4) Synthesis of Triaryl Boronic Acid Pinacol Ester Track 9

3-Bromo-4'-fluoro-1,1'-biphenyl (S9a)

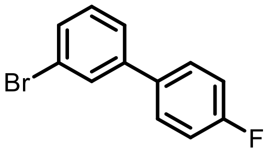

A solution of 1-bromo-3-iodobenzene (6.2 g, 22 mmol, 1.1 eq), 4-fluorophenylboronic acid (2.8 g, 20 mmol, 1.0 eq), tetrakis(triphenylphosphine)palladium(0) (0.71 g, 3.0 mol%) and potassium carbonate (5.5 g, 24 mmol, 2.0 eq) in toluene: ethanol: water (150 mL, 8:1:1) was heated to reflux and stirred at this temperature for 16 h. After this time the reaction mixture was cooled to room temperature and poured over water (30 mL). The aqueous phase was then extracted with ethyl acetate (3 × 30 mL) and the combined organic phases were washed with brine (3 × 20 mL), dried over $MgSO_4$, filtered, and concentrated under reduced pressure. The resulting residue was purified by flash column chromatography on silica gel (hexane, isocratic) to afford the title compound (5.0 g, 99%) as a yellow oil.

***R*_f** (hexane): 0.35. **^{1}H NMR** (400 MHz, $CDCl_3$) δ 7.59 (s, 1H), 7.42 – 7.31 (m, 4H), 7.22 – 7.15 (m, 1H), 7.07 – 6.98 (m, 2H). **^{13}C NMR** (101 MHz, $CDCl_3$) δ 162.9 (d, $^{1}J_{C–F}$ = 247.3 Hz), 142.4, 135.9 (d, $^{4}J_{C–F}$ = 3.2 Hz), 130.5, 130.4, 130.2, 128.9 (d, $^{3}J_{C–F}$ = 8.2 Hz), 125.8, 123.1, 116.0 (d, $^{2}J_{C–F}$ = 21.6 Hz). **^{19}F NMR** (376 MHz, $CDCl_3$) δ −114.20 – −114.33 (m). **IR** ν (cm^{-1}): 2925, 2586, 2445, 1737, 1605, 1595. **HRMS** (EI) *m/z*: Calculated for $C_{12}H_8BrF$ 249.9793. Found 249.9783 $[M]^+$.

(4'-Fluoro-[1,1'-biphenyl]-3-yl)boronic acid (S9b)

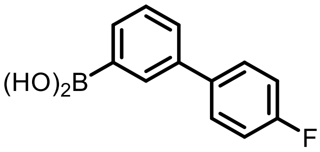

To a solution of **3-Bromo-4'-fluoro-1,1'-biphenyl (S9a)** (5.00 g, 20.0 mmol, 1.00 eq) in THF (150 mL) at −78 °C was added *n*-BuLi (16.9 mL of 1.30 M in hexanes, 22.0 mmol, 1.10 eq) dropwise. The reaction was stirred for 30 min at −78 °C, after which, triisopropyl borate (0.52 mL, 2.50 mmol, 1.20 eq) was added dropwise. The reaction was allowed to warm to room temperature and stirred for 3 h. After this time the reaction was quenched by the careful addition of 1 M HCl (20 mL). The aqueous phase was extracted with ethyl acetate (3 × 20 mL) and the combined organic extracts were washed with brine (3 × 10 mL), dried over $MgSO_4$, filtered, and concentrated under reduced pressure to give an off white solid which was then washed with ice cold hexane to afford the title compound (4.38 g, quant.) as a white solid.

^{1}H NMR (400 MHz, $(CD_3)_2SO$) δ 8.16 (s, 2H), 8.08 (s, 1H), 7.77 (d, J = 7.3 Hz, 1H), 7.73 – 7.64 (m, 3H), 7.45 – 7.39 (m, 1H), 7.34 – 7.25 (m, 2H). **^{13}C NMR** (101 MHz, $(CD_3)_2SO$) *Note: ^{13}C NMR signals for the aromatic carbon atom adjacent to the boron atom are not observed due to rapid quadrupolar relaxation.* δ 161.8 (d, $^{1}J_{C\text{–}F}$ = 243.9 Hz), 138.1, 137.1 (d, $^{4}J_{C\text{–}F}$ = 3.2 Hz), 133.2, 132.4, 128.6 (d, $^{3}J_{C\text{–}F}$ = 8.1 Hz), 128.3, 128.1, 115.7 (d, $^{2}J_{C\text{–}F}$ = 21.3 Hz). **^{19}F NMR** (376 MHz, $(CD_3)_2SO$) δ −115.60 – −115.92 (m). **IR** ν (cm^{-1}): 3202, 2970, 2952, 1736, 1512, 1365. **HRMS** (Negative ion, nanospray) *m/z*: Calculated for $C_{12}H_{10}BO_2F$ 216.0758. Found 251.0442 $[M + Cl]^{-}$. **mp:** 147–157 °C.

4-Bromo-4''-fluoro-1,1':3',1''-terphenyl (S9c)

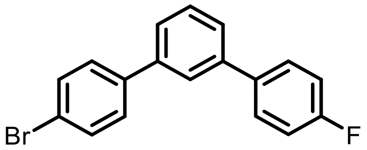

A solution of 1-bromo-4-iodobenzene (2.75 g, 9.72 mmol, 1.05 eq), **(4'-fluoro-[1,1'-biphenyl]-3-yl)boronic acid (S9b)** (2.00 g, 9.26 mmol, 1.00 eq), tetrakis(triphenylphosphine)palladium(0) (0.534 g, 5.00 mol%) and potassium carbonate (2.55 g, 18.5 mmol, 2.00 eq) in toluene: ethanol: water (80 mL, 8:1:1) was heated to reflux and stirred at this temperature for 16 h. After this time the reaction mixture was cooled to room temperature and poured over water (30 mL). The aqueous phase was then extracted with ethyl acetate (3 × 30 mL) and the combined organic phases were washed with brine (3 × 20 mL), dried over $MgSO_4$, filtered, and concentrated under reduced pressure. The resulting residue was purified by flash column chromatography on silica gel (hexane, isocratic) to afford the title compound (1.17 g, 39%) as a white solid.

***R*f** (hexane): 0.50. **^{1}H NMR** (400 MHz, $CDCl_3$) δ 7.70 (s, 1H), 7.63 – 7.55 (m, 4H), 7.55 – 7.48 (m, 5H), 7.19 – 7.12 (m, 2H). **^{13}C NMR** (101 MHz, $CDCl_3$) δ 162.7 (d, $^1J_{C\text{–}F}$ = 246.7 Hz), 141.1, 140.8, 140.1, 137.2, 132.1, 129.5, 128.9, 128.9 (d, $^3J_{C\text{–}F}$ = 7.9 Hz), 126.5, 126.0, 125.9, 121.9, 115.8 (d, $^2J_{C\text{–}F}$ = 21.5 Hz). **^{19}F NMR** (376 MHz, $CDCl_3$) δ −115.14 – −115.27 (m). **IR** ν (cm^{-1}): 3029, 2969, 2929, 1726, 1605, 1512, 1478. **HRMS** (EI) *m/z*: Calculated for $C_{18}H_{12}BrF$ 326.0106. Found 326.0094 $[M]^+$. **mp:** 105–106 °C.

2-(4''-Fluoro-[1,1':3',1''-terphenyl]-4-yl)-4,4,5,5-tetramethyl-1,3,2-dioxaborolane (9)

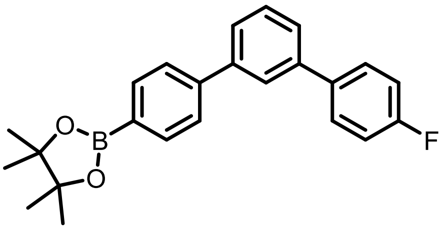

To a solution of **4-bromo-4''-fluoro-1,1':3',1''-terphenyl (S9c)** (1.17 g, 3.66 mmol, 1.00 eq) in THF (30 mL) at −78 °C was added *n*-BuLi (3.1 mL of 1.30 M in hexanes, 4.04 mmol, 1.10 eq) dropwise. The reaction was stirred for 30 min at −78 °C, after which, 2-isopropoxy-4,4,5,5-tetramethyl-1,3,2-dioxaborolane (0.90 mL, 4.40 mmol, 1.20 eq) was added dropwise. The reaction was allowed to warm to room temperature and stirred for 3 h. After this time, the reaction was quenched by the careful addition of saturated aqueous KH_2PO_4 (10 mL). The aqueous phase was extracted with ethyl acetate (3 × 20 mL) and the combined organic phases were washed with brine (3 × 10 mL), dried over $MgSO_4$, filtered, and concentrated under reduced pressure. The resulting residue was triturated using hexane to afford the title compound(1.37 g, quant.) as a waxy solid.

^{1}H NMR (400 MHz, $CDCl_3$) δ 7.92 (d, *J* = 8.2 Hz, 2H), 7.77 (s, 1H), 7.66 (d, *J* = 8.2 Hz, 2H), 7.64 – 7.56 (m, 3H), 7.58 – 7.43 (m, 2H), 7.15 (apparent t, *J* = 8.6 Hz, 2H), 1.38 (s, 12H). **^{13}C NMR** (101 MHz, $CDCl_3$) *Note: ^{13}C NMR signals for the aromatic carbon atom adjacent to the boron atom are not observed due to rapid quadrupolar relaxation.* δ 162.7 (d, $^1J_{C–F}$ = 246.4 Hz), 143.8, 141.8, 141.0, 137.4 (d, $^4J_{C–F}$ = 3.2 Hz), 135.4, 129.4, 128.9 (d, $^3J_{C–F}$ = 8.2 Hz), 126.6, 126.4, 126.3, 126.1, 115.79 (d, $^2J_{C–F}$ = 21.6 Hz), 84.0, 25.0. **^{19}F NMR** (376 MHz, $CDCl_3$) δ −115.24 – −115.56 (m). **IR** ν (cm^{-1}): 2977, 2924, 2879, 1736, 1607, 1512, 1360. **HRMS** (negative ion, MALDI) *m/z*: Calculated for $C_{24}H_{24}BFO_2$ 374.1853. Found 373.1789 $[M − H]^−$.

2.5) Synthesis of Bridged Biaryl Boronic Acid Pinacol Ester Tracks 10–15

(4-Bromophenyl)(4-fluorophenyl)methanol (S10a)

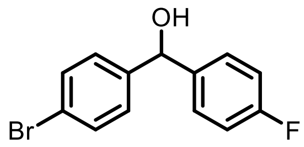

To a solution of 4-bromobenzaldehyde (3.70 g, 20.0 mmol, 1.00 eq) in THF (20 mL) at 0 °C, was added 4-fluorophenyl magnesium bromide (2.00 M in Et_2O, 11.0 mL, 22.0 mmol, 1.10 eq) dropwise. The reaction was then stirred at 0 °C for 1 h and then quenched with the careful addition of saturated NH_4Cl (30 mL) at 0 °C. The aqueous phase was extracted with ethyl acetate (3 × 20 mL) and the combined organic phases were washed with brine (2 × 20 mL), dried over $MgSO_4$, filtered, and concentrated under reduced pressure. The resulting yellow oil was purified via flash column chromatography on silica gel (hexane:EtOAc, 80:20) to afford the title compound (4.47 g, 80%) as a colourless oil.

***R*f** (hexane:EtOAc, 80:20): 0.25. **^{1}H NMR** (400 MHz, $CDCl_3$) δ 7.48 – 7.43 (m, 2H), 7.33 – 7.27 (m, 2H), 7.27 – 7.20 (m, 2H), 7.01 (m, 2H), 5.77 (d, *J* = 3.4 Hz, 1H), 2.28 (d, *J* = 3.4 Hz, 1H). **^{13}C NMR** (101 MHz, $CDCl_3$) δ 162.5 (d, $^1J_{C\text{–}F}$ = 246.5 Hz), 142.7, 139.3 (d, $^4J_{C\text{–}F}$ = 3.0 Hz), 131.8, 128.4 (d, $^3J_{C\text{–}F}$ = 8.0 Hz), 128.3, 121.7, 115.6 (d, $^2J_{C\text{–}F}$ = 21.5 Hz), 75.1. **^{19}F NMR** (377 MHz, $CDCl_3$) δ −113.9 – −114.6 (m). **IR** ν (cm^{-1}): 3341, 2966, 2922, 2866, 2844, 1603, 1506, 1222. **HRMS** (APCI) *m/z*: Calculated for $C_{13}H_{10}BrFO$ 279.9899. Found 279.9894 $[M]^+$.

1-Bromo-4-(4-fluorobenzyl)benzene (S10b)

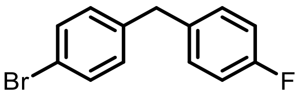

Sodium borohydride (0.434 g, 11.5 mmol, 2.50 eq) was added portionwise to trifluoroacetic acid (6 mL) at 0 °C, followed by the dropwise addition of a solution of (**4-bromophenyl)(4-fluorophenyl)methanol (S10a)** (1.29 g, 4.61 mmol, 1.00 eq) in DCM (25 mL). The reaction was monitored by ^{1}H and ^{19}F NMR spectroscopy and after stirring for 20 h an additional equivalent of sodium borohydride (0.174 g, 4.61 mmol, 1.00 eq) was added and the reaction mixture was stirred for a further 1.5 h. After this time, the reaction mixture was subject to the slow addition of aqueous NaOH solution (2 M) until pH 7 was reached. The aqueous phase was then extracted with ether (3 × 20 mL) and the combined organic phases were washed with brine (2 × 20 mL), dried over $MgSO_4$, filtered, and concentrated under reduced pressure. The resulting orange oil was purified via flash column chromatography on silica gel (hexane, isocratic) to afford the title compound (0.90 g, 73%) as a viscous colourless oil.

$\boldsymbol{R_f}$ (hexane): 0.33. **^{1}H NMR** (400 MHz, $CDCl_3$) 7.46 – 7.38 (m, 2H), 7.17 – 7.08 (m, 2H), 7.05 (d, J = 8.5 Hz, 2H), 7.02 – 6.94 (m, 2H), 3.91 (s, 2H). **^{13}C NMR** (101 MHz, $CDCl_3$) 161.7 (d, $^{1}J_{C\text{–}F}$ = 244.5 Hz), 140.1, 136.2 (d, $^{4}J_{C\text{–}F}$ = 3.3 Hz), 131.7, 130.7, 130.4 (d, $^{3}J_{C\text{–}F}$ = 7.9 Hz), 120.2, 115.5 (d, $^{2}J_{C\text{–}F}$ = 21.1 Hz), 40.6. **^{19}F NMR {^{1}H}** (376 MHz, $CDCl_3$) δ −116.85 (s). **IR** ν (cm^{-1}): 2923, 2855, 1680, 1602, 1507, 1486, 1220, 1156. **HRMS** (EI) *m/z*: Calculated for $C_{13}H_{10}BrF$ 263.9950. Found 263.9944 $[M]^+$.

2-(4-(4-Fluorobenzyl)phenyl)-4,4,5,5-tetramethyl-1,3,2-dioxaborolane (10)

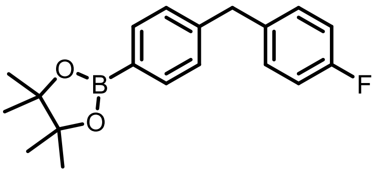

To a solution of **1-bromo-4-(4-fluorobenzyl)benzene (S29b)** (4.10 g, 15.5 mmol, 1.00 eq) in THF (80 mL) at −78 °C was added *n*-BuLi (12.5 mL of 1.37 M in hexanes, 17.1 mmol, 1.10 eq) dropwise, after which the reaction mixture was stirred for 2 h. After this time, 2-isopropoxy-4,4,5,5-tetramethyl-1,3,2-dioxaborolane (3.80 mL, 18.7 mmol, 1.20 eq) was added dropwise and the reaction mixture was allowed to warm to room temperature and stirred for a further 16 h. After this time, the reaction was quenched by the careful addition of saturated aqueous KH_2PO_4 (30 mL). The aqueous phase was extracted with ether (3 × 30 mL) and the combined organic extracts were washed with brine (3 × 30 mL), dried over $MgSO_4$, filtered, and concentrated under reduced pressure. The resulting yellow oil was purified via flash column chromatography on silica gel (hexane:EtOAc, 95:5) and the resultant white solid was then washed with ice-cold hexane and filtered to afford the title compound(3.62 g, 74%) as a white solid.

***R*f** (hexane:EtOAc, 95:5): 0.40. **^{1}H NMR** (400 MHz, $CDCl_3$) δ 7.74 (d, *J* = 8.0 Hz, 2H), 7.18 (d, *J* = 8.0 Hz, 2H), 7.15 – 7.08 (m, 2H), 7.00 – 6.90 (m, 2H), 3.96 (s, 2H), 1.33 (s, 12H). **^{13}C NMR** (101 MHz, $CDCl_3$) *Note: ^{13}C NMR signals for the aromatic carbon atom adjacent to the boron atom are not observed due to rapid quadrupolar relaxation.* δ 161.5 (d, $^1J_{C–F}$ = 243.8 Hz), 144.3, 136.7, 135.2, 130.4 (d, $^3J_{C–F}$ = 7.9 Hz), 128.4, 115.3 (d, $^2J_{C–F}$ = 21.2 Hz), 83.8, 41.4, 25.0. **^{19}F NMR** (377 MHz, $CDCl_3$) δ−117.07 – −117.88 (m). **IR** ν (cm^{-1}): 3044, 2979, 2916, 1611, 1507, 1390, 1357. **HRMS** (EI) *m/z*: Calculated for $C_{19}H_{22}BFO_2$ 312.1697. Found 312.1690 [M]$^+$. **mp**: 73–75 °C.

(4-Bromophenyl)(4'-fluoro-[1,1'-biphenyl]-4-yl)methanol (S11a)

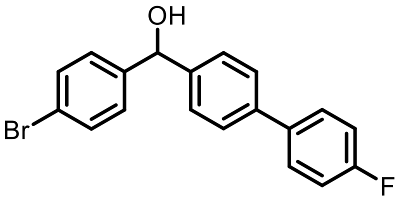

To a solution of **4-bromo-4'-fluoro-1,1'-biphenyl (S1)** (3.77 g, 15.0 mmol, 1.00 eq) in THF (75 mL) at −78 °C was added *n*-BuLi (12.8 mL of 1.23 M in hexanes, 15.8 mmol, 1.05 eq) dropwise. The reaction mixture was stirred for 1 h at −78 °C, after which time, a solution of 4-bromobenzaldehyde (3.05 g, 16.5 mmol, 1.10 eq) in THF (25 ml) was added dropwise. The reaction was allowed to warm to room temperature and stirred overnight. The reaction was then quenched by the careful addition of 1M HCl (30 mL). The aqueous phase was extracted with ethyl acetate (3 × 30 mL) and the combined organic phases were washed with brine (3 × 20 mL), dried over $MgSO_4$, filtered, and concentrated under reduced pressure. The resulting residue was purified via flash column chromatography on silica gel (hexane:EtOAc, 90:10) to provide the title compound (2.11 g, 39%) as a viscous oil.

***R*f** (hexane:EtOAc, 90:10): 0.25. **^{1}H NMR** (400 MHz, $CDCl_3$) δ 7.57 – 7.44 (m, 6H), 7.43 – 7.38 (m, 2H), 7.38 – 7.25 (m, 2H), 7.17 – 7.06 (m, 2H), 5.85 (s, 1H). **^{13}C NMR** (101 MHz, $CDCl_3$) δ 162.7 (d, $^{1}J_{C–F}$ = 246.8 Hz), 142.9, 142.6, 140.1, 137.0, 131.9, 128.8 (d, $^{3}J_{C–F}$ = 8.0 Hz), 128.4, 127.5, 127.2, 121.8, 115.9 (d, $^{2}J_{C–F}$ = 21.5 Hz), 75.6. **^{19}F NMR {^{1}H}** (376 MHz, $CDCl_3$) δ −115.49 (s). **IR** ν (cm^{-1}): 3365, 3028, 2922, 2857, 1602, 1495, 1485, 1231. **HRMS** (EI) *m/z*: Calculated for $C_{19}H_{14}BrFO$ 356.0212. Found 356.0200 $[M]^{+}$.

4-(4-Bromobenzyl)-4'-fluoro-1,1'-biphenyl (S11b)

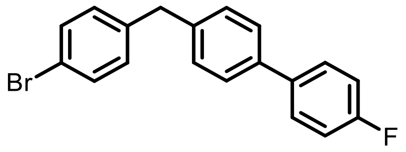

Sodium borohydride (0.670 g, 17.7 mmol, 3.00 eq) was added portionwise to trifluoroacetic acid (10 mL) at 0 °C, followed by the dropwise addition of a solution of **(4-bromophenyl)(4'-fluoro-[1,1'-biphenyl]-4-yl)methanol (S11a)** (2.11 g, 5.90 mmol, 1.00 eq) in DCM (20 mL). After stirring for 6 h the reaction mixture was subject to the slow addition of aqueous NaOH solution (2 M) until pH 7 was reached. The aqueous phase was then extracted with DCM (3 × 30 mL) and the combined organic phases were washed with brine (2 × 20 mL), dried over $MgSO_4$, filtered, and concentrated under reduced pressure. The resulting residue was purified via flash column chromatography on silica gel (hexane:toluene, 95:5) to afford the title compound (1.00 g, 50%) as a white solid.

***R*f** (hexane): 0.20. **^{1}H NMR** (400 MHz, $CDCl_3$) δ 7.61 – 7.39 (m, 6H), 7.27 – 7.20 (m, 2H), 7.18 – 7.06 (m, 4H), 3.99 (s, 2H). **^{13}C NMR** (101 MHz, $CDCl_3$) δ 162.4 (d, $^{1}J_{C–F}$ = 245.9 Hz), 139.9, 139.6, 138.3, 137.0, 131.6, 130.7, 129.3, 128.5 (d, $^{3}J_{C–F}$ = 7.9 Hz), 127.2, 120.1, 115.6 (d, $^{2}J_{C–F}$ = 21.6 Hz), 40.9. **^{19}F NMR {^{1}H}** (376 MHz, $CDCl_3$) δ − 74.94 (s, TFA), − 115.92 (s). **IR** ν (cm^{-1}): 3026, 2949, 2919, 2852, 1901, 1744, 1599, 1494, 1485. **HRMS** (EI) *m/z*: Calculated for $C_{19}H_{14}BrF$ 340.0263. Found 340.0252 $[M]^{+}$. **mp**: 89–92 °C

2-(4-((4'-Fluoro-[1,1'-biphenyl]-4-yl)methyl)phenyl)-4,4,5,5-tetramethyl-1,3,2-dioxaborolane (11)

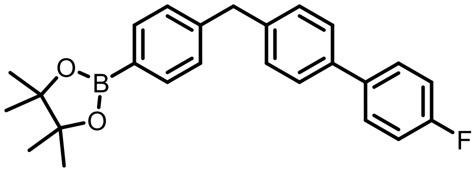

To a solution of **4-(4-bromobenzyl)-4'-fluoro-1,1'-biphenyl (S11b)** (0.93 g, 2.7 mmol, 1.0 eq) in THF (30 mL) at −78 °C was added *n*-BuLi (2.4 mL of 1.23 M in hexanes, 3.0 mmol, 1.1 eq) dropwise. The reaction was stirred for 30 min at −78 °C, after which time, 2-isopropoxy-4,4,5,5-tetramethyl-1,3,2-dioxaborolane (0.65 mL, 3.3 mmol, 1.2 eq) was added dropwise. The reaction was allowed to warm to room temperature and stirred for 3 h. After this time, the reaction was quenched by the careful addition of saturated aqueous KH_2PO_4 (30 mL). The aqueous phase was extracted with ethyl acetate (3 × 20 mL) and the combined organic phases were washed with brine (2 × 20 mL), dried over $MgSO_4$, filtered, and concentrated under reduced pressure. The resulting residue was purified by flash column chromatography on silica gel (hexane, isocratic) to afford the title compound (1.00 g, 94%) as a white solid.

***R*$_f$** (hexane): 0.15. **^{1}H NMR** (400 MHz, $CDCl_3$) δ 7.84 (d, *J* = 8.3 Hz, 2H), 7.58 – 7.53 (m, 2H), 7.49 (d, *J* = 8.4 Hz, 2H), 7.33 – 7.26 (m, 4H), 7.18 – 7.11 (m, 2H), 4.07 (s, 2H), 1.39 (s, 12H). **^{13}C NMR** (101 MHz, $CDCl_3$) *Note: ^{13}C NMR signals for the aromatic carbon atom adjacent to the boron atom are not observed due to rapid quadrupolar relaxation.* δ 162.4 (d, $^1J_{C–F}$ = 246.1 Hz), 144.3, 140.1, 138.2, 135.2, 137.2 (d, $^4J_{C–F}$ = 3.2 Hz), 129.4, 128.6 (d, $^3J_{C–F}$ = 8.1 Hz), 128.5, 127.1, 115.6 (d, $^2J_{C–F}$ = 21.4 Hz), 83.8, 41.8, 24.9. **^{19}F NMR {^{1}H}** (376 MHz, $CDCl_3$) δ −115.90 (s). **IR** ν (cm^{-1}): 3027, 2978, 2950, 2853, 1600, 1494, 1485. **HRMS** (EI) *m/z*: Calculated for $C_{25}H_{26}BFO$ 388.2010. Found 388.1999 $[M]^+$. **mp**:100–105 °C.

(4'-Bromo-[1,1'-biphenyl]-4-yl)(4-fluorophenyl)methanol (S12a)

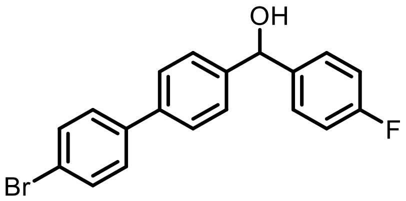

To a solution of 4,4'-dibromo-1,1'-biphenyl (9.36 g, 30.0 mmol, 1.00 eq) in THF (80 mL) at −78 °C was added *n*-BuLi (25.2 mL of 1.31 M in hexanes, 33.0 mmol, 1.10 eq) dropwise. The reaction was stirred for 30 min at −78 °C, after which time, 4-fluorobenzaldehyde (4.09 g, 33.0 mmol, 1.10 eq) was added dropwise. The reaction was allowed to warm to room temperature and stirred for 4 h, after which time the reaction was quenched with the careful addition of H_2O (30 mL) and the resulting mixture was stirred for a further 1 h at room temperature. The aqueous phase was extracted with ethyl acetate (3 × 50 mL) and the combined organic phases were washed with brine (3 × 30 mL), dried over $MgSO_4$, filtered, and concentrated under reduced pressure. The resulting residue was purified by flash column chromatography on silica gel (hexane:EtOAc, gradient 100:0–50:50) to afford the title compound (5.8 g, 54%) as a viscous oil.

***R*$_f$** (hexane:EtOAc, 90:10): 0.10. **^{1}H NMR** (400 MHz, $CDCl_3$) δ 7.62 – 7.47 (m, 4H), 7.47 – 7.32 (m, 6H), 7.09 – 6.98 (m, 2H), 5.88 (d, *J* = 3.4 Hz, 1H), 2.26 (d, *J* = 3.4 Hz, 1H). **^{13}C NMR** (126 MHz, $CDCl_3$) δ 162.3 (d, $^1J_{C\text{–}F}$ = 246.0 Hz), 143.1, 139.6, 139.5, 139.4, 139.4, 131.9, 128.3 (d, $^3J_{C\text{–}F}$ = 8.1 Hz), 127.1, 127.0, 121.7, 115.4 (d, $^2J_{C\text{–}F}$ = 21.5 Hz), 75.4. **^{19}F NMR** (377 MHz, $CDCl_3$) δ −114.71 – −114.82 (m). **IR** ν (cm^{-1}): 3325, 3030, 2885, 1898, 1602, 1506, 1483. **HRMS** (APCI) *m/z*: Calculated for $C_{19}H_{14}BrFO$ 356.0212. Found 339.0194 $[M+H-H_2O]^+$.

4-Bromo-4'-(4-fluorobenzyl)-1,1'-biphenyl (S12b)

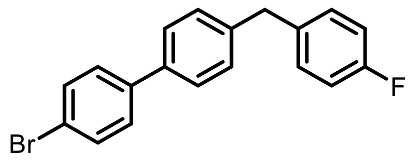

Sodium borohydride (1.89 g, 50.0 mmol, 3.00 eq) was added portionwise to trifluoroacetic acid (25 mL) at 0 °C, followed by the dropwise addition of a solution of **(4'-bromo-[1,1'-biphenyl]-4-yl)(4-fluorophenyl)methanol (S12a)** (5.80 g, 16.3 mmol, 1.00 eq) in DCM (25 mL). The reaction was allowed to warm to room temperature and was stirred for a further 4 h. After this time, the reaction mixture was subject to the slow addition of aqueous NaOH solution (2 M) until pH 7 was reached. The aqueous phase was then extracted with DCM (3 × 30 mL) and the combined organic phases were washed with brine (2 × 20 mL), dried over $MgSO_4$, filtered, and concentrated under reduced pressure. The resulting orange oil was purified via flash column chromatography on silica gel (hexane, isocratic) to afford the title compound (4.75 g, 85%) as a white solid.

***R*f** (hexane): 0.40. **^{1}H NMR** (500 MHz, $CDCl_3$) δ 7.57 – 7.51 (m, 2H), 7.49 – 7.45 (m, 2H), 7.46 – 7.39 (m, 2H), 7.24 (d, J = 6.5 Hz, 2H), 7.23 – 7.13 (m, 2H), 7.03 – 6.95 (m, 2H), 3.99 (s, 2H). **^{13}C NMR** (126 MHz, $CDCl_3$) δ 161.7 (d, $^{1}J_{C\text{–}F}$ = 244.2 Hz), 140.7, 140.0, 138.2, 136.7 (d, $^{4}J_{C\text{–}F}$ = 3.2 Hz), 132.1, 130.5 (d, $^{3}J_{C\text{–}F}$ = 7.8 Hz), 129.6, 128.7, 121.6, 127.3, 115.5 (d, $^{2}J_{C\text{–}F}$ = 21.2 Hz), 40.9. **^{19}F NMR** (376 MHz, $CDCl_3$) δ −76.49 (s, TFA), −116.90 – −117.30 (m). **IR** ν (cm^{-1}): 3363, 2903, 2891, 1900, 1679, 1607, 1509. **HRMS** (EI) *m/z*: Calculated for $C_{19}H_{14}BrF$ 340.0263. Found 340.0254 $[M]^+$. **mp**: 107–119 °C.

2-(4'-(4-Fluorobenzyl)-[1,1'-biphenyl]-4-yl)-4,4,5,5-tetramethyl-1,3,2-dioxaborolane (12)

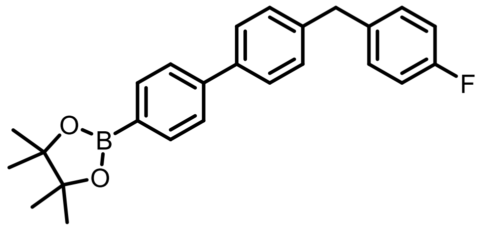

A solution of **4-bromo-4'-(4-fluorobenzyl)-1,1'-biphenyl (S12b)** (3.00 g, 8.80 mmol, 1.00 eq), bis(pinacolato)-diboron (2.65 g, 10.5 mmol, 1.20 eq), potassium acetate (3.80 g, 17.6 mmol, 2.00 eq) and Pd(dppf)Cl_2•CH_2Cl_2 (0.359 g, 5.00 mol%) in 1,4-dioxane (80 mL) was heated to reflux and stirred at this temperature for 16 h. After this time the reaction was allowed to cool to room temperature and treated with water (20 mL). The aqueous phase was extracted with DCM (3 × 30 mL) and combined organic phases were washed with brine (3 × 30 mL), dried over $MgSO_4$, filtered, and concentrated under reduced pressure. The resulting residue was purified by flash column chromatography on silica gel (hexane, isocratic) to afford the title compound (1.91 g, 64%) as a white solid.

$\boldsymbol{R}_f$ (hexane): 0.10. **^{1}H NMR** (400 MHz, $CDCl_3$) δ 7.91 – 7.84 (m, 2H), 7.63 – 7.50 (m, 4H), 7.26 – 7.22 (m, 2H), 7.20 – 7.15 (m, 2H), 7.04 – 6.93 (m, 2H), 4.00 (s, 2H), 1.37 (s, 12H). **^{13}C NMR** (101 MHz, $CDCl_3$) *Note: ^{13}C NMR signals for the aromatic carbon atom adjacent to the boron atom are not observed due to rapid quadrupolar relaxation.* δ 161.7 (d, $^1J_{C\text{–}F}$ = 244.2 Hz), 143.8, 140.6, 139.2, 136.8 (d, $^4J_{C\text{–}F}$ = 3.3 Hz), 135.5, 130.6 (d, $^3J_{C\text{–}F}$ = 7.9 Hz), 129.5, 127.6, 126.5, 115.5 (d, $^2J_{C\text{–}F}$ = 21.2 Hz), 84.0, 41.0, 25.1. **^{19}F NMR {^{1}H}** (377 MHz, $CDCl_3$) δ −117.25. **IR** ν (cm^{-1}): 3077, 3030, 2978, 2904, 1901, 1681, 1607. **HRMS** (EI) *m/z*: Calculated for $C_{25}H_{26}BFO_2$ 388.2010. Found 388.2000 $[M]^+$. **mp**: 142–150 °C.

Chloro(4-fluorophenyl)dimethylsilane (S13a)

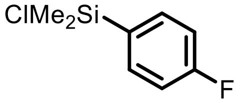

A solution of 1-bromo-4-fluorobenzene (3.5 g, 20 mmol, 1.0 eq) in THF (12 mL) was added dropwise to magnesium turnings (0.53 g, 22 mmol, 1.1 eql) in THF (8 mL), activated with a crystal of iodine. The mixture was heated to reflux for 1 hour before being cooled to room temperature. This solution was then added dropwise to a stirred solution of dichlorodimethylsilane (3.1 g, 24 mmol, 1.2 eq) in Et_2O (20 mL) at room temperature. The reaction mixture was stirred at room temperature for 18 hours before the solvent was removed under reduced pressure. Hexane (100 mL) was added and the resulting slurry filtered through Celite®, and washed with hexane (3 × 20 mL). The solvent was removed under reduced pressure and the residue purified by vacuum distillation to afford the title compound (1.6 g, 43%, 60 °C/1 mbar) as a pale pink oil.

^{1}H NMR ($CDCl_3$, 400 MHz) δ 7.61 (m, 2H), 7.11 (m, 2H), 0.68 (s, 6H). **^{19}F {^{1}H} NMR** ($CDCl_3$, 376 MHz) δ −109.7 (s). Data in accordance with the literature.[2]

(4-Bromophenyl)(4-fluorophenyl)dimethylsilane (S13b)

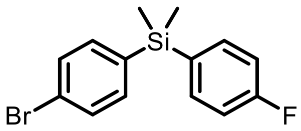

To a stirred solution of 1,4-dibromobenzene (1.4 g, 6.0 mmol, 1.0 eq) in THF (30 mL) cooled to −78 °C was added *n*-BuLi (4.5 mL of 1.32 M in hexanes, 6.0 mmol, 1.0 eq). The reaction mixture was stirred at −78 °C for 1 h before a solution of **chloro(4-fluorophenyl)dimethylsilane (S13a)** (1.2 g, 6.0 mmol, 1.0 eq.) in THF (6 mL) was added dropwise. The reaction mixture was allowed to warm to room temperature and stirred overnight before being cooled to 0 °C and quenched with 1M HCl (15 mL). The phases were separated and the aqueous phase extracted with ether (2 × 20 mL). The combined organic phases were washed with brine, dried over $MgSO_4$, filtered and concentrated under reduced pressure. The residue was purified by flash column chromatography on silica gel (hexane, isocratic) to give the title compound (1.1 g, 61%) as a colourless oil.

***R*f** (hexane): 0.50. **^{1}H NMR** ($CDCl_3$, 400 MHz): δ 7.50–7.44 (m, 4H), 7.37–7.34 (m, 2H), 7.08–7.03 (m, 2 H), 0.54 (s, 6H). **^{13}C NMR** (101 MHz, $CDCl_3$) δ 164.1 (d, $^1J_{C\text{–}F}$ = 248.7 Hz), 137.0, 136.2 (d, $^3J_{C\text{–}F}$ = 7.4 Hz), 135.9, 133.2 (d, $^4J_{C\text{–}F}$ = 3.8 Hz), 131.2, 124.3, 115.3 (d, $^2J_{C\text{–}F}$ = 19.6 Hz), –2.2. **^{19}F NMR** ($CDCl_3$, 376 MHz): δ −111.5 (tt, *J* = 6.3, 6.1 Hz). **IR ν** (cm^{-1}): 3032, 2957, 1901, 1585, 1498, 1479, 1407, 1386, 1376. **HRMS** (EI) *m/z*: Calculated for $C_{14}H_{14}FBrSi$ 308.0027. Found 308.0025 $[M]^+$.

(4-Fluorophenyl)dimethyl(4-(4,4,5,5-tetramethyl-1,3,2-dioxaborolan-2-yl)phenyl)silane (13)

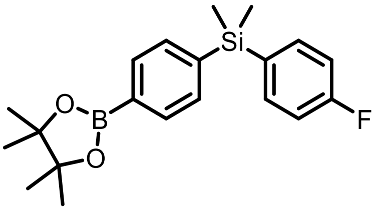

To a stirred solution of **(4-Bromophenyl)(4-fluorophenyl)dimethylsilane (S13b)** (1.0 g, 3.2 mmol, 1.0 eq.) in THF (30 mL) cooled to −78 °C was added *n*-BuLi (2.6 mL of 1.32 M in hexane, 3.5 mmol, 1.1 eq.). The reaction mixture was stirred at −78 °C for 1 h followed by the addition of 2-isopropoxy-4,4,5,5-tetramethyl-1,3,2-dioxaborolane (0.8 mL, 3.9 mmol, 1.2 eq.) dropwise. The reaction mixture was allowed to warm to room temperature and stirred overnight before being cooled to 0 °C and quenched with saturated aqueous KH_2PO_4 (15 mL) and stirred for a further 1 hour. The phases were separated and the aqueous phase extracted with ethyl acetate (2 × 20 mL). The combined organic phases were washed with brine, dried over $MgSO_4$, filtered and concentrated under reduced pressure. The resulting residue was purified by flash column chromatography on silica gel (hexane:EtOAc, 95:5) to give the title compound (0.9 g, 77%) as a white solid.

***R*f** (hexane): 0.50. **^{1}H NMR** ($CDCl_3$, 400 MHz) δ 7.80–7.78 (m, 2 H), 7.53–7.51 (m, 2 H), 7.49–7.44 (m, 2 H), 7.06–7.01 (m, 2 H), 1.34 (s, 12 H), 0.54 (s, 6 H). **^{13}C NMR** (101 MHz, $CDCl_3$) *Note: ^{13}C NMR signals for the aromatic carbon atom adjacent to the boron atom are not observed due to rapid quadrupolar relaxation*. δ 164.0 (d, $^1J_{C\text{–}F}$ = 248.2 Hz), 141.7, 136.3 (d, $^3J_{C\text{–}F}$ = 7.4 Hz), 134.1, 133.7, 133.6, 115.2 (d, $^2J_{C\text{–}F}$ = 19.6 Hz), 84.0, 25.0, -2.2. **^{19}F NMR** ($CDCl_3$, 376 MHz) δ −111.9 (tt, *J* = 9.5, 6.2 Hz). **IR** ν (cm^{-1}): 2980, 1602, 1587, 1500, 1387, 1358, 1328, 1307. **HRMS** (EI) *m/z*: Calculated for $C_{20}H_{26}BFO_2Si$ 356.1779. Found 341.1538 $[M - Me]^+$. **mp:** 109–111 °C.

4-Chloro-*N*-(4-fluorophenyl)-*N*-methylaniline (S14a)

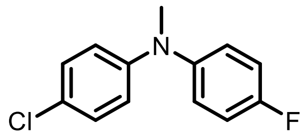

To a stirred mixture of $Pd(OAc)_2$ (0.11 g, .05 mol%), *rac*-BINAP (0.47 g, 7.5 mol%) and Cs_2CO_3 (4.9 g, 15 mmol. 1.5 eq) in anhydrous toluene (50 mL) was added 1-bromo-4-fluorobenzene (1.1 mL, 10 mmol, 1.0 eq) and 4-chloro-*N*-methylaniline (1.2 mL, 10 mmol, 1.0 eq). The reaction mixture was heated to 85 °C for 20 h before being cooled to room temperature and filtered through Celite®, washing with ethyl acetate. The filtrate was concentrated under reduced pressure and the residue was purified by flash column chromatography on silica gel (hexane:EtOAc, 90:10) to give the title compound **(**2.1 g, 90%) as an orange oil.

$\boldsymbol{R}_f$ (hexane:EtOAc, 90:10): 0.60. **^{1}H NMR** ($CDCl_3$, 400 MHz) δ 7.19–7.15 (m, 2 H), 7.07–6.99 (m, 4 H, H-6), 6.79–6.76 (m, 2 H), 3.25 (s, 3 H). **^{13}C NMR** (101 MHz, $CDCl_3$) δ 159.3 (d, $^1J_{C\text{–}F}$ = 242.9 Hz), 148.1, 145.0 (d, $^4J_{C\text{–}F}$ = 2.8 Hz), 129.2, 125.2 (d, $^3J_{C\text{–}F}$ = 7.9 Hz), 124.7, 118.7, 116.4 (d, $^2J_{C\text{–}F}$ = 22.4 Hz), 40.8. **^{19}F NMR** ($CDCl_3$, 376 MHz) δ −119.5 (tt, J = 7.9, 5.1 Hz). **IR** ν (cm^{-1}): 2880, 2813, 1878, 1592, 1598, 1505, 1489, 1336, 1308. **HRMS** (ESI) *m/z*: Calculated for $C_{13}H_{11}NFCl$ 235.0564 Found 236.0648 $[M+H]^+$.

4-Fluoro-*N*-methyl-*N*-(4-(4,4,5,5-tetramethyl-1,3,2-dioxaborolan-2-yl)phenyl)aniline (14)

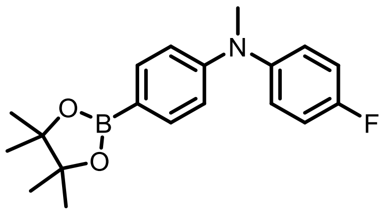

A solution of $Pd(dba)_2$ (0.19 g, 6.0 mol%) and PCy_3 (0.23 mg, 15 mol%) was stirred in 1,4-dioxane (30 mL) for 30 minutes before KOAc (0.75 g, 7.5 mmol, 1.5 eq), bis(pinacolato)-diboron (1.4 g, 5.5 mmol, 1.1 eq) and **4-chloro-*N*-(4-fluorophenyl)-*N*-methylaniline (S14a)** (1.2 g, 5.0 mmol, 1.0 eq) were added. The reaction mixture was heated to 80 °C for 20 h. After this time, the reaction mixture was allowed to cool to room temperature before being poured into water (20 mL) and the aqueous phase extracted with ethyl acetate (3 × 25 mL). The combined organic phases were washed with brine, dried over $MgSO_4$, filtered, and concentrated under reduced pressure. The resulting residue was purified by flash column chromatography on silica gel (hexane:EtOAc, 90:10) to give the title compound (1.1 g, 67%) as white solid.

***R*f** (hexane:EtOAc, 90:10): 0.21. **^{1}H NMR** ($CDCl_3$, 400 MHz) δ 7.68–7.64 (m, 2H), 7.15–7.12 (m, 2H), 7.06–7.02 (m, 2H), 6.79–6.75 (m, 2H), 3.29 (s, 3H), 1.33 (s, 12H). **^{13}C NMR** (101 MHz, $CDCl_3$) *Note: ^{13}C NMR signals for the aromatic carbon atom adjacent to the boron atom are not observed due to rapid quadrupolar relaxation*. δ 159.9 (d, $^1J_{C–F}$ = 243.8 Hz), 144.6 (d, $^4J_{C–F}$ = 3.1 Hz), 136.2, 128.6 (d, J = 19.4 Hz), 127.0 (d, $^3J_{C–F}$ = 8.2 Hz), 116.5 (d, $^2J_{C–F}$ = 22.4 Hz), 114.9, 83.5, 40.5, 25.0. **^{19}F NMR** ($CDCl_3$, 376 MHz) δ −118.1 (tt, J = 8.1, 4.9 Hz). **IR** ν (cm^{-1}): 2978, 2928, 2874, 2815, 1896, 1606, 1598, 1552, 1505, 1465, 1417, 1396, 1370, 1358, 1345, 1312, 1270, 1237. **HRMS** (ESI) *m/z*: Calculated for $C_{19}H_{23}NO_2BF$ 327.1806. Found 328.1878 $[M+H]^+$. **mp**: 116–118 °C.

1-Bromo-4-(4-fluorophenoxy)benzene (S15a)

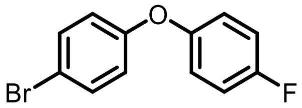

A mixture of 4-fluorophenol (2.5 g, 22 mmol, 1.1 eq), 1-bromo-4-iodobenzene (5.7 g, 20 mmol, 1.0 eq), Cs_2CO_3 (13 g, 40 mmol, 20 eq), CuI (0.075 g, 2.0 mol%) and *N*,*N*-dimethylglycine (0.16 g, 1.5 mmol, 7.5 mol%) in 1,4-dioxane (100 mL, 0.2 M) was heated to reflux for 24 h. The mixture was then allowed to cool to room temperature before being poured into a mixture of water (30 mL) and ethyl acetate (30 mL). The phases were separated and the aqueous phase was extracted with ethyl acetate (2 × 30 mL). The combined organic layers were washed with brine, dried over $MgSO_4$, filtered and concentrated under reduced pressure. The resulting residue was purified by flash column chromatography on silica gel (hexane:EtOAc, 99:1) to provide the title compound (1.6 g, 30%) as a brown oil. Note that the sample contains 1-iodo-4-(4-fluorophenoxy)benzene as an impurity (approx. 8%) and the mixture was carried through to the subsequent borylation reaction without further purification.

^{1}H NMR ($CDCl_3$, 400 MHz) δ 7.42 (dt, 2 H, J = 10.0, 2.2 Hz), 7.07–7.01 (m, 2H), 7.00–6.95 (m, 2 H), 6.84 (dt, 2 H, J = 10.1, 2.2 Hz). **^{13}C NMR** (101 MHz, $CDCl_3$) δ 159.2 (d, $^1J_{C\text{–}F}$ = 242.5 Hz), 157.1, 152.6 (d, $^4J_{C\text{–}F}$ = 2.6 Hz), 132.9, 120.9 (d, $^3J_{C\text{–}F}$ = 8.3 Hz), 120.0, 116.7 (d, $^2J_{C\text{–}F}$ = 23.5 Hz), 115.7. **^{19}F NMR** ($CDCl_3$, 376 MHz) δ −119.3 (tt, J = 7.9, 4.4 Hz). Data in accordance with the literature.[3]

2-(4-(4-Fluorophenoxy)phenyl)-4,4,5,5-tetramethyl-1,3,2-dioxaborolane (15)

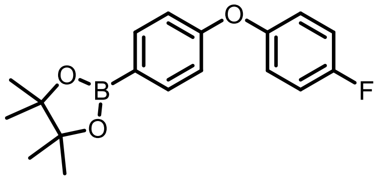

To a stirred solution of **1-bromo-4-(4-fluorophenoxy)benzene (S15a)** (1.8 g, 6.5 mmol, 1.0 eq) in THF (65 mL) cooled to −78 °C was added *n*-BuLi (5.9 mL of 1.2 M in hexanes, 7.2 mmol, 1.1 eq). The reaction mixture was stirred at −78 °C for 1 h followed by the addition of 2-isopropoxy-4,4,5,5-tetramethyl-1,3,2-dioxaborolane (1.6 mL, 7.8 mmol, 1.2 eq) dropwise. The reaction mixture was allowed to warm to room temperature and stirred overnight before being cooled to 0 °C and quenched with saturated aqueous KH_2PO_4 (20 mL) and stirred for a further 1 h. The phases were separated and the aqueous phase extracted with ethyl acetate (2 × 20 mL). The combined organic phases were washed with brine, dried over $MgSO_4$, filtered and concentrated under reduced pressure. The residue was purified by flash column chromatography (hexane:EtOAc, 90:10) to give the title compound (1.3 g, 64%) as a white solid.

^{1}H NMR ($CDCl_3$, 400 MHz) δ 7.79–7.76 (m, 2 H), 7.06–6.97 (m, 4 H), 6.95–6.92 (m, 2 H), 1.34 (s, 12 H). **^{13}C NMR** (101 MHz, $CDCl_3$) *Note: ^{13}C NMR signals for the aromatic carbon atom adjacent to the boron atom are not observed due to rapid quadrupolar relaxation.* δ 160.7, 159.3 (d, $^{1}J_{C-F}$ = 242.2 Hz), 152.4 (d, $^{4}J_{C-F}$ = 2.7 Hz), 136.9, 121.3 (d, $^{3}J_{C-F}$ = 8.3 Hz), 117.3, 116.6 (d, $^{2}J_{C-F}$ = 23.4 Hz), 83.9, 25.0. **^{19}F NMR** ($CDCl_3$, 376 MHz) δ −119.5 (tt, *J* = 7.8, 4.7 Hz). **mp:** 74–76 °C. Data in accordance with the literature.[4]

2.6) Synthesis of Polyaniline Tracks 16 and 17

The experimental procedures in the following section (Section 2.6) have been adapted from the literature.[5,6]

N-(4-Bromo-phenyl)-*N'*-phenyl-benzene-1,4-diamine (S16a)

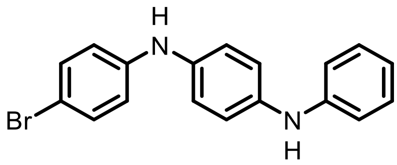

A solution of *N*-phenyl-*p*-phenylenediamine (3.68 g, 20.0 mmol, 1.00 eq), *p*-dibromobenzene (4.72 g, 20.0 mmol, 1.00 eq), sodium *tert*-butoxide (4.80 g, 50.0 mmol, 2.50 eq), *rac*-BINAP (1.12 g, 9.00 mol%), and $Pd_2(dba)_3$ (0.549 g, 3.00 mol%) in THF (100 mL) was heated to reflux and stirred for 16 h. After this time, the reaction mixture was cooled to room temperature and concentrated under reduced pressure. The resulting residue was purified by flash column chromatography on silica gel (DCM, isocratic) to afford the title compound (5.63 g, 83%) as an orange powder.

$\boldsymbol{R_f}$ (DCM): 0.50. **^{1}H NMR** (400 MHz, $(CD_3)_2CO$) δ 7.32 – 7.27 (m, 2H), 7.24 – 7.17 (m, 2H), 7.10 (d, J = 2.3 Hz, 4H), 7.06 – 7.01 (m, 2H), 6.97 – 6.91 (m, 2H), 6.82 – 6.73 (m, 1H). **^{13}C NMR** (101 MHz, $(CD_3)_2CO$) 145.9, 145.8, 139.1, 136.9, 132.7, 130.0, 122.1, 120.7, 120.1, 117.7, 116.8, 110.3. **mp:** 111–115 °C. Data in accordance with the literature.[5]

***N*-(4-Bromo-phenyl)-*N*,*N'*-dimethyl-*N'*-phenyl-benzene-1,4-diamine (S16b)**

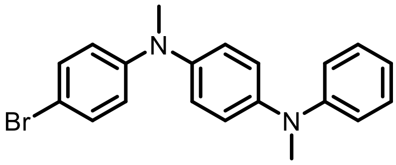

To a solution of ***N*-(4-bromo-phenyl)-*N'*-phenyl-benzene-1,4-diamine (S16a)** (5.63 g, 16.6 mmol, 1.00 eq) in THF (100 mL) at −78°C was added methyllithium (36.3 mL of a 1.6 M solution in diethyl ether, 58.1 mmol, 3.50 eq) dropwise and the reaction mixture was stirred at this temperature for 1 h. After this time, the reaction was warmed to −60°C and was treated with the dropwise addition methyl iodide (15.7 mL, 250 mmol, 15.0 eq). The reaction mixture was then allowed to warm to room temperature and stirred for a further 16 h. After this time the reaction mixture was treated with aqueous KOH (2 M, 50 mL) and stirred for a further 2 h at room temperature. The aqueous phase was extracted with DCM (3 × 30 mL) and the combined organic phases were washed with brine (3 × 20 mL), dried over $MgSO_4$, filtered, and concentrated under reduced pressure. The resulting residue was triturated with hexane to provide a solid which was isolated by Buchner filtration (washing with cold hexane) to afford the title compound (5.57 g, 91%) as a white solid.

^{1}H NMR (400 MHz, $(CD_3)_2CO$) δ 7.34 – 7.22 (m, 4H), 7.11 – 6.98 (m, 6H), 6.96 – 6.87 (m, 1H,), 6.82 – 6.73 (m, 2H), 3.30 (s, 3H), 3.26 (s, 3H). **^{13}C NMR** (101 MHz, $(CD_3)_2CO$) δ 150.1, 149.7, 146.4, 143.3, 132.4, 130.0, 126.1, 123.0, 121.6, 120.4, 118.8, 110.9, 40.7, 40.6. **mp:** 83–87 °C. Data in accordance with the literature.[5]

N^1,N^4-dimethyl-N^1-phenyl-N^4-(4-(4,4,5,5-tetramethyl-1,3,2-dioxaborolan-2-yl)phenyl)benzene-1,4-diamine (16)

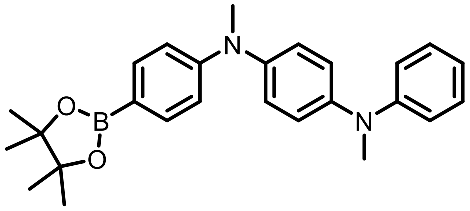

To a solution of ***N*-(4-bromo-phenyl)-*N,N'*-dimethyl-*N'*-phenyl-benzene-1,4-diamine (S16b)** (1.50 g, 4.08 mmol, 1.00 eq) in THF (40 mL) at −78 °C was added *n*-BuLi (3.45 mL of 1.30 M in hexanes, 4.49 mmol, 1.10 eq) dropwise and the reaction was stirred at −78 °C for 1 h. After this time, 2-isopropoxy-4,4,5,5-tetramethyl-1,3,2- dioxaborolane (1.00 mL, 4.89 mmol, 1.20 eq) was added dropwise and the reaction was allowed to warm to room temperature and stirred at this temperature for 3 h. After this time, the reaction was quenched by the careful addition of saturated aqueous KH_2PO_4 (30 mL). The aqueous phase was extracted with ethyl acetate (3 × 20 mL) and the combined organic phases were washed with brine (2 × 20 mL), dried over $MgSO_4$, filtered, and concentrated under reduced pressure. The resulting residue was purified by flash column chromatography on silica gel (hexane:EtOAc, 95:5) to afford the title compound (0.852 g, 50%) as a white solid.

***R*f** (hexane:EtOAc, 95:5): 0.15. **^{1}H NMR** (400 MHz, C_6D_6) δ 8.12 (d, *J* = 8.6 Hz, 2H), 7.06 (d, *J* = 8.8 Hz, 2H), 6.94 – 6.73 (m, 9H), 2.84 (s, 3H), 2.82 (s, 3H), 1.10 (s, 12H). **^{13}C NMR** (101 MHz, C_6D_6) *Note: ^{13}C NMR signals for the aromatic carbon atom adjacent to the boron atom are not observed due to rapid quadrupolar relaxation* δ 152.3, 149.6, 145.9, 142.8, 136.8, 129.5, 126.3, 122.5, 121.2, 120.1, 115.3, 83.3, 40.2, 40.0, 25.0. **IR ν (cm^{-1}):** 2925, 2853,1724, 1563, 1462. **HRMS** (ESI) *m/z*: Calculated for $C_{26}H_{31}BN_2O_2$ 414.2479. Found 415.2543 [M + H]$^+$. **mp:** 131–132 °C.

***N*[1],*N*[4]-bis(4-bromophenyl)benzene-1,4-diamine (S17a)**

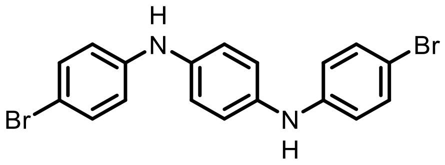

A solution of *p*-phenylenediamine (2.00 g, 18.5 mmol, 1.00 eq), 1,4-dibromobenzene (9.16 g, 38.9 mmol, 2.10 eq), sodium *tert*-butoxide (4.62 g, 48.0 mmol, 2.60 eq), $Pd_2(dba)_3$ (0.507 g, 3.00 mol%) and *rac*-BINAP (1.04 g, 9.00 mol %) in THF (100 mL) was heated to reflux and stirred at this temperature for 3 days. After this time, the reaction mixture was cooled to room temperature and the reaction mixture was diluted with ethyl acetate and filtered through Celite®, washing with ethyl acetate. The filtrate was concentrated under reduced pressure to afford the title compound (6.84 g, 89%) as a green solid. Note that the sample contains 1,4-dibromobenzene as an impurity (approx. 5%) and the mixture was carried through to the subsequent reaction without further purification.

^{1}H NMR (400 MHz, $(CD_3)_2CO$) δ 7.48 (s), 7.28 (d, J = 9.0 Hz, 4H), 7.08 (s, 4H), 6.94 (d, J = 9.0 Hz, 4H). **^{13}C NMR** (101 MHz, $(CD_3)_2CO$) δ 144.7, 137.0, 131.9, 120.8, 117.2, 109.8. **mp:** 143–149 °C. Data in accordance with the literature.[5]

N^1,N^4-bis(4-bromophenyl)-N^1,N^4-dimethylbenzene-1,4-diamine (S17b)

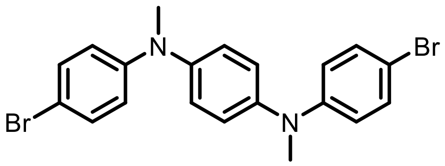

A solution of **N^1,N^4-bis(4-bromophenyl)benzene-1,4-diamine (S17a)** (3.00 g, 7.21 mmol, 1.00 eq) in THF (50 mL) at −78 °C was treated with the dropwise addition of methyllithium (15.7 mL of a 1.6 M solution in diethyl ether, 25.2 mmol, 3.50 eq) and the mixture was stirred at this temperature for a further 1 h. The reaction mixture was then warmed to −60°C and treated with the dropwise addition of methyl iodide (6.70 mL, 108 mmol, 15.0 eq). The reaction mixture was then allowed to warm to room temperature and stirred for a further 16 h. After this time the reaction mixture was treated with aqueous KOH (2 M, 50 mL) and stirred for a further 2 h at room temperature. The aqueous phase was extracted with DCM (3 × 30 mL) and the combined organic phases were washed with brine (3 × 20 mL), dried over $MgSO_4$, filtered, and concentrated under reduced pressure to afford the title compound (3.12 g, 97%) as a brown solid.

^{1}H NMR (400 MHz, $(CD_3)_2CO$) δ 7.35 (d, J = 9.2 Hz, 4H), 7.12 (s, 4H), 6.86 (d, J = 9.2 Hz, 4H), 3.30 (s, 6H). **^{13}C NMR** (101 MHz, $(CD_3)_2CO$) δ 149.5, 145.0, 132.6, 125.2, 120.0, 111.8, 40.6. **mp:** 155–63 °C. Data in accordance with the literature.[5]

N^1-(4-bromophenyl)-N^1,N^4-dimethyl-N^4-(4-(methyl(4-(methyl(phenyl)amino)phenyl)amino)phenyl) benzene-1,4-diamine (S17d)

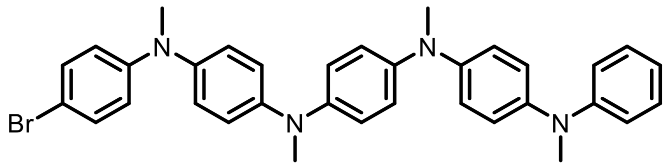

A solution of **N^1,N^4-bis(4-bromophenyl)-N^1,N^4-dimethylbenzene-1,4-diamine (S17b)** (5.00 g, 11.2 mmol, 1.00 eq), *N*-phenyl-*p*-phenylenediamine (2.27 g, 12.3 mmol, 1.10 eq), sodium *tert*-butoxide (2.15 g, 22.4 mmol, 2.00 eq), $Pd_2(dba)_3$ (0.307 g, 3.00 mol%), and *rac*-BINAP (0.670 g, 9.00 mol%) in THF (100 mL) was heated to reflux and stirred for 16 h. After this time, the reaction mixture was cooled to room temperature, diluted with DCM (50 mL) and filtered over Celite®, washing with DCM. The filtrate was concentrated under reduced pressure. The resulting black residue was purified by flash column chromatography on silica gel (DCM:hexane, 75:25) to afford **N^1-(4-bromophenyl)-N^1,N^4-dimethyl-N^4-(4-((4-(phenylamino)phenyl)amino)phenyl)benzene-1,4-diamine (S17d)** as a pale pink solid (1.30 g, 2.38 mmol), which identified by ^{1}H NMR spectroscopy.

N^1-(4-bromophenyl)-N^1,N^4-dimethyl-N^4-(4-((4-(phenylamino)phenyl)amino)phenyl)benzene-1,4-diamine (S17c) was used immediately in the subsequent without further characterisation. A solution of **N^1-(4-bromophenyl)-N^1,N^4-dimethyl-N^4-(4-((4-(phenylamino)phenyl)amino)phenyl)benzene-1,4-diamine (S17c)** (1.30 g, 2.38 mmol, 1.0 eq) in THF (20 mL) at −78 °C was treated with the dropwise addition of methyllithium (5.20 mL of a 1.6 M solution in diethyl ether, 8.28 mmol, 3.50 eq) and the mixture was stirred at this temperature for a further 1 h. The reaction mixture was then warmed to −60°C and treated with the dropwise addition of methyl iodide (2.20 mL, 34.5 mmol, 15.0 eq). The reaction mixture was then allowed to warm to room temperature and stirred for a further 16 h. After this time the reaction mixture was treated with aqueous KOH (2 M, 50 mL) and stirred for a further 2 h at room temperature. The aqueous phase was extracted with DCM (3 × 30 mL) and the combined organic phases were washed with brine (3 × 20 mL), dried over $MgSO_4$, filtered, and concentrated under reduced pressure. The resulting residue was triturated with hexane to provide a solid which was isolated by Buchner filtration (washing with cold hexane) to afford the title compound (1.22 g, 18% from **S17b**) as a pale pink solid.

^{1}H NMR (600 MHz, C_6D_6) δ 7.24 (d, J = 9.0 Hz, 2H), 7.19 – 7.13 (m, 2H), 7.01 – 6.89 (m, 10H), 6.89 – 6.77 (m, 5H), 6.51 (d, J = 9.0 Hz, 2H), 2.98 (s, 6H), 2.97 (s, 3H), 2.81 (s, 3H). **^{13}C NMR** (151 MHz, C_6D_6) δ 150.0, 149.1, 146.8, 145.2, 145.1, 143.5, 143.1, 141.1, 132.0, 129.4, 129.4, 126.1, 124.4, 124.1, 121.9, 121.5, 120.0, 119.3, 118.2, 117.7, 110.8, 40.5, 40.3, 40.3. **IR** ν (cm^{-1}): 3032, 2945, 2887, 2819, 1726, 1593, 1512. **HRMS** (ESI) *m/z*: Calculated for $C_{34}H_{33}BrN_4$ 576.1889. Found 576.1857 $[M]^+$. **mp**: 178–180 °C.

N^1,N^4-dimethyl-N^1-(4-(methyl(4-(4,4,5,5-tetramethyl-1,3,2-dioxaborolan-2-yl)phenyl)amino)phenyl)-N^4-(4-(methyl(phenyl)amino)phenyl)benzene-1,4-diamine (17)

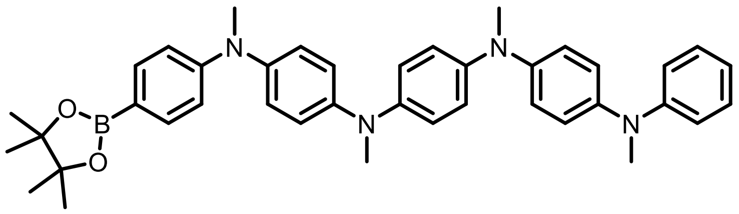

A solution of **N^1-(4-bromophenyl)-N^1,N^4-dimethyl-N^4-(4-(methyl(4-(methyl(phenyl)amino)phenyl)amino)phenyl) benzene-1,4-diamine (S17d)** (0.689 g, 1.25 mmol, 1.00 eq), bis(pinacolato)-diboron (0.350 g, 1.38 mmol, 1.10 eq), Cs_2CO_3 (0.886 g, 2.51 mmol, 2.00 eq), $Pd(OAc)_2$ (0.0141 g, 5.00 mol%) and XPhos (0.0596 g, 10.0 mol%) in anhydrous toluene (40 mL) was heated to reflux and stirred at this temperature for 16 h. After this time the reaction was allowed to cool to room temperature and filtered though Celite®, washing with ethyl acetate (30 mL). The solvent was removed under reduced pressure and the resulting residue was triturated with hexane to provide a solid which was isolated by Buchner filtration (washing with cold hexane) to afford the title compound (0.703 g, 90%) as a pale yellow solid.

^{1}H NMR (400 MHz, C_6D_6) δ 8.20 (d, *J* = 8.8 Hz, 2H), 7.19 – 7.16 (m, 2H), 6.97 – 6.88 (m, 14H), 6.86 – 6.77 (m, 3H), 2.97 (s, 3H), 2.97 (s, 3H), 2.95 (s, 3H), 2.90 (s, 3H), 1.16 (s, 12H). **^{13}C NMR** (101 MHz, C_6D_6) δ 152.5, 150.1, 146.9, 145.3, 145.0, 143.3, 143.2, 141.0, 136.8, 129.4, 126.9, 124.6, 124.0, 121.8, 121.6, 119.8, 119.3, 117.9, 114.6, 40.5, 40.5, 40.3, 40.1, 25.1. **IR** ν (cm^{-1}): 3675, 2985, 2942, 2901, 2087, 1770, 1736, 1447. **HRMS** (MALDI) *m/z*: Calculated for $C_{40}H_{45}O_2N_4B$ 624.3637. Found 624.3631 $[M]^+$. **mp:** decomposition at 177 °C.

3) Rhodium-Catalysed Norbornane Incorporation for Controlled Translational Motion Along Polyaromatic Tracks

3.1) General Procedures

General Procedure A:

Under a nitrogen atmosphere an over-dried Schlenk tube (15 mm diameter) was charged with the polyaromatic track (0.20 mmol, 1.0 eq), CsF (61 mg, 0.40 mmol, 2.0 eq), dppp (5.0 mg, 5.0 mol%), $[RhCl(cod)]_2$ (2.5 mg, 2.5 mol%) and norbornene (207 mg, 2.2 mmol, 11 eq), followed by the addition of (not anhydrous) IPA (10 µL) and 1,4-dioxane (1.0 mL). The reaction mixture was heated to 80 °C and stirred at that temperature for 16 hours. After this time, the reaction mixture was cooled to room temperature and the reaction mixture was filtered through Celite®, eluting with ethyl acetate. The filtrate was then concentrated under reduced pressure and a known quantity of 1,3,5-trimethoxybenzene as an internal standard was added to the residue before analysis of the crude reaction mixture by ^{1}H NMR spectroscopy. Purification by flash column chromatography and preparative HPLC using stated eluent systems was undertaken to afford isolated products.

General Procedure B:

Under a nitrogen atmosphere an over-dried Schlenk tube (15 mm diameter) was charged with the polyaromatic track (0.20 mmol, 1.0 eq), CsF (61 mg, 0.40 mmol, 2.0 eq), dppp (10 mg, 10 mol%), $[RhCl(cod)]_2$ (5.0 mg, 5.0 mol%) and norbornene (207 mg, 2.2 mmol, 11 eq), followed by the addition of (not anhydrous) IPA (10 µL) and1,4-dioxane (1.0 mL). The reaction mixture was heated to 80 °C and stirred at that temperature for 16 hours. After this time, the reaction mixture was cooled to room temperature and the reaction mixture was filtered through Celite®, eluting with ethyl acetate. The filtrate was then concentrated under reduced pressure and a known quantity of 1,3,5-trimethoxybenzene as an internal standard was added to the residue before analysis of the crude reaction mixture by ^{1}H NMR spectroscopy. Purification by flash column chromatography and preparative HPLC using stated eluent systems was undertaken to afford isolated products.

General Procedure C:

Under a nitrogen atmosphere an over-dried Schlenk tube (15 mm diameter) was charged with the polyaromatic track (0.20 mmol, 1.0 eq), CsF (61 mg, 0.40 mmol, 2.0 eq), dppp (20 mg, 20 mol%), $[RhCl(cod)]_2$ (10 mg, 10 mol%) and norbornene (414 mg, 4.4 mmol, 22 eq), followed by the addition of (not anhydrous) IPA (10 µL) and1,4-dioxane (1.0 mL). The reaction mixture was heated to 80 °C and stirred at that temperature for 16 hours. After this time, the reaction mixture was cooled to room

temperature and the reaction mixture was filtered through Celite®, eluting with hexane. The filtrate was then concentrated under reduced pressure.

General Procedure D:

Under a nitrogen atmosphere an over-dried Schlenk tube (15 mm diameter) was charged with the polyaromatic track (0.10 mmol, 1.0 eq), CsF (30 mg, 0.20 mmol, 2.0 eq), dppp (10 mg, 20 mol%), $[RhCl(cod)]_2$ (5.0 mg, 10 mol%) and norbornene (276 mg, 2.9 mmol, 29 eq), followed by the addition of (not anhydrous) IPA (10 µL) and1,4-dioxane (1.0 mL). The reaction mixture was heated to 80 °C and stirred at that temperature for 16 hours. After this time, the reaction mixture was cooled to room temperature and the reaction mixture was filtered through Celite®, eluting with hexane. The filtrate was then concentrated under reduced pressure.

3.2) Analysis of Reactions using ^{1}H NMR Spectroscopy

Yields were determined using ^{1}H NMR spectroscopy using the following protocol: a known amount of 1,3,5-trimethoxybenzene was added to the concentrated filtrate of the crude mixture before the whole material was dissolved in $CDCl_3$ (~0.7 mL), from which a sample was removed and analysed by ^{1}H NMR spectroscopy. By comparing the integral of the aromatic signal of 1,3,5-trimethoxybenzene (5.99 ppm) to a signal corresponding to one proton of the trisubstituted product the number of mmol of trisubstituted product could be calculated.

In a typical ^{1}H NMR spectrum of a crude reaction mixture comprising di- and tri-substituted biaryl track, the signals for the benzylic protons of the norbornane substituents on the first ring are observed in the region 2.8–3.0 ppm, while the signals for the benzylic proton of the norbornane substituent on the second ring are observed in the region 2.6–2.8 ppm. By analysing the relative integrals of the signals in these two regions, in combination with the integral of the aromatic signal of 1,3,5-trimethoxybenzene, yields of the di- and tri-substituted products (and their ratio) can be determined.

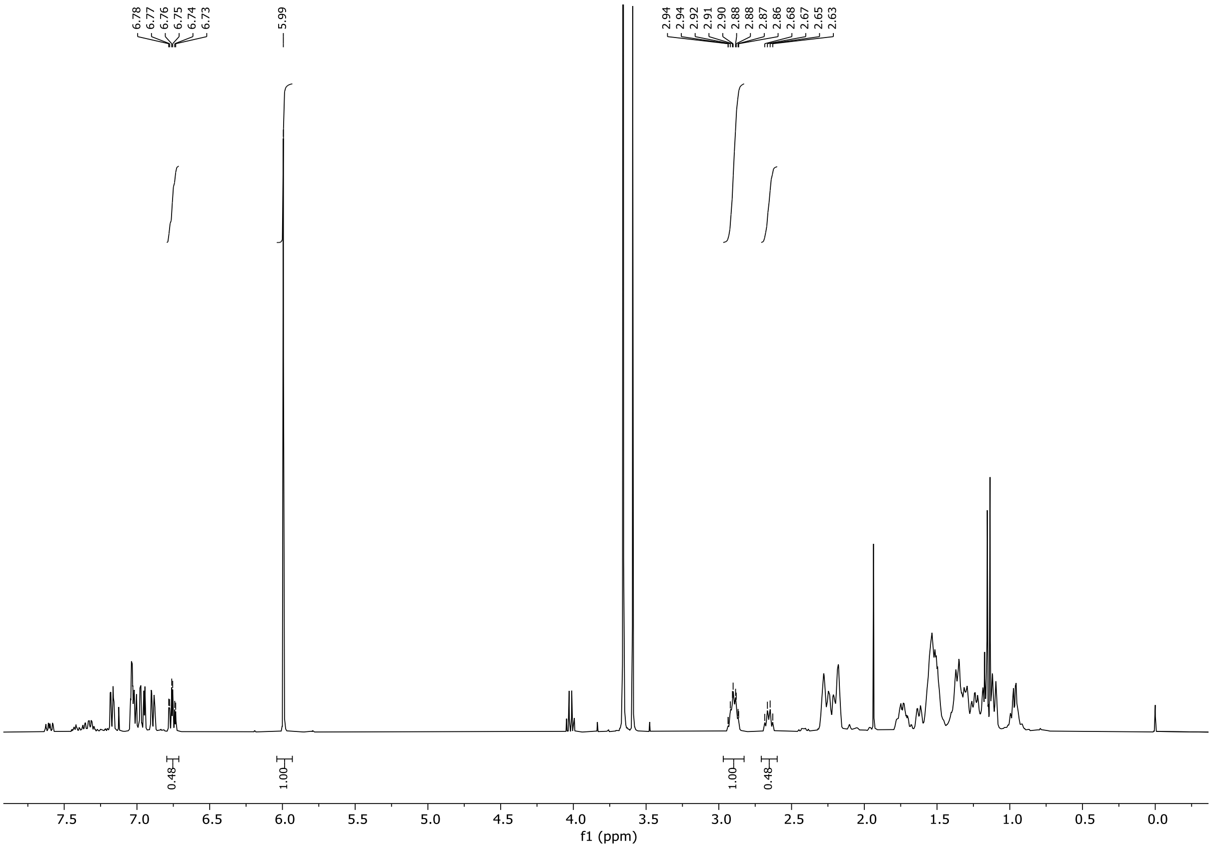

Figure S1 ^{1}H NMR spectrum of crude reaction mixture of **1** subjected to General Procedure A with 1,3,5-trimethoxybenzene as an internal standard (approx. 0.20 mmol).

Reactions were generally conducted in duplicate, triplicate or quadruplate and ^{1}H NMR yields for these individual runs are provided in the tables associated with each polyaromatic track. The isolated yields reported in Figures 2 and 3 and accompanying text of the manuscript derive from a single run and these are clearly indicated in the tables.

3.3) Details on Characterisation and Representation of Polysubstituted Polyaromatic Tracks

Polyaromatic tracks substituted with multiple norbornane units comprise two enantiomeric sets of $2^{(n-1)}$ diastereoisomers, where n = number of norbornane units (e.g., a track substituted with three norbornane units comprises two enantiomeric sets of four diastereoisomers).

Polysubstituted tracks are isolated as mixtures of stereoisomers and no attempt is made to deconvolute the stereoisomer mixture, although the presence of stereoisomers is evident from the ^{1}H, ^{13}C, and ^{19}F NMR spectra (i.e., more signals present than expected for a single stereoisomer).

For clarity, all incorporated norbornane units are depicted arbitrarily as (*R*,*R*,*S*) in the polysubstituted track structures.

For some polysubstituted tracks, the aliphatic region of the ^{1}H NMR spectrum over-integrates. This over-integration is attributed to the presence of a signal corresponding to H_2O (1.56 ppm in $CDCl_3$) that falls in this region. To account for this, integrals of the aliphatic region are reported with "+ H_2O (XH)" where appropriate.

3.4) Selected Optimisation for the Rhodium-Catalysed Incorporation of Norbornene into 2-(4'-Fluoro-[1,1'-biphenyl]-4-yl)-4,4,5,5-tetramethyl-1,3,2-dioxaborolane (1)

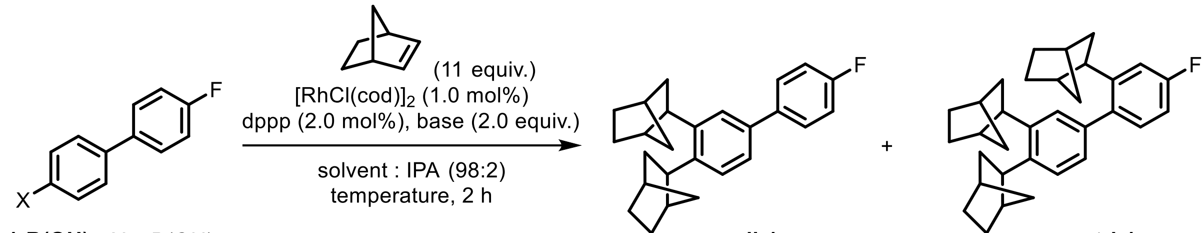

1-B(OH)$_2$, X = B(OH)$_2$
1, X = Bpin

Table S1 below details selected optimisation studies for the development of the rhodium-catalysed incorporation of norbornene into track **2-(4'-fluoro-[1,1'-biphenyl]-4-yl)-4,4,5,5-tetramethyl-1,3,2-dioxaborolane (1)**. The optimisation studies used reaction conditions from the iterative C–H alkylation process reported by Miura and co-workers as a starting point;[7] during the initial replication of the literature results, the inclusion of IPA as a co-solvent was identified as beneficial to reproducible reactivity in our hands and was used thereafter. Initial studies (Entries 1–14) were conducted on the corresponding boronic acid derivative (**4'-fluoro-[1,1'-biphenyl]-4-yl)boronic acid (1-B(OH)$_2$)**). When the optimal conditions (Entry 7) were applied to track **2-(4'-fluoro-[1,1'-biphenyl]-4-yl)-4,4,5,5-tetramethyl-1,3,2-dioxaborolane (1)**, identical results were obtained (Entry 16); all subsequent studies used the corresponding pinacolato-derived tracks due to their favourable solubility profile. To allow optimal comparison of biaryl track substrates, the rhodium-catalysed incorporation of norbornene into **2-(4'-fluoro-[1,1'-biphenyl]-4-yl)-4,4,5,5-tetramethyl-1,3,2-dioxaborolane (1)** was also conducted at higher loadings of [RhCl(cod)]$_2$ (2.5 mol%) and dppp (5 mol%) and for a longer reaction time (16 h) (Entry 17).

Table S1 Selected optimisation studies: **1** or **1-B(OH)$_2$** (0.20 mmol), norbornene (11 equiv.), [RhCl(cod)]$_2$ (1.0 mol%), dppp (2.0 mol%), base (2.0 equiv.), solvent : IPA (98:2 v:v), 2 h; [a] determined by ^{1}H NMR spectroscopy using 1,3,5-trimethoxybenzene as an internal standard; [b] determined by ^{19}F NMR spectroscopy using 4,4'-difluoro-1,1'-biphenyl as an internal standard; [c] no [RhCl(cod)]$_2$; [d] no dppp; [e] no CsF; [f] no norbornene; [g] norbornene replaced with cyclohexene (11 equiv.); [h] **1** (0.20 mmol), norbornene (11 equiv.), [RhCl(cod)]$_2$ (2.5 mol%), dppp (5.0 mol%), CsF (2.0 equiv.), 1,4-dioxane : IPA (98:2), 80 °C, 16 h, isolated yield of **tri-1** in parentheses; [i] **1** (0.20 mmol), norbornadiene (11 equiv.), [RhCl(cod)]$_2$ (2.5 mol%), dppp (5.0 mol%), CsF (2.0 equiv.), 1,4-dioxane : IPA (98:2), 80 °C, 16 h, no reaction observed.

entry	X	solvent	base	temperature (°C)	tri-1 (%, NMR yield[a])	tri-1 : di-1[b]	deboronation (%, NMR yield[b])
1	B(OH)$_2$	PhMe	CsF	100	79	95 : 5	10
2	B(OH)$_2$	PhMe	Cs_2CO_3	100	71	97 : 3	6
3	B(OH)$_2$	1,4-dioxane	CsF	100	78	98 : 2	3
4	B(OH)$_2$	1,4-dioxane	Cs_2CO_3	100	83	98 : 2	4

5	$B(OH)_2$	PhMe	CsF	80	72	94 : 6	4
6	$B(OH)_2$	PhMe	Cs_2CO_3	80	71	93 : 7	8
7	$B(OH)_2$	1,4-dioxane	CsF	80	92	98 : 2	2
8	$B(OH)_2$	1,4-dioxane	Cs_2CO_3	80	76	95 : 5	3
9	$B(OH)_2$	PhMe	CsF	50	76	97 : 3	4
10	$B(OH)_2$	1,4-dioxane	CsF	50	52	94 : 6	6
11[c]	$B(OH)_2$	1,4-dioxane	CsF	80	0	n.d.	n.d.
12[d]	$B(OH)_2$	1,4-dioxane	CsF	80	5	55.5 : 44.5	22
13[e]	$B(OH)_2$	1,4-dioxane	CsF	80	0	n.d.	n.d.
14[f]	$B(OH)_2$	1,4-dioxane	CsF	80	0	n.d.	96
15[g]	B(pin)	1,4-dioxane	CsF	80	0	n.d.	100
16	B(pin)	1,4-dioxane	CsF	80	90	98 : 2	n.d.
17[h]	B(pin)	1,4-dioxane	CsF	80	91 (84)	98 : 2	n.d.
18[i]	B(pin)	1,4-dioxane	CsF	80	0	0	0

3.5) Biaryl Boronic Acid Pinacol Ester Tracks 1–8 as Substrates for Rh-Catalysed Norbornane Incorporation

2-(4'-Fluoro-[1,1'-biphenyl]-4-yl)-4,4,5,5-tetramethyl-1,3,2-dioxaborolane (1)

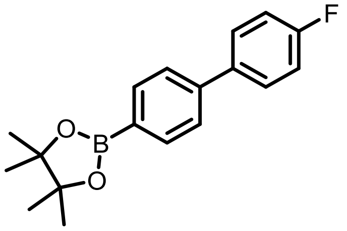

Track **2-(4'-fluoro-[1,1'-biphenyl]-4-yl)-4,4,5,5-tetramethyl-1,3,2-dioxaborolane (1)** was subjected to General Procedure A and the crude reaction mixture analysed by ^{1}H NMR spectroscopy (Table S2).

Table S2 ^{1}H NMR analysis for **1** subjected to General Procedure A; yields determined using 1,3,5-trimethoxybenzene as an internal standard as described in Supplementary Information Section 3.2.

**entry**	**tri-1 (%, NMR yield)**	**di-1 (%, NMR yield)**	**Tri-1 (%, isolated)**
1	91	2	76 mg, 84%
2	91	4	
3	81	3	
4	84	1	

Entry 1 was purified by flash column chromatography on silica gel (hexane, isocratic), followed by further purification with preparative HPLC (hexane, isocratic), to afford **tri-1** (76 mg, 84%) as a colourless oil.

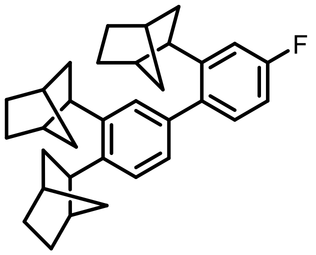

***R*f** (hexane): 0.30. **^{1}H NMR** (400 MHz, $CDCl_3$) δ 7.29 (dd, J = 8.0, 2.0 Hz, 1H), 7.19 – 7.11 (m, 2H), 7.08 (dd, J = 11.2, 2.7 Hz, 1H), 7.01 (dd, J = 7.9, 1.9 Hz, 1H), 6.88 (td, J = 8.3, 2.7 Hz, 1H), 3.07 – 2.96 (m, 2H), 2.82 – 2.72 (m, 1H), 2.48 – 2.23 (m, 6H), 1.93 – 1.78 (m, 2H), 1.78 – 1.56 (m, 9H), 1.56 – 1.19 (m, 9H + H_2O (2H)), 1.15 – 1.03 (m, 2H). **^{13}C NMR** (101 MHz, $CDCl_3$) δ 162.3 (d, $^{1}J_{C–F}$ = 244.0 Hz), 148.1, 148.0, 144.9, 144.9, 144.8, 143.8, 143.8, 143.8, 138.6, 138.5, 138.4, 131.6, 131.6, 131.5, 131.5, 127.1, 127.0, 127.0, 126.5, 126.5, 125.2, 125.1, 125.1, 125.1, 112.7 (d, $^{2}J_{C–F}$ = 21.5 Hz), 111.7

(d, $^{2}J_{C\text{–}F}$ = 20.9 Hz), 43.8, 43.6, 43.5, 43.5, 43.4, 43.2, 43.2, 43.1, 43.0, 43.0, 43.0, 42.9, 42.8, 42.7, 40.5, 40.5, 40.4, 40.3, 40.3, 40.2, 40.2, 40.1, 40.0, 37.1, 37.1, 37.0, 37.0, 36.9, 36.9, 36.8, 36.8, 36.8, 36.7, 36.7, 36.7, 36.6, 31.3, 31.2, 31.2, 30.6, 29.1, 29.1, 29.0, 28.6. **^{19}F NMR {^{1}H}** (377 MHz, $CDCl_3$) δ −115.48 (s), −115.49 (s), −115.49 (s), −115.51 (s). **IR** ν (cm^{-1}): 2948, 2868, 1605, 1583, 1482, 1472, 1454, 1417, 1354, 1310, 1297, 1267, 1243, 1213, 1155. **HMRS** (Negative ion, MALDI) *m/z*: Calculated for $C_{33}H_{38}F$ 454.3036. Found 453.2961 $[M – H]^{-}$.

Di-1 could be isolated from a separate reaction to provide analytically pure material for characterisation.

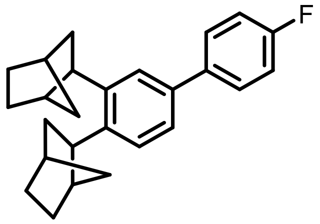

***R*f** (hexane): 0.30. **^{1}H NMR** (500 MHz, $CDCl_3$): δ 7.55 – 7.49 (m, 2H), 7.43 – 7.40 (m, 1H), 7.35 – 7.27 (m, 2H), 7.14 – 7.07 (m, 2H), 3.04 – 2.93 (m, 2H), 2.41 – 2.30 (m, 4H), 1.90 – 1.77 (m, 2H), 1.73 – 1.58 (m, 8H), 1.43 – 1.25 (m, 6H). **^{13}C NMR** (126 MHz, $CDCl_3$, polynomial fit method (polynomial order 15) applied) δ 162.3 (d, $^{1}J_{C\text{–}F}$ = 245.5 Hz), 145.9, 145.8, 144.6, 144.6, 137.9, 137.9, 137.2, 137.2, 128.7 (d, $^{3}J_{C\text{–}F}$ = 7.9 Hz), 126.1, 126.1, 124.4, 124.0, 123.9, 115.6 (d, $^{2}J_{C\text{–}F}$ = 21.3 Hz), 43.2, 43.1, 43.0, 43.0, 43.0, 42.9, 42.7, 42.7, 40.1, 40.1, 40.0, 40.0, 37.0, 37.0, 36.9, 36.9, 36.8, 36.7, 36.7, 36.6, 31.2, 31.1, 29.1, 29.1. **^{19}F NMR {^{1}H}** (377 MHz, $CDCl_3$): δ −116.53 (s), −116.54 (s). **IR** ν (cm^{-1}): 2951, 2868, 1513, 1488, 1232, 818. **HRMS** (APCI) *m/z*: Calculated for $C_{26}H_{29}F$ 360.2253. Found 361.2337 $[M + H]^{+}$.

4,4,5,5-Tetramethyl-2-(4'-(trifluoromethyl)-[1,1'-biphenyl]-4-yl)-1,3,2-dioxaborolane (2)

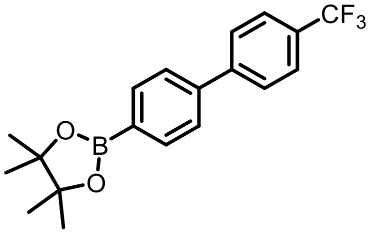

Track **4,4,5,5-tetramethyl-2-(4'-(trifluoromethyl)-[1,1'-biphenyl]-4-yl)-1,3,2-dioxaborolane (2)** was subjected to General Procedure B and the crude reaction mixture analysed by ^{1}H NMR spectroscopy (Table S3).

Table S3 ^{1}H NMR analysis for **2** subjected to General Procedure B; yields determined using 1,3,5-trimethoxybenzene as an internal standard as described in Supplementary Information Section 3.2.

entry	tri-2 (%, NMR yield)	di-2 (%, NMR yield)	tri-2 (%, isolated)
1	63	17	
2	70	24	
3	68	18	62 mg, 61%

Entry 3 was purified by flash column chromatography on silica gel (hexane, isocratic), followed by further purification with preparative HPLC (hexane, isocratic), to afford **tri-2** (62 mg, 61%) as a colourless oil.

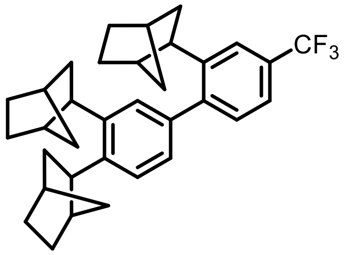

***R*f** (hexane): 0.30. **^{1}H NMR** (400 MHz, $CDCl_3$) δ 7.63 – 7.59 (m, 1H), 7.49 – 7.42 (m, 1H), 7.34 – 7.27 (m, 2H), 7.21 – 7.11 (m, 1H), 7.05 – 6.98 (m, 1H), 3.07 – 2.96 (m, 2H), 2.88 – 2.78 (m, 1H), 2.44 – 2.23 (m, 6H), 1.94 – 1.16 (m, 24H + H_2O (2H)), 1.13 – 1.05 (m, 2H). **^{13}C NMR** (101 MHz, $CDCl_3$) δ 146.4, 145.1, 145.0, 145.0, 144.4, 144.3, 144.3, 138.1, 130.6, 130.6, 129.4, 129.1, 126.6, 126.5, 126.5, 126.0, 125.3, 125.2, 125.2, 125.2, 123.3, 122.5, 122.0, 121.9, 43.6, 43.5, 43.4, 43.3, 43.3, 43.1, 43.1, 43.0, 43.0, 43.0, 43.0, 42.8, 42.7, 42.6, 40.4, 40.3, 40.3, 40.2, 40.2, 40.1, 40.1, 40.0, 37.1, 37.0, 37.0, 37.0, 36.9, 36.9, 36.8, 36.8, 36.7, 36.7, 36.6, 36.6, 31.2, 31.2, 31.1, 31.1, 30.6, 29.1, 29.0, 29.0, 28.5. **^{19}F NMR** (376 MHz, $CDCl_3$) δ −62.09. **IR** ν (cm^{-1}): 2949, 2868 2908, 2104, 1892, 1547, 1476 1447,

1303, 1296. **HRMS** (Negative ion, MALDI) *m/z*: Calculated for $C_{34}H_{39}F_3$ 504.3004. Found 504.3016 $[M]^{-}$.

di-2 could be isolated (7 mg, 3%, **di-2** : **tri-2** = 6 : 1) as a colourless oil.

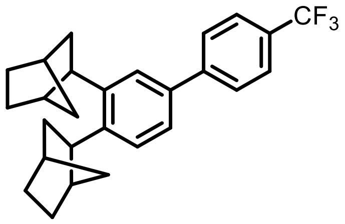

***R*f** (hexane): 0.30. **^{1}H NMR** (500 MHz, $CDCl_3$) δ 7.67 (s, 4H, H-6 + H-7), 7.50 – 7.46 (m, 1H, H-3), 7.38 – 7.33 (m, 2H, H-10 + H-11), 3.09 – 2.95 (m, 2H, H-12 + H-13), 2.42 – 2.29 (m, 6H), 1.92 – 1.16 (m, 14H + H_2O (15H)). **^{13}C NMR** (126 MHz, $CDCl_3$) δ 146.1, 146.1, 145.8, 145.7, 145.4, 136.8, 130.6, 130.4, 129.1, 128.9, 127.4, 126.3, 125.8, 125.8, 125.7, 125.7, 125.6, 125.4, 124.9, 124.7, 124.3, 124.2, 123.5, 43.6, 43.2, 43.2, 43.1, 43.1, 43.0, 43.0, 42.7, 42.7, 41.6, 40.5, 40.2, 40.1, 38.8, 37.1, 37.1, 37.0, 37.0, 36.9, 36.9, 36.8, 36.7, 36.7, 36.5, 31.2, 31.2, 31.2, 31.1, 30.7, 30.6, 29.3, 29.1, 28.6. **^{19}F NMR** (377 MHz, $CDCl_3$) δ -62.31 (s). **IR** ν (cm^{-1}): 2940, 2873, 2115, 1892, 1584, 1418, 1300. **HRMS** (EI) *m/z*: Calculated for $C_{27}H_{29}F_3$ 410.2221. Found 410.2213 $[M]^{+}$.

2-(4'-Methoxy-[1,1'-biphenyl]-4-yl)-4,4,5,5-tetramethyl-1,3,2-dioxaborolane (3)

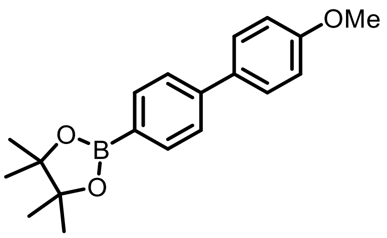

Track **2-(4'-methoxy-[1,1'-biphenyl]-4-yl)-4,4,5,5-tetramethyl-1,3,2-dioxaborolane (3)** was subjected to General Procedure B and the crude reaction mixture analysed by ^{1}H NMR spectroscopy (Table S4).

Table S4 ^{1}H NMR analysis for **3** subjected to General Procedure B; yields determined using 1,3,5-trimethoxybenzene as an internal standard as described in Supplementary Information Section 3.2.

entry	tri-3 (%, NMR yield)	di-3 (%, NMR yield)	tri-3 (%, isolated)
1	83	8	
2	90	2	81 mg, 87%

Entry 2 was purified by flash column chromatography on silica gel (hexane:EtOAc, 98:2), followed by further purification with preparative HPLC (hexane:EtOAc, gradient, 100:0–98:2), to afford **tri-3** (81 mg, 87%) as a colourless oil.

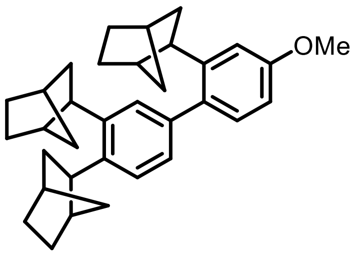

***R*f** (hexane:EtOAc, 98:2): 0.20. **^{1}H NMR** (400 MHz, $CDCl_3$) δ 7.28 (dd, *J* = 8.1, 2.1 Hz, 1H), 7.19 – 7.11 (m, 2H), 7.06 – 7.00 (m, 1H), 6.95 (d, *J* = 2.6 Hz, 1H), 6.76 (dd, *J* = 8.3, 2.6 Hz, 1H), 3.86 (s, 3H), 3.05 – 2.95 (m, 2H), 2.84 – 2.71 (m, 1H), 2.46 – 2.27 (m, 6H), 1.91 – 1.25 (m, 20H + H_2O (1H)), 1.25 – 1.17 (m, 2H), 1.15 – 1.04 (m, 2H). **^{13}C NMR** (101 MHz, $CDCl_3$) δ 158.8, 147.1, 144.7, 144.7, 144.7, 143.4, 143.4, 143.4, 139.0, 135.5, 131.2, 131.2, 127.3, 127.2, 127.2, 126.7, 126.6, 125.0, 125.0, 125.0, 125.0, 112.4, 112.4, 109.5, 109.5, 55.3, 43.8, 43.6, 43.6, 43.5, 43.5, 43.1, 43.1, 43.0, 43.0, 43.0, 42.9, 42.8, 42.6, 40.5, 40.5, 40.3, 40.3, 40.3, 40.2, 40.1, 40.1, 40.0, 37.1, 37.0, 37.0, 37.0, 36.9, 36.8, 36.8, 36.7, 36.7, 36.6, 36.5, 31.3, 31.2, 31.2, 31.1, 30.7, 29.1, 29.1, 28.6. **IR** ν (cm^{-1}): 2946, 2889, 2066,

1607, 1579, 1442, 1335. **HRMS** (MALDI) *m/z*: Calculated for $C_{34}H_{42}O$ 466.3230. Found 466.3236 $[M]^+$.

2-(3'-Methoxy-[1,1'-biphenyl]-4-yl)-4,4,5,5-tetramethyl-1,3,2-dioxaborolane (4)

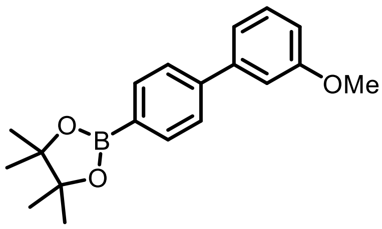

Track **2-(3'-methoxy-[1,1'-biphenyl]-4-yl)-4,4,5,5-tetramethyl-1,3,2-dioxaborolane (4)** was subjected to General Procedure B and the crude reaction mixture analysed by ^{1}H NMR spectroscopy (Table S5).

Table S5 ^{1}H NMR analysis for **4** subjected to General Procedure B; yields determined using 1,3,5-trimethoxybenzene as an internal standard as described in Supplementary Information Section 3.2; NMR yield of **tetra-4** and unconfirmed additional **polysubstituted-4**.

entry	polysubstituted-4 (%, NMR yield)	tetra-4 (%, isolated)
1	76	
2	86	95 mg, 84%

Entry 2 was purified by flash column chromatography on silica gel (hexane:EtOAc, 98:2), followed by further purification with preparative HPLC (hexane:EtOAc, gradient, 100:0–98:2), to afford **tetra-4** (95 mg, 84%) as a colourless oil.

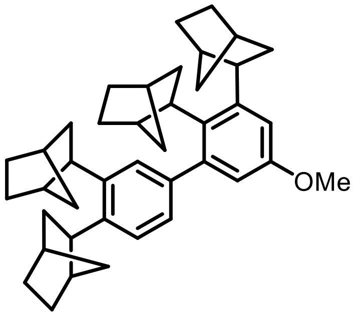

***R*f** (hexane:EtOAc, 98:2): 0.20. **^{1}H NMR** (600 MHz, $CDCl_3$) δ 7.24 – 6.92 (m, 3H), 6.89 (dd, *J* = 11.9, 2.9 Hz, 1H), 6.52 – 6.41 (m, 1H), 3.81 – 3.70 (m, 3H), 3.10 – 2.90 (m, 4H), 2.52 – 0.99 (m, 38H + H_2O (1H)), 0.67 – 0.48 (m, 1H), 0.20 – 0.00 (m, 1H). **^{13}C NMR** (151 MHz, $CDCl_3$) δ 155.8, 155.8, 148.3, 147.9, 147.9, 147.9, 144.8, 144.8, 144.6, 144.2, 143.8, 143.8, 143.7, 141.4, 141.4, 141.4, 134.8, 134.8, 134.8, 127.8, 127.5, 127.3, 127.3, 127.2, 127.1, 127.1, 126.7, 126.7, 124.6, 124.5, 124.4, 124.4, 124.2,

113.4, 113.4, 113.3, 113.3, 113.3, 113.3, 112.3, 112.3, 112.2, 112.2, 112.2, 55.3, 44.7, 44.7, 44.7, 44.7, 44.3, 44.3, 44.3, 44.2, 43.7, 43.7, 43.6, 43.6, 43.6, 43.6, 43.6, 43.5, 43.5, 43.4, 43.4, 43.4, 43.4, 43.3, 43.3, 43.2, 43.2, 43.2, 43.1, 43.1, 43.1, 43.0, 43.0, 42.9, 42.9, 42.9, 42.8, 42.8, 42.7, 42.7, 42.7, 42.7, 42.6, 42.4, 42.4, 42.4, 42.3, 42.3, 42.3, 41.9, 41.9, 41.8, 41.8, 41.8, 41.7, 41.7, 41.7, 41.6, 41.1, 41.1, 41.1, 41.0, 40.9, 40.9, 40.9, 40.8, 40.8, 40.7, 40.6, 40.6, 39.9, 39.9, 39.9, 39.8, 39.8, 39.3, 39.2, 39.2, 39.1, 39.0, 39.0, 37.2, 37.2, 37.2, 37.1, 37.1, 37.0, 37.0, 37.0, 37.0, 36.9, 36.9, 36.9, 36.8, 36.7, 36.7, 36.7, 36.6, 36.6, 36.6, 36.5, 36.5, 36.4, 36.3, 36.3, 36.2, 36.2, 36.1, 36.1, 34.5, 34.5, 34.5, 34.4, 34.3, 34.2, 34.2, 34.2, 34.2, 31.3, 31.3, 31.3, 31.2, 31.2, 31.2, 31.2, 31.1, 31.0, 30.9, 30.9, 29.8, 29.2, 29.2, 29.1, 29.1, 29.1, 29.1, 28.0, 28.0, 28.0, 27.9, 27.9. **IR** ν (cm^{-1}): 2946, 2868, 2049, 1597, 1477, 1452, 1426. **HRMS** (Negative ion, MALDI) *m/z*: Calculated for $C_{41}H_{52}O$ 560.4018. Found 545.3793 $[M – Me]^{–}$.

4,4,5,5-Tetramethyl-2-(4'-methyl-[1,1'-biphenyl]-4-yl)-1,3,2-dioxaborolane (5)

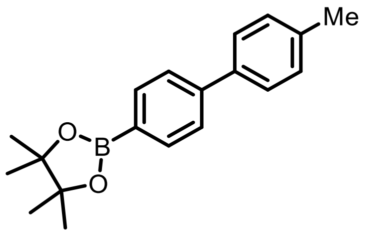

Track **4,4,5,5-tetramethyl-2-(4'-methyl-[1,1'-biphenyl]-4-yl)-1,3,2-dioxaborolane (5)** was subjected to General Procedure B and the crude reaction mixture analysed by ^{1}H NMR spectroscopy (Table S6).

Table S6 ^{1}H NMR analysis for **5** subjected to General Procedure B; yields determined using 1,3,5-trimethoxybenzene as an internal standard as described in Supplementary Information Section 3.2.

entry	tri-5 (%, NMR yield)	di-5 (%, NMR yield)	tri-5 (%, isolated)
1	73	12	
2	77	15	
3	78	19	65 mg, 72%

Entry 3 was purified by flash column chromatography on silica gel (hexane, isocratic), followed by further purification with preparative HPLC (hexane, isocratic), to afford **tri-5** (65 mg, 72%) as a colourless oil.

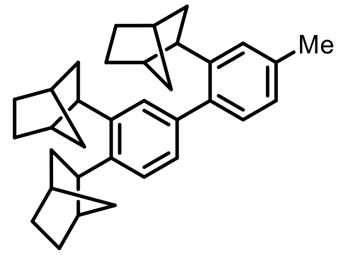

$\boldsymbol{R}_f$ (hexane): 0.30. ^{1}**H NMR** (400 MHz, $CDCl_3$) δ 7.29 (dd, J = 7.9, 2.0 Hz, 1H), 7.25 – 7.15 (m, 2H), 7.11 (d, J = 7.7 Hz, 1H), 7.07 – 7.01 (m, 2H), 3.05 – 2.95 (m, 2H), 2.82 – 2.74 (m, 1H), 2.44 – 2.27 (m, 9H), 1.91 – 1.56 (m, 11H + H_2O (2H)), 1.52 – 1.02 (m, 13H). ^{13}**C NMR** (101 MHz, $CDCl_3$) δ 145.4, 144.7, 144.7, 144.7, 143.5, 143.5, 143.4, 140.1, 140.1, 140.0, 139.3, 139.3, 136.7, 136.6, 130.3, 130.3, 127.2, 127.1, 127.0, 126.6, 126.5, 126.5, 126.5, 126.5, 126.4, 125.9, 125.0, 125.0, 125.0, 125.0, 43.7, 43.7, 43.6, 43.6, 43.2, 43.2, 43.1, 43.1, 43.0, 43.0, 42.9, 42.8, 42.7, 40.4, 40.4, 40.4, 40.3, 40.3, 40.2, 40.2, 40.2, 40.1, 40.0, 37.1, 37.1, 37.0, 37.0, 36.9, 36.9, 36.8, 36.8, 36.7, 36.7, 36.6, 36.6, 31.3, 31.3, 31.2, 31.2, 31.2, 30.7, 29.1, 29.1, 29.1, 28.7, 21.7.

IR ν (cm^{-1}): 2947, 2867, 1609, 1484, 1473, 1453, 1353, 1310, 1297, 1264, 1215, 1138. **HRMS** (Negative ion, MALDI) *m/z*: Calculated for $C_{34}H_{42}$ 450.3287. Found 449.3271 $[M-H]^{-}$.

A sample enriched in **di-5** could be isolated (**di-5** : **tri-5** = 1.37:1.0) and was used to identify the signals corresponding to **di-5**.

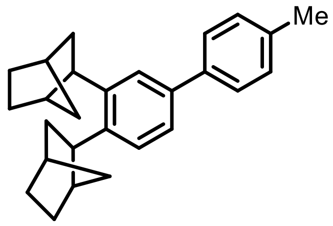

R*f** (hexane): 0.30. **^{1}H NMR** (500 MHz, $CDCl_3$) *The aromatic signals corresponding to **di-5 δ 7.61 – 7.57 (m, 1H), 7.53 – 7.46 (m, 2H), 7.46 – 7.41 (m, 1H), 7.36 – 7.31 (m, 1H), 7.26 – 7.24 (m, 2H).

All signals for the mixture of **di-5** and **tri-5** are listed below.

^{1}H NMR (500 MHz, $CDCl_3$) δ 7.61 – 7.57 (m), 7.53 – 7.46 (m), 7.46 – 7.41 (m), 7.36 – 7.31 (m), 7.29 – 7.26 (m), 7.26 – 7.24 (m), 7.22 – 7.15 (m), 7.12 – 7.08 (m), 7.05 – 7.00 (m), 3.05 – 2.91 (m), 2.81 – 2.72 (m), 2.55 – 2.20 (m), 1.91 – 0.97 (m). **^{13}C NMR** (126 MHz, $CDCl_3$, polynomial fit method (polynomial order 15) applied) δ 145.7, 145.7, 145.4, 145.4, 144.7, 144.7, 144.7, 144.3, 144.3, 143.8, 143.5, 143.5, 143.5, 143.4, 142.5, 141.8, 141.7, 141.3, 140.1, 140.1, 140.1, 139.3, 139.3, 139.3, 139.0, 138.5, 138.4, 138.2, 138.2, 137.2, 136.8, 136.7, 136.7, 136.0, 135.8, 130.3, 130.3, 129.6, 129.6, 129.6, 129.5, 128.8, 128.7, 127.6, 127.2, 127.1, 127.1, 127.1, 127.1, 127.0, 126.9, 126.9, 126.6, 126.5, 126.5, 126.5, 126.5, 126.4, 126.0, 126.0, 125.9, 125.0, 125.0, 125.0, 125.0, 124.5, 124.5, 124.0, 123.9, 60.6, 47.2, 44.6, 43.7, 43.7, 43.6, 43.6, 43.3, 43.2, 43.2, 43.1, 43.1, 43.1, 43.1, 43.0, 43.0, 43.0, 42.9, 42.9, 42.8, 42.8, 42.7, 42.7, 42.1, 41.9, 41.3, 40.4, 40.4, 40.4, 40.4, 40.3, 40.3, 40.2, 40.2, 40.2, 40.1, 40.1, 40.0, 40.0, 39.9, 39.8, 39.3, 37.7, 37.3, 37.3, 37.1, 37.1, 37.1, 37.0, 37.0, 37.0, 36.9, 36.9, 36.8, 36.8, 36.8, 36.8, 36.8, 36.7, 36.7, 36.7, 36.6, 36.6, 36.6, 36.6, 36.3, 34.3, 34.0, 33.9, 31.3, 31.3, 31.2, 31.2, 31.2, 30.7, 29.9, 29.1, 29.1, 29.1, 29.1, 28.7, 28.1, 27.9, 27.7, 24.2, 23.9, 21.7, 21.2, 21.2. **IR** ν (cm^{-1}):2949, 2986, 2901, 2868, 1901, 1709, 1508, 1445, 1400. **HRMS** (EI) *m/z*: Calculated for $C_{27}H_{32}$ 356.2493. Found 356.2499 $[M]^+$.

2-(4'-Fluoro-2-methyl-[1,1'-biphenyl]-4-yl)-4,4,5,5-tetramethyl-1,3,2-dioxaborolane (6)

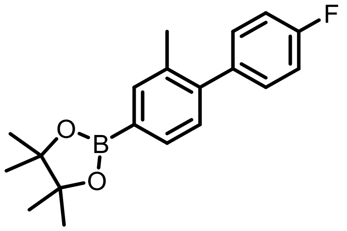

Track **2-(4'-fluoro-2-methyl-[1,1'-biphenyl]-4-yl)-4,4,5,5-tetramethyl-1,3,2-dioxaborolane (6)** was subjected to General Procedure A and the crude reaction mixture analysed by ^{1}H NMR spectroscopy (Table S7).

Table S7 ^{1}H NMR analysis for **6** subjected to General Procedure A; yields determined using 1,3,5-trimethoxybenzene as an internal standard as described in Supplementary Information Section 3.2.

entry	tri-6 (%, NMR yield)	di-6 (%, NMR yield)	tri-6 (%, isolated)
1	103	5	
2	96	8	
3	99	2	62 mg, 66%

Entry 3 was purified by flash column chromatography on silica gel (hexane, isocratic), followed by further purification with preparative HPLC (hexane, isocratic), to afford **tri-6** (62 mg, 66%) as a colourless oil.

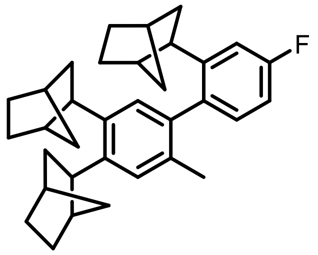

***R*f** (hexane): 0.25. **^{1}H NMR** (400 MHz, $CDCl_3$) δ 7.18 – 6.82 (m, 5H), 3.04 – 2.90 (m, 2H), 2.53 – 2.12 (m, 7H), 2.04 – 1.95 (m, 3H), 1.92 – 1.22 (m, 21H + H_2O (1H)), 1.22 – 0.97 (m, 3H). **^{13}C NMR** (101 MHz, $CDCl_3$) δ 163.4, 163.4, 161.0, 160.9, 148.7, 148.6, 148.2, 148.2, 148.2, 148.1, 144.2, 144.1, 144.0, 143.9, 142.2, 142.1, 142.1, 137.8, 137.6, 137.5, 132.3, 132.2, 131.2, 131.2, 131.1, 131.0, 131.0, 131.0, 127.7, 127.7, 127.5, 127.4, 127.1, 126.9, 126.9, 126.8, 112.8, 112.6, 112.3, 112.1, 111.8, 111.8, 111.6, 111.6, 44.0, 44.0, 43.8, 43.8, 43.8, 43.8, 43.6, 43.2, 43.2, 43.1, 42.9, 42.8, 42.8, 42.8, 42.7, 42.7, 42.6, 42.5, 42.4, 41.9, 41.9, 41.6, 41.5, 40.8, 40.4, 40.4, 40.3, 40.1, 40.0, 39.9, 39.3, 38.5, 38.5, 38.4, 37.4, 37.1, 37.0, 37.0, 37.0, 36.9, 36.9, 36.9, 36.8, 36.8, 36.7, 36.7, 36.6, 36.6, 36.3, 36.1, 35.9, 35.9,

35.8, 31.2, 31.2, 31.2, 31.1, 31.0, 30.9, 30.7, 30.6, 30.6, 29.1, 29.1, 29.1, 28.5, 28.3, 20.1, 19.9. **^{19}F NMR {^{1}H}** (377 MHz, $CDCl_3$) δ -115.53, -115.54, -115.58, -115.60. **IR** ν (cm^{-1}): 2994, 2882, 1563, 1444, 1457, 1418, 1352, 1297. **HRMS** (Negative ion, MALDI) *m/z*: Calculated for $C_{34}H_{41}F$ 468.3192. Found 467.3126 $[M-H]^{-}$.

2-(4'-Fluoro-2'-methyl-[1,1'-biphenyl]-4-yl)-4,4,5,5-tetramethyl-1,3,2-dioxaborolane (7)

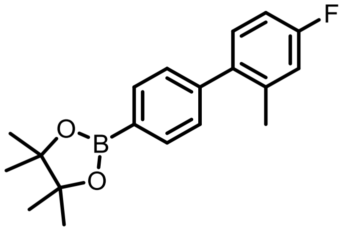

Track **2-(4'-fluoro-2'-methyl-[1,1'-biphenyl]-4-yl)-4,4,5,5-tetramethyl-1,3,2-dioxaborolane (7)** was subjected to General Procedure A and the crude reaction mixture analysed by ^{1}H NMR spectroscopy (Table S8).

Table S8 ^{1}H NMR analysis for **7** subjected to General Procedure A; yields determined using 1,3,5-trimethoxybenzene as an internal standard as described in Supplementary Information Section 3.2.

entry	tri-7 (%, NMR yield)	di-7 (%, NMR yield)	tri-7 (%, isolated)
1	94	4	
2	101	5	
3	99	1	55 mg, 60%

Entry 3 was purified by flash column chromatography on silica gel (hexane, isocratic), followed by further purification with preparative HPLC (hexane, isocratic), to afford **tri-7** (55 mg, 60%) as a colourless oil.

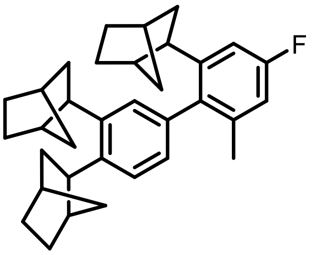

***R*f** (hexane): 0.25. **^{1}H NMR** (400 MHz, $CDCl_3$) δ 7.31 – 7.26 (m, 1H), 7.02 – 6.75 (m, 4H), 3.09 – 2.84 (m, 2H), 2.53 – 2.43 (m, 1H), 2.43 – 2.12 (m, 6H), 2.02 – 1.96 (m, 3H), 1.92 – 1.70 (m, 3H), 1.70 – 1.09 (m, 19H + H_2O (1H)), 1.07 – 0.72 (m, 2H). **^{13}C NMR** (101 MHz, $CDCl_3$) δ 161.8 (d, $^{1}J_{C\text{–}F}$ = 243.1 Hz), 148.6, 148.5, 148.4, 145.2, 145.2, 145.0, 145.0, 145.0, 144.9, 144.8, 143.6, 143.6, 143.5, 143.5, 143.5, 138.7, 138.7, 138.3, 138.2, 137.1, 137.1, 137.0, 136.9, 113.2 (d, $^{2}J_{C\text{–}F}$ = 20.5 Hz), 109.9, 109.8, 109.7, 109.6, 44.3, 44.1, 44.1, 43.9, 43.8, 43.7, 43.5, 43.3, 43.2, 43.1, 43.1, 43.1, 43.0, 43.0, 43.0, 43.0, 42.9, 42.9, 42.9, 42.8, 42.8, 42.7, 42.7, 42.6, 42.5, 42.3, 40.9, 40.7, 40.6, 40.5, 40.5, 40.5, 40.4, 40.3, 40.3, 40.1, 40.0, 39.8, 39.7, 39.6, 39.5, 39.4, 39.3, 37.1, 37.1, 37.0, 37.0, 37.0, 36.9, 36.9, 36.9, 36.8, 36.8, 36.7, 36.6, 36.6, 36.6, 36.4, 36.4, 36.2, 36.2, 36.1, 36.0, 31.2, 31.2, 31.2, 31.2, 31.1, 31.1, 30.6,

30.6, 30.6, 30.5, 30.5, 29.1, 29.1, 29.0, 29.0, 29.0, 28.6, 28.5, 21.5, 21.5, 21.5. **^{19}F NMR {^{1}H}** (377 MHz, $CDCl_3$) δ −116.32, −116.33, −116.34. **IR** ν (cm^{-1}): 2988, 2990, 2889, 1523, 1469, 1450, 1351, 1297, 1126. **HRMS** (Negative ion, MALDI) *m/z*: Calculated for $C_{34}H_{41}F$ 468.3192. Found 467.3128 $[M - H]^{-}$.

2-(4'-Fluoro-2,2'-dimethyl-[1,1'-biphenyl]-4-yl)-4,4,5,5-tetramethyl-1,3,2-dioxaborolane (8)

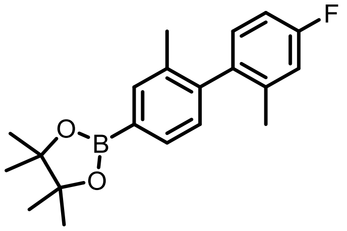

Track **2-(4'-fluoro-2,2'-dimethyl-[1,1'-biphenyl]-4-yl)-4,4,5,5-tetramethyl-1,3,2-dioxaborolane (8)** was subjected to General Procedure B and the crude reaction mixture analysed by ^{1}H NMR spectroscopy (Table S9).

Table S9 ^{1}H NMR analysis for **8** subjected to General Procedure B; yields determined using 1,3,5-trimethoxybenzene as an internal standard as described in Supplementary Information Section 3.2.

entry	di-8 (%, NMR yield)	di-8 (%, isolated)
1	55	40 mg, 51%
2	49	

Entry 1 was purified by flash column chromatography on silica gel (hexane, isocratic), followed by further purification with preparative HPLC (hexane, isocratic), to afford **di-8** (40 mg, 51%) as a colourless oil.

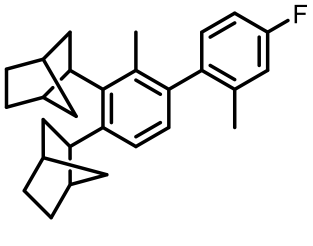

***R*f** (hexane): 0.35. **^{1}H NMR** (400 MHz, $CDCl_3$) δ 7.15 – 7.03 (m, 2H), 7.03 – 6.85 (m, 3H), 3.03 – 2.88 (m, 2H), 2.43 – 2.21 (m, 4H), 2.12 – 1.93 (m, 6H), 1.88 – 1.48 (m, 9H + H_2O (2H)), 1.49 – 1.12 (m, 7H). **^{13}C NMR** (101 MHz, $CDCl_3$) δ 161.9 (d, $^{1}J_{C\text{–}F}$ = 244.3 Hz), 144.3, 144.2, 142.6, 142.5, 138.6, 138.5, 138.1, 137.2, 137.2, 132.4, 131.1, 131.1, 131.0, 127.2, 127.1, 127.1, 127.0, 126.9, 126.9, 116.3 (d, $^{2}J_{C\text{–}F}$ = 20.8 Hz), 112.3 (d, $^{2}J_{C\text{–}F}$ = 20.7 Hz), 43.2, 43.1, 42.9, 42.9, 42.9, 42.8, 42.8, 42.8, 42.7, 42.5, 40.4, 40.2, 40.2, 40.1, 40.0, 39.9, 39.8, 39.8, 37.1, 37.0, 37.0, 36.9, 36.9, 36.7, 36.7, 36.6, 36.6, 36.5, 36.5, 36.4, 31.2, 31.2, 31.2, 31.1, 31.1, 31.1, 29.1, 29.1, 20.4, 20.4, 20.3, 19.7. **^{19}F NMR {^{1}H}** (377 MHz, $CDCl_3$) δ −116.87, −116.89, −116.89, −116.90 (note: minor additional unidentified ^{19}F signals observed at −116.63, −116.66, −116.76. **IR** ν (cm^{-1}): 2996, 2994, 2885, 1547, 1516, 1443, 1410, 1301. **HRMS** (EI) *m/z*: Calculated for $C_{28}H_{33}F$ 388.2566. Found 388.2555 $[M]^{+}$.

3.6) Triaryl Boronic Acid Pinacol Ester Track 9 as Substrate for Rh-Catalysed Norbornane Incorporation

2-(4''-Fluoro-[1,1':3',1''-terphenyl]-4-yl)-4,4,5,5-tetramethyl-1,3,2-dioxaborolane (9)

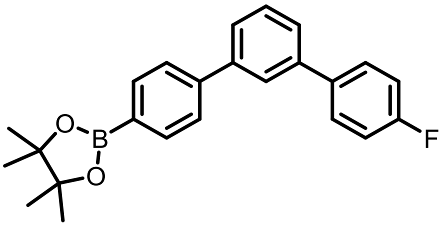

Track **2-(4''-fluoro-[1,1':3',1''-terphenyl]-4-yl)-4,4,5,5-tetramethyl-1,3,2-dioxaborolane (9)** was subjected to General Procedure B and the crude reaction mixture analysed by ^{1}H NMR spectroscopy (Table S10).

Table S10 ^{1}H NMR analysis for **9** subjected to General Procedure B; yields determined using 1,3,5-trimethoxybenzene as an internal standard as described in Supplementary Information Section 3.2.

entry	penta-9 (%, NMR yield)	penta-9 (%, isolated)
1	87	
2	94	
3	106	121 mg, 84%

Entry 3 was purified by flash column chromatography on silica gel (hexane, isocratic), followed by further purification with preparative HPLC (hexane, isocratic), to afford **penta-9** (121 mg, 84%) as a colourless oil.

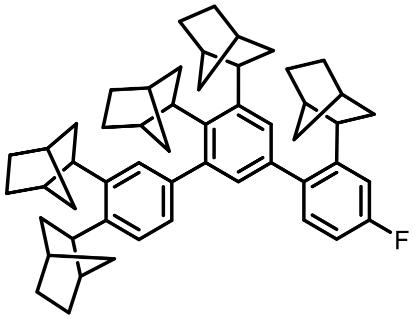

***R*f** (hexane): 0.30. **^{1}H NMR** (600 MHz, $CDCl_3$) δ 7.24 – 6.91 (m, 6H), 6.90 – 6.77 (m, 2H), 3.22 – 3.06 (m, 2H), 3.06 – 2.93 (m, 2H), 2.88 – 2.76 (m, 1H), 2.53 – 2.13 (m, 9H), 2.12 – 1.03 (m, 39H), 0.71 – 0.54 (m, 1H), 0.28 – 0.14 (m, 1H). **^{13}C NMR** (151 MHz, $CDCl_3$) δ 162.3 (d, $^{1}J_{C-F}$ = 244.0 Hz), 148.1, 148.1, 148.0, 146.3, 146.2, 146.2, 146.2, 145.8, 145.8, 145.8, 145.8, 145.7, 145.7, 144.2, 144.2, 144.1,

144.0, 144.0, 144.0, 143.9, 143.9, 143.9, 143.8, 143.8, 143.7, 143.6, 143.6, 143.6, 143.5, 143.5, 143.3, 143.3, 143.3, 143.2, 143.2, 143.1, 143.0, 143.0, 142.9, 141.4, 141.4, 141.3, 141.3, 141.3, 140.7, 140.7, 138.2, 138.2, 137.3, 137.3, 137.3, 137.2, 131.6, 131.6, 131.5, 131.5, 131.0, 131.0, 131.0, 130.9, 130.9, 130.8, 130.8, 128.1, 128.0, 128.0, 127.9, 127.8, 127.8, 127.7, 127.6, 127.6, 127.5, 127.5, 127.4, 127.3, 127.3, 127.2, 127.2, 127.1, 126.9, 126.9, 126.8, 126.8, 126.8, 126.7, 126.1, 126.1, 126.0, 126.0, 126.0, 125.9, 124.7, 124.7, 124.6, 124.6, 124.5, 124.5, 124.4, 124.4, 124.4, 124.3, 124.2, 124.2, 112.7 (d, $^{2}J_{C–F}$ = 21.3 Hz), 111.8 (d, $^{2}J_{C–F}$ = 20.8 Hz), 44.7, 44.6, 44.6, 44.6, 44.5, 44.5, 44.2, 44.2, 44.1, 44.1, 43.9, 43.9, 43.8, 43.8, 43.7, 43.7, 43.7, 43.6, 43.6, 43.5, 43.5, 43.5, 43.4, 43.4, 43.4, 43.3, 43.3, 43.3, 43.2, 43.2, 43.2, 43.2, 43.1, 43.1, 43.1, 43.0, 43.0, 42.9, 42.9, 42.9, 42.9, 42.9, 42.8, 42.8, 42.7, 42.7, 42.6, 42.5, 42.4, 42.4, 42.3, 42.1, 42.0, 42.0, 41.9, 41.9, 41.8, 41.8, 41.8, 41.7, 41.7, 41.7, 41.6, 41.0, 40.9, 40.9, 40.8, 40.8, 40.8, 40.7, 40.7, 40.7, 40.6, 40.5, 40.4, 39.9, 39.9, 39.4, 39.4, 39.3, 39.2, 37.4, 37.3, 37.3, 37.3, 37.2, 37.2, 37.1, 37.1, 37.1, 37.0, 37.0, 37.0, 36.9, 36.9, 36.9, 36.8, 36.8, 36.8, 36.7, 36.7, 36.7, 36.6, 36.6, 36.5, 36.5, 36.4, 36.4, 36.3, 36.3, 36.3, 36.2, 34.5, 34.5, 34.5, 34.2, 34.2, 34.2, 31.4, 31.3, 31.3, 31.3, 31.3, 31.2, 31.2, 31.1, 31.0, 31.0, 31.0, 30.7, 29.2, 29.2, 29.1, 29.1, 29.1, 29.1, 29.0, 28.6, 28.6, 28.6, 28.1, 28.1, 28.0, 28.0. **^{19}F NMR** (376 MHz, $CDCl_3$) δ −115.24 – −115.46 (m). **IR** ν (cm^{-1}): 2949, 2888, 2403, 2103, 1993, 1770, 1553, 1475. **HRMS** (APCI) *m/z*: Calculated for $C_{53}H_{63}F$ 718.4914. Found 719.4968 $[M + H]^{+}$.

Protodeborylated starting material (**PDB-9**) was also isolated from the reaction (4 mg, 8%) as a colourless film.

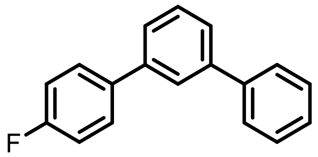

***R*f** (hexane): 0.30. **^{1}H NMR** (600 MHz, $CDCl_3$) δ 7.76 – 7.74 (m, 1H), 7.66 – 7.63 (m, 2H), 7.62 – 7.56 (m, 3H), 7.54 – 7.50 (m, 2H), 7.49 – 7.44 (m, 2H), 7.41 – 7.35 (m, 1H), 7.18 – 7.12 (m, 2H). **^{13}C NMR** (151 MHz, $CDCl_3$) δ 162.6 (d, $^{1}J_{C–F}$ = 246.9 Hz), 141.9, 141.1, 140.8, 137.3 (d, $^{4}J_{C–F}$ = 3.2 Hz), 129.3, 128.8, 128.8, 127.5, 127.3, 126.1 (d, $^{2}J_{C–F}$ = 25.9 Hz), 126.0. **^{19}F NMR** (565 MHz, $CDCl_3$) δ −115.58 (dt, *J* = 10.3, 5.2 Hz). **IR** ν (cm^{-1}): 2973, 2898, 2900, 2109, 1774, 1498, 1477. **HRMS** (EI) *m/z*: Calculated for $C_{18}H_{13}F$ 248.0996. Found 248.0989.

3.7) Bridged Biaryl Boronic Acid Pinacol Ester Tracks 10–15 as Substrates for Rh-Catalysed Norbornane Incorporation

2-(4-(4-Fluorobenzyl)phenyl)-4,4,5,5-tetramethyl-1,3,2-dioxaborolane (10)

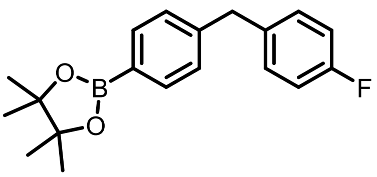

Track **2-(4-(4-fluorobenzyl)phenyl)-4,4,5,5-tetramethyl-1,3,2-dioxaborolane (10)** was subjected to General Procedure A and the crude reaction mixture analysed by ^{1}H NMR spectroscopy (Table S11).

Table S11 ^{1}H NMR analysis for **10** subjected to General Procedure A; yields determined using 1,3,5-trimethoxybenzene as an internal standard as described in Supplementary Information Section 3.2.

**entry**	**tri-10 (%, NMR yield)**	**di-10 (%, NMR yield)**	**tri-10 (%, isolated)**
1	74	6	
2	96	1	86 mg, 91%
3	96	1	

Entry 2 was purified by flash column chromatography on silica gel (hexane, isocratic), followed by further purification with preparative HPLC (hexane, isocratic), to afford **tri-10** (86 mg, 91%) as a colourless oil.

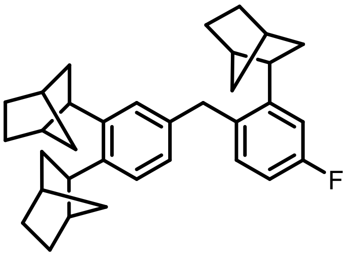

***R*f** (hexane): 0.30. **^{1}H NMR** (400 MHz, $CDCl_3$) δ 7.17 (dd, J = 8.0, 1.7 Hz, 1H), 7.03 (dt, J = 8.7, 2.2 Hz, 2H), 6.99 (d, J = 3.1 Hz, 1H), 6.85 (dd, J = 8.0, 1.9 Hz, 1H), 6.79 (td, J = 8.3, 2.8 Hz, 1H), 3.94 (ABq, Δν AB = 0.032, J_{AB} = 12.99, 2H), 2.99 – 2.89 (m, 2H), 2.89 – 2.82 (m, 1H), 2.39 – 2.23 (m, 6H), 1.86 – 1.72 (m, 3H), 1.70 – 1.47 (m, 12H), 1.44 – 1.17 (m, 9H). **^{13}C NMR** (101 MHz, $CDCl_3$) δ 161.7 (d, $^{1}J_{C\text{–}F}$ = 242.6 Hz), 148.2, 148.1, 145.3, 145.3, 143.1, 143.0, 137.4, 137.4, 134.6, 134.6, 131.6 (d, $^{3}J_{C\text{–}F}$ = 8.1 Hz), 126.3, 125.6, 125.6, 112.6 (d, $^{2}J_{C\text{–}F}$ = 21.9 Hz), 111.9 (d, $^{2}J_{C\text{–}F}$ = 20.8 Hz), 43.5, 43.5, 43.1, 43.0, 43.0, 43.0, 42.9, 42.8, 42.7, 42.7, 42.7, 42.3, 42.3, 40.2, 40.2, 40.2, 40.1, 40.1, 40.0, 39.4, 38.4,

38.3, 37.0, 37.0, 36.9, 36.9, 36.9, 36.7, 36.7, 36.6, 36.5, 31.2, 31.2, 31.1, 30.7, 29.1, 29.1, 29.0. **^{19}F NMR {^{1}H}** (377 MHz, $CDCl_3$): δ −116.88 (s). **IR** ν (cm^{-1}): 2948, 2867, 1587, 1489, 1475, 1453, 1417, 1352, 1310, 1297, 1270, 1235, 1215, 1170. **HRMS** (Negative ion, MALDI) *m/z*: Calculated for $C_{34}H_{41}F$ 468.3192. Found 467.3126 $[M - H]^-$.

Di-10 could be isolated from a separate reaction to provide analytically pure material for characterisation.

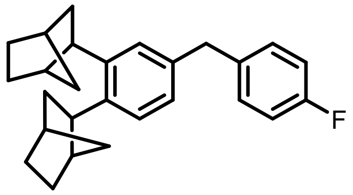

***R*f** (hexane): 0.30. **^{1}H NMR** (400 MHz, $CDCl_3$) δ 7.20 – 7.09 (m, 3H), 7.06 (t, J = 1.9 Hz, 1H), 7.00 – 6.93 (m, 2H), 6.90 (dd, J = 8.0, 2.0 Hz, 1H), 3.91 (s, 2H), 2.97 – 2.86 (m, 2H), 2.38 – 2.31 (m, 2H), 2.31 – 2.23 (m, 2H), 1.83 – 1.73 (m, 2H), 1.67 – 1.50 (m, 8H + H_2O (1H)), 1.41 – 1.16 (m, 6H). **^{13}C NMR** (101 MHz, $CDCl_3$) δ 161.5 (d, $^{1}J_{C\text{–}F}$ = 243.8 Hz), 145.5, 145.5, 143.3, 143.2, 137.6, 137.2, 137.2, 130.3 (d, $^{3}J_{C\text{–}F}$ = 7.7 Hz), 126.2, 125.8, 125.7, 125.6, 125.6, 115.2 (d, $^{2}J_{C\text{–}F}$ = 21.1 Hz), 43.1, 43.0, 43.0, 42.9, 42.9, 42.8, 42.7, 42.6, 41.0, 40.1, 40.1, 40.0, 39.9, 37.0, 36.9, 36.6, 36.5, 31.2, 31.2, 31.1, 31.1, 29.1. **^{19}F NMR {^{1}H}** (377 MHz, $CDCl_3$): δ −117.79 (s). **IR** ν (cm^{-1}): 2949, 2868, 1656, 1600, 1507, 1475, 1454, 1413, 1310, 1296, 1266, 1221. **HRMS** (Negative ion, MALDI) *m/z*: Calculated for $C_{27}H_{31}F$ 374.2410. Found 373.2331 $[M - H]^-$.

2-(4-((4'-Fluoro-[1,1'-biphenyl]-4-yl)methyl)phenyl)-4,4,5,5-tetramethyl-1,3,2-dioxaborolane (11)

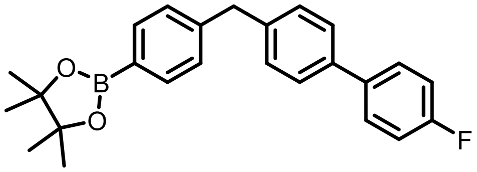

Track **2-(4-((4'-fluoro-[1,1'-biphenyl]-4-yl)methyl)phenyl)-4,4,5,5-tetramethyl-1,3,2-dioxaborolane (11)** was subjected to General Procedure A and the crude reaction mixture analysed by ^{1}H NMR spectroscopy (Table S12).

Table S12 ^{1}H NMR analysis for **11** subjected to General Procedure A; yields determined using 1,3,5-trimethoxybenzene as an internal standard as described in Supplementary Information Section 3.2.

entry	tetra-11 (%, NMR yield)	tri-11 (%, NMR yield)	tetra-11 (%, isolated)
1	79	4	86 mg, 67%
2	77	6	
3	76	3	

Entry 1 was purified by flash column chromatography on silica gel (hexane, isocratic), followed by further purification with preparative HPLC (hexane, isocratic), to afford **tetra-11** (86 mg, 67%) as a colourless oil.

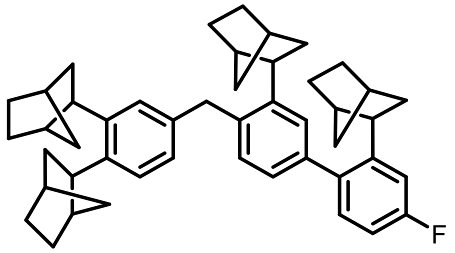

***R*f** (hexane): 0.30. **^{1}H NMR** (400 MHz, $CDCl_3$) δ 7.25 – 7.06 (m, 6H), 7.05 – 6.95 (m, 2H), 6.91 (td, *J* = 8.3, 2.7 Hz, 1H), 4.22 – 3.96 (m, 2H), 3.03 – 2.92 (m, 3H), 2.86 – 2.76 (m, 1H), 2.42 – 2.30 (m, 8H), 1.90 – 1.19 (m, 30H), 1.17 – 1.03 (m, 2H). **^{13}C NMR** (101 MHz, $CDCl_3$) δ 162.3 (d, $^{1}J_{C\text{–}F}$ = 244.2 Hz),, 148.1, 148.0, 145.3, 145.3, 143.0, 142.9, 139.5, 138.7, 138.6, 138.6, 137.6, 137.6, 137.6, 137.6, 131.6, 131.6, 131.5, 131.5, 130.1, 127.0, 126.9, 126.7, 126.6, 126.4, 126.4, 125.8, 125.6, 112.8, 112.8, 112.6, 112.5, 111.8 (d, $^{2}J_{C\text{–}F}$ = 20.9 Hz), 43.9, 43.4, 43.4, 43.3, 43.1, 43.1, 43.0, 43.0, 43.0, 42.9, 42.9, 42.8, 42.7, 42.7, 42.7, 42.6, 42.4, 40.5, 40.5, 40.3, 40.3, 40.2, 40.2, 40.1, 40.1, 40.1, 39.6, 39.3, 38.9, 38.8, 37.1, 37.0, 37.0, 37.0, 36.9, 36.8, 36.7, 36.7, 36.6, 36.6, 36.6, 31.2, 31.2, 31.2, 30.9, 30.7, 29.1, 29.1,

29.1, 28.6. **^{19}F NMR {^{1}H}** (377 MHz, $CDCl_3$): δ −115.31 (s), −115.32 (s. **IR** ν (cm^{-1}): 2949, 2868, 1606, 1583, 1474, 1453, 1418, 1351, 1310, 1297, 1268, 1243, 1215, 1154, 1139. **HRMS** (APCI) *m/z*: Calculated for $C_{47}H_{55}F$ 638.4288. Found 639.4359 $[M + H]^+$.

Tri-11 could be isolated (2 mg, 2%) as a colourless oil.

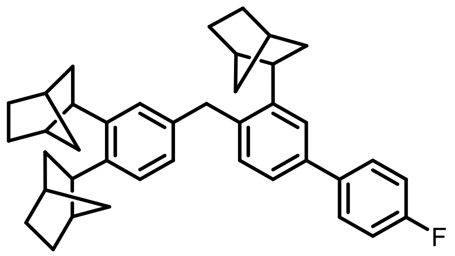

***R*f** (hexane): 0.30. **^{1}H NMR** (600 MHz, $CDCl_3$) δ 7.58 – 7.52 (m, 2H), 7.47 – 7.44 (m, 1H), 7.30 – 7.24 (m, 1H), 7.19 (dd, J = 8.0, 2.7 Hz, 1H), 7.16 – 7.10 (m, 4H), 6.93 – 6.88 (m, 1H), 4.13 – 3.91 (m, 2H), 2.98 – 2.90 (m, 3H), 2.44 – 2.24 (m, 6H), 1.85 – 1.77 (m, 3H), 1.76 – 0.79 (m, 21H + H_2O (16H)). **^{13}C NMR** (151 MHz, $CDCl_3$) δ 162.4 (d, J = 245.9 Hz), 146.1, 145.4, 143.1, 143.0, 138.5, 138.1, 138.0, 138.0, 137.4, 137.4, 130.9, 129.1, 128.8, 128.7, 128.6, 126.5, 126.2, 125.8, 125.8, 125.7, 124.3, 124.1, 115.5 (d, $^{2}J_{C–F}$ = 21.2 Hz), 43.5, 43.1, 43.1, 43.0, 43.0, 43.0, 42.9, 42.8, 42.8, 42.7, 42.7, 42.4, 42.4, 42.1, 40.6, 40.2, 40.2, 40.1, 40.1, 40.0, 39.4, 38.8, 38.8, 37.1, 37.0, 37.0, 36.9, 36.9, 36.8, 36.7, 36.7, 36.7, 36.7, 36.6, 36.6, 31.2, 31.2, 31.1, 30.8, 30.7, 29.9, 29.1, 29.1, 28.7.**^{19}F NMR {^{1}H}** (377 MHz, $CDCl_3$): δ −116.37 (s). **IR** ν (cm^{-1}): 2949, 2868, 1735, 1605, 1514, 1488, 1453, 1310, 1297, 1220, 1157, 1096. **HRMS** (APCI) *m/z*: Calculated for $C_{40}H_{44}F$ 544.3505. Found 545.3578 $[M + H]^+$.

2-(4'-(4-Fluorobenzyl)-[1,1'-biphenyl]-4-yl)-4,4,5,5-tetramethyl-1,3,2-dioxaborolane (12)

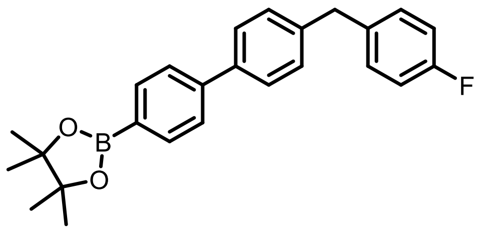

Track **2-(4'-(4-fluorobenzyl)-[1,1'-biphenyl]-4-yl)-4,4,5,5-tetramethyl-1,3,2-dioxaborolane (12)** was subjected to General Procedure A and the crude reaction mixture analysed by ^{1}H NMR spectroscopy (Table S13).

Table S13 ^{1}H NMR analysis for **12** subjected to General Procedure A; yields determined using 1,3,5-trimethoxybenzene as an internal standard as described in Supplementary Information Section 3.2.

entry	tetra-12 (%, NMR yield)	tri-12 (%, NMR yield)	tetra-12 (%, isolated)
1	69	21	
2	75	19	
3	76	14	77 mg, 60%

Entry 3 was purified by flash column chromatography on silica gel (hexane, isocratic), followed by further purification with preparative HPLC (hexane, isocratic), to afford **tetra-12** (77 mg, 60%) as a colourless oil.

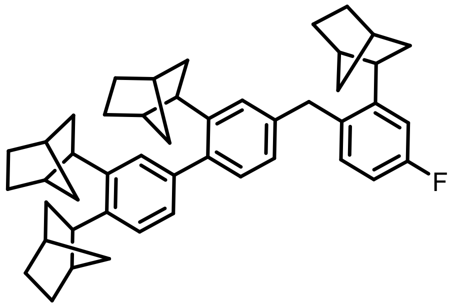

***R*f** (hexane): 0.30. **^{1}H NMR** (400 MHz, $CDCl_3$) δ 7.29 (dd, J = 8.0, 2.0 Hz, 1H), 7.21 – 7.15 (m, 2H), 7.14 – 7.00 (m, 4H), 6.94 – 6.90 (m, 1H), 6.84 (td, J = 8.3, 2.7 Hz, 1H), 4.01 (ABq, Δν AB = 0.034, J_{AB} = 13.49, 2H), 3.06 – 2.96 (m, 2H), 2.95 – 2.88 (m, 1H), 2.83 – 2.75 (m, 1H), 2.43 – 2.24 (m, 8H), 1.92 – 1.02 (m, 32H, + H_2O (2H)). **^{13}C NMR** (101 MHz, $CDCl_3$) δ 161.8 (d, $^{1}J_{C\text{–}F}$ = 242.9 Hz), 148.3, 148.2, 145.4, 144.8, 144.7, 144.7, 143.6, 143.5, 143.5, 140.6, 139.4, 139.2, 134.4, 134.4, 131.8, 131.7, 130.5, 130.4, 127.1, 127.0, 127.0, 126.5, 126.5, 126.4, 125.5, 125.1, 125.0, 125.0, 125.0, 112.7 (d, $^{2}J_{C\text{–}F}$ = 21.9 Hz), 112.0 (d, $^{2}J_{C\text{–}F}$ = 20.8 Hz), 43.8, 43.8, 43.7, 43.7, 43.6, 43.6, 43.6, 43.5, 43.5, 43.2, 43.1, 43.1, 43.0,

43.0, 42.9, 42.8, 42.7, 42.7, 42.4, 40.6, 40.6, 40.6, 40.4, 40.3, 40.3, 40.2, 40.2, 40.1, 40.0, 39.5, 38.6, 37.1, 37.1, 37.0, 37.0, 37.0, 36.9, 36.9, 36.8, 36.7, 36.7, 36.7, 36.6, 36.6, 31.3, 31.3, 31.2, 31.2, 31.2, 30.8, 30.7, 29.1, 29.1, 29.0, 28.7.**^{19}F NMR {^{1}H}** (377 MHz, $CDCl_3$): δ −116.62 (s). **IR** ν (cm^{-1}): 2950, 2869, 1609, 1587, 1487, 1474, 1453, 1417, 1354, 1311, 1297, 1270, 1215, 1171, 1139. **HRMS**(Negative ion, MALDI) *m/z*: Calculated for $C_{47}H_{55}F$ 638.4288. Found 637.4207 $[M - H]^-$.

Tri-12 could be isolated (3 mg, 3%, **tri-12** : **tetra-12** = 9 : 1) as a colourless oil.

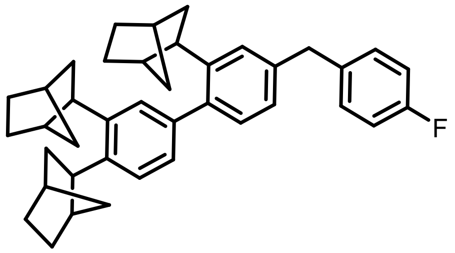

***R*f** (hexane): 0.30. **^{1}H NMR** (500 MHz, $CDCl_3$) δ 7.28 – 7.25 (m, 1H), 7.22 – 7.17 (m, 3H), 7.16 – 7.14 (m, 1H), 7.12 – 7.08 (m, 1H), 7.03 – 6.95 (m, 4H), 3.99 (s, 2H), 3.02 – 2.95 (m, 2H), 2.81 – 2.73 (m, 1H), 2.41 – 2.24 (m, 8H), 1.88 – 1.00 (m, 22H + H_2O (13H)). **^{13}C NMR** (126 MHz, $CDCl_3$) δ 161.6 (d, $^{1}J_{C\text{–}F}$ = 243.7 Hz), 145.6, 145.6, 144.8, 144.8, 144.7, 144.7, 143.6, 143.6, 143.6, 140.8, 140.8, 139.6, 139.2, 139.2, 137.0, 137.0, 131.8, 130.5, 130.5, 130.4, 129.3, 128.9, 127.4, 127.2, 127.1, 127.1, 127.0, 127.0, 126.5, 126.4, 126.4, 126.3, 126.3, 126.2, 126.2, 125.5, 125.1, 125.0, 125.0, 125.0, 115.3 (d, $^{2}J_{C\text{–}F}$ = 21.1 Hz). 43.7, 43.6, 43.6, 43.5, 43.2, 43.1, 43.1, 43.0, 43.0, 42.9, 42.8, 42.7, 42.7, 42.3, 41.3, 40.5, 40.5, 40.3, 40.3, 40.3, 40.2, 40.2, 40.1, 40.0, 39.5, 38.6, 37.1, 37.1, 37.0, 37.0, 36.9, 36.9, 36.8, 36.8, 36.8, 36.7, 36.7, 36.6, 36.6, 36.6, 31.3, 31.3, 31.2, 31.2, 31.2, 31.2, 30.7, 30.7, 30.4, 29.1, 29.1, 29.1, 29.0, 28.7. **^{19}F NMR {^{1}H}** (377 MHz, $CDCl_3$): δ −116.71 (s), −117.48 (s). **IR** ν (cm^{-1}): 2948, 2867, 1606, 1507, 1485, 1473, 1453, 1355, 1311, 1297, 1218, 1156, 1139, 1092, 1037. **HRMS** (Negative ion, MALDI) *m/z*: Calculated for $C_{40}H_{45}F$ 544.3505. Found 543.3432 $[M - H]^-$.

(4-Fluorophenyl)dimethyl(4-(4,4,5,5-tetramethyl-1,3,2-dioxaborolan-2-yl)phenyl)silane (13)

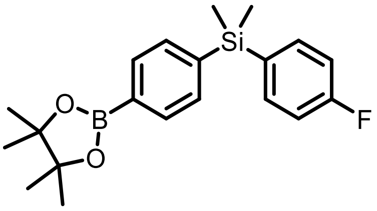

Track **(4-fluorophenyl)dimethyl(4-(4,4,5,5-tetramethyl-1,3,2-dioxaborolan-2-yl)phenyl)silane (13)** was subjected to General Procedure A and the crude reaction mixture analysed by ^{1}H NMR spectroscopy (Table S14).

Table S14 ^{1}H NMR analysis for **13** subjected to General Procedure A; yields determined using 1,3,5-trimethoxybenzene as an internal standard as described in Supplementary Information Section 3.2.

entry	tri-13 (%, NMR yield)	tri-13 (%, isolated)
1	94	
2	84	84 mg, 82%
3	85	

Entry 2 was purified by flash column chromatography on silica gel (hexane, isocratic), followed by further purification with preparative HPLC (hexane, isocratic), to afford **tri-13** (84 mg, 82%) as a colourless oil.

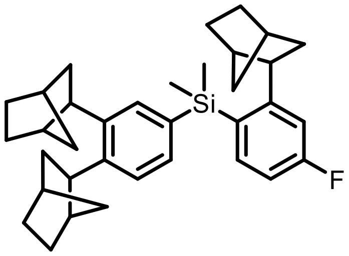

***R*f** (hexane): 0.45. **^{1}H NMR** (500 MHz, $CDCl_3$) δ 7.43 (dd, 1H, *J* = 8.3, 7.0 Hz, H-5), 7.34–7.33 (m, 1H), 7.23–7.19 (m, 2H), 6.99 (dd, *J* = 11.8, 2.5 Hz,1H), 6.85 (td, *J* = 8.4, 2.5 Hz, 1H), 2.97 – 2.89 (m, 2H), 2.75 – 2.66 (m, 1H), 2.36 – 2.20 (m, 5H), 1.99 – 1.94 (m, 1H), 1.83 – 1.48 (m, 9H + H_2O (5 H)), 1.47 – 1.19 (m, 11H), 1.19 – 1.12 (m, 2H), 1.02 – 0.93 (m, 1H), 0.85 – 0.77 (m, 1H), 0.55 (s, 6H). **^{13}C NMR** (126 MHz, $CDCl_3$) δ 164.6 (d, *J* = 246.9 Hz),, 157.7, 157.7, 146.4, 146.3, 146.3, 146.3, 144.5, 144.5, 137.2 (d, *J* = 7.8 Hz), 135.4, 135.3, 132.5, 132.5, 131.3, 131.3, 131.1, 131.1, 125.1, 125.1, 113.2 (d, *J* = 20.0 Hz), 111.8 (d, *J* = 19.3 Hz), 47.1, 47.1, 47.1, 44.6, 43.2, 43.2, 43.1, 43.1, 43.1, 43.0, 43.0, 43.0, 42.7, 42.7, 42.6, 41.6, 41.6, 41.6, 40.3, 40.2, 40.1, 40.1, 40.1, 40.1, 40.1, 37.1, 37.1, 37.1, 37.1,

37.0, 37.0, 36.9, 36.8, 36.7, 36.7, 36.7, 36.6, 36.6, 36.5, 36.5, 31.3, 31.3, 31.2, 31.2, 31.2, 30.9, 29.1, 29.1, 29.1, 29.1, 28.3, 28.3, 28.2, -0.2, -0.3, -0.3, -0.3, -0.3. **$^{19}F\{^{1}H\}$ NMR** ($CDCl_3$, 376 MHz) δ −111.8 (s), −111.8 (s), −111.8 (s). **IR** ν (cm^{-1}): 2949, 2912, 2868, 2108, 1932, 1605, 1575, 1457. **HRMS** (MALDI) *m/z*: Calculated for $C_{35}H_{45}FSi$ 512.3275. Found 535.3175 $[M+Na]^+$.

4-Fluoro-*N*-methyl-*N*-(4-(4,4,5,5-tetramethyl-1,3,2-dioxaborolan-2-yl)phenyl)aniline (14)

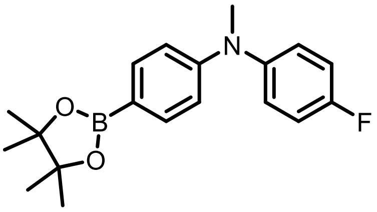

Track **4-fluoro-*N*-methyl-*N*-(4-(4,4,5,5-tetramethyl-1,3,2-dioxaborolan-2-yl)phenyl)aniline (14)** was subjected to General Procedure A and the crude reaction mixture analysed by ^{1}H NMR spectroscopy (Table S15).

Table S15 ^{1}H NMR analysis for **14** subjected to General Procedure A; yields determined using 1,3,5-trimethoxybenzene as an internal standard as described in Supplementary Information Section 3.2.

entry	tri-14 (%, NMR yield)	tri-14 (%, isolated)
1	81	
2	104	87 mg, 90%
3	98	
4	106	

Entry 2 was purified by flash column chromatography on silica gel (hexane:DCM, 95:5) to afford **tri-14** (87 mg, 90%) as a colourless oil.

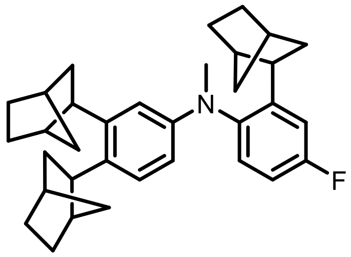

***R*f** (hexane:DCM, 95:5): 0.30. **^{1}H NMR** (400 MHz, CDCl$_3$): δ 7.18 – 6.99 (m, 3H), 6.95 – 6.84 (m, 1H), 6.44 – 6.33 (m, 2H), 3.18 (s, 3H), 2.99 – 2.79 (m, 3H), 2.41 – 2.08 (m, 6H), 1.83 – 1.09 (m, 24H + H_2O (2H)). **^{13}C NMR** (101 MHz, CDCl$_3$): δ 161.3 (d, $^1J_{C\text{–}F}$ = 244.0 Hz), 149.5, 149.4, 147.6, 147.6, 147.5, 145.8, 145.8, 145.7, 143.4, 143.3, 134.0, 134.0, 133.9, 133.9, 130.3, 130.2, 126.0, 126.0, 126.0, 113.8, 113.6, 111.4, 111.4, 111.4, 109.9, 109.8, 109.8, 43.3, 43.3, 43.2, 43.2, 43.2, 43.1, 43.0, 42.9, 42.8, 42.5, 42.4, 42.4, 42.4, 41.8, 40.5, 40.4, 40.2, 40.2, 40.1, 40.1, 40.0, 37.0, 36.9, 36.9, 36.9, 36.8, 36.8, 36.7, 36.6, 36.6, 36.5, 36.5, 36.4, 31.2, 31.2, 31.2, 31.1, 30.9, 29.9, 29.2, 29.2, 29.1, 29.0, 28.5. **^{19}F NMR {^{1}H}** (377 MHz, CDCl$_3$): δ −115.08 (s), −115.09 (s), −115.09 (s), −115.11 (s). **IR** ν (cm^{-1}): 2950, 2868, 1609, 1491, 1219. **HRMS** (APCI) *m/z*: Calculated for $C_{34}H_{42}FN$ 483.3301. Found 484.3374 [M + H]$^+$.

2-(4-(4-Fluorophenoxy)phenyl)-4,4,5,5-tetramethyl-1,3,2-dioxaborolane (15)

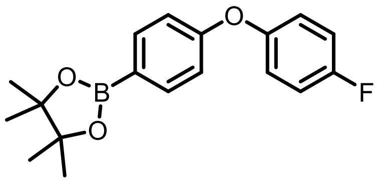

Track **2-(4-(4-fluorophenoxy)phenyl)-4,4,5,5-tetramethyl-1,3,2-dioxaborolane (15)** was subjected to General Procedure A and the crude reaction mixture analysed by ^{1}H NMR spectroscopy (Table S16).

Table S16 ^{1}H NMR analysis for **15** subjected to General Procedure A; yields determined using 1,3,5-trimethoxybenzene as an internal standard as described in Supplementary Information Section 3.2.

entry	tri-15 (%, NMR yield)	di-15 (%, NMR yield)	tri-15 (%, isolated)
1	76	15	
2	52	37	45 mg, 48%
3	52	47	
4	42	49	

Entry 2 was purified by flash column chromatography on silica gel (hexane, isocratic), followed by further purification with preparative HPLC (hexane, isocratic), to afford **tri-15** (45 mg, 48%) and **di-15** (26 mg, 35%) as colourless oils.

Tri-15

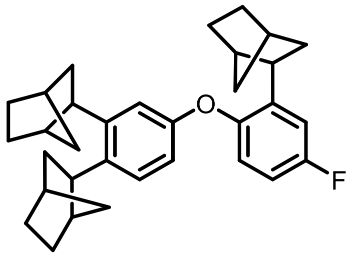

***R*f** (hexane): 0.30. **^{1}H NMR** (500 MHz, $CDCl_3$) δ 7.14 (dd, 1H, J = 8.6, 2.2 Hz), 7.01 (dd, 1H, J = 10.1, 2.5 Hz), 6.88 – 6.87 (m, 1H), 6.81 – 6.76 (m, 2H), 6.58 (dd, 1H, J = 8.6, 2.7 Hz), 2.96 – 2.86 (m, 3H), 2.36 – 2.24 (m, 6H), 1.82 – 1.74 (m, 3H), 1.65 – 1.48 (m, 11H + H_2O (5H)), 1.39 – 1.18 (m, 10H). **^{13}C NMR** (126 MHz, $CDCl_3$, polynomial fit method (polynomial order 15) applied) δ 158.9 (d, $^{1}J_{C–F}$ = 240.4 Hz), 155.8, 155.7, 150.7, 150.7, 147.3, 147.3, 147.3, 141.2, 141.1, 139.7, 139.7, 139.7, 139.7, 126.6, 126.6, 120.3 (d, $^{3}J_{C–F}$ = 8.6 Hz), 115.6, 115.6, 113.9, 113.9, 113.9, 113.9, 113.5 (d, $^{2}J_{C–F}$ = 23.7 Hz), 112.6 (d, $^{2}J_{C–F}$ = 23.1 Hz), 43.2, 43.2, 43.1, 43.1, 42.9, 42.9, 42.8, 42.8, 42.7, 42.6, 42.6, 41.6, 40.9, 40.8, 40.3, 40.3, 40.2, 40.2, 40.1, 40.0, 40.0, 39.1, 37.0, 37.0, 36.9, 36.9, 36.7, 36.6, 36.5, 36.5, 31.7,

31.2, 31.1, 31.1, 30.5, 29.1, 29.0, 28.9. **^{19}F{^{1}H} NMR** ($CDCl_3$, 376 MHz) δ −111.67–−111.75 (m). **IR** ν (cm^{-1}): 2948, 2864, 1894, 1590, 1557, 1498, 1338, 1303. **HRMS** (Negative ion, MALDI) *m/z*: Calculated for $C_{33}H_{39}FO$ 470.2985. Found 469.2916 $[M-H]^-$.

Di-15

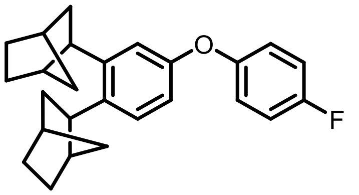

***R*f** (hexane): 0.20. **^{1}H NMR** ($CDCl_3$, 500 MHz) δ 7.18 (dd, 1H, J = 8.4, 2.4 Hz), 7.02 – 6.92 (m, 5H), 6.68 (dd, 1H, J = 8.4, 2.7 Hz), 2.95 – 2.87 (m, 2H), 2.36 – 2.33 (m, 2H), 2.28 – 2.26 (m, 2H), 1.83 – 1.77 (m, 2H), 1.65 – 1.49 (m, 7H + H_2O (2H)), 1.39 – 1.18 (m, 7H). **^{13}C NMR** ($CDCl_3$, 126 MHz) δ 158.7 (d, $^{1}J_{C\text{–}F}$ = 240.7 Hz), 155.1, 155.0, 153.6, 147.5, 147.5, 140.6, 140.5, 126.8, 126.8, 120.0, 120.0 (d, $^{3}J_{C\text{–}F}$ = 8.2 Hz), 116.5, 116.4, 116.3 (d, $^{2}J_{C\text{–}F}$ = 23.2 Hz), 114.9, 114.9, 43.3, 43.2, 43.1, 43.0, 42.8, 42.8, 42.7, 42.7, 40.3, 40.2, 40.1, 37.1, 37.0, 37.0, 36.9, 36.7, 36.7, 36.6, 36.6, 31.2, 31.2, 31.1, 29.1, 29.0. **^{19}F NMR** ($CDCl_3$, 376 MHz) δ −121.0 (tt, J = 8.2, 4.6 Hz), −121.0 (tt, J = 8.2, 4.6 Hz). **IR** ν (cm^{-1}): 3029, 2946, 2866, 1893, 1557, 1468, 1454, 1310. **HRMS** (EI) *m/z*: Calculated for $C_{26}H_{29}FO$ 376.2202. Found 376.2189 $[M]^+$.

3.8) Mechanistic Studies to Investigate Directionally Sequential Processive Incorporation of Norbornane Units

3.8.1) Deuterium oxide quenching experiment

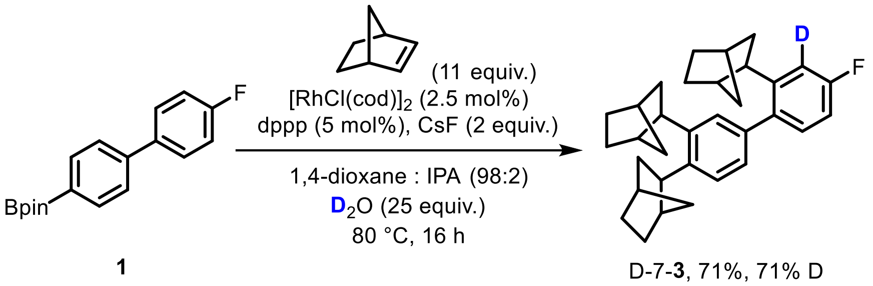

An over-dried Schlenk tube (15 mm diameter) was charged with CsF (61 mg, 0.40 mmol, 2.0 eq) and placed under high vacuum and heated at 100 °C with stirring for 2 h to remove any residual H_2O. After cooling to room temperature, the reaction vessel was further charged with track **2-(4'-fluoro-[1,1'-biphenyl]-4-yl)-4,4,5,5-tetramethyl-1,3,2-dioxaborolane (1)** (0.20 mmol, 1.0 eq), dppp (5.0 mg, 5.0 mol%), $[RhCl(cod)]_2$ (2.5 mg, 2.5 mol%) and norbornene (207 mg, 2.2 mmol, 11 eq). D_2O (100 μL) was then added, followed by IPA (10 μL) (not anhydrous), and 1,4-dioxane (1.0 mL). The reaction mixture was heated to 80 °C and stirred at that temperature for 16 hours. After this time, the reaction mixture was cooled to room temperature and the reaction mixture was filtered through Celite®, eluting with ethyl acetate. The filtrate was then concentrated under reduced pressure and a known quantity of 1,3,5-trimethoxybenzene as an internal standard was added to the residue before analysis of the crude reaction mixture by ^{1}H NMR spectroscopy (Table S17).

Table S17 ^{1}H NMR analysis for **1** subjected to deuterium oxide quenching experiment; yields determined using 1,3,5-trimethoxybenzene as an internal standard as described in Supplementary Information Section 3.2.

entry	D-7-tri-1 (%, NMR yield)	D-di-1 (%, NMR yield)	D-7-tri-1 (%, isolated)
1	99	0	
2	100	1	
3	99	4	65 mg, 71%

Entry 3 was purified by flash column chromatography on silica gel (hexane, isocratic), followed by purification by preparative HPLC (hexane, isocratic), to afford **D-7-tri-1** (65 mg, 71%, 71% D incorporation by ^{1}H NMR spectroscopic analysis) as a colourless oil.

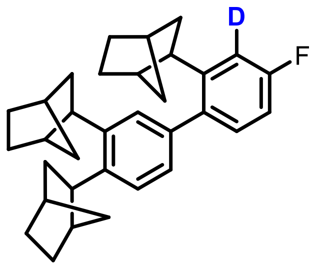

***R*f** (hexane): 0.30. **^{1}H NMR** (400 MHz, $CDCl_3$) δ 7.29 (dd, J = 8.0, 2.0 Hz, 1H), 7.19 – 7.11 (m, 2H), 7.08 (dd, J = 11.2, 2.7 Hz, 0.29H), 7.01 (dd, J = 8.0, 1.9 Hz, 1H), 6.88 (t, J = 8.3 Hz, 1H), 3.07 – 2.95 (m, 2H), 2.81 – 2.71 (m, 1H), 2.43 – 2.26 (m, 6H), 1.92 – 1.56 (m, 10H + H_2O (2H)), 1.55 – 1.27 (m, 12H), 1.26 – 1.17 (m, 2H), 1.14 – 1.03 (m, 2H). **^{13}C NMR** (101 MHz, $CDCl_3$) δ 162.2 (d, $^1J_{C–F}$ = 244.2 Hz), 148.0, 148.0, 144.9, 144.9, 144.8, 143.8, 143.8, 143.8, 138.6, 138.4, 138.4, 131.6, 131.5, 131.5, 131.5, 127.1, 127.0, 127.0, 126.5, 126.4, 125.1, 125.1, 125.1, 125.1, 112.7 (d, $^2J_{C–F}$ = 22.2 Hz), 111.7 (d, $^2J_{C–F}$ = 20.9 Hz), 43.8, 43.6, 43.5, 43.4, 43.4, 43.2, 43.1, 43.1, 43.0, 43.0, 43.0, 42.9, 42.7, 42.6, 40.5, 40.5, 40.3, 40.3, 40.3, 40.2, 40.1, 40.1, 40.0, 37.1, 37.0, 37.0, 37.0, 36.9, 36.8, 36.8, 36.8, 36.7, 36.7, 36.7, 36.6, 36.6, 31.2, 31.2, 31.1, 30.6, 29.1, 29.0, 28.6. **^{19}F NMR {^{1}H}** (377 MHz, $CDCl_3$) δ −115.47, −115.47, −115.48, −115.50, −115.77, −115.77, −115.78, −115.80. **IR** ν (cm^{-1}): 2951, 2874, 1602, 1538, 1405, 1219, 1147. **HRMS** (EI) *m/z*: Calculated for $C_{33}H_{38}DF$ 455.3099. Found 455.3079 $[M]^+$. Quantification using QExactive Accurate Mass EI-GC-MS gave %D_1 = 73.96% and %D_0 = 26.02%.

3.8.2) Deuterated isopropyl alcohol quenching experiment

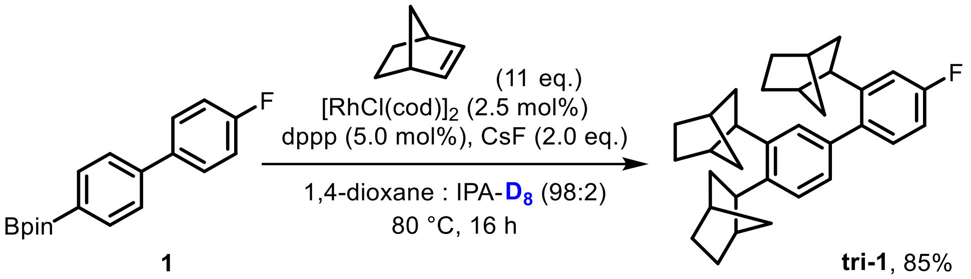

1 **tri-1**, 85%

Under a nitrogen atmosphere an over-dried Schlenk tube (15 mm diameter) was charged with **2-(4'-fluoro-[1,1'-biphenyl]-4-yl)-4,4,5,5-tetramethyl-1,3,2-dioxaborolane (1)** (0.20 mmol, 1.0 eq.), CsF (61 mg, 0.40 mmol, 2.0 eq.), dppp (5.0 mg, 5.0 mol%), $[RhCl(cod)]_2$ (2.5 mg, 2.5 mol%) and norbornene (207 mg, 2.2 mmol, 11 eq.), followed by the addition d_8-IPA (10 µL) and 1,4-dioxane (1.0 mL). The reaction mixture was heated to 80 °C and stirred at that temperature for 16 hours. After this time, the reaction mixture was cooled to room temperature and the reaction mixture was filtered through Celite®, eluting with ethyl acetate. The filtrate was then concentrated under reduced pressure and a known quantity of 1,3,5-trimethoxybenzene as an internal standard was added to the residue before analysis of the crude reaction mixture by ^{1}H NMR spectroscopy (Table S18).

Table S18 ^{1}H NMR analysis for **1** subjected to d_8-IPA (quenching experiment; yields determined using 1,3,5-trimethoxybenzene as an internal standard as described in Supplementary Information Section 3.2.

entry	tri-1 (%, NMR yield)	di-1 (%, NMR yield)	tri-1 (%, isolated)
1	85	10	77 mg, 85%
2	70	11	-

Entry 1 was purified by flash column chromatography on silica gel (hexane, isocratic), followed by purification by preparative HPLC (hexane, isocratic), to afford **tri-1** (77 mg, 85%, 0% D incorporation by ^{1}H NMR spectroscopic analysis) as a colourless oil.

The absence of deuterium incorporation observed in the presence of d8-IPA in contrast to the high levels of deuterium incorporation that arise when the reaction is conducted in the presence of 25 equivalents of D_2O (S3.8.1) lead us to conclude that the key terminating protoderhodation events arise from adventitious H_2O rather than the IPA co-solvent

3.8.3) Study of increasing equivalents of H_2O on optimised reaction conditions for track 1

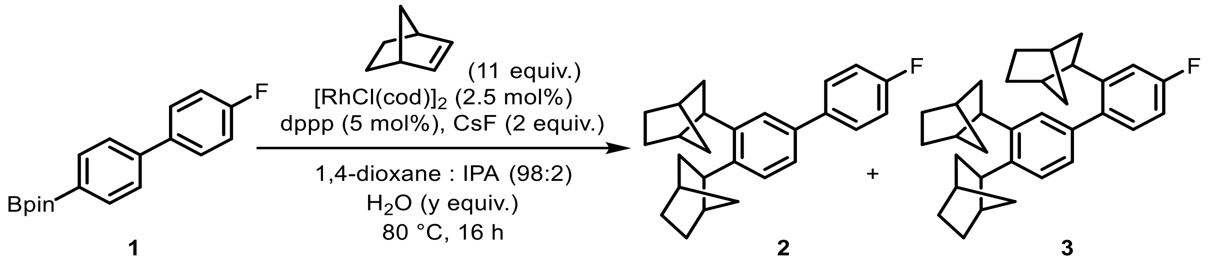

An over-dried Schlenk tube (15 mm diameter) was charged with CsF (61 mg, 0.40 mmol, 2.0 eq) and placed under high vacuum and heated at 100 °C with stirring for 2 h to remove any residual H_2O. After cooling to room temperature, the reaction vessel was further charged with track **2-(4'-fluoro-[1,1'-biphenyl]-4-yl)-4,4,5,5-tetramethyl-1,3,2-dioxaborolane (1)** (0.20 mmol, 1.0 eq), dppp (5.0 mg, 5.0 mol%), $[RhCl(cod)]_2$ (2 .5 mg, 2.5 mol%) and norbornene (207 mg, 2.2 mmol, 11 eq). H_2O (50 µL or 100 µL or 200 µL) was then added, followed by IPA (10 µL) (not anhydrous), and 1,4-dioxane (1.0 mL). The reaction mixture was heated to 80 °C and stirred at that temperature for 16 hours. After this time, the reaction mixture was cooled to room temperature and the reaction mixture was filtered through Celite®, eluting with ethyl acetate. The filtrate was then concentrated under reduced pressure and a known quantity of 1,3,5-trimethoxybenzene as an internal standard was added to the residue before analysis of the crude reaction mixture by ^{1}H NMR spectroscopy (Table S19).

Table S19 ^{1}H NMR analysis for study of increasing equivalents of H_2O on optimised reaction conditions for track **1**; yields determined using 1,3,5-trimethoxybenzene as an internal standard as described in Supplementary Information Section 3.2.

**entry**	**H_2O (µL, eq)**	**tri-1 (%, NMR yield)**	**di-1 (%, NMR yield)**	**di-1 : tri-1**
1	0 µL (0 eq)	96	0	-
2	0 µL (0 eq)	89	1	-
3	0 µL (0 eq)	85	1	-
**average (1–3)**	**0 µL (0 eq)**	**90**	**1**	**1.0 : 90**
4	50 µL (14 eq)	73	9	-
5	50 µL (14 eq)	71	8	-
6	50 µL (14 eq)	85	11	-
**average (4–6)**	**50 µL (14 eq)**	**76**	**9**	**1.0 : 8.4**
7	100 µL (28 eq)	71	24	-
8	100 µL (28 eq)	63	20	-
9	100 µL (28 eq)	67	21	-
**average (7–9)**	**100 µL (28 eq)**	**67**	**22**	**1.0 : 3.0**
10	200 µL (56 eq)	60	36	-

11	200 µL (56 eq)	65	32	-
12	200 µL (56 eq)	54	32	-
**average (10–12)**	**200 µL (56 eq)**	**60**	**33**	**1.0 : 1.8**

3.8.4) Di-1 subjected to optimised reaction conditions

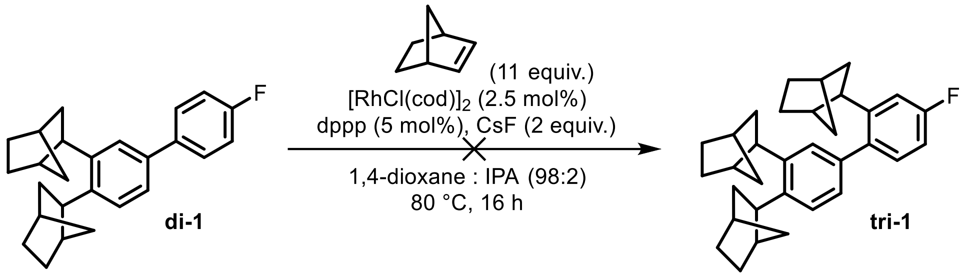

Under a nitrogen atmosphere an over-dried Schlenk tube (15 mm diameter) was charged with di-substituted track **di-1** (10 mg, 0.03 mmol, 1.0 eq), CsF (8 mg, 0.05 mmol, 2.0 eq), dppp (0.6 mg, 5.0 mol%), $[RhCl(cod)]_2$ (0.3 mg, 3 mol%) and norbornene (30 mg, 0.3 mmol, 11 eq), followed by the addition of (not anhydrous) IPA (10 µL) and1,4-dioxane (1.0 mL). The reaction mixture was heated to 80 °C and stirred at that temperature for16 hours. After this time, the reaction mixture was cooled to room temperature and the reaction mixture was filtered through Celite®, eluting with ethyl acetate. The filtrate was then concentrated under reduced pressure and a known quantity of 1,3,5-trimethoxybenzene as an internal standard was added to the residue before analysis of the crude reaction mixture by ^{1}H NMR spectroscopy, which confirmed that no tri-substituted track **tri-1** had been formed.

The crude reaction mixture was purified by flash column chromatography on silica gel (hexane, isocratic) to afford **di-1** (10 mg, 100%) as a colourless oil.

3.8.5) Bis-deutero track D_2-1 subjected to optimised reaction conditions

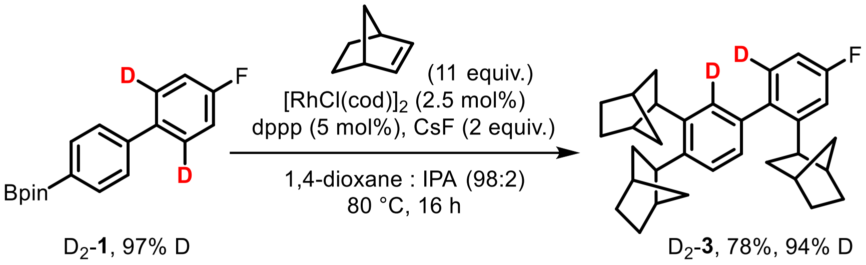

Track **2-(4'-fluoro-[1,1'-biphenyl]-4-yl-2',6'-*d₂*)-4,4,5,5-tetramethyl-1,3,2-dioxaborolane (D_2-1)** was subjected to General Procedure A and the crude reaction was purified by flash column chromatography on silica gel (hexane, isocratic), followed by further purification with preparative HPLC (hexane, isocratic), to afford D_2-**tri-1** (71 mg, 78%, 94% D incorporation by ^{1}H NMR spectroscopic analysis) as a colourless oil.

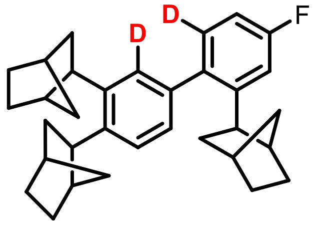

***R*f** (hexane): 0.30. **^{1}H NMR** (400 MHz, $CDCl_3$) δ 7.29 (dd, *J* = 8.0, 2.0 Hz, 1H), 7.14 (m, 0.12H), 7.09 (dd, *J* = 11.1, 2.7 Hz, 1H), 7.01 (d, *J* = 7.9 Hz, 1H), 6.89 (dd, *J* = 8.2, 2.7 Hz, 1H), 3.01 (m, 2H), 2.77 (m, 1H), 2.44 – 2.26 (m, 6H), 1.85 (m, 2H), 1.78 – 1.56 (m, 9H), 1.56 – 1.18 (m, 11H + H_2O (1H)), 1.14 – 1.01 (m, 2H). **^{13}C NMR** (101 MHz, $CDCl_3$) δ 162.2 (d, $^1J_{C\text{–}F}$ = 244.1 Hz), 148.1, 148.0, 144.8, 144.8, 144.8, 143.8, 143.8, 143.8, 138.5, 138.3, 131.2 (weak m), 126.5 (weak m), 126.5, 125.2, 125.1, 125.1, 125.1, 125.1, 112.7 (d, $^2J_{C\text{–}F}$ = 21.5 Hz), 111.6 (d, $^2J_{C\text{–}F}$ = 20.8 Hz), 43.8, 43.6, 43.5, 43.5, 43.4, 43.2, 43.2, 43.1, 43.1, 43.1, 43.0, 43.0, 43.0, 42.9, 42.8, 42.7, 40.5, 40.5, 40.4, 40.3, 40.3, 40.2, 40.2, 40.1, 40.0, 37.1, 37.1, 37.0, 37.0, 36.9, 36.9, 36.8, 36.8, 36.8, 36.7, 36.7, 36.7, 36.6, 31.3, 31.2, 31.2, 31.2, 30.6, 29.1, 29.1, 29.1, 28.6.. **^{19}F NMR {^{1}H}** (376 MHz, $CDCl_3$) δ −115.50, −115.50, −115.51, −115.53. **IR** ν (cm^{-1}): 2950, 2868, 1600, 1581, 1453, 1352, 1297, 1234, 1214. **HRMS** (EI) *m/z*: Calculated for $C_{33}H_{37}D_2F$ 456.3161. Found 456.3162 $[M]^+$. Quantification using QExactive Accurate Mass EI-GC-MS gave %D_2 = 93.30%, %D_1 = 5.56%, and D_0 = 1.14%.

3.9) Proposed Mechanism for Directional Motion of the Rhodium Centre

Based on the experiments detailed in Section 3.8 and the relevant literature we propose the mechanism outlined in Figure S2 to explain the directional motion of the rhodium centre across the representative biaryl track, **1**.

Figure S2 Proposed mechanism for directional motion of Rh centre along biaryl track **1**, including representative rhodacycles for the key C(sp^3)-to-C(sp^2) and C(sp^2)-to-C(sp^2) Rh migrations.

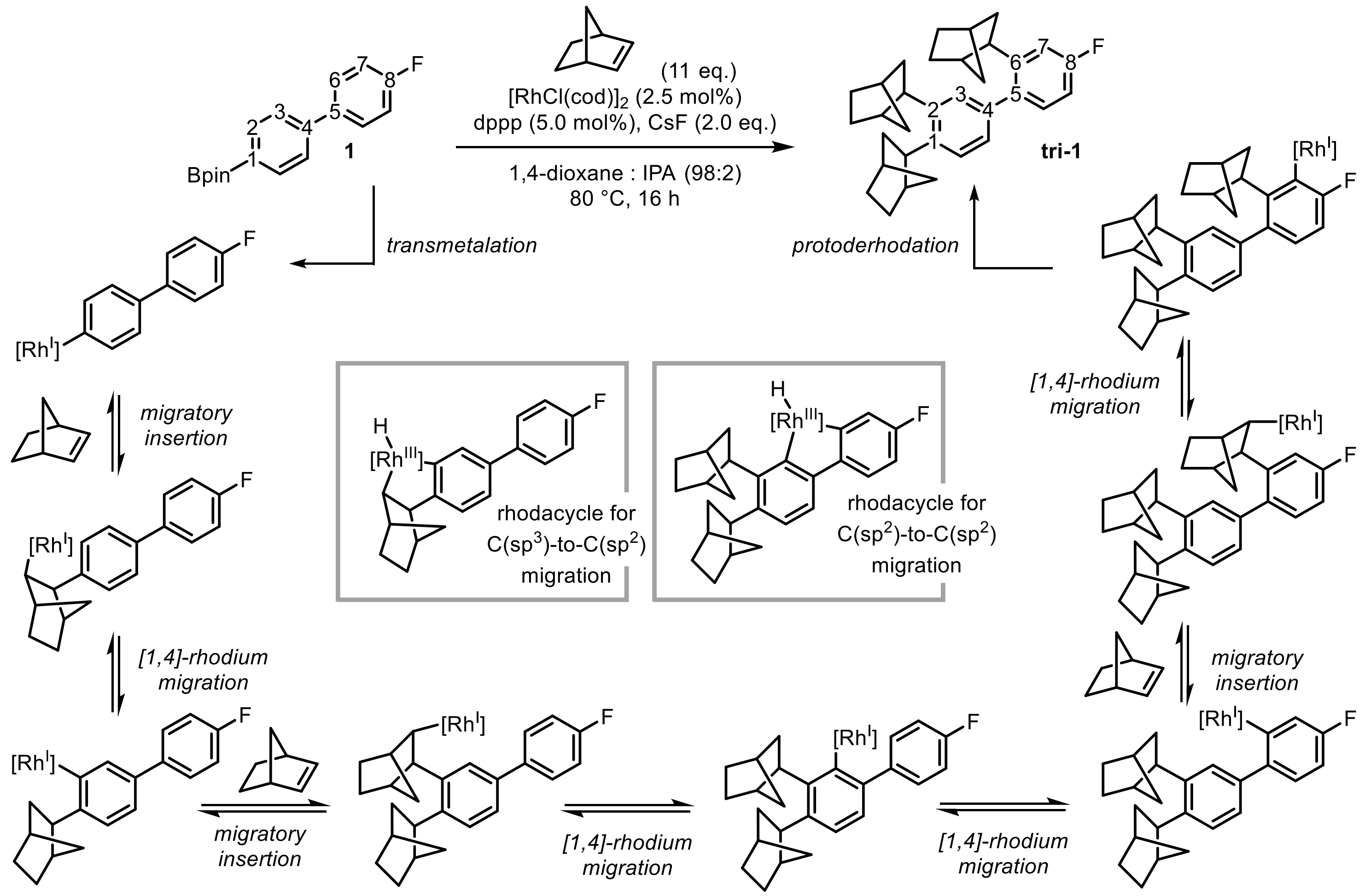

3.10) Polyaniline Boronic Acid Pinacol Ester Tracks 16 and 17 as Substrates for Rh-Catalysed Norbornane Incorporation

3.10.1) *N*^{1},*N*^{4}-dimethyl-*N*^{1}-phenyl-*N*^{4}-(4-(4,4,5,5-tetramethyl-1,3,2-dioxaborolan-2-yl)phenyl)benzene-1,4-diamine (16)

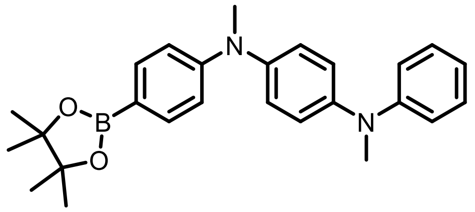

Track ***N*^{1},*N*^{4}-dimethyl-*N*^{1}-phenyl-*N*^{4}-(4-(4,4,5,5-tetramethyl-1,3,2-dioxaborolan-2-yl)phenyl)benzene-1,4-diamine (16)** was subjected to General Procedure C and the crude reaction mixture was purified by flash column chromatography on silica gel (hexane, isocratic) to afford a mixture of **hexa-16** and **tetra-16** (132 mg, 80% combined yield, **tetra-16 : hexa-16** = 1.0 : 6.7).

Further purification with preparative HPLC (hexane, isocratic), allowed the isolation of an analytically pure sample of **hexa-16** (58 mg, 34%) and **tetra-16** (10 mg, 8%, **tetra-16** : **hexa-16** = 5 : 1) as colourless oils.

Hexa-16

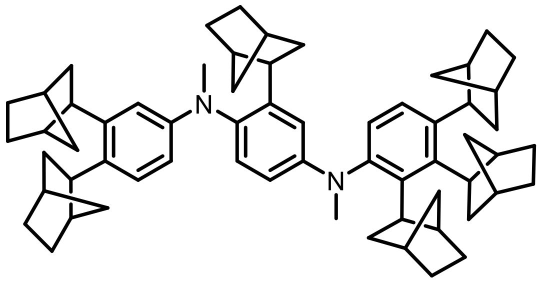

***R*$_{f}$** (hexane): 0.20. **^{1}H NMR** (600 MHz, $CDCl_3$) δ 7.32 – 7.19 (m, 1H), 7.16 – 7.10 (m, 1H), 7.00 – 6.83 (m, 2H), 6.75 – 6.40 (m, 4H), 3.55 – 2.89 (m, 12H), 2.65 – 0.85 (m, 60H + H_2O (3H)). **^{13}C NMR** (151 MHz, $CDCl_3$) δ 151.1, 151.1, 151.0, 150.9, 150.9, 150.3, 150.3, 150.3, 150.2, 150.2, 148.8, 148.8, 148.8, 148.0, 148.0, 147.9, 147.9, 147.9, 147.8, 147.8, 147.7, 147.7, 147.6, 147.6, 146.8, 146.8, 146.8, 146.7, 146.6, 146.3, 146.2, 146.2, 146.1, 146.0, 146.0, 145.6, 145.6, 145.5, 145.5, 145.5, 145.4, 145.4, 145.4, 145.4, 145.3, 145.3, 145.2, 145.2, 145.2, 145.1, 145.0, 145.0, 144.9, 144.9, 144.9, 144.8, 144.8, 140.5, 137.8, 137.7, 137.7, 137.7, 137.7, 137.6, 133.4, 133.3, 133.2, 133.2, 129.5, 129.2, 129.1, 126.5, 126.4, 126.4, 126.4, 126.3, 126.3, 126.3, 126.2, 126.2, 126.1, 113.2, 112.8, 111.7, 111.6, 111.5, 111.4, 110.1, 110.0, 45.7, 45.7, 45.7, 45.4, 45.3, 45.2, 45.0, 45.0, 44.9, 44.8, 44.8, 44.6, 44.6, 44.6, 44.5, 44.5,

44.4, 44.1, 44.0, 44.0, 44.0, 43.9, 43.9, 43.9, 43.8, 43.8, 43.8, 43.7, 43.7, 43.6, 43.6, 43.6, 43.6, 43.5, 43.5, 43.5, 43.4, 43.4, 43.3, 43.3, 43.2, 43.2, 43.2, 43.2, 43.1, 43.1, 43.1, 43.1, 43.0, 42.9, 42.9, 42.9, 42.8, 42.8, 42.8, 42.7, 42.7, 42.6, 42.6, 42.5, 42.5, 42.4, 42.4, 42.4, 42.3, 42.3, 42.3, 42.3, 42.2, 42.2, 42.1, 42.1, 42.1, 42.0, 41.9, 41.9, 41.8, 41.7, 41.7, 41.7, 41.7, 41.6, 41.6, 41.5, 41.5, 41.4, 41.4, 41.3, 41.2, 41.0, 41.0, 40.9, 40.9, 40.7, 40.6, 40.5, 40.4, 40.4, 40.3, 40.3, 40.3, 40.2, 40.1, 40.0, 40.0, 39.7, 39.6, 39.6, 39.5, 39.5, 39.5, 39.5, 39.4, 39.4, 39.4, 39.3, 39.3, 39.3, 39.2, 39.2, 39.2, 39.2, 37.6, 37.6, 37.6, 37.5, 37.5, 37.5, 37.4, 37.4, 37.4, 37.4, 37.3, 37.3, 37.2, 37.2, 37.1, 37.1, 37.0, 37.0, 37.0, 36.9, 36.9, 36.9, 36.8, 36.8, 36.8, 36.7, 36.7, 36.7, 36.6, 36.6, 35.4, 35.3, 35.3, 35.2, 35.1, 35.1, 33.7, 33.7, 33.5, 33.4, 33.3, 33.1, 33.1, 32.9, 32.8, 32.8, 32.4, 31.5, 31.5, 31.5, 31.4, 31.4, 31.3, 31.2, 31.2, 31.2, 31.2, 31.1, 30.2, 29.5, 29.5, 29.4, 29.4, 29.3, 29.3, 28.9, 28.9, 28.8, 28.8, 28.5, 28.5, 28.4, 28.3, 28.3, 28.2, 28.2, 28.1, 27.9, 27.8. **IR** ν (cm^{-1}): 2987, 2900, 1938, 2087, 1736, 1447, 1356, 1257. **HRMS** (MALDI) *m/z*: Calculated for $C_{62}H_{80}N_2$ 852.6316. Found 852.6312 $[M]^+$.

Tetra-16

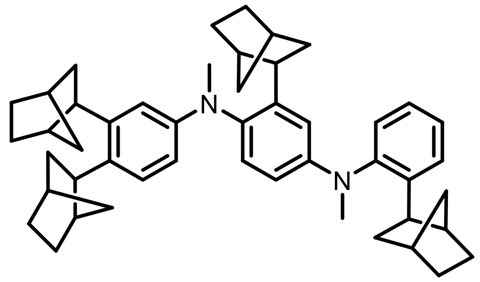

R*f** (hexane): 0.20. **^{1}H NMR** (600 MHz, $CDCl_3$) *Note: over-integration of aliphatic region corresponds to presence of 20% **hexa-16 δ 7.33 (d, *J* = 9.5 Hz, 2H), 7.28 – 7.22 (m, 2H), 7.22 – 7.17 (m, 1H), 6.78 (t, *J* = 7.2 Hz, 1H), 6.74 – 6.64 (m, 3H), 6.65 – 6.60 (m, 1H), 3.16 (s, 3H), 3.09 (s, 3H), 3.08 – 3.01 (m, 1H + **hexa-16** (1H)), 3.01 – 2.91 (m, 2H + **hexa-16** (1H)), 2.91 – 2.85 (m, 1H), 2.50 – 0.82 (m, 40H + **hexa-16** (13H) + H_2O (4H)). **^{13}C NMR** (151 MHz, $CDCl_3$) δ 150.3, 148.1, 148.1, 146.2, 146.2, 146.2, 145.9, 145.8, 145.8, 145.8, 145.3, 134.1, 134.0, 133.9, 129.4, 126.6, 126.6, 117.4, 113.2, 112.1, 112.1, 110.0, 109.9, 43.7, 43.7, 43.6, 43.6, 43.6, 43.5, 43.5, 43.4, 43.4, 43.3, 43.3, 43.3, 43.2, 42.9, 42.9, 42.8, 42.8, 42.1, 42.1, 40.9, 40.9, 40.7, 40.6, 40.6, 40.5, 40.4, 40.4, 40.3, 40.3, 40.2, 40.0, 39.9, 39.8, 39.8, 39.2, 37.6, 37.5, 37.3, 37.3, 37.3, 37.2, 37.2, 37.2, 37.1, 37.0, 37.0, 37.0, 36.9, 36.9, 36.8, 36.8, 36.8, 36.7, 36.7, 36.6, 31.5, 31.4, 31.4, 31.3, 31.2, 31.1, 31.1, 30.2, 29.5, 29.5, 29.4, 29.4, 29.3, 28.8, 28.8, 27.8. **IR** ν (cm^{-1}): 2988, 2900, 1607, 1556, 1445, 1301. **HRMS** (MALDI) *m/z*: Calculated for $C_{48}H_{60}N_2$ 664.4756. Found 664.4761 $[M]^+$.

3.10.2) *N*[1],*N*[4]-dimethyl-*N*[1]-(4-(methyl(4-(4,4,5,5-tetramethyl-1,3,2-dioxaborolan-2-yl)phenyl)amino)phenyl)-*N*[4]-(4-(methyl(phenyl)amino)phenyl)benzene-1,4-diamine (17)

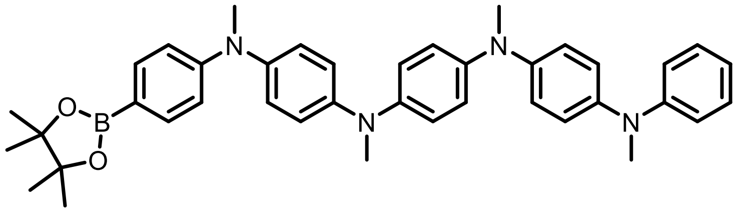

Track ***N*[1],*N*[4]-dimethyl-*N*[1]-(4-(methyl(4-(4,4,5,5-tetramethyl-1,3,2-dioxaborolan-2-yl)phenyl)amino)phenyl)-*N*[4]-(4-(methyl(phenyl)amino)phenyl)benzene-1,4-diamine (17)** was subjected to General Procedure D. The crude reaction mixture was filtered through Celite ® and washed with hexane. The solvent was removed under reduced pressure and the resulting residue was immediately subject to analysis by MALDI HRMS using dithranol (10mg/mL in THF) as the matrix. Figure S3 shows a plot of laser power vs. relative abundance. Due to the different sublimation profile of the components of the reaction mixture (**di-17**, m/z 686; **tri-17**, m/z 780; **tetra-17**, m/z 874; **penta-17**, m/z 968; **hexa-17**, m/z 1062; **hepta-17**, m/z 1156; **octa-17**, m/z 1250; *note that an additional unidentified component with m/z 710 is also observed in the spectrum*) the intensities observed in the MALDI-MS will vary with laser power. As you increase the power, **octa-substituted 17 (octa-17)** is seen in increasing relative abundance to the point at which a sufficient plume of ions is produced that accurately represents the total population of species in the original sample. Increasing the laser power further has very little effect on this population and only results in a loss of spectral resolution due to the increased number of ions generated.

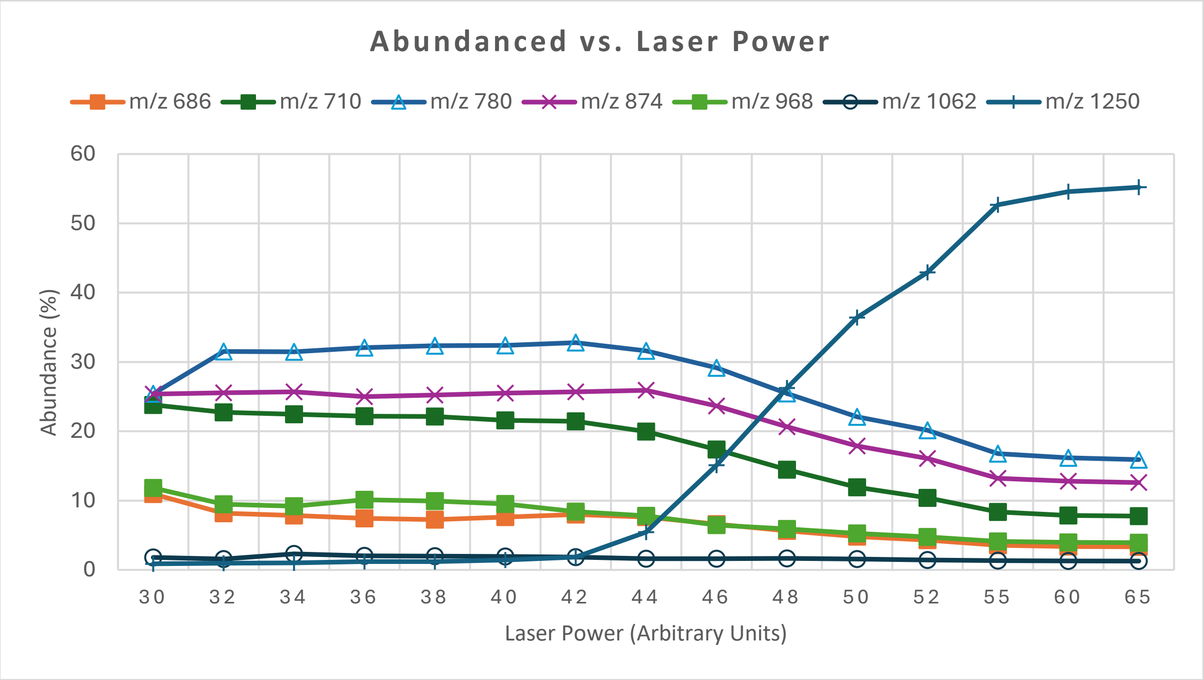

Figure S3 Chart of laser power vs. abundance for the MALDI-MS analysis of the crude reaction mixture of track ***N*[1],*N*[4]-dimethyl-*N*[1]-(4-(methyl(4-(4,4,5,5-tetramethyl-1,3,2-dioxaborolan-2-yl)phenyl)amino)phenyl)-*N*[4]-(4-(methyl(phenyl)amino)phenyl)benzene-1,4-diamine (17)** subject to General Procedure D.

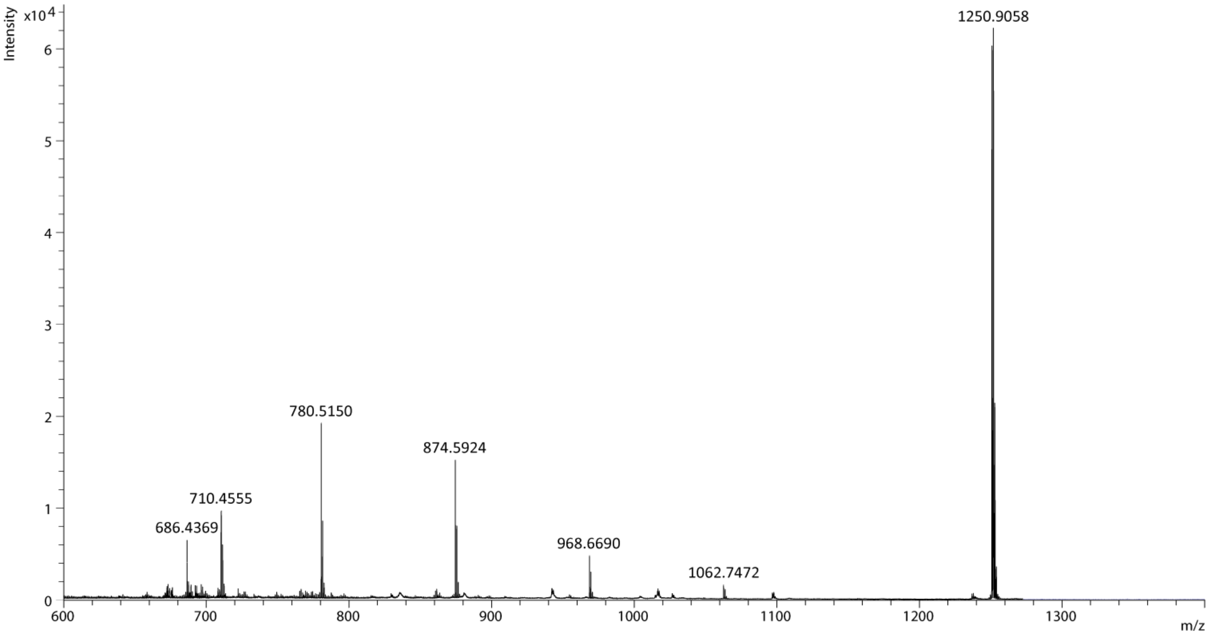

Figure S4 MALDI HRMS spectrum of crude reaction mixture of track ***N*[1],*N*[4]-dimethyl-*N*[1]-(4-(methyl(4-(4,4,5,5-tetramethyl-1,3,2-dioxaborolan-2-yl)phenyl)amino)phenyl)-*N*[4]-(4-(methyl(phenyl)amino)phenyl)benzene-1,4-diamine (17)** subjected to General Procedure D at 55 laser power.

Based on the analysis detailed in Figures S3 and S4 above, we conclude that **octa-substituted 17 (octa-17)** is the major component of the reaction mixture at approximately 55% of the total.

Octa-substituted 17 (**octa-17**)

HRMS (MALDI) *m/z*: Calculated for $C_{90}H_{114}N_4$ 1250.9043. Found 1250.9058 $[M]^+$.

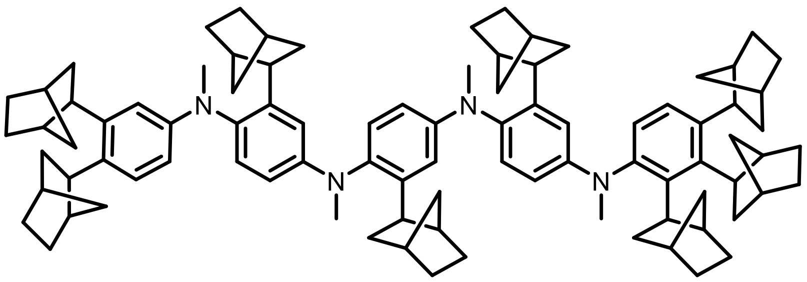

4) NMR and Selected HRMS Spectra

4-Bromo-4'-fluoro-1,1'-biphenyl (S1) (^{1}H NMR, 400 MHz, $CDCl_3$)

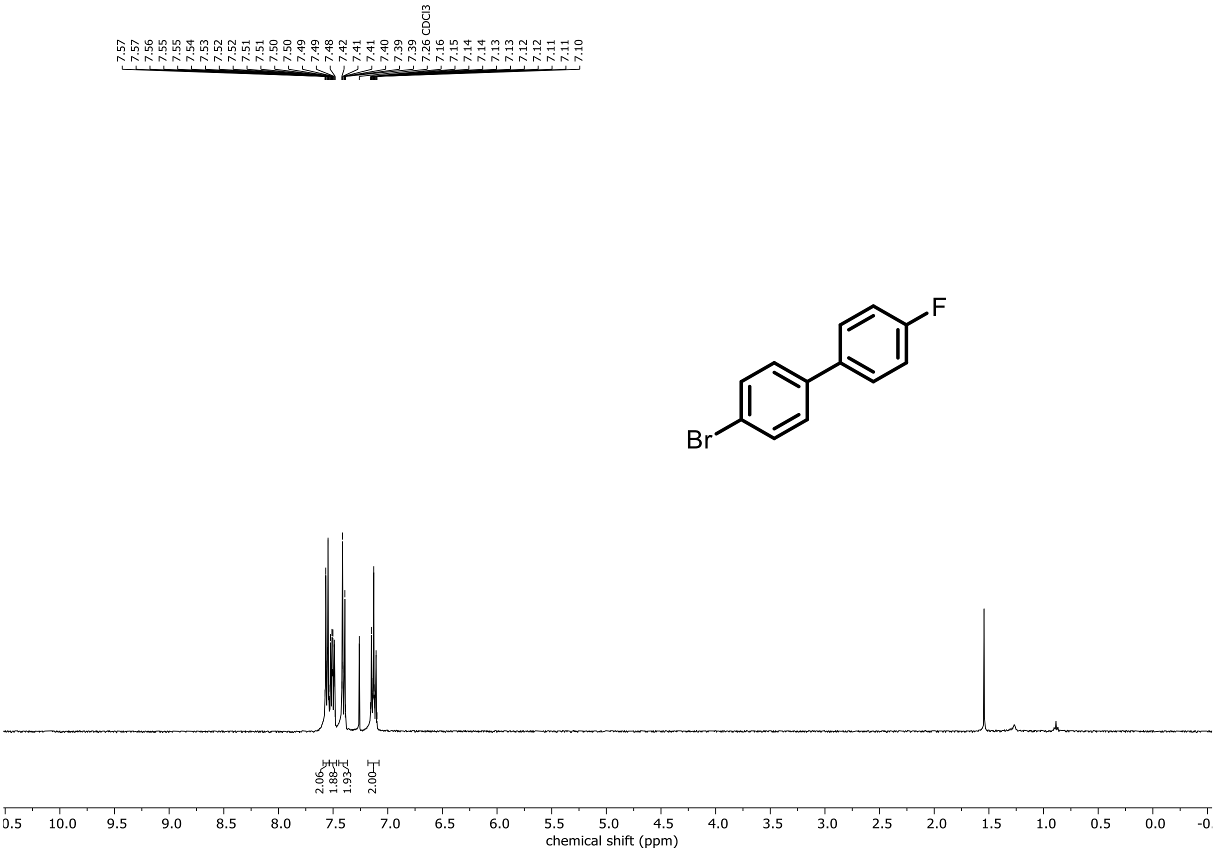

4-Bromo-4'-fluoro-1,1'-biphenyl (S1) (^{13}C NMR, 101 MHz, $CDCl_3$)

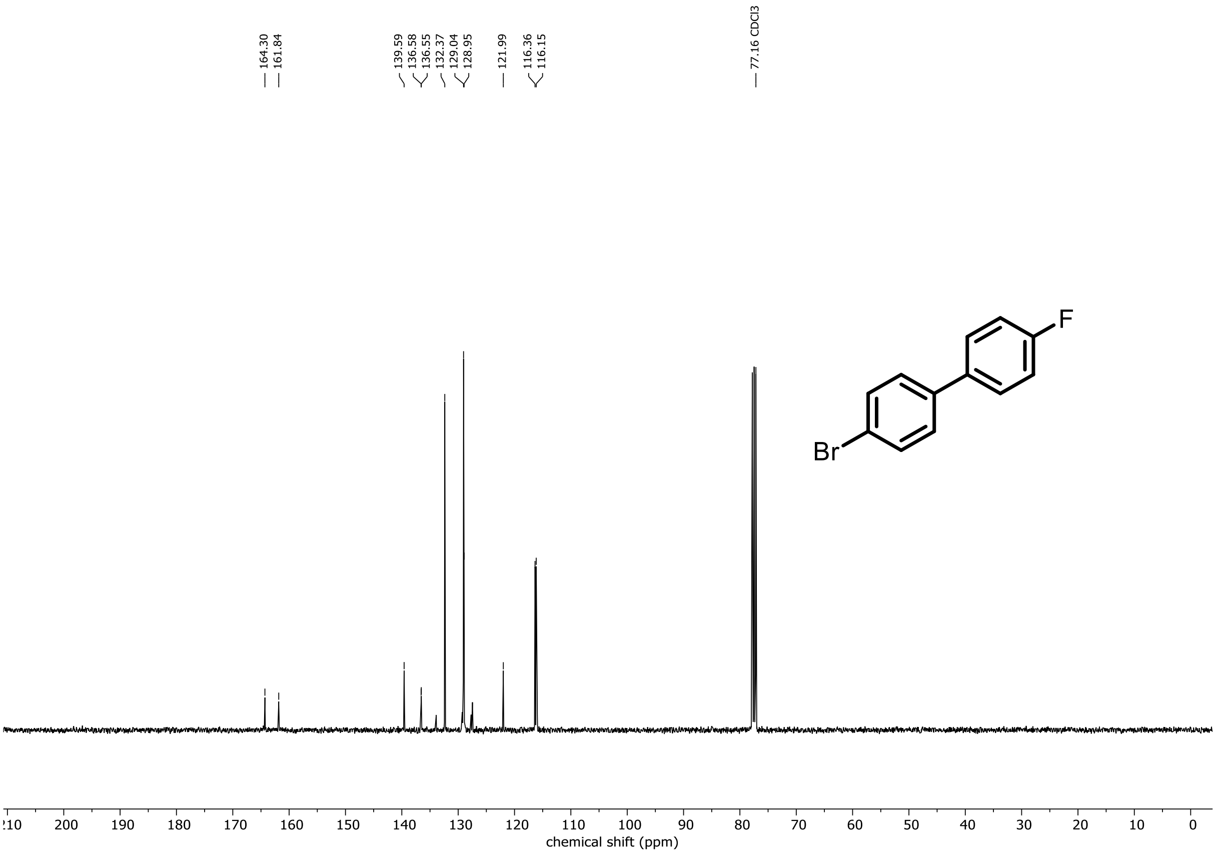

4-Bromo-4'-fluoro-1,1'-biphenyl (S1) (^{19}F NMR, 376 MHz, $CDCl_3$)

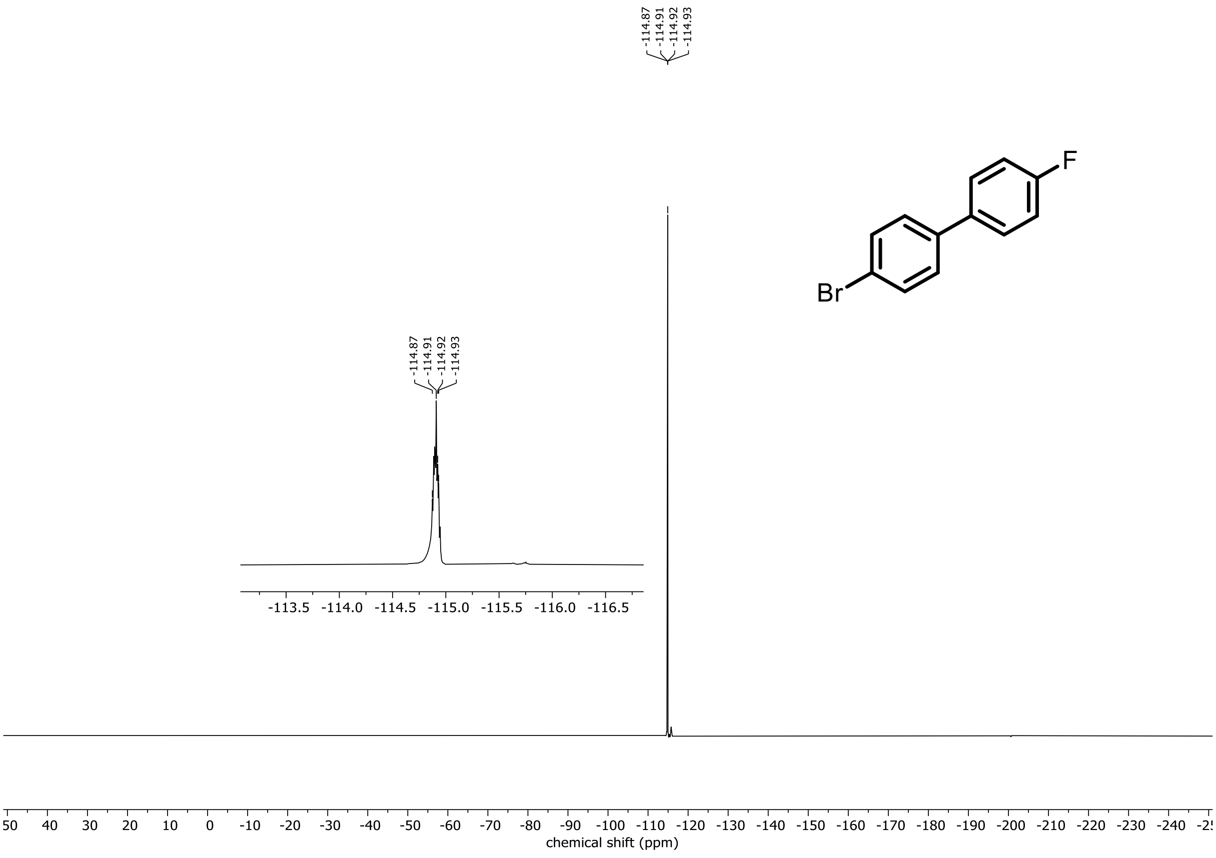

4-Bromo-4'-(trifluoromethyl)-1,1'-biphenyl (S2) (^{1}H NMR, 400 MHz, $CDCl_3$)

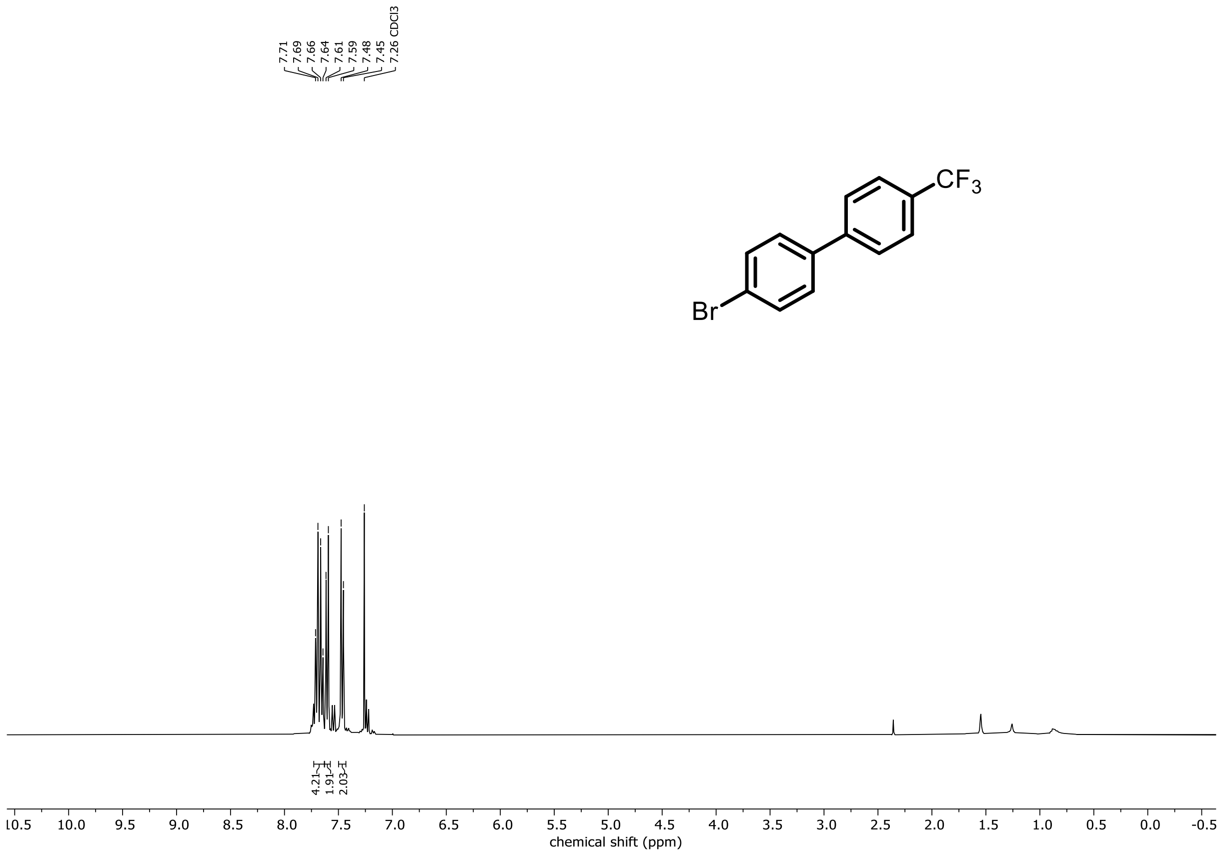

4-Bromo-4'-(trifluoromethyl)-1,1'-biphenyl (S2) (^{13}C NMR, 101 MHz, $CDCl_3$)

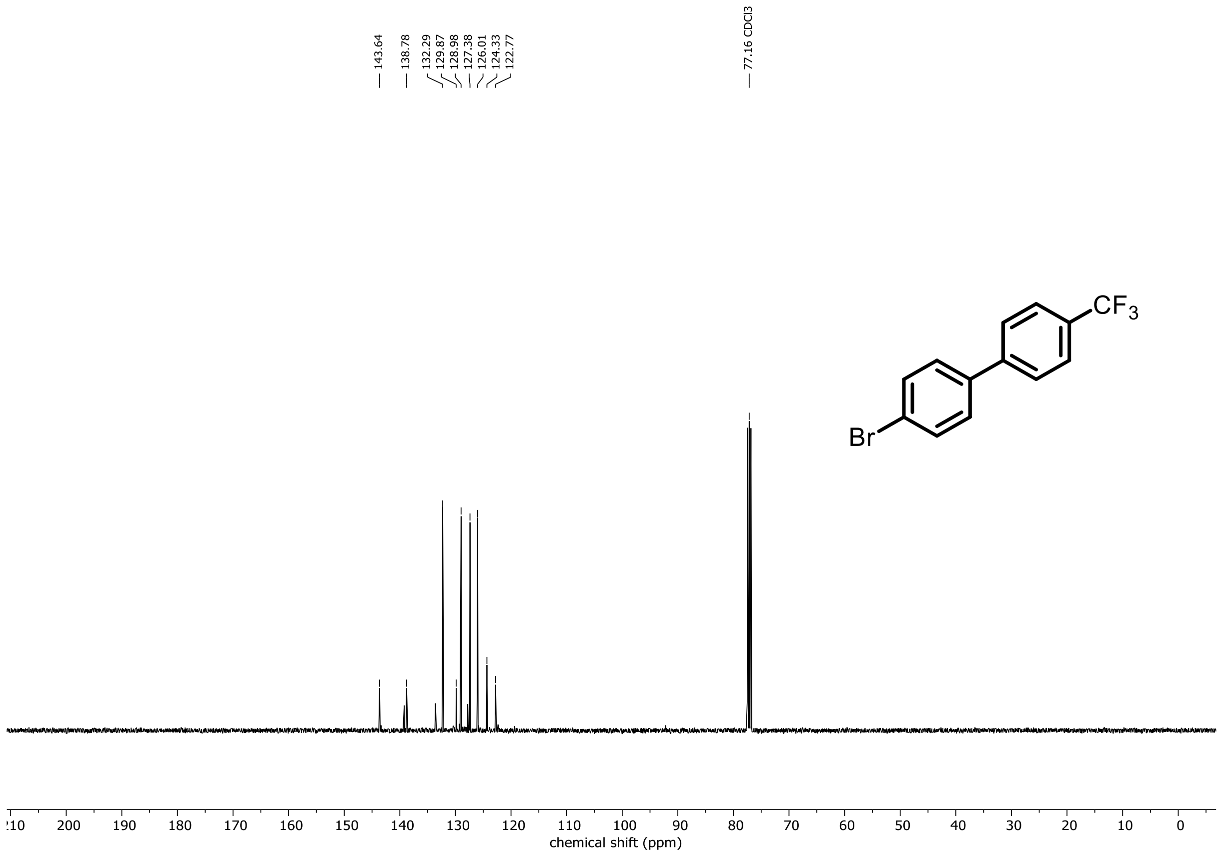

4-Bromo-4'-(trifluoromethyl)-1,1'-biphenyl (S2) (^{19}F NMR, 376 MHz, $CDCl_3$)

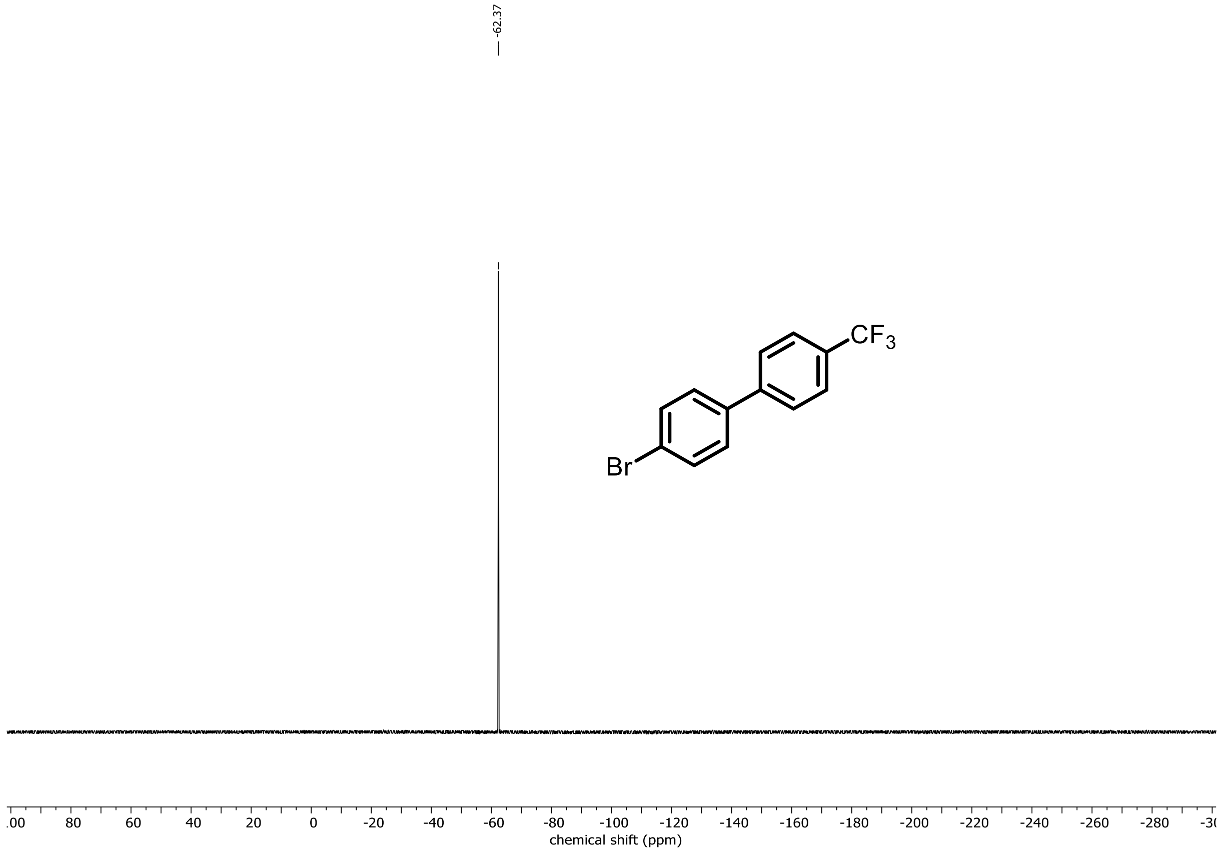

4-Bromo-4'-(methoxy)-1,1'-biphenyl (S3) (^{1}H NMR, 400 MHz, $CDCl_3$)

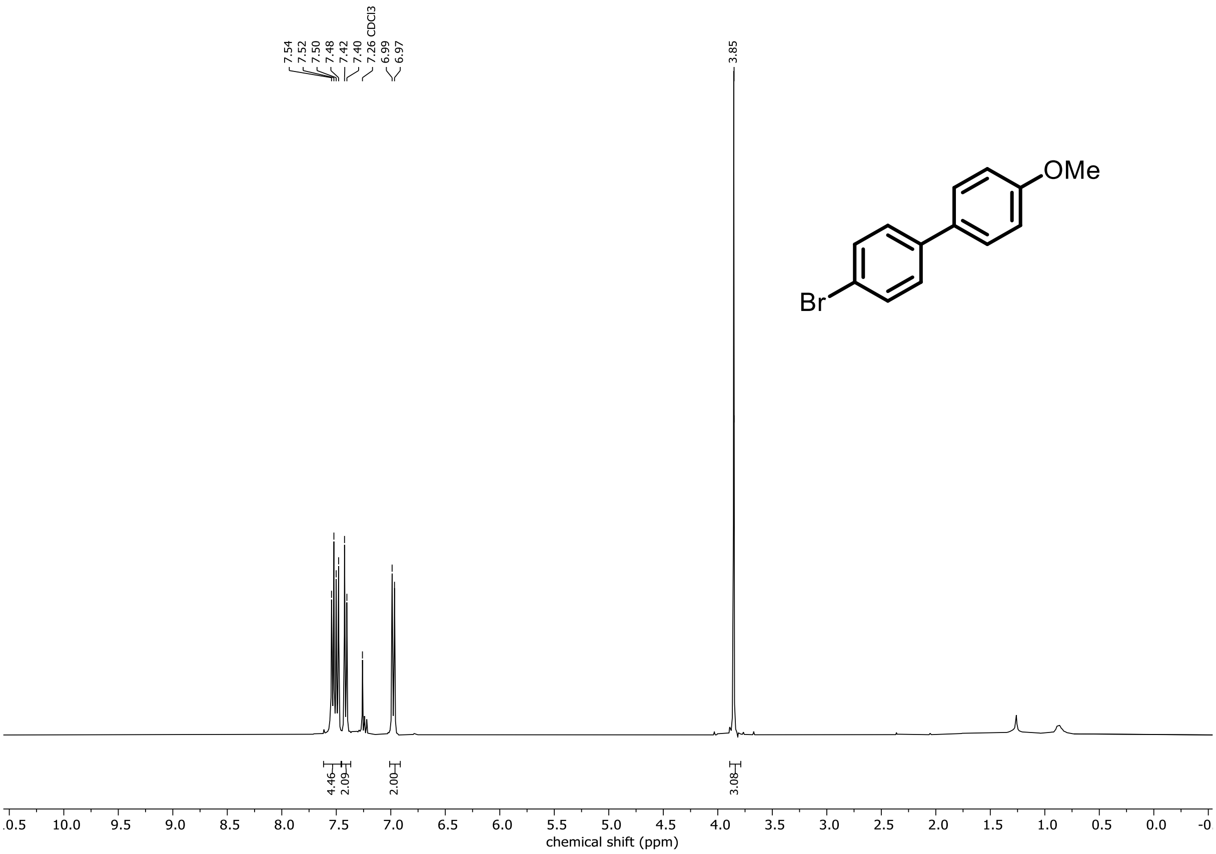

4-Bromo-4'-(methoxy)-1,1'-biphenyl (S3) (^{13}C NMR, 101 MHz, $CDCl_3$)

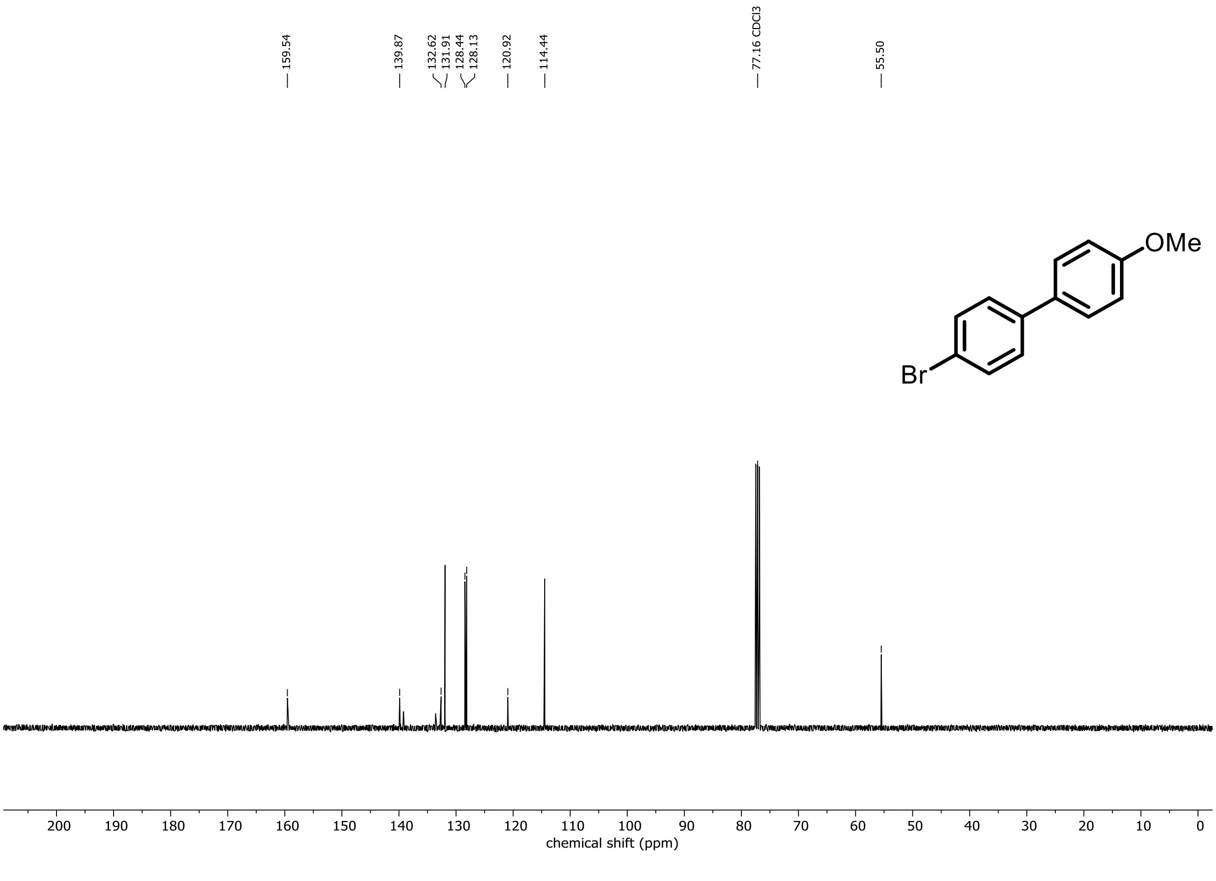

4-Bromo-3'-(methoxy)-1,1'-biphenyl (S4) (^{1}H NMR, 400 MHz, $CDCl_3$)

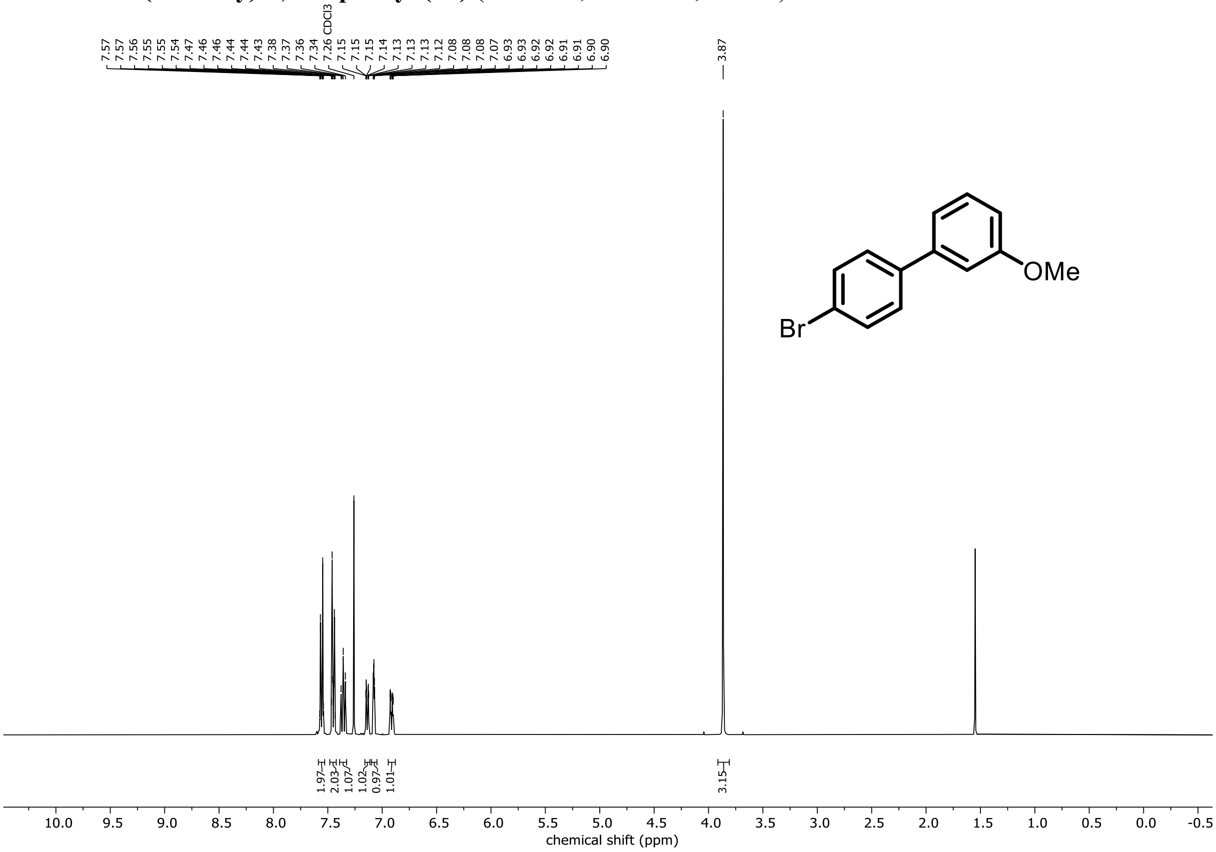

4-Bromo-3'-(methoxy)-1,1'-biphenyl (S4) (^{13}C NMR, 101 MHz, $CDCl_3$)

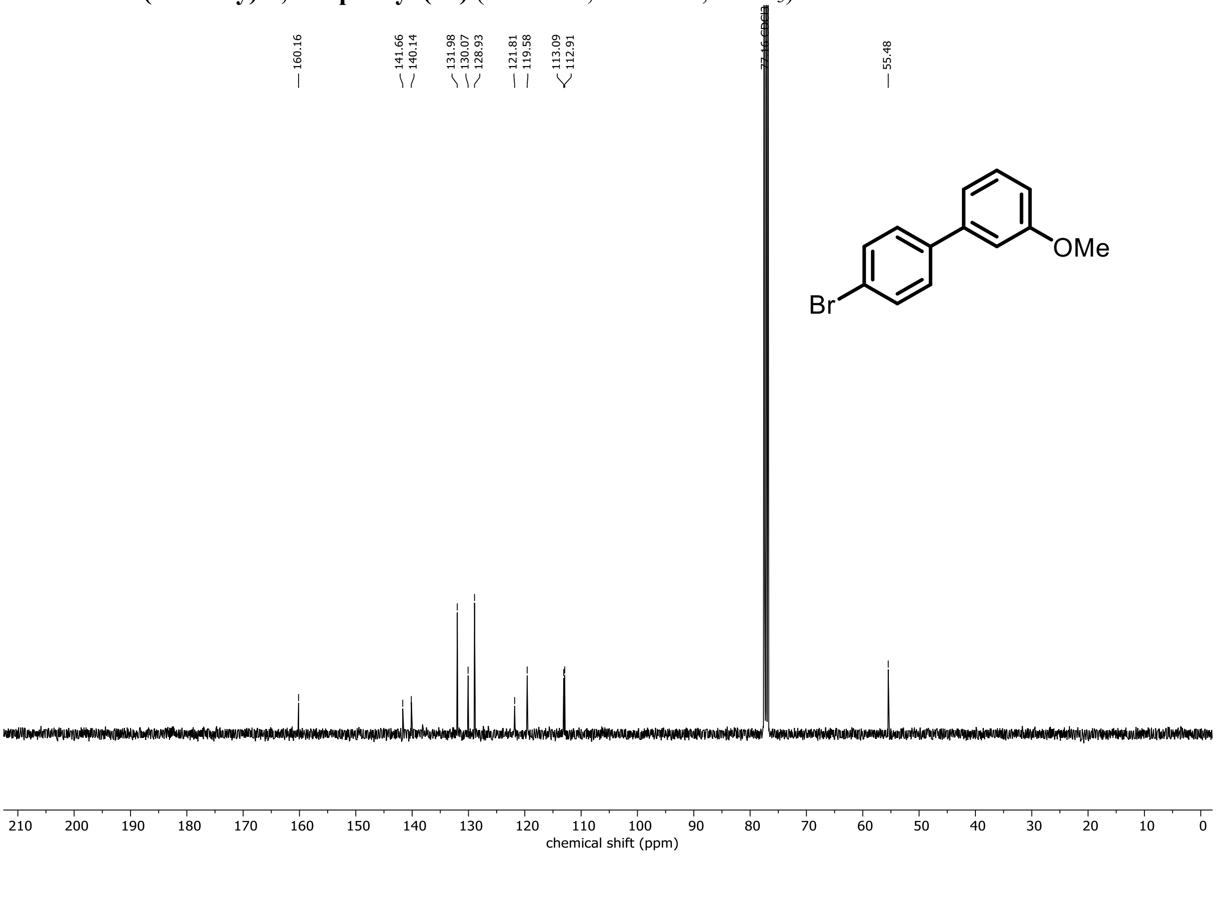

4-Bromo-4'-methyl-1,1'-biphenyl (S5) (^{1}H NMR, 400 MHz, $CDCl_3$)

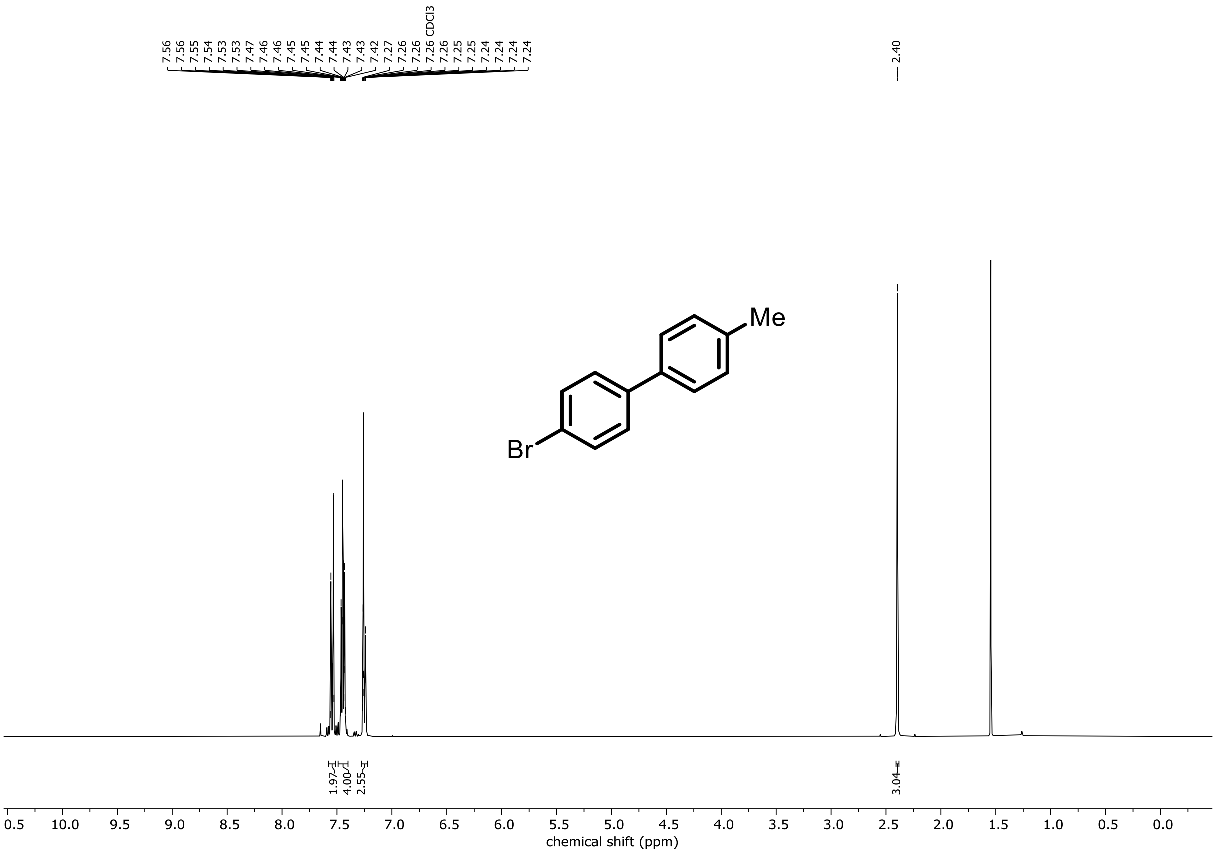

4-Bromo-4'-methyl-1,1'-biphenyl (S5) (^{13}C NMR, 101 MHz, $CDCl_3$)

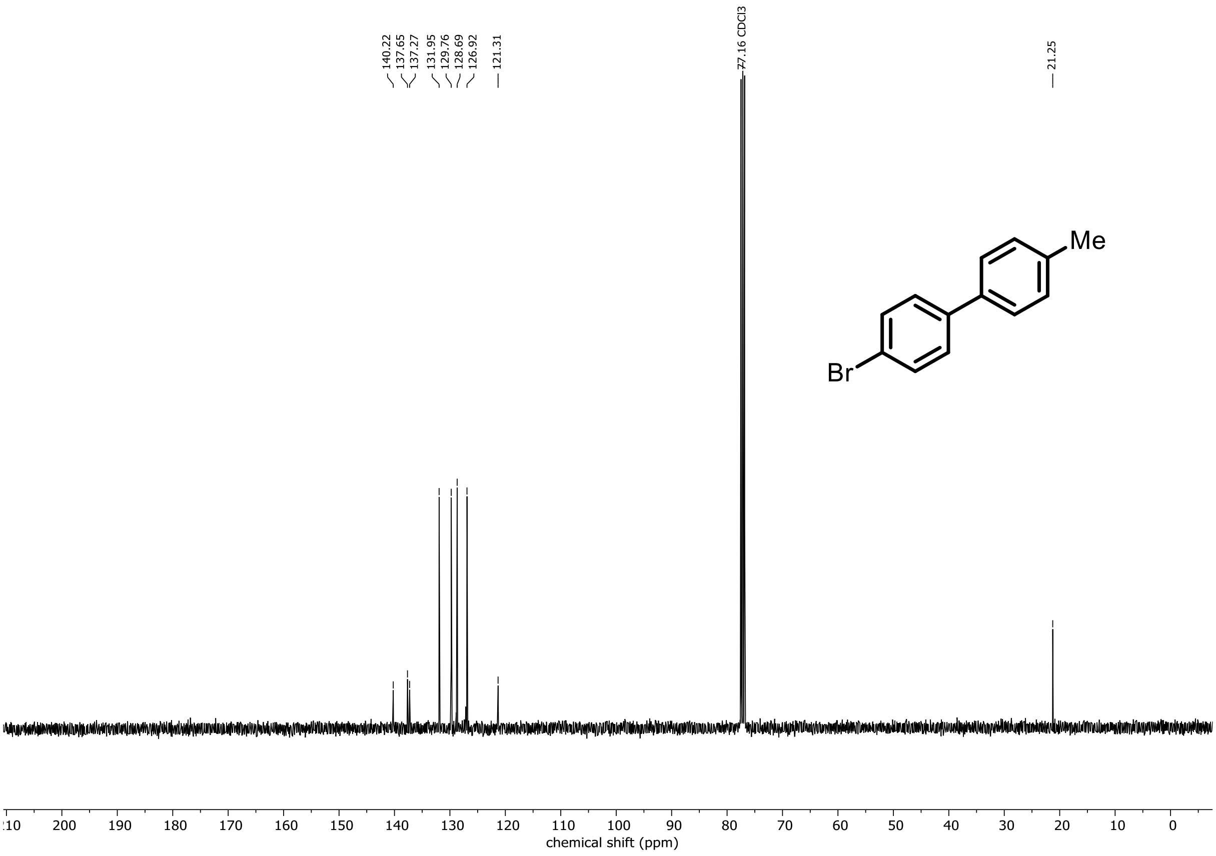

4-Chloro-4'-fluoro-2-methyl-1,1'-biphenyl (S6) (^{1}H NMR, 400 MHz, $CDCl_3$)

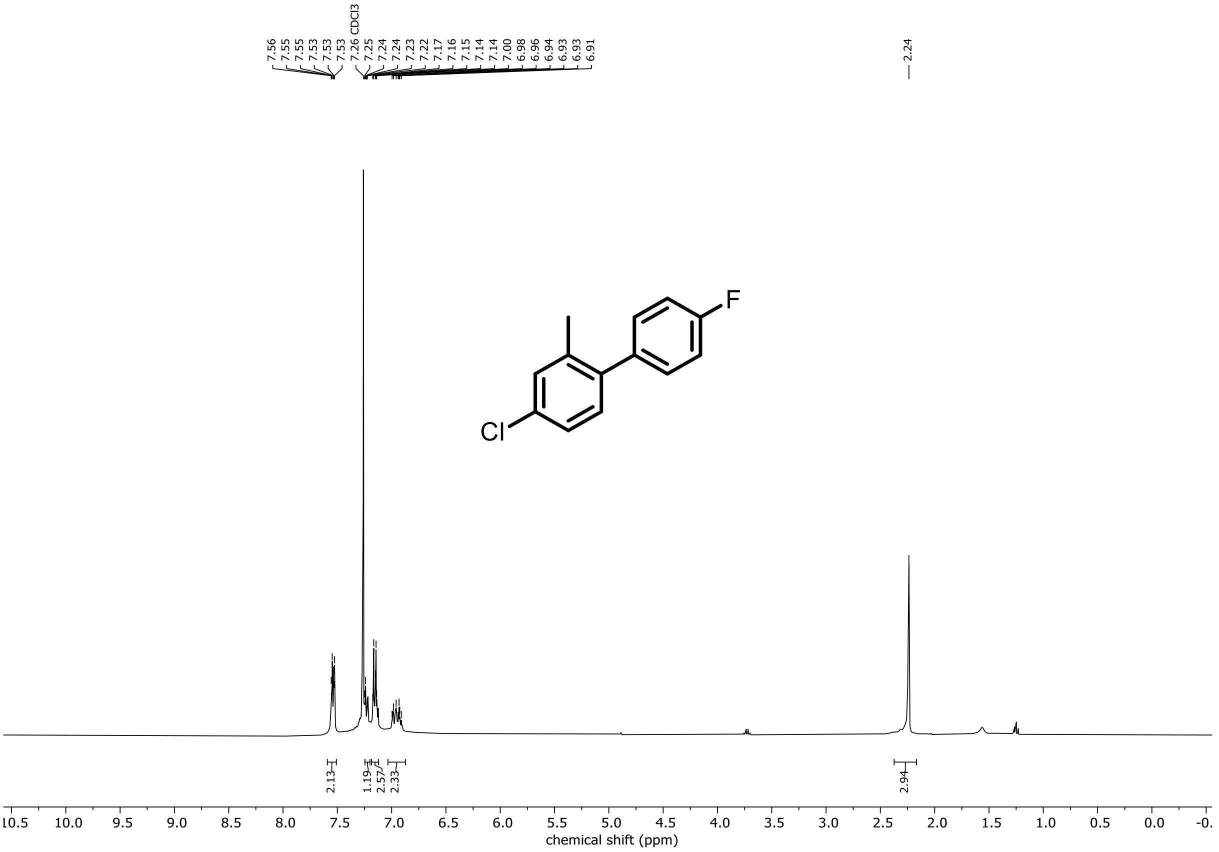

4-Chloro-4'-fluoro-2-methyl-1,1'-biphenyl (S6) (^{13}C NMR, 126 MHz, $CDCl_3$)

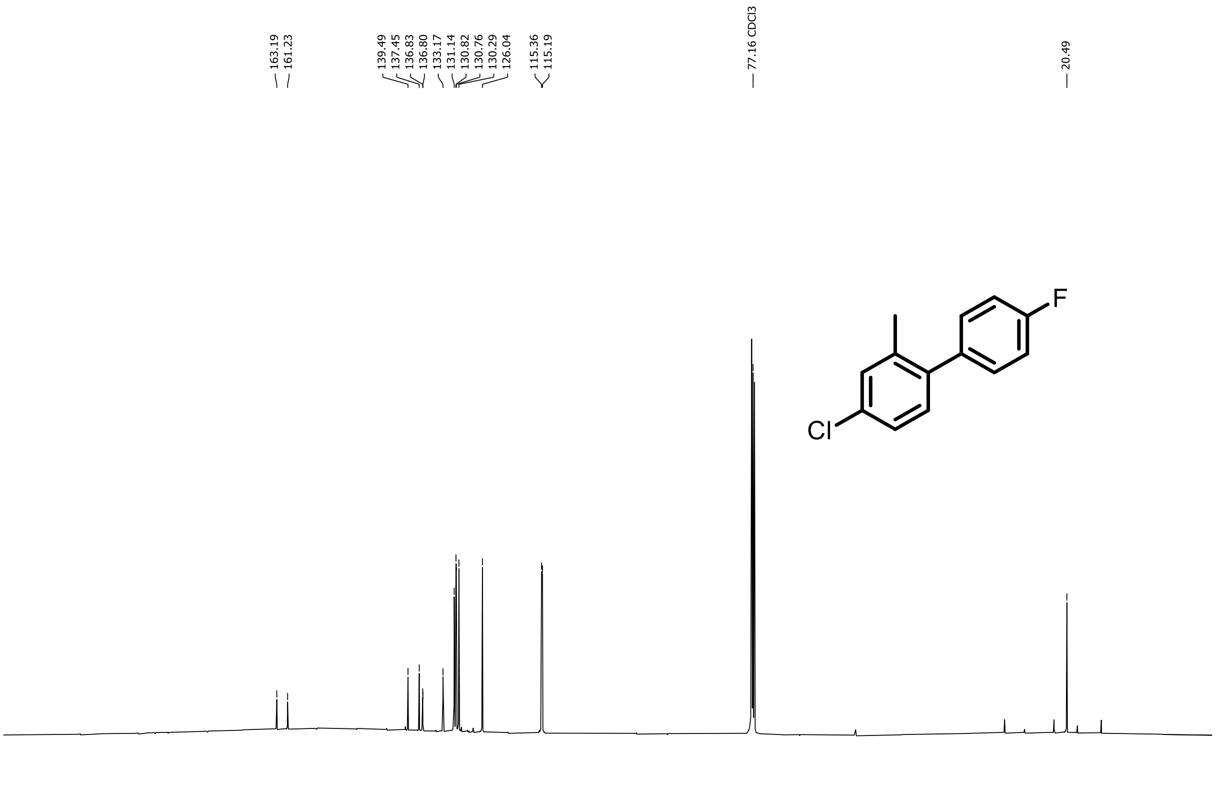

4-Chloro-4'-fluoro-2-methyl-1,1'-biphenyl (S6) (^{19}F NMR, 376 MHz, $CDCl_3$)

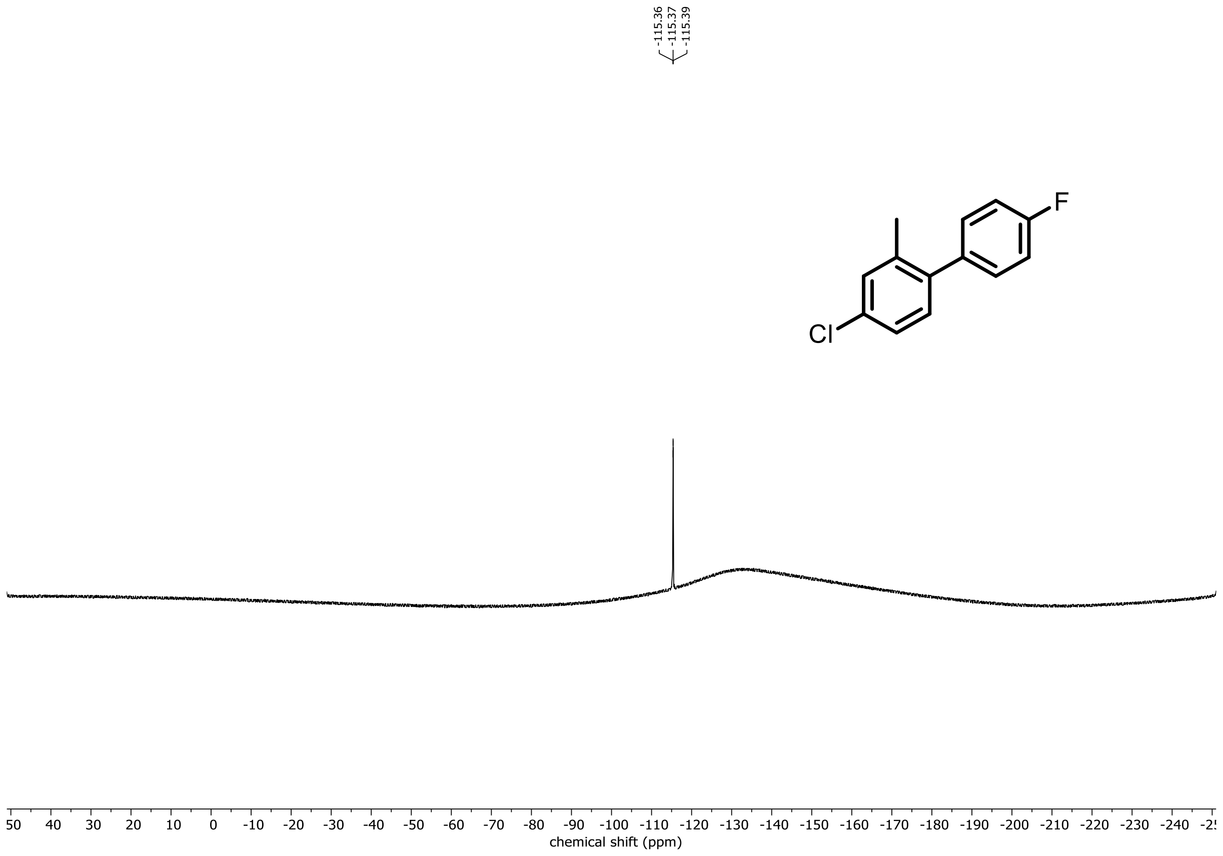

4'-Bromo-4-fluoro-2-methyl-1,1'-biphenyl (S7) (^{1}H NMR, 400 MHz, $CDCl_3$)

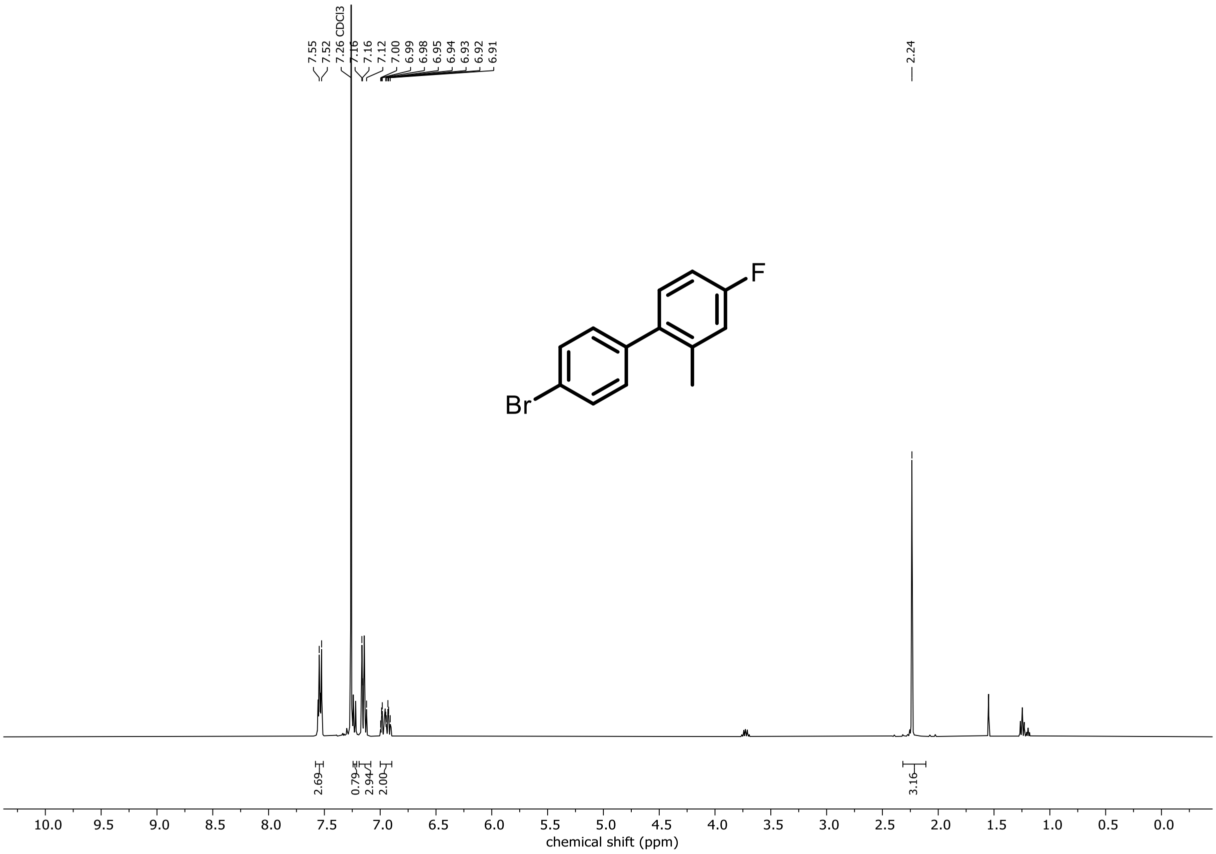

4'-Bromo-4-fluoro-2-methyl-1,1'-biphenyl (S7) (^{13}C NMR, 126 MHz, $CDCl_3$)

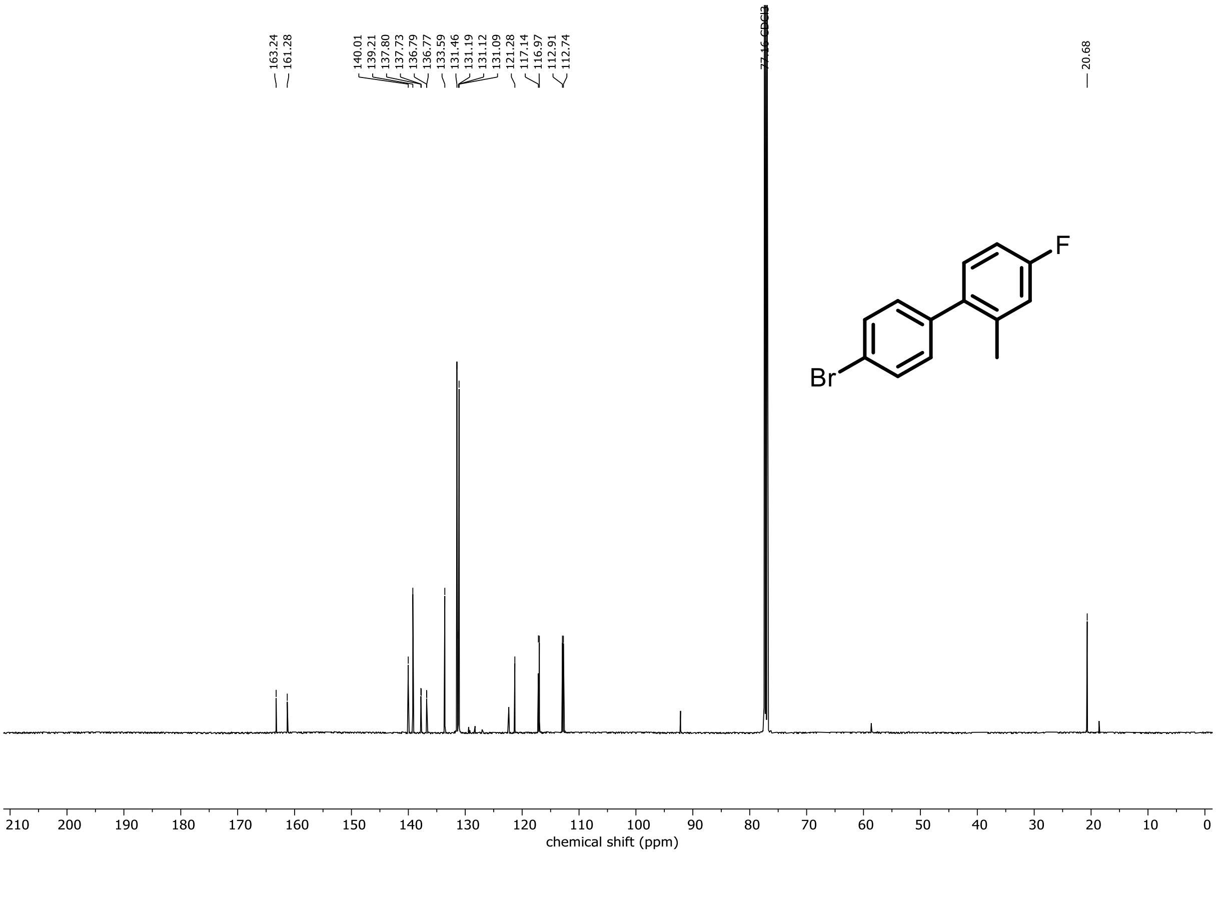

4'-Bromo-4-fluoro-2-methyl-1,1'-biphenyl (S7) (^{19}F NMR, 376 MHz, $CDCl_3$)

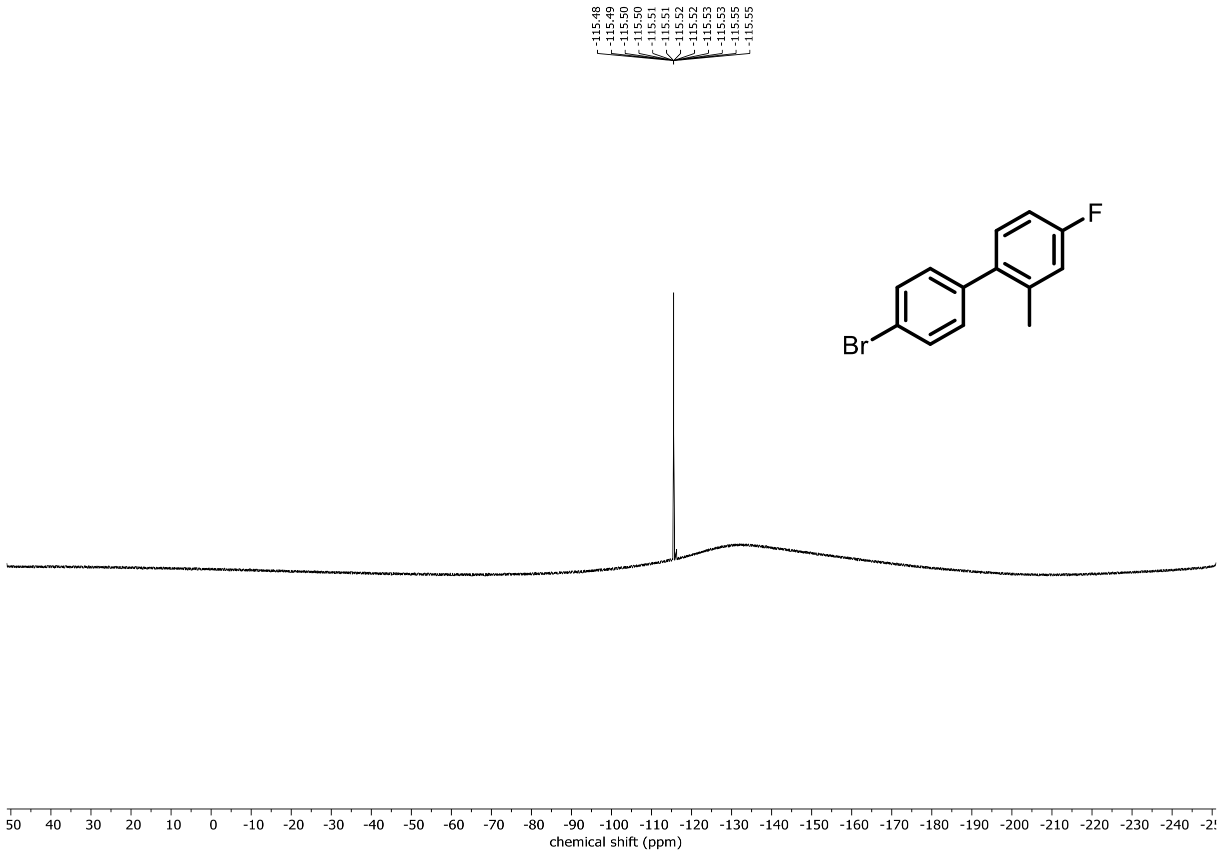

4-Chloro-4'-fluoro-2,2'-dimethyl-1,1'-biphenyl (S8) (^{1}H NMR, 400 MHz, $CDCl_3$)

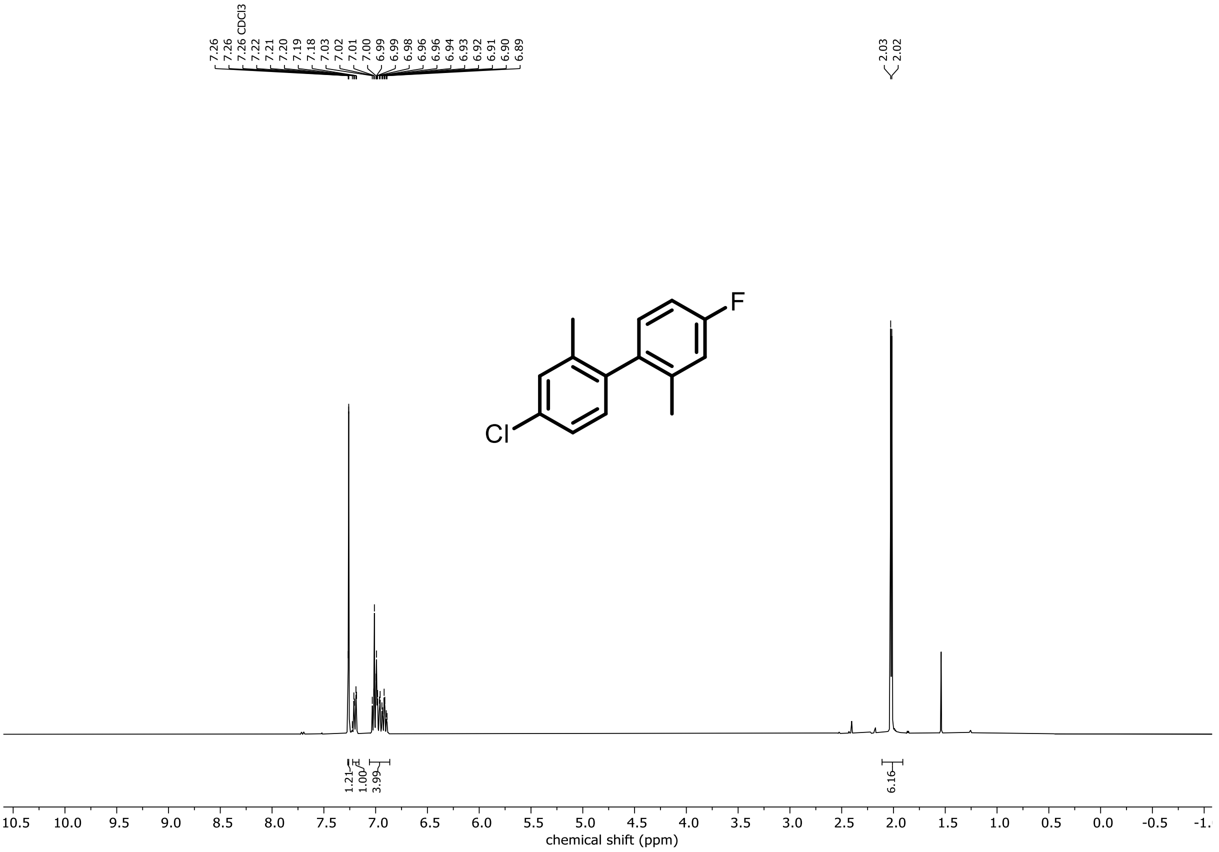

4-Chloro-4'-fluoro-2,2'-dimethyl-1,1'-biphenyl (S8) (^{13}C NMR, 101 MHz, $CDCl_3$)

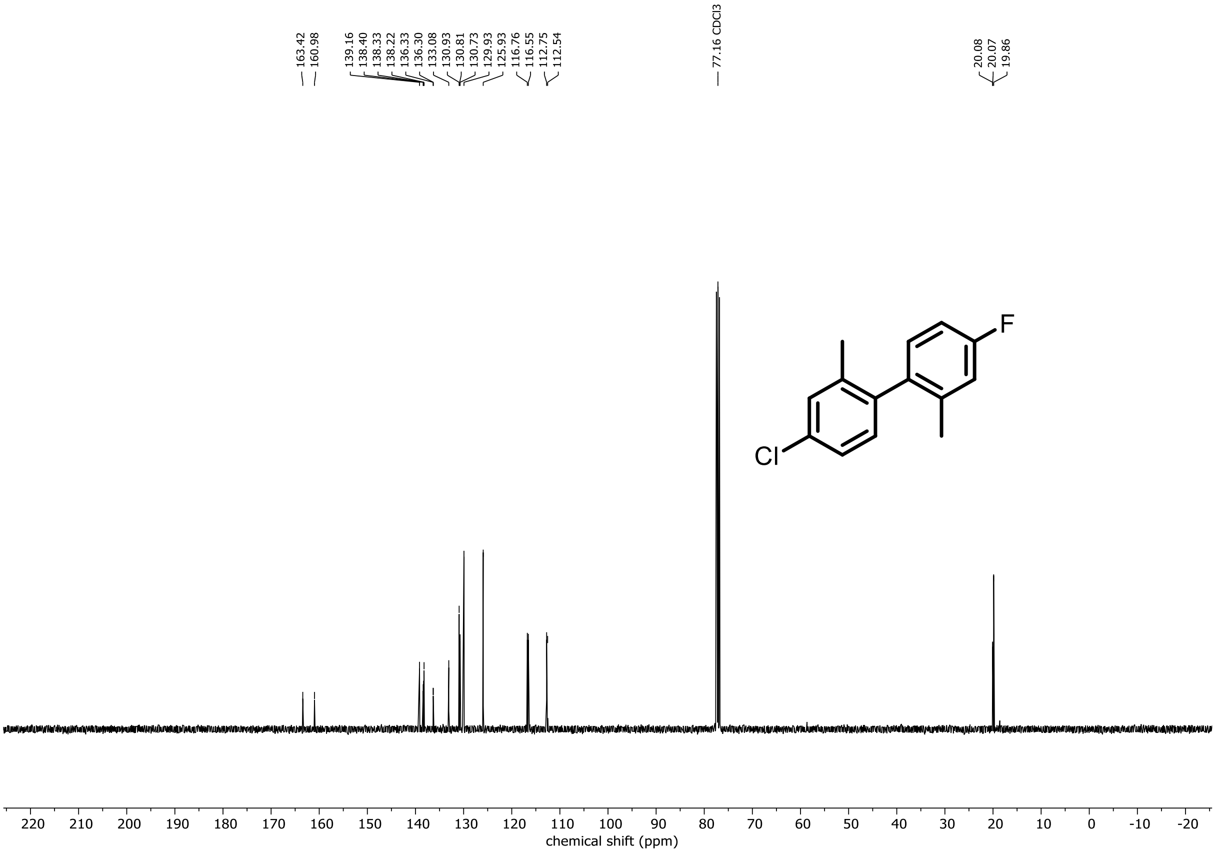

4-Chloro-4'-fluoro-2,2'-dimethyl-1,1'-biphenyl (S8) (^{19}F NMR, 376 MHz, $CDCl_3$)

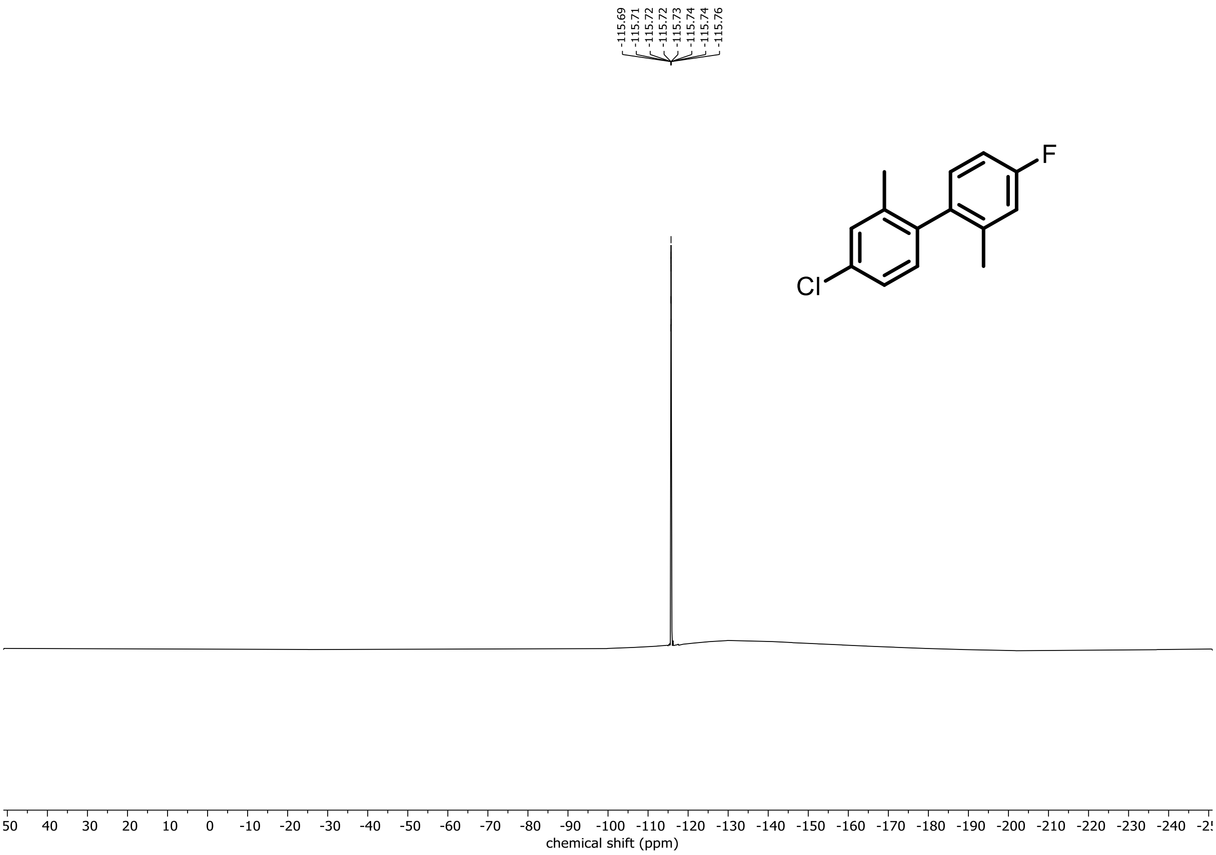

2-(4'-Fluoro-[1,1'-biphenyl]-4-yl)-4,4,5,5-tetramethyl-1,3,2-dioxaborolane (1) (^{1}H NMR, 400 MHz, $CDCl_3$)

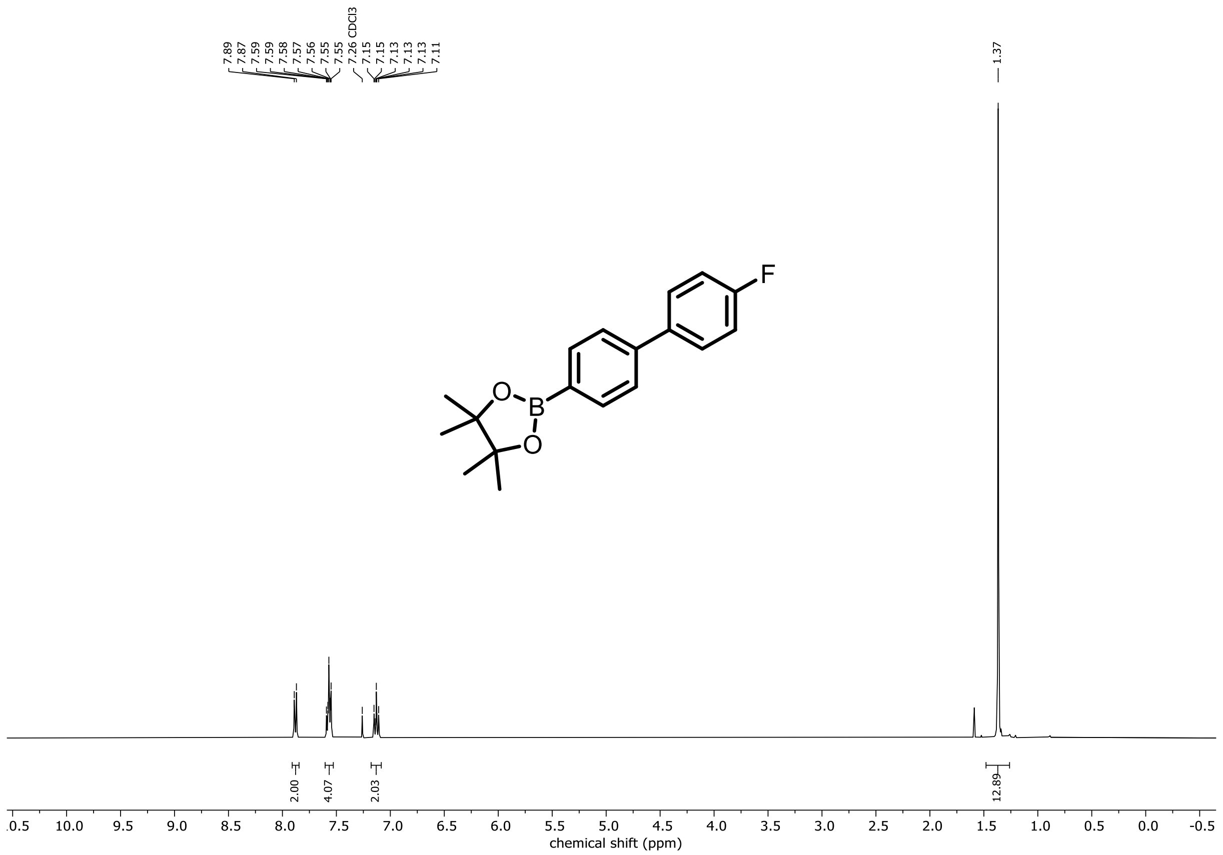

2-(4'-Fluoro-[1,1'-biphenyl]-4-yl)-4,4,5,5-tetramethyl-1,3,2-dioxaborolane (1) (^{13}C NMR, 101 MHz, $CDCl_3$)

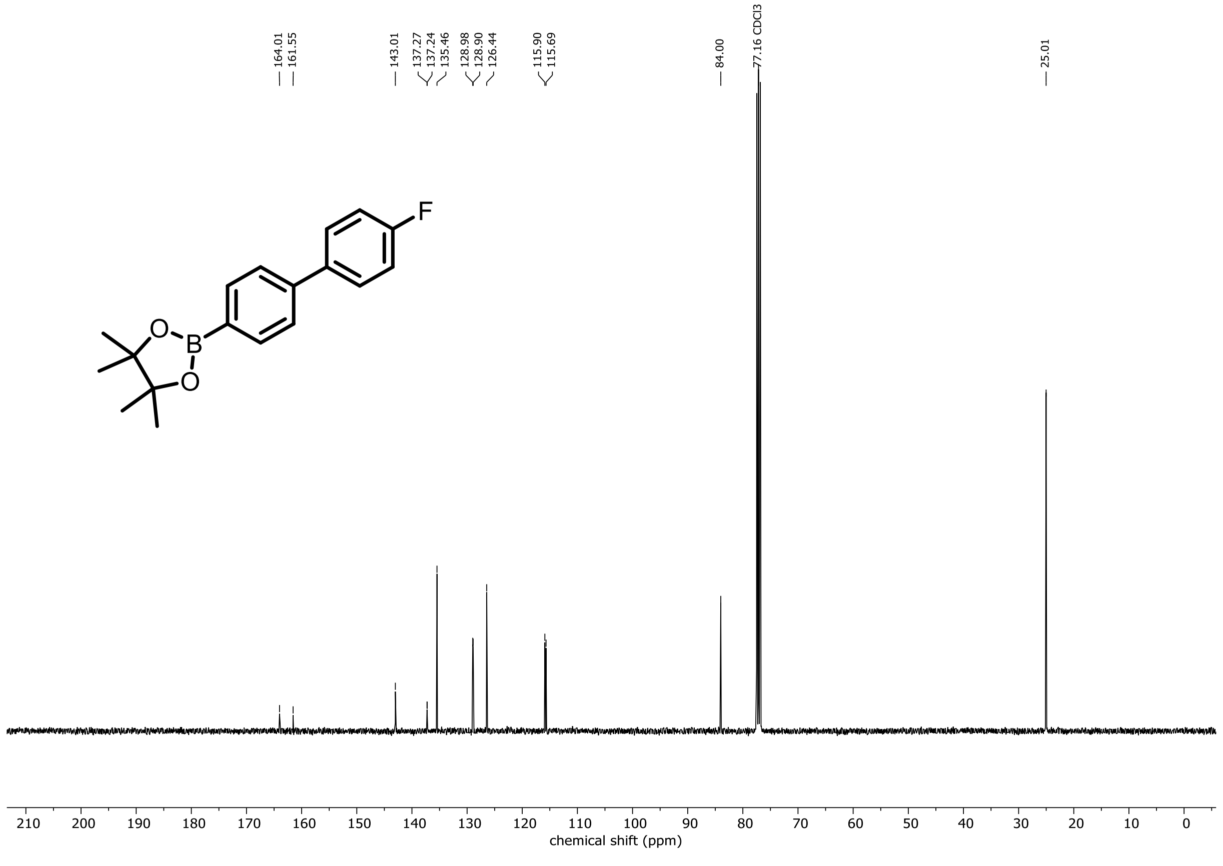

2-(4'-Fluoro-[1,1'-biphenyl]-4-yl)-4,4,5,5-tetramethyl-1,3,2-dioxaborolane (1) (^{19}F NMR, 376 MHz, $CDCl_3$)

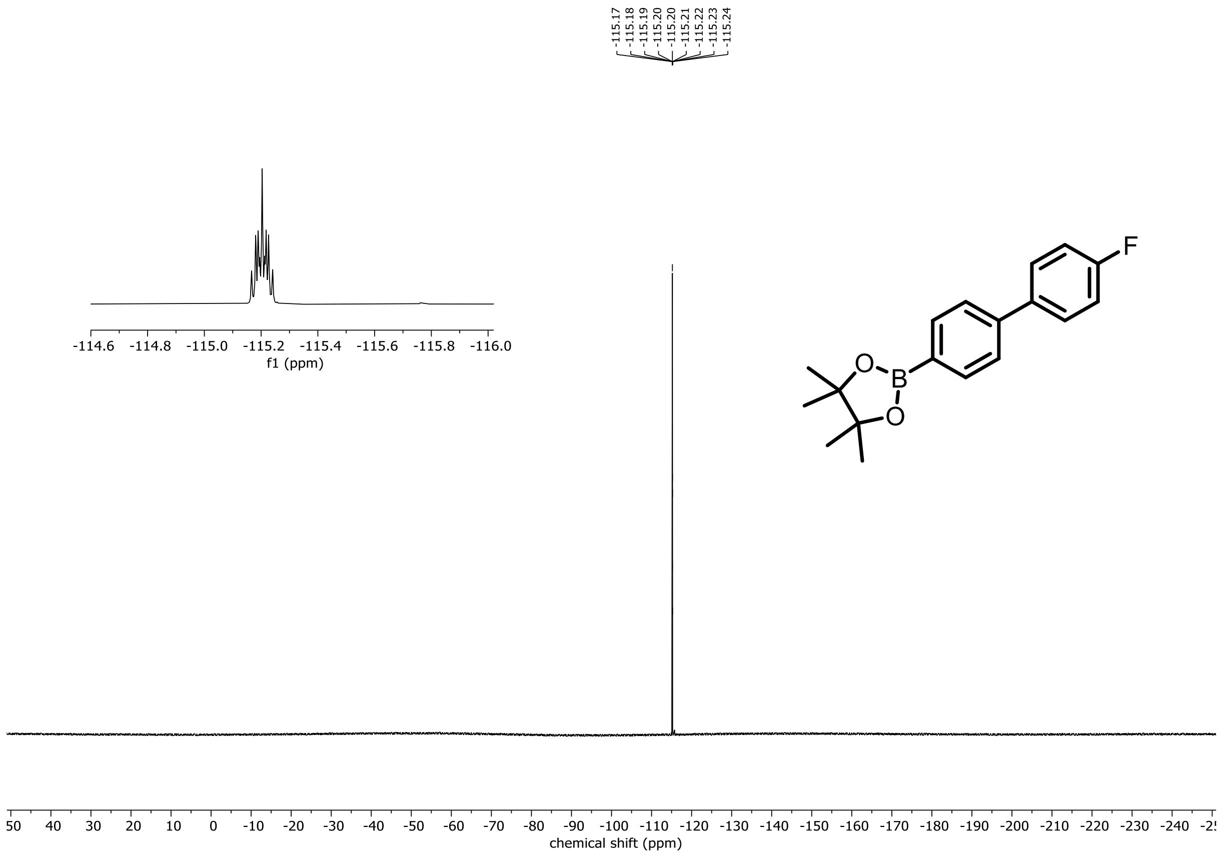

4,4,5,5-Tetramethyl-2-(4'-(trifluoromethyl)-[1,1'-biphenyl]-4-yl)-1,3,2-dioxaborolane (2) (^{1}H NMR, 400 MHz, $CDCl_3$)

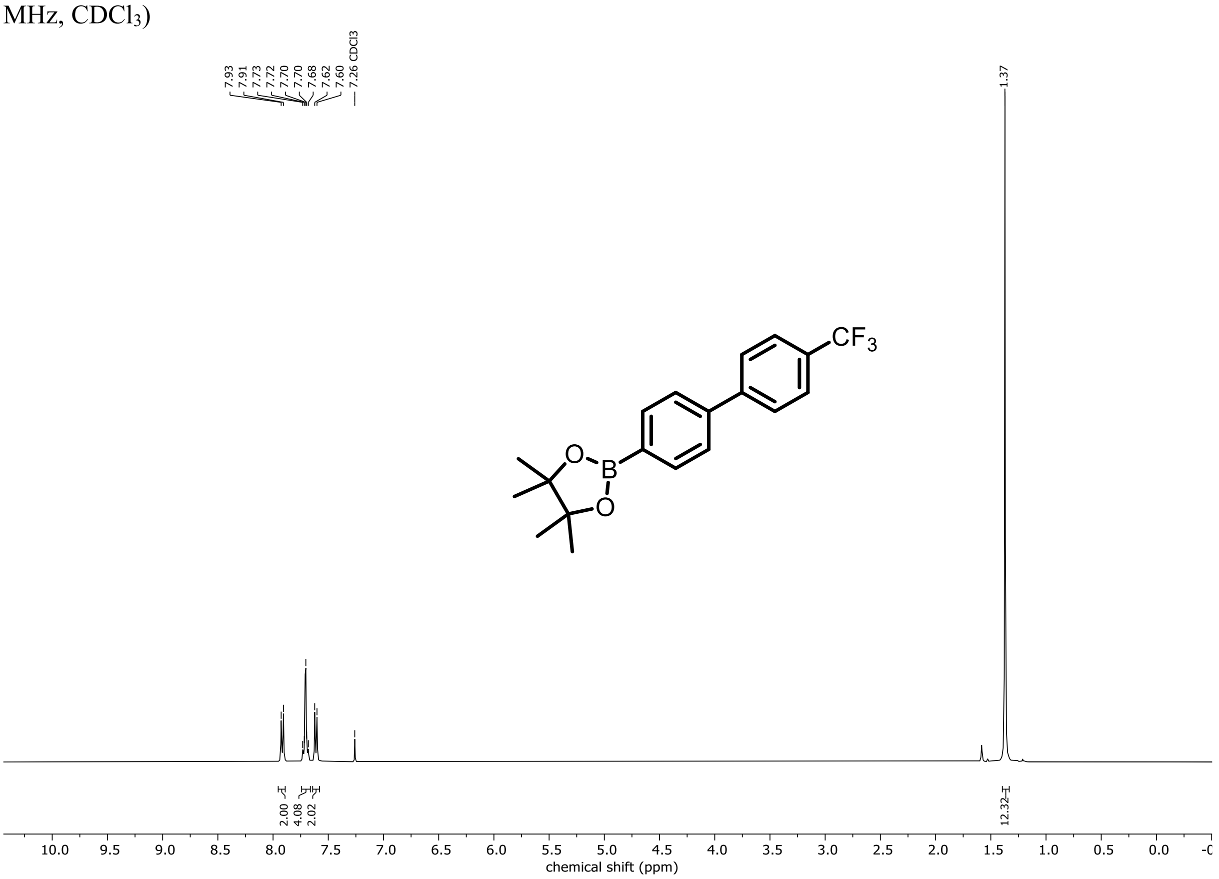

4,4,5,5-Tetramethyl-2-(4'-(trifluoromethyl)-[1,1'-biphenyl]-4-yl)-1,3,2-dioxaborolane (2) (^{13}C NMR, 101 MHz, $CDCl_3$)

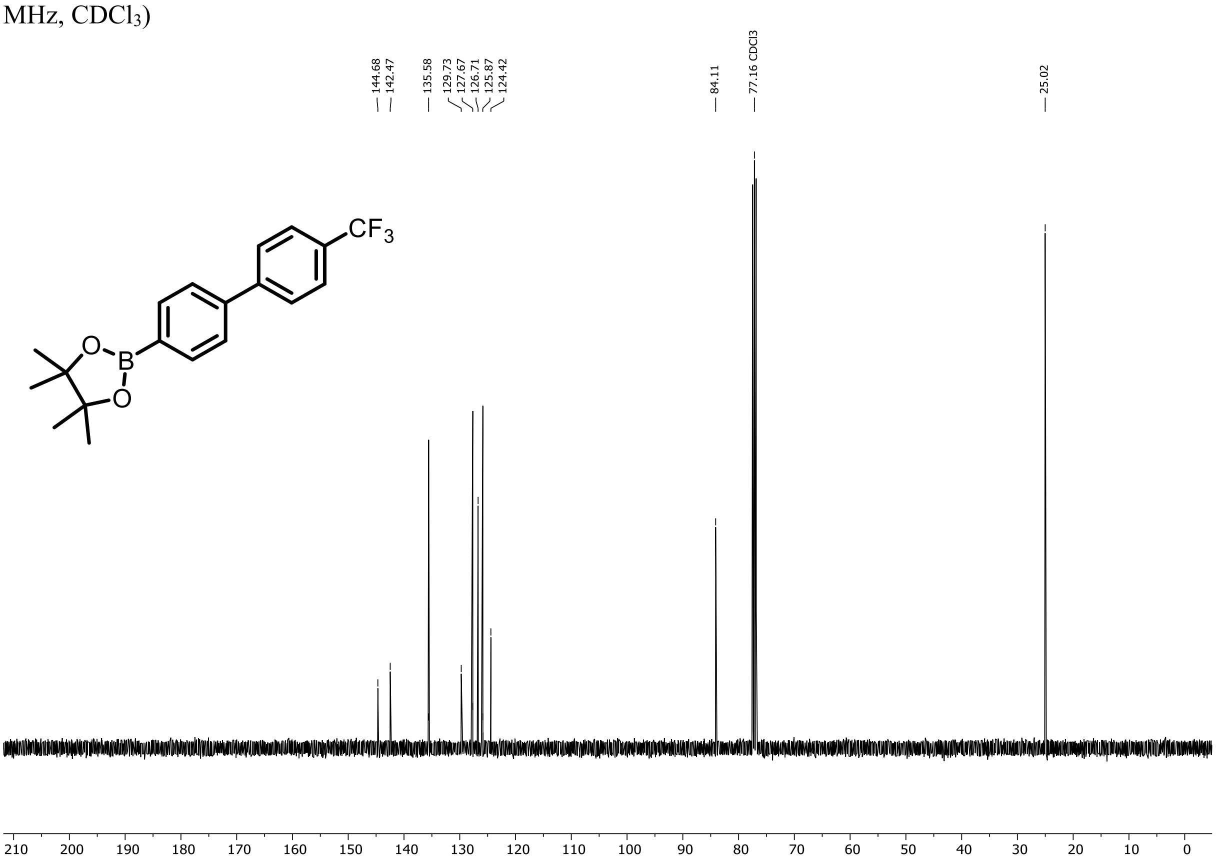

4,4,5,5-Tetramethyl-2-(4'-(trifluoromethyl)-[1,1'-biphenyl]-4-yl)-1,3,2-dioxaborolane (2) (^{19}F NMR, 376 MHz, $CDCl_3$)

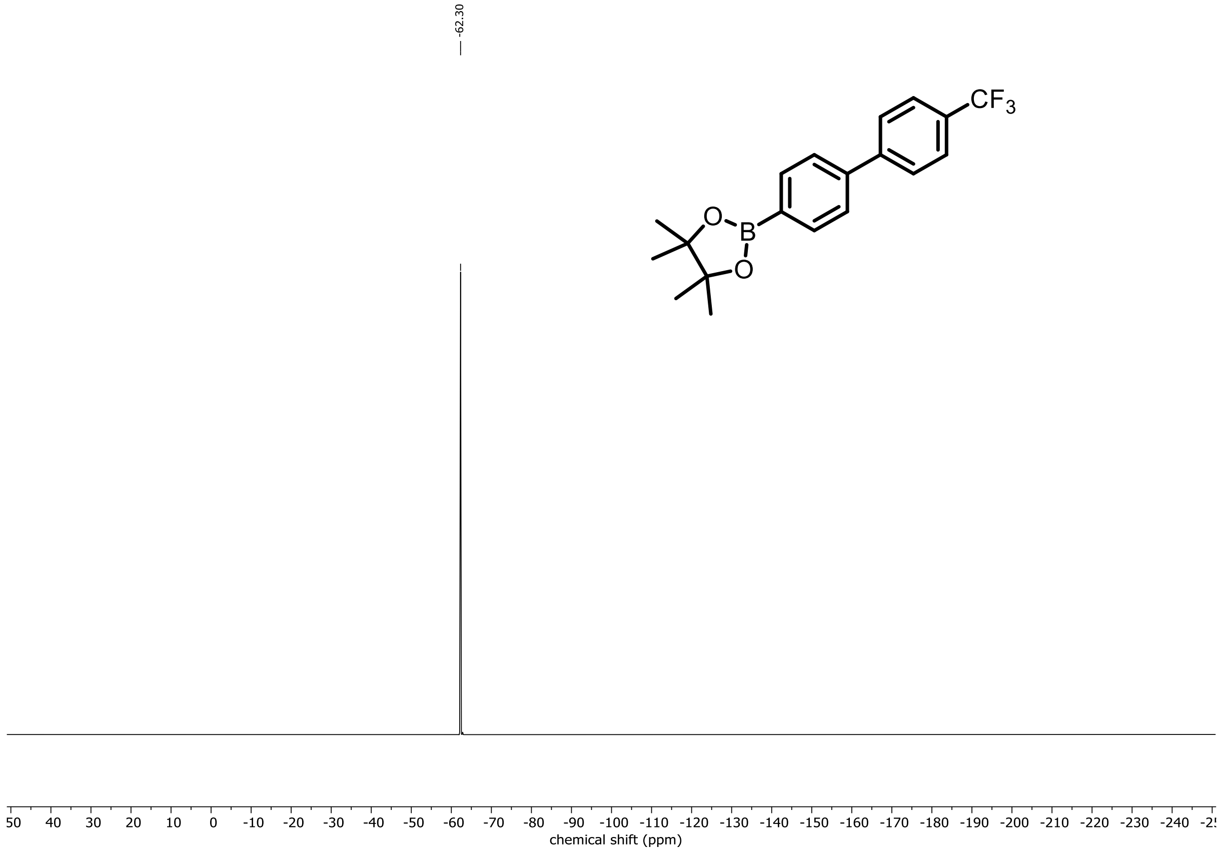

2-(4'-Methoxy-[1,1'-biphenyl]-4-yl)-4,4,5,5-tetramethyl-1,3,2-dioxaborolane (3) (^{1}H NMR, 400 MHz, $CDCl_3$)

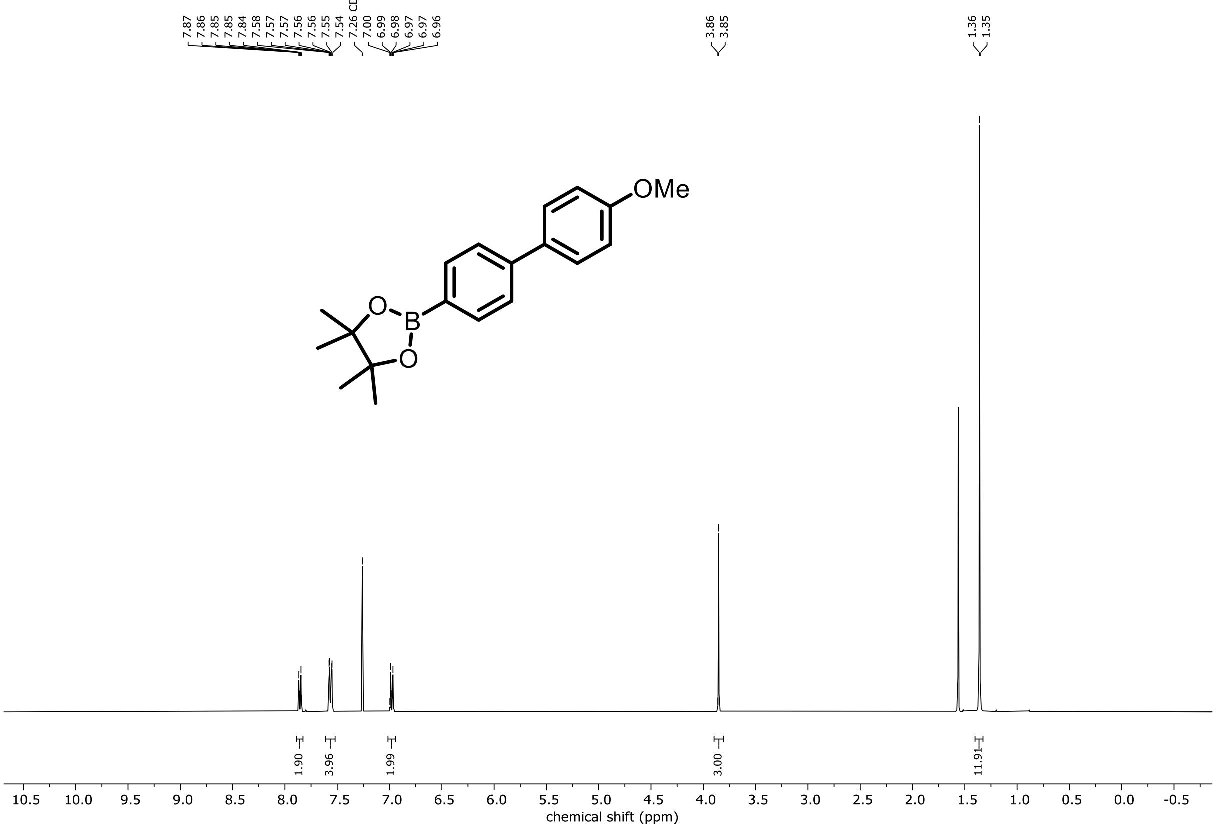

2-(4'-Methoxy-[1,1'-biphenyl]-4-yl)-4,4,5,5-tetramethyl-1,3,2-dioxaborolane (3) (^{13}C NMR, 101 MHz, $CDCl_3$)

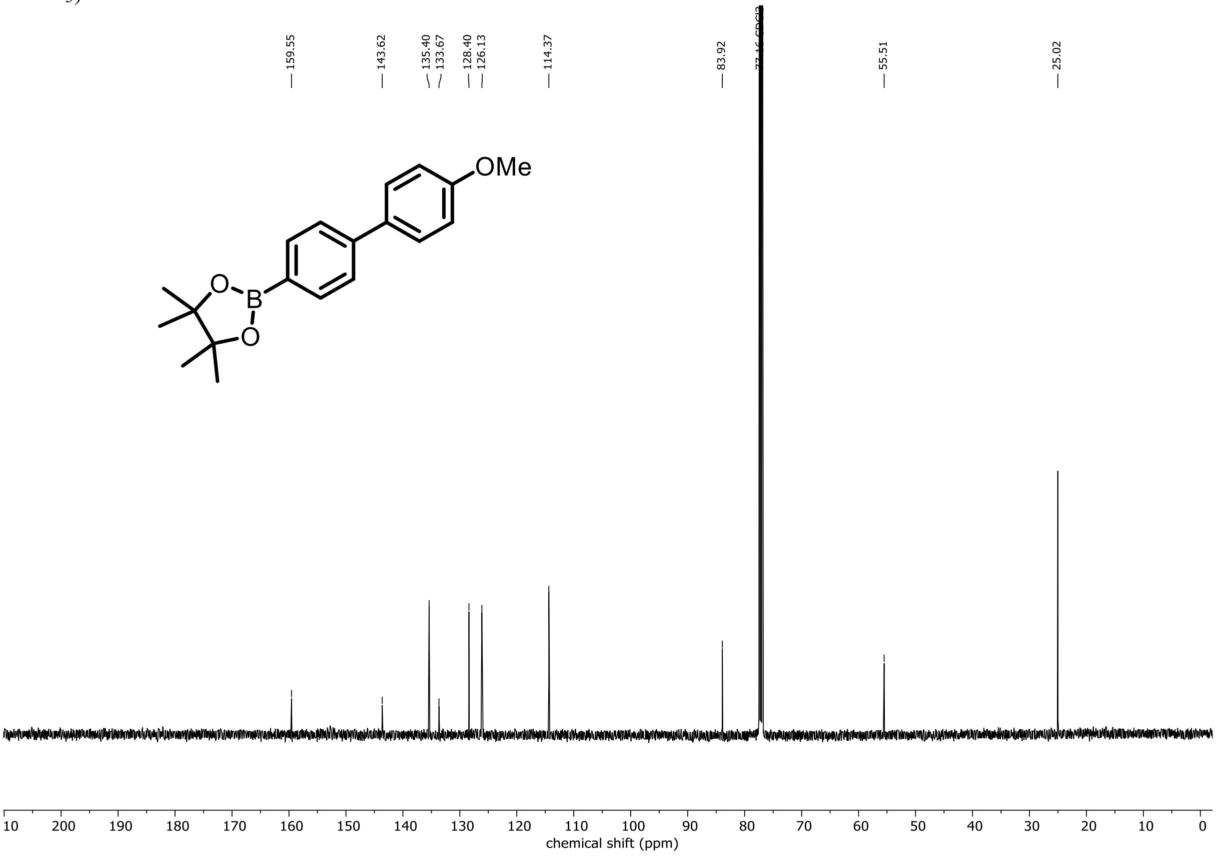

2-(3'-Methoxy-[1,1'-biphenyl]-4-yl)-4,4,5,5-tetramethyl-1,3,2-dioxaborolane (4) (^{1}H NMR, 400 MHz, $CDCl_3$)

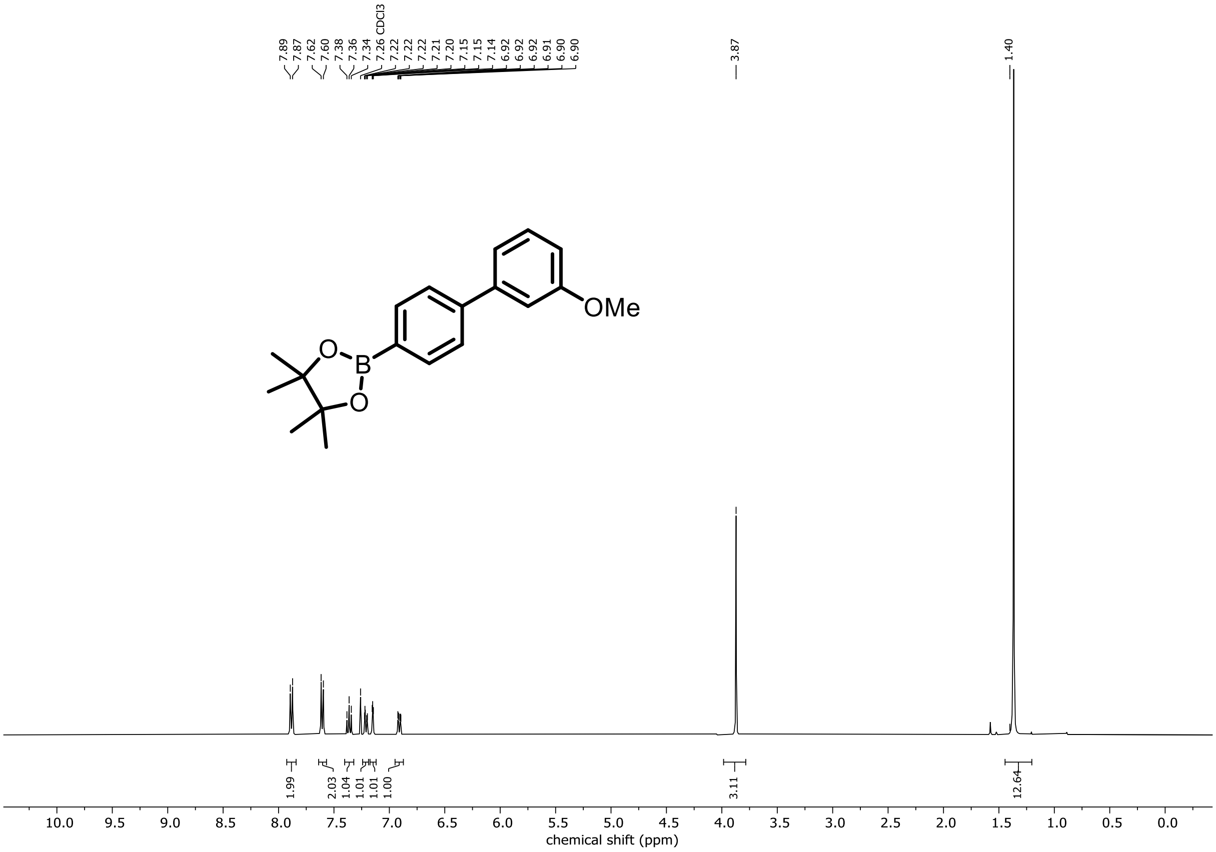

2-(3'-Methoxy-[1,1'-biphenyl]-4-yl)-4,4,5,5-tetramethyl-1,3,2-dioxaborolane (4) (^{13}C NMR, 101 MHz, $CDCl_3$)

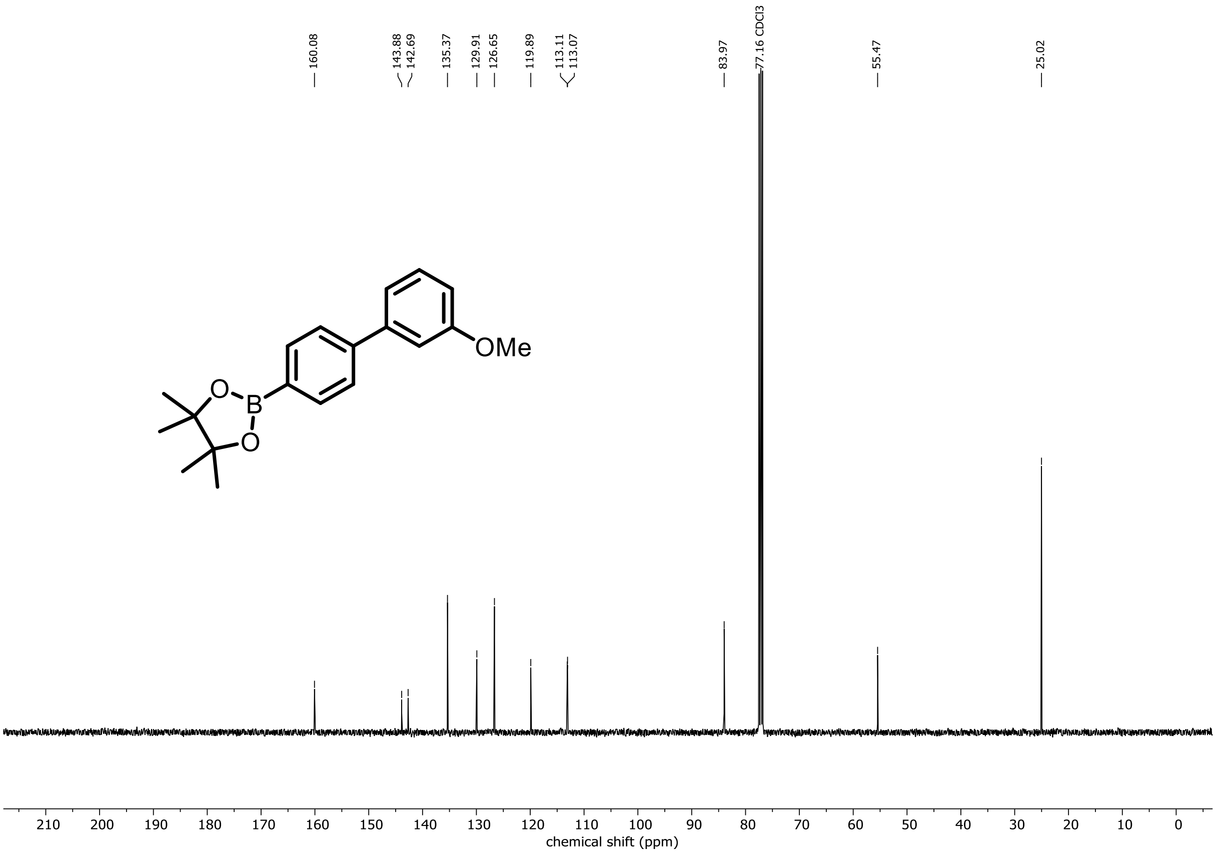

4,4,5,5-Tetramethyl-2-(4'-methyl-[1,1'-biphenyl]-4-yl)-1,3,2-dioxaborolane (5) (^{1}H NMR, 400 MHz, $CDCl_3$)

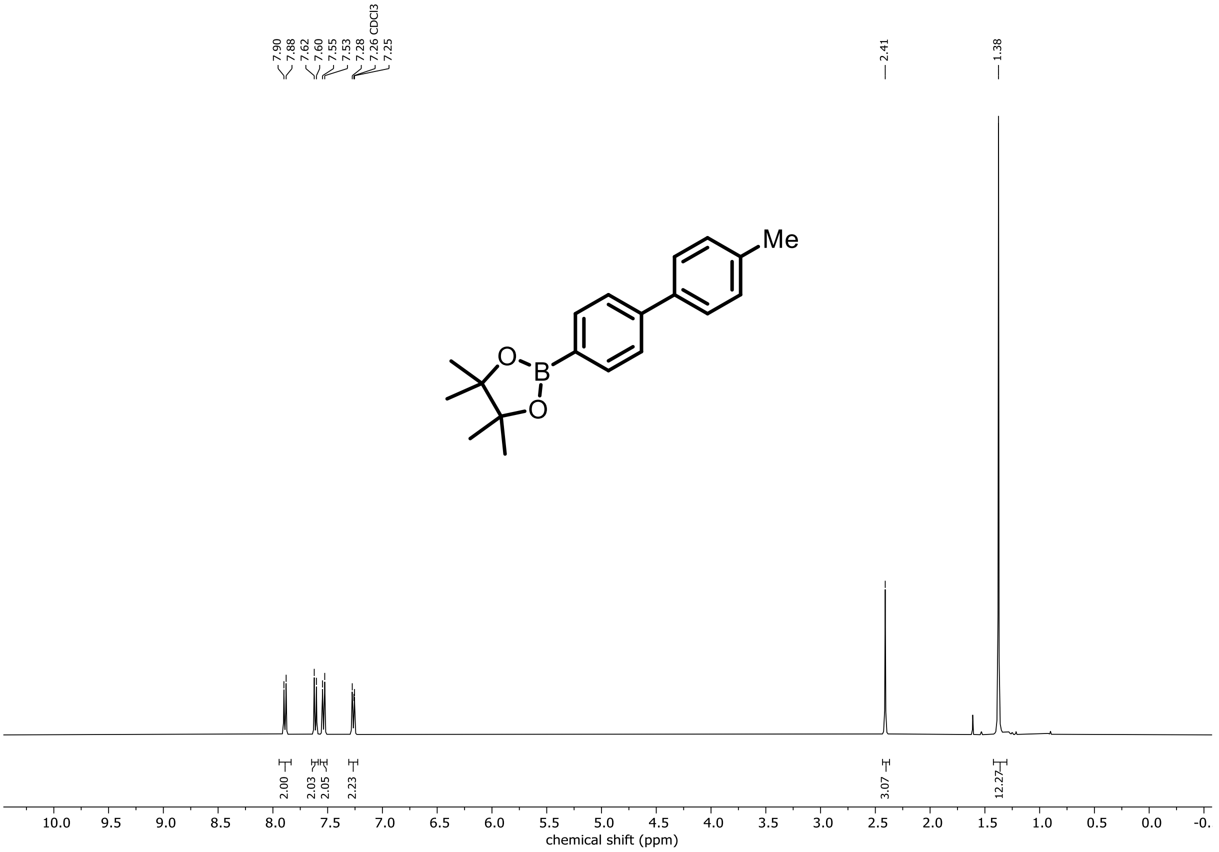

4,4,5,5-Tetramethyl-2-(4'-methyl-[1,1'-biphenyl]-4-yl)-1,3,2-dioxaborolane (5) (^{13}C NMR, 101 MHz, $CDCl_3$)

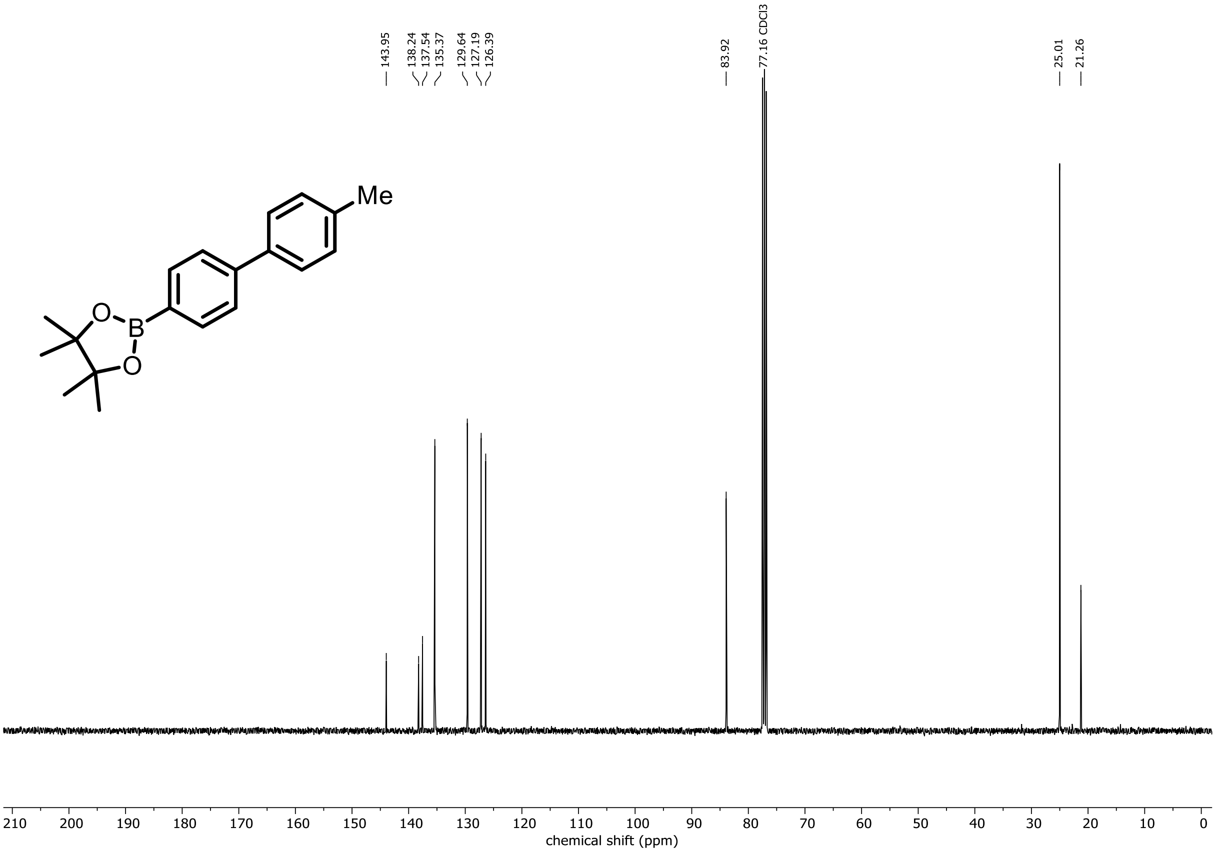

2-(4'-Fluoro-2-methyl-[1,1'-biphenyl]-4-yl)-4,4,5,5-tetramethyl-1,3,2-dioxaborolane (6) (^{1}H NMR, 400 MHz, $CDCl_3$)

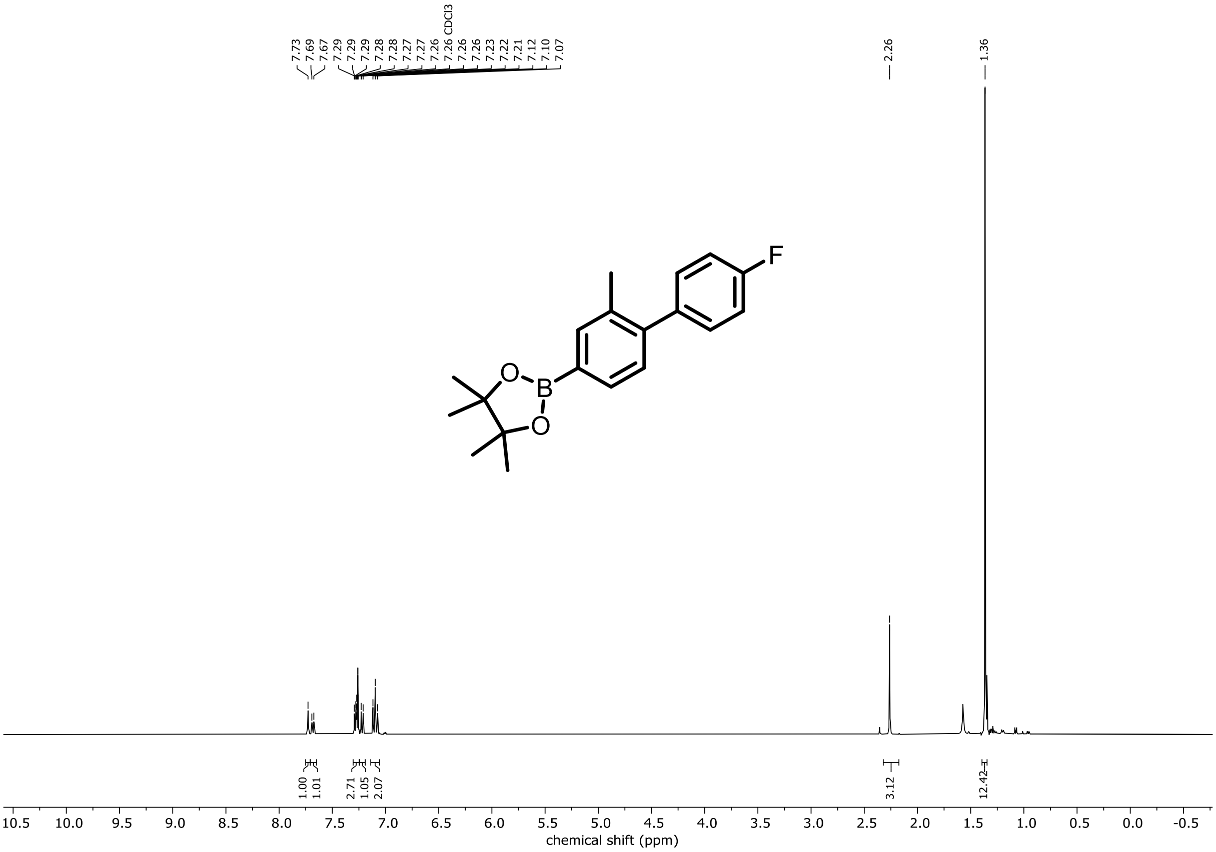

2-(4'-Fluoro-2-methyl-[1,1'-biphenyl]-4-yl)-4,4,5,5-tetramethyl-1,3,2-dioxaborolane (6) (^{13}C NMR, 101 MHz, $CDCl_3$)

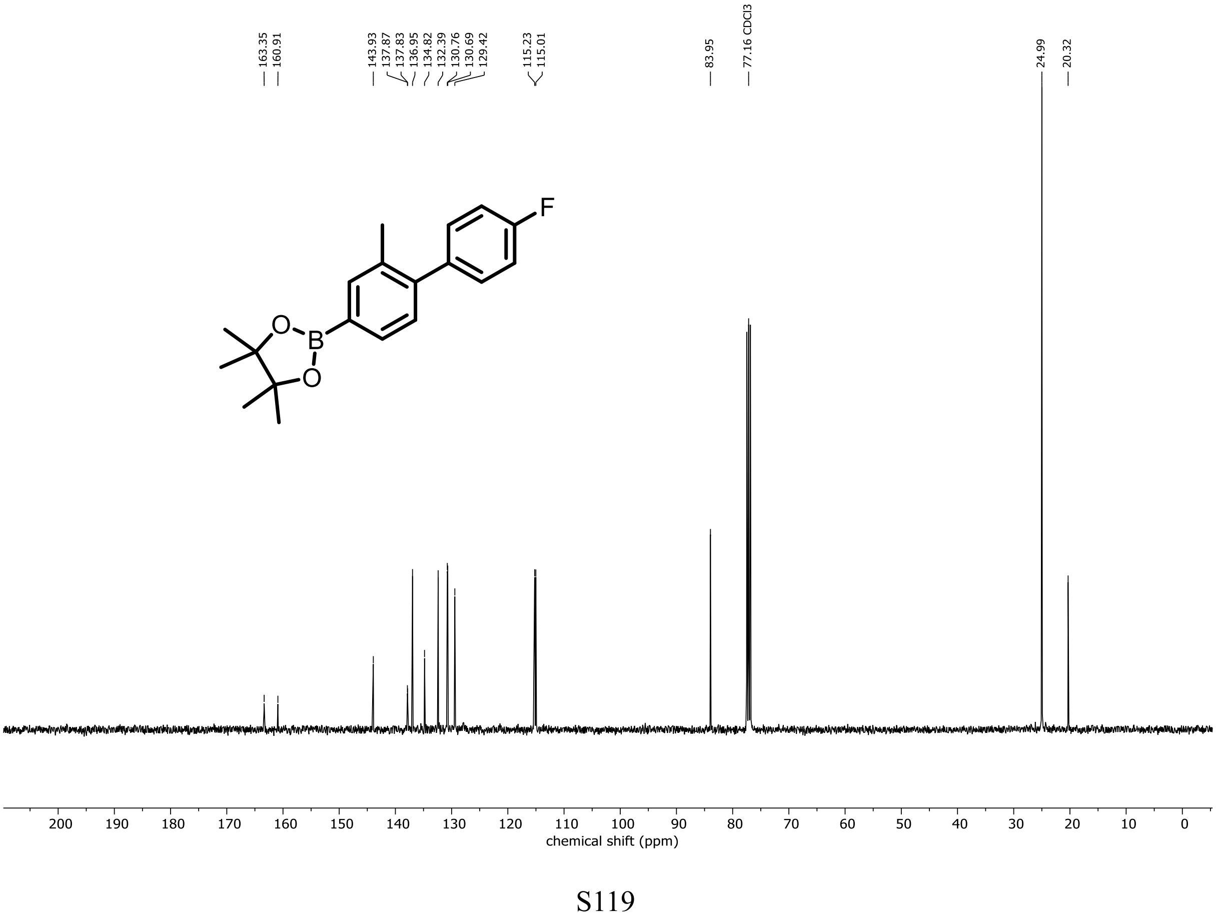

2-(4'-Fluoro-2-methyl-[1,1'-biphenyl]-4-yl)-4,4,5,5-tetramethyl-1,3,2-dioxaborolane (6) (^{19}F NMR, 376 MHz, $CDCl_3$)

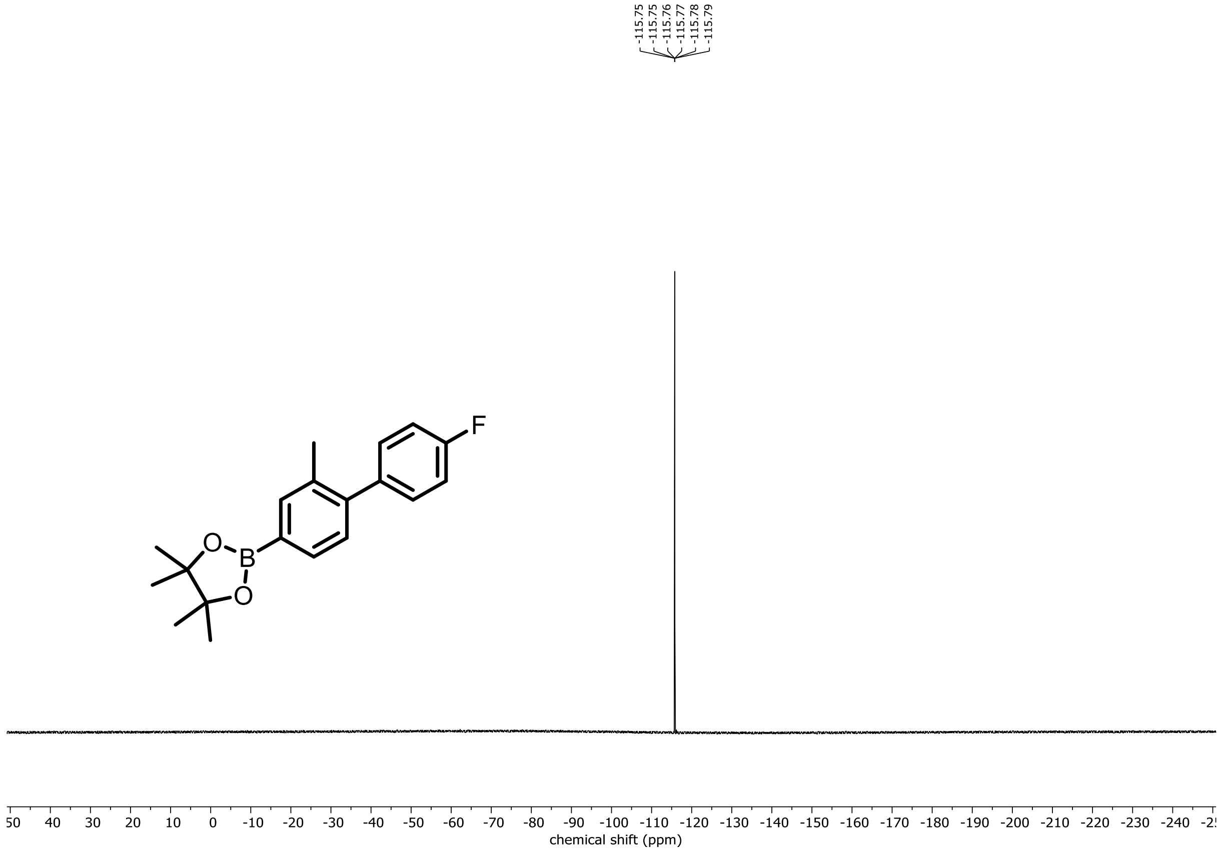

2-(4'-Fluoro-2'-methyl-[1,1'-biphenyl]-4-yl)-4,4,5,5-tetramethyl-1,3,2-dioxaborolane (7) (^{1}H NMR, 400 MHz, $CDCl_3$)

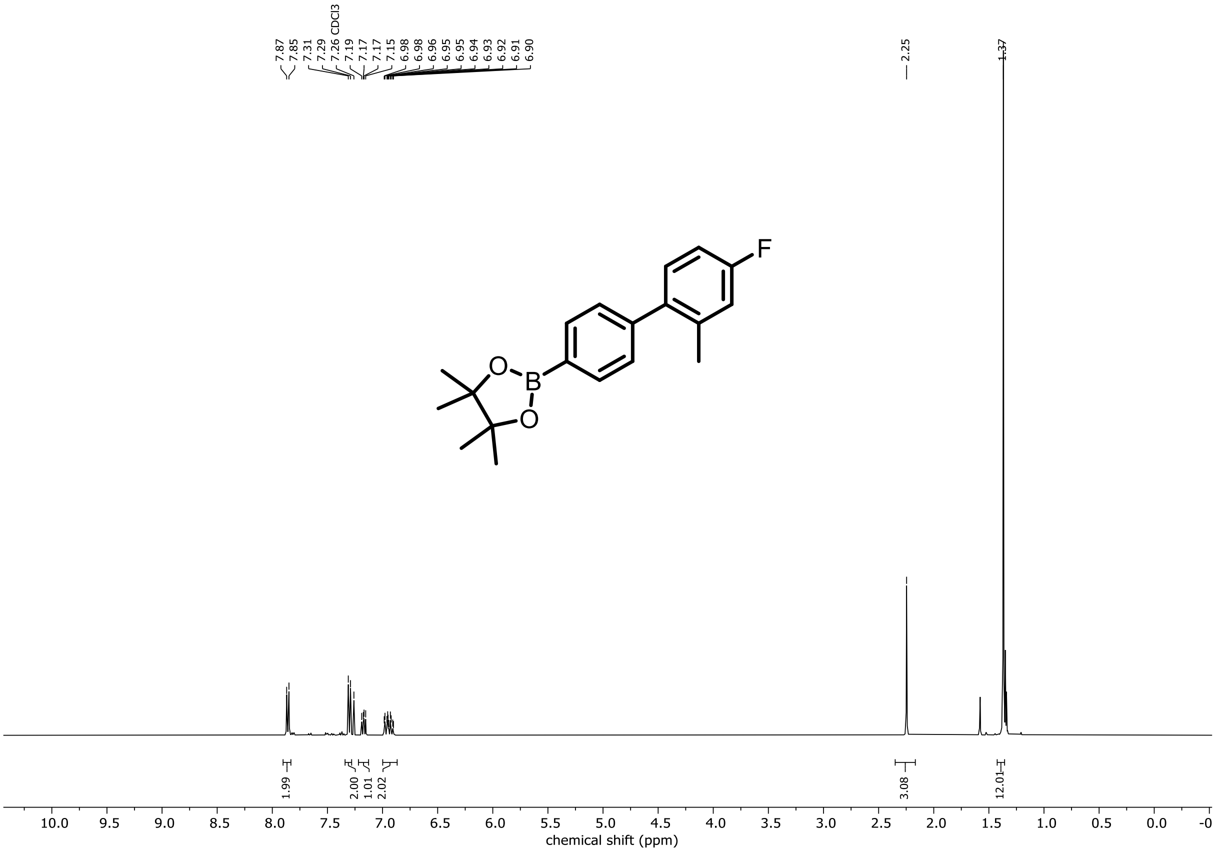

2-(4'-Fluoro-2'-methyl-[1,1'-biphenyl]-4-yl)-4,4,5,5-tetramethyl-1,3,2-dioxaborolane (7) (^{13}C NMR, 101 MHz, $CDCl_3$)

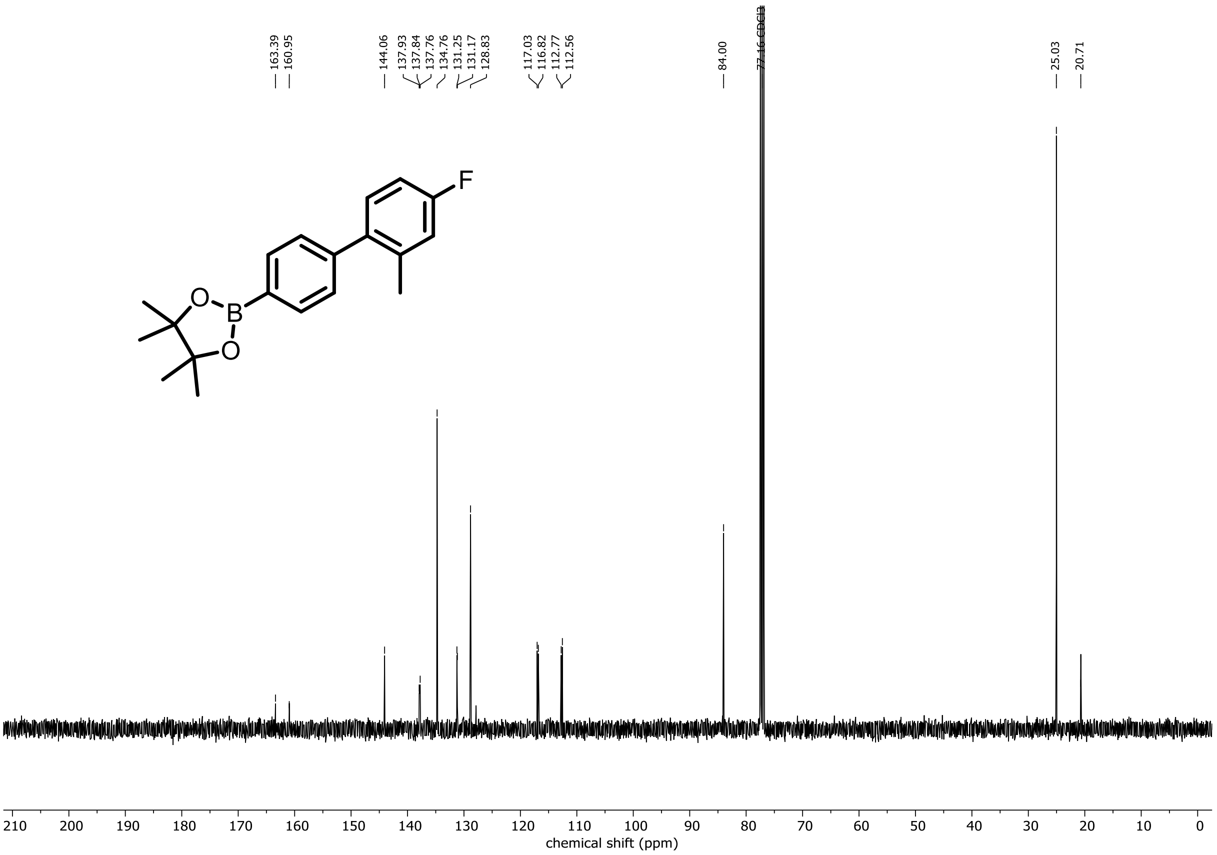

2-(4'-Fluoro-2'-methyl-[1,1'-biphenyl]-4-yl)-4,4,5,5-tetramethyl-1,3,2-dioxaborolane (7) (^{19}F NMR, 376 MHz, $CDCl_3$)

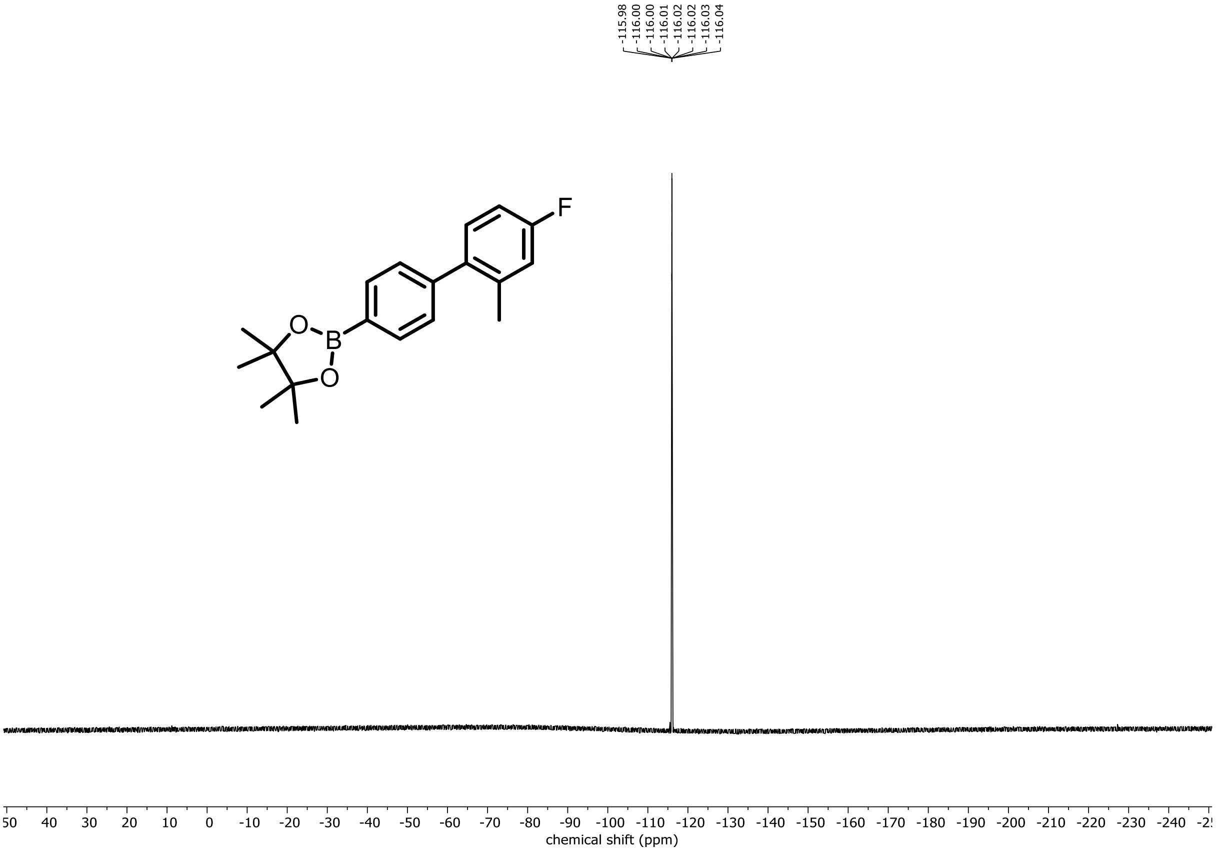

2-(4'-Fluoro-2,2'-dimethyl-[1,1'-biphenyl]-4-yl)-4,4,5,5-tetramethyl-1,3,2-dioxaborolane (8) (^{1}H NMR, 400 MHz, $CDCl_3$)

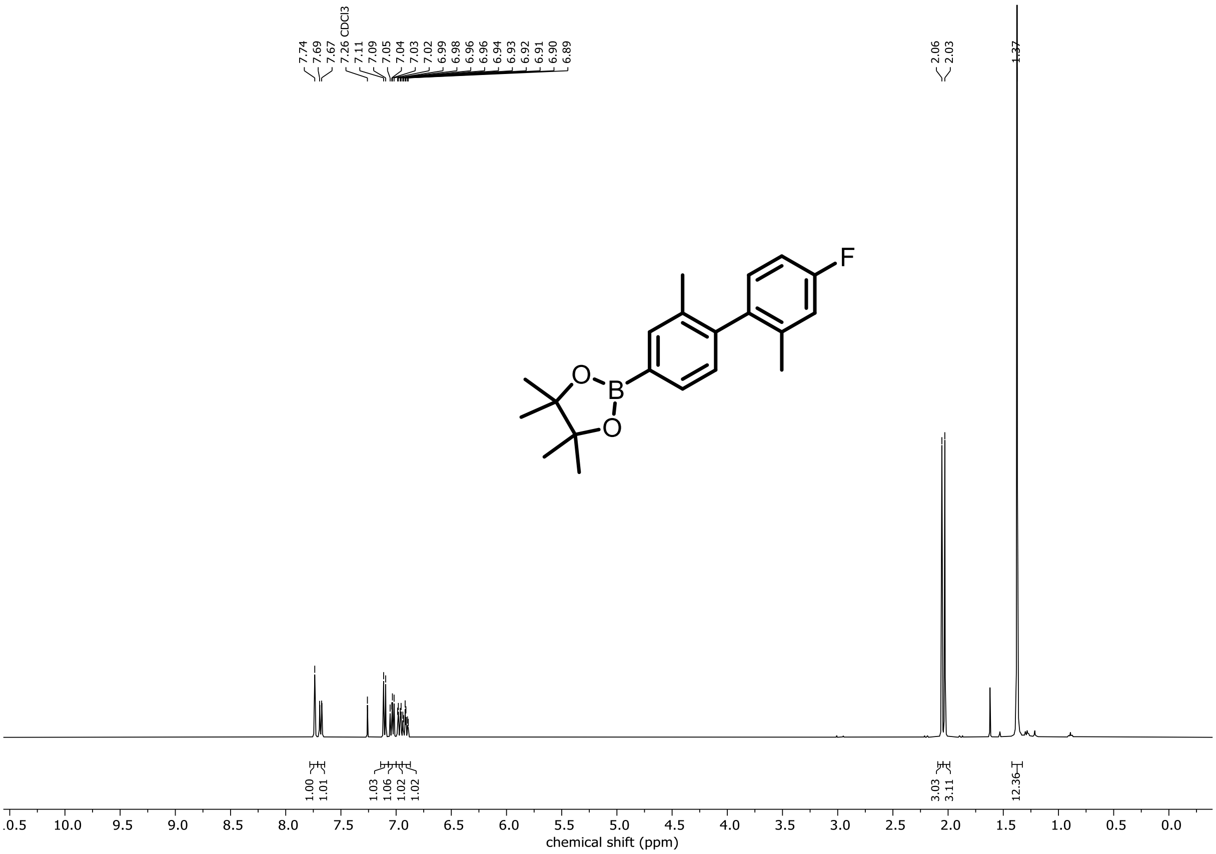

2-(4'-Fluoro-2,2'-dimethyl-[1,1'-biphenyl]-4-yl)-4,4,5,5-tetramethyl-1,3,2-dioxaborolane (8) (^{13}C NMR, 101 MHz, $CDCl_3$)

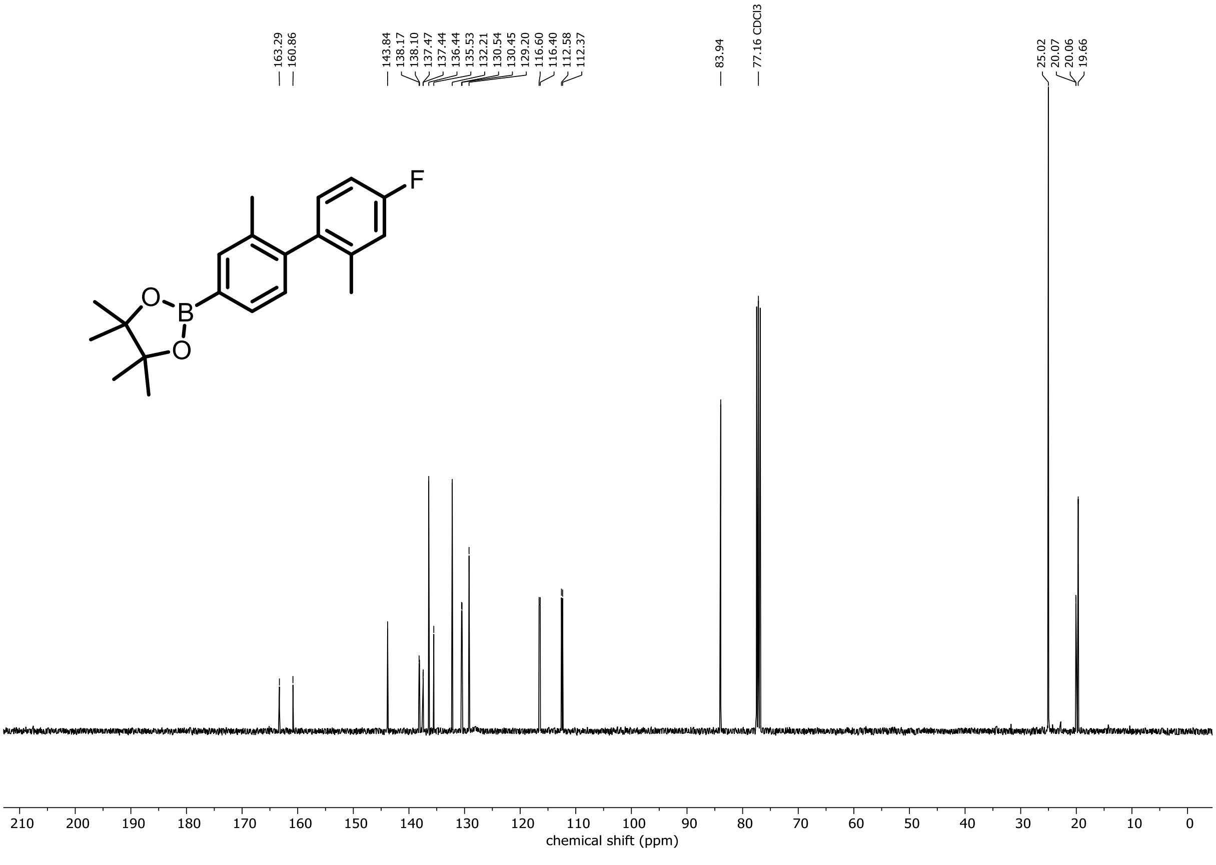

2-(4'-Fluoro-2,2'-dimethyl-[1,1'-biphenyl]-4-yl)-4,4,5,5-tetramethyl-1,3,2-dioxaborolane (8) (^{19}F {^{1}H} NMR, 376 MHz, $CDCl_3$)

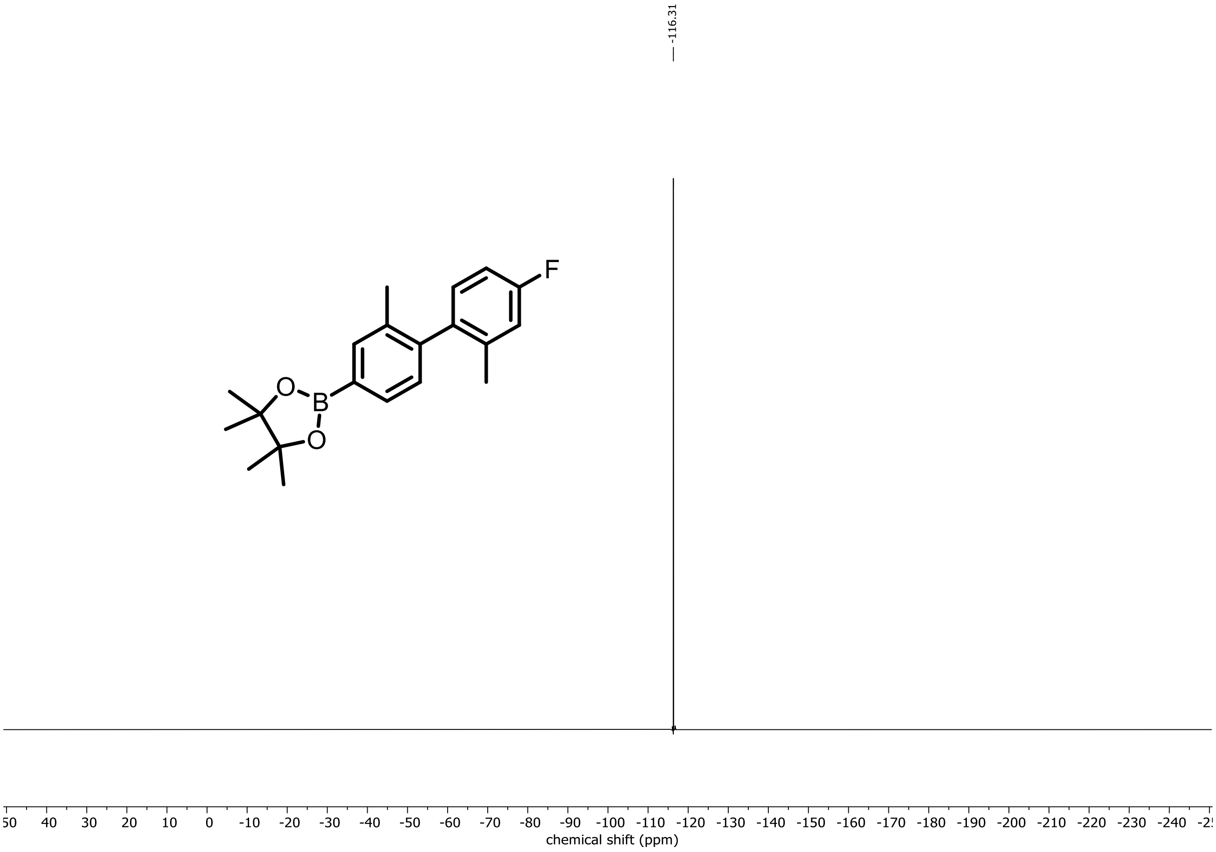

4-Fluorobenzen-2,6-d₂-amine (D₂-S1a) (^{1}H NMR, 400 MHz, $CDCl_3$)

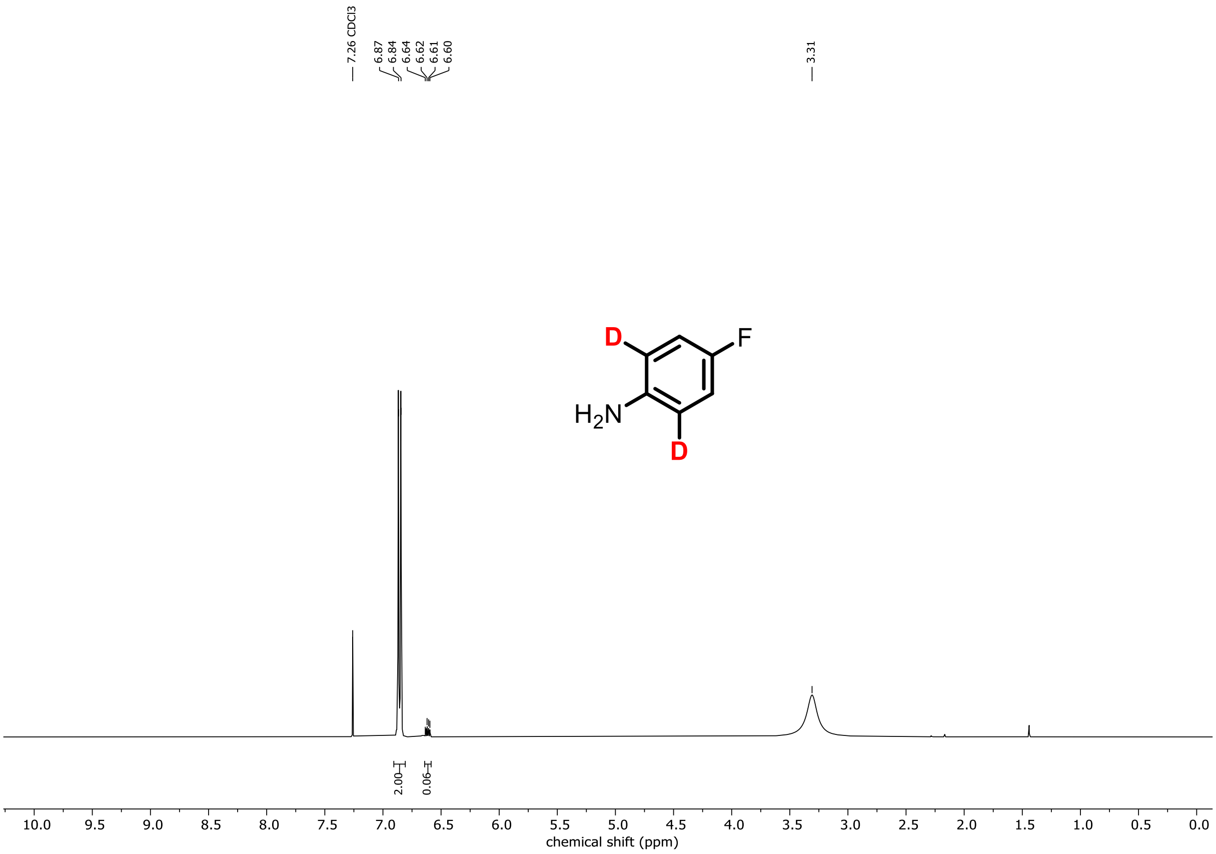

4-Fluorobenzen-2,6-d₂-amine (D₂-S1a) (^{13}C NMR, 101 MHz, $CDCl_3$)

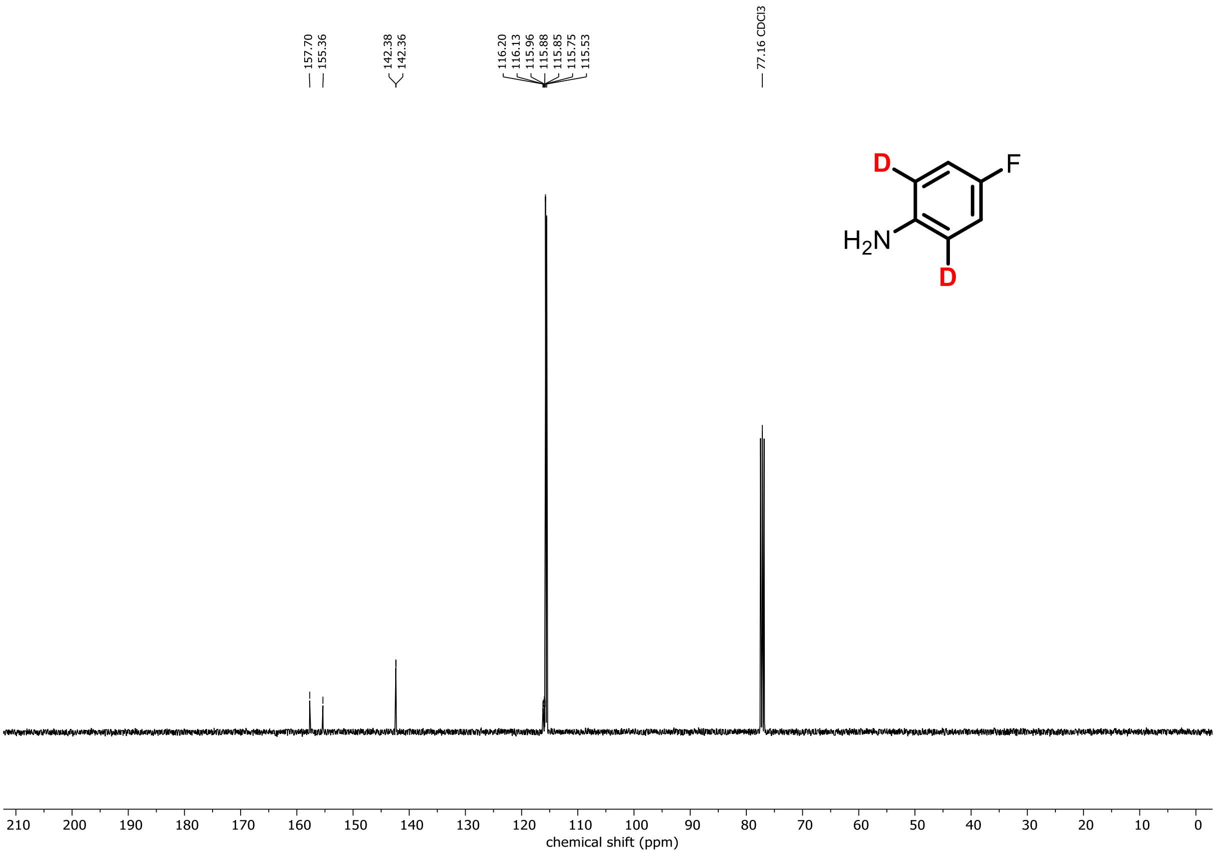

4-Fluorobenzen-2,6-d$_2$-amine (D$_2$-S1a) (^{19}F {^{1}H} NMR, 376 MHz, $CDCl_3$)

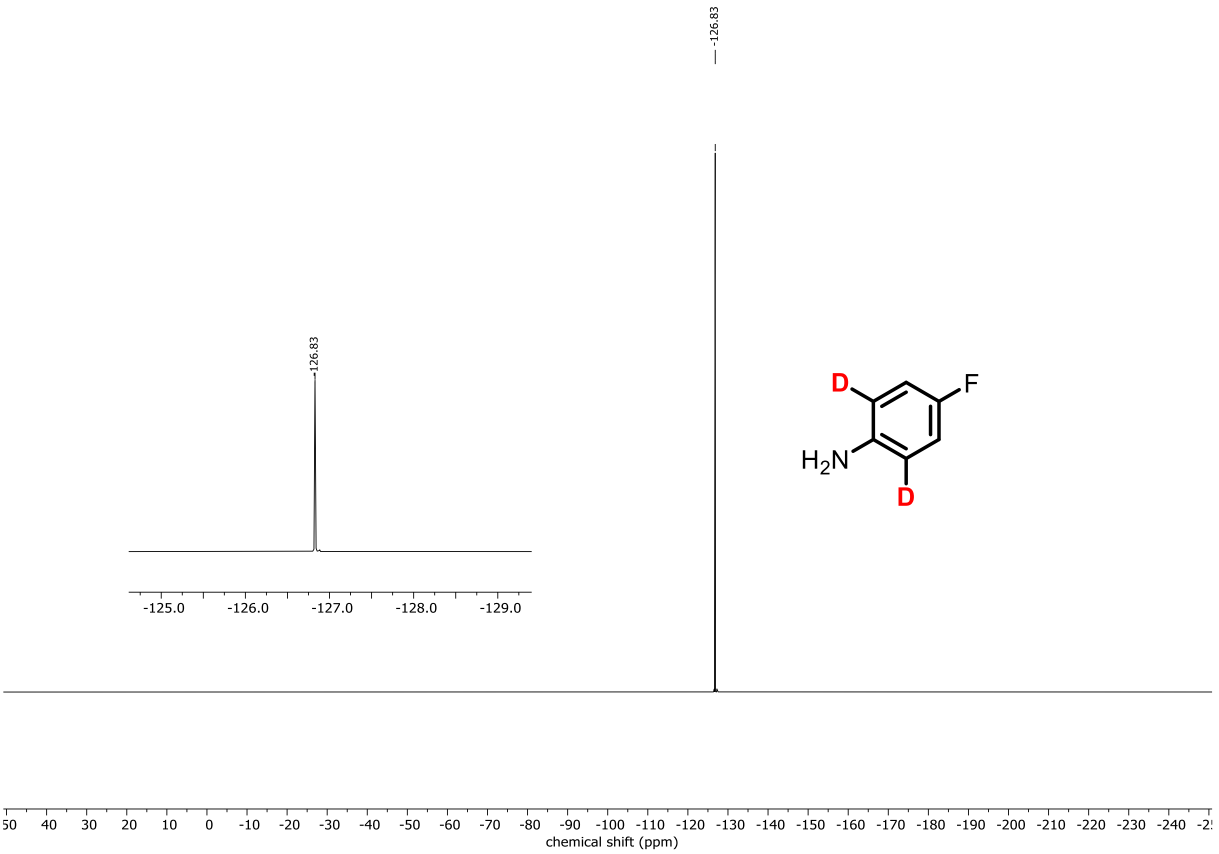

1-Bromo-4-fluorobenzene-2,6-d$_2$ (D$_2$-S1b) (^{1}H NMR, 400 MHz, $CDCl_3$)

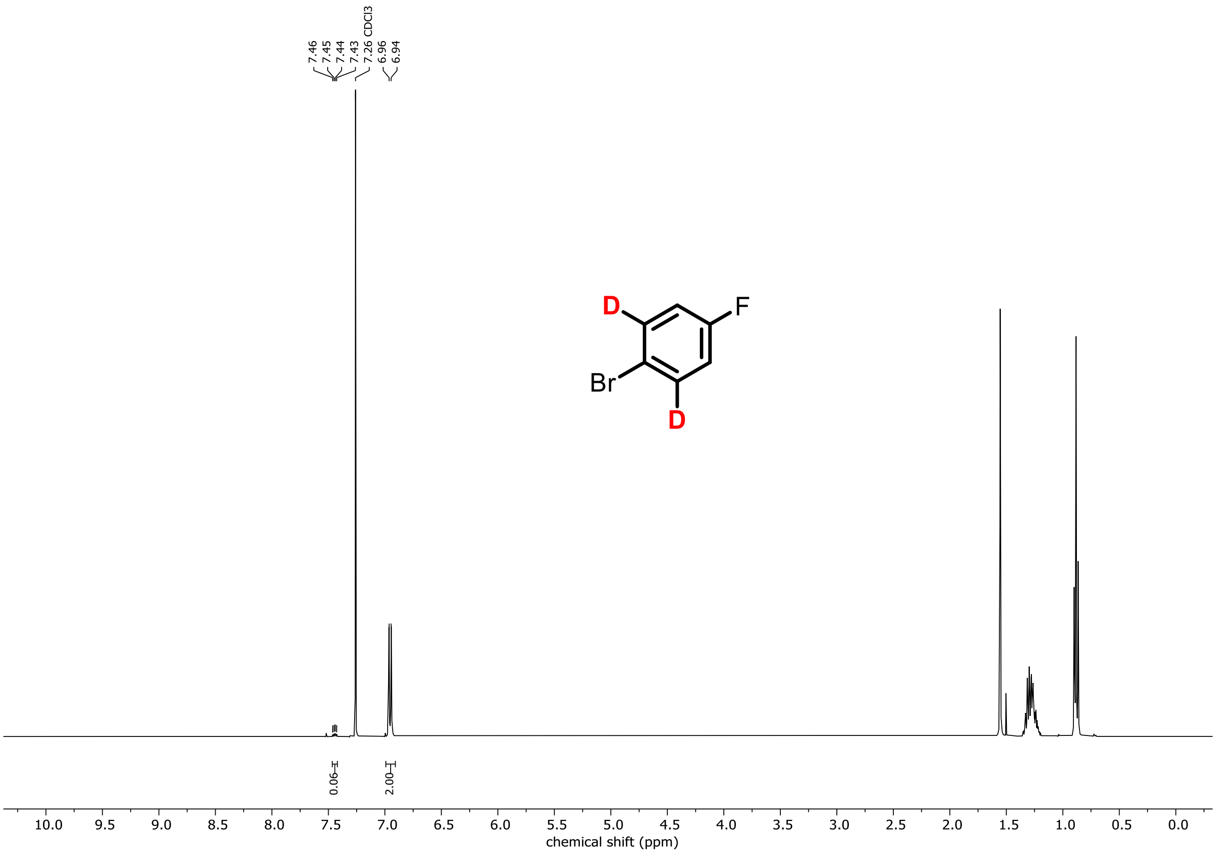

1-Bromo-4-fluorobenzene-2,6-d$_2$ (D$_2$-S1b) (^{19}F {^{1}H} NMR, 376 MHz, $CDCl_3$)

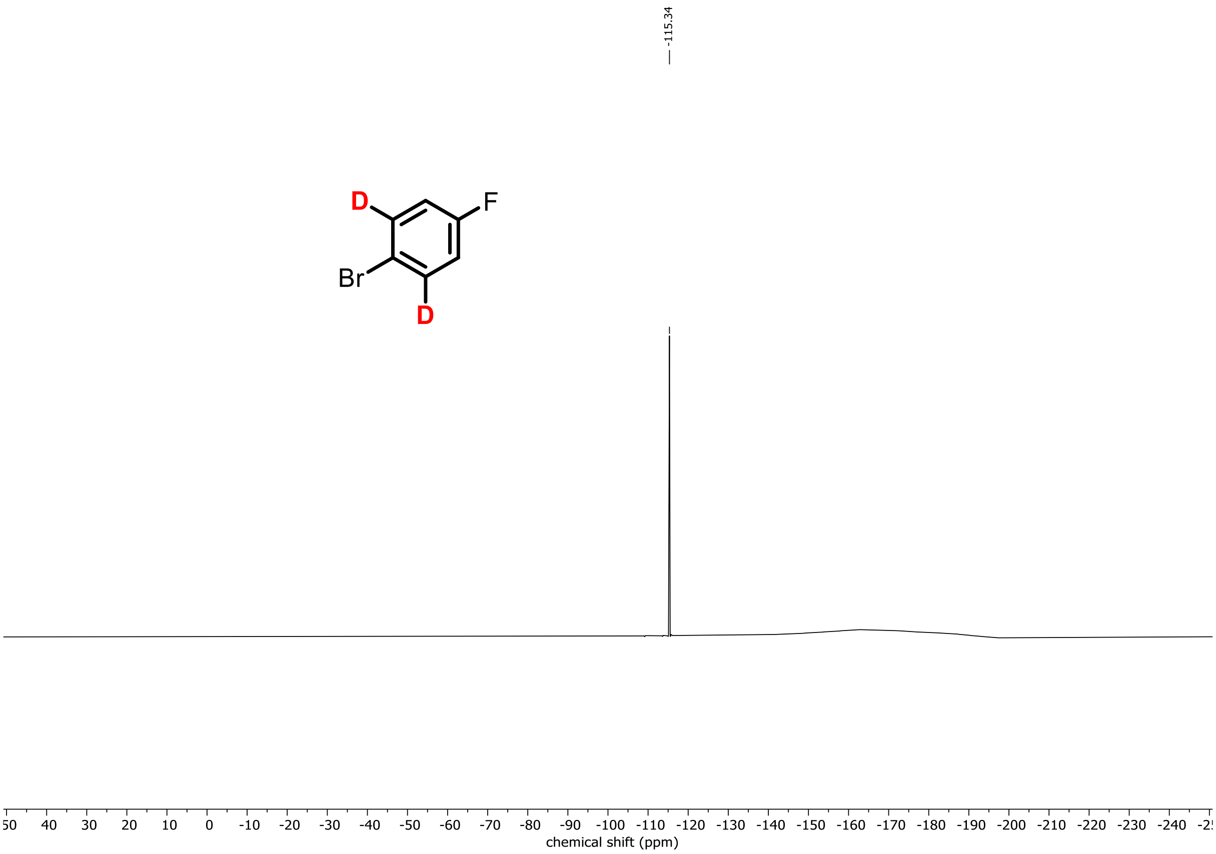

(4-Fluorophenyl-2,6-d$_2$)boronic acid (D$_2$-S1c) (^{1}H NMR, 400 MHz, $(CD_3)_2SO$)

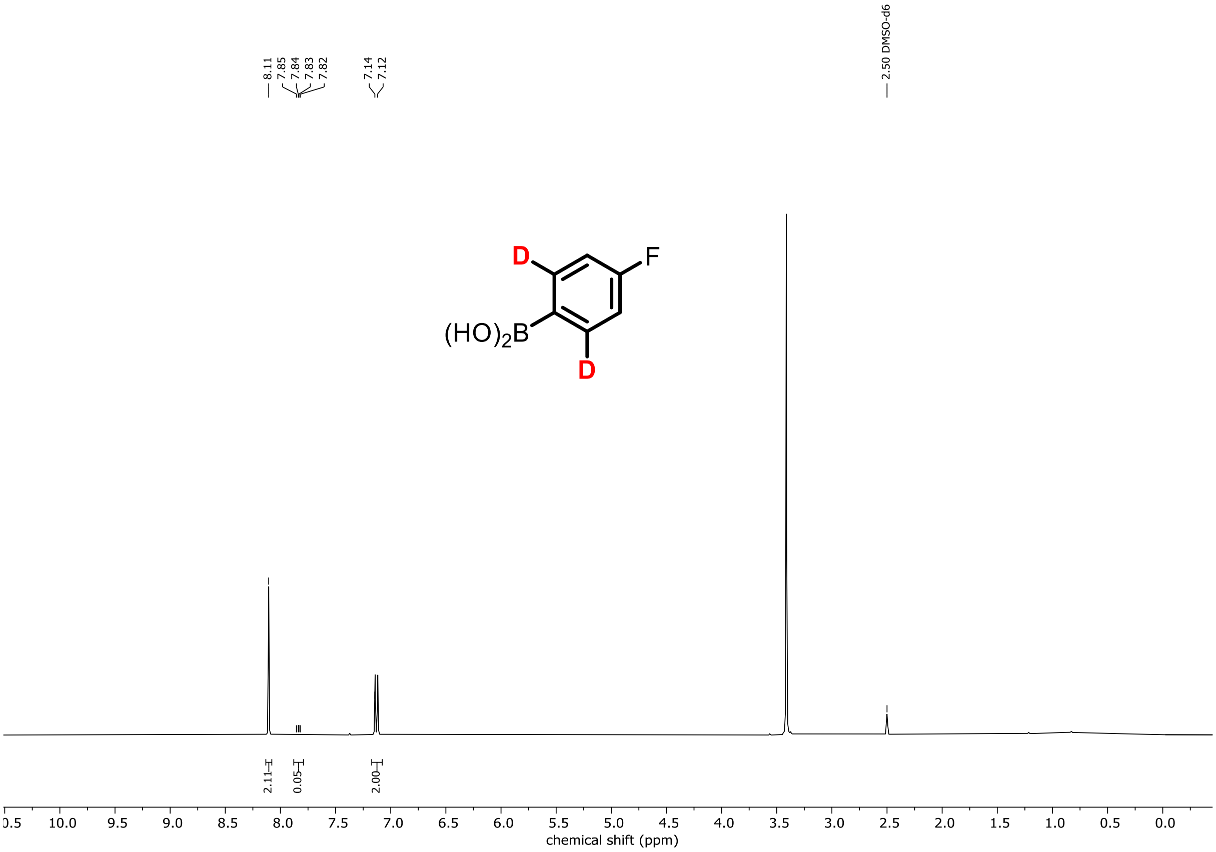

(4-Fluorophenyl-2,6-d$_2$)boronic acid (D$_2$-S1c) (^{13}C NMR, 101 MHz, $(CD_3)_2SO$)

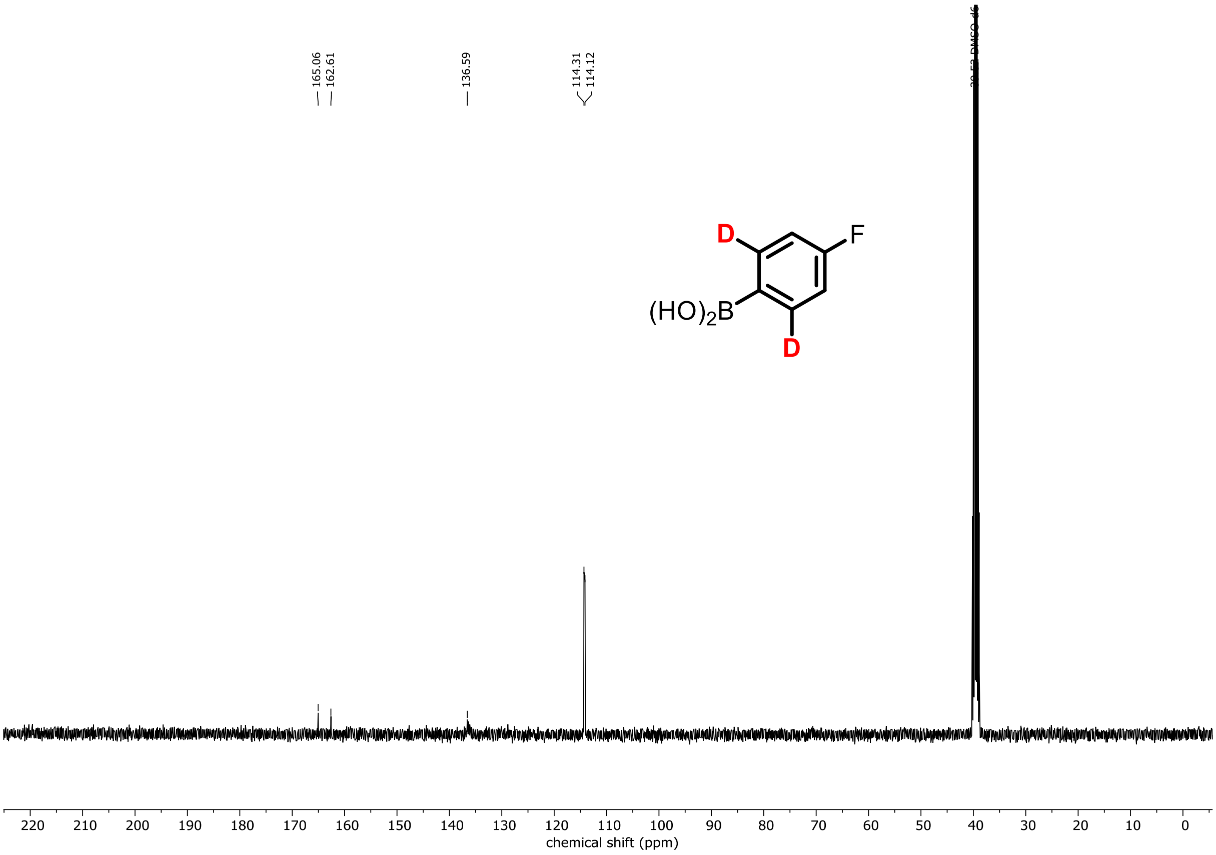

(4-Fluorophenyl-2,6-d$_2$)boronic acid (D$_2$-S1c) (^{19}F NMR, 376 MHz, $(CD_3)_2SO$)

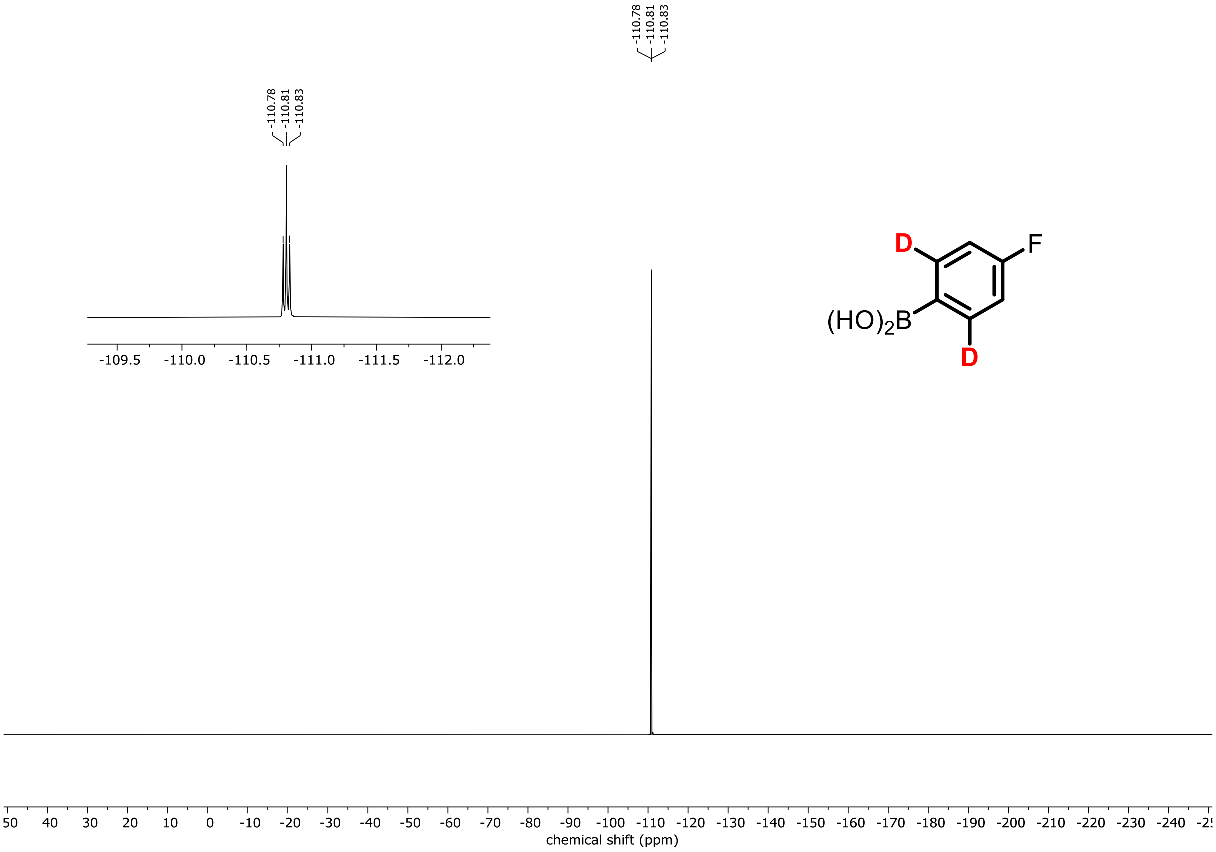

4-Bromo-4'-fluoro-1,1'-biphenyl-2',6'-d₂ (D₂-S1) (^{1}H NMR, 400 MHz, $CDCl_3$)

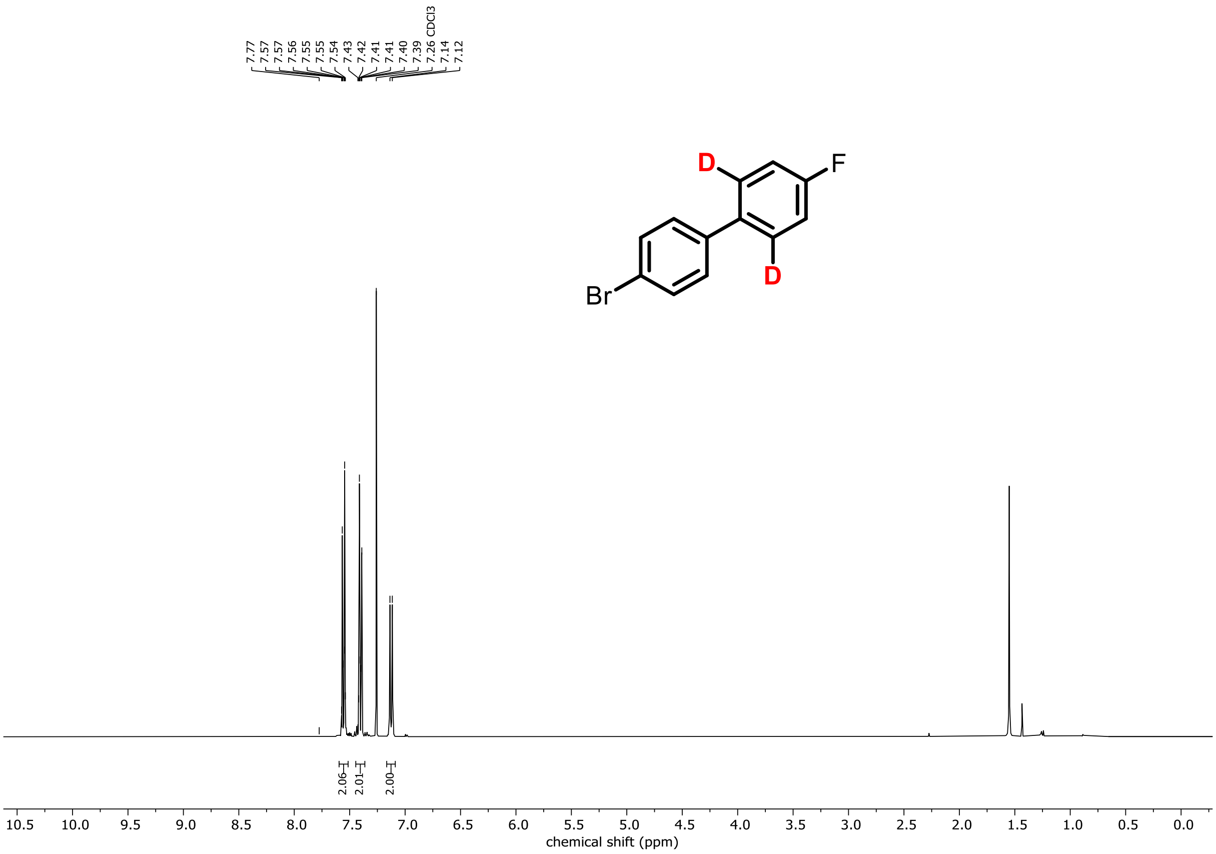

4-Bromo-4'-fluoro-1,1'-biphenyl-2',6'-d₂ (D₂-S1) (^{13}C NMR, 101 MHz, $CDCl_3$)

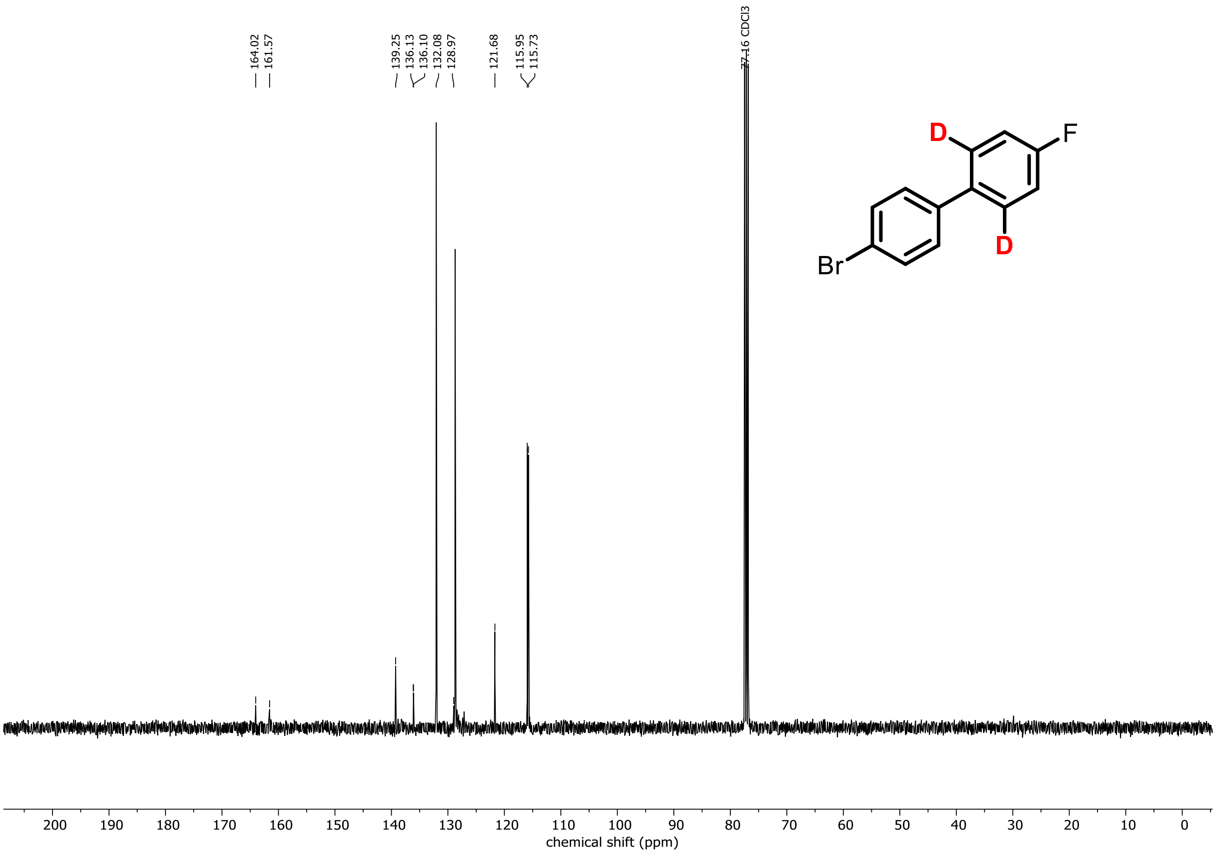

4-Bromo-4'-fluoro-1,1'-biphenyl-2',6'-d$_2$ (D$_2$-S1) (^{19}F {^{1}H} NMR, 376 MHz, $CDCl_3$)

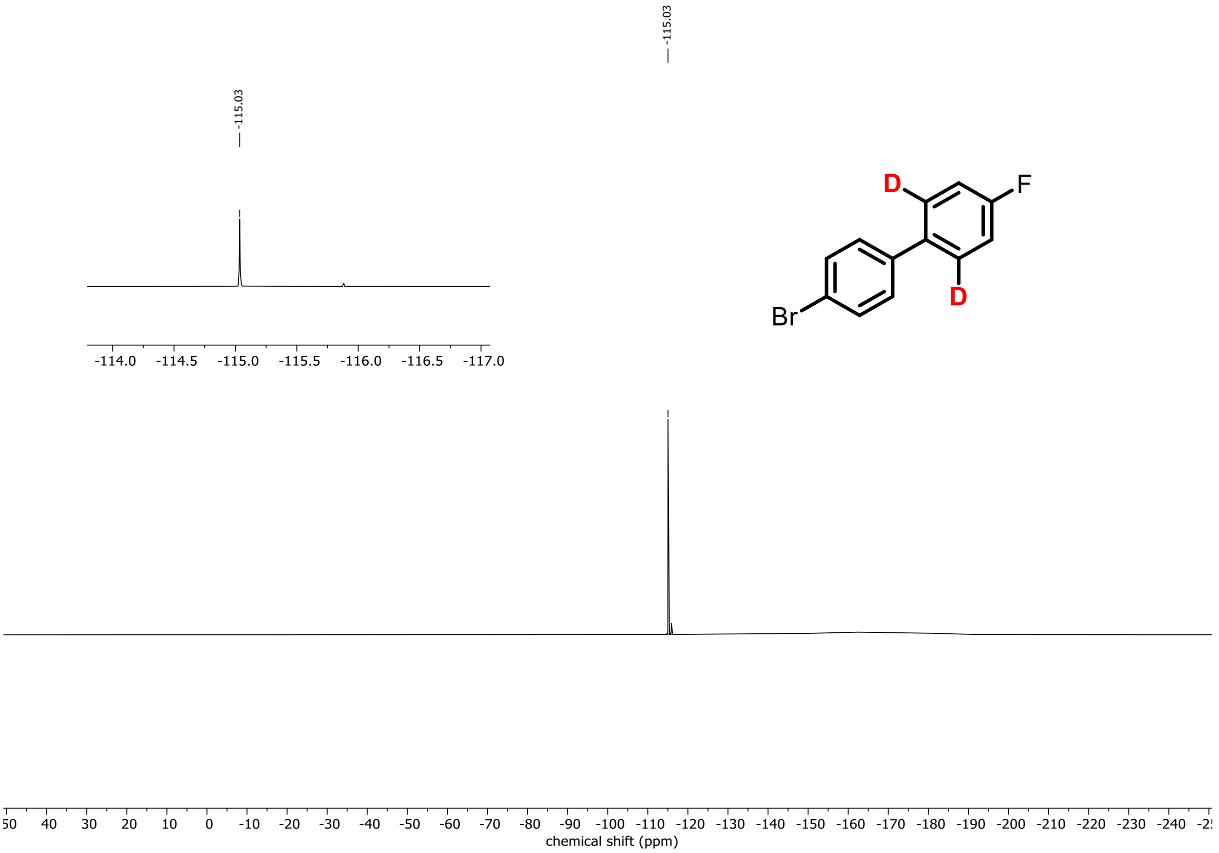

2-(4'-Fluoro-[1,1'-biphenyl]-4-yl-2',6'-d2)-4,4,5,5-tetramethyl-1,3,2-dioxaborolane (D$_2$-1) (^{1}H NMR, 400 MHz, $CDCl_3$)

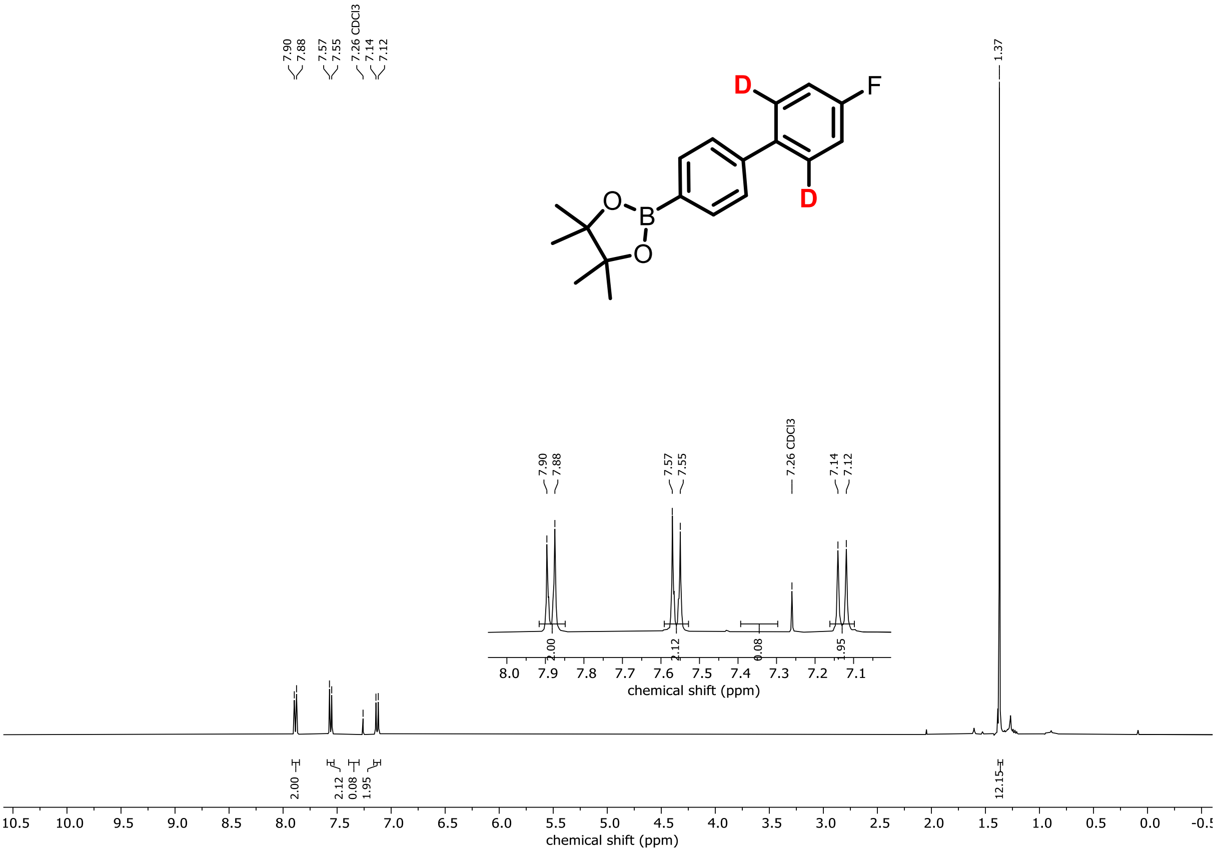

2-(4'-Fluoro-[1,1'-biphenyl]-4-yl-2',6'-d2)-4,4,5,5-tetramethyl-1,3,2-dioxaborolane (D$_2$-1) (^{13}C NMR, 101 MHz, $CDCl_3$)

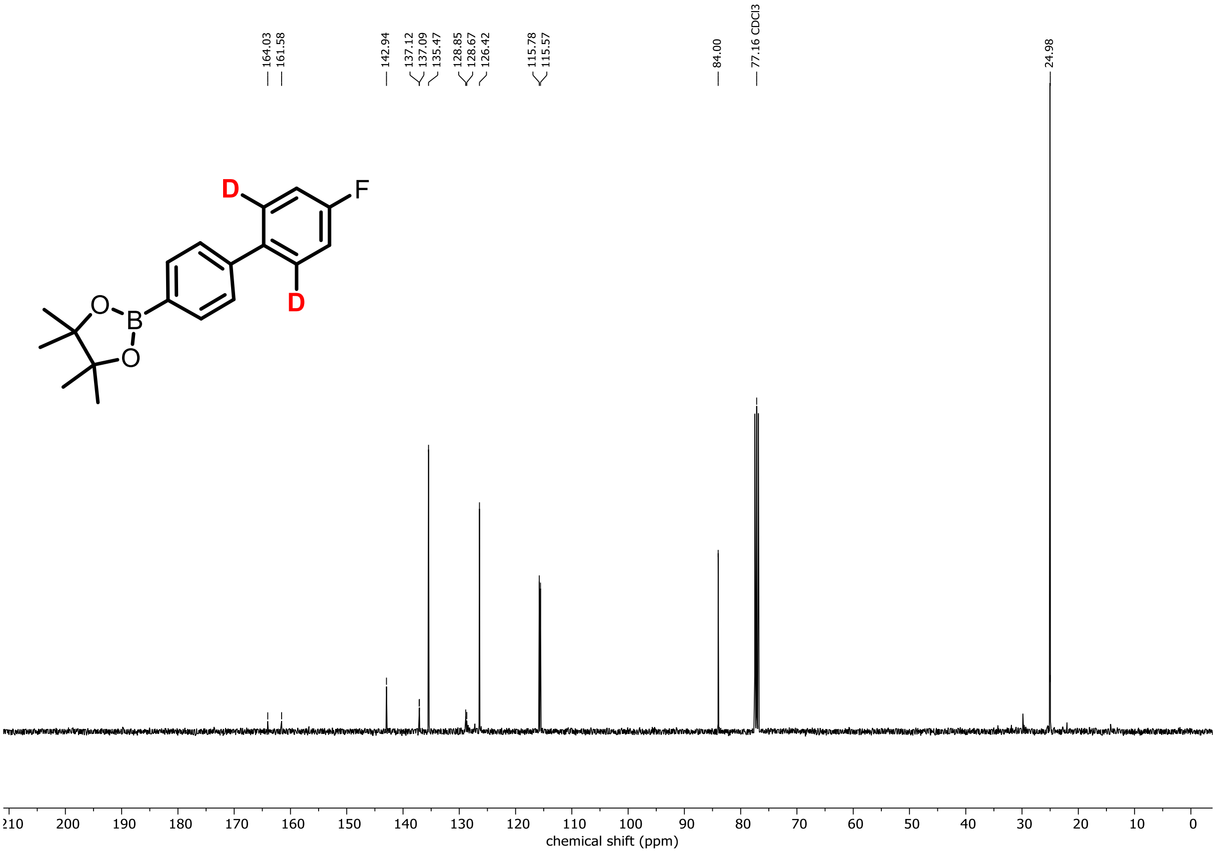

2-(4'-Fluoro-[1,1'-biphenyl]-4-yl-2',6'-d$_2$)-4,4,5,5-tetramethyl-1,3,2-dioxaborolane (D$_2$-1) (^{19}F {^{1}H} NMR, 376 MHz, CDCl$_3$)

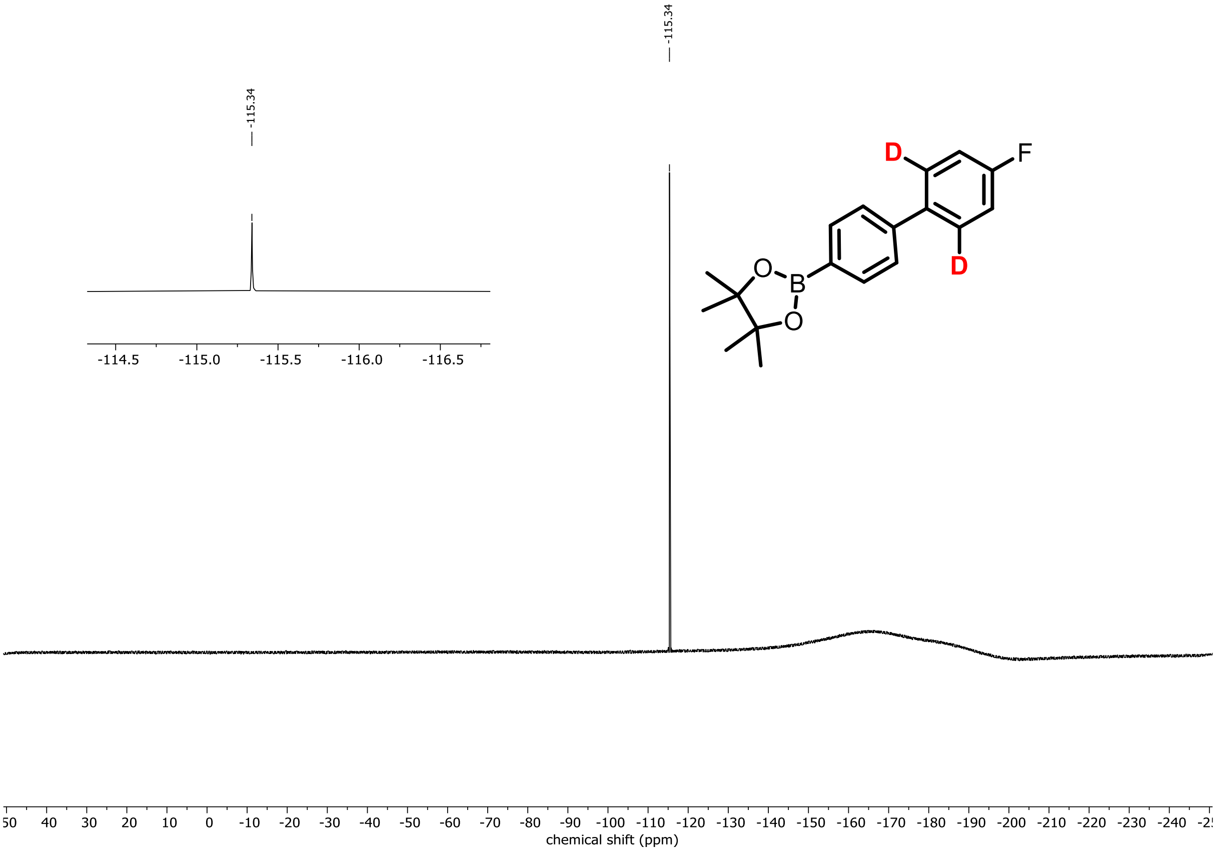

3-Bromo-4'-fluoro-1,1'-biphenyl (S9a) (^{1}H NMR, 400 MHz, $CDCl_3$)

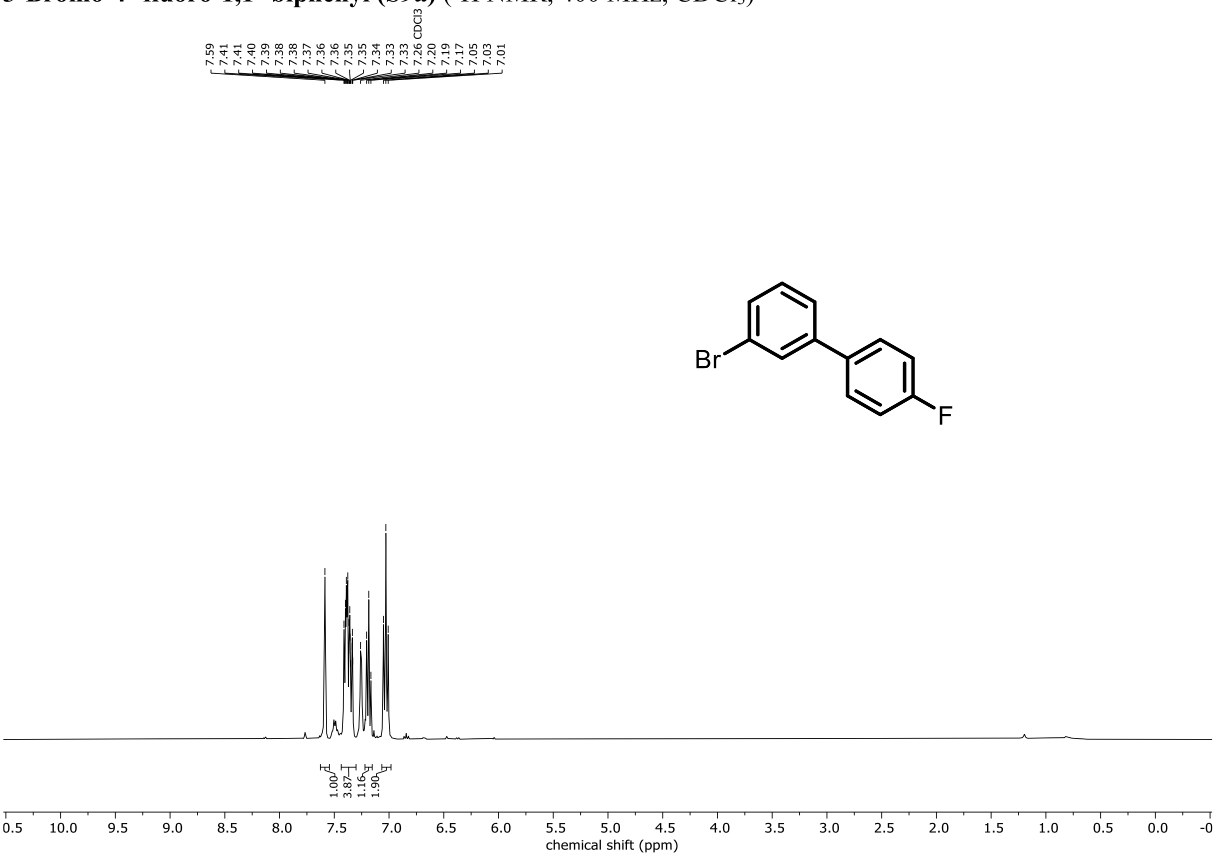

3-Bromo-4'-fluoro-1,1'-biphenyl (S9a) (^{13}C NMR, 101 MHz, $CDCl_3$)

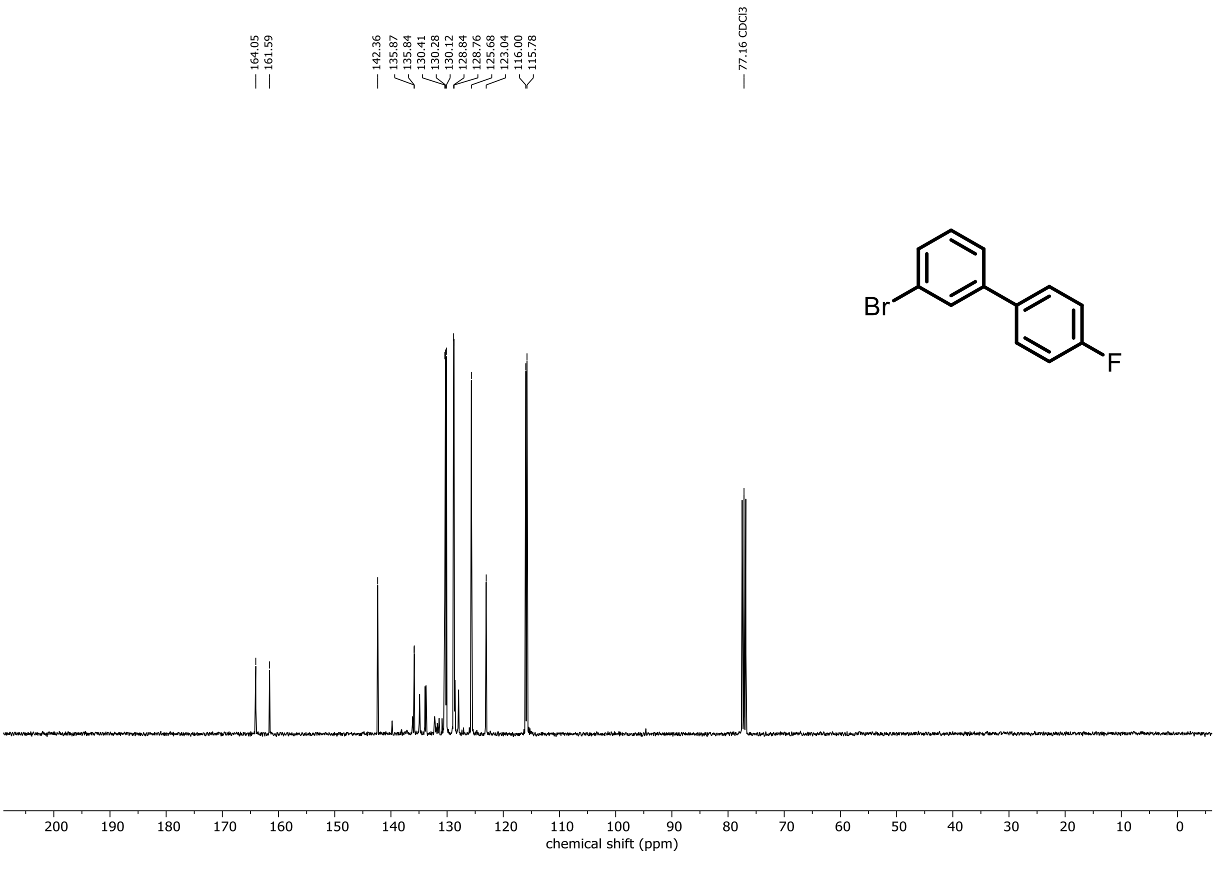

3-Bromo-4'-fluoro-1,1'-biphenyl (S9a) (^{19}F NMR, 377 MHz, $CDCl_3$)

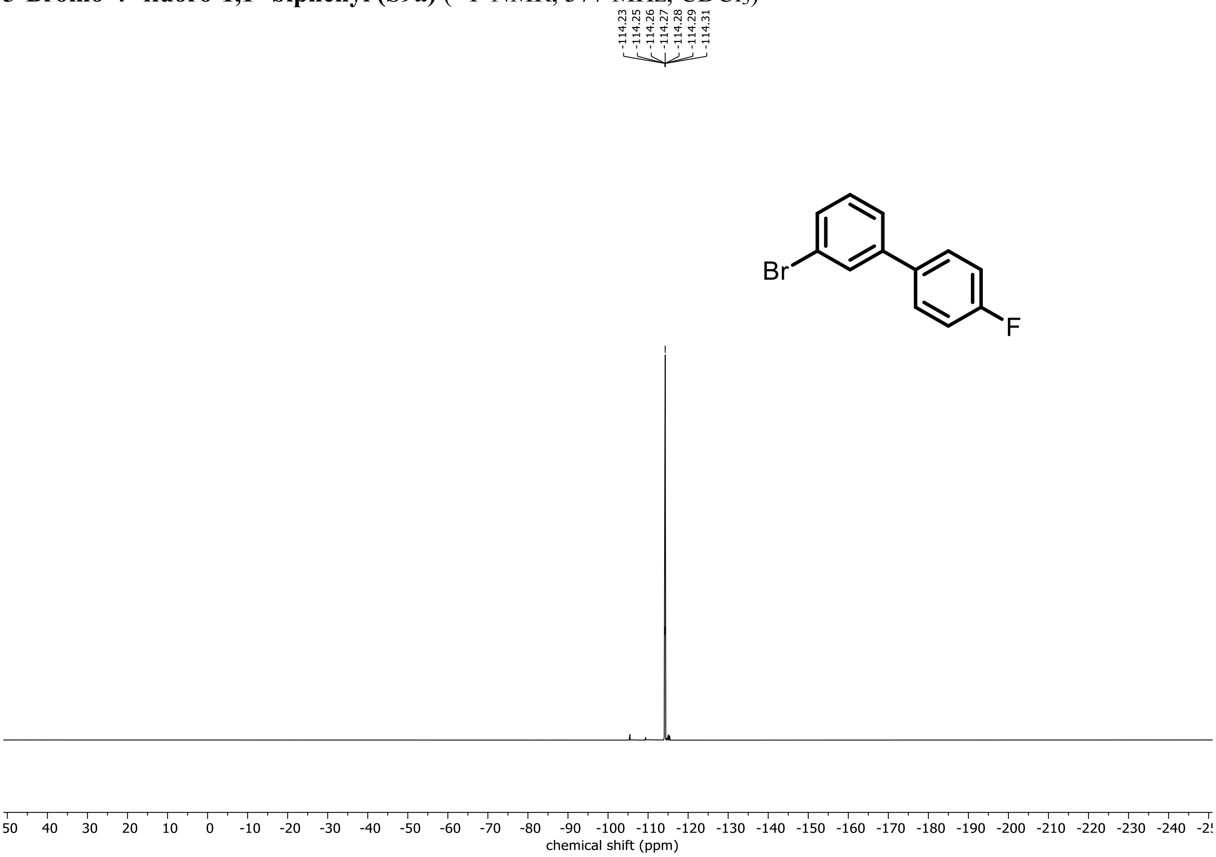

(4'-Fluoro-[1,1'-biphenyl]-3-yl)boronic acid (S9b) (^{1}H NMR, 400 MHz, $(CD_3)_2SO$)

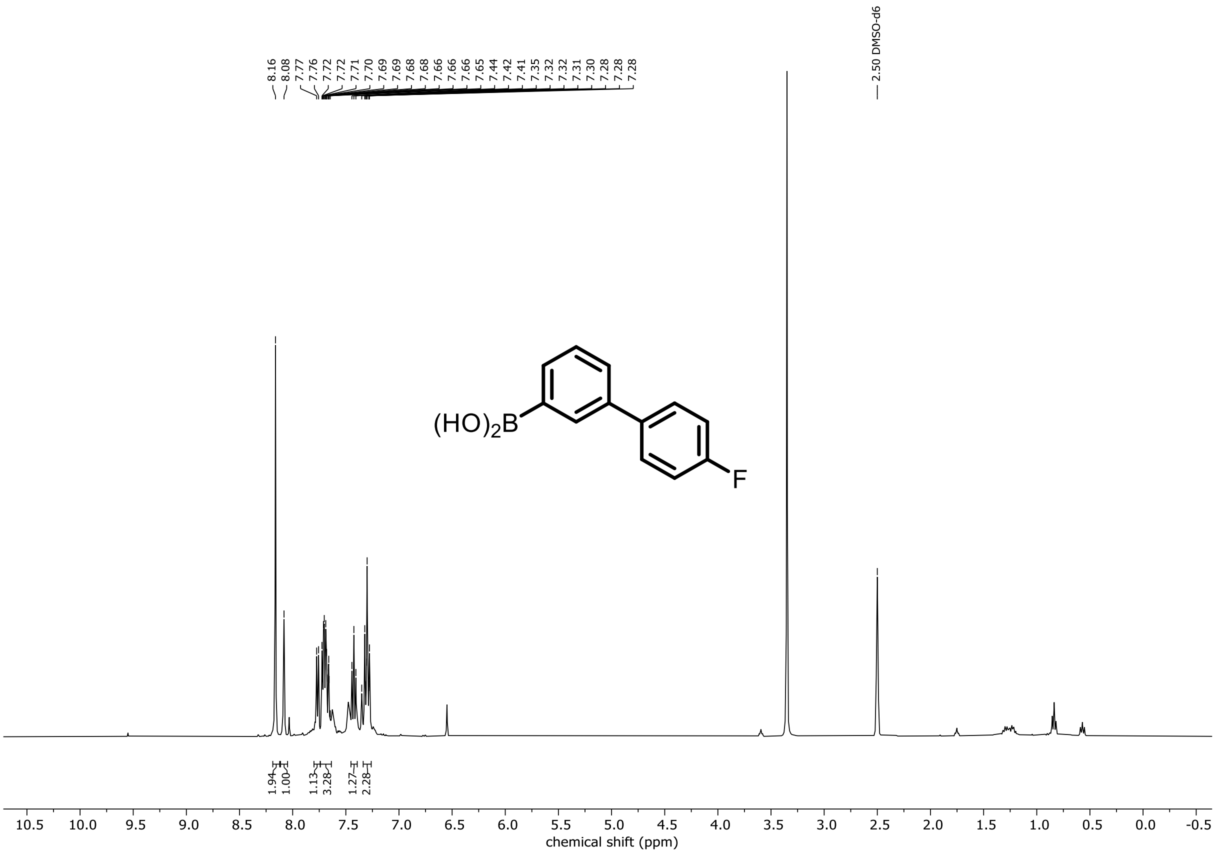

(4'-Fluoro-[1,1'-biphenyl]-3-yl)boronic acid (S9b) (^{13}C NMR, 101 MHz, $(CD_3)_2SO$)

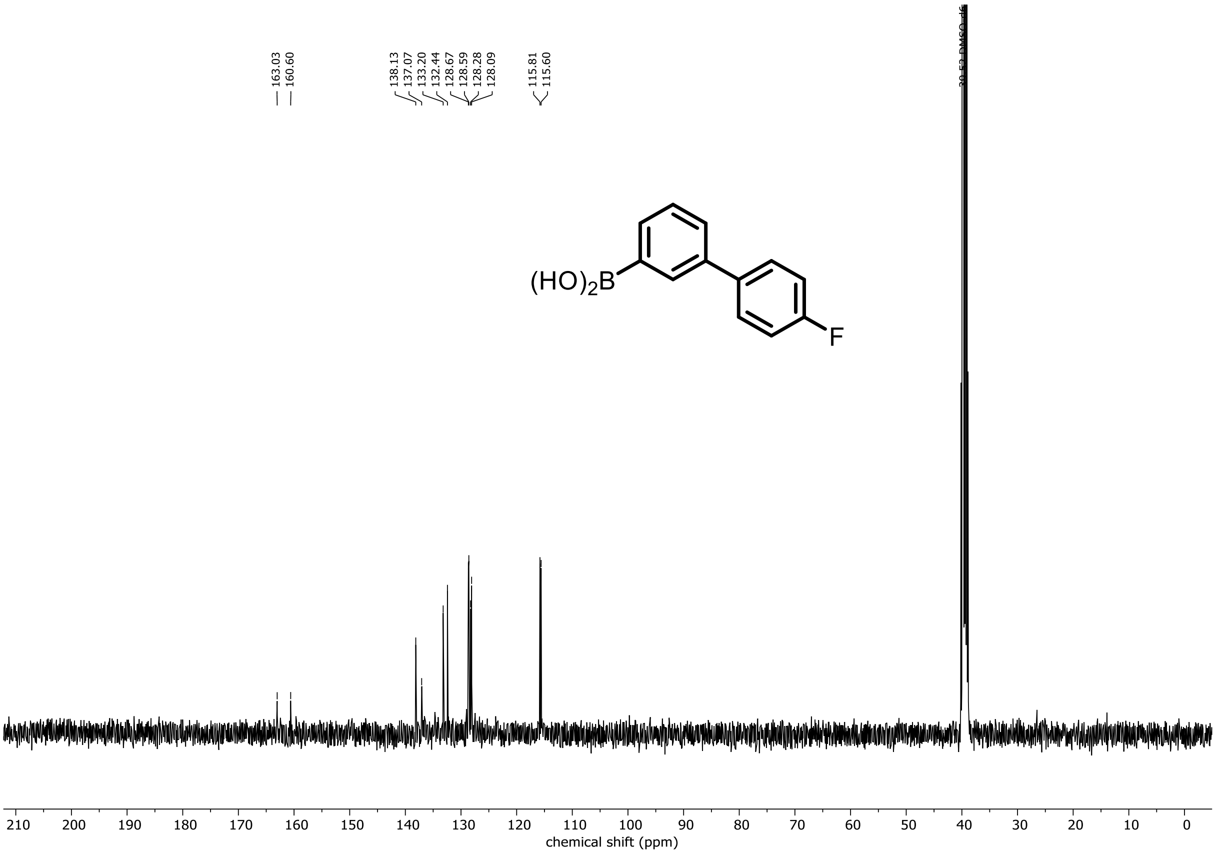

(4'-Fluoro-[1,1'-biphenyl]-3-yl)boronic acid (S9b) (^{19}F NMR, 377 MHz $(CD_3)_2SO$)

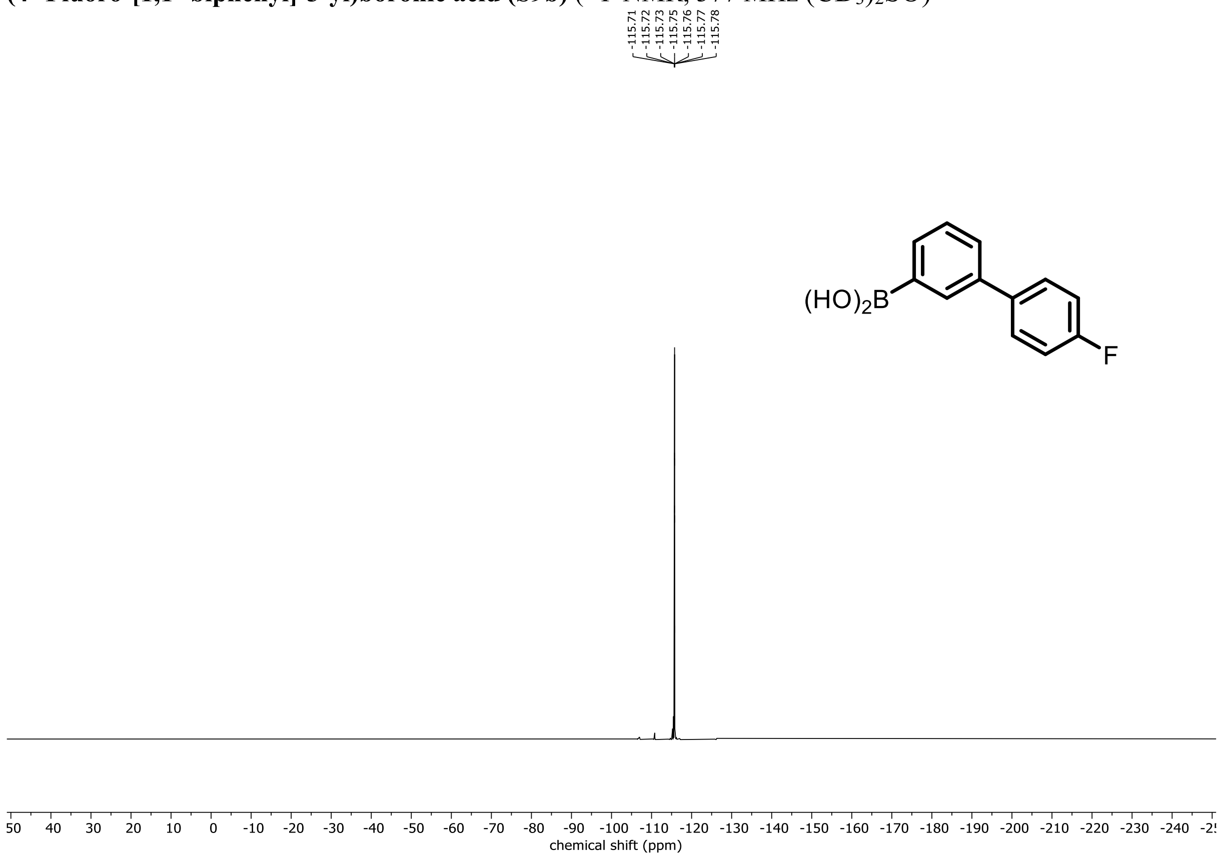

4-Bromo-4''-fluoro-1,1':3',1''-terphenyl (S9c) (^{1}H NMR, 400 MHz, $CDCl_3$)

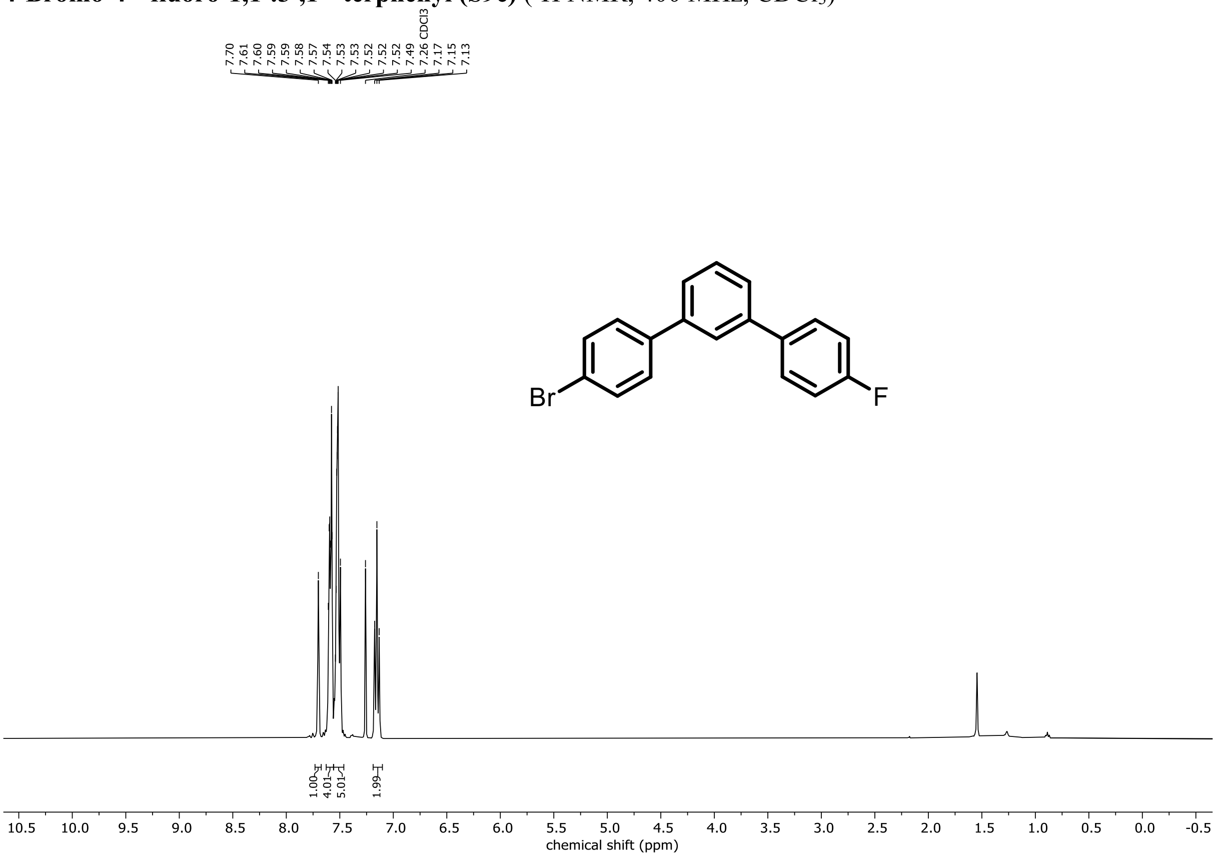

4-Bromo-4''-fluoro-1,1':3',1''-terphenyl (S9c) (^{13}C NMR, 101 MHz, 400 MHz, $CDCl_3$)

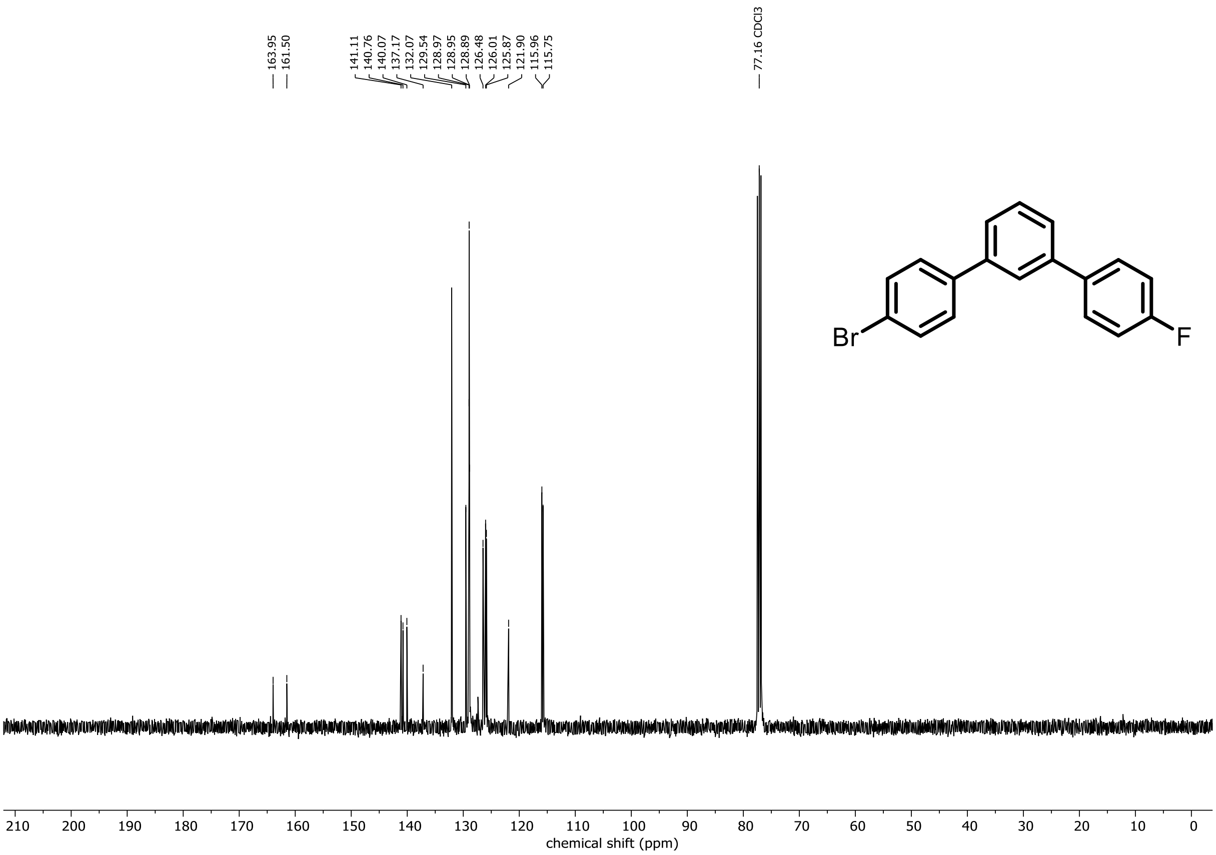

4-Bromo-4''-fluoro-1,1':3',1''-terphenyl (S9c) (^{19}F NMR, 377 MHz, $CDCl_3$)

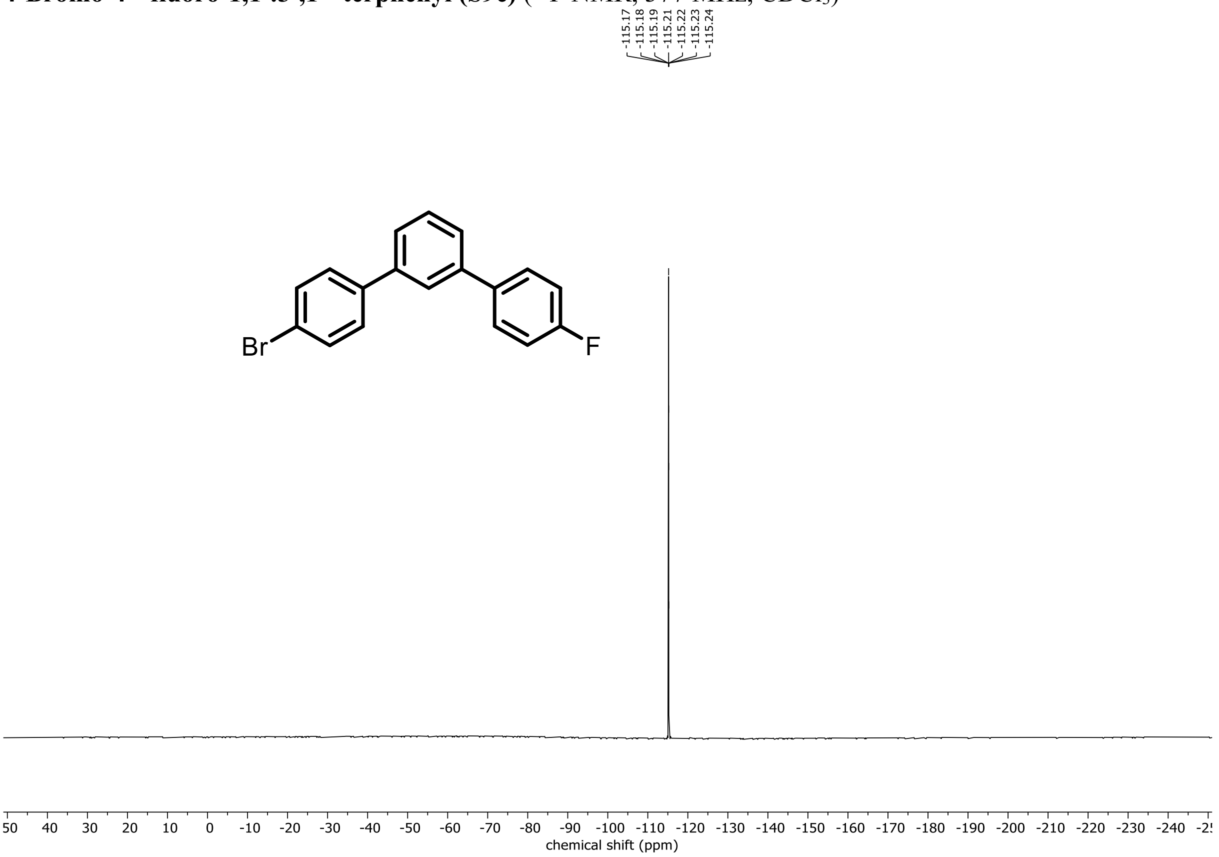

2-(4''-Fluoro-[1,1':3',1''-terphenyl]-4-yl)-4,4,5,5-tetramethyl-1,3,2-dioxaborolane (9) (^{1}H NMR, 400 MHz, $CDCl_3$)

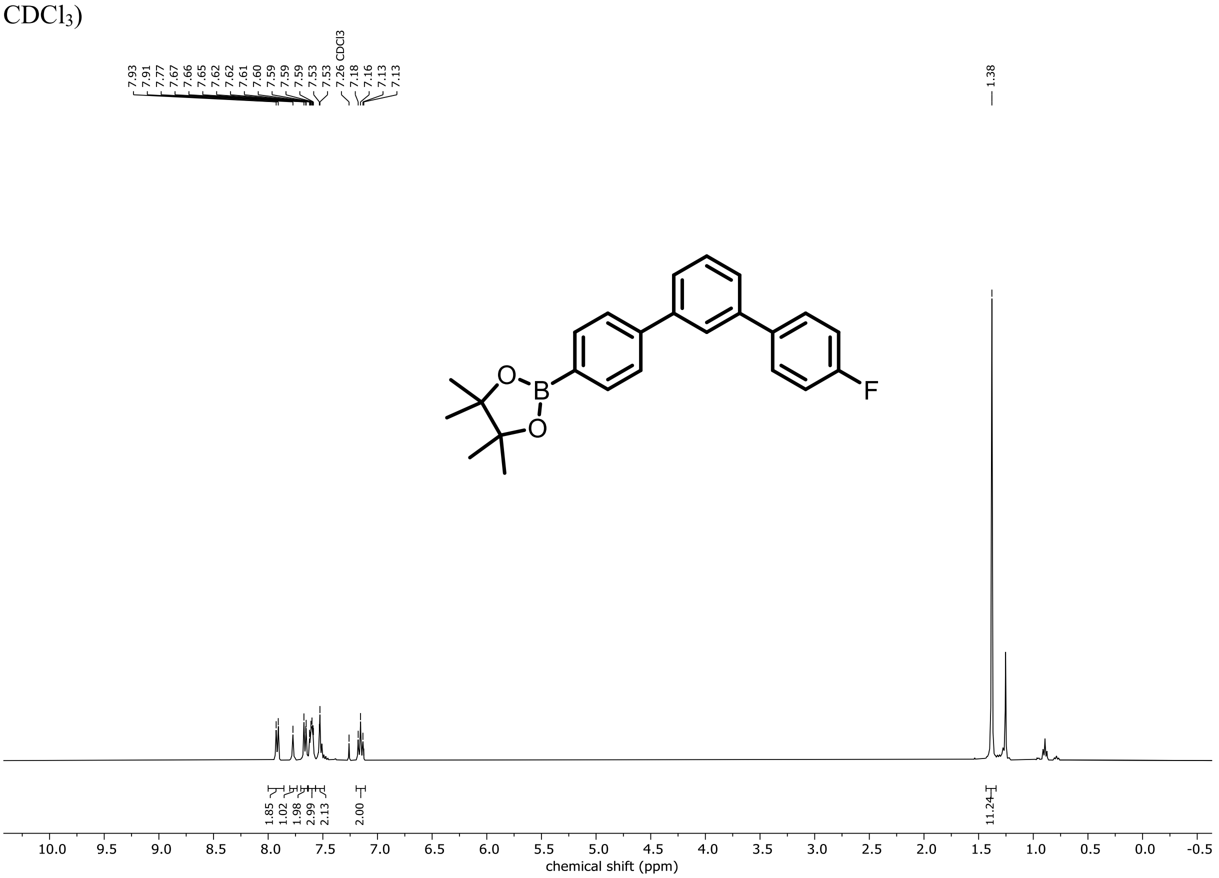

2-(4''-Fluoro-[1,1':3',1''-terphenyl]-4-yl)-4,4,5,5-tetramethyl-1,3,2-dioxaborolane (9) (^{13}C NMR, 101 MHz, 400 MHz, $CDCl_3$)

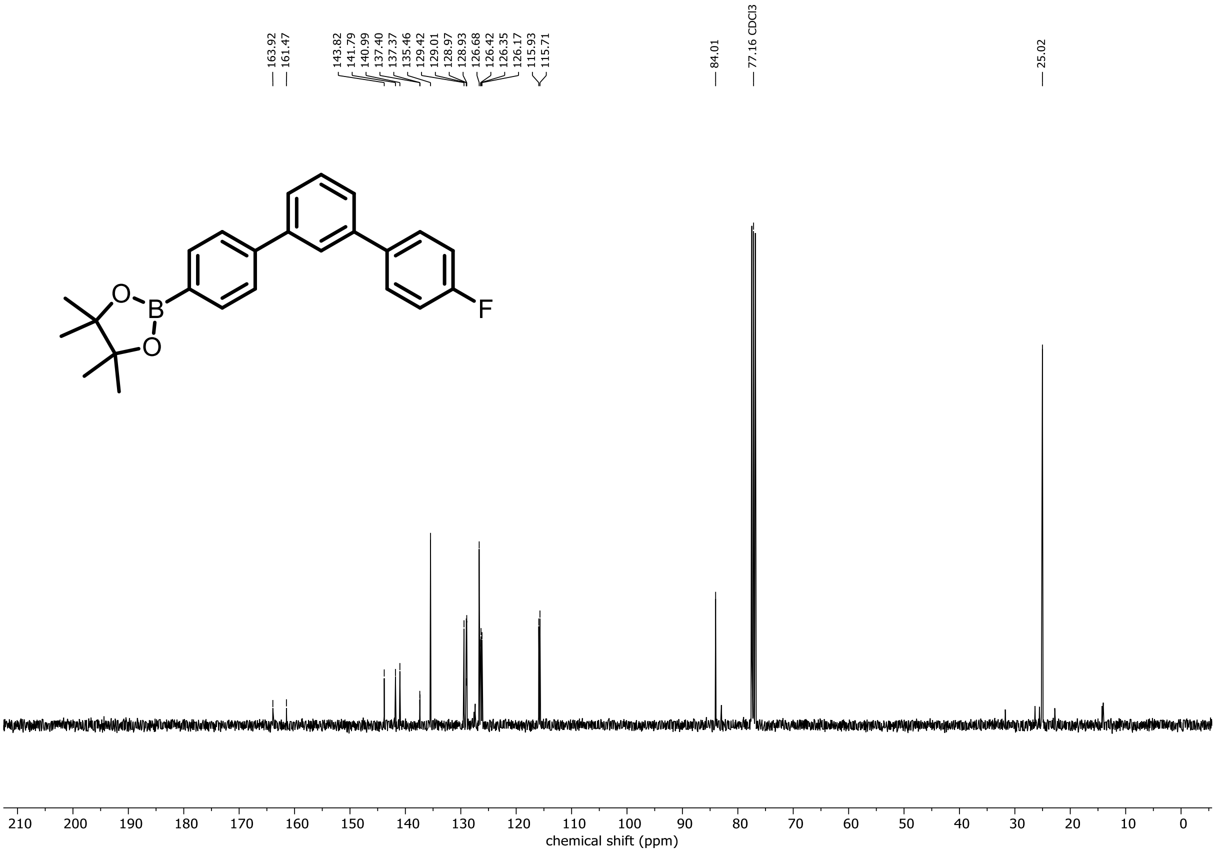

2-(4''-Fluoro-[1,1':3',1''-terphenyl]-4-yl)-4,4,5,5-tetramethyl-1,3,2-dioxaborolane (9) (^{19}F NMR, 377 MHz, $CDCl_3$)

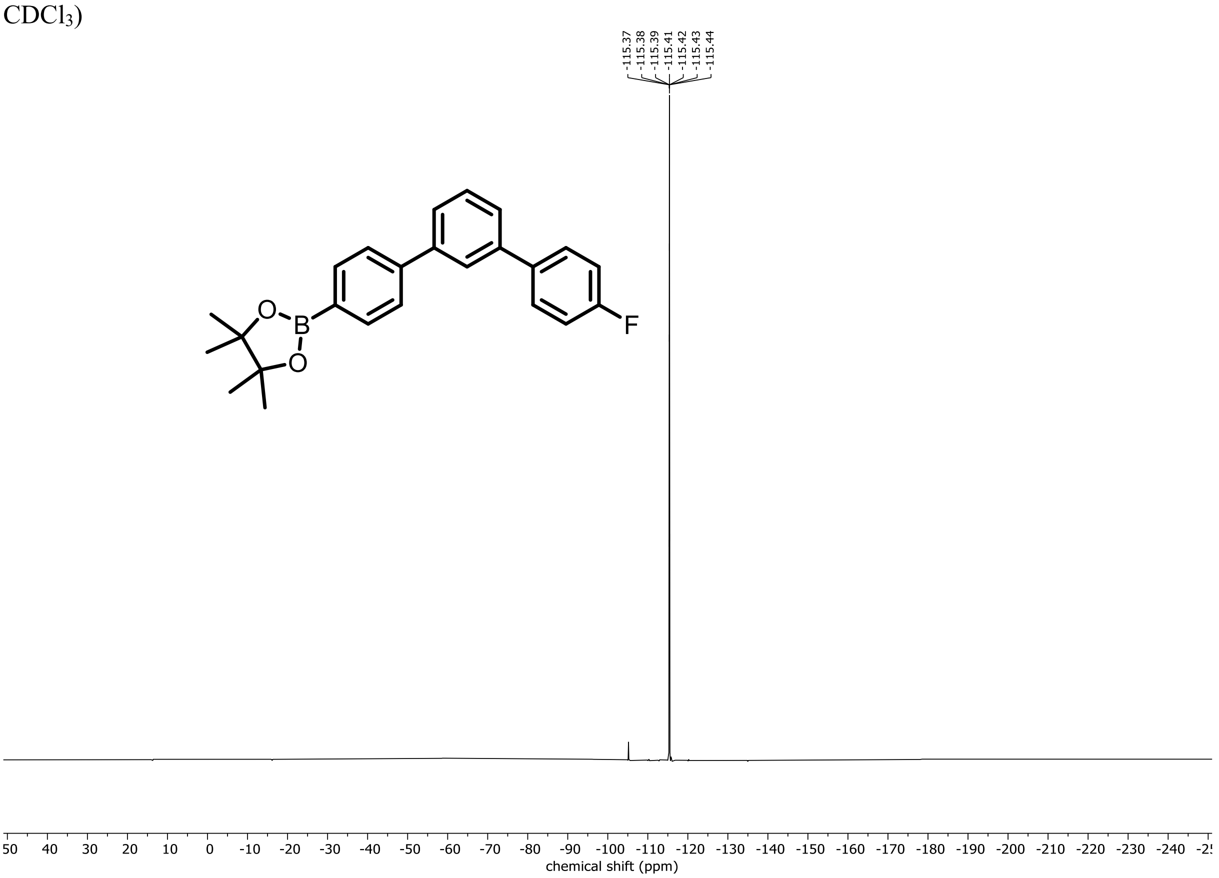

(4-Bromophenyl)(4-fluorophenyl)methanol (S10a) (^{1}H NMR, 400 MHz, $CDCl_3$)

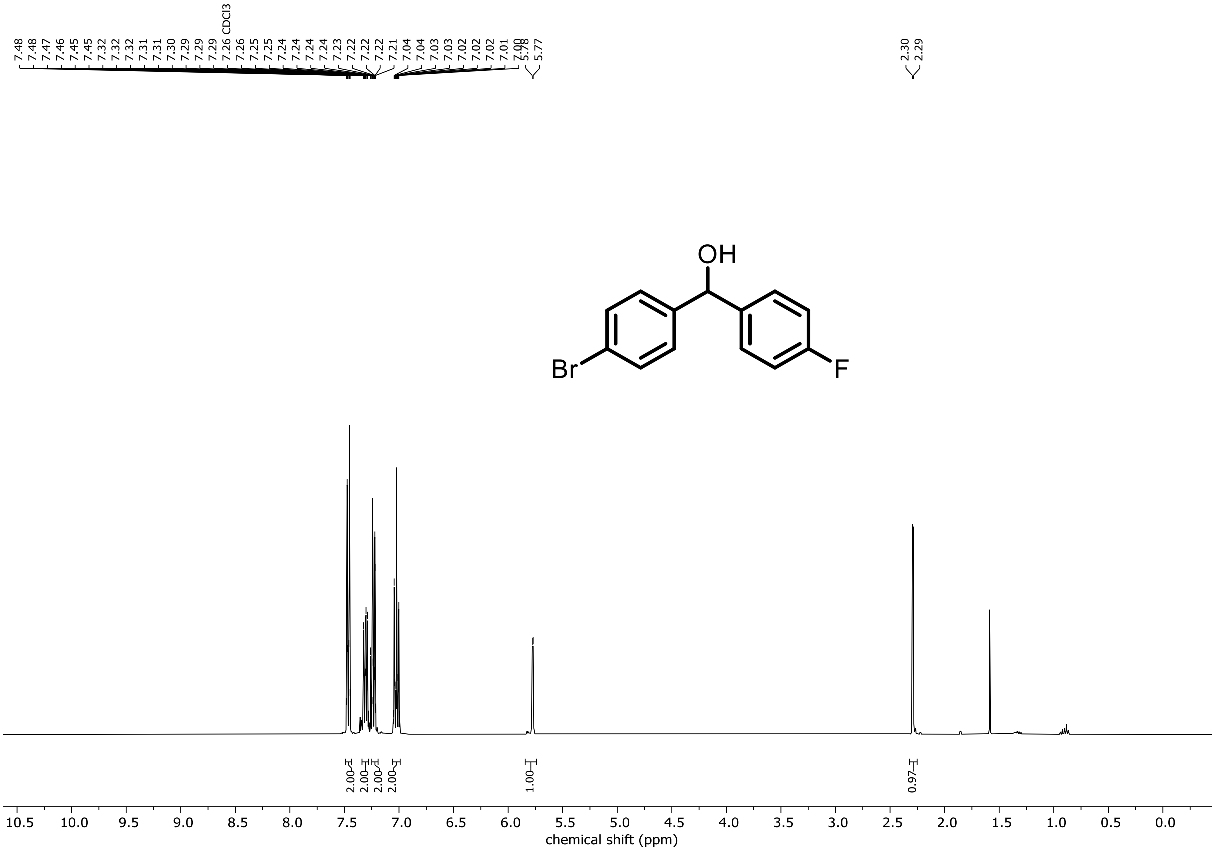

(4-romophenyl)(4-fluorophenyl)methanol (S10a) (^{13}C NMR, 101 MHz, $CDCl_3$)

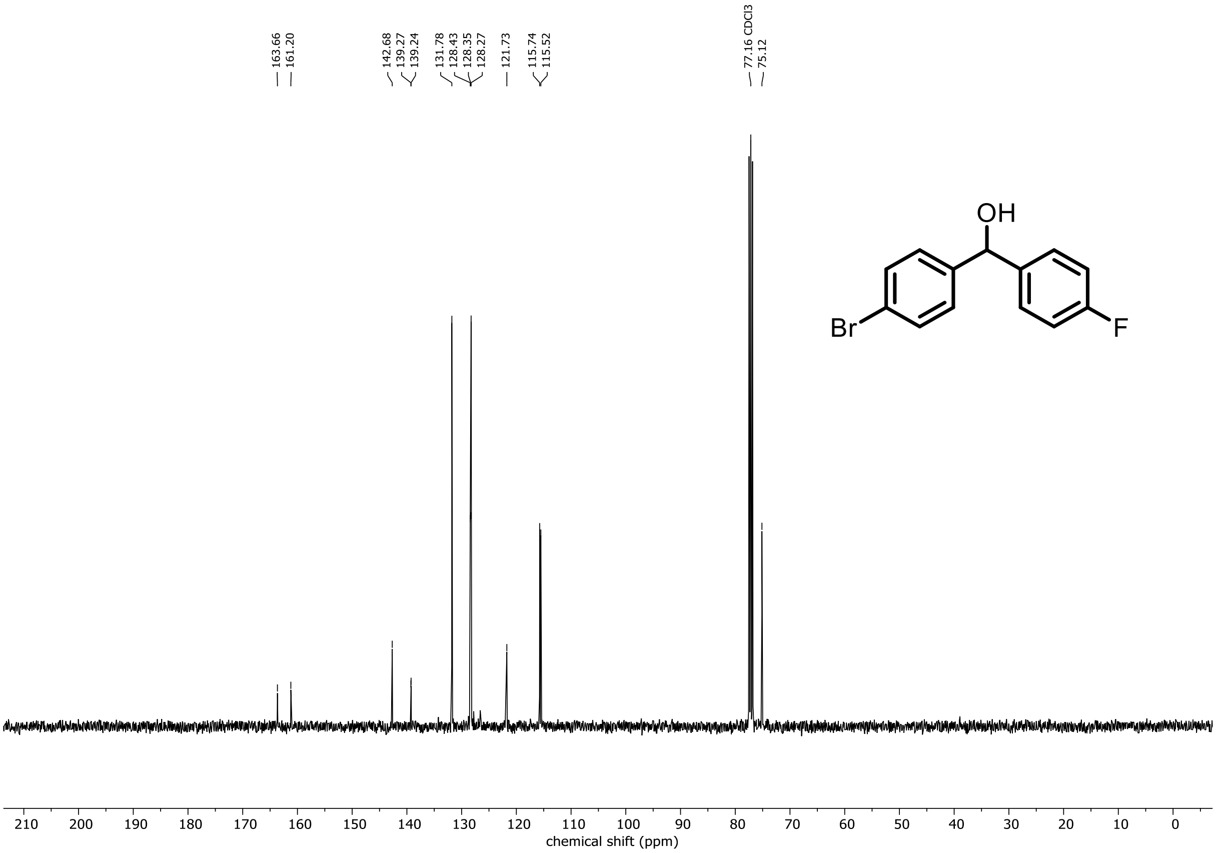

(4-Bromophenyl)(4-fluorophenyl)methanol (S10a) (^{19}F NMR, 377 MHz, $CDCl_3$)

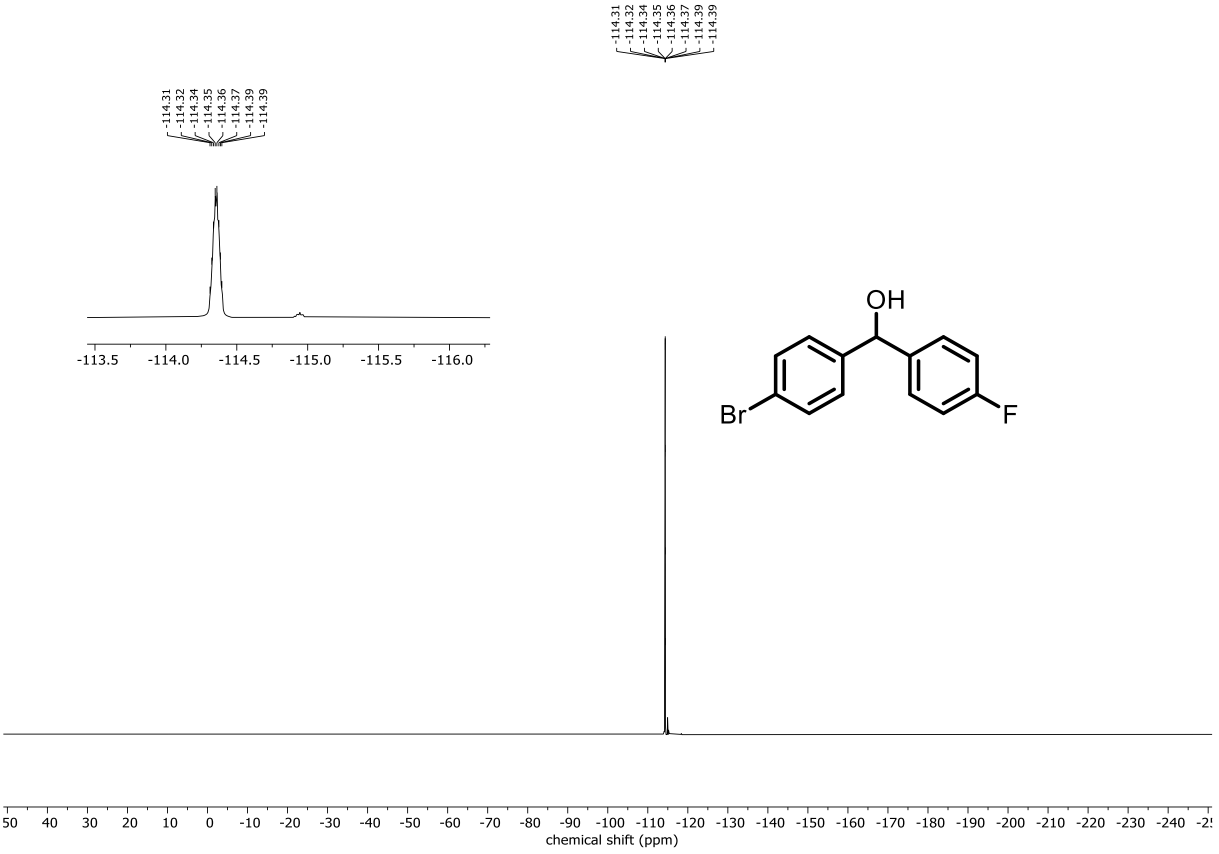

1-Bromo-4-(4-fluorobenzyl)benzene (S10b) (^{1}H NMR, 400 MHz, $CDCl_3$)

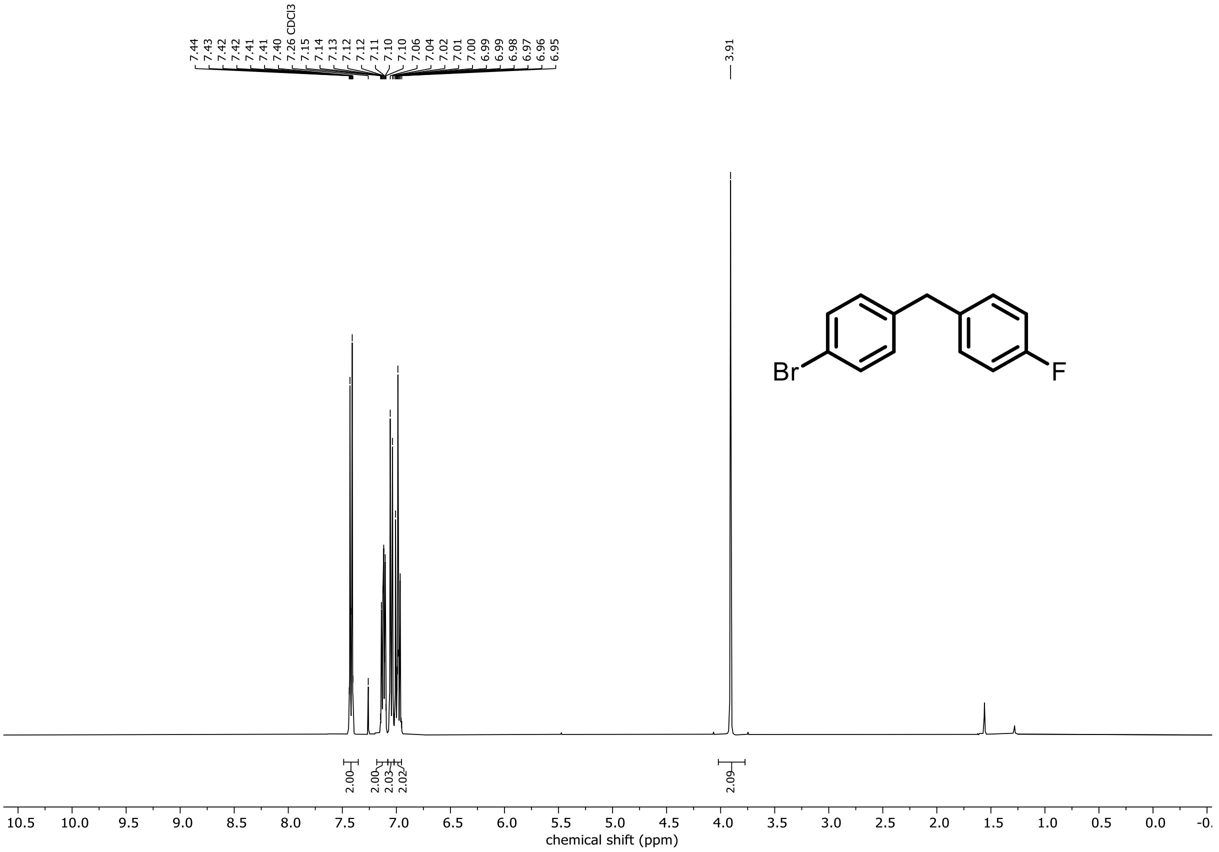

1-Bromo-4-(4-fluorobenzyl)benzene (S10b) (^{13}C NMR, 101 MHz, $CDCl_3$)

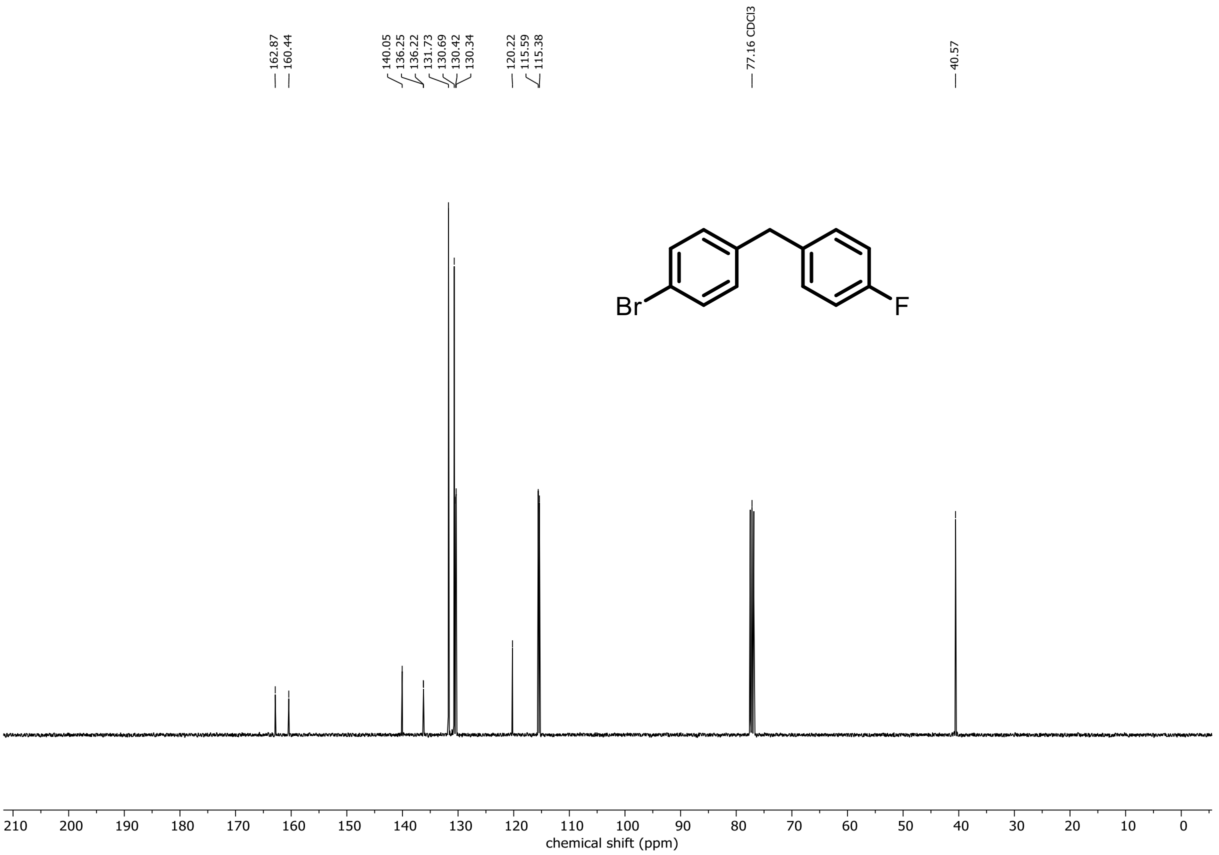

1-Bromo-4-(4-fluorobenzyl)benzene (S10b) (^{19}F {^{1}H} NMR, 376 MHz, $CDCl_3$)

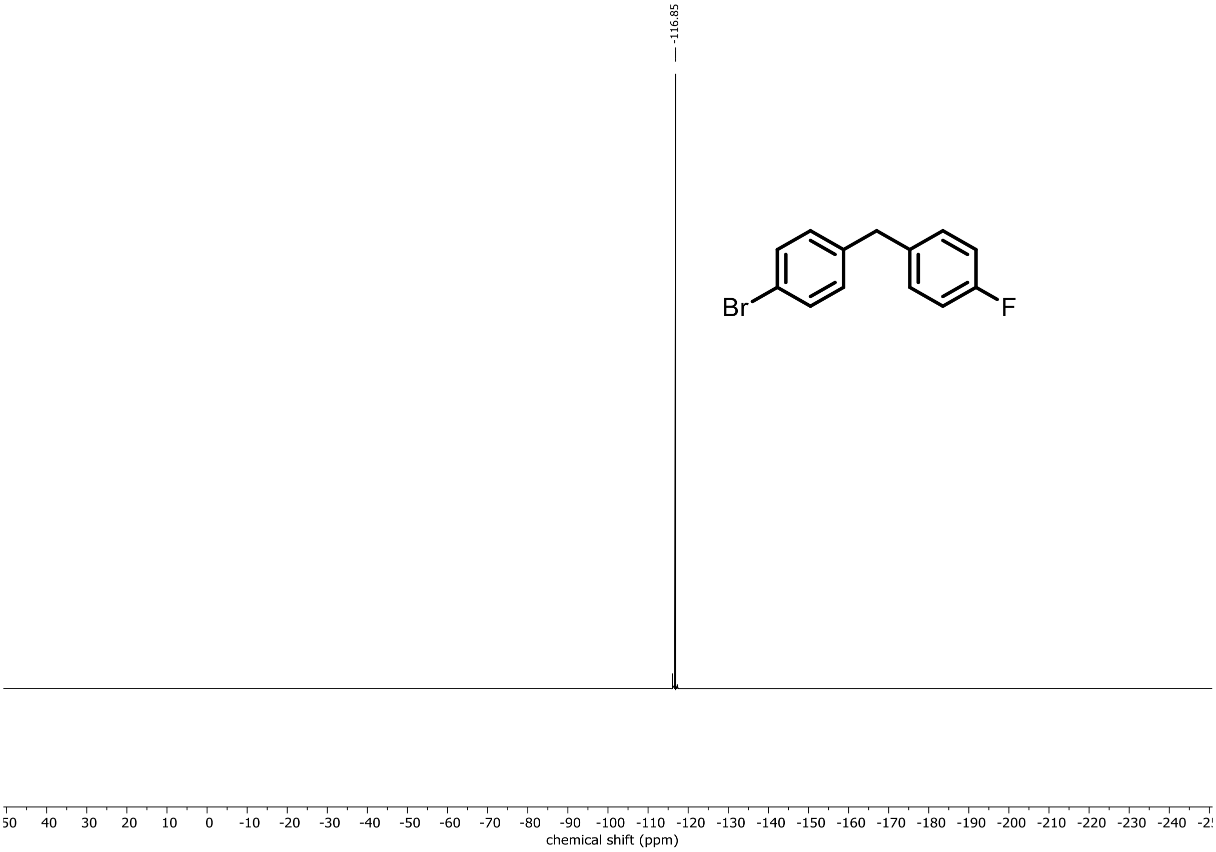

2-(4-(4-Fluorobenzyl)phenyl)-4,4,5,5-tetramethyl-1,3,2-dioxaborolane (10) (^{1}H NMR, 400 MHz, $CDCl_3$)

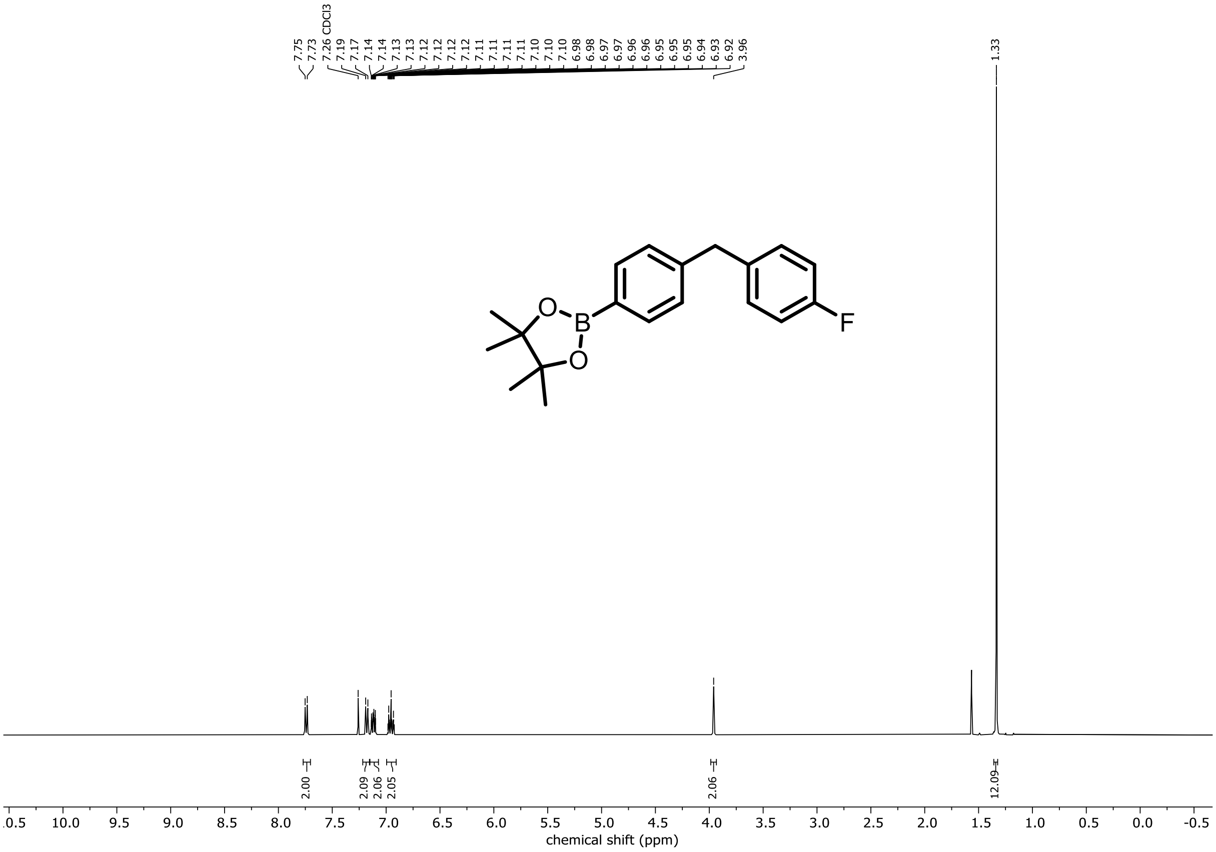

2-(4-(4-Fluorobenzyl)phenyl)-4,4,5,5-tetramethyl-1,3,2-dioxaborolane (10) (^{13}C NMR, 101 MHz, $CDCl_3$)

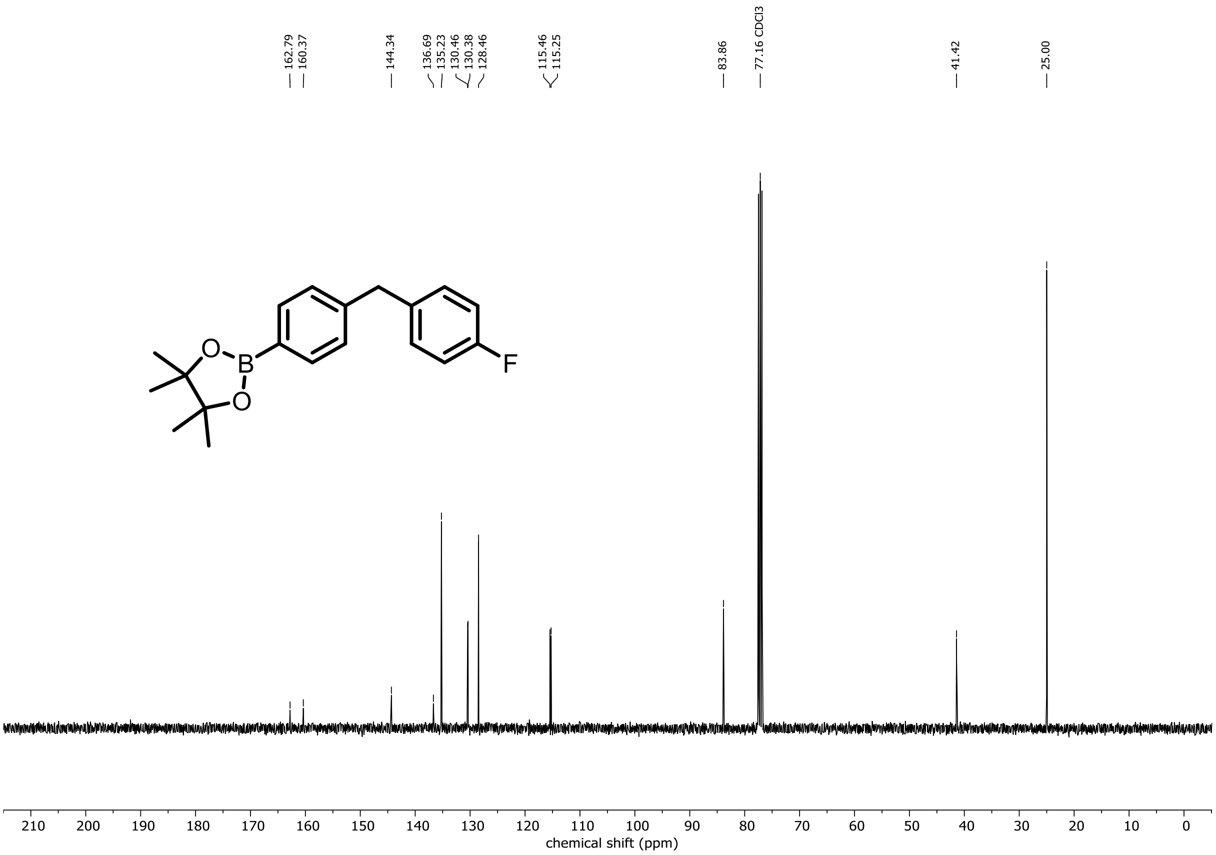

2-(4-(4-Fluorobenzyl)phenyl)-4,4,5,5-tetramethyl-1,3,2-dioxaborolane (10) (^{19}F NMR, 376 MHz, $CDCl_3$)

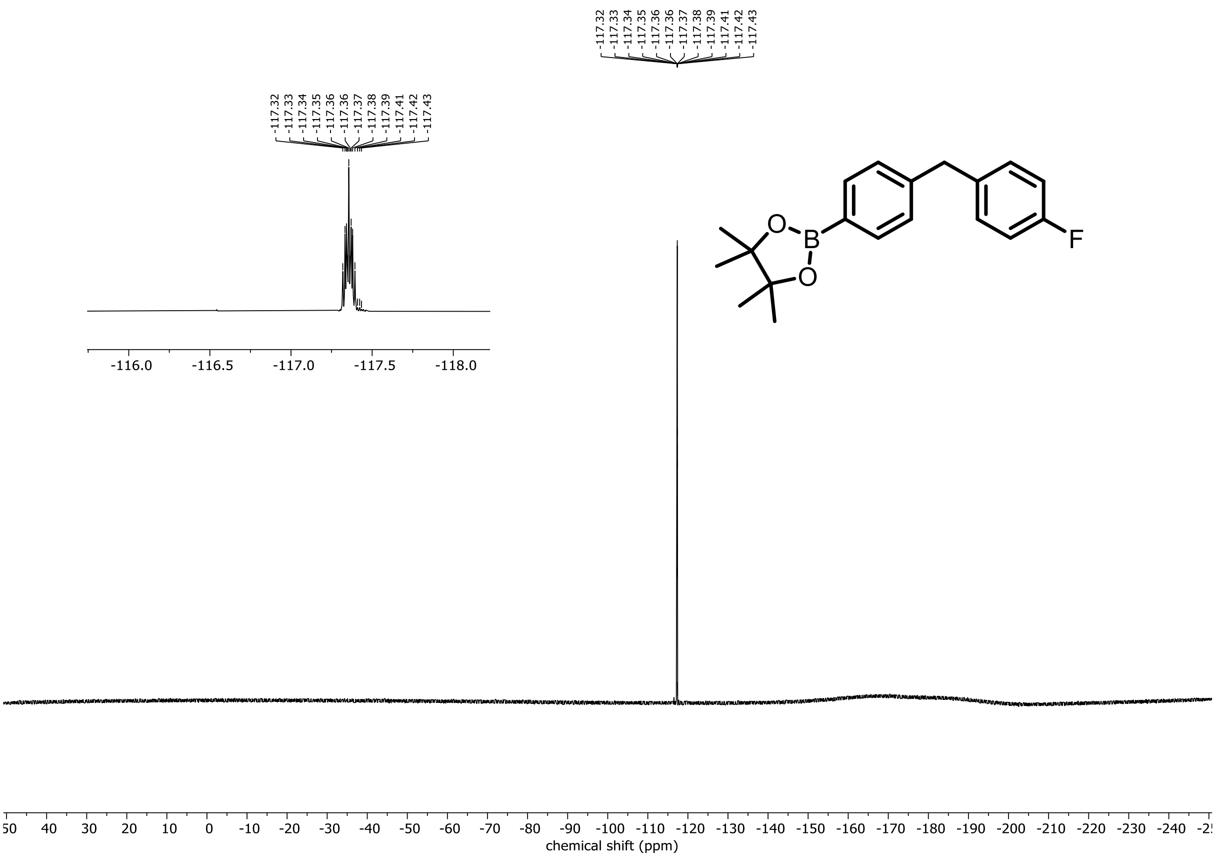

(4-Bromophenyl)(4'-fluoro-[1,1'-biphenyl]-4-yl)methanol (S11a) (^{1}H NMR, 400 MHz, $CDCl_3$)

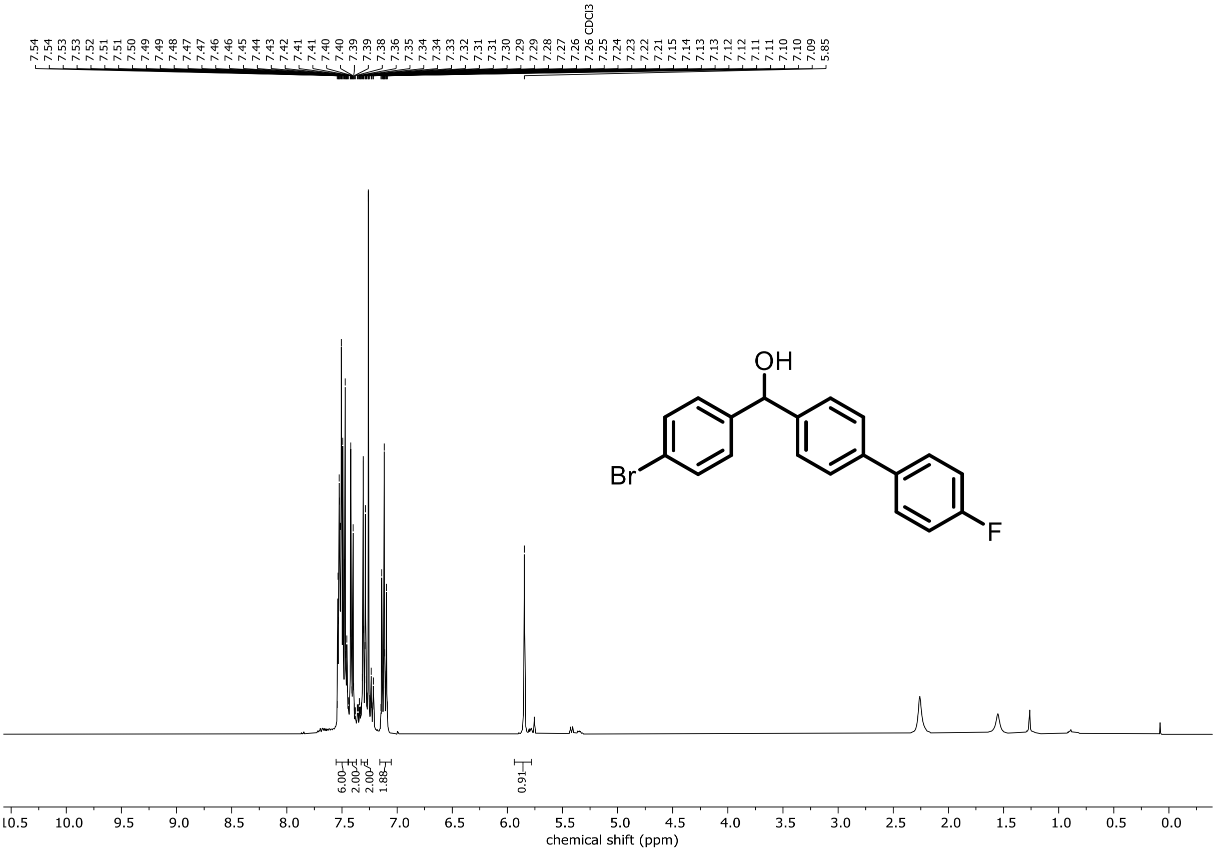

(4-Bromophenyl)(4'-fluoro-[1,1'-biphenyl]-4-yl)methanol (S11a) (^{13}C NMR, 101 MHz, $CDCl_3$)

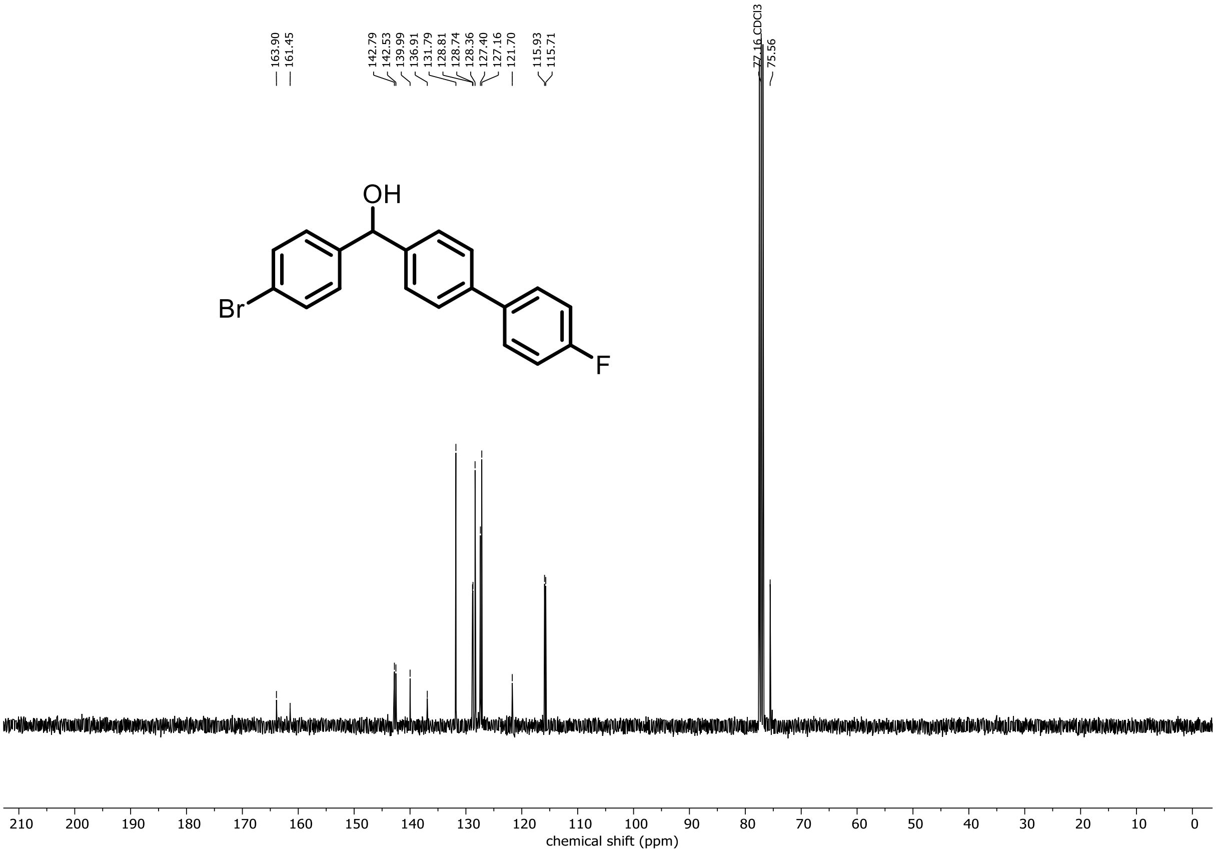

(4-Bromophenyl)(4'-fluoro-[1,1'-biphenyl]-4-yl)methanol (S11a) (^{19}F {1H} NMR, 376 MHz, $CDCl_3$)

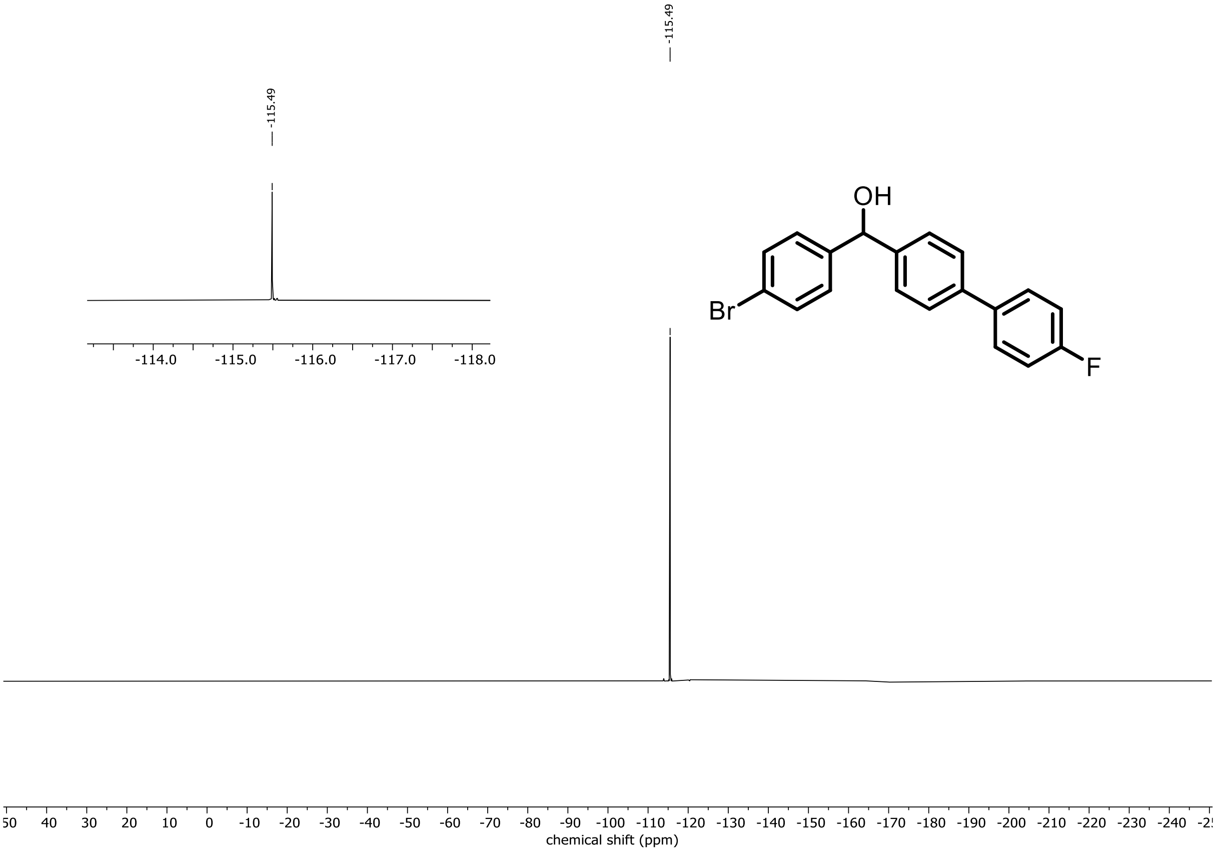

4-(4-Bromobenzyl)-4'-fluoro-1,1'-biphenyl (S11b) (^{1}H NMR, 400 MHz, $CDCl_3$)

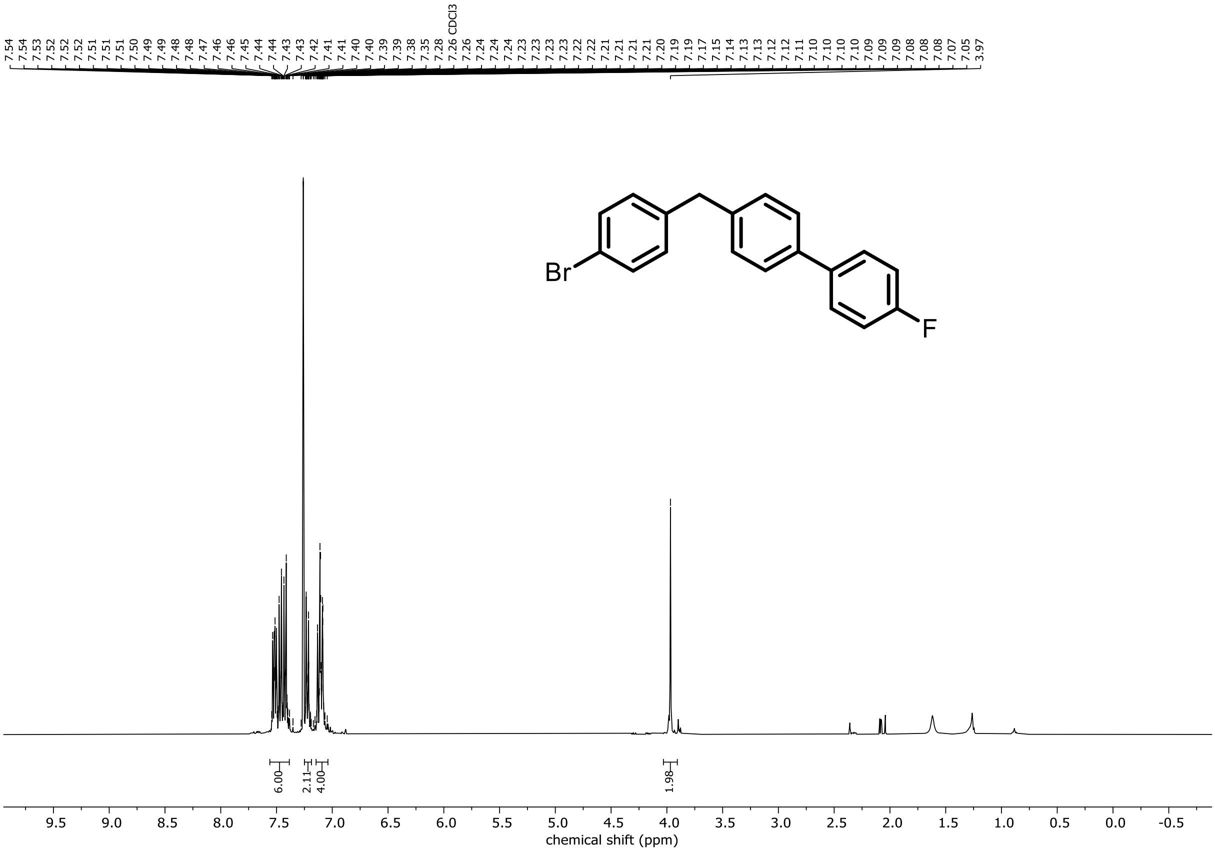

4-(4-Bromobenzyl)-4'-fluoro-1,1'-biphenyl (S11b) (^{13}C NMR, 101 MHz, $CDCl_3$)

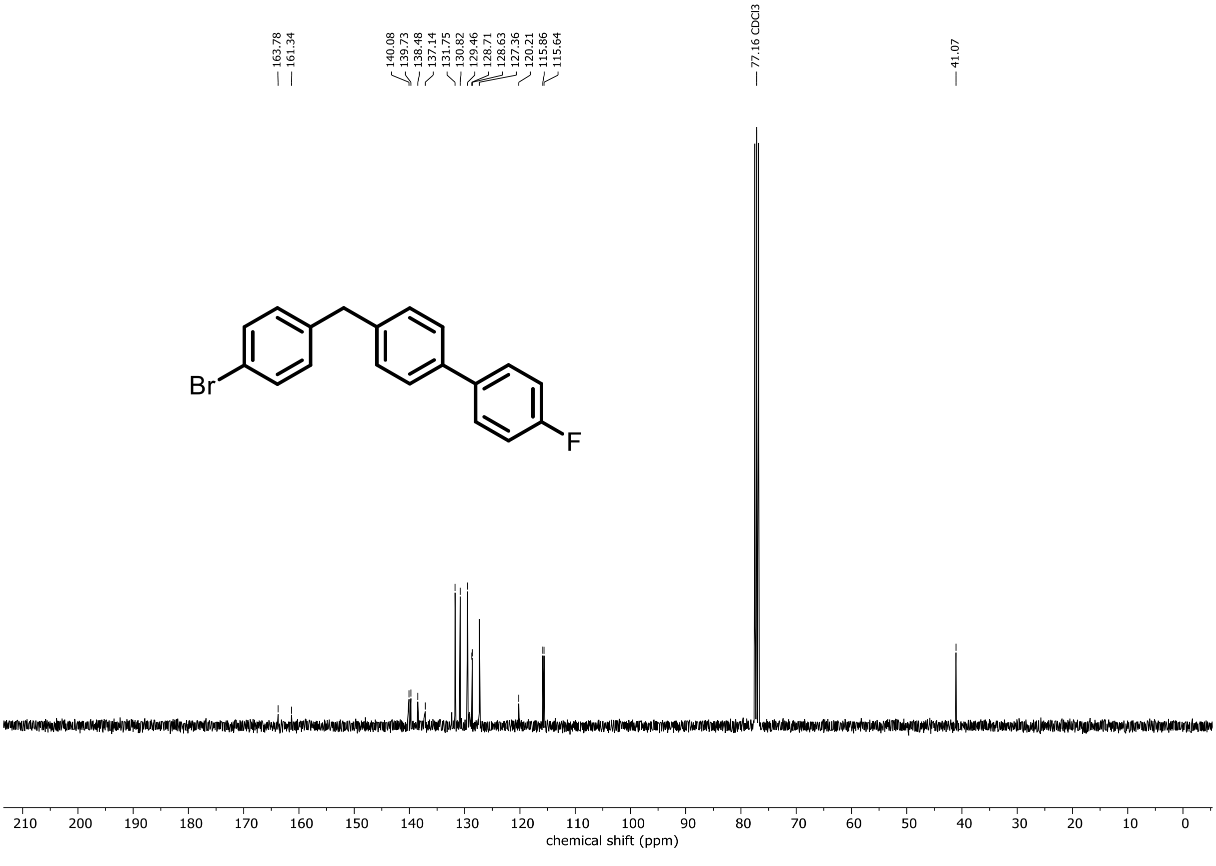

4-(4-Bromobenzyl)-4'-fluoro-1,1'-biphenyl (S11b) (^{19}F {1H} NMR, 376 MHz, $CDCl_3$)

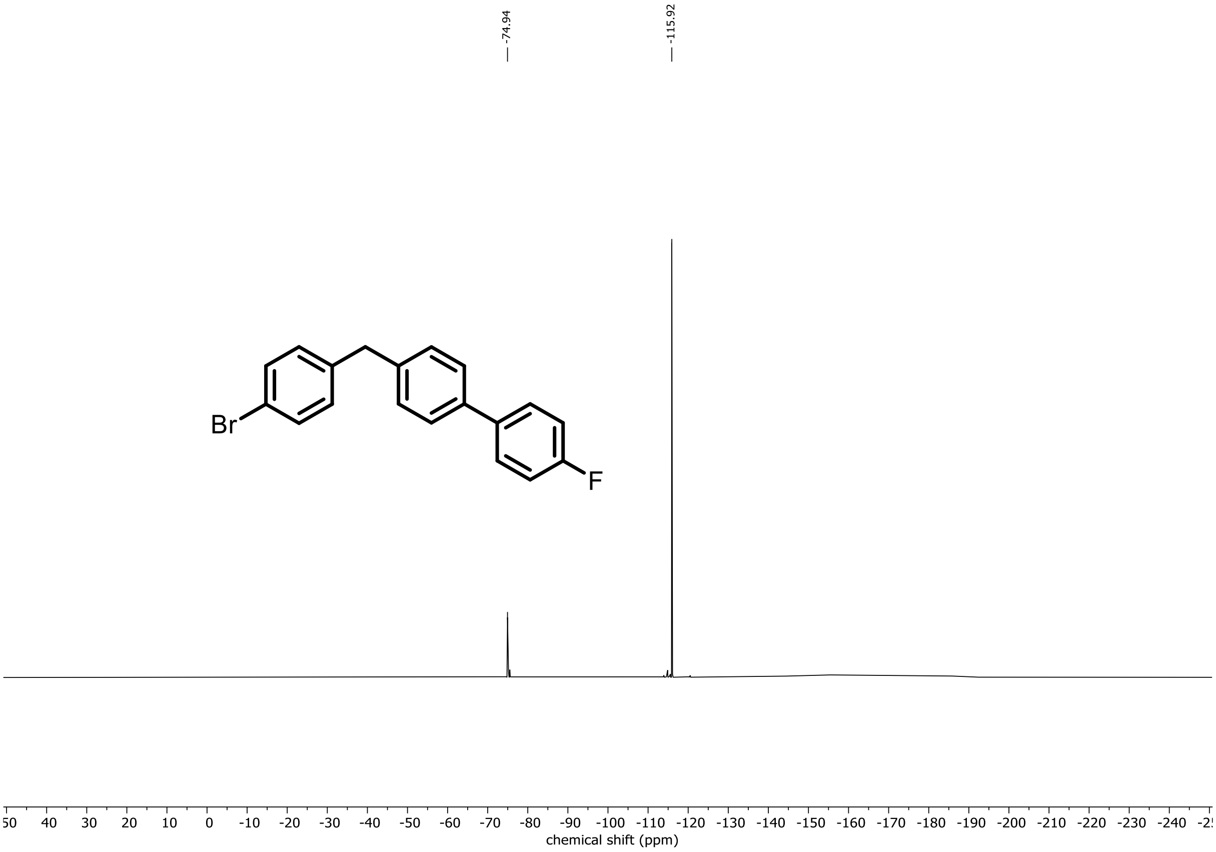

2-(4-((4'-Fluoro-[1,1'-biphenyl]-4-yl)methyl)phenyl)-4,4,5,5-tetramethyl-1,3,2-dioxaborolane (11) (^{1}H NMR, 400 MHz, $CDCl_3$)

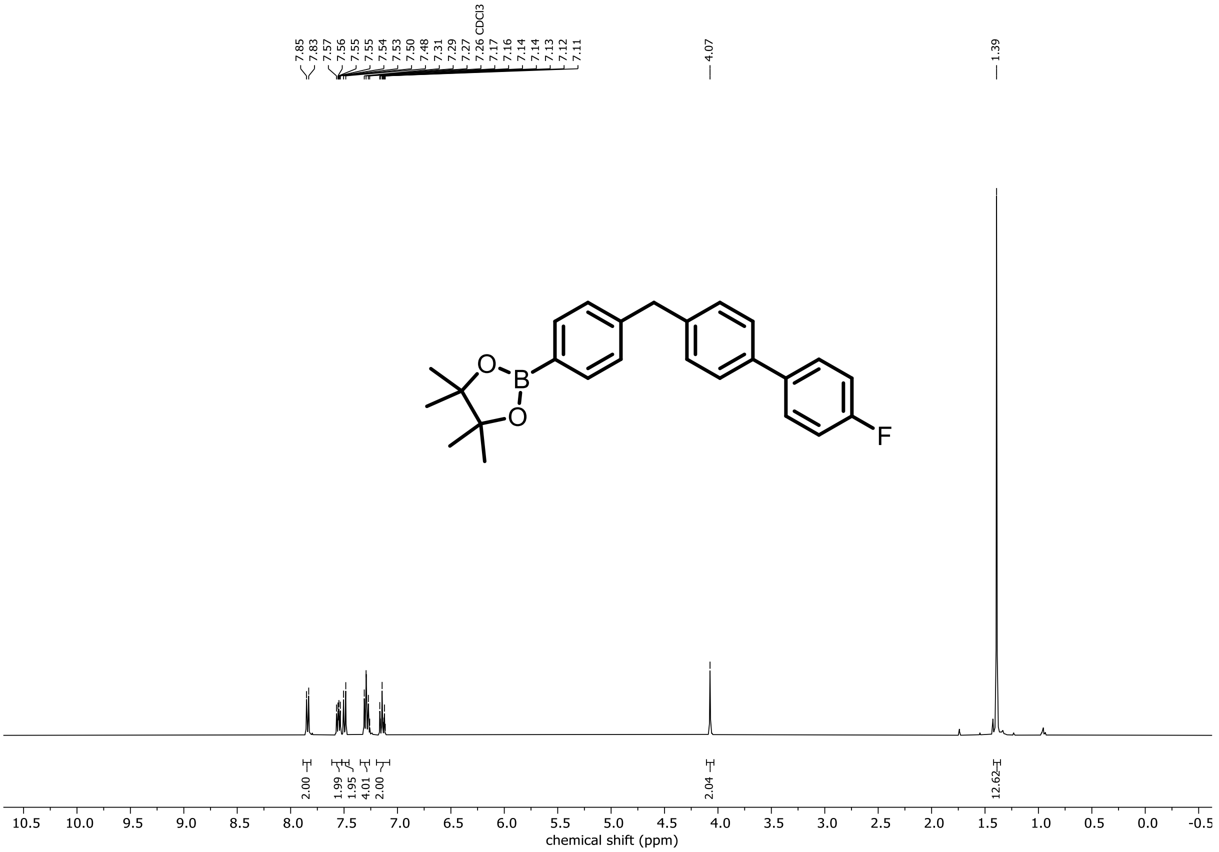

2-(4-((4'-Fluoro-[1,1'-biphenyl]-4-yl)methyl)phenyl)-4,4,5,5-tetramethyl-1,3,2-dioxaborolane (11) (^{13}C NMR, 101 MHz, $CDCl_3$)

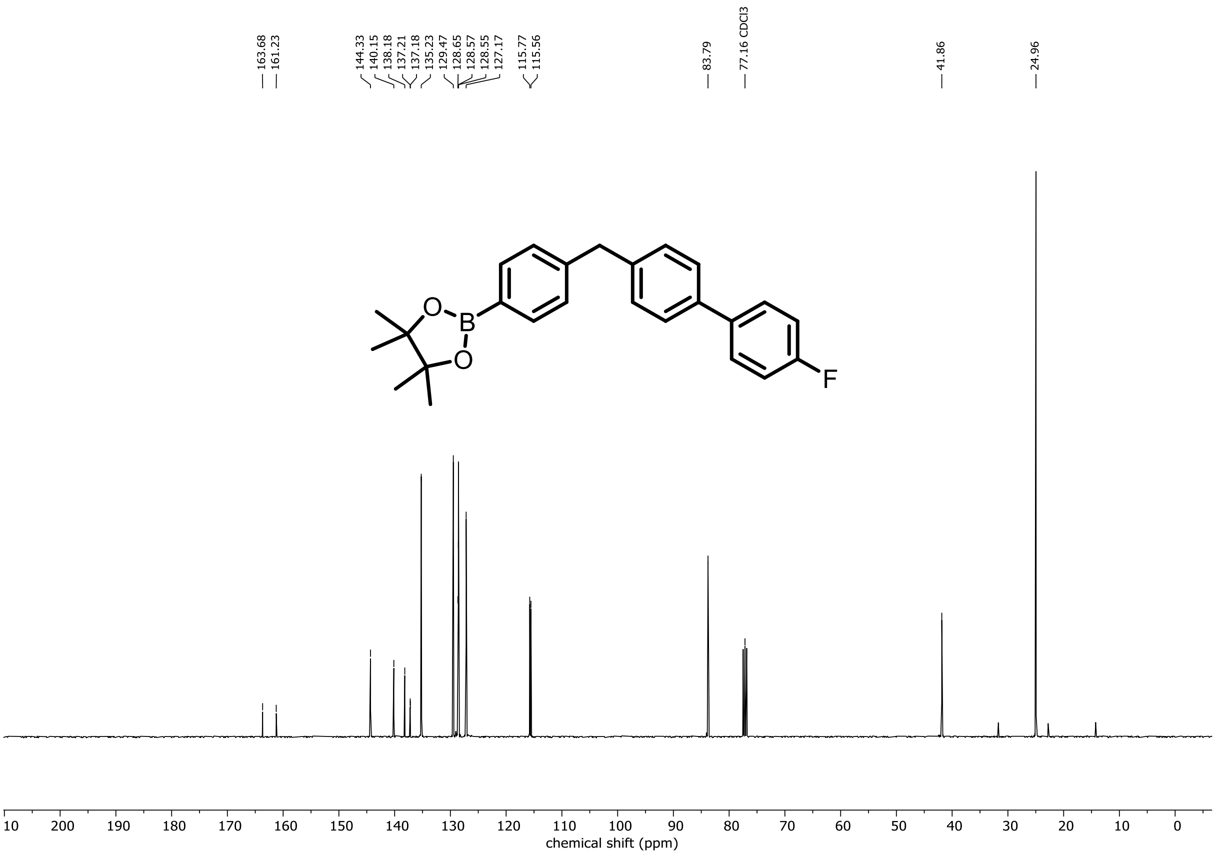

2-(4-((4'-Fluoro-[1,1'-biphenyl]-4-yl)methyl)phenyl)-4,4,5,5-tetramethyl-1,3,2-dioxaborolane (11) (^{19}F {1H} NMR, 376 MHz, $CDCl_3$)

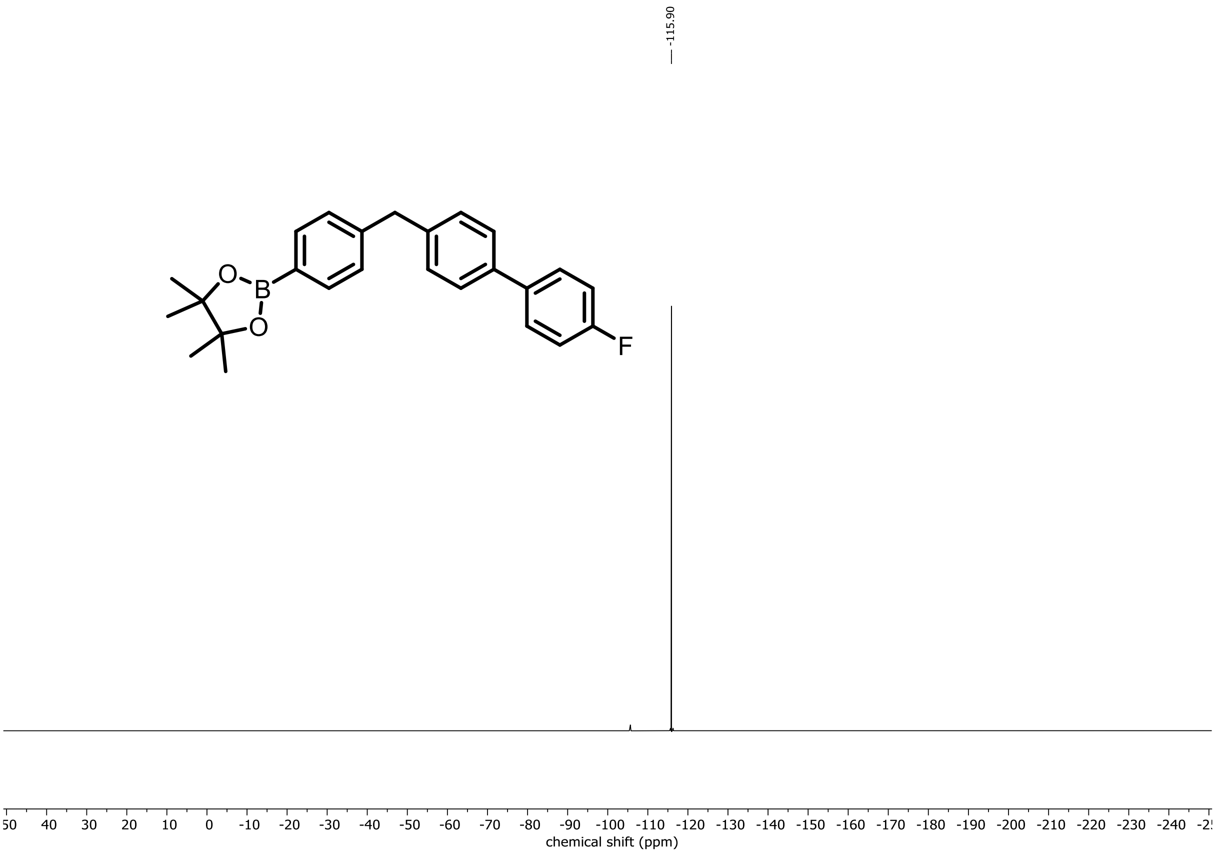

(4'-Bromo-[1,1'-biphenyl]-4-yl)(4-fluorophenyl)methanol (S12a) (^{1}H NMR, 400 MHz, $CDCl_3$)

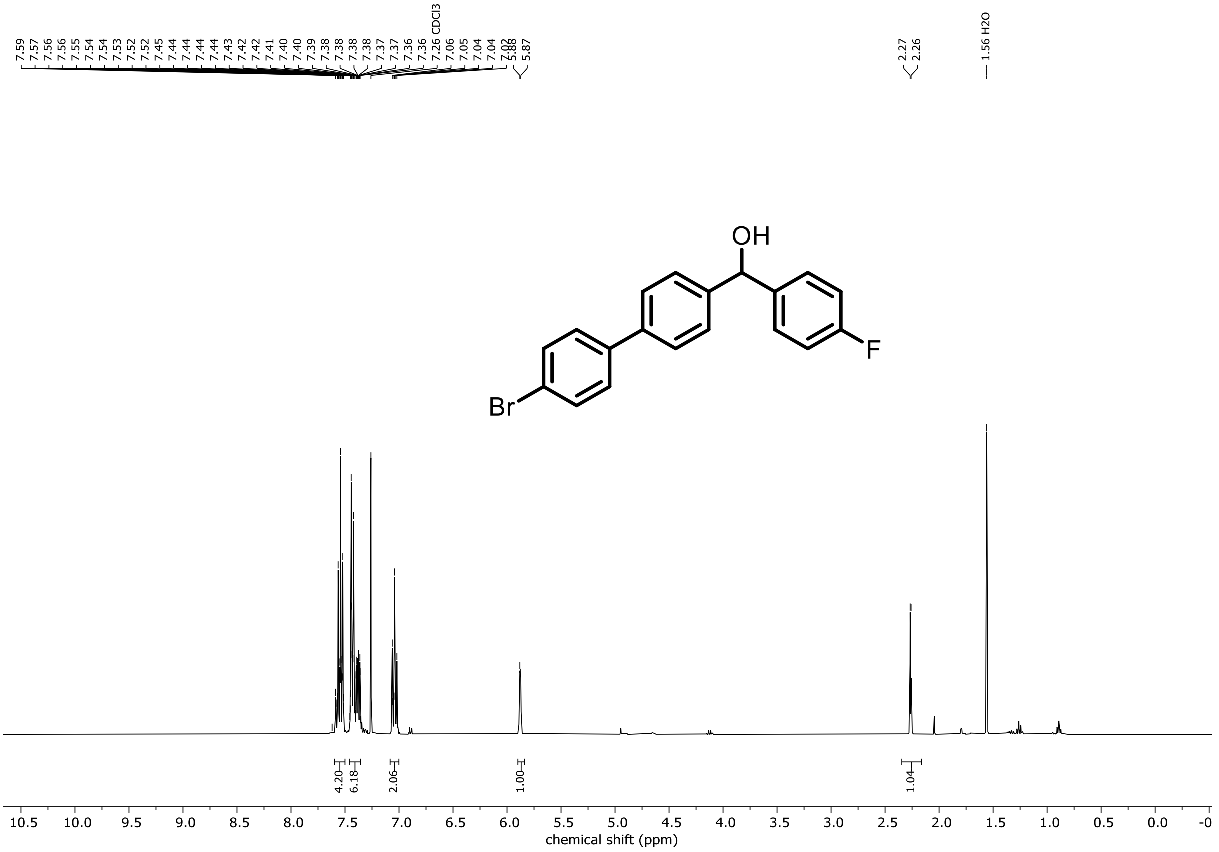

(4'-Bromo-[1,1'-biphenyl]-4-yl)(4-fluorophenyl)methanol (S12a) (^{13}C NMR, 101 MHz, $CDCl_3$)

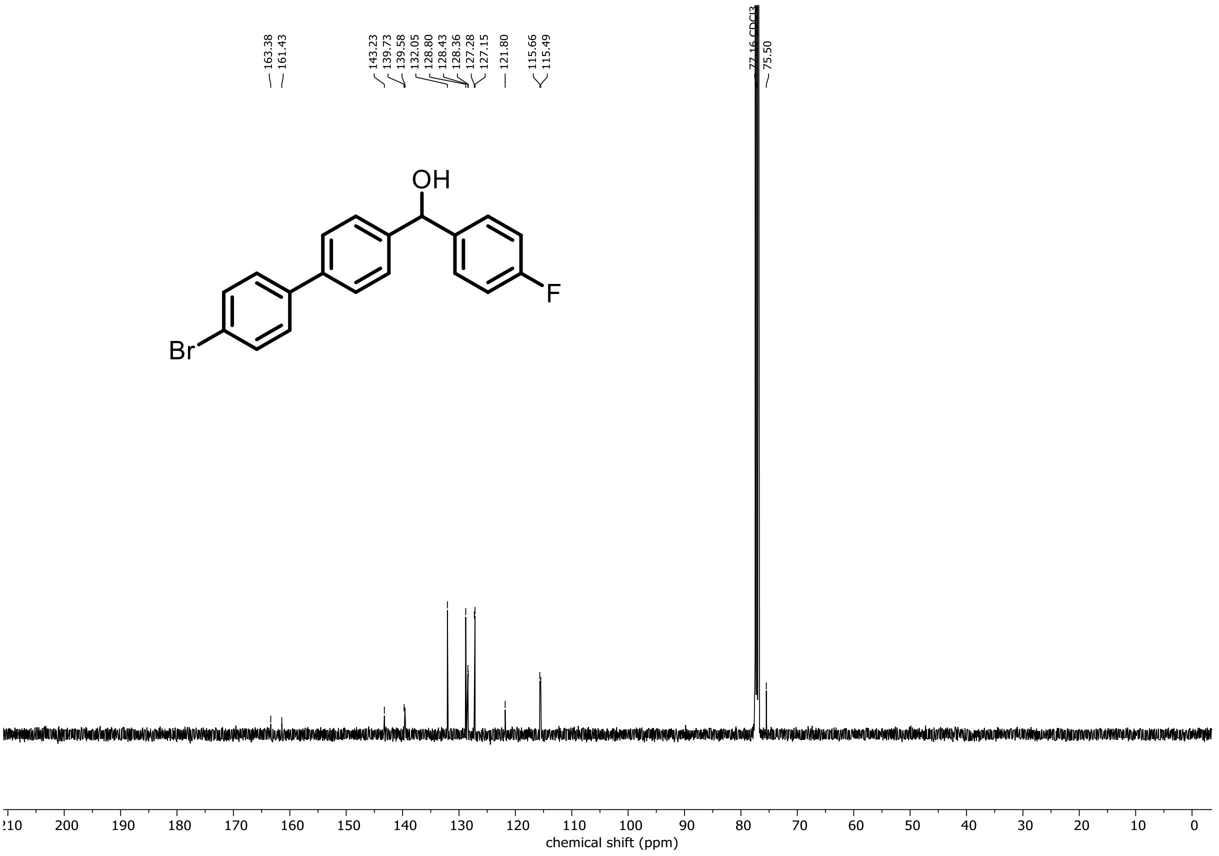

(4'-Bromo-[1,1'-biphenyl]-4-yl)(4-fluorophenyl)methanol (S12a) (^{19}F NMR, 376 MHz, $CDCl_3$)

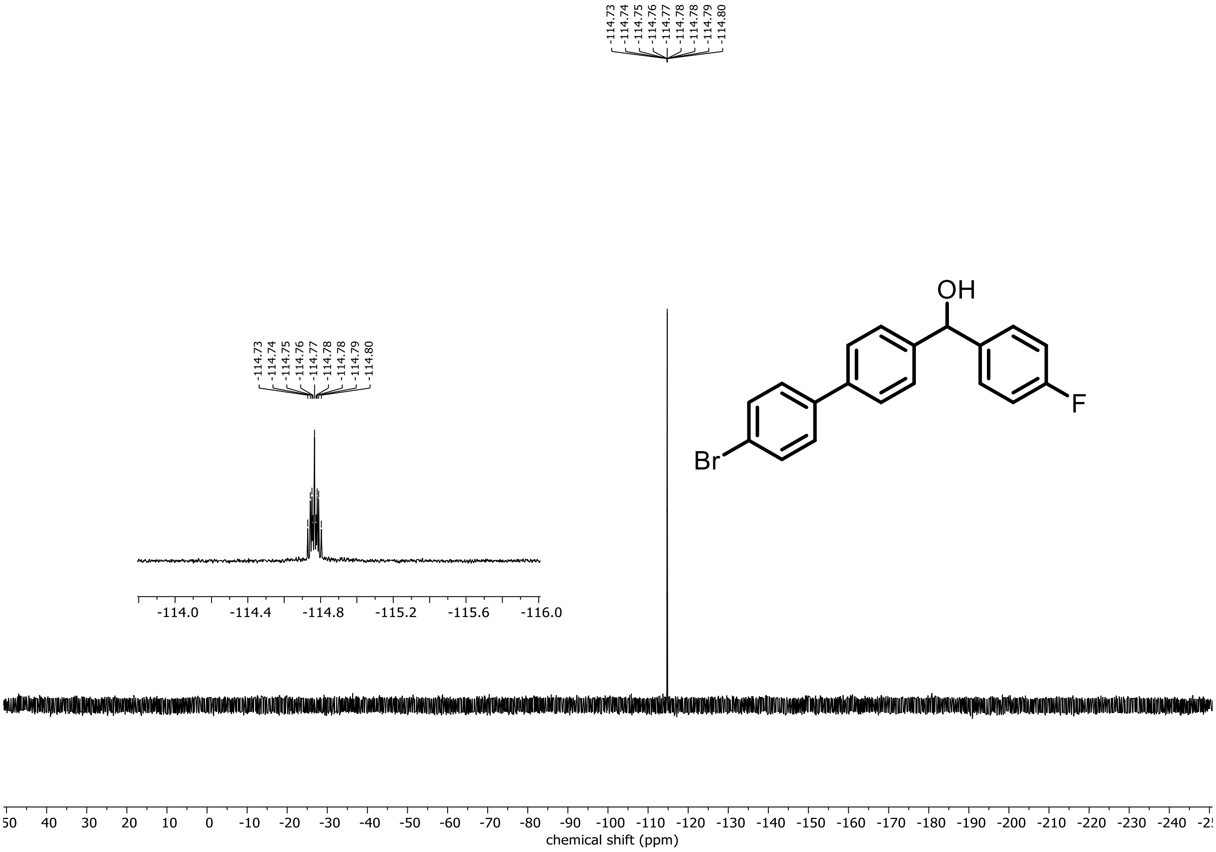

4-bromo-4'-(4-fluorobenzyl)-1,1'-biphenyl (S12b) (^{1}H NMR, 400 MHz, $CDCl_3$)

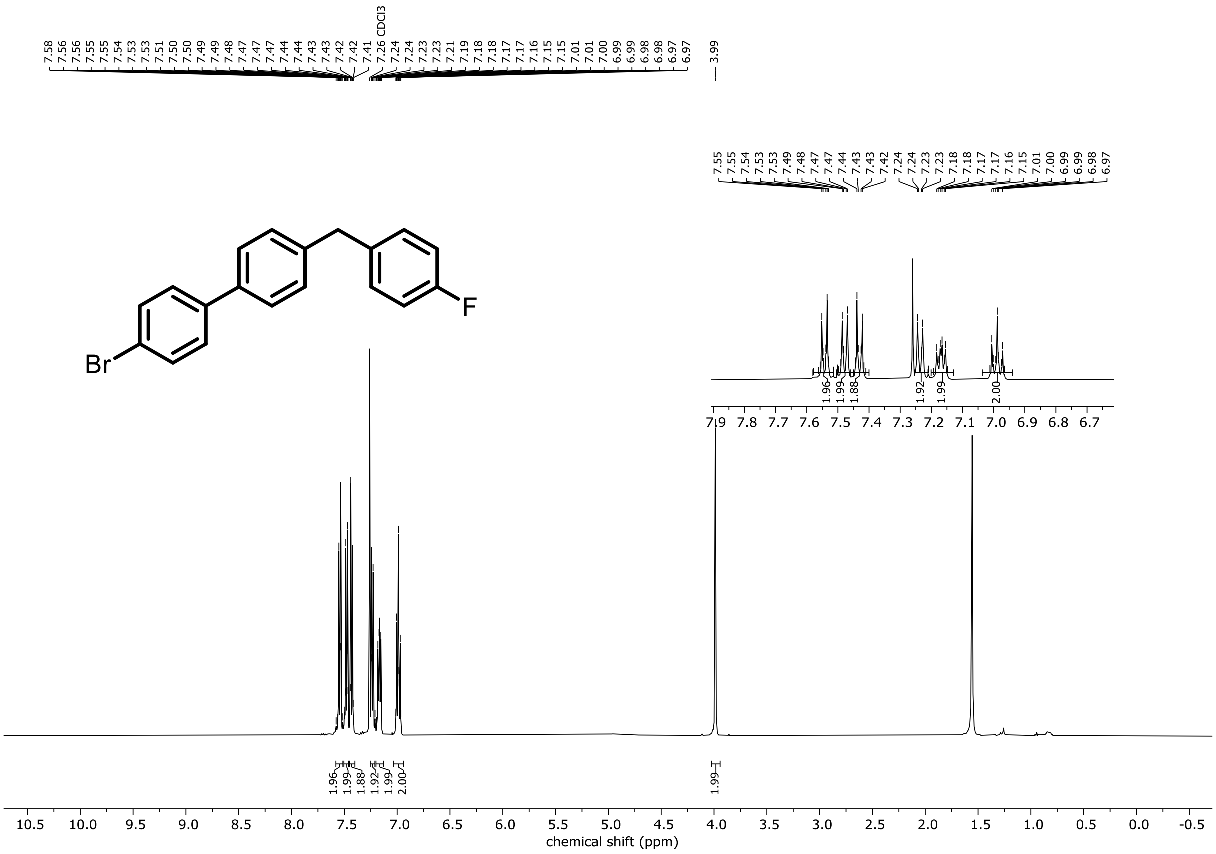

4-bromo-4'-(4-fluorobenzyl)-1,1'-biphenyl (S12b) (^{13}C NMR, 101 MHz, $CDCl_3$)

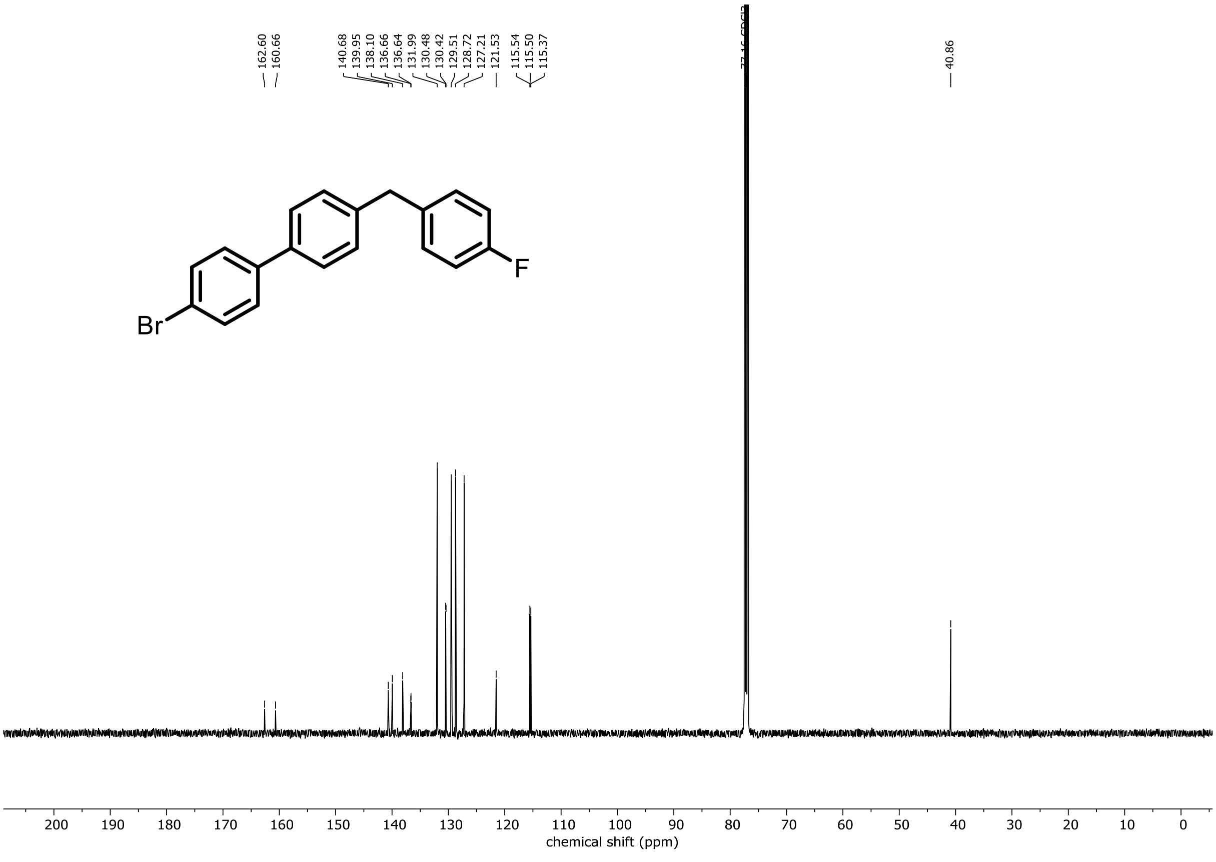

4-bromo-4'-(4-fluorobenzyl)-1,1'-biphenyl (S12b) (^{19}F {^{1}H} NMR, 376 MHz, $CDCl_3$)

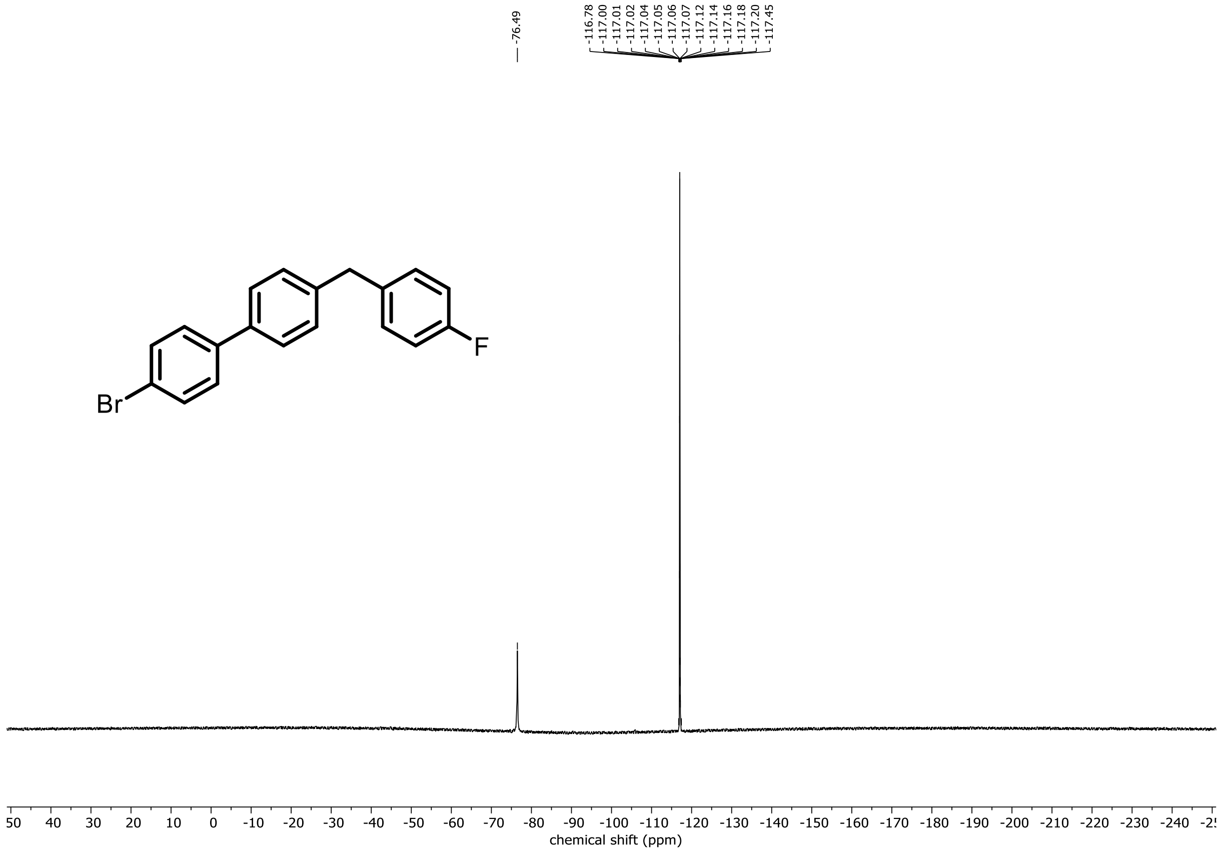

2-(4'-(4-fluorobenzyl)-[1,1'-biphenyl]-4-yl)-4,4,5,5-tetramethyl-1,3,2-dioxaborolane (12) (^{1}H NMR, 400 MHz, $CDCl_3$)

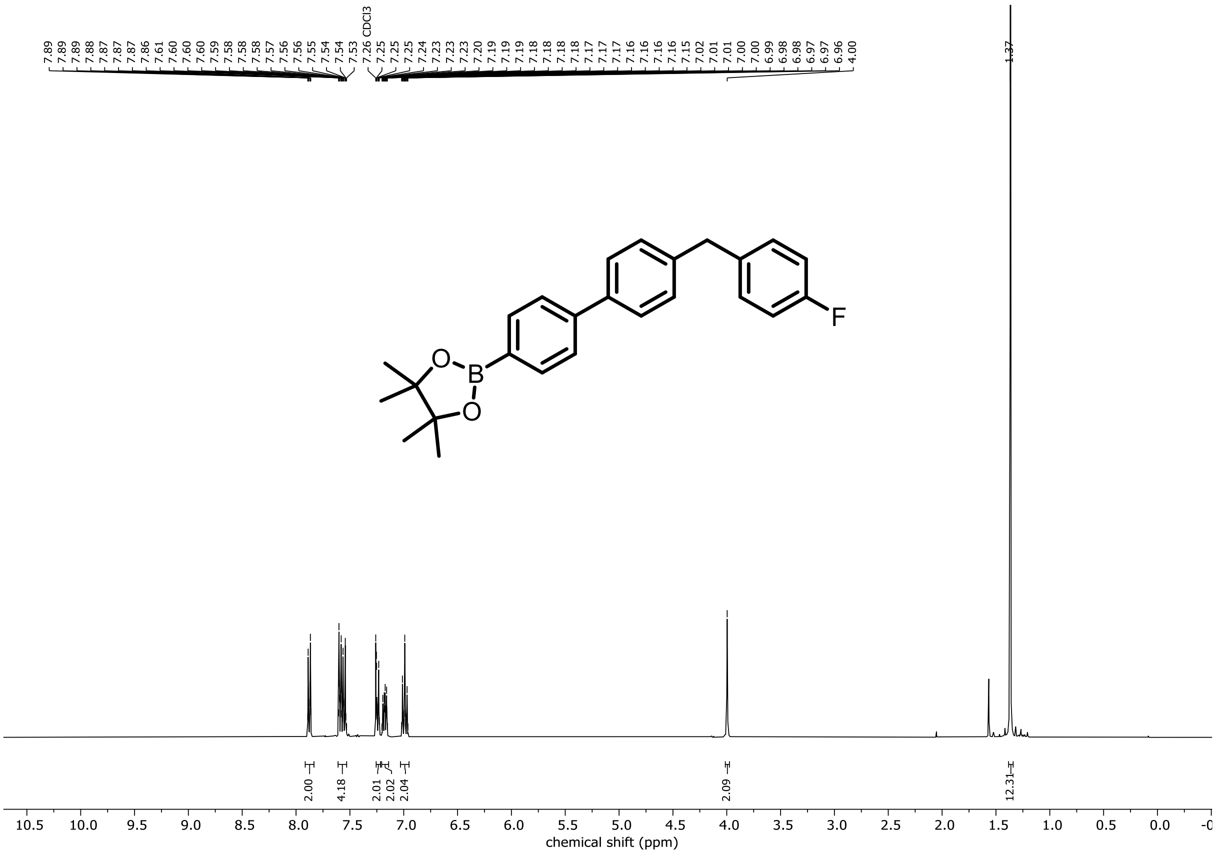

2-(4'-(4-fluorobenzyl)-[1,1'-biphenyl]-4-yl)-4,4,5,5-tetramethyl-1,3,2-dioxaborolane (12) (^{13}C NMR, 101 MHz, $CDCl_3$)

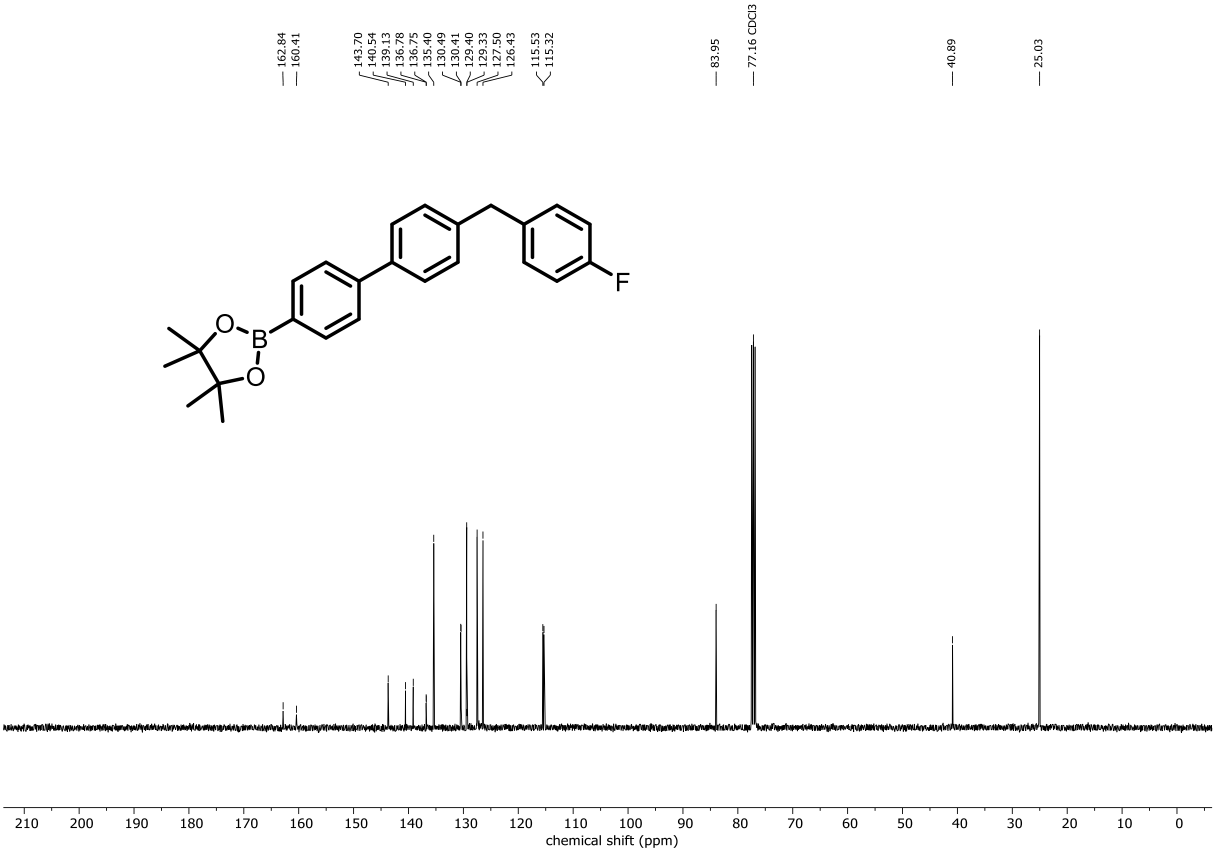

2-(4'-(4-fluorobenzyl)-[1,1'-biphenyl]-4-yl)-4,4,5,5-tetramethyl-1,3,2-dioxaborolane (12) (^{19}F {^{1}H} NMR, 376 MHz, $CDCl_3$)

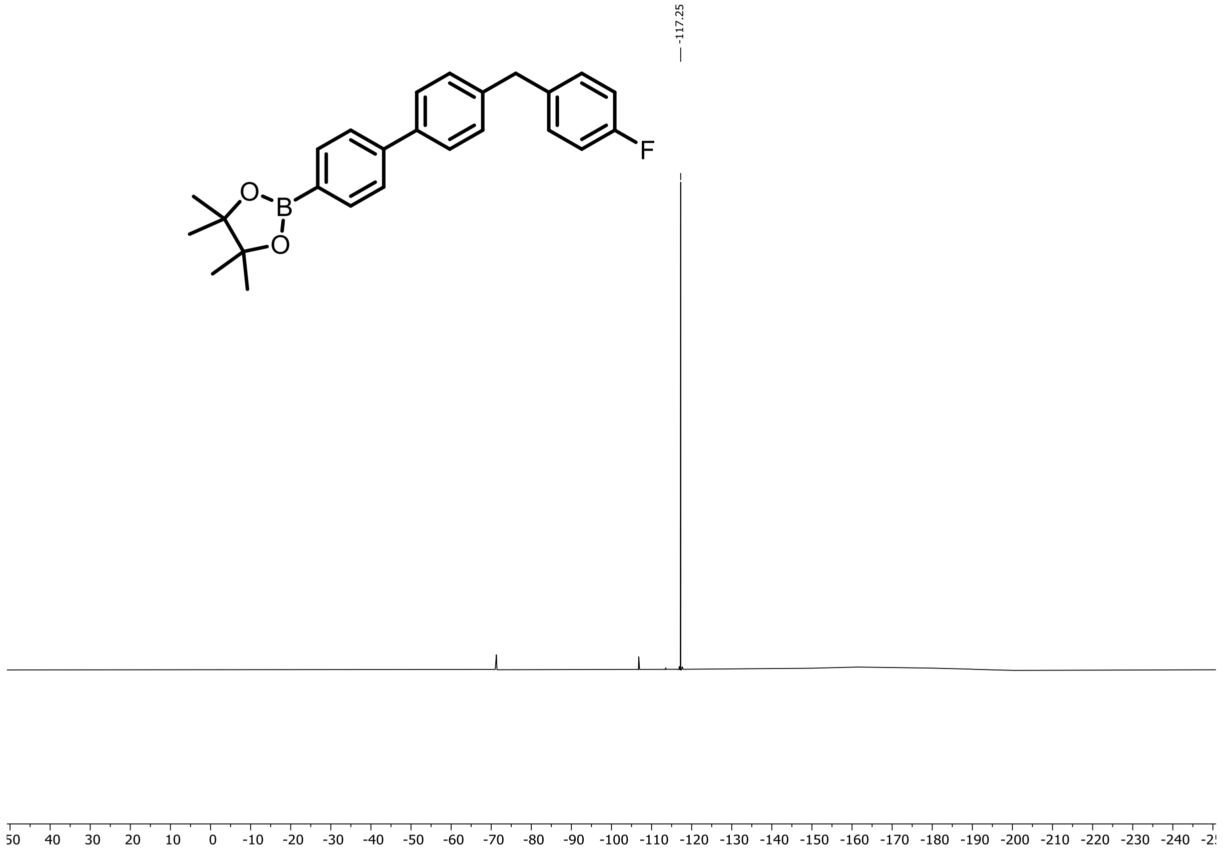

Chloro(4-fluorophenyl)dimethylsilane (S13a) (^{1}H NMR, 400 MHz, $CDCl_3$)

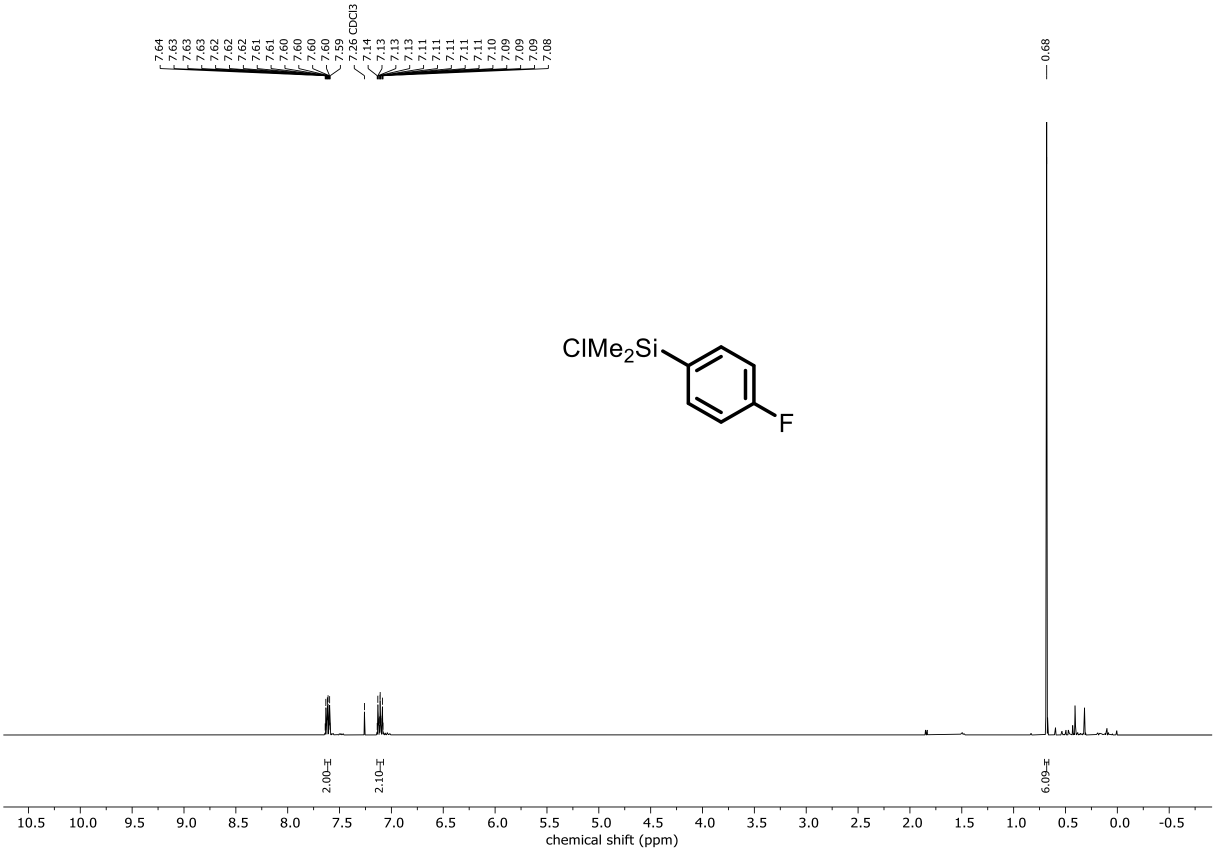

Chloro(4-fluorophenyl)dimethylsilane (S13a) (^{19}F {^{1}H} NMR, 376 MHz, $CDCl_3$)

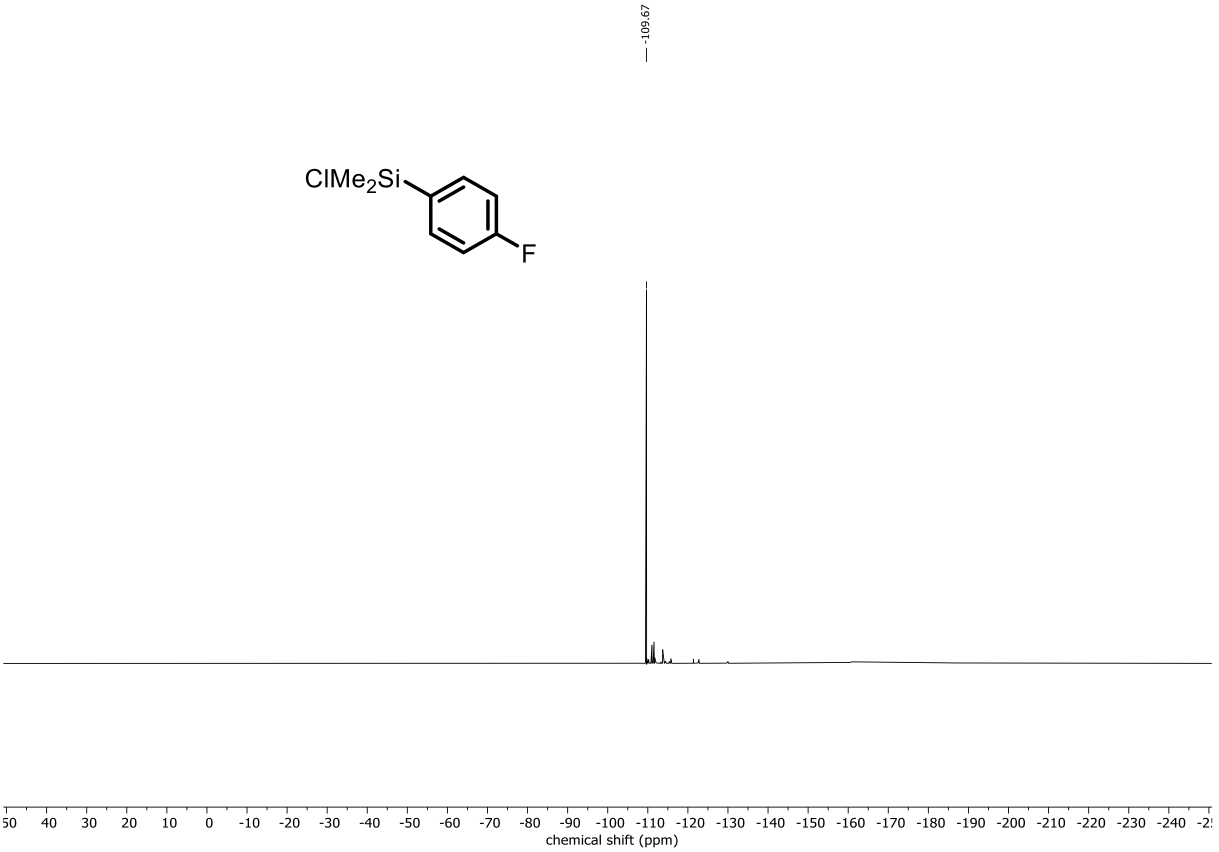

(4-Bromophenyl)(4-fluorophenyl)dimethylsilane (S13b) (^{1}H NMR, 400 MHz, $CDCl_3$)

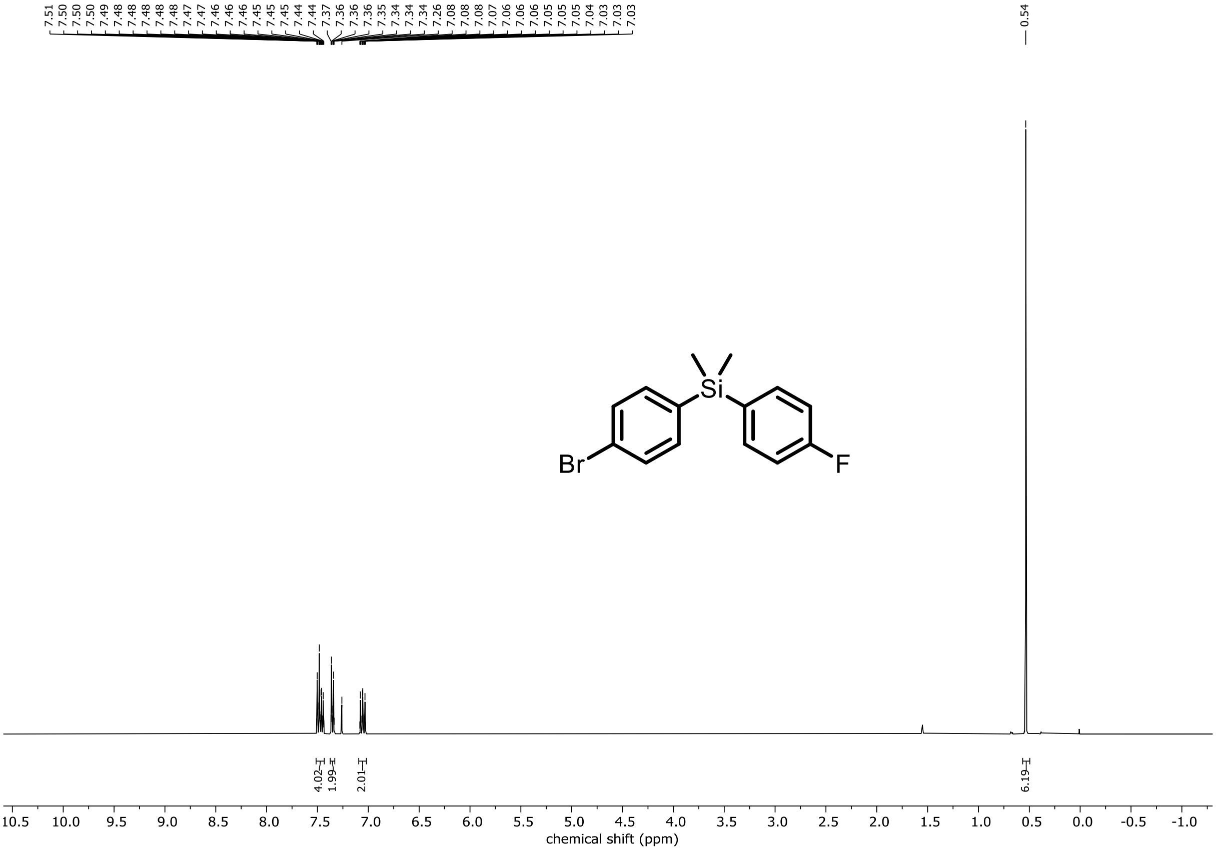

(4-Bromophenyl)(4-fluorophenyl)dimethylsilane (S13b) (^{13}C NMR, 101 MHz, $CDCl_3$)

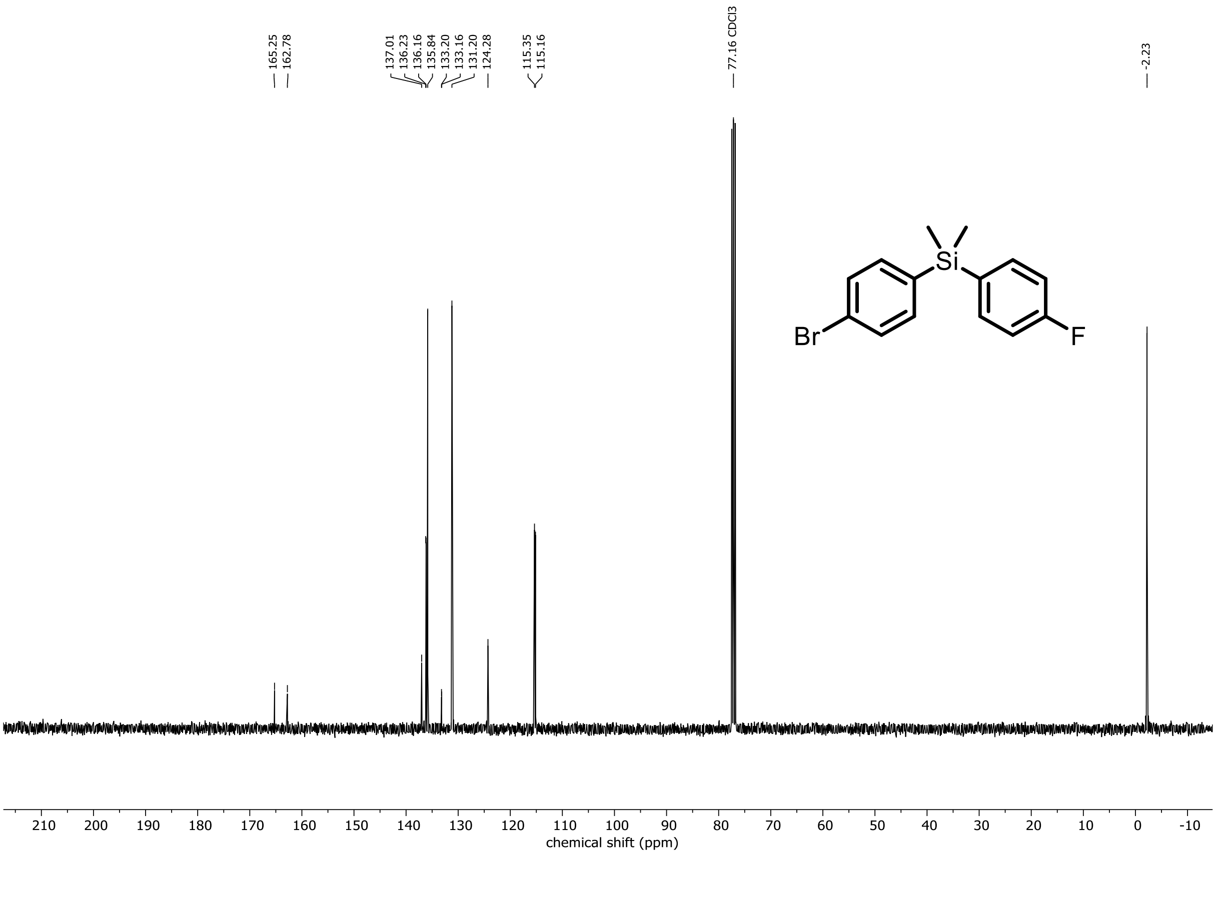

(4-Bromophenyl)(4-fluorophenyl)dimethylsilane (S13b) (^{19}F NMR, 376 MHz, $CDCl_3$)

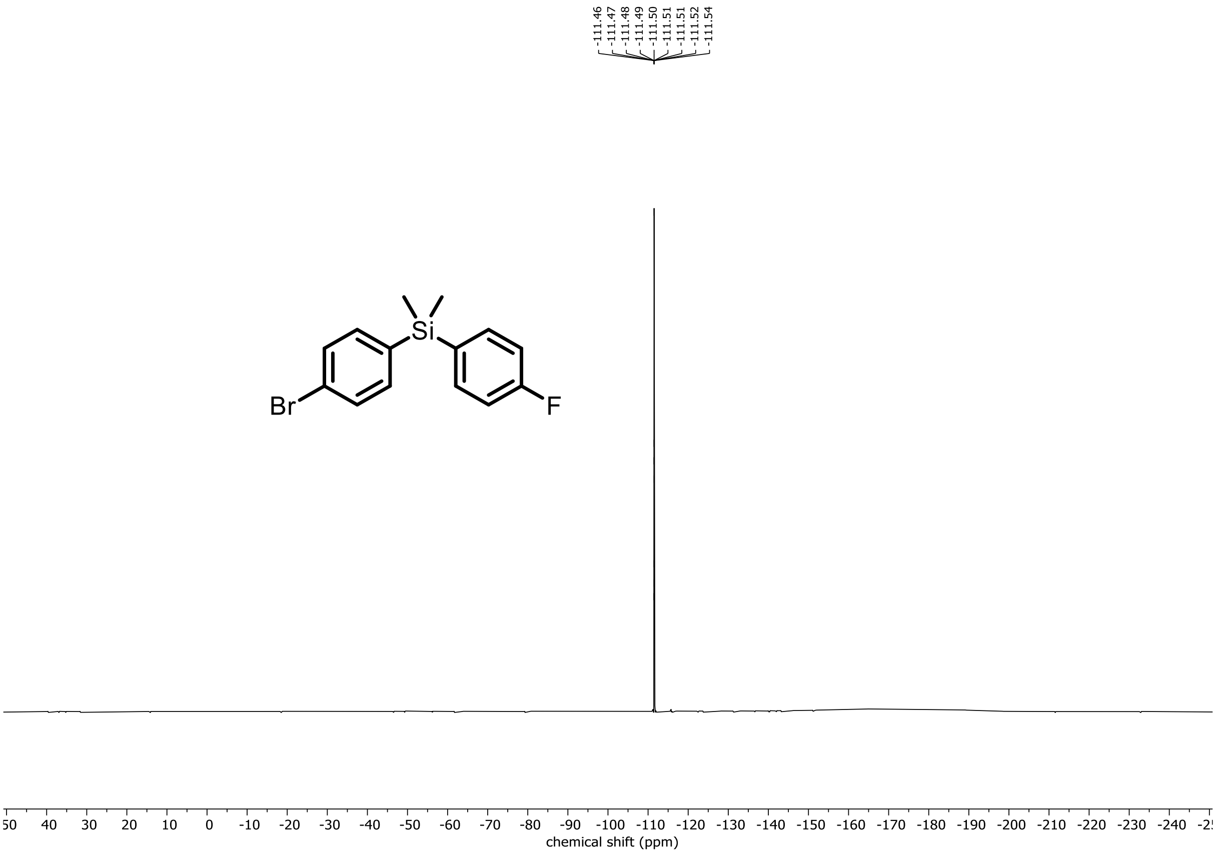

(4-Fluorophenyl)dimethyl(4-(4,4,5,5-tetramethyl-1,3,2-dioxaborolan-2-yl)phenyl)silane (13) (^{1}H NMR, 400 MHz, $CDCl_3$)

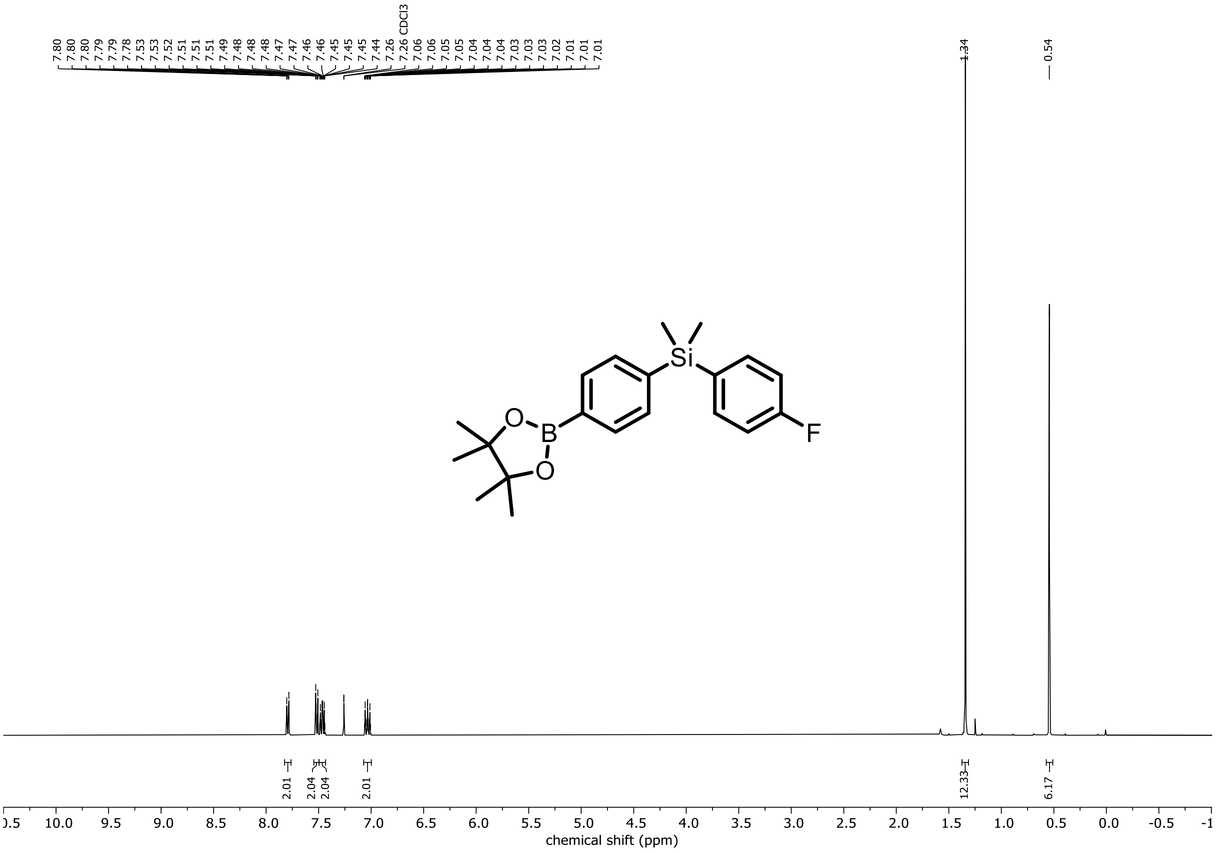

(4-Fluorophenyl)dimethyl(4-(4,4,5,5-tetramethyl-1,3,2-dioxaborolan-2-yl)phenyl)silane (13) (^{13}C NMR, 101 MHz, $CDCl_3$)

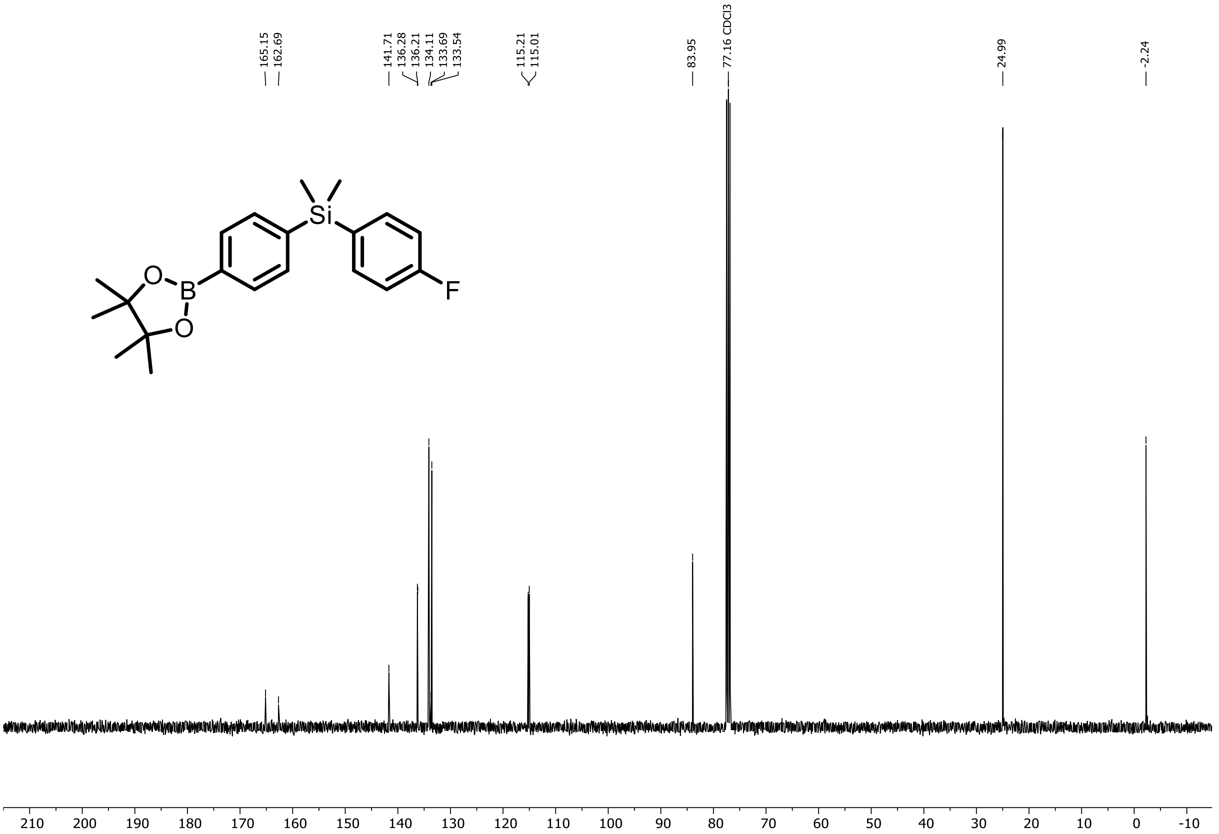

(4-Fluorophenyl)dimethyl(4-(4,4,5,5-tetramethyl-1,3,2-dioxaborolan-2-yl)phenyl)silane (13) (^{19}F NMR, 376 MHz, $CDCl_3$)

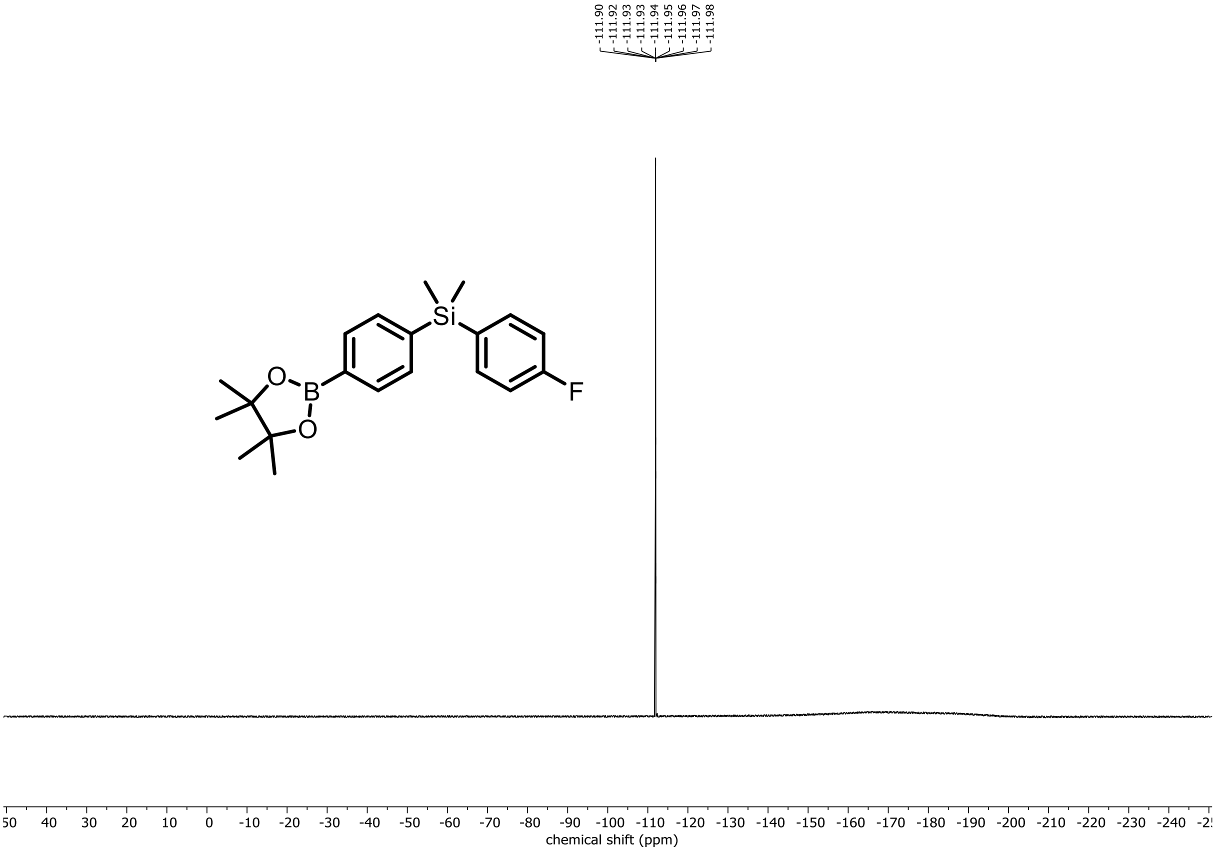

4-Chloro-*N*-(4-fluorophenyl)-*N*-methylaniline (S14a) (^{1}H NMR, 400 MHz, $CDCl_3$)

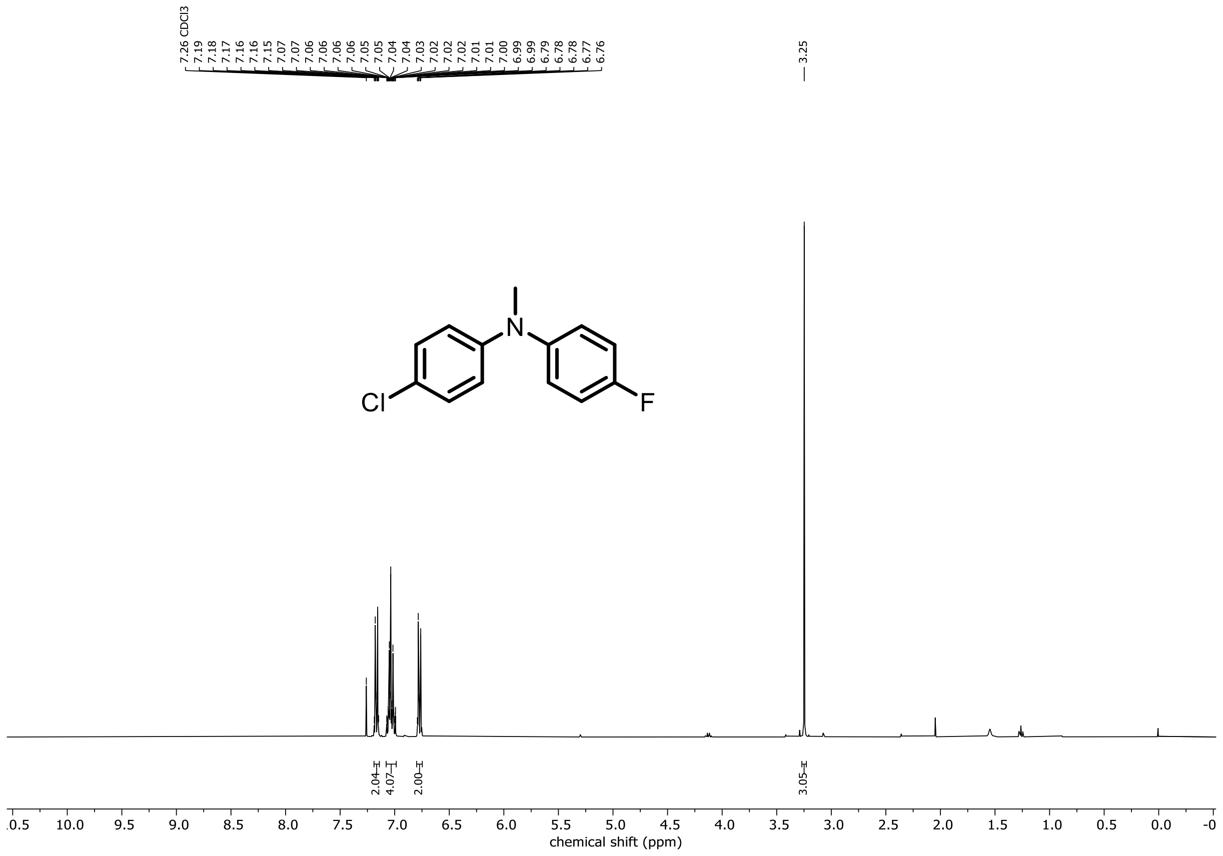

4-Chloro-*N*-(4-fluorophenyl)-*N*-methylaniline (S14a) (^{13}C NMR, 101 MHz, $CDCl_3$)

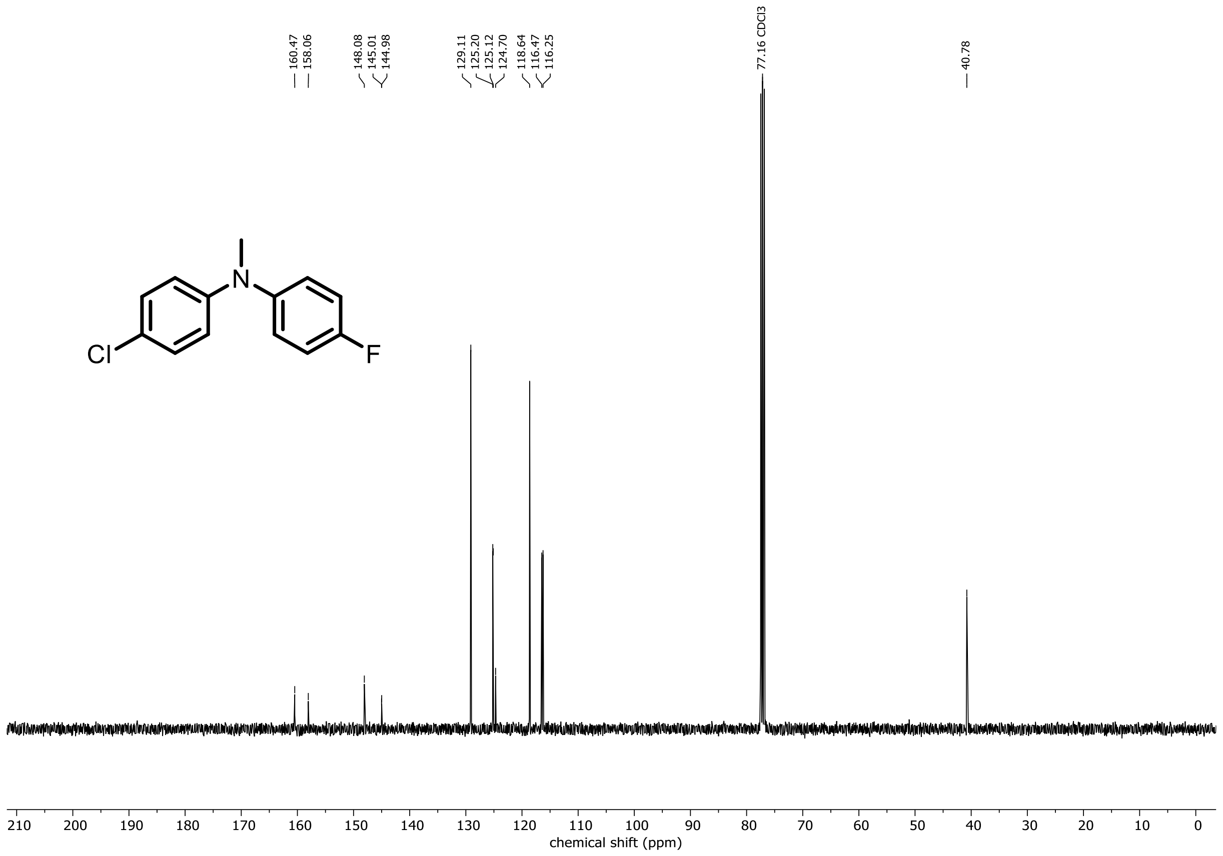

4-Chloro-*N*-(4-fluorophenyl)-*N*-methylaniline (S14a) (^{19}F NMR, 376 MHz, $CDCl_3$)

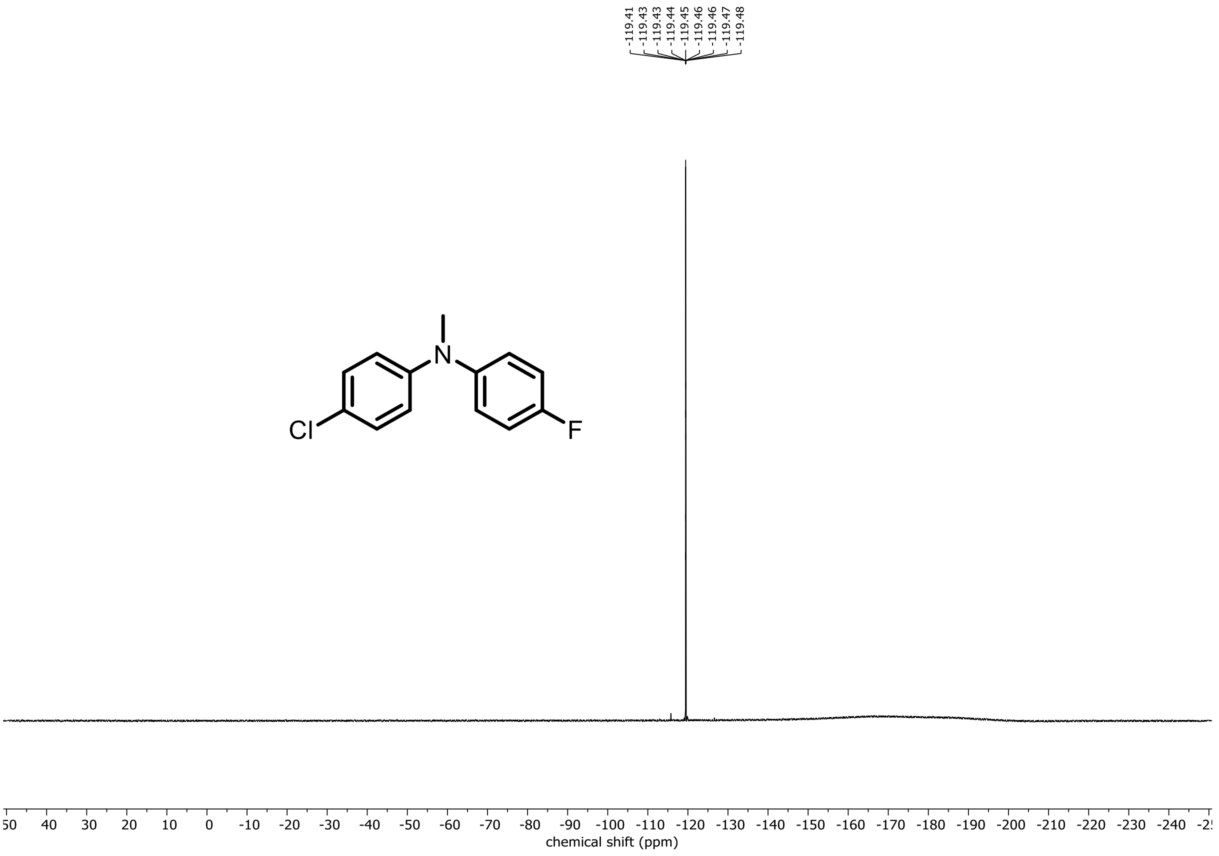

4-Fluoro-*N*-methyl-*N*-(4-(4,4,5,5-tetramethyl-1,3,2-dioxaborolan-2-yl)phenyl)aniline (14) (^{1}H NMR, 400 MHz, $CDCl_3$)

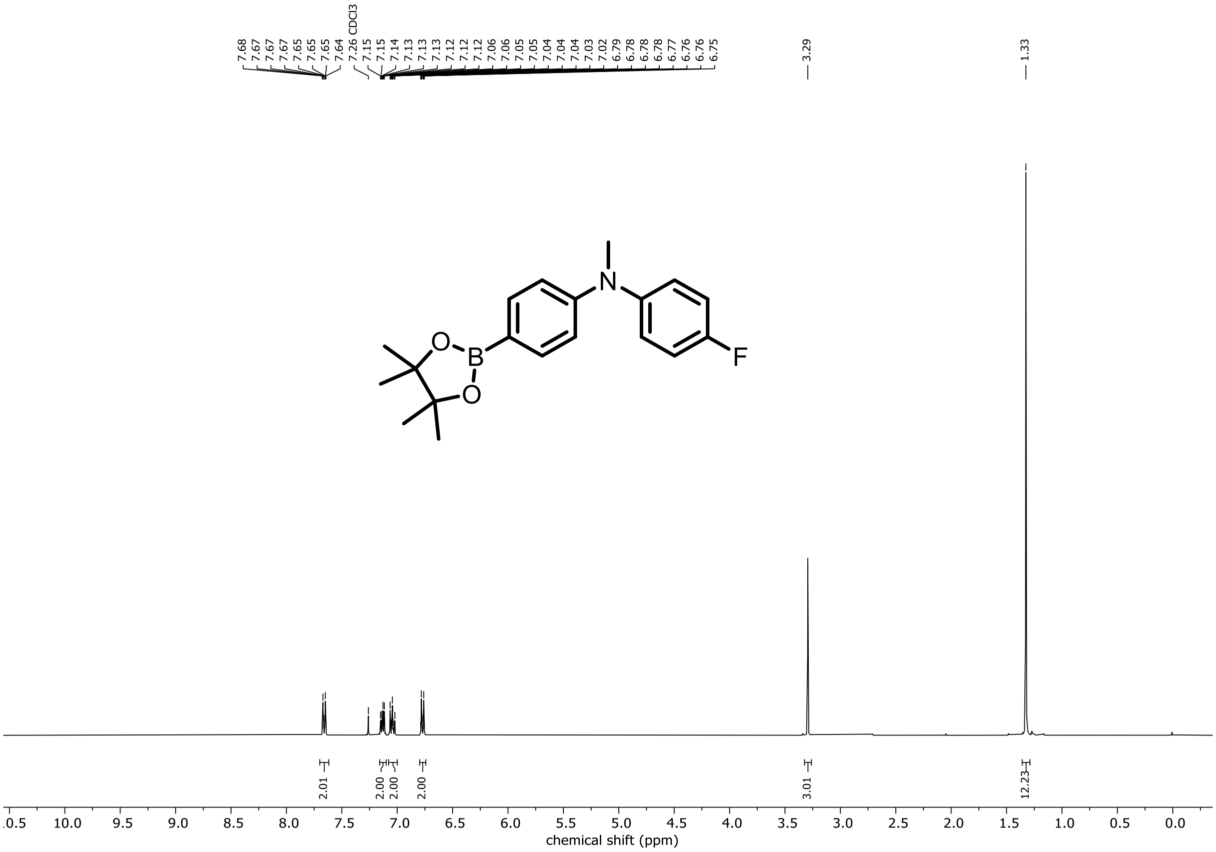

4-Fluoro-*N*-methyl-*N*-(4-(4,4,5,5-tetramethyl-1,3,2-dioxaborolan-2-yl)phenyl)aniline (14) (^{13}C NMR, 101 MHz, $CDCl_3$)

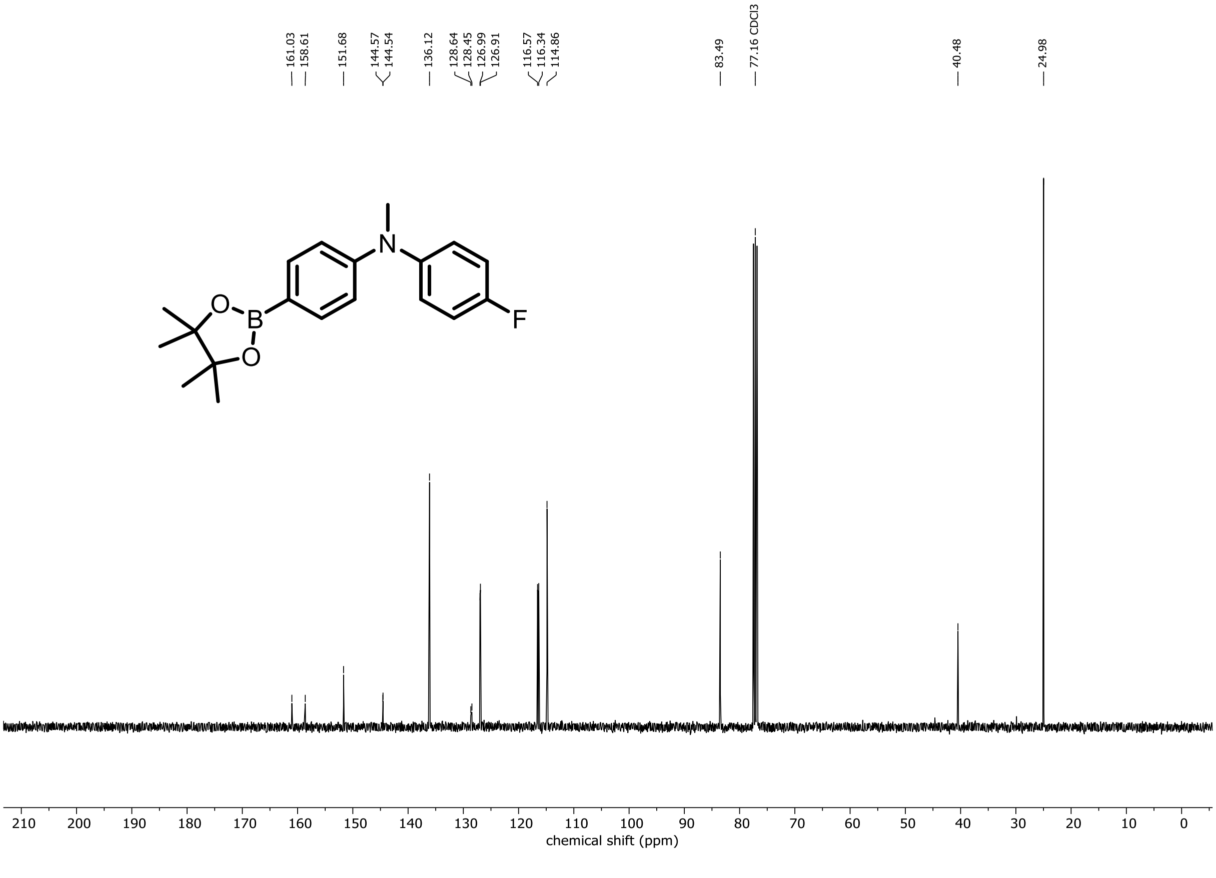

4-Fluoro-*N*-methyl-*N*-(4-(4,4,5,5-tetramethyl-1,3,2-dioxaborolan-2-yl)phenyl)aniline (14) (^{19}F NMR, 376 MHz, $CDCl_3$)

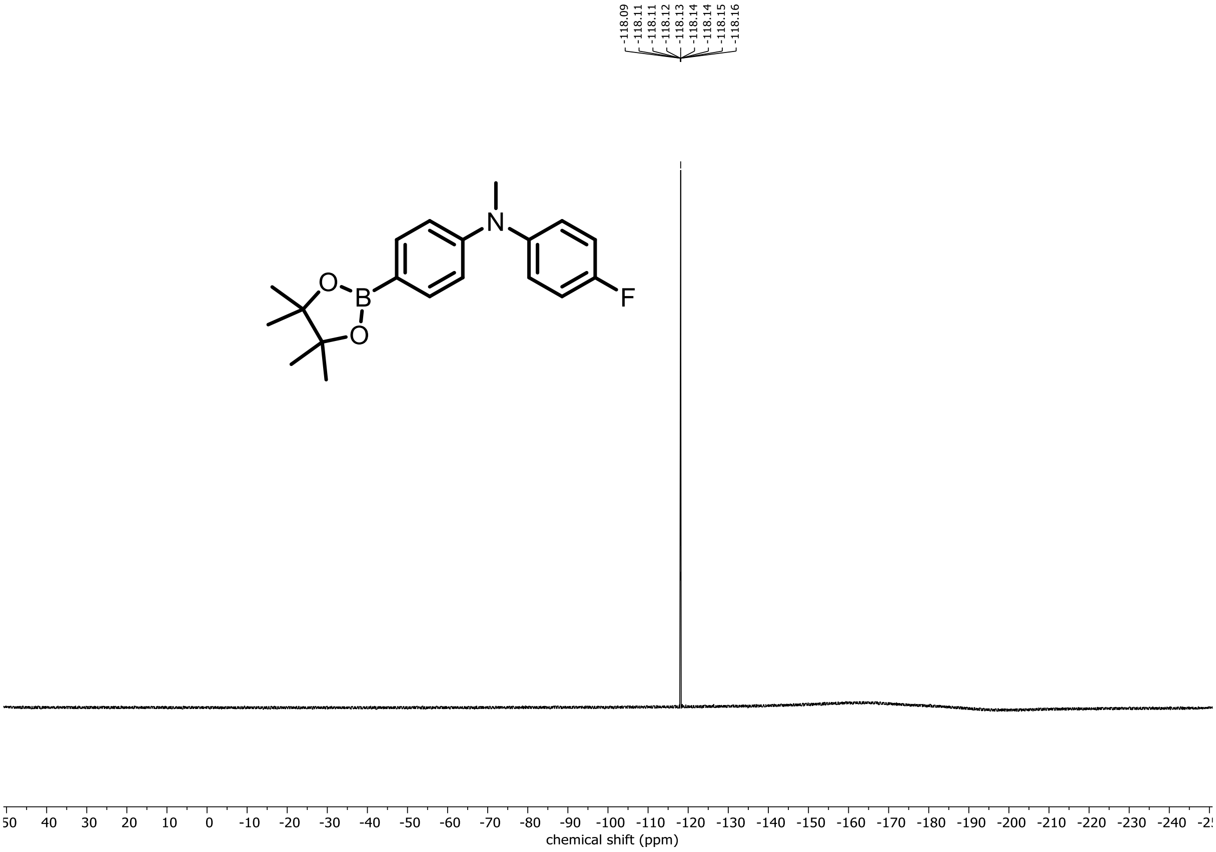

1-Bromo-4-(4-fluorophenoxy)benzene (S15a) (^{1}H NMR, 400 MHz, $CDCl_3$)

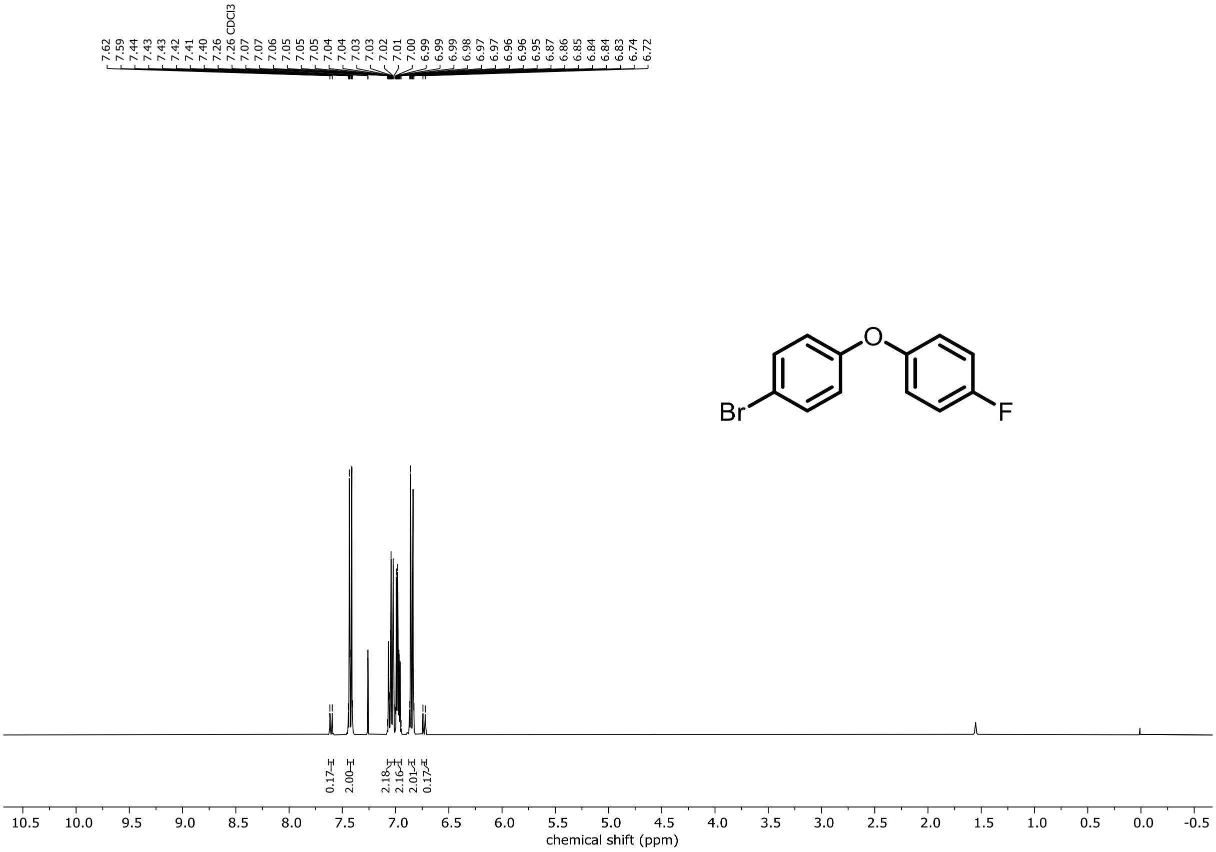

1-Bromo-4-(4-fluorophenoxy)benzene (S15a) (^{13}C NMR, 101 MHz, $CDCl_3$)

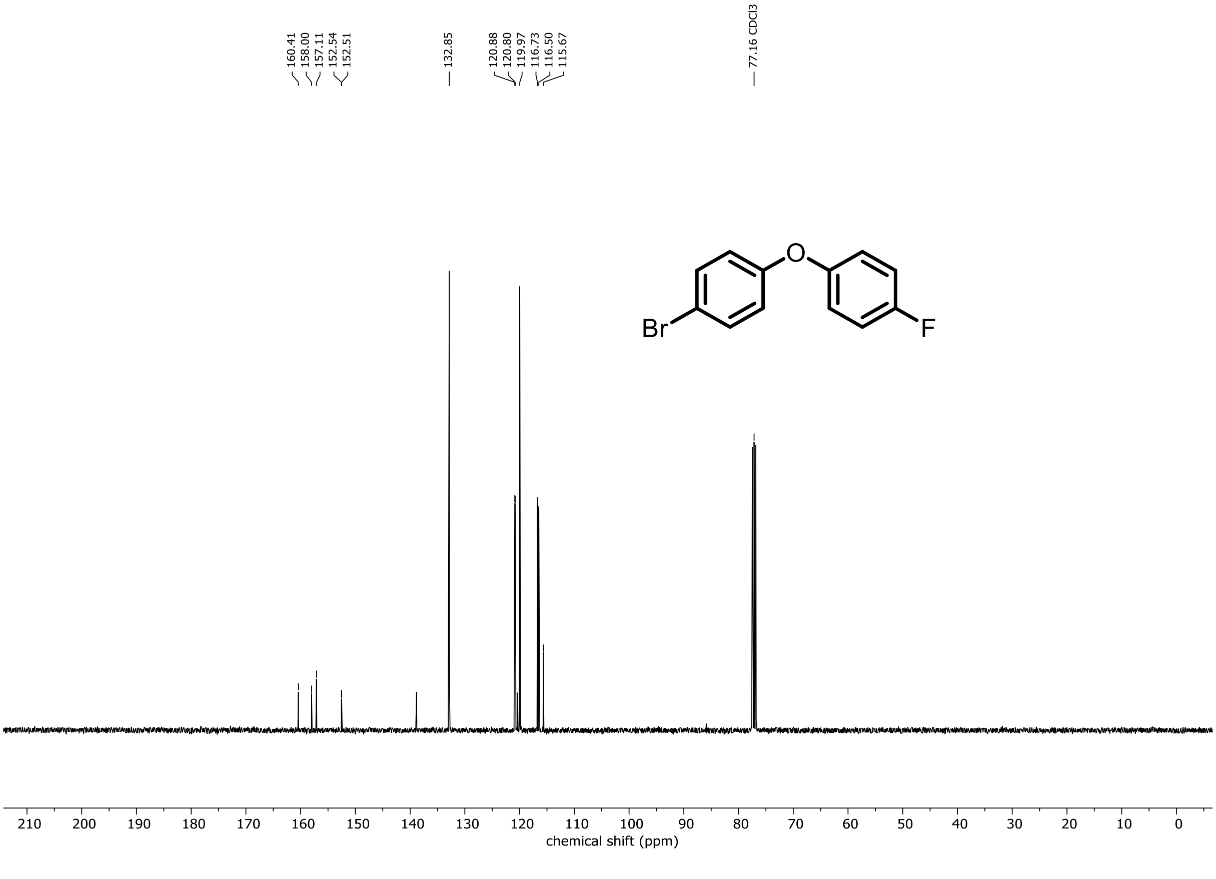

1-Bromo-4-(4-fluorophenoxy)benzene (S15a) (^{19}F NMR, 376 MHz, $CDCl_3$)

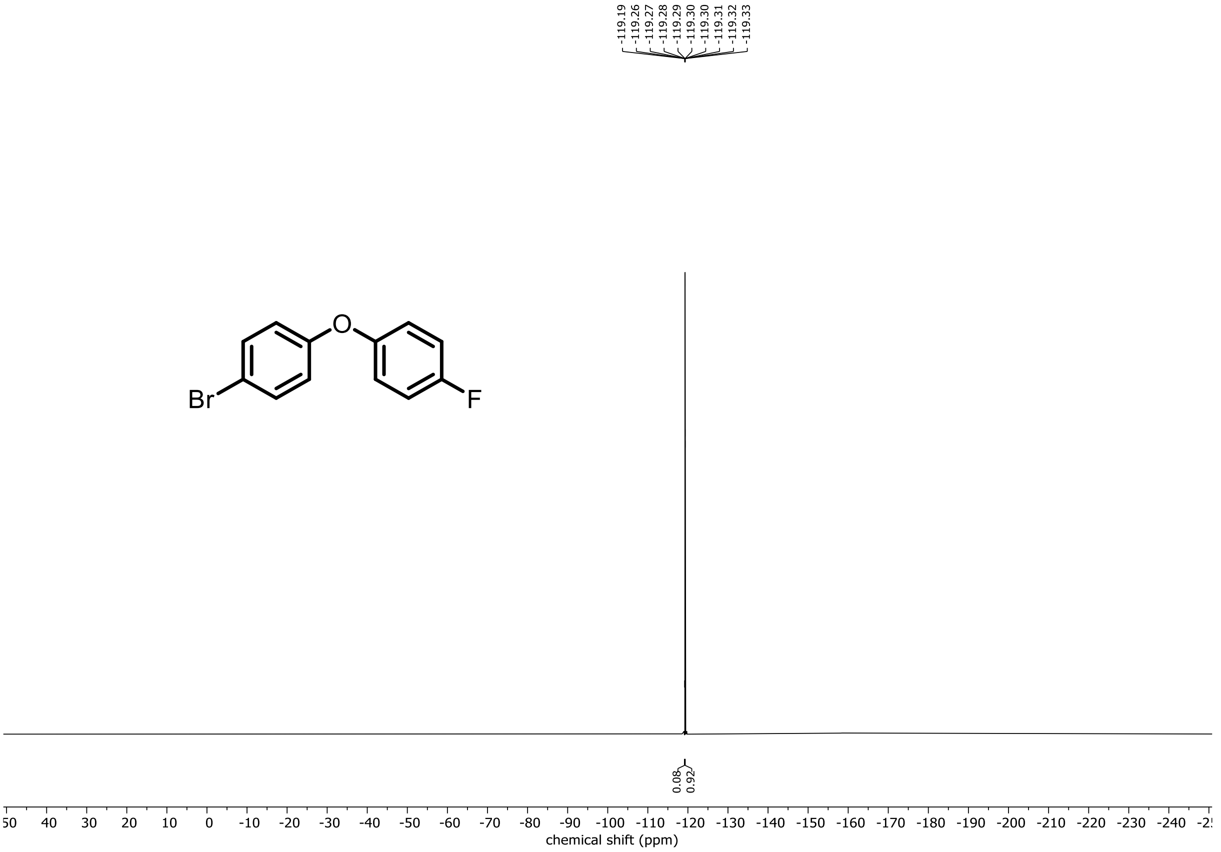

2-(4-(4-fluorophenoxy)phenyl)-4,4,5,5-tetramethyl-1,3,2-dioxaborolane (15) (^{1}H NMR, 400 MHz, $CDCl_3$)

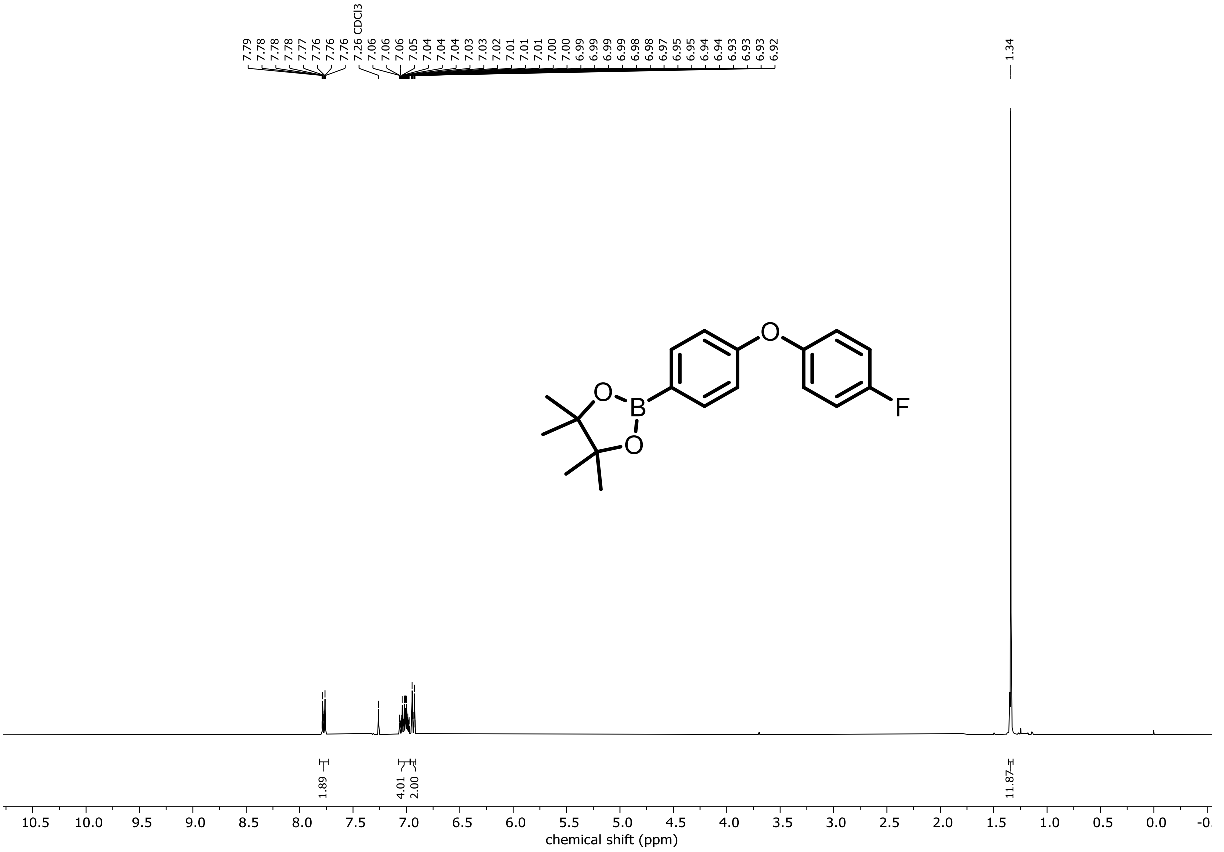

2-(4-(4-fluorophenoxy)phenyl)-4,4,5,5-tetramethyl-1,3,2-dioxaborolane (15) (^{13}C NMR, 101 MHz, $CDCl_3$)

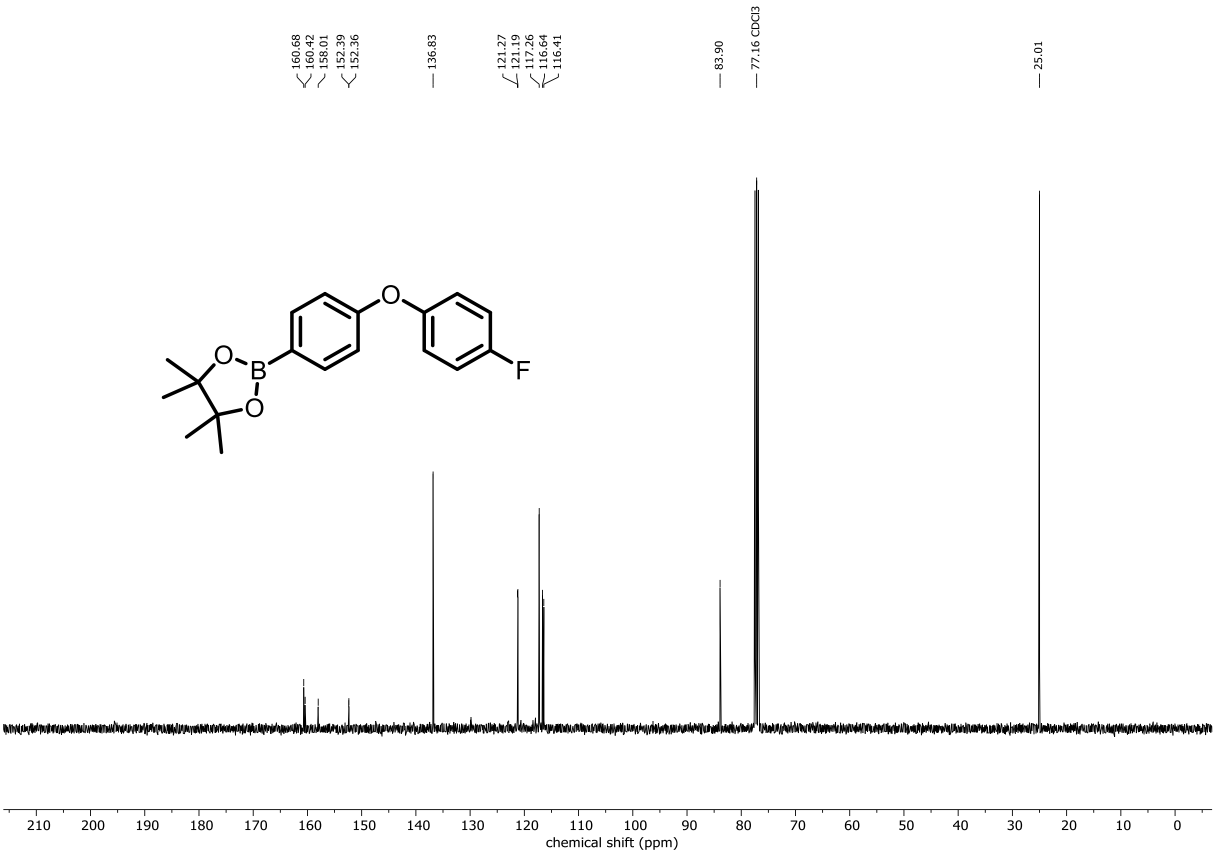

2-(4-(4-fluorophenoxy)phenyl)-4,4,5,5-tetramethyl-1,3,2-dioxaborolane (15) (^{19}F NMR, 376 MHz, $CDCl_3$)

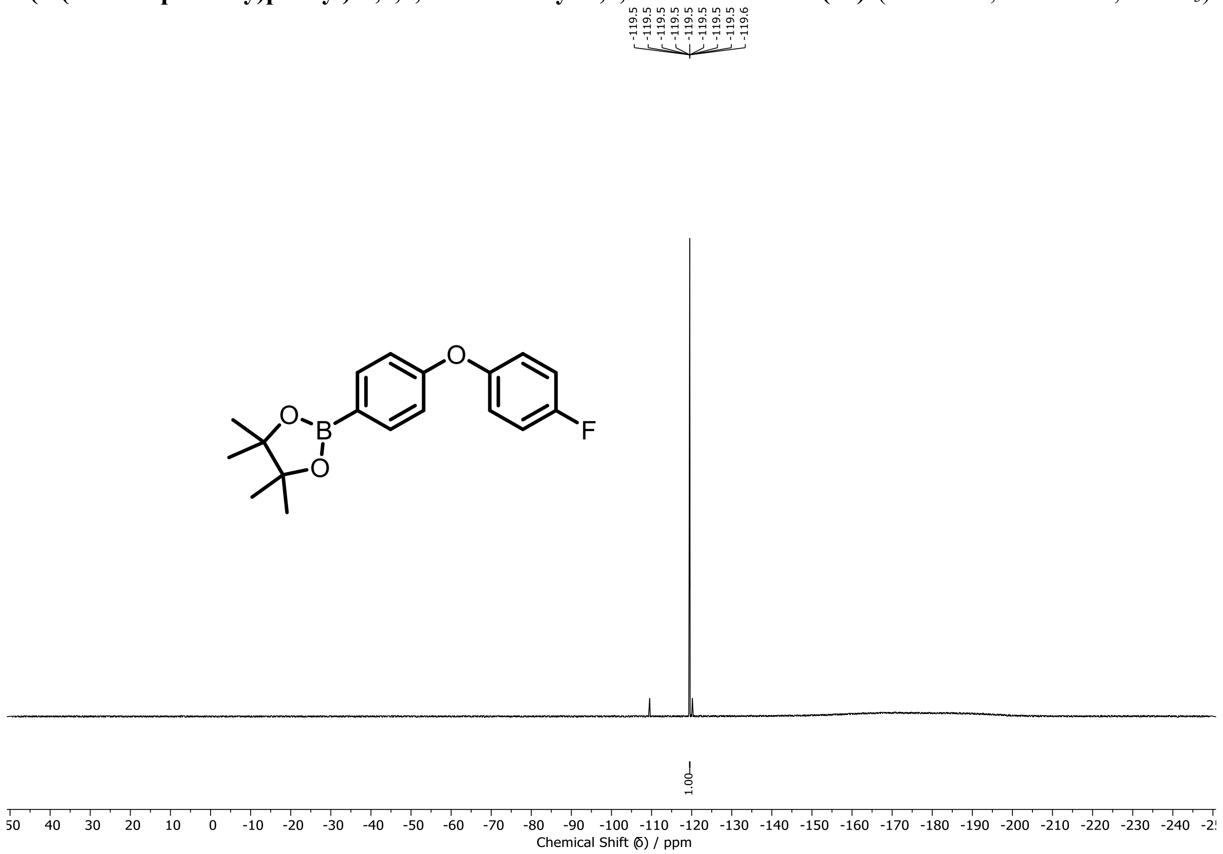

***N*-(4-Bromo-phenyl)-*N'*-phenyl-benzene-1,4-diamine (S16a)** (^{1}H NMR, 400 MHz, $(CD_3)_2CO$)

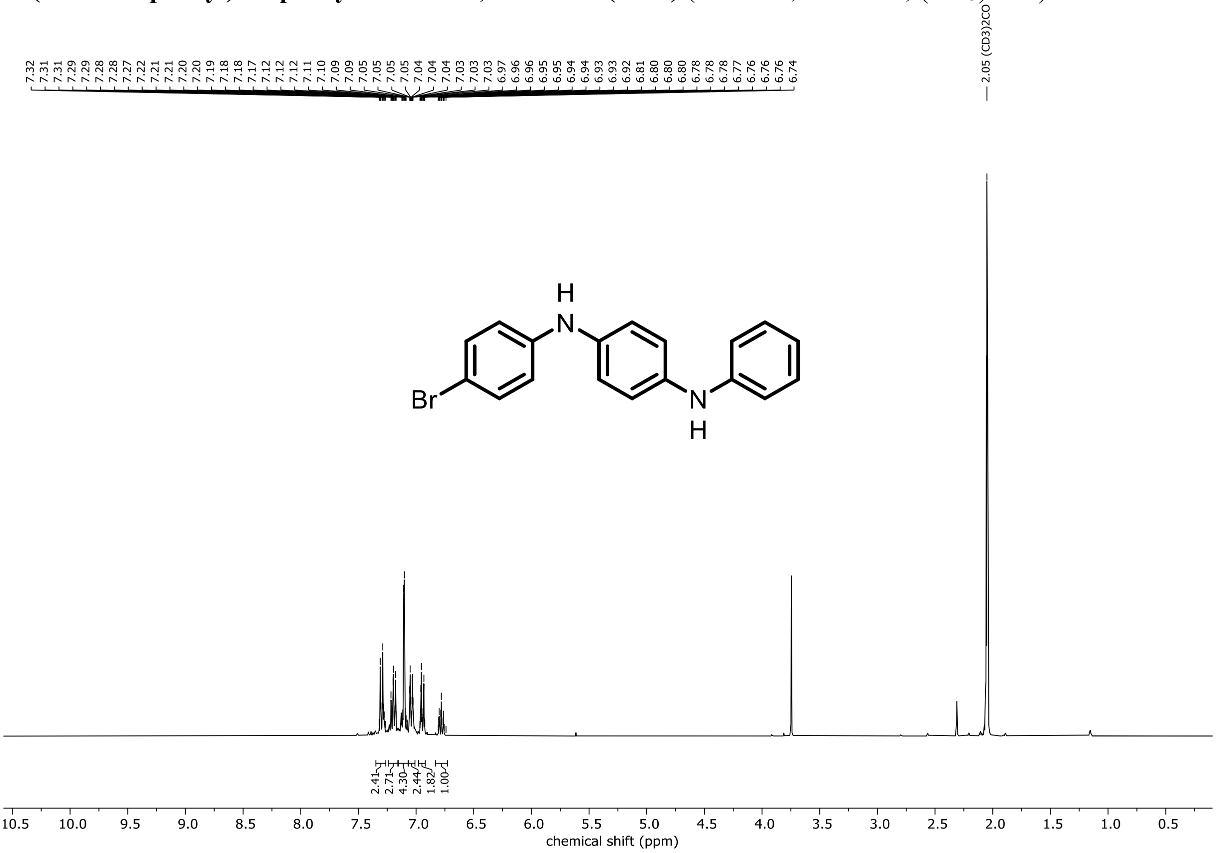

***N*-(4-Bromo-phenyl)-*N'*-phenyl-benzene-1,4-diamine (S16a)** (^{13}C NMR, 101 MHz, $(CD_3)_2CO$)

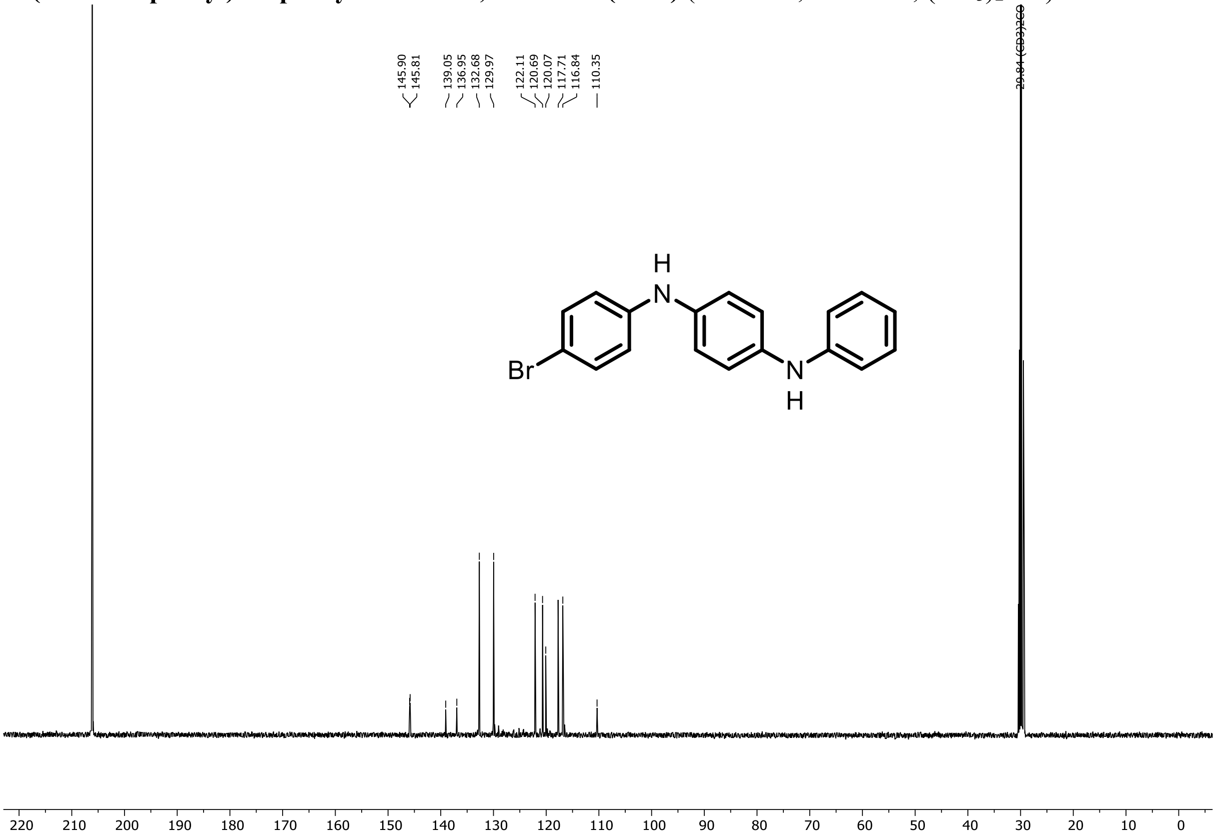

***N*-(4-Bromo-phenyl)-*N,N′*-dimethyl-*N′*-phenyl-benzene-1,4-diamine (S16b)** (^{1}H NMR, 400 MHz, $(CD_3)_2CO$)

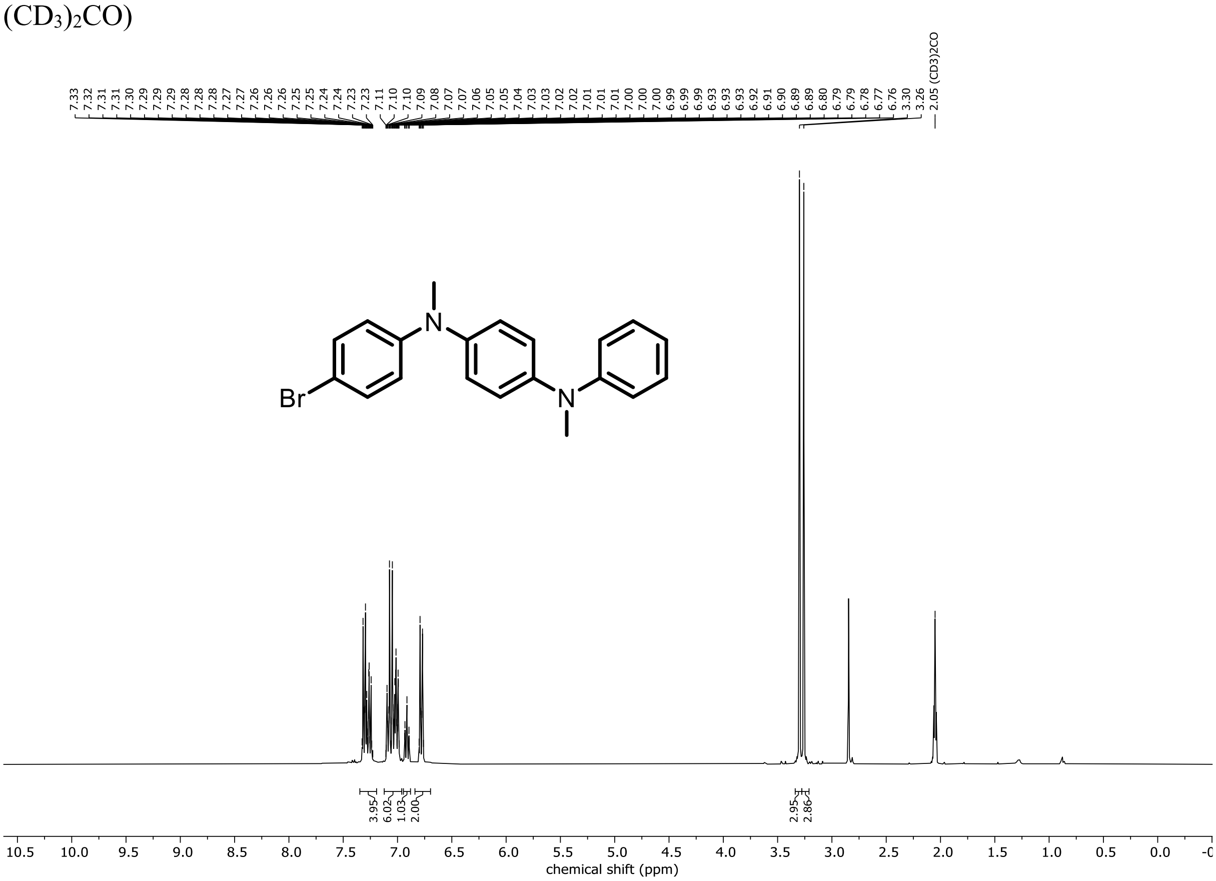

***N*-(4-Bromo-phenyl)-*N,N′*-dimethyl-*N′*-phenyl-benzene-1,4-diamine (S16b)** (^{13}C NMR, 101 MHz, $(CD_3)_2CO$)

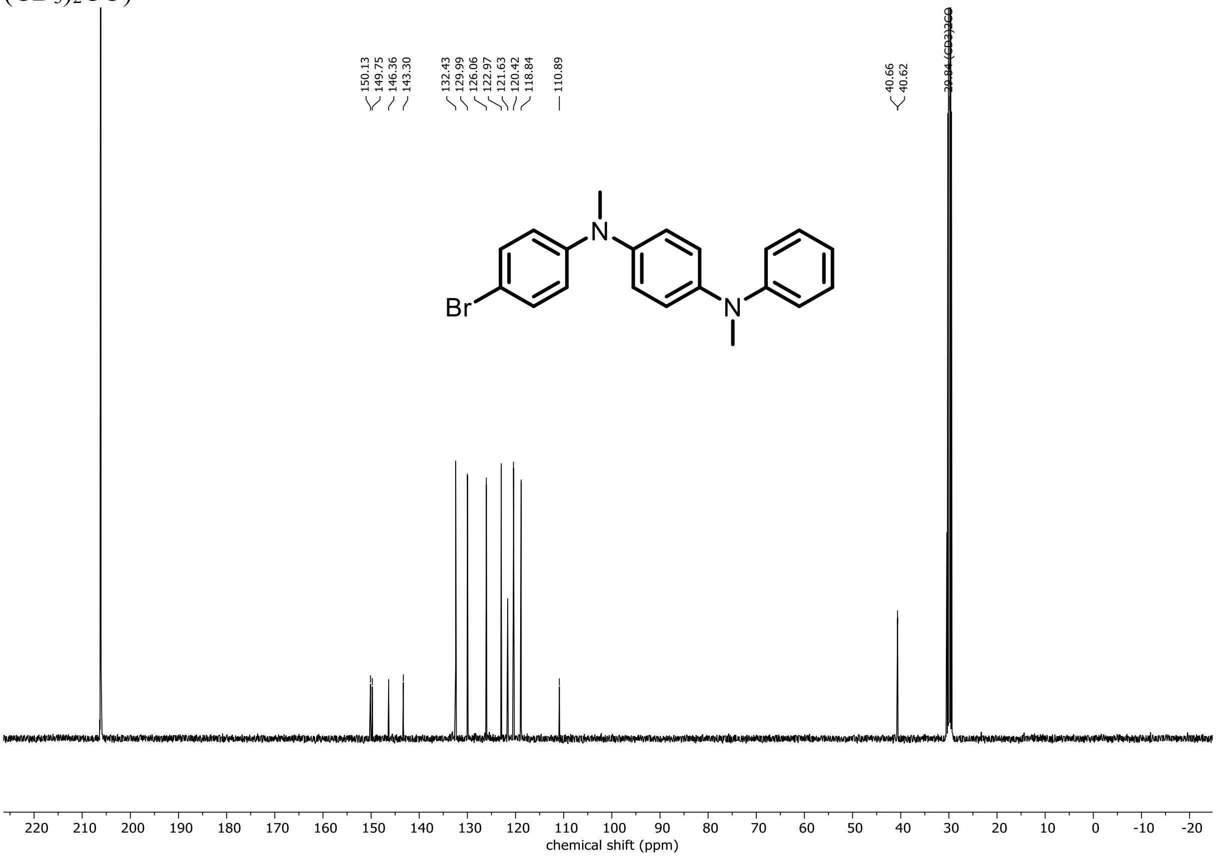

***N*[1],*N*[4]-dimethyl-*N*[1]-phenyl-*N*[4]-(4-(4,4,5,5-tetramethyl-1,3,2-dioxaborolan-2-yl)phenyl)benzene-1,4-diamine (16)** (^{1}H NMR, 400 MHz, C_6D_6)

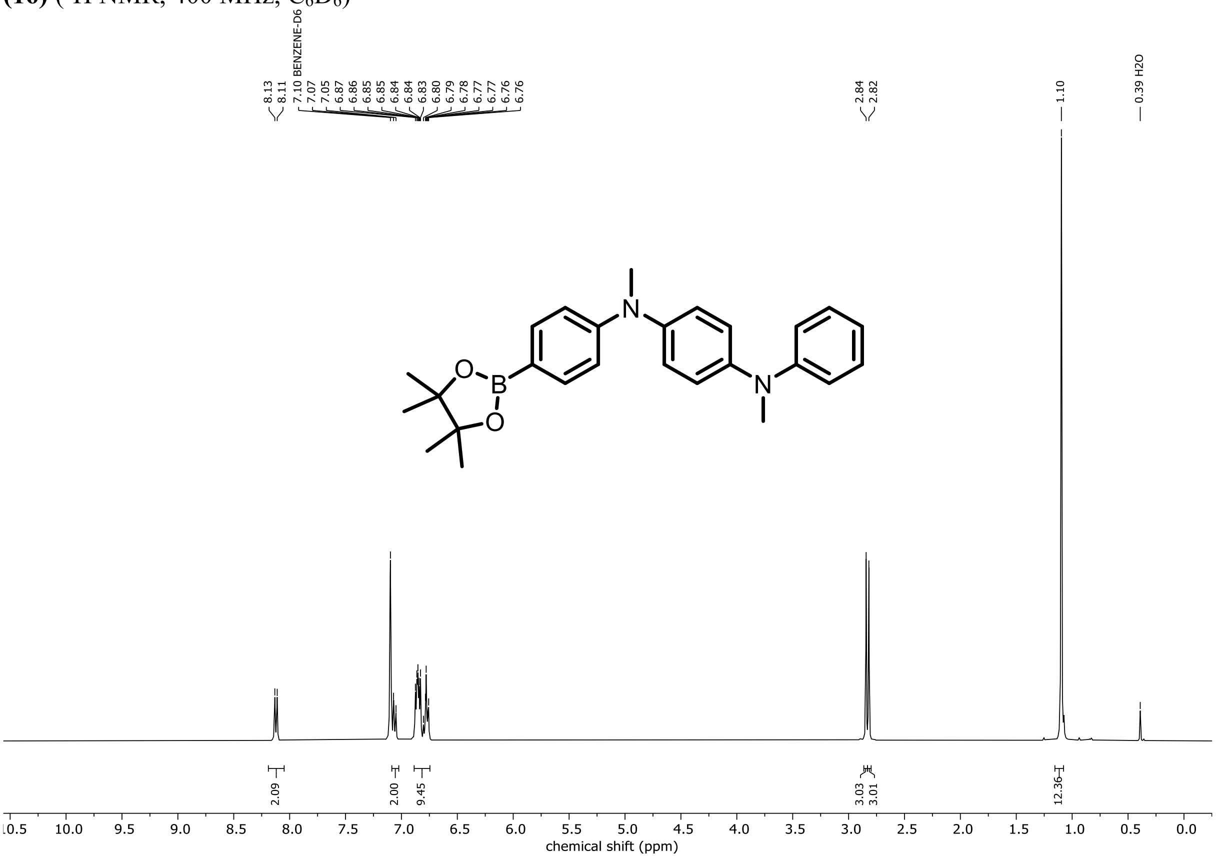

***N*[1],*N*[4]-dimethyl-*N*[1]-phenyl-*N*[4]-(4-(4,4,5,5-tetramethyl-1,3,2-dioxaborolan-2-yl)phenyl)benzene-1,4-diamine (16)** (^{13}C NMR, 101 MHz, MHz, C_6D_6)

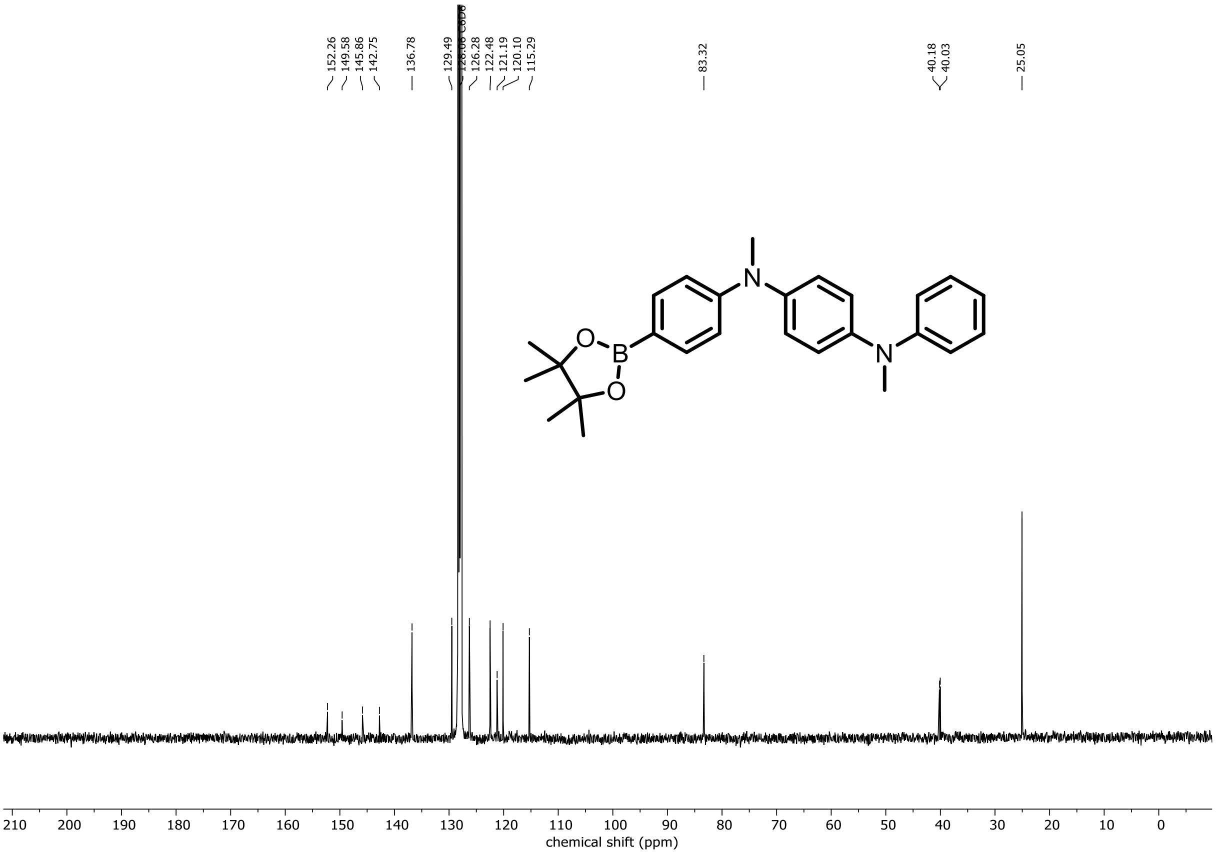

***N¹,N⁴*-bis(4-bromophenyl)benzene-1,4-diamine (S17a)** (^{1}H NMR, 400 MHz, $(CD_3)_2CO$)

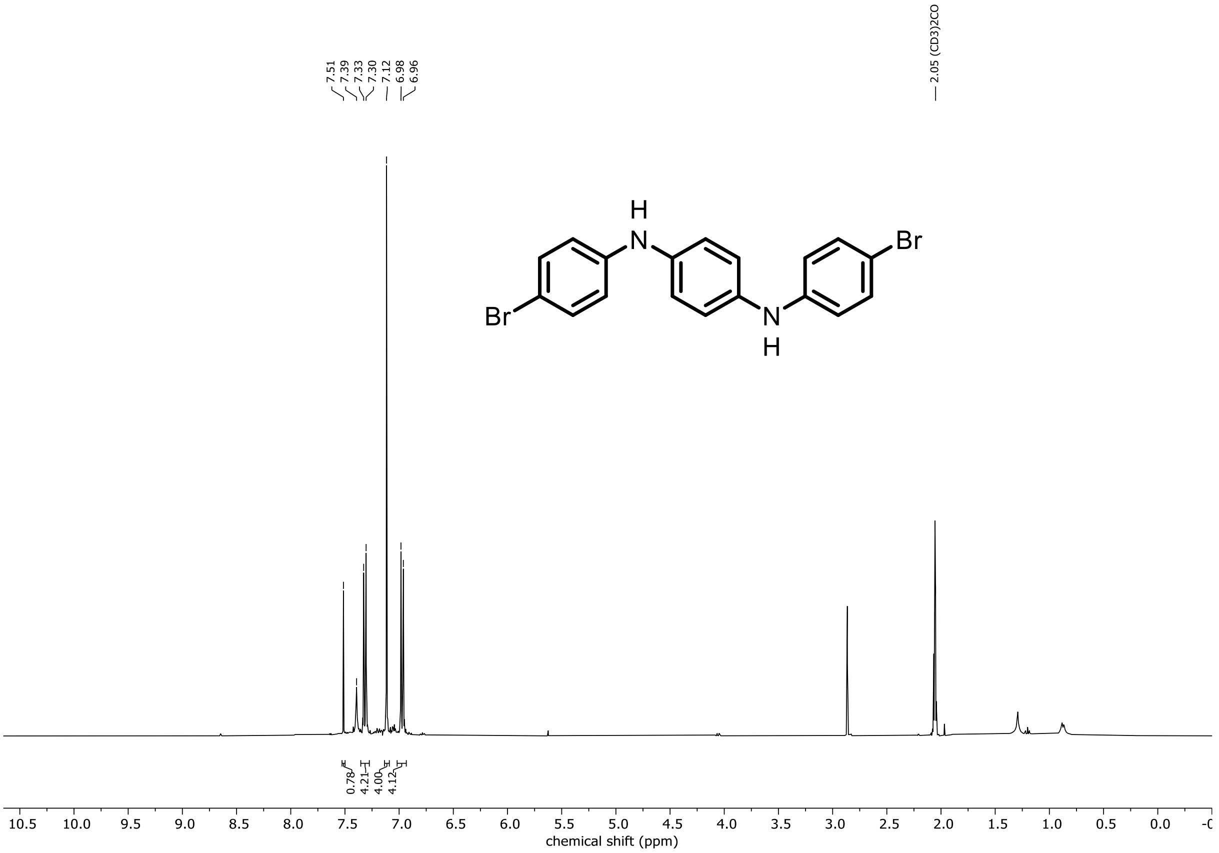

***N¹,N⁴*-bis(4-bromophenyl)benzene-1,4-diamine (S17a)** (^{13}C NMR, 101 MHz, MHz, $(CD_3)_2CO$)

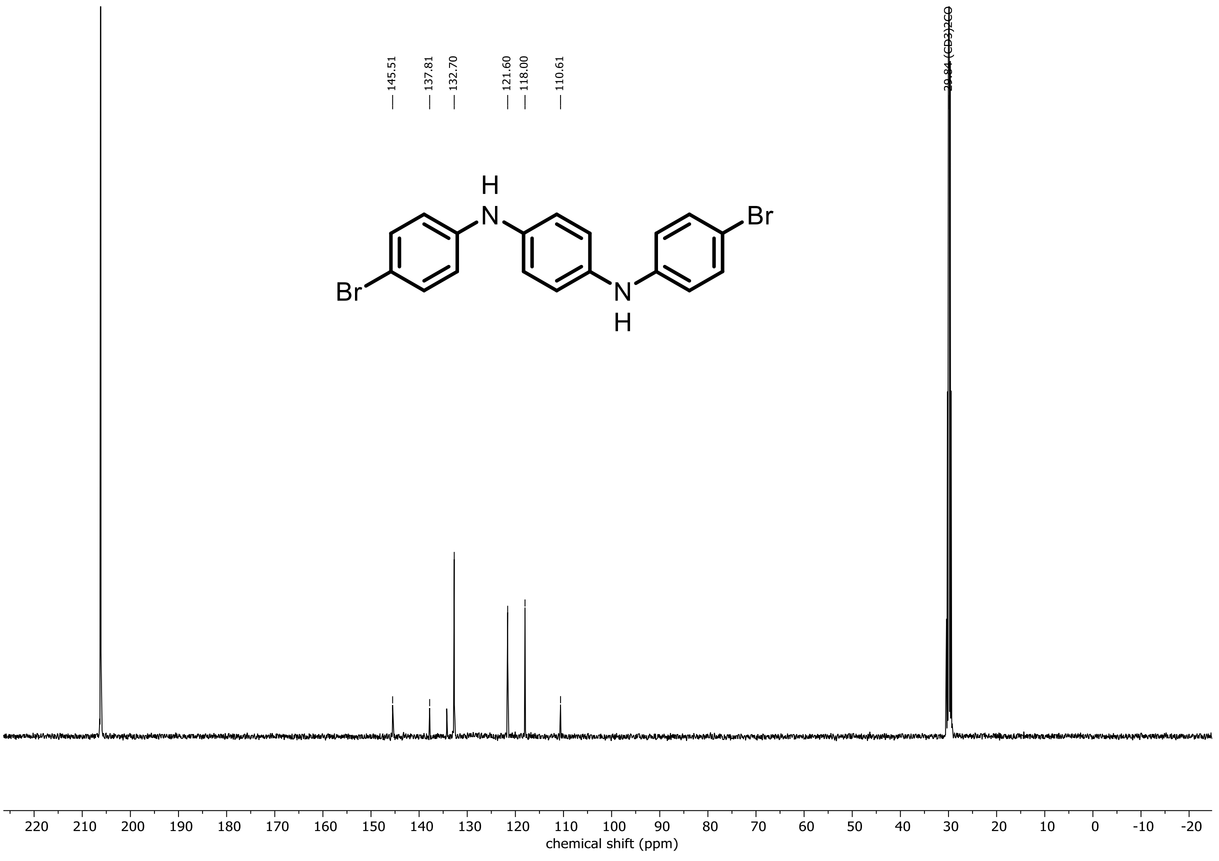

***N*[1],*N*[4]-bis(4-bromophenyl)-*N*[1],*N*[4]-dimethylbenzene-1,4-diamine (S17b)** (^{1}H NMR, 400 MHz, $(CD_3)_2CO$)

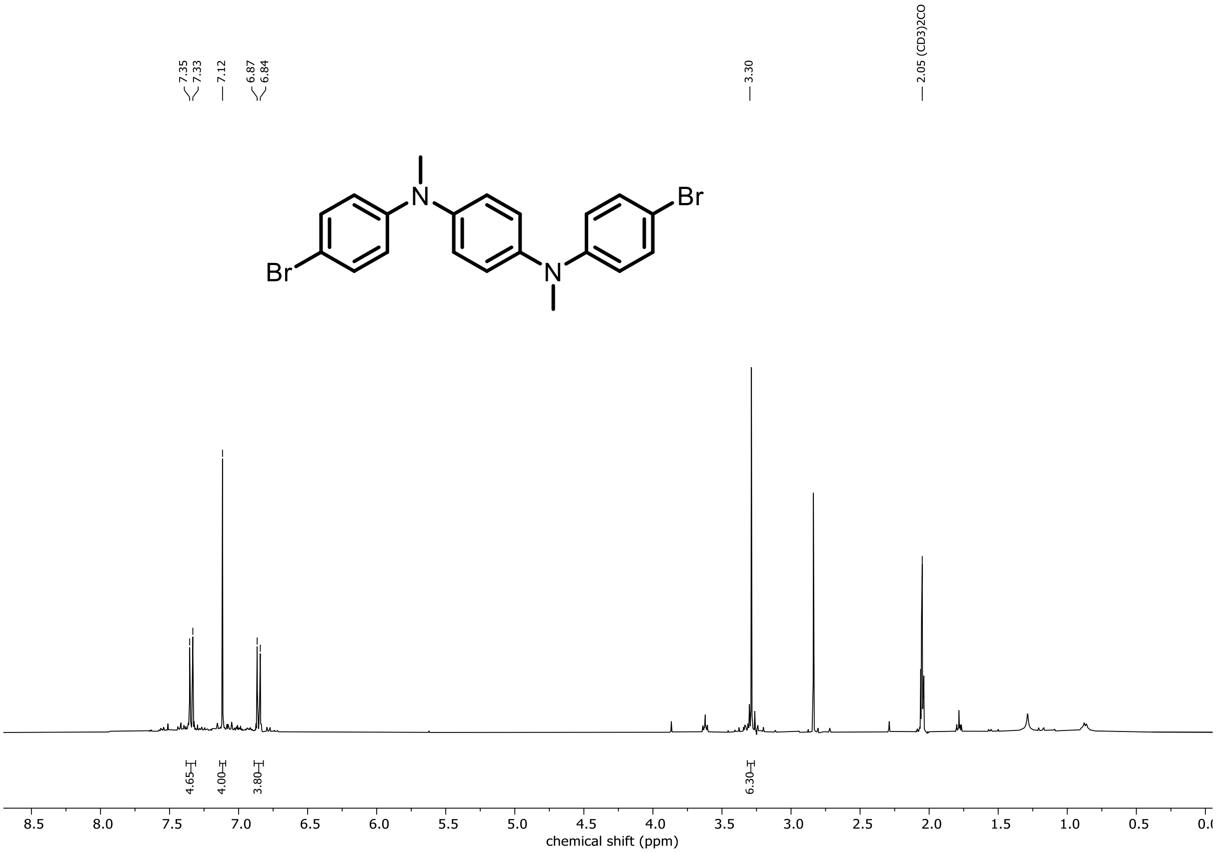

***N*[1],*N*[4]-bis(4-bromophenyl)-*N*[1],*N*[4]-dimethylbenzene-1,4-diamine (S17b)** (^{13}C NMR, 101 MHz, MHz, $(CD_3)_2CO$)

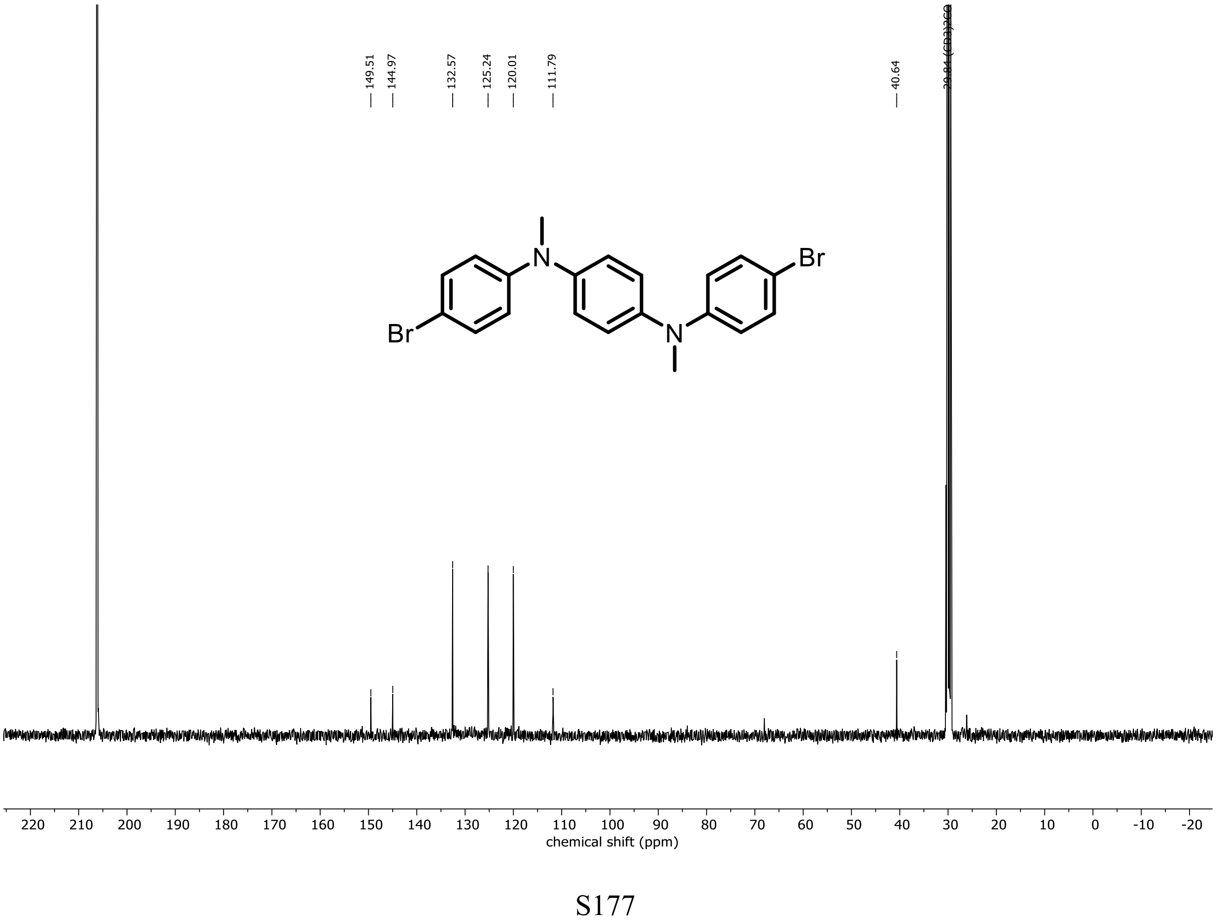

***N*[1]-(4-bromophenyl)-*N*[1],*N*[4]-dimethyl-*N*[4]-(4-(methyl(4-(methyl(phenyl)amino)phenyl)amino)phenyl) benzene-1,4-diamine (S17d)** (^{1}H NMR, 600 MHz, C_6D_6)

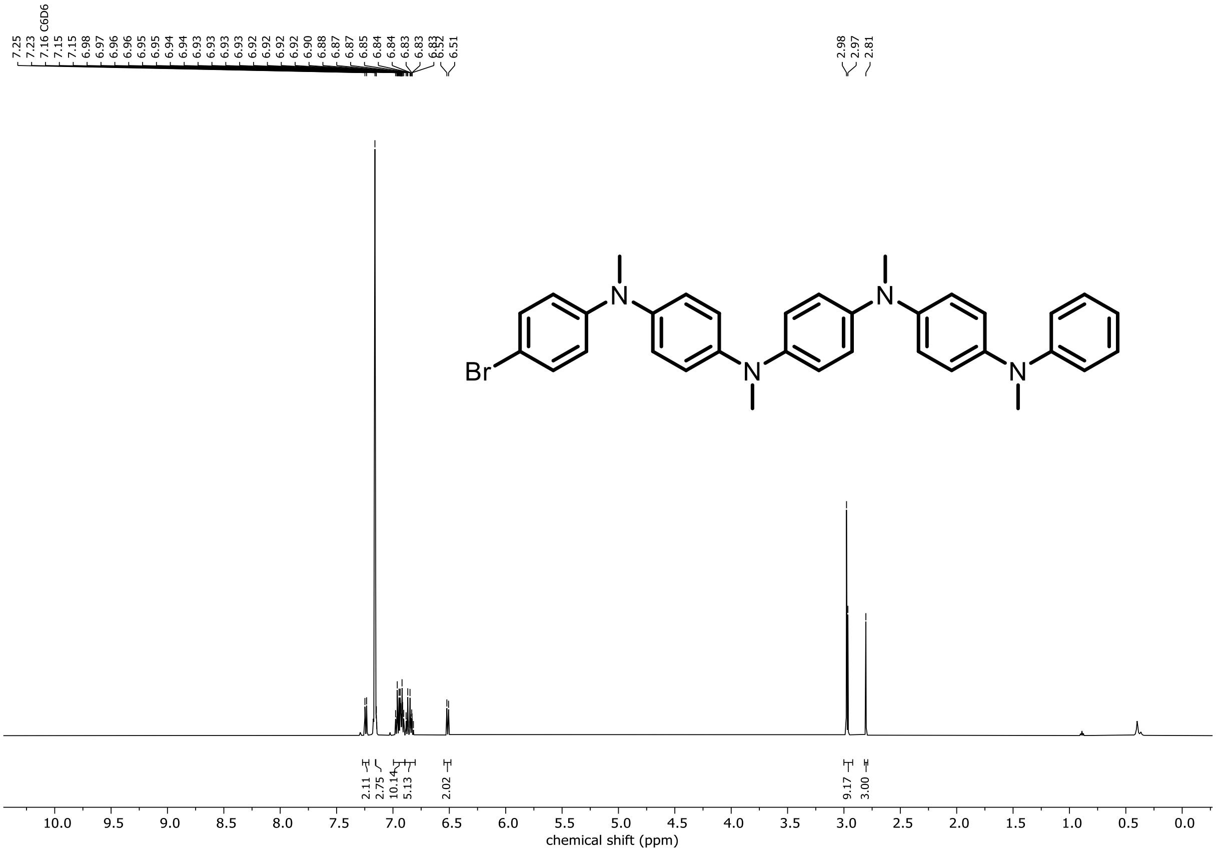

***N*[1]-(4-bromophenyl)-*N*[1],*N*[4]-dimethyl-*N*[4]-(4-(methyl(4-(methyl(phenyl)amino)phenyl)amino)phenyl) benzene-1,4-diamine (S17d)** (^{13}C NMR, 151 MHz, C_6D_6)

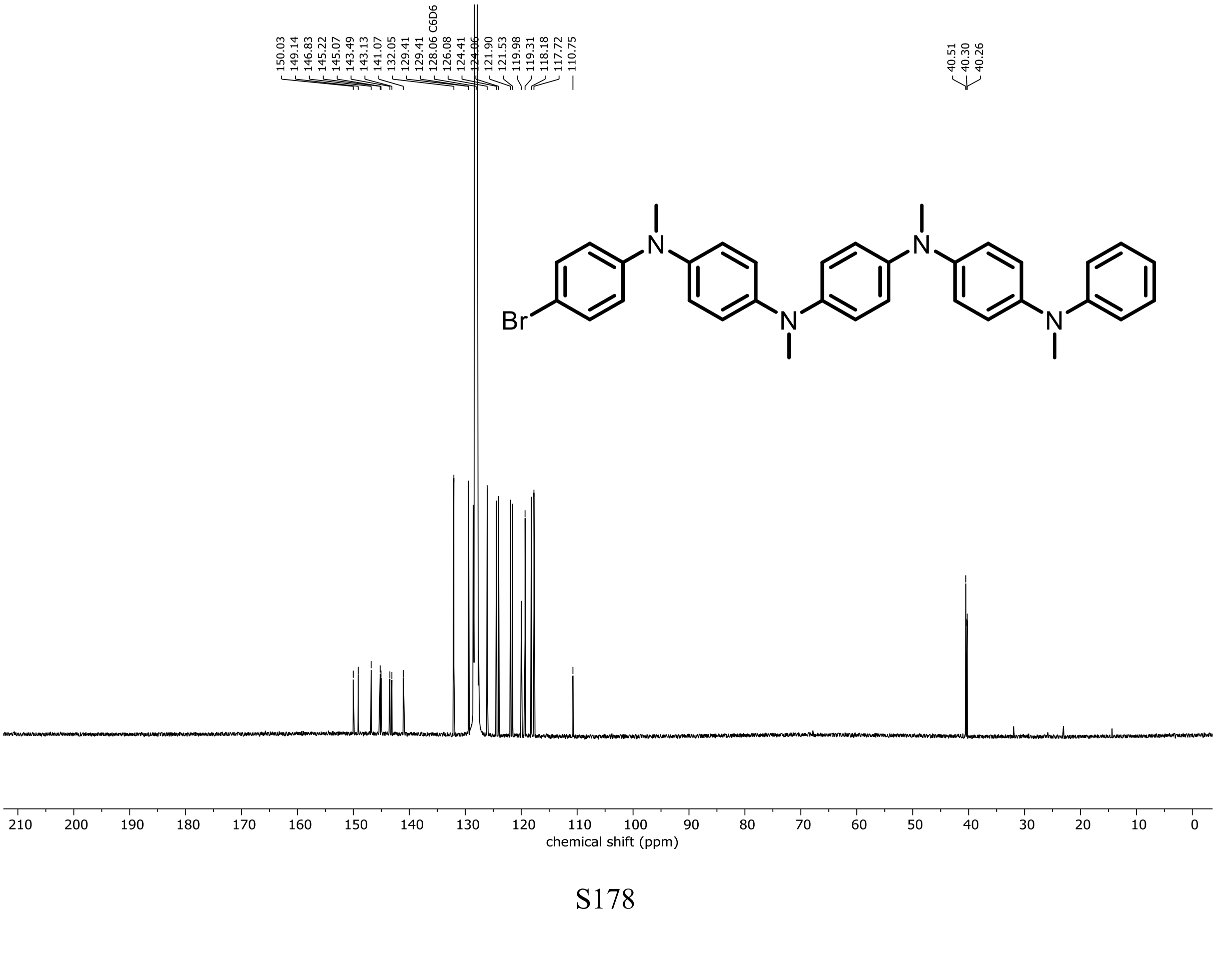

N^1,N^4-dimethyl-N^1-(4-(methyl(4-(4,4,5,5-tetramethyl-1,3,2-dioxaborolan-2-yl)phenyl)amino)phenyl)-N^4-(4-(methyl(phenyl)amino)phenyl)benzene-1,4-diamine (17) (^{1}H NMR, 400 MHz, C_6D_6)

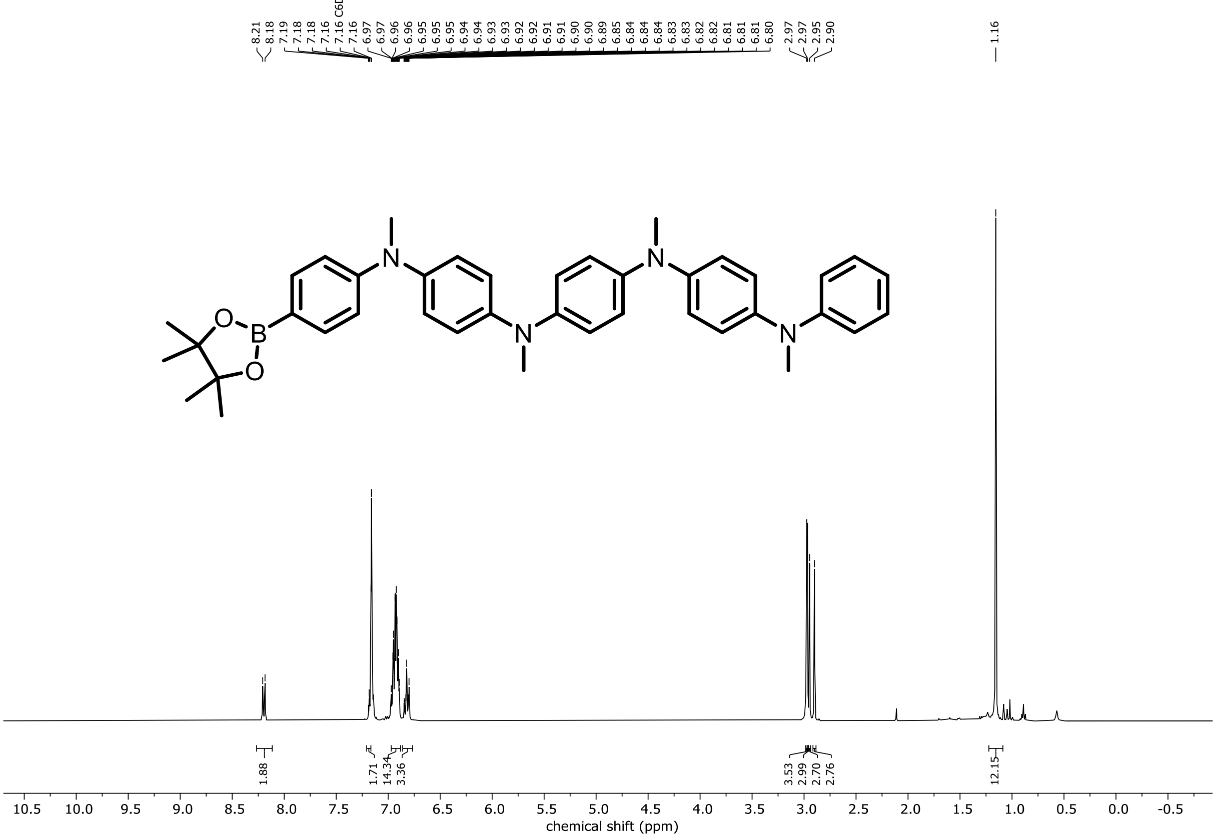

N^1,N^4-dimethyl-N^1-(4-(methyl(4-(4,4,5,5-tetramethyl-1,3,2-dioxaborolan-2-yl)phenyl)amino)phenyl)-N^4-(4-(methyl(phenyl)amino)phenyl)benzene-1,4-diamine (17) (^{13}C NMR, 101 MHz, MHz, C_6D_6)

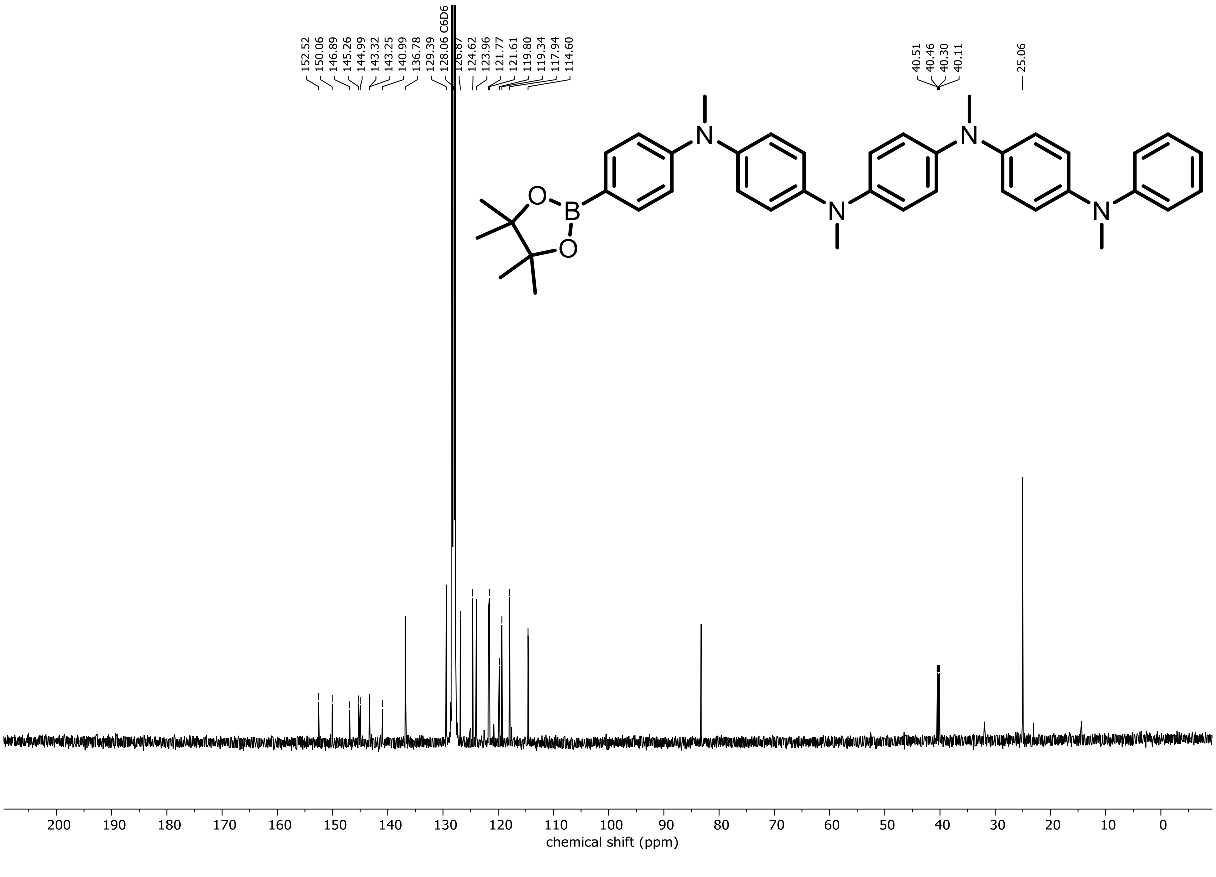

Tri-substituted 1 (tri-1) (^{1}H NMR, 400 MHz, $CDCl_3$)

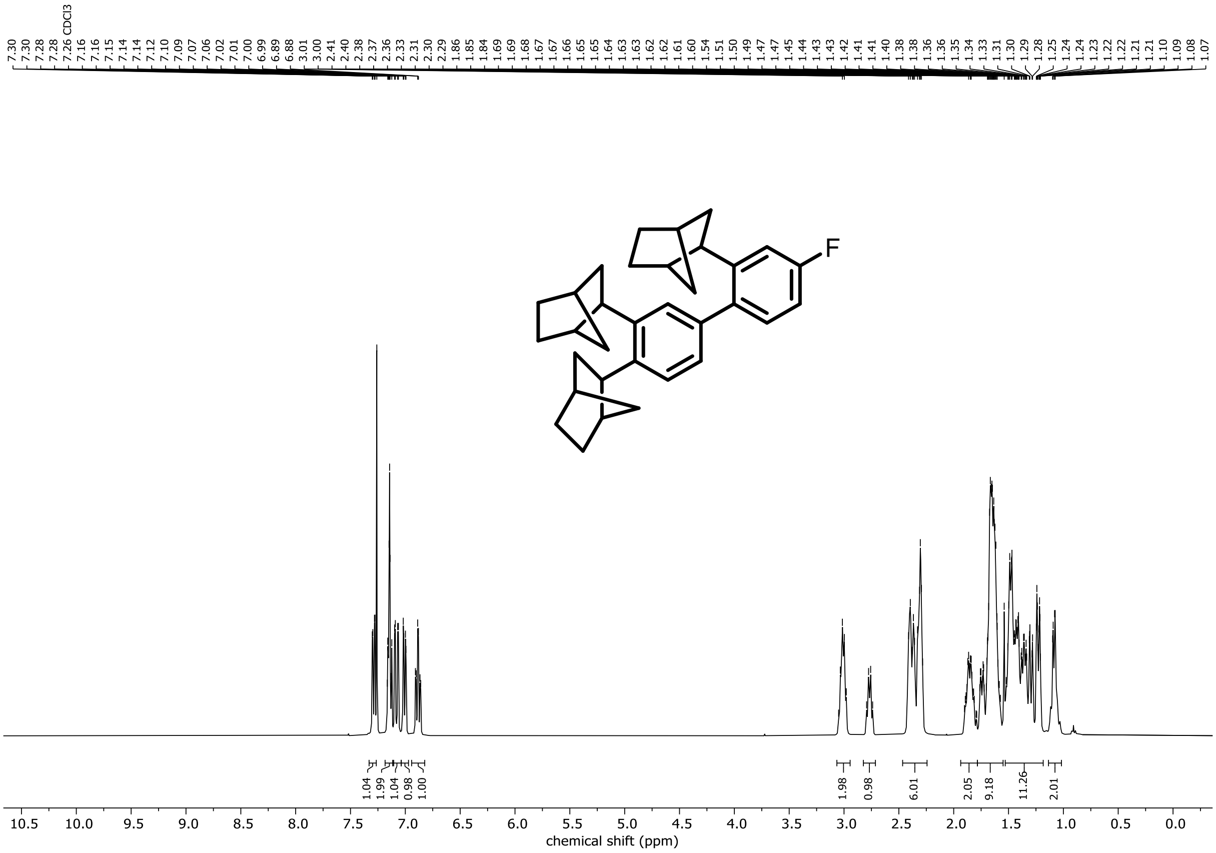

Tri-substituted 1 (tri-1) (^{13}C NMR, 101 MHz, $CDCl_3$)

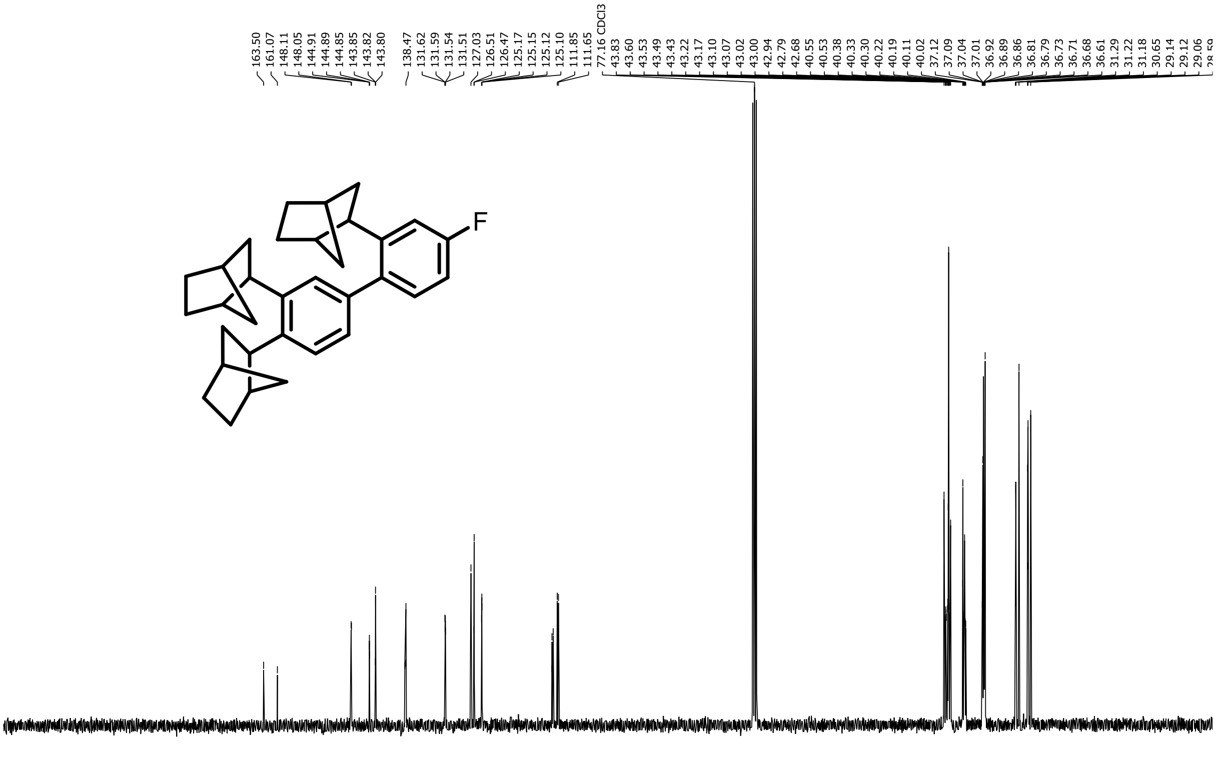

Tri-substituted 1 (tri-1) (^{19}F {^{1}H} NMR, 376 MHz, $CDCl_3$)

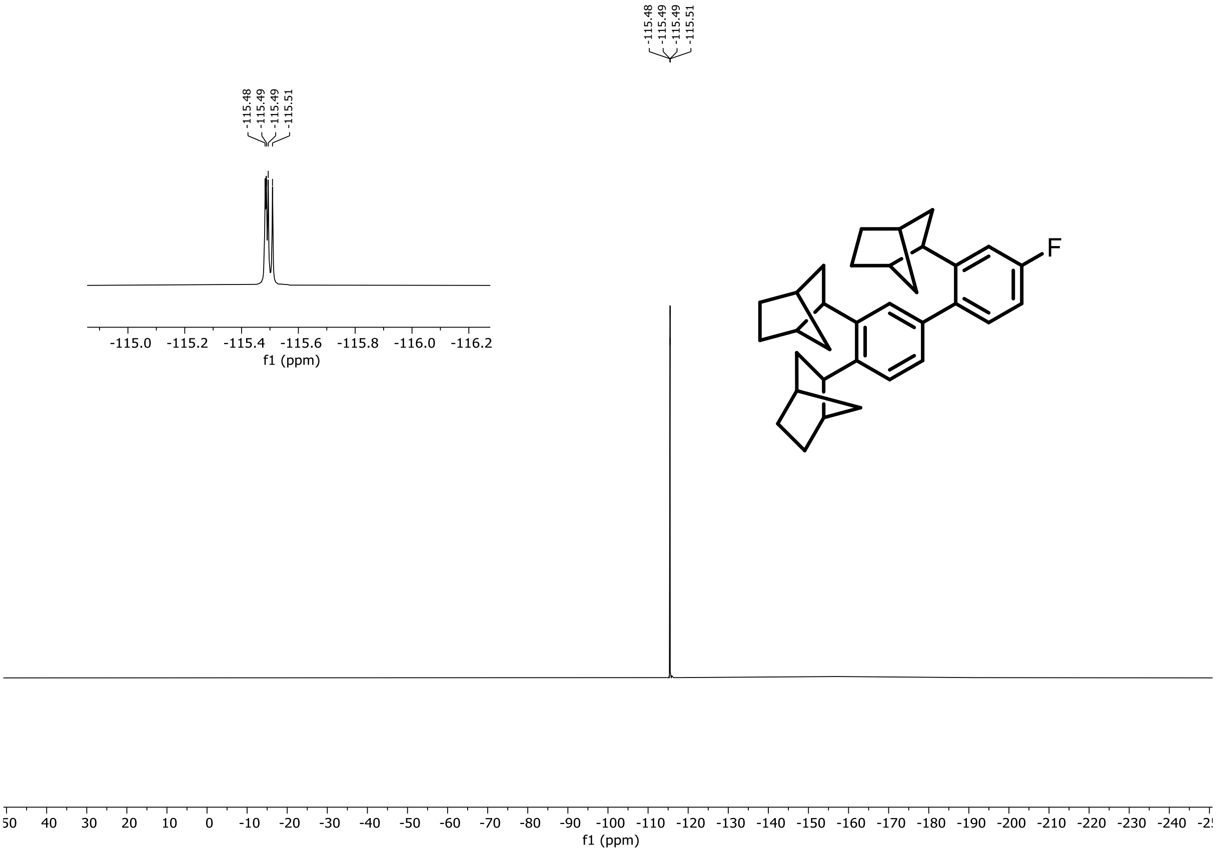

Di-substituted 1 (di-1) (^{1}H NMR, 500 MHz, CDCl$_3$)

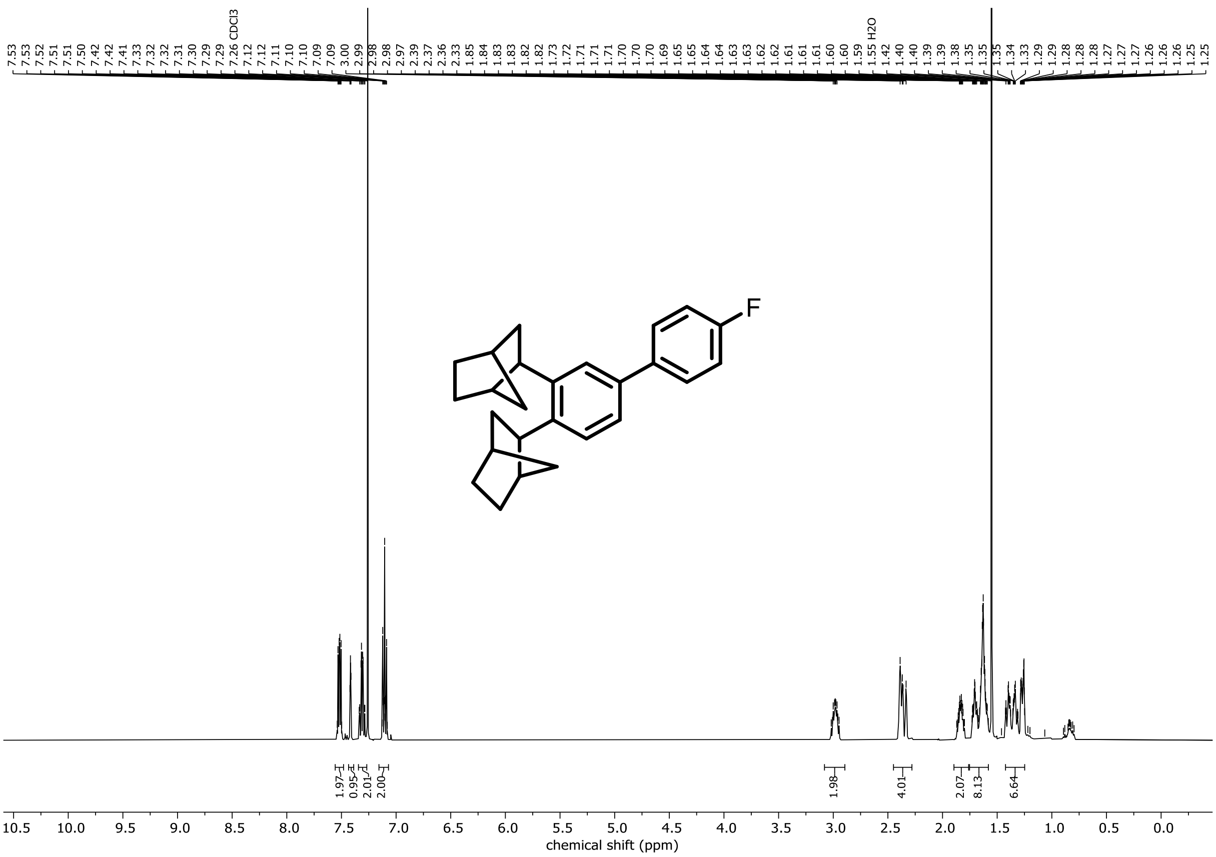

Di-substituted 1 (di-1) (^{13}C NMR, 126 MHz, CDCl$_3$)

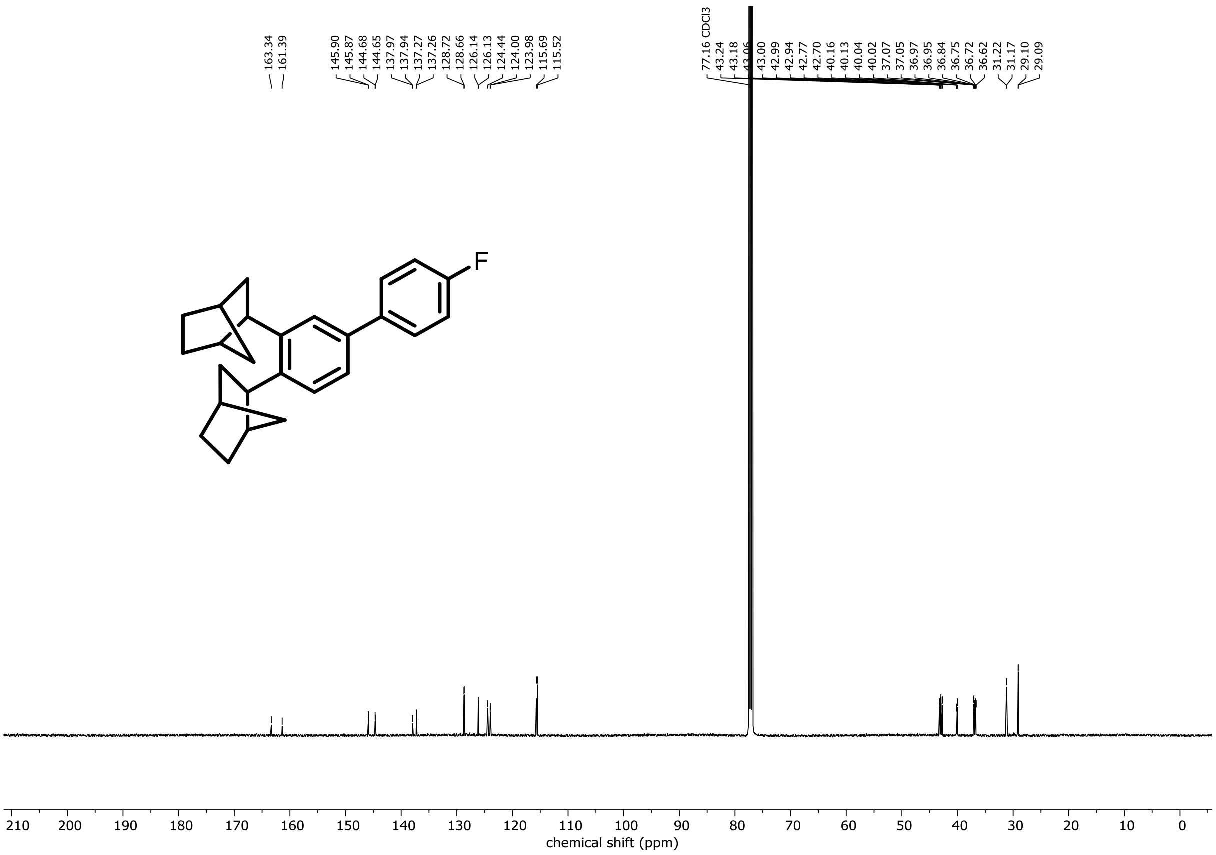

Di-substituted 1 (di-1) (^{19}F {^{1}H} NMR, 376 MHz, $CDCl_3$)

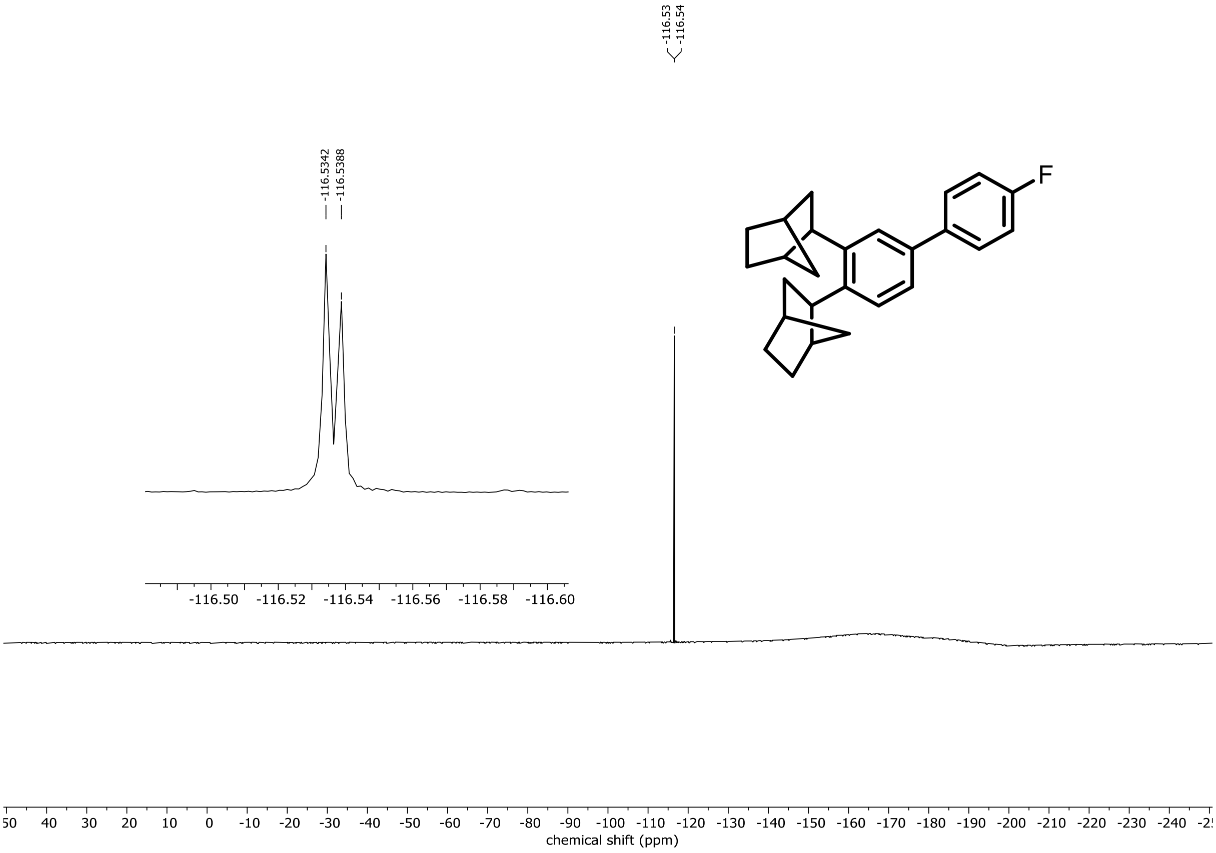

Tri-substituted 2 (tri-2) (^{1}H NMR, 400 MHz, $CDCl_3$)

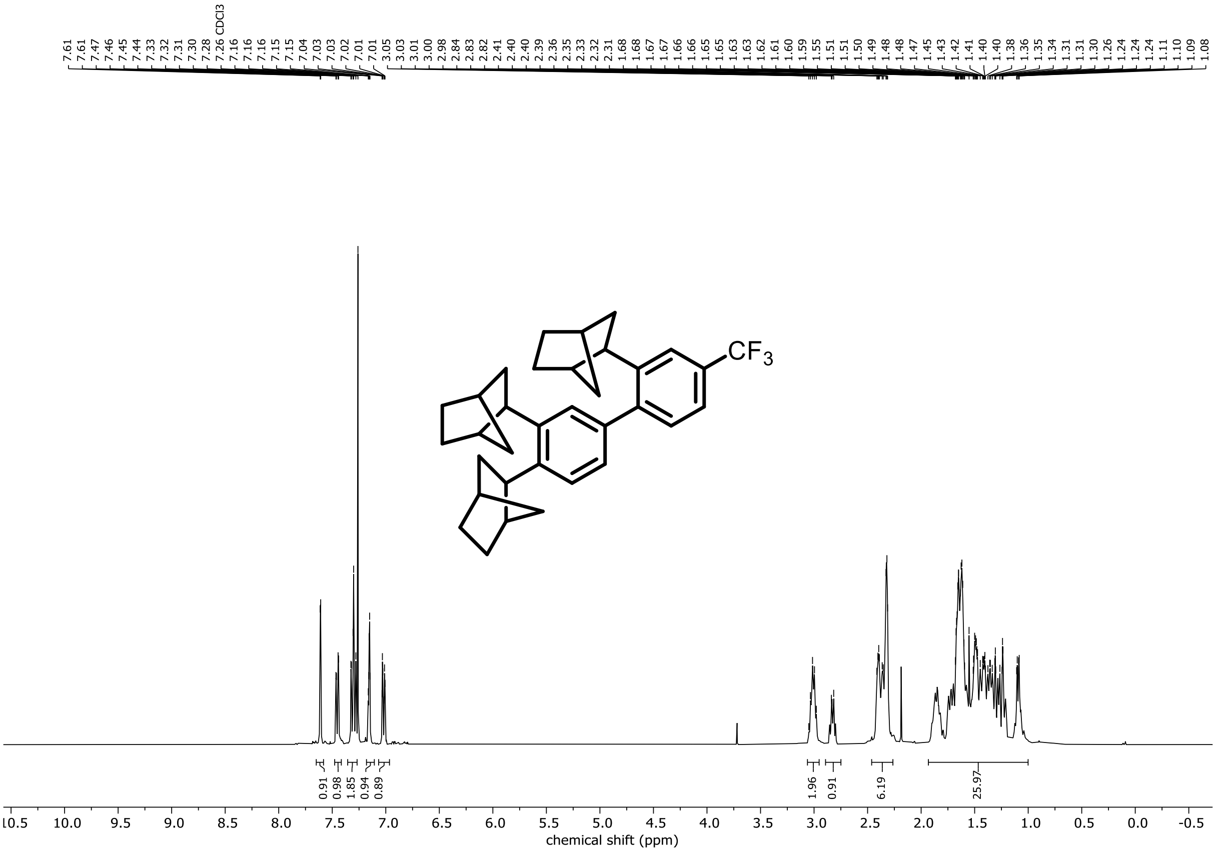

Tri-substituted 2 (tri-2) (^{13}C NMR, 101 MHz, $CDCl_3$)

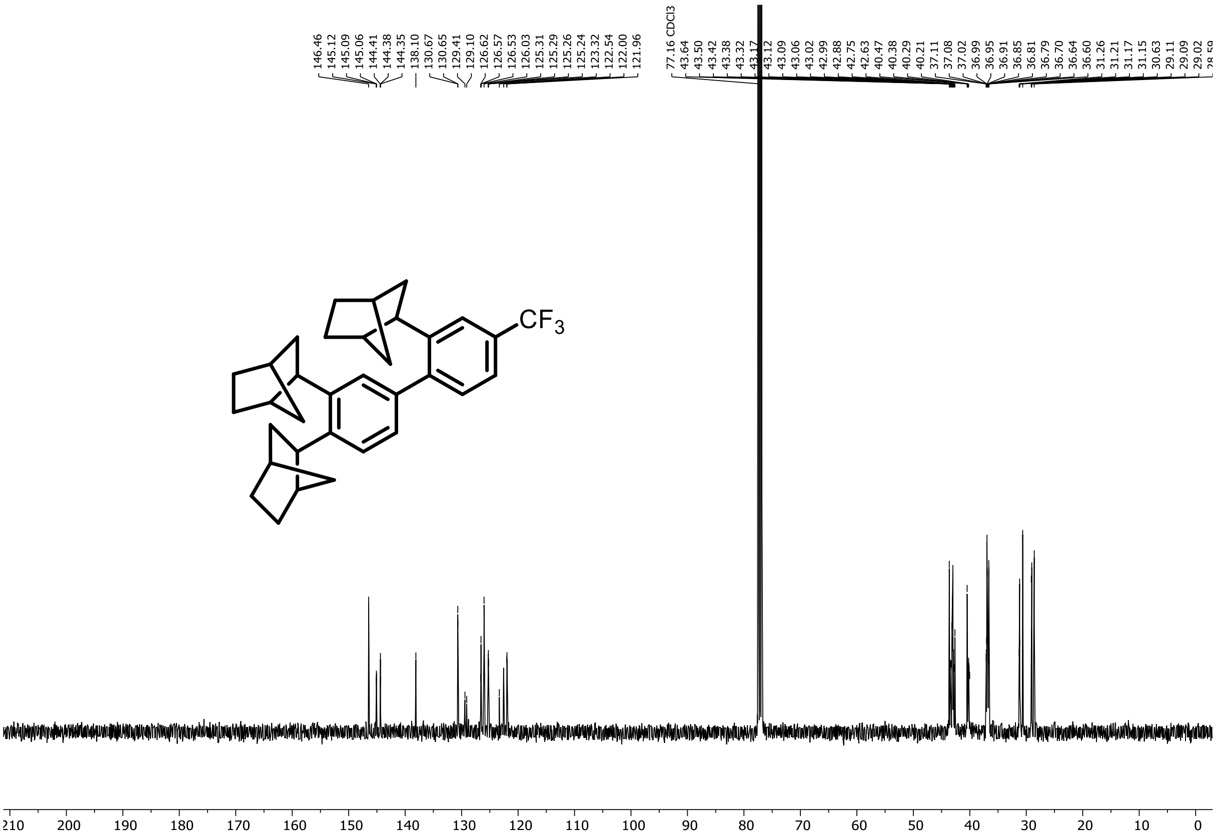

Tri-substituted 2 (tri-2) ($^{19}F\{^{1}H\}$ NMR, 376 MHz, $CDCl_3$)

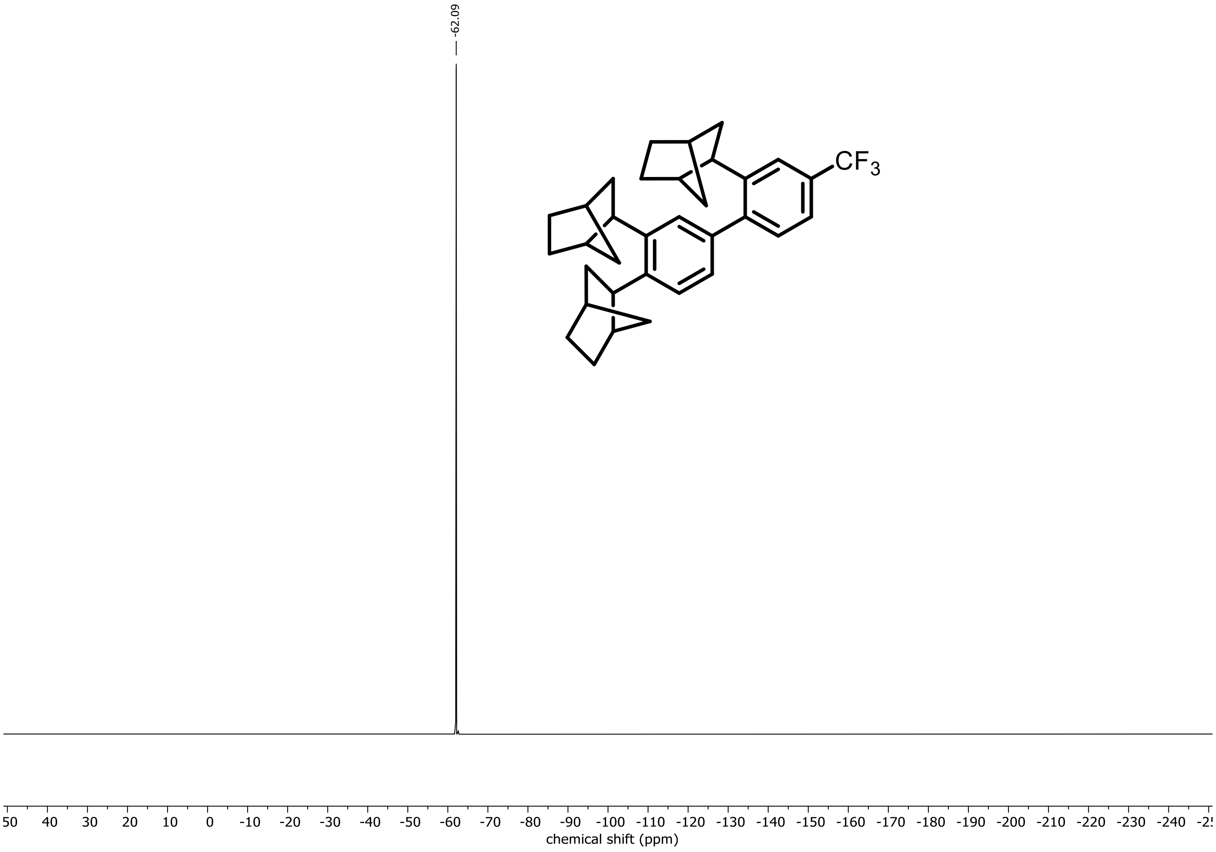

Di-substituted 2 (di-2) (^{1}H NMR, 500 MHz, $CDCl_3$)

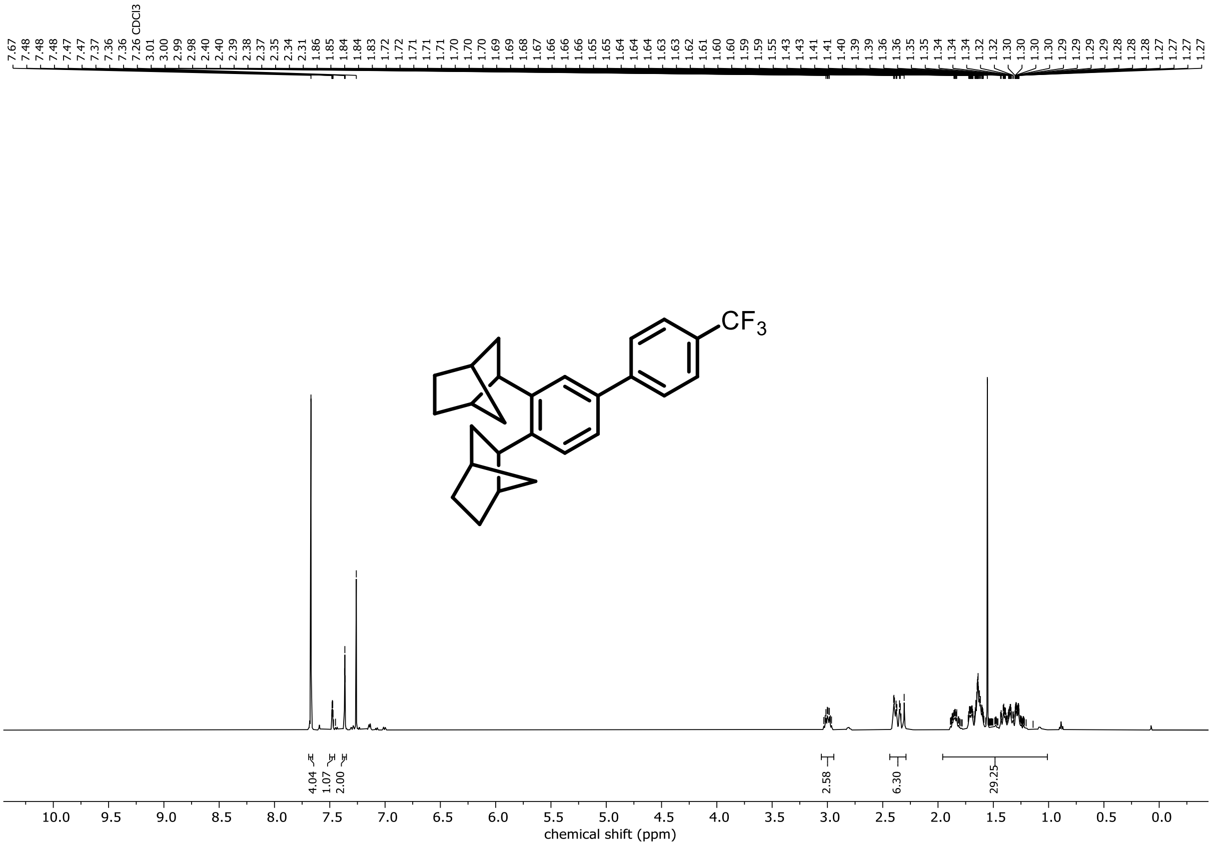

Di-substituted 2 (di-2) (^{13}C NMR, 126 MHz, $CDCl_3$)

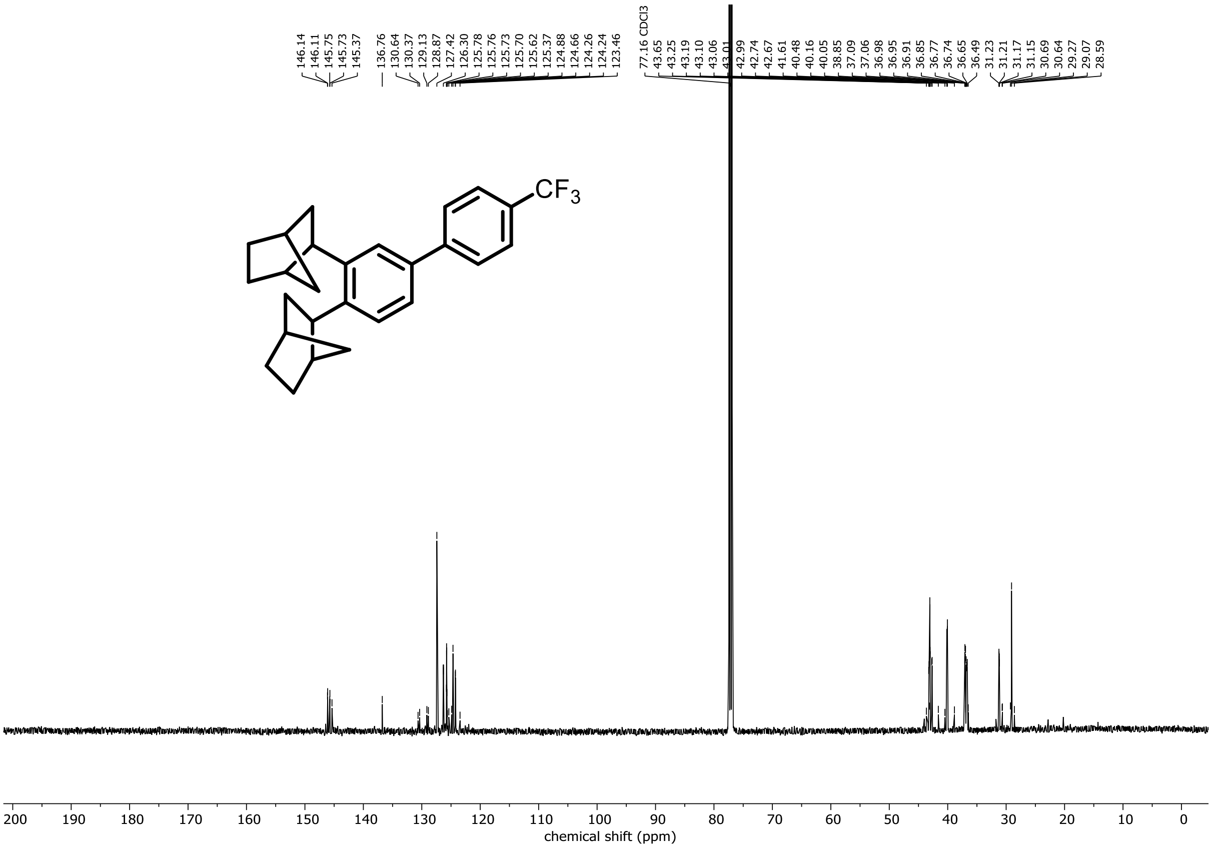

Di-substituted 2 (di-2) (^{19}F NMR, 377 MHz, $CDCl_3$)

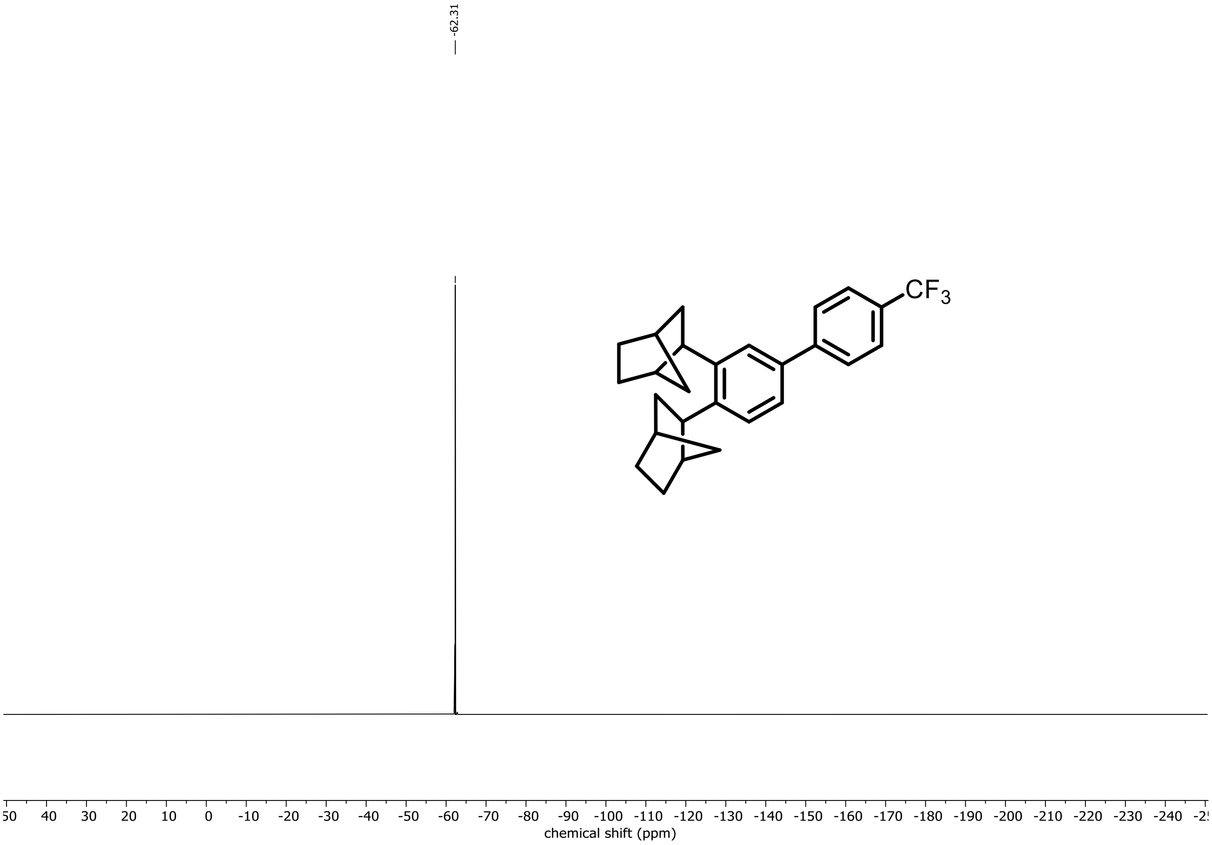

Tri-substituted 3 (tri-3) (^{1}H NMR, 400 MHz, $CDCl_3$)

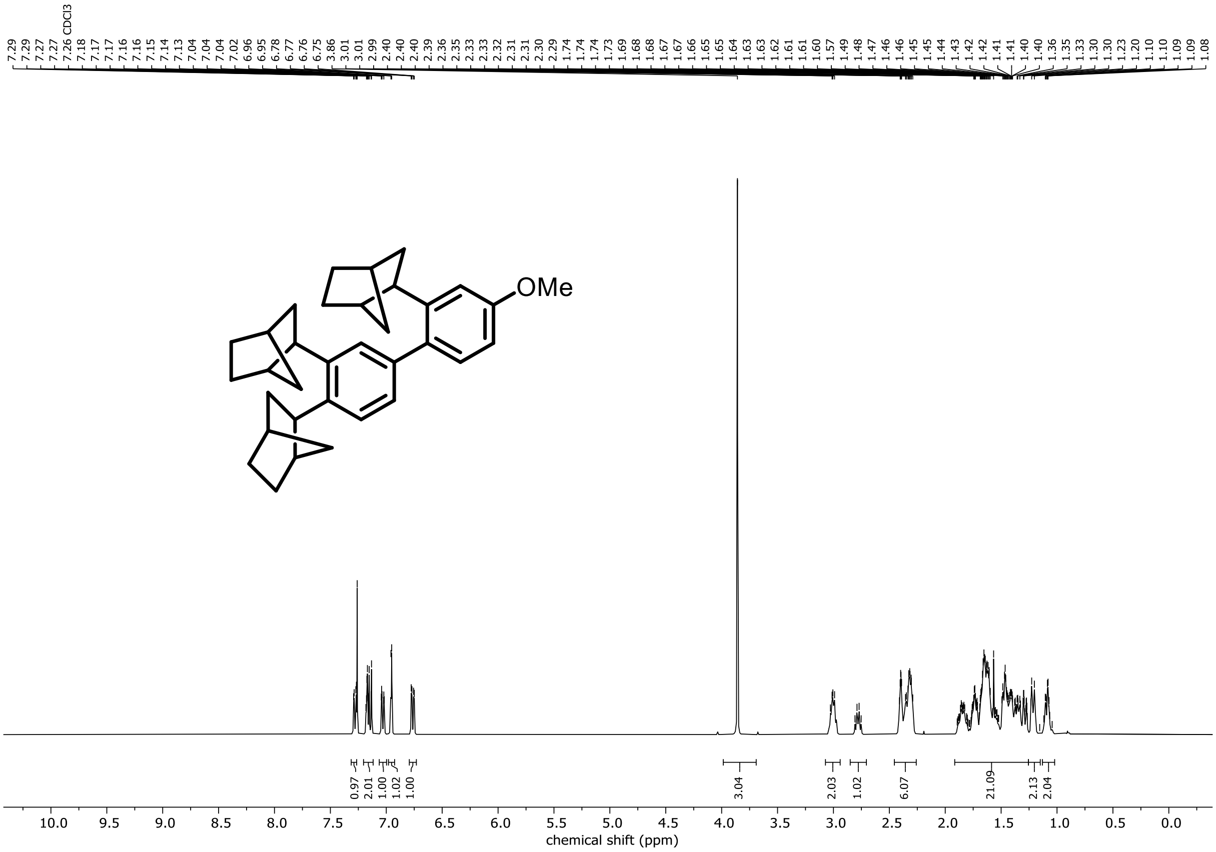

Tri-substituted 3 (tri-3) (^{13}C NMR, 101 MHz, $CDCl_3$)

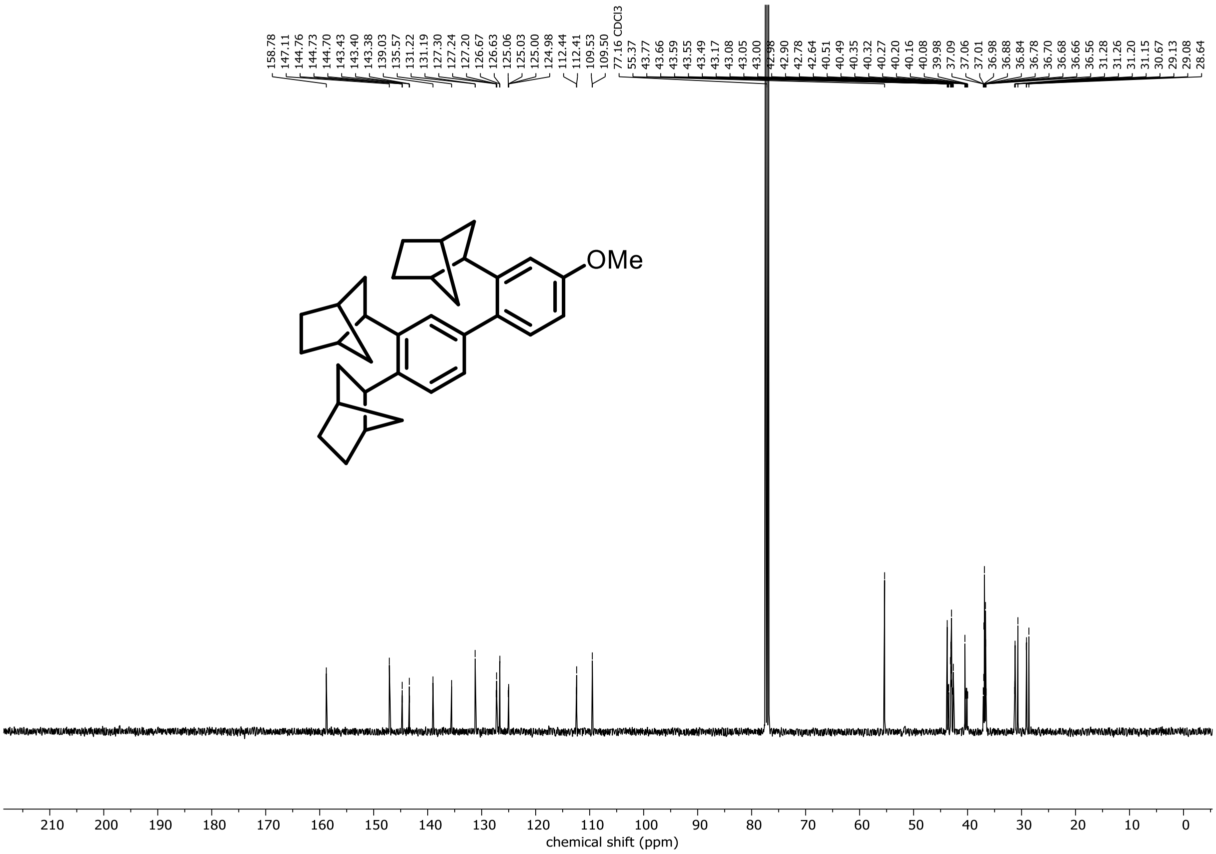

Tetra-substituted 4 (tetra-4) (^{1}H NMR, 600 MHz, $CDCl_3$)

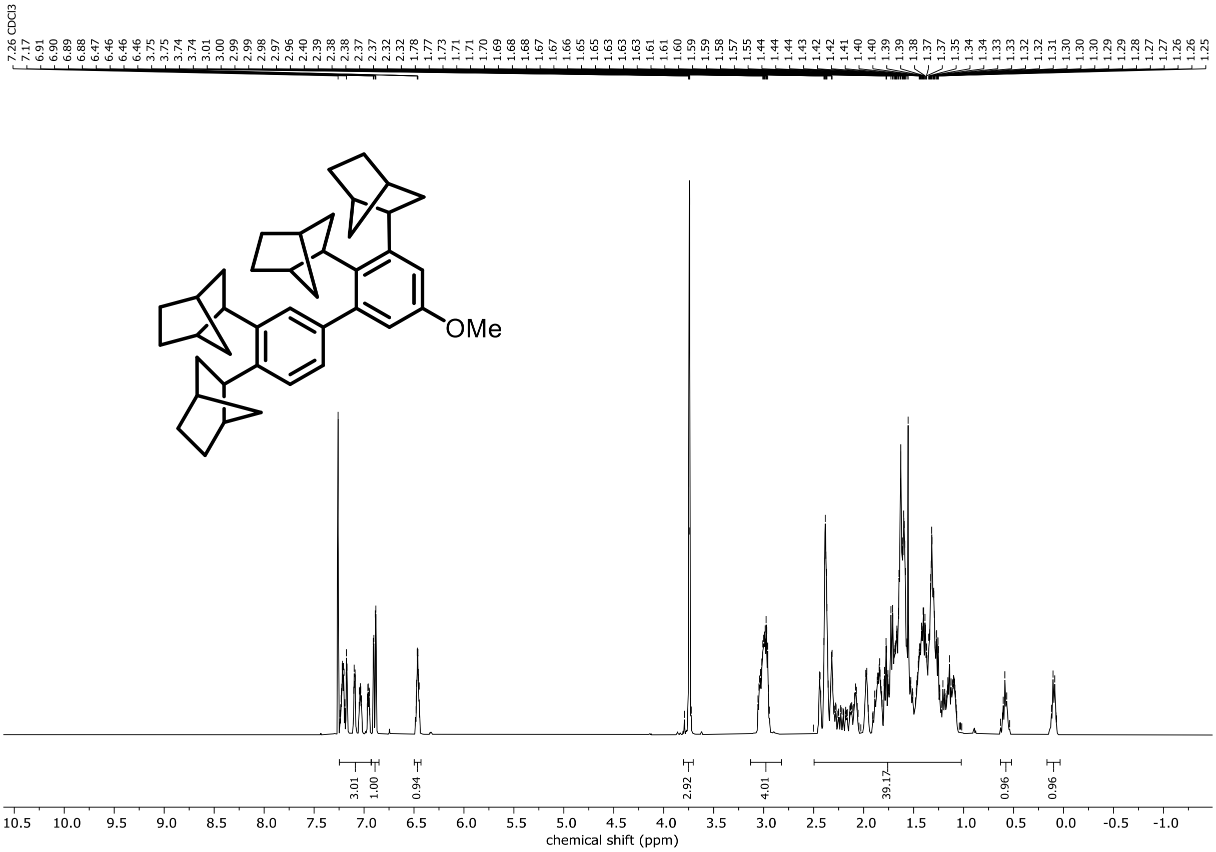

Tetra-substituted 4 (tetra-4) (^{13}C NMR, 151 MHz, $CDCl_3$)

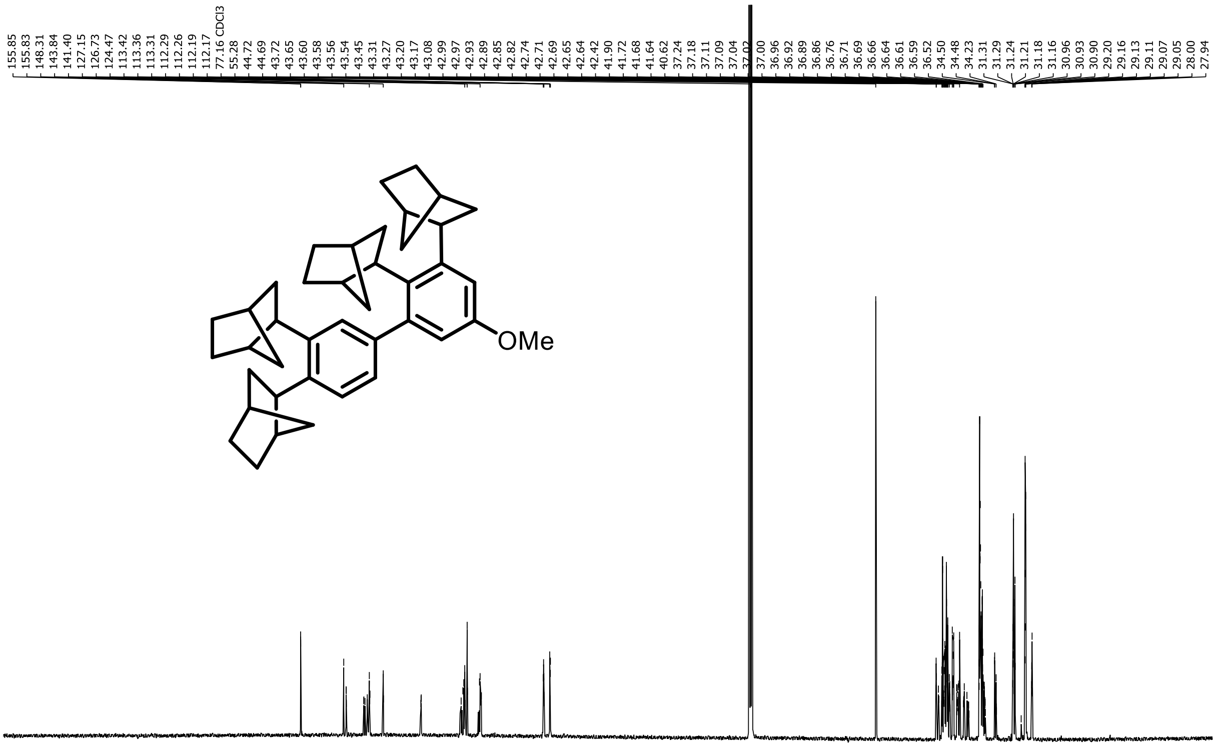

Tri-substituted 5 (tri-5) (^{1}H NMR, 400 MHz, $CDCl_3$)

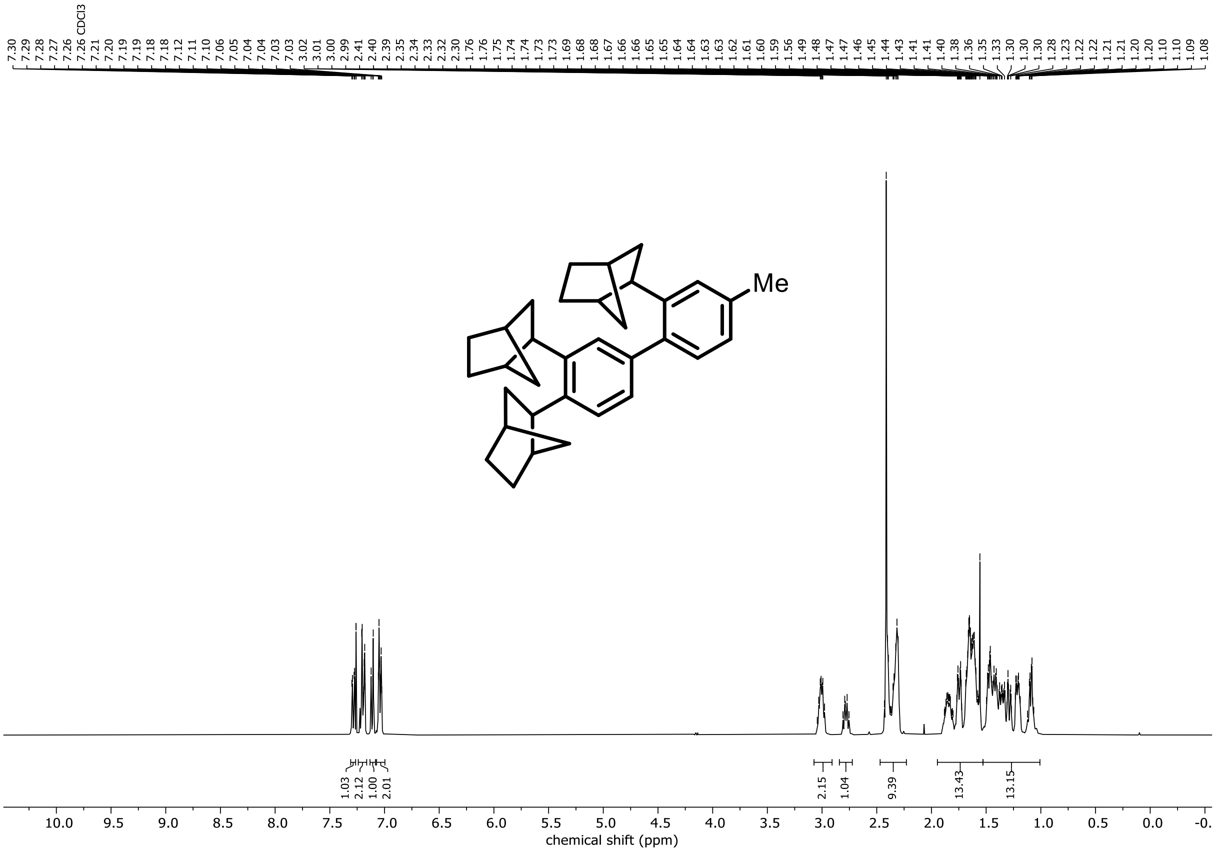

Tri-substituted 5 (tri-5) (^{13}C NMR, 101 MHz, $CDCl_3$)

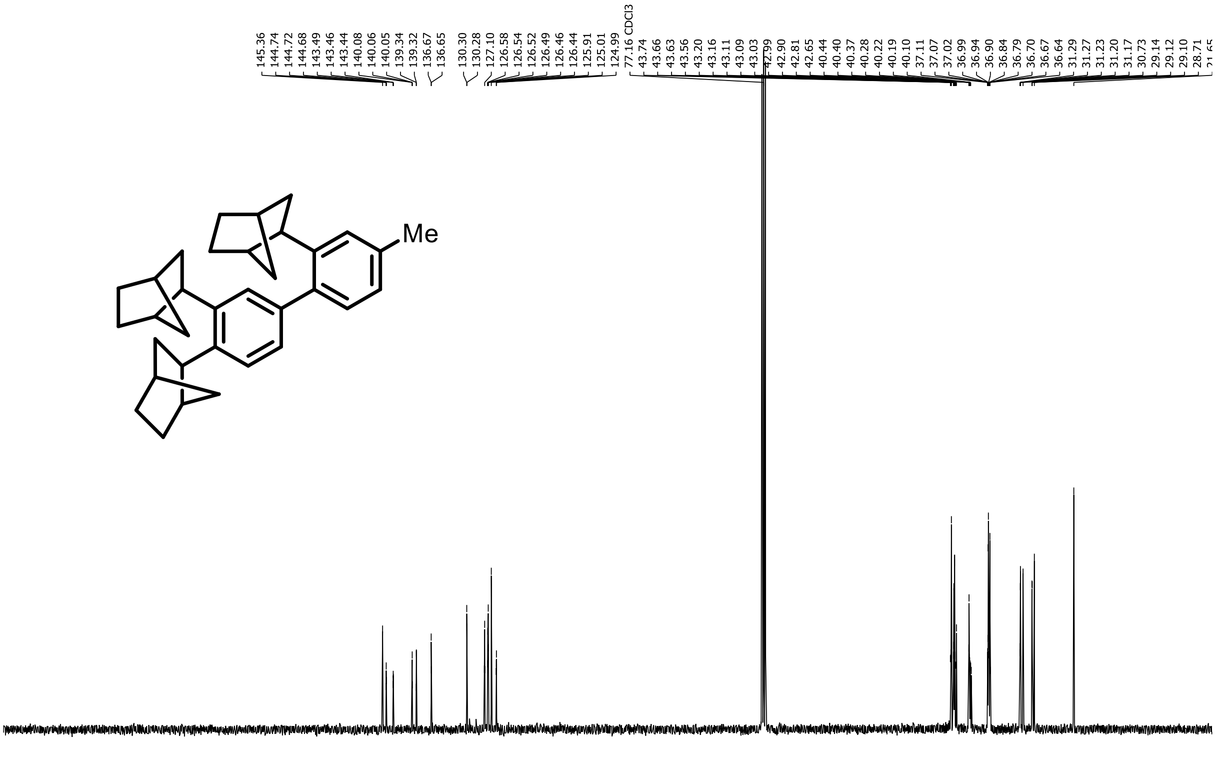

Di-substituted 5 (di-5) (^{1}H NMR, 500 MHz, $CDCl_3$; **di-5** : **tri-5** = 1.37:1.0)

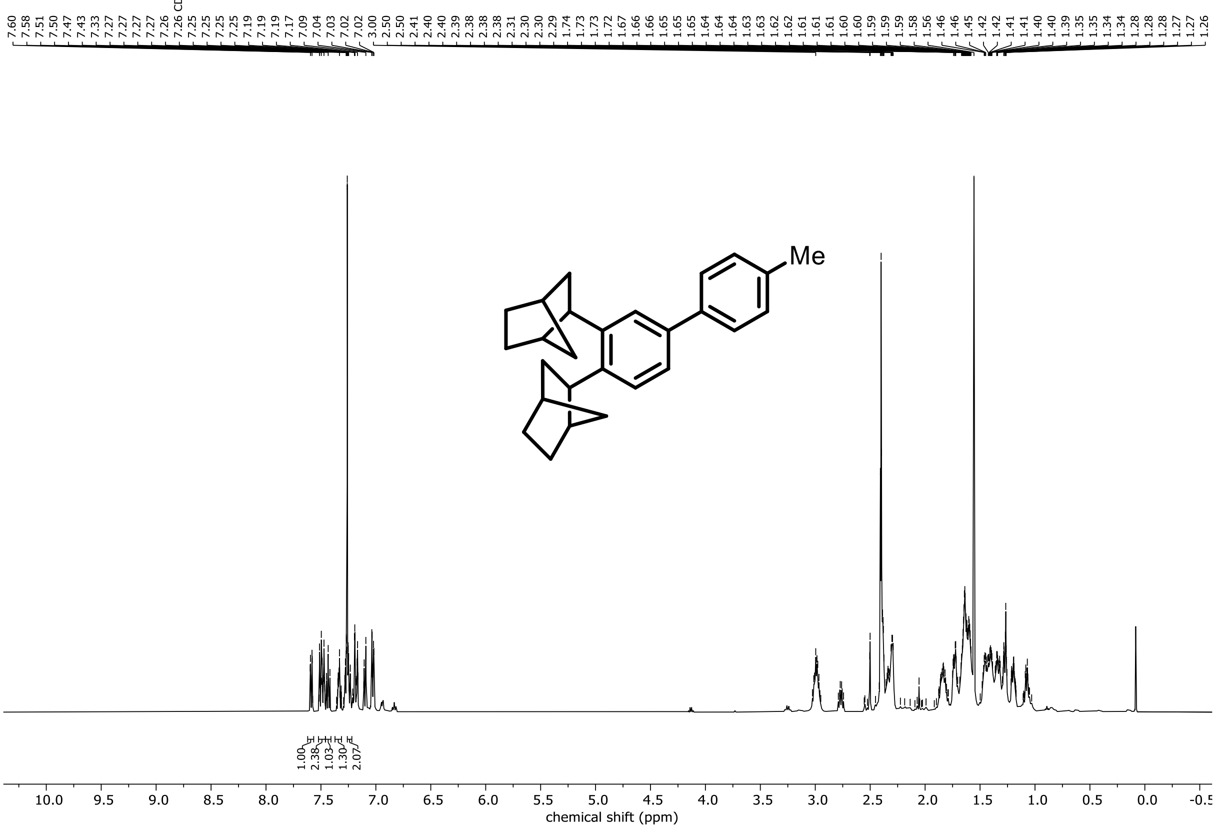

Di-substituted 5 (di-5) (^{13}C NMR, 126 MHz, $CDCl_3$; **di-5** : **tri-5** = 1.37:1.0)

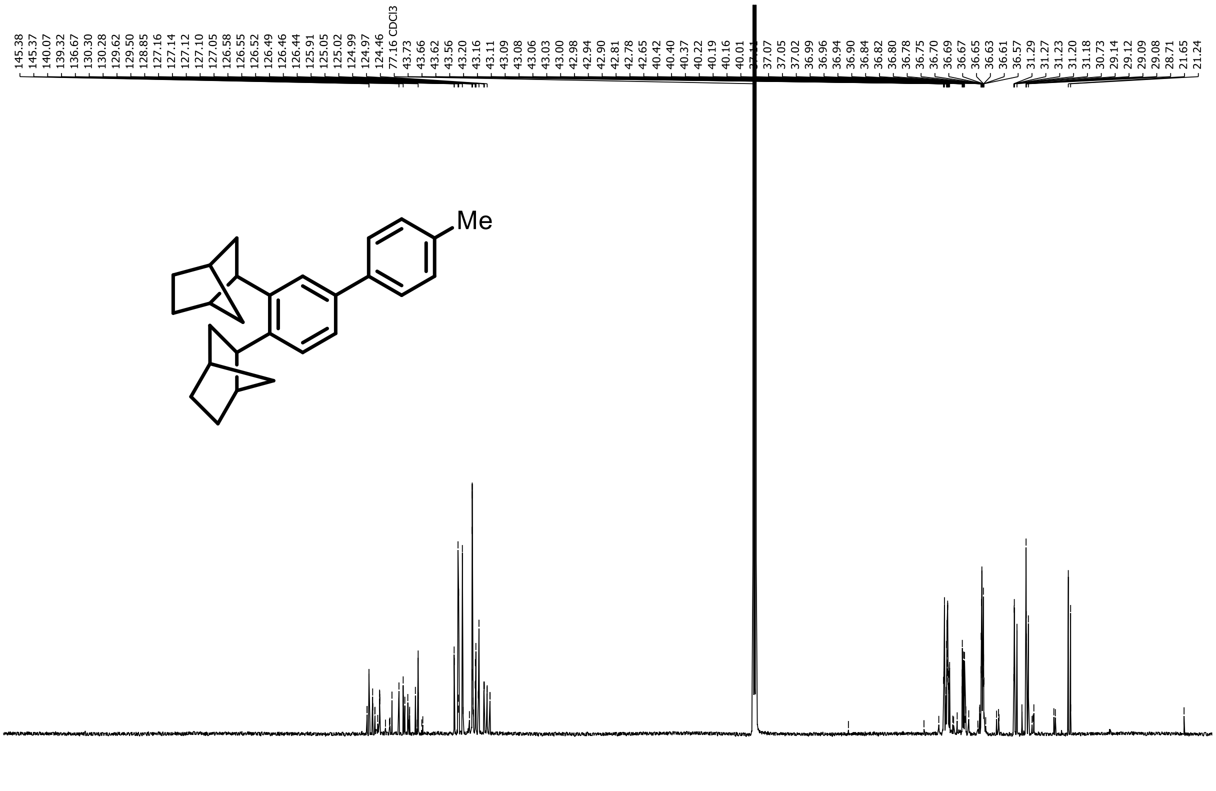

Tri-substituted 6 (tri-6) (^{1}H NMR, 400 MHz, $CDCl_3$)

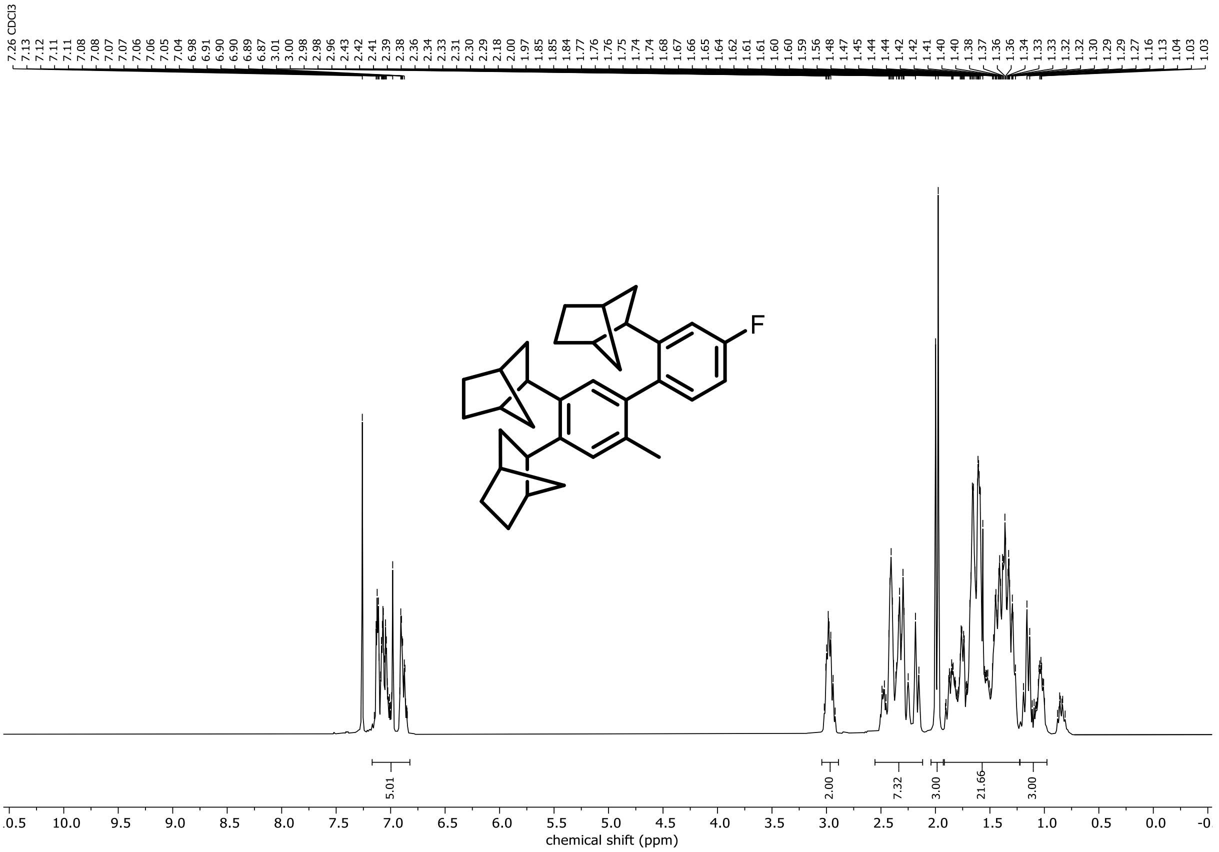

Tri-substituted 6 (tri-6) (^{13}C NMR, 101 MHz, $CDCl_3$)

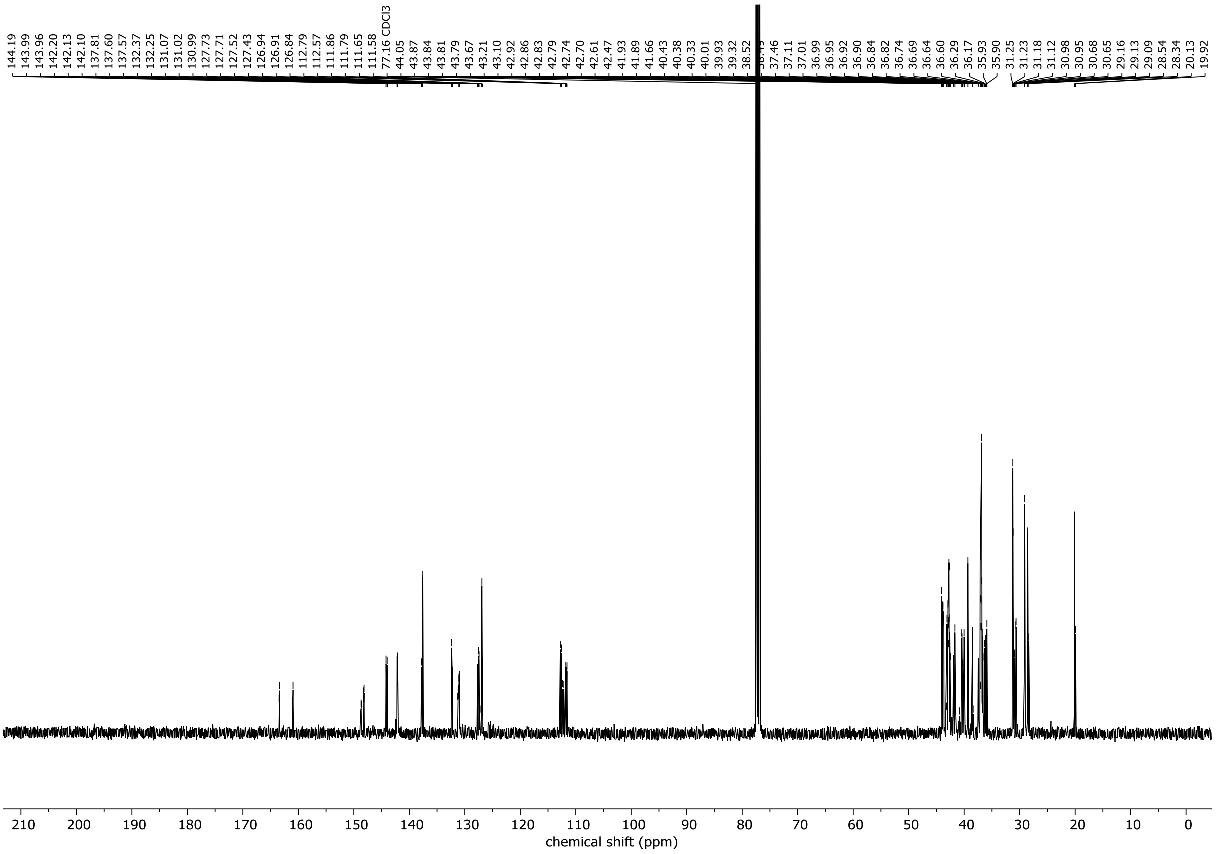

Tri-substituted 6 (tri-6) (^{19}F {^1H} NMR, 376 MHz, $CDCl_3$)

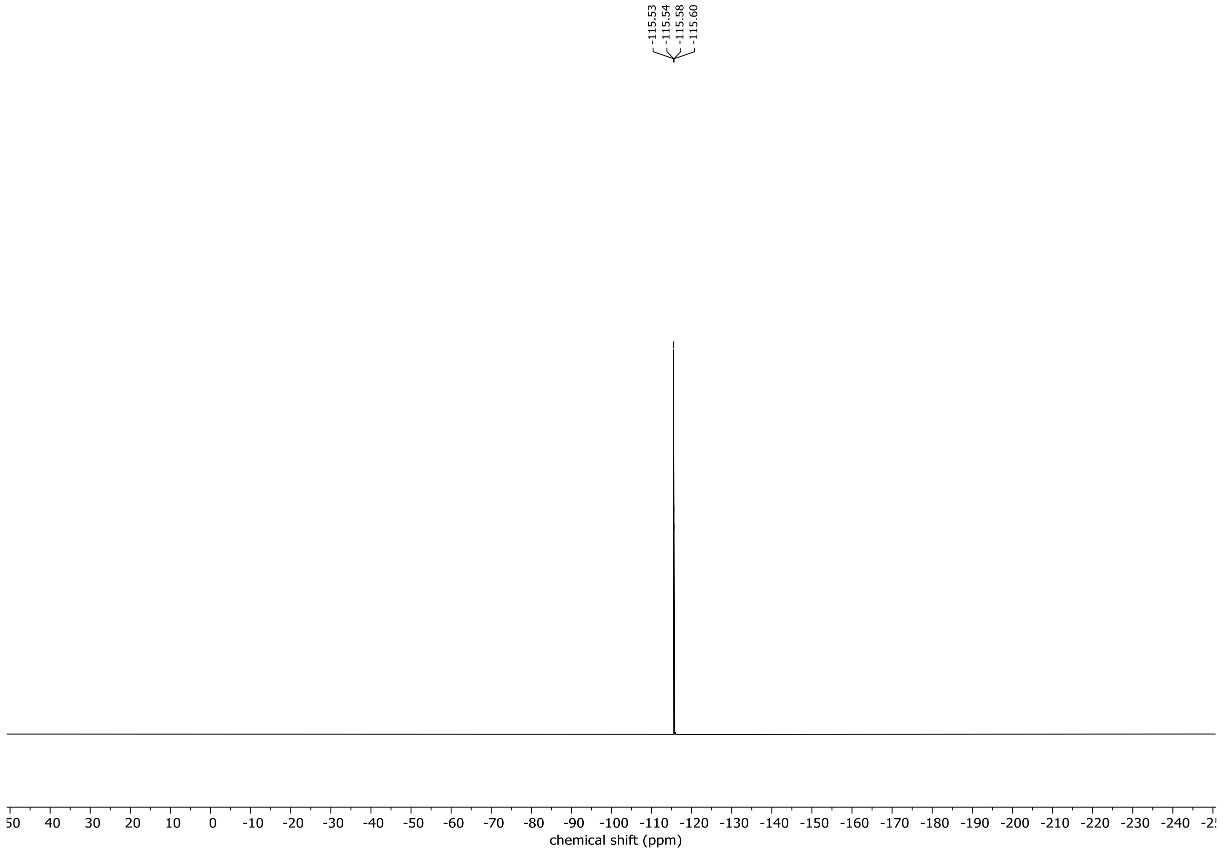

Tri-substituted 7 (tri-7) (^{1}H NMR, 400 MHz, $CDCl_3$)

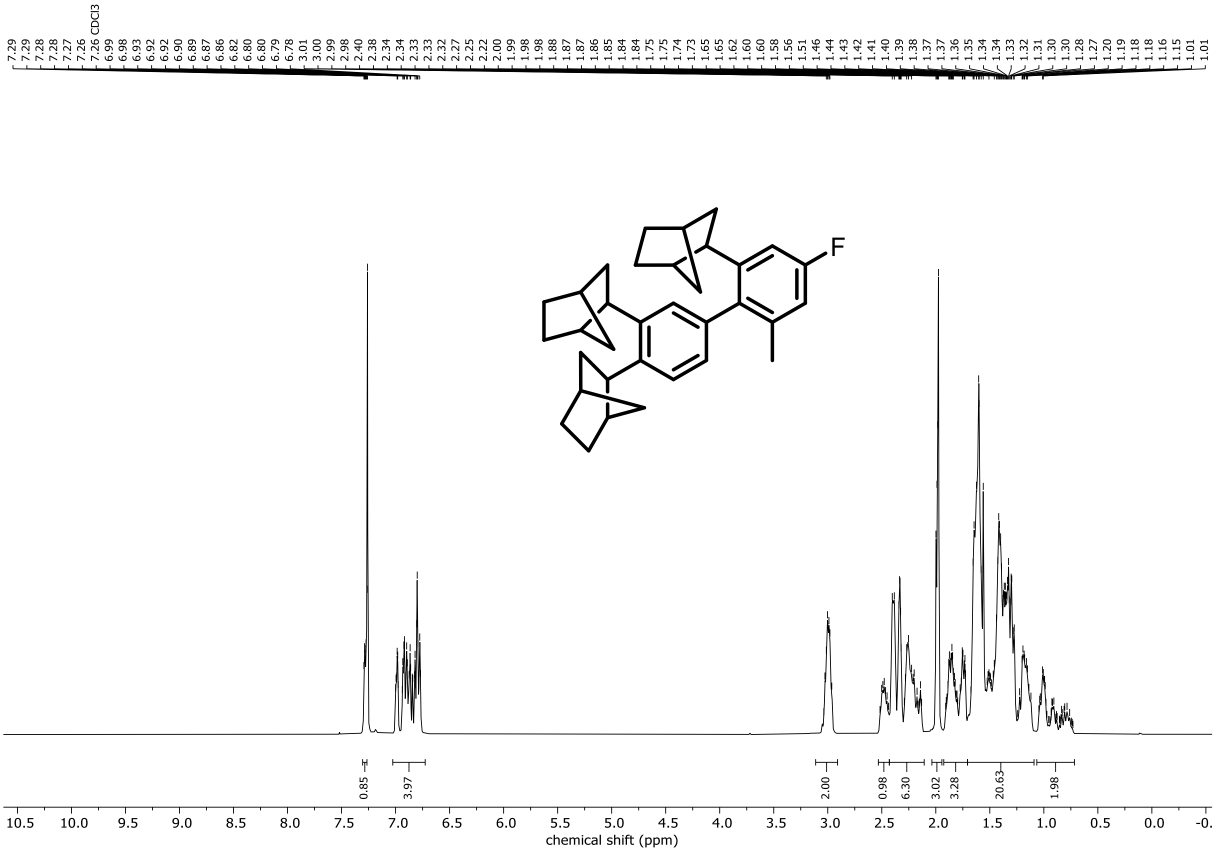

Tri-substituted 7 (tri-7) (^{13}C NMR, 101 MHz, $CDCl_3$)

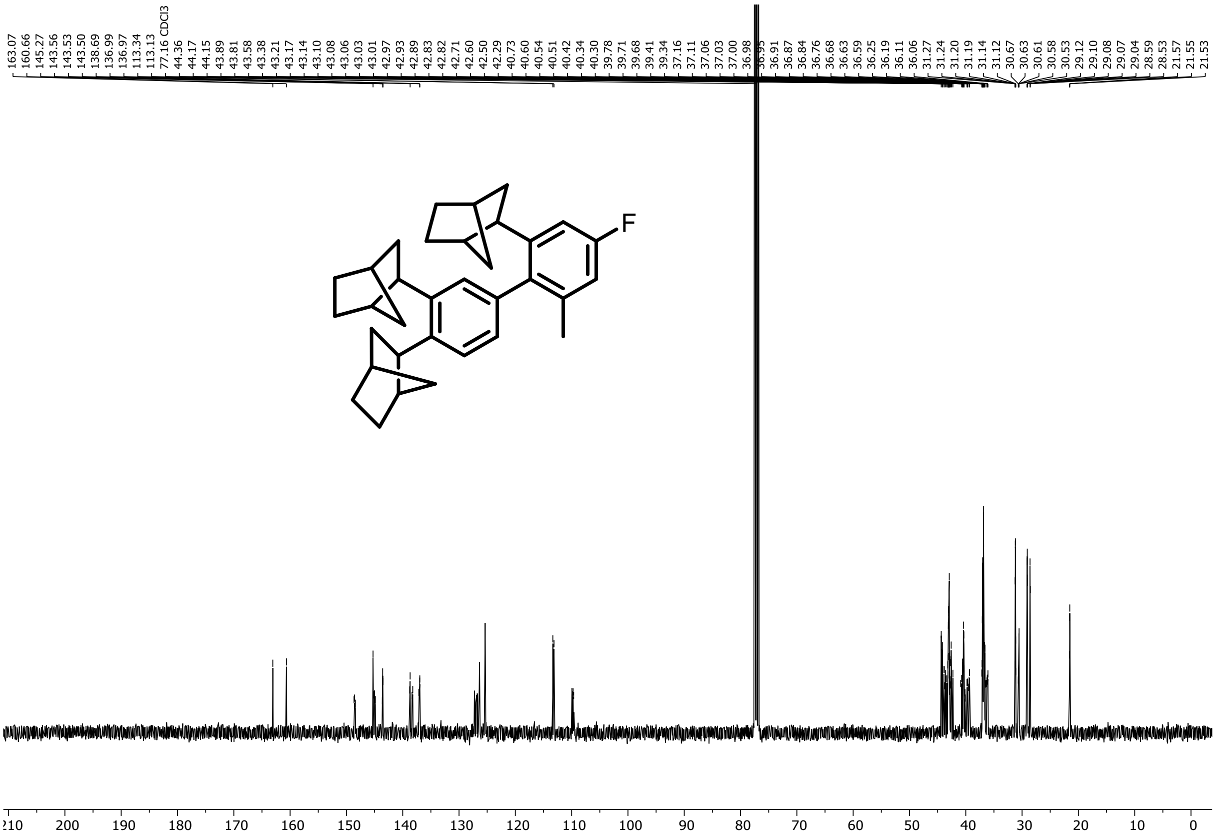

Tri-substituted 7 (tri-7) (^{19}F {^{1}H} NMR, 376 MHz, $CDCl_3$)

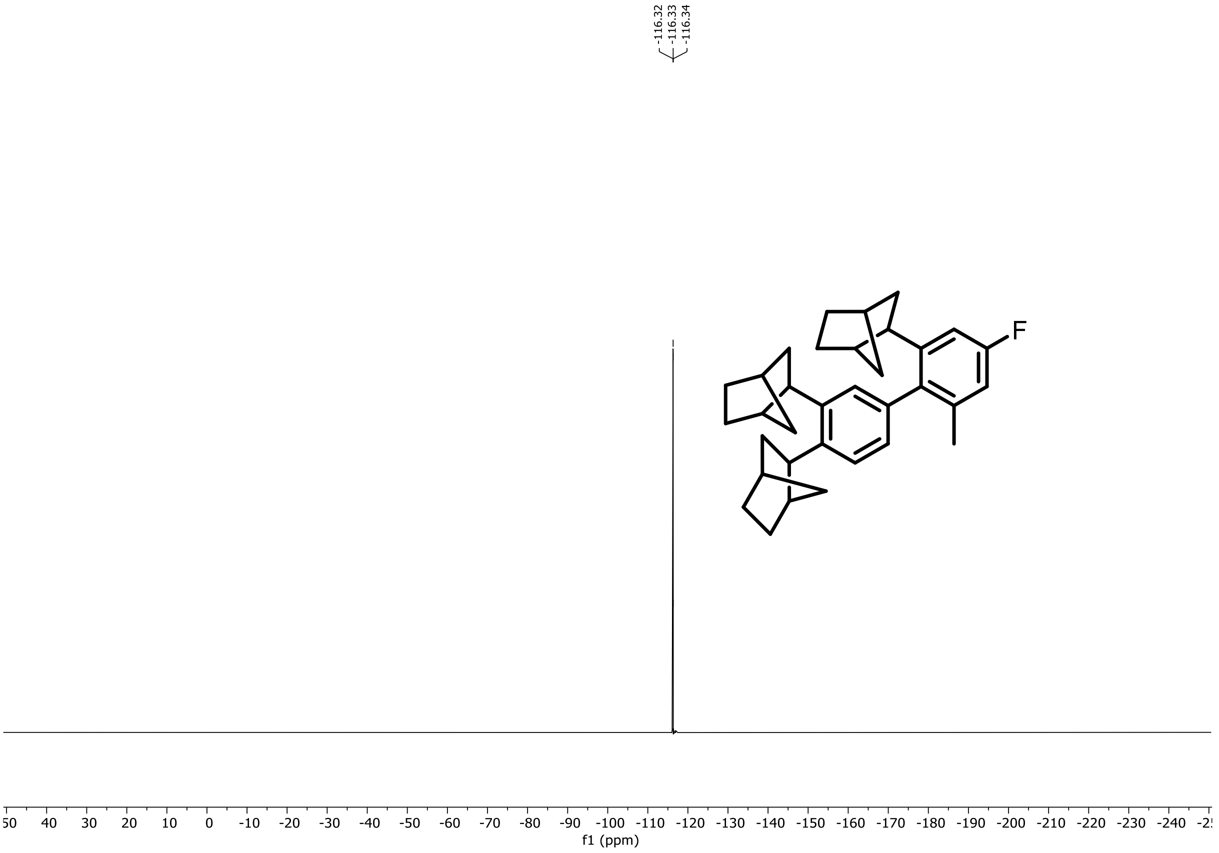

Di-substituted 8 (di-8) (^{1}H NMR, 400 MHz, $CDCl_3$)

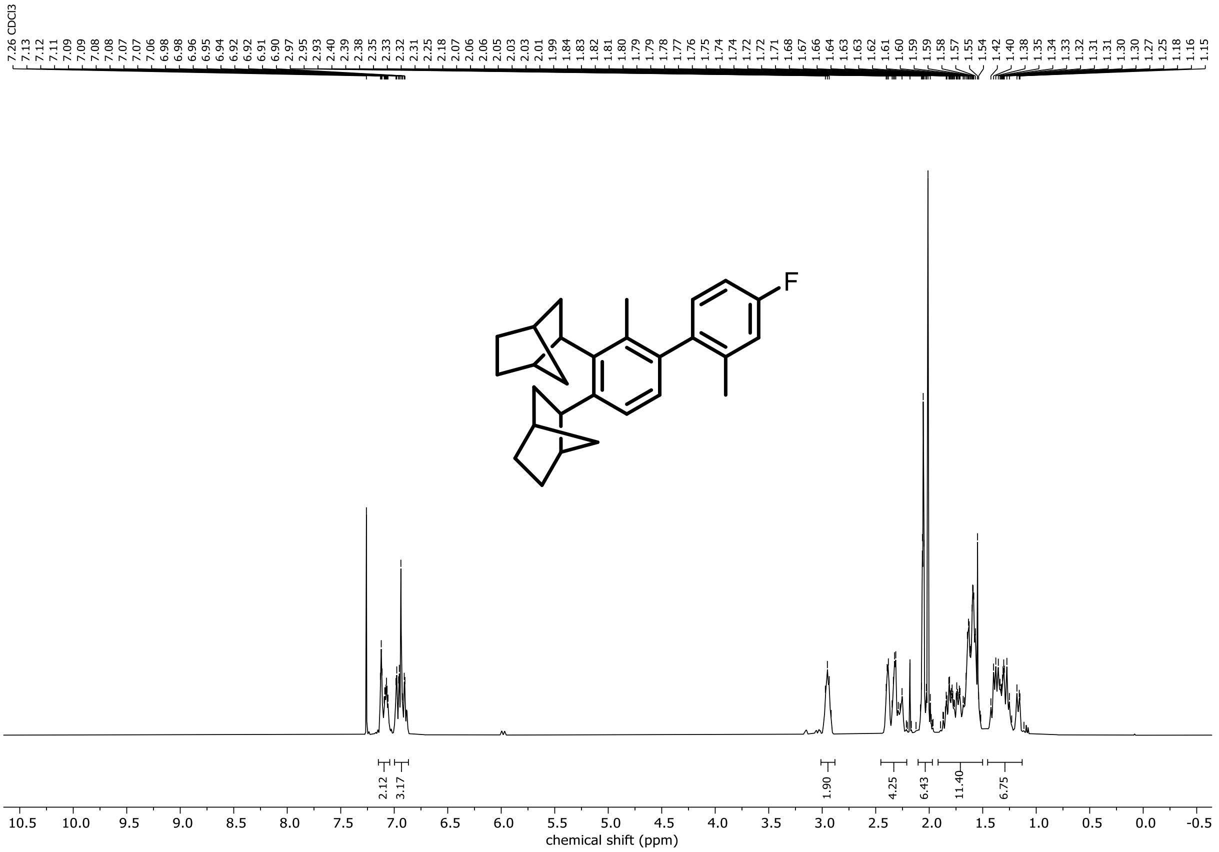

Di-substituted 8 (di-8) (^{13}C NMR, 101 MHz, $CDCl_3$)

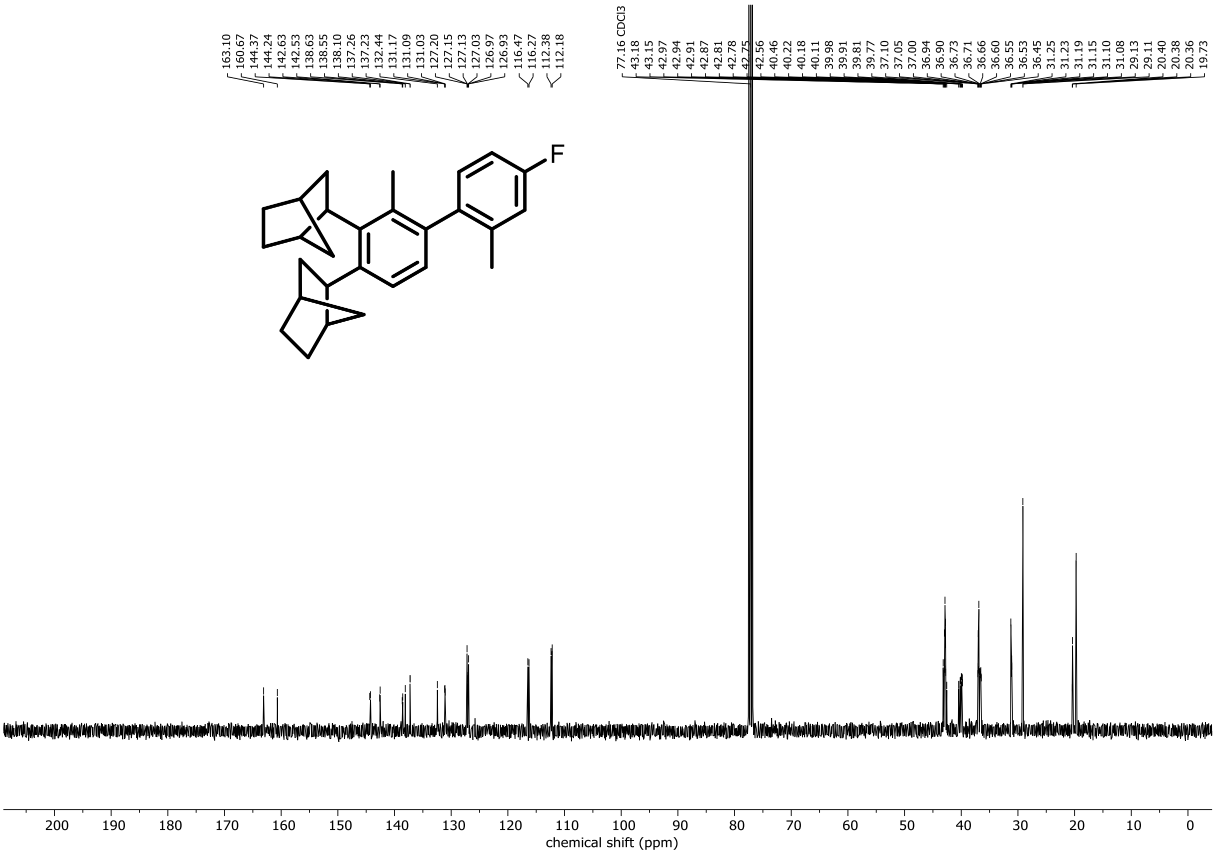

Di-substituted 8 (di-8) (^{19}F {^{1}H} NMR, 376 MHz, $CDCl_3$)

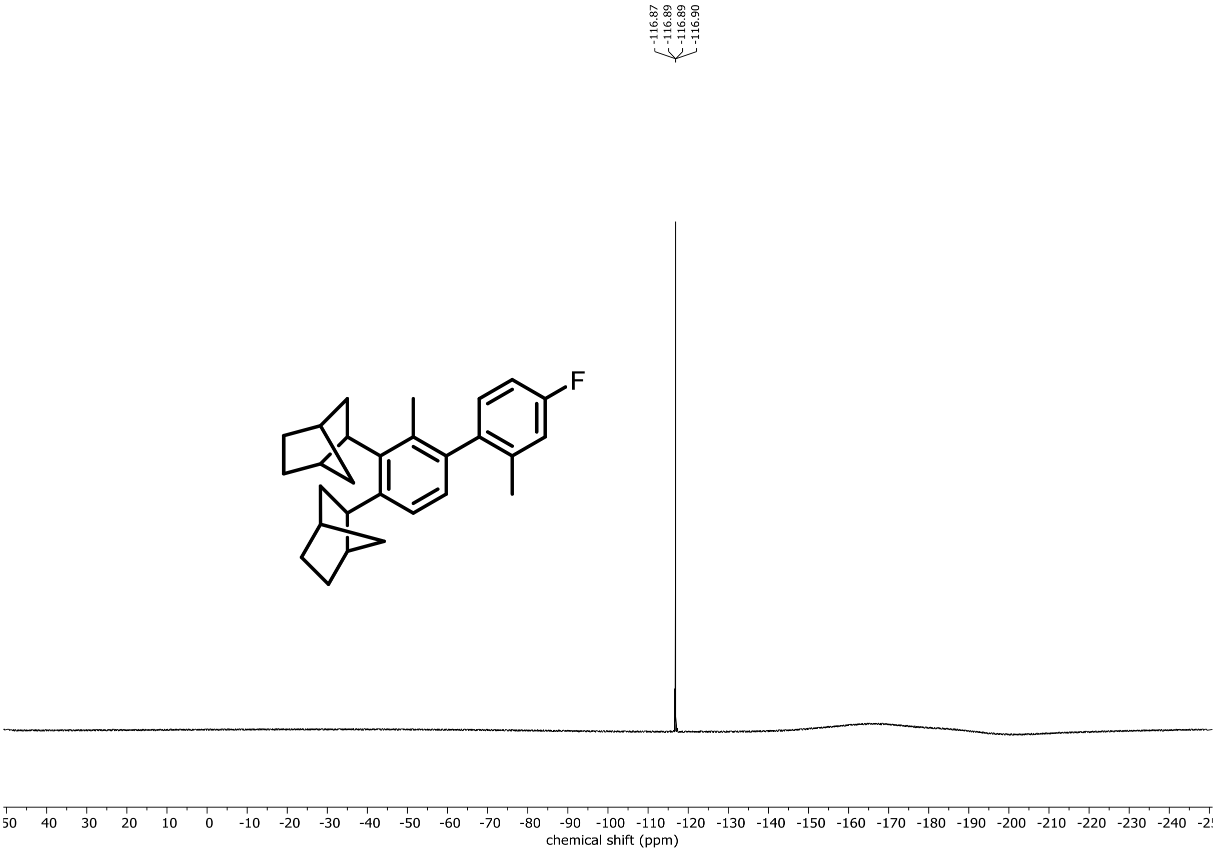

Penta-substituted 9 (penta-9) (^{1}H NMR, 600 MHz, $CDCl_3$)

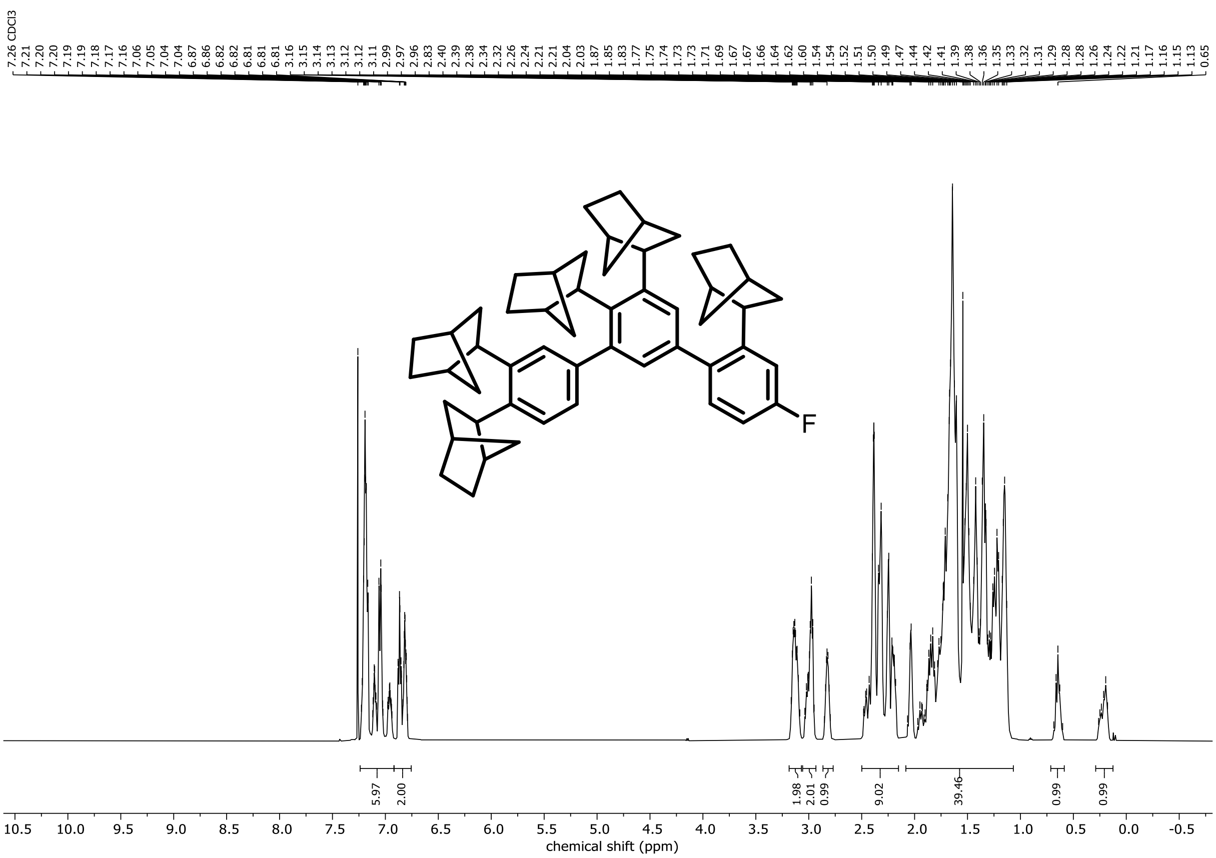

Penta-substituted 9 (penta-9) (^{13}C NMR, 151 MHz, $CDCl_3$)

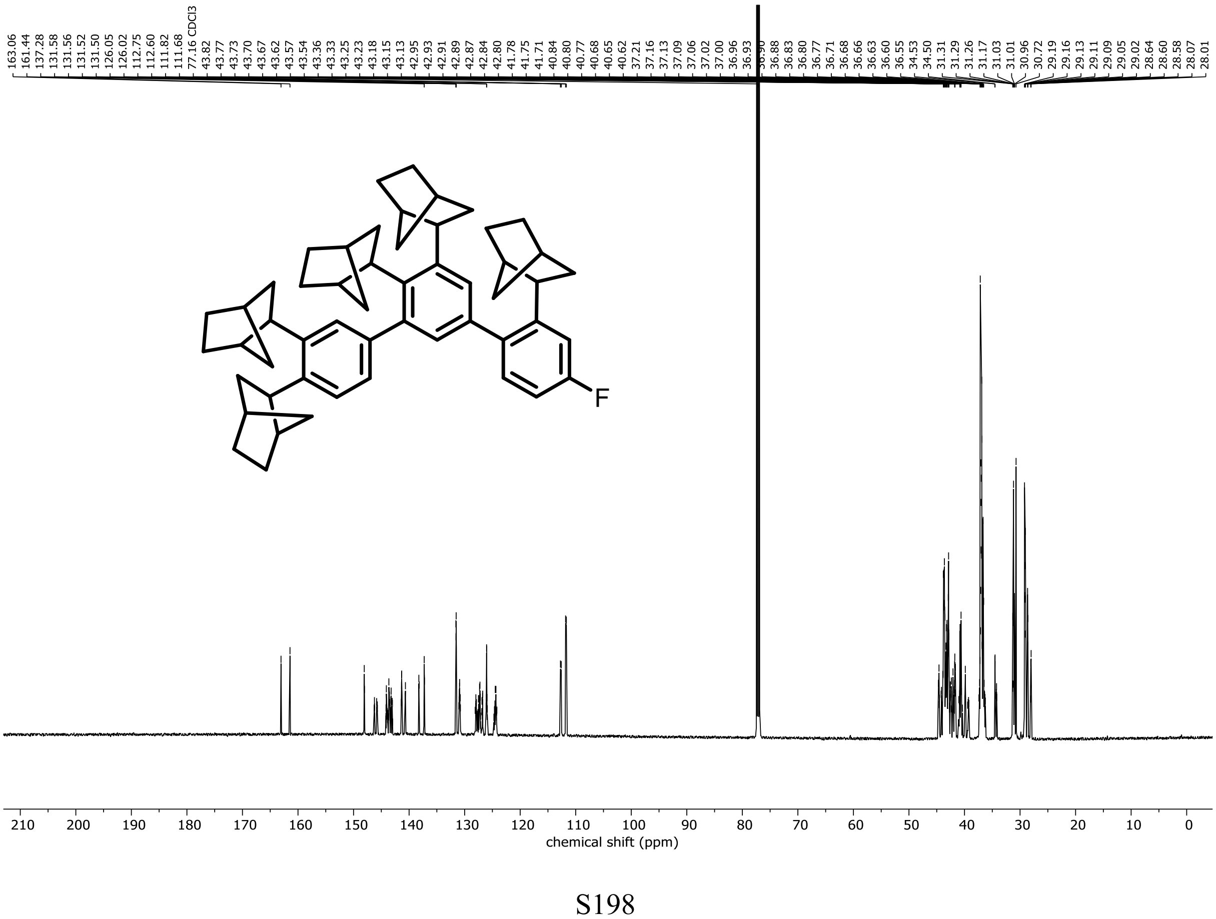

Penta-substituted 9 (penta-9) (^{19}F NMR, 376 MHz, $CDCl_3$)

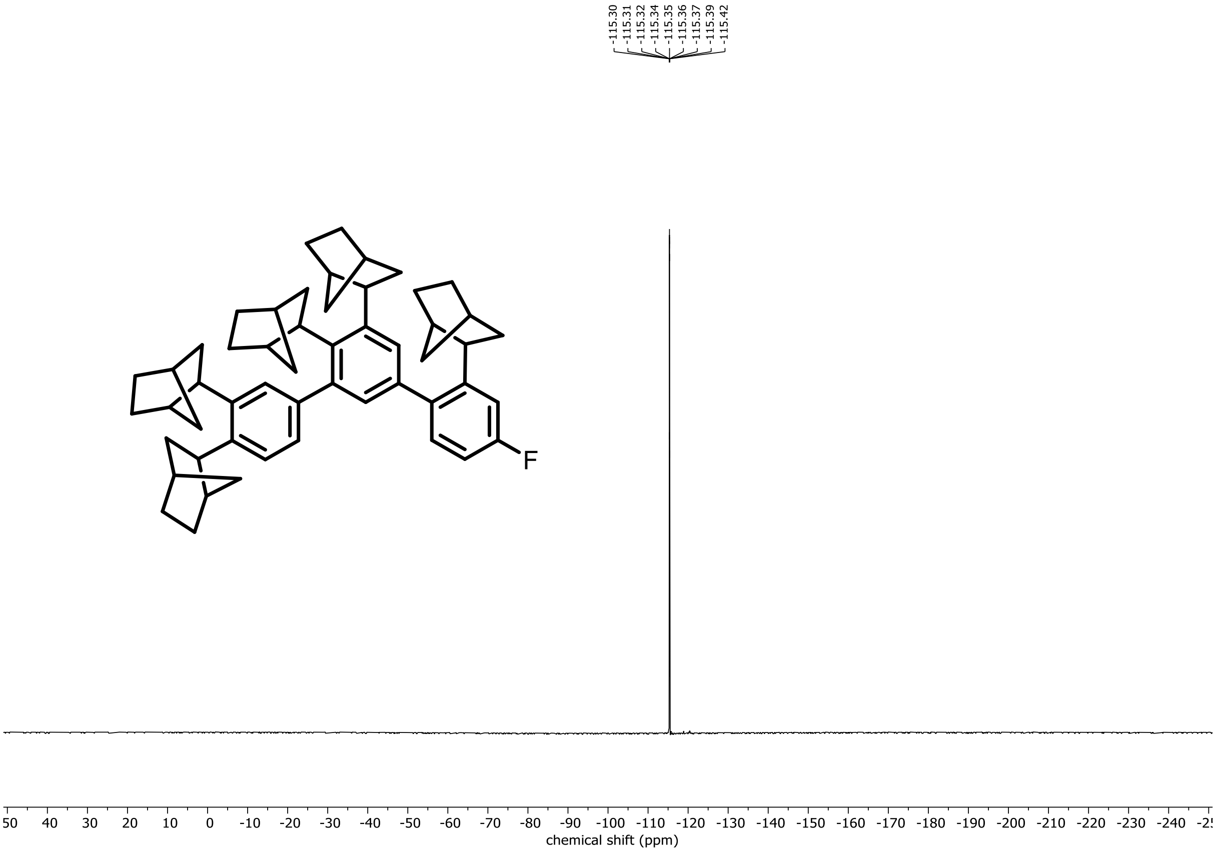

Deborylated 9 (PDB-9) (^{1}H NMR, 600 MHz, $CDCl_3$)

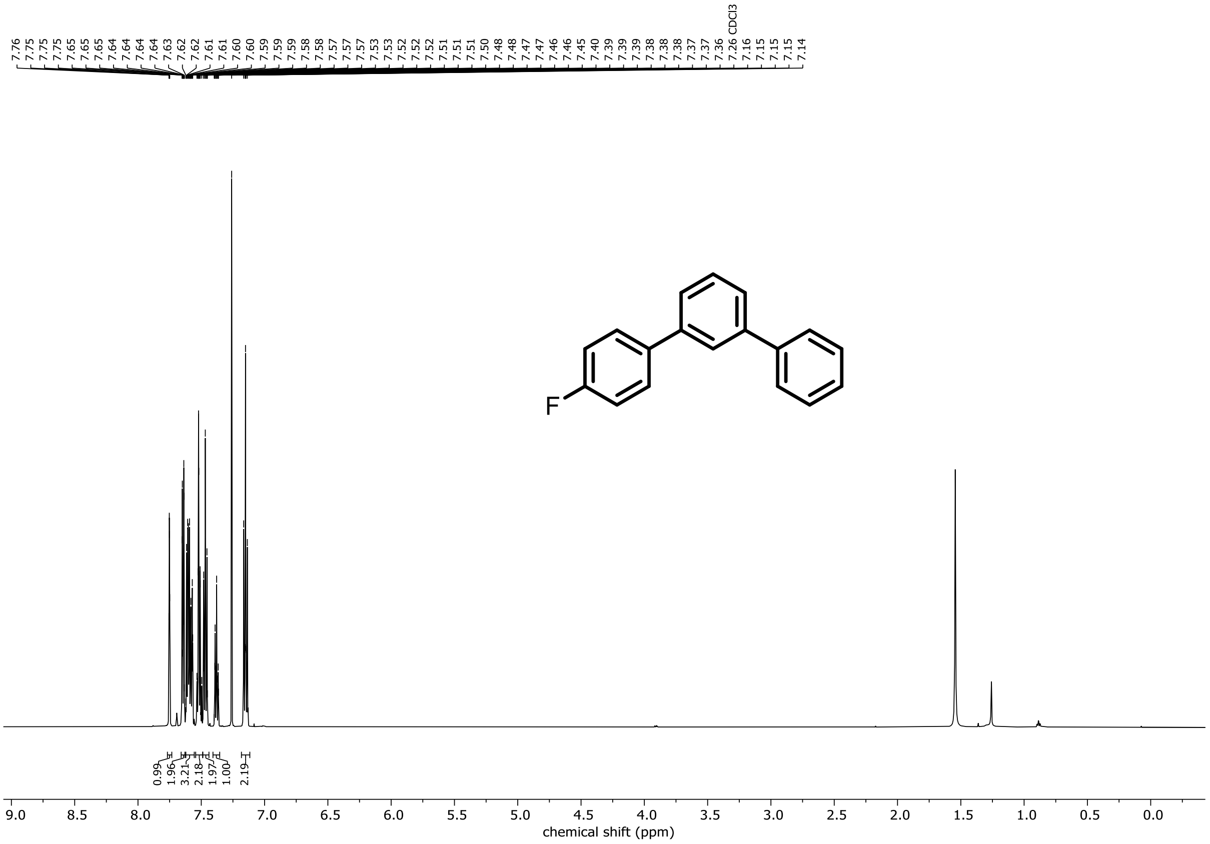

Deborylated 9 (PDB-9) (^{13}C NMR, 151 MHz, $CDCl_3$)

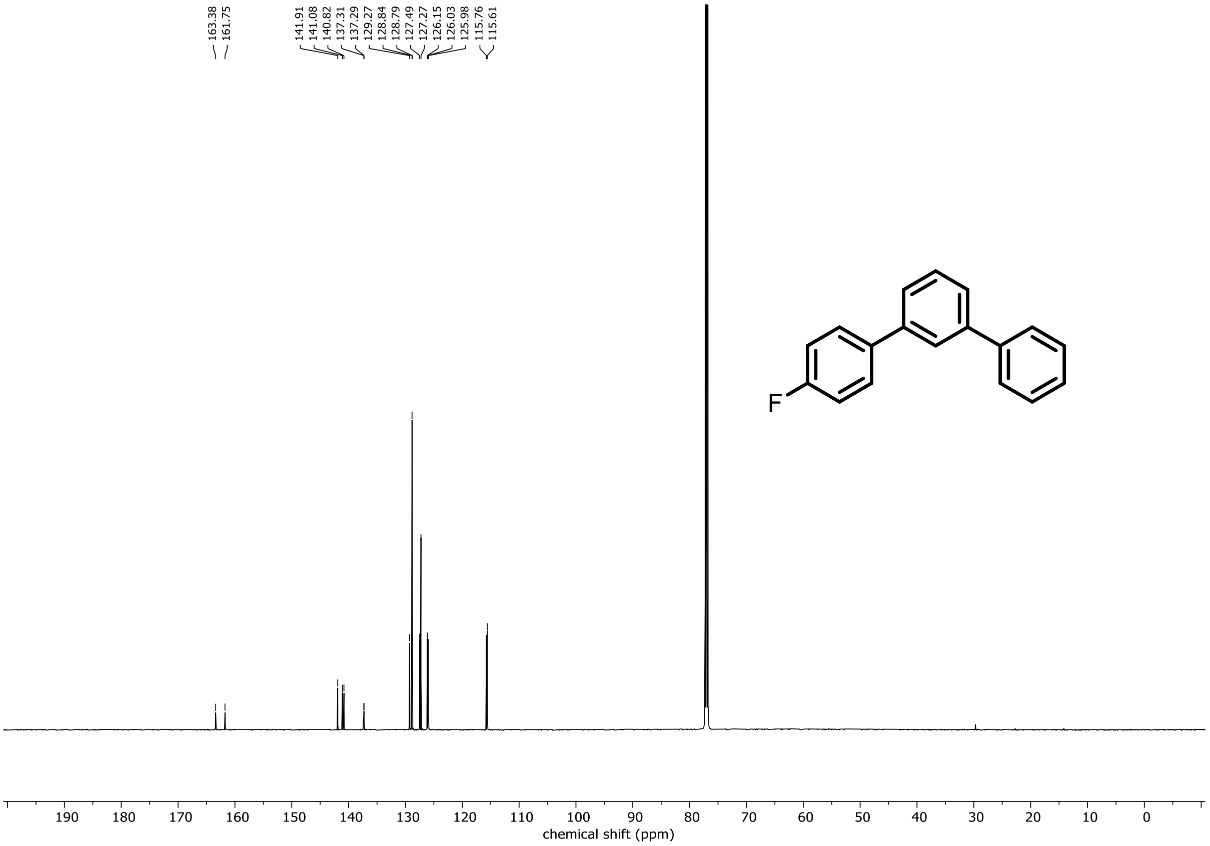

Deborylated 9 (PDB-9) (^{19}F NMR, 565 MHz, $CDCl_3$)

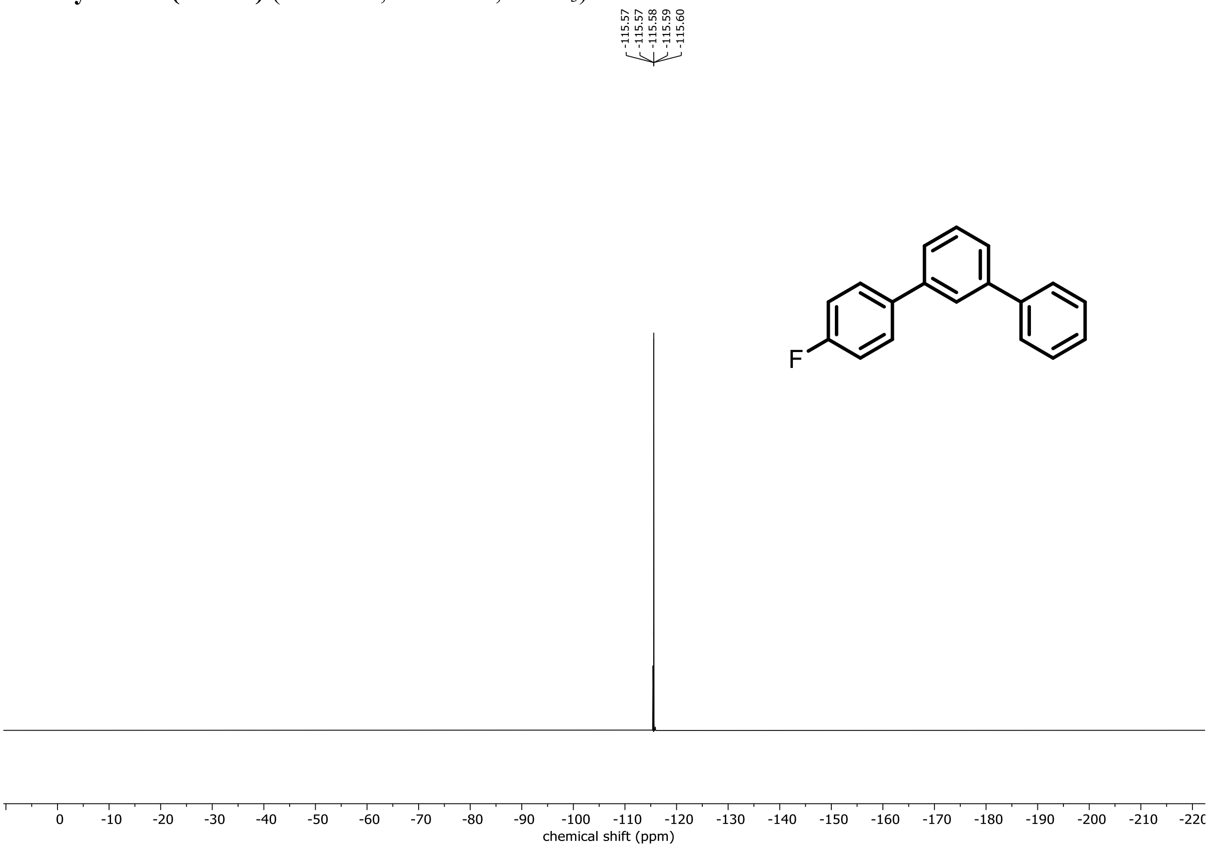

Tri-substituted 10 (tri-10) (^{1}H NMR, 400 MHz, $CDCl_3$)

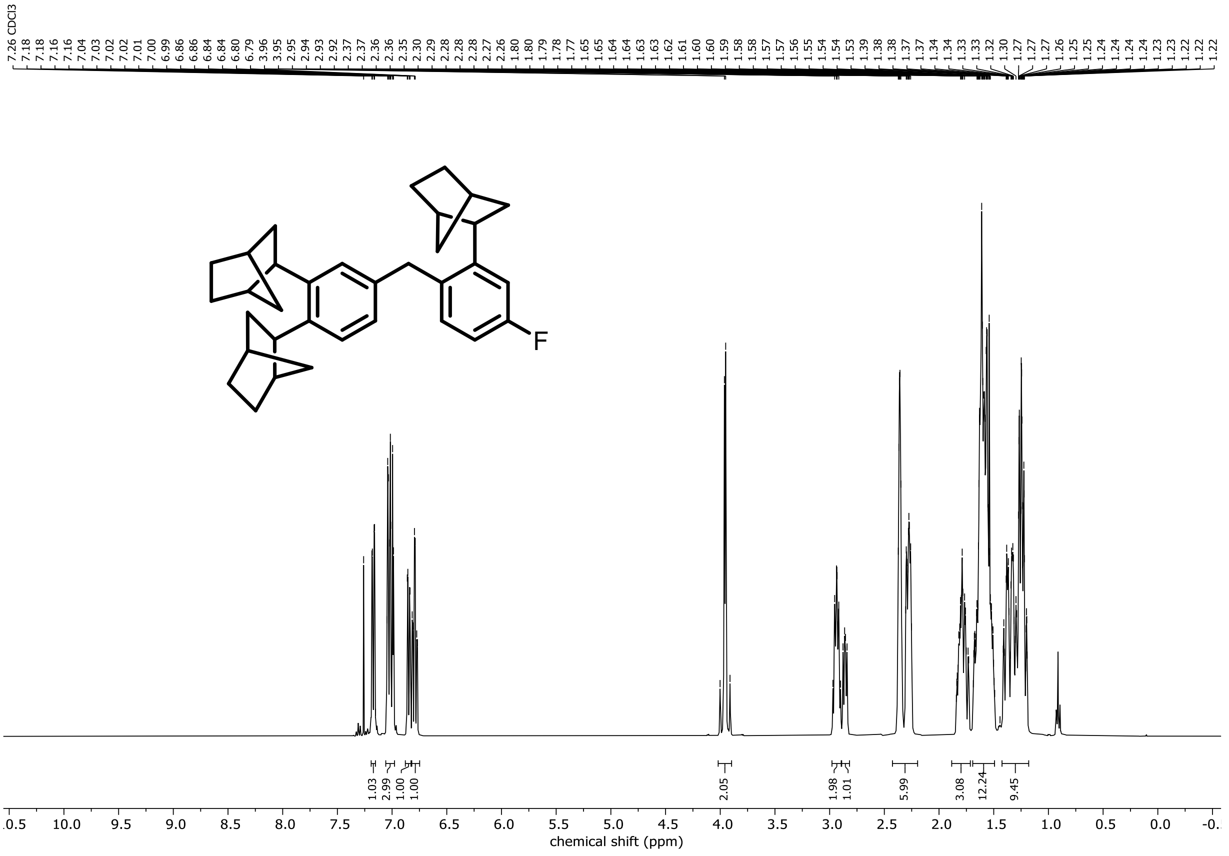

Tri-substituted 10 (tri-10) (^{13}C NMR, 101 MHz, $CDCl_3$)

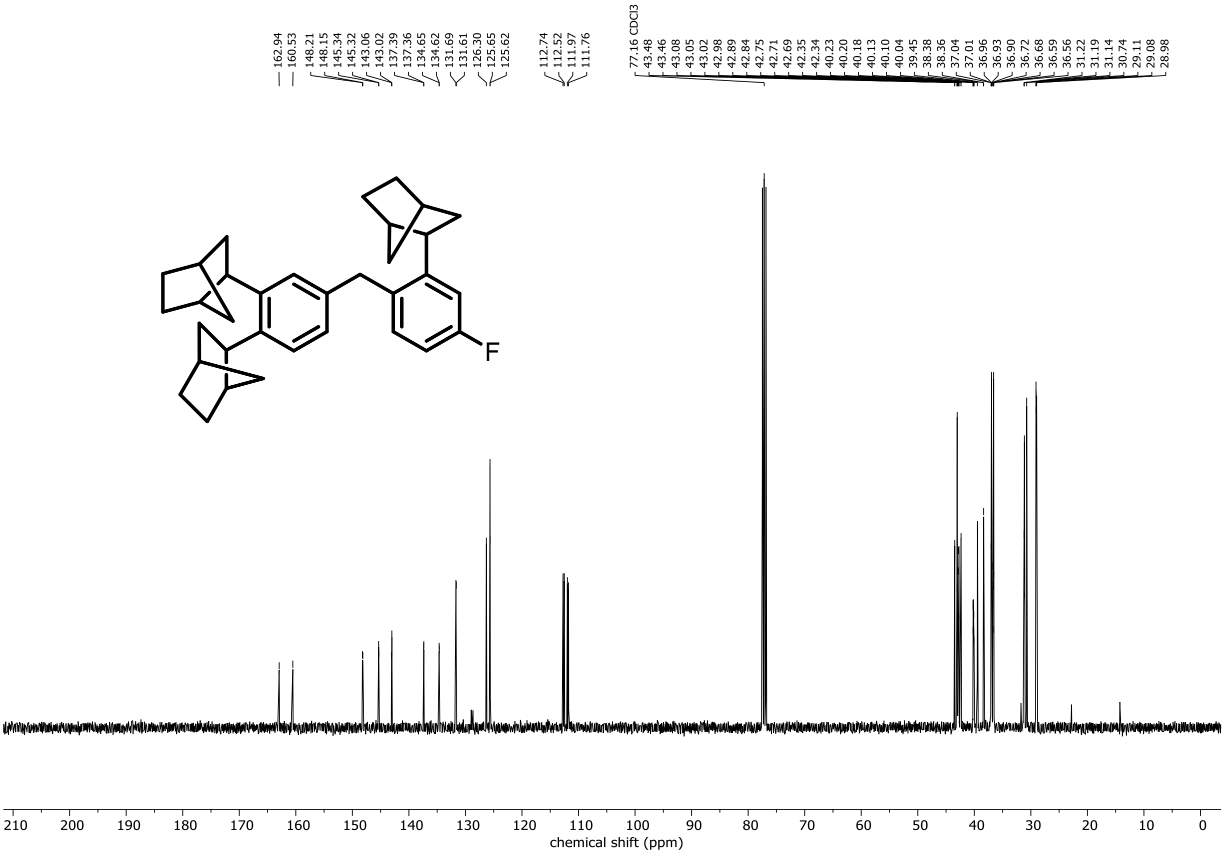

Tri-substituted 10 (tri-10) (^{19}F {^{1}H} NMR, 376 MHz, $CDCl_3$)

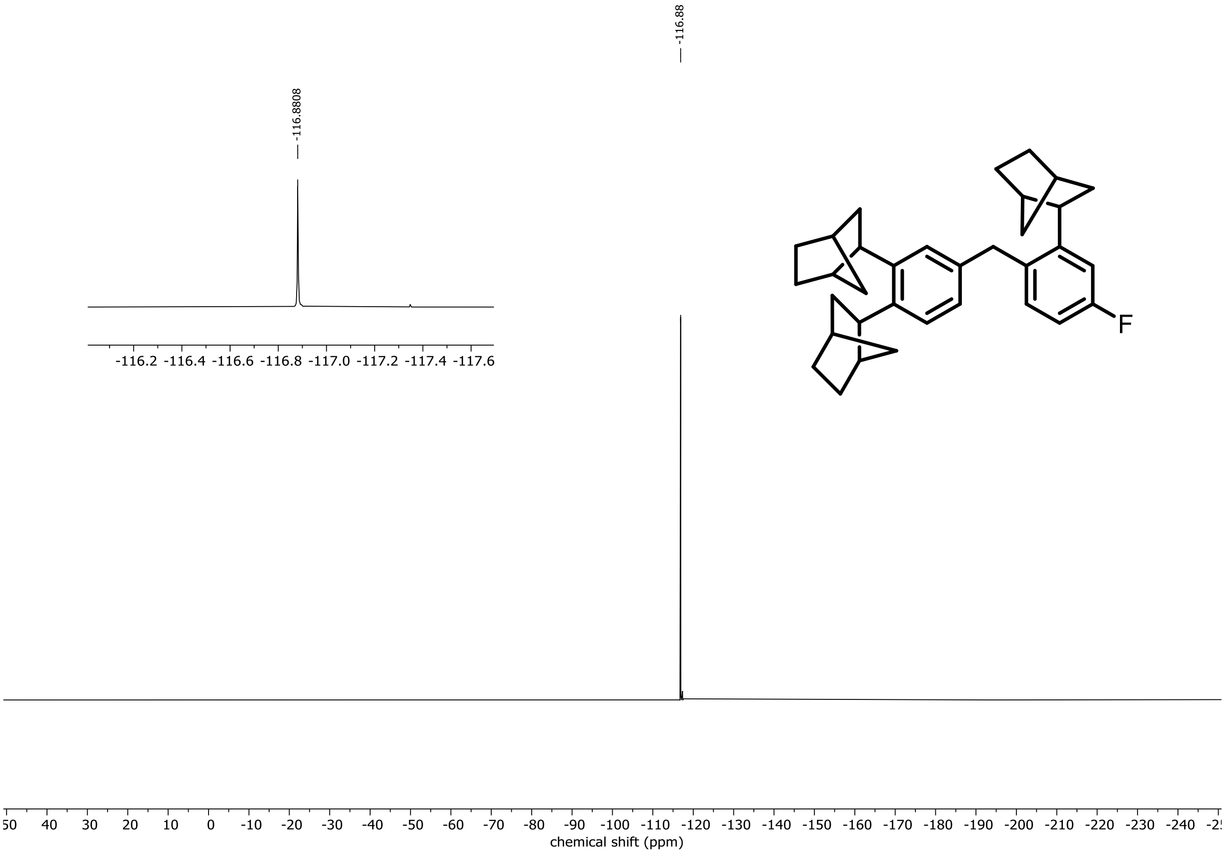

Di-substituted 10 (di-10) (^{1}H NMR, 400 MHz, CDCl$_3$)

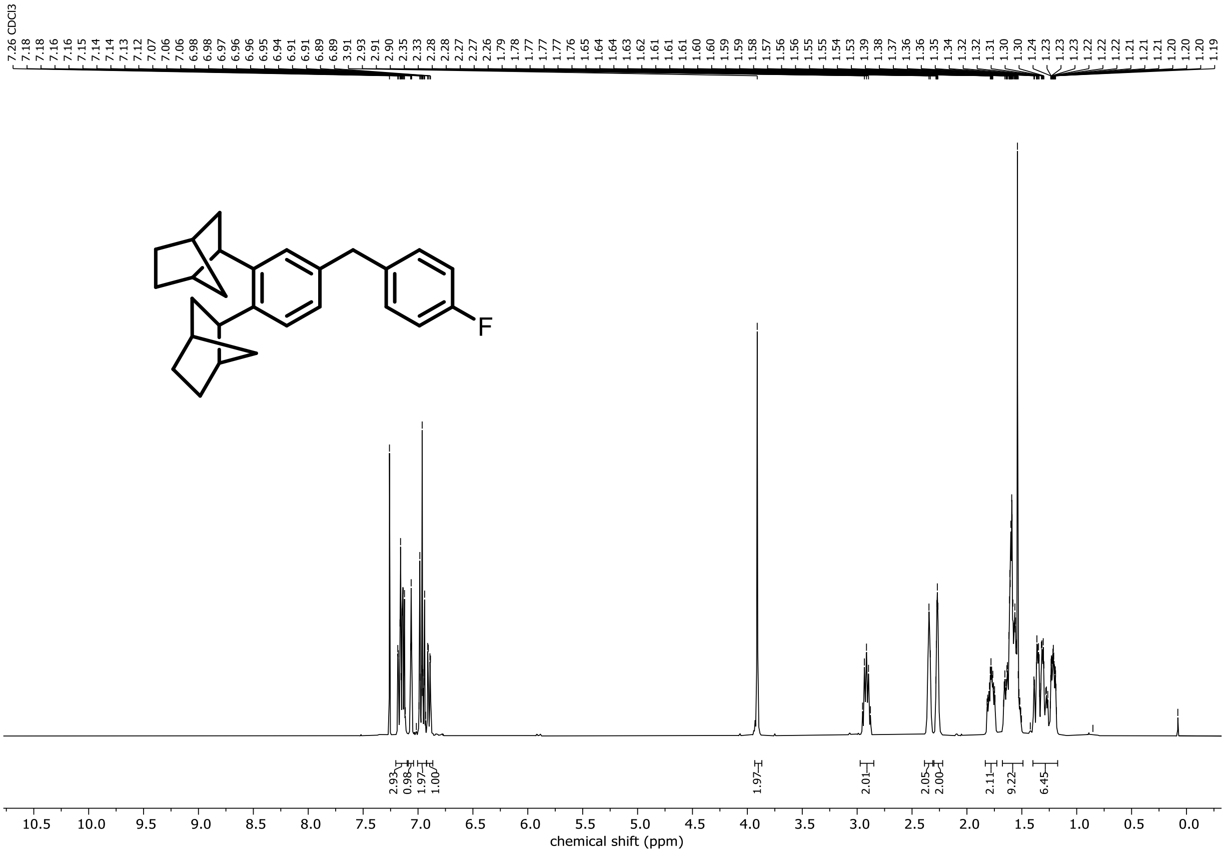

Di-substituted 10 (di-10) (^{13}C NMR, 101 MHz, CDCl$_3$)

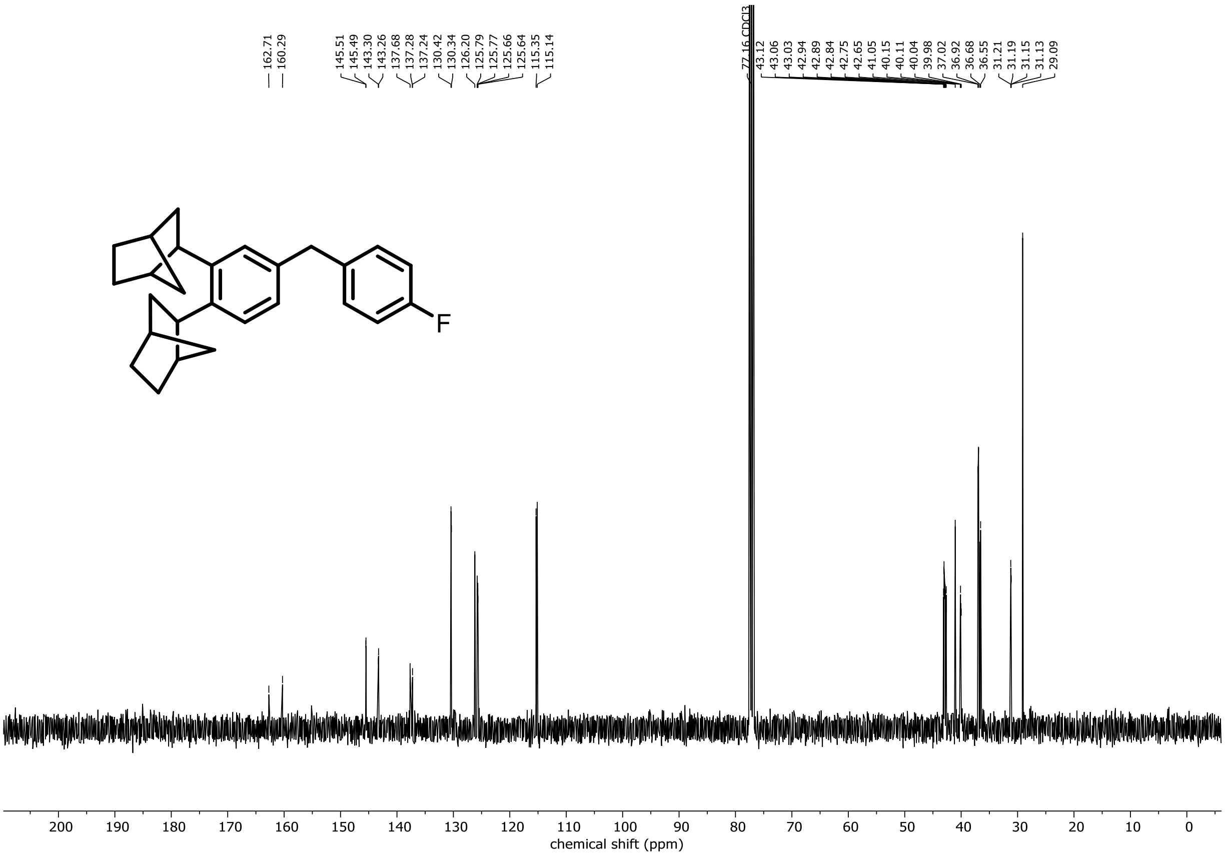

Di-substituted 10 (di-10) (^{19}F {^{1}H} NMR, 376 MHz, $CDCl_3$)

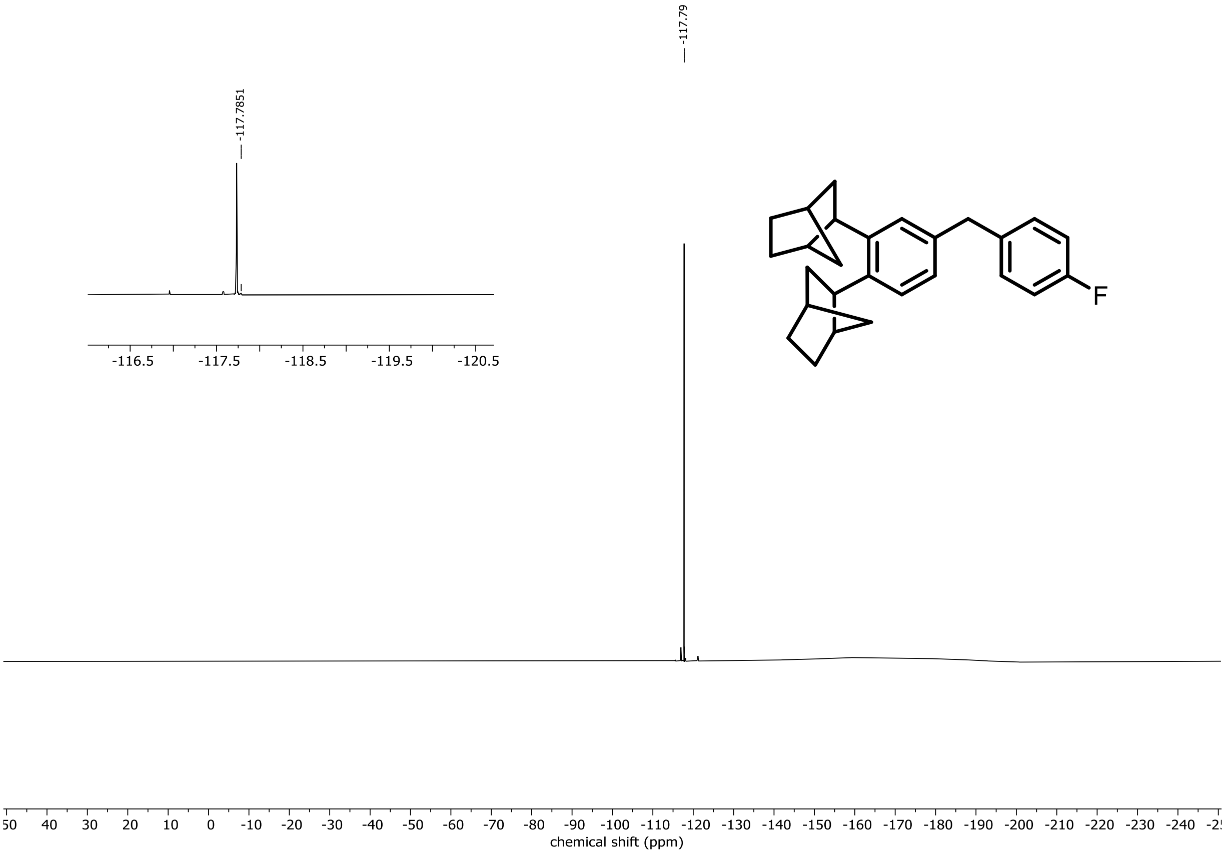

Tetra-substituted 11 (tetra-11) (^{1}H NMR, 400 MHz, $CDCl_3$)

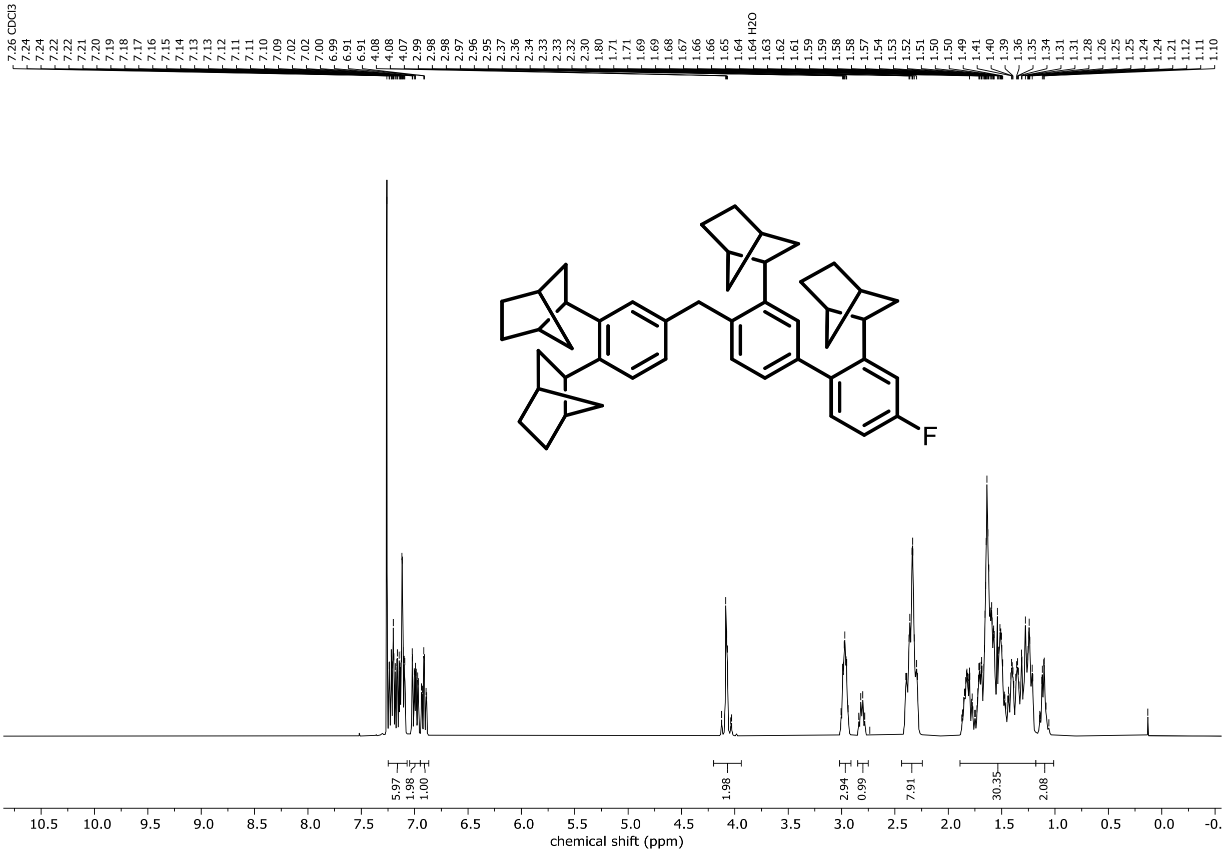

Tetra-substituted 11 (tetra-11) (^{13}C NMR, 101 MHz, $CDCl_3$)

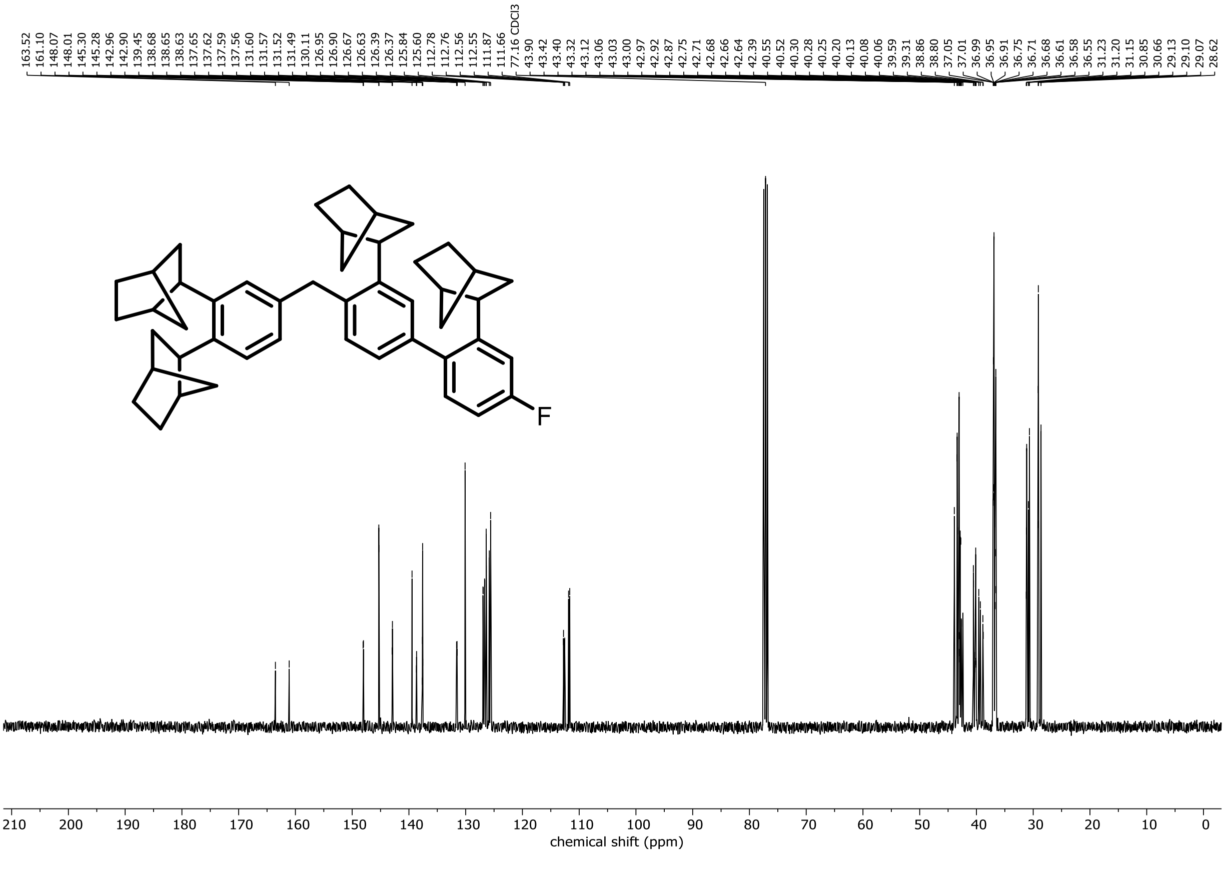

Tetra-substituted 11 (tetra-11) (^{19}F {^{1}H} NMR, 376 MHz, $CDCl_3$)

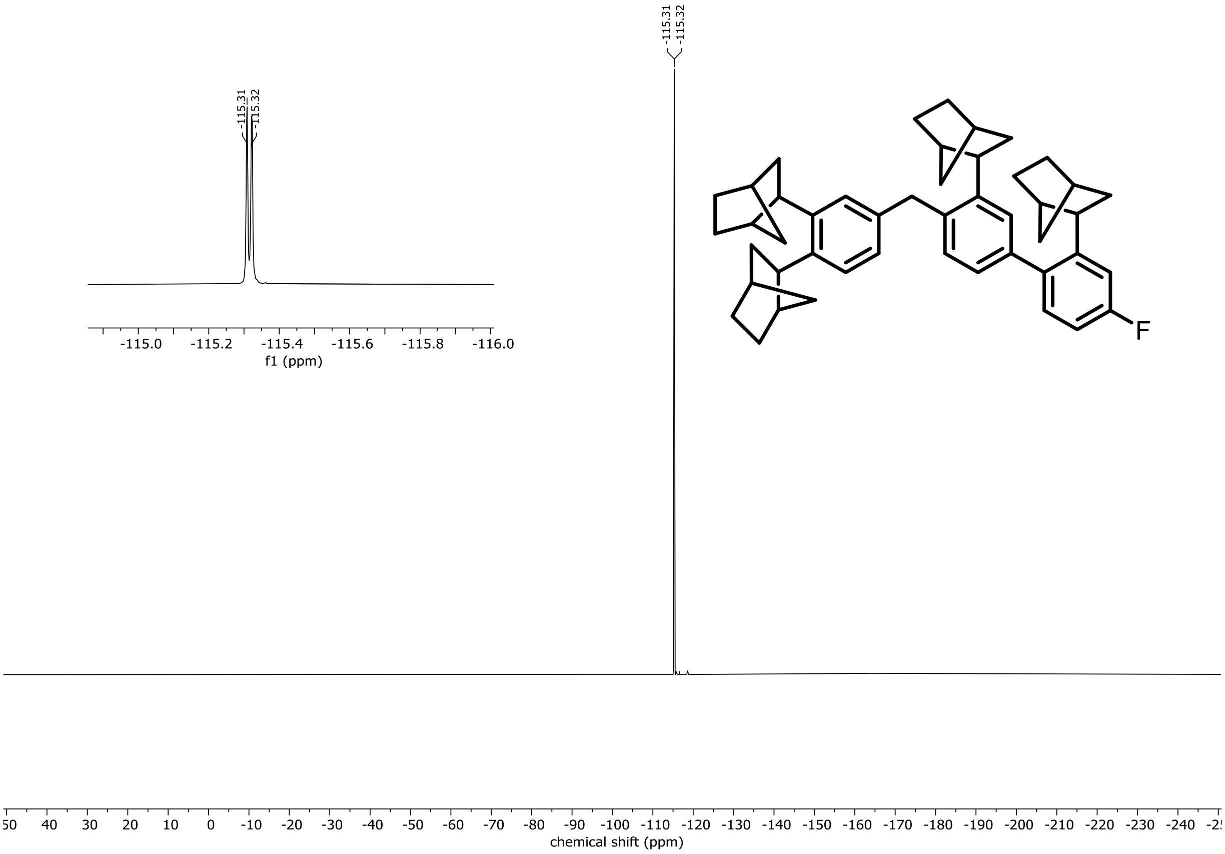

Tri-substituted 11 (tri-11) (^{1}H NMR, 600 MHz, $CDCl_3$)

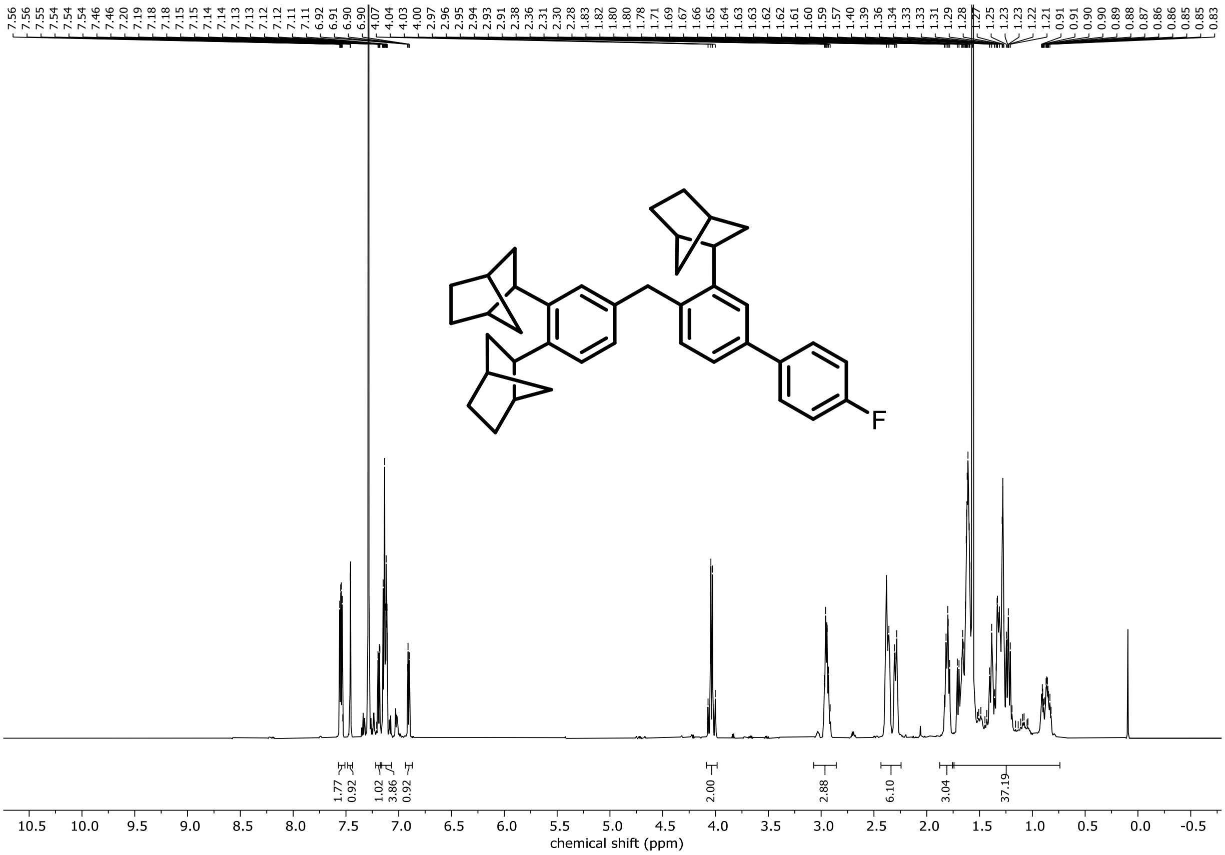

Tri-substituted 11 (tri-11) ^{13}C NMR, 151 MHz, $CDCl_3$)

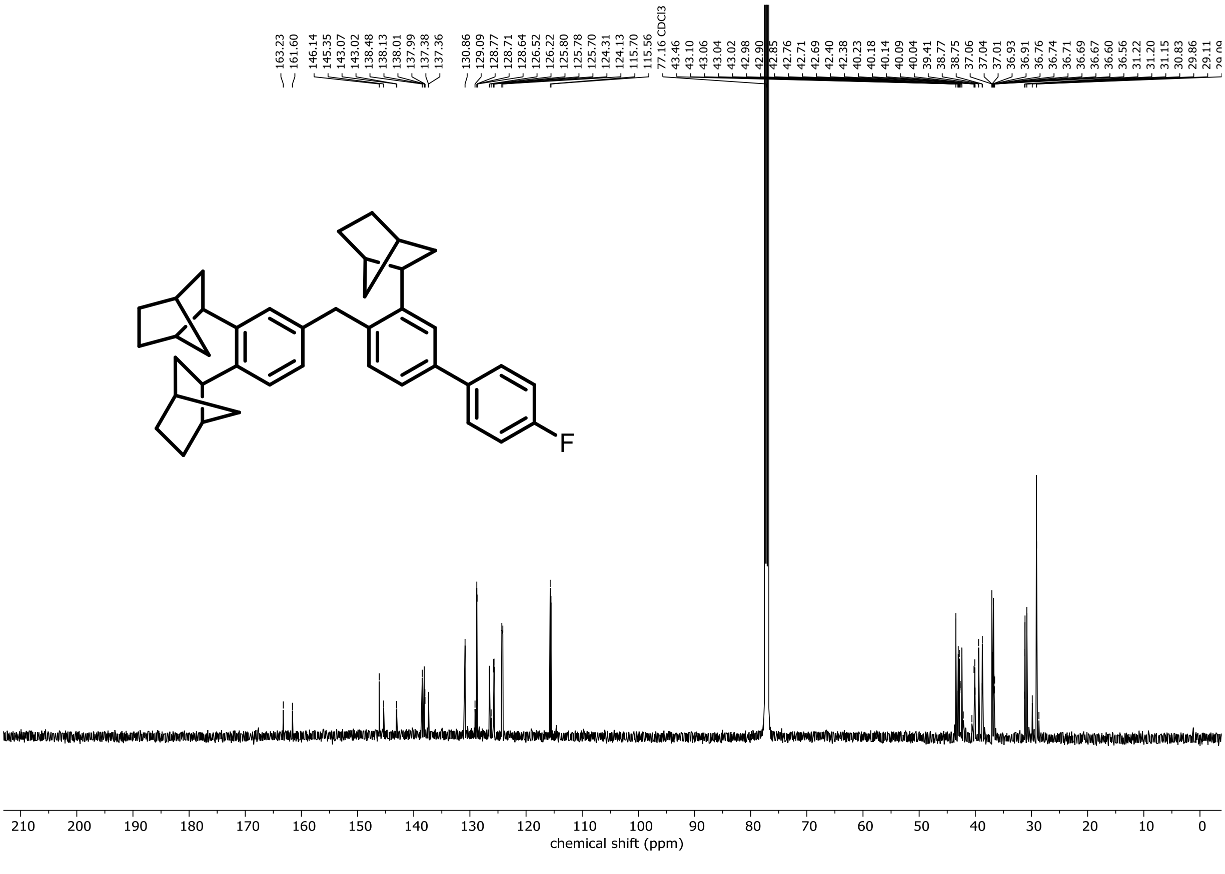

Tri-substituted 11 (tri-11) ($^{19}F\ \{^{1}H\}$ NMR, 376 MHz, $CDCl_3$)

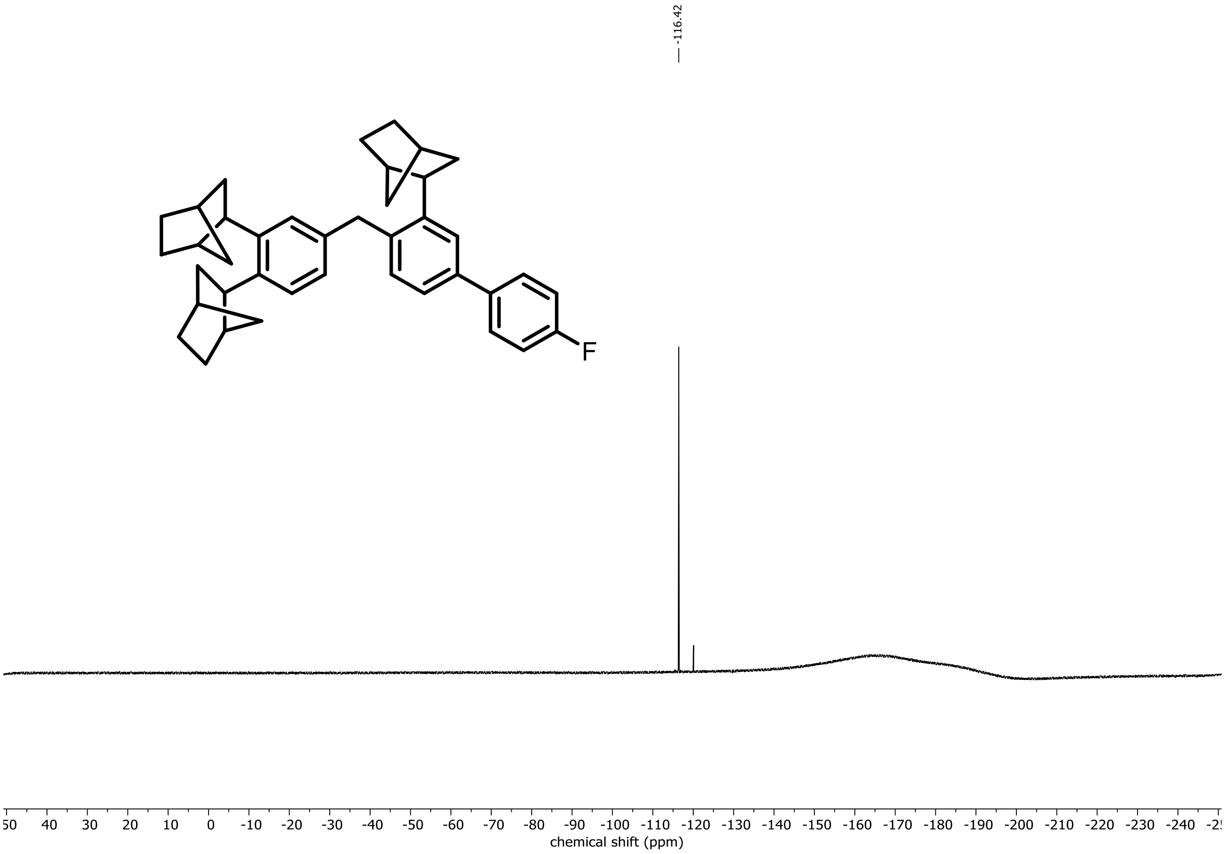

Tetra-substituted 12 (tetra-12) (^{1}H NMR, 400 MHz, $CDCl_3$)

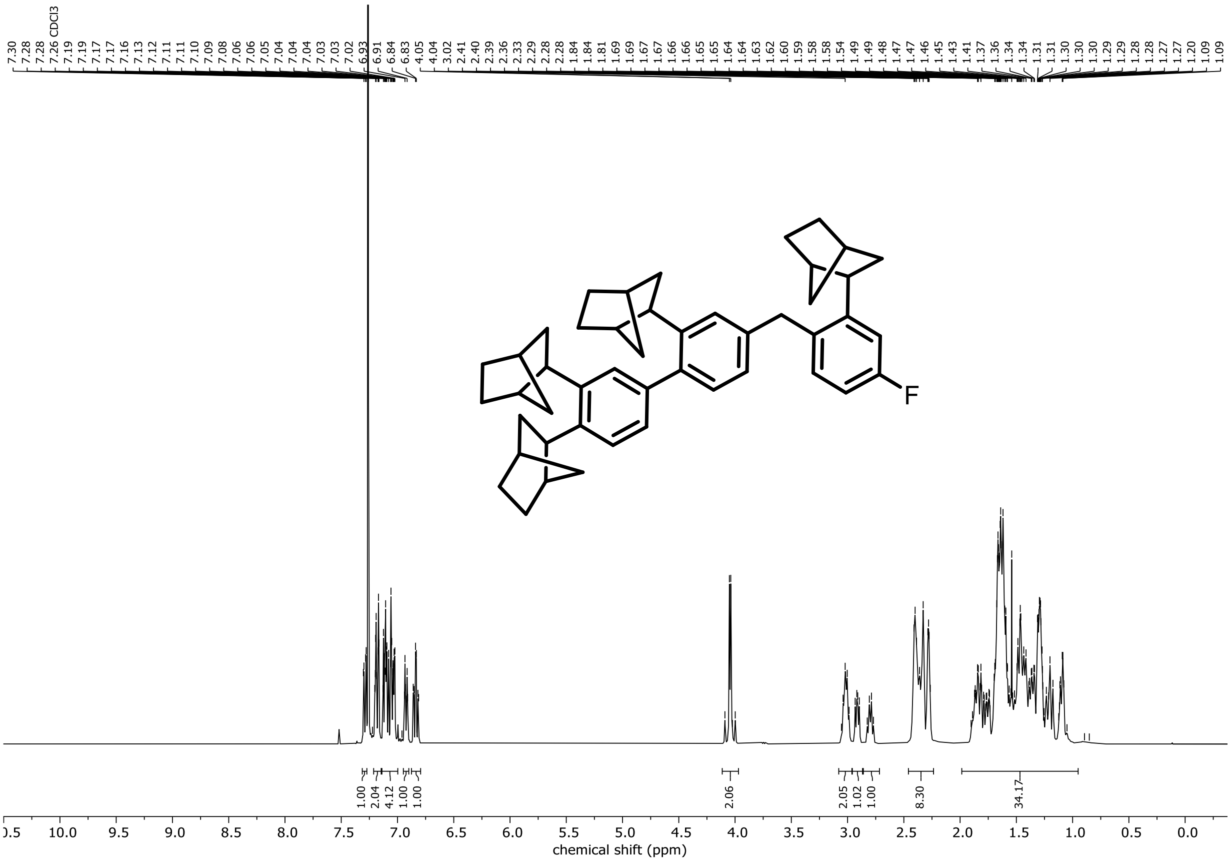

Tetra-substituted 12 (tetra-12) **(**^{13}C NMR, 101 MHz, $CDCl_3$)

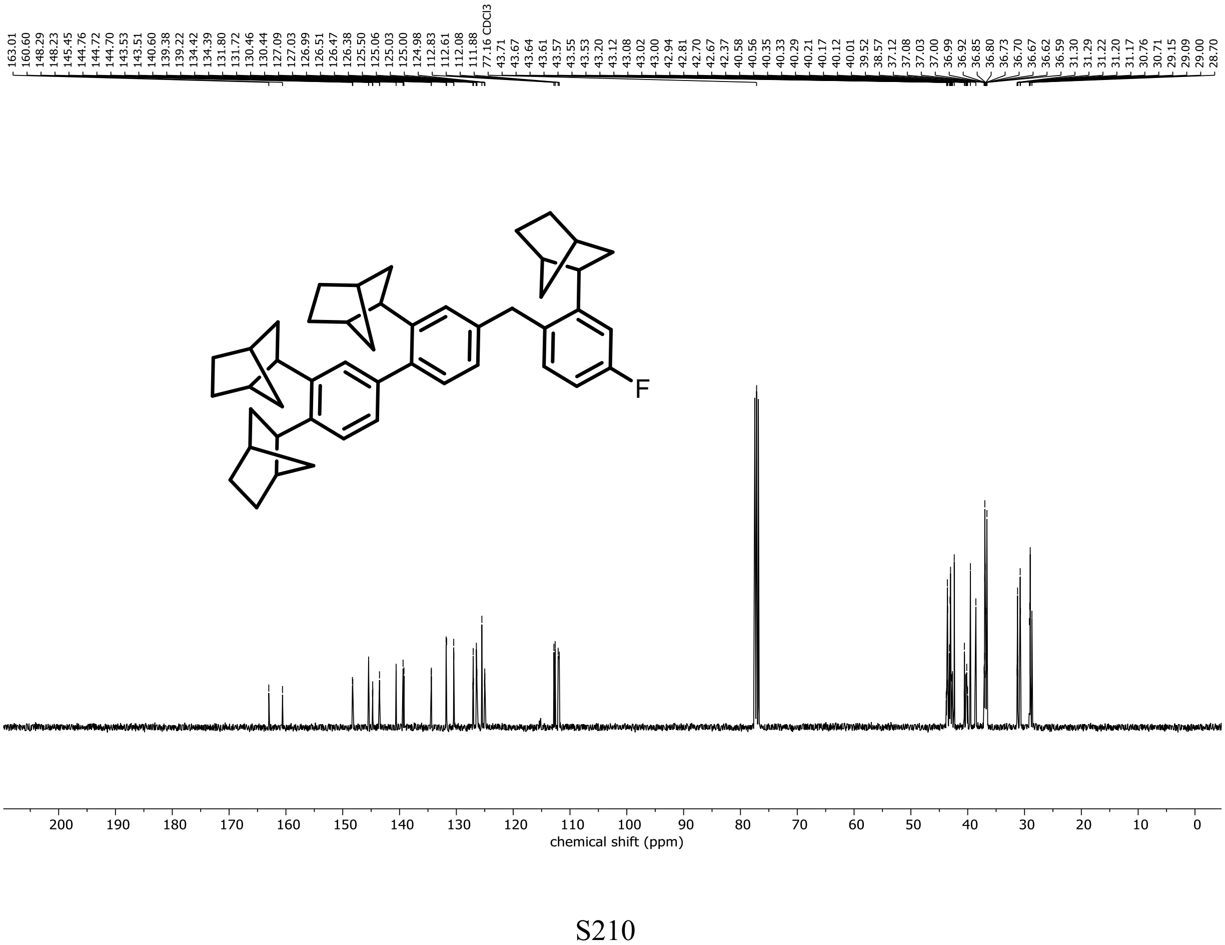

Tetra-substituted 12 (tetra-12) (^{19}F {^{1}H} NMR, 376 MHz, $CDCl_3$)

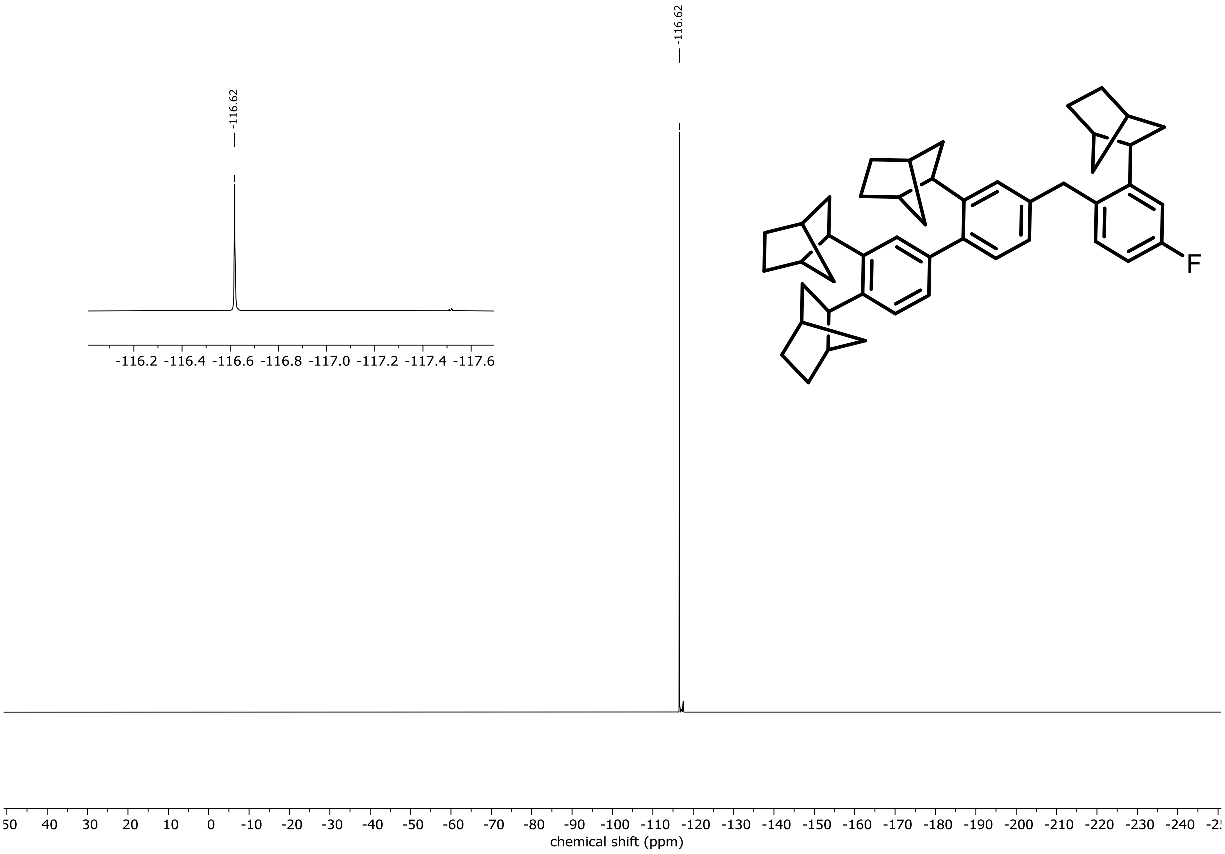

Tri-substituted 12 (tri-12) (^{1}H NMR, 500 MHz, $CDCl_3$)

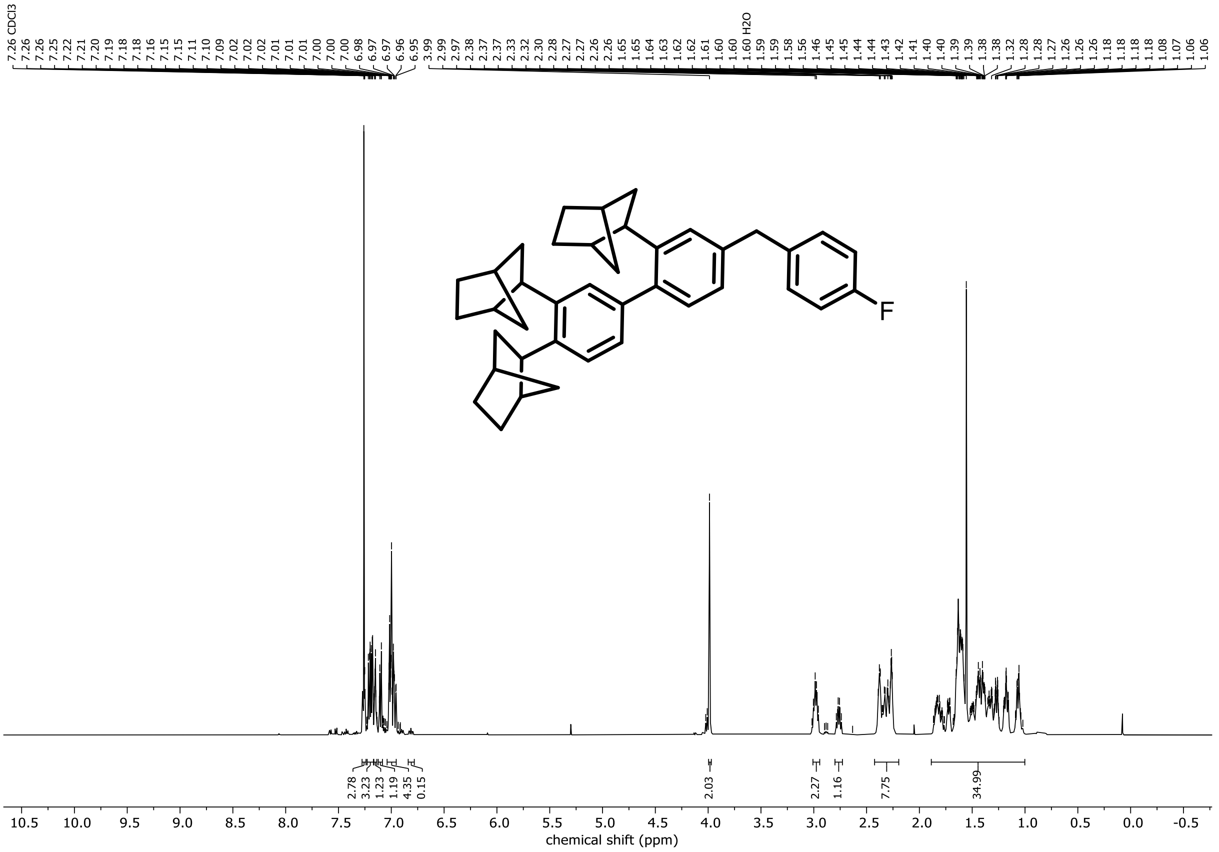

Tri-substituted 12 (tri-12) (^{13}C NMR, 126 MHz, $CDCl_3$)

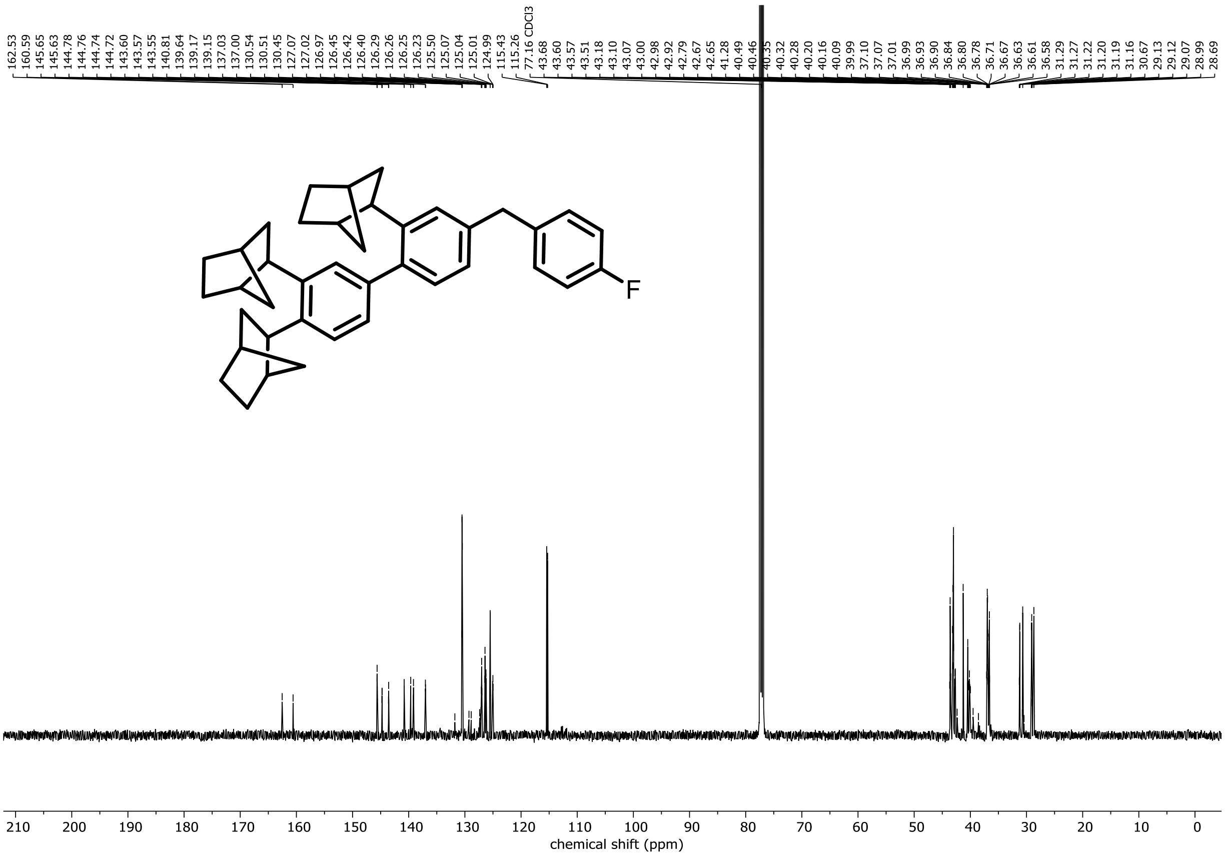

Tri-substituted 12 (tri-12) (^{19}F {^{1}H} NMR, 376 MHz, $CDCl_3$)

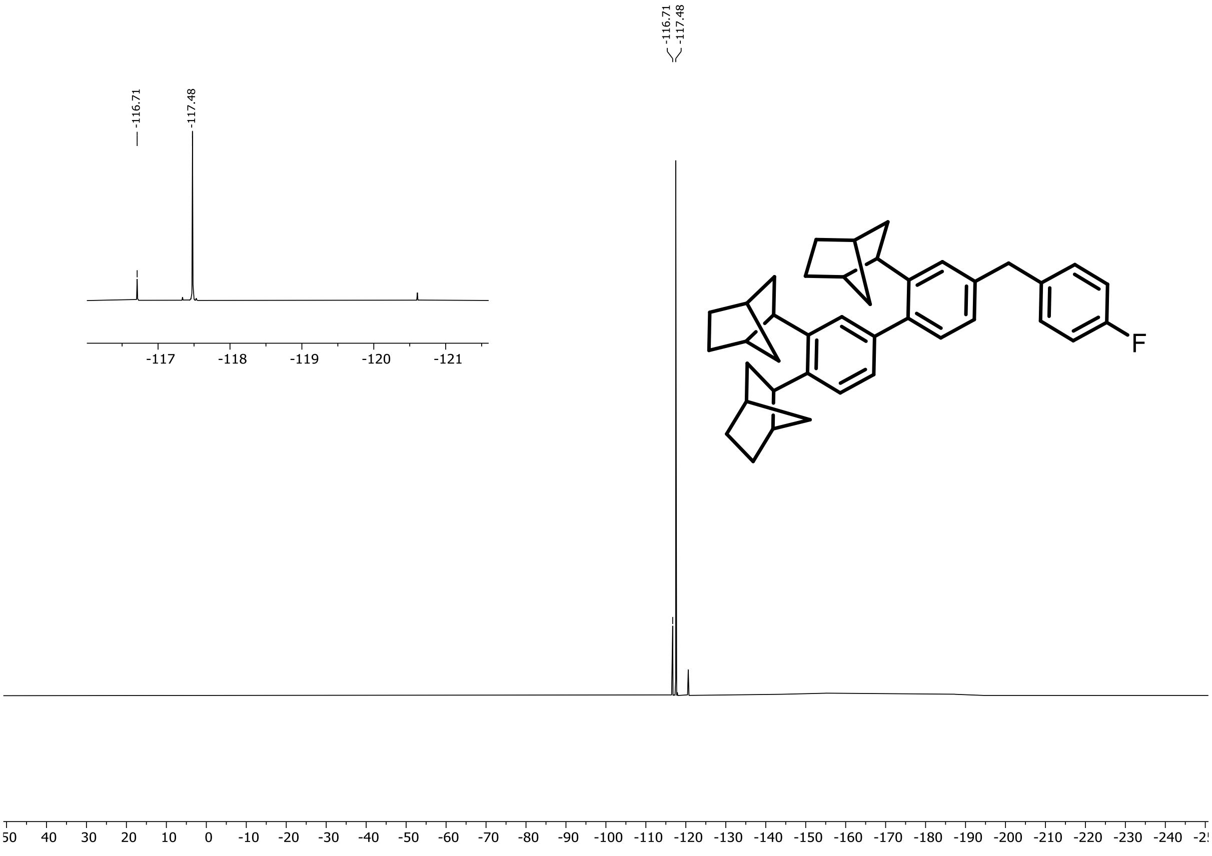

Tri-substituted 13 (tri-13) (^{1}H NMR, 500 MHz, $CDCl_3$)

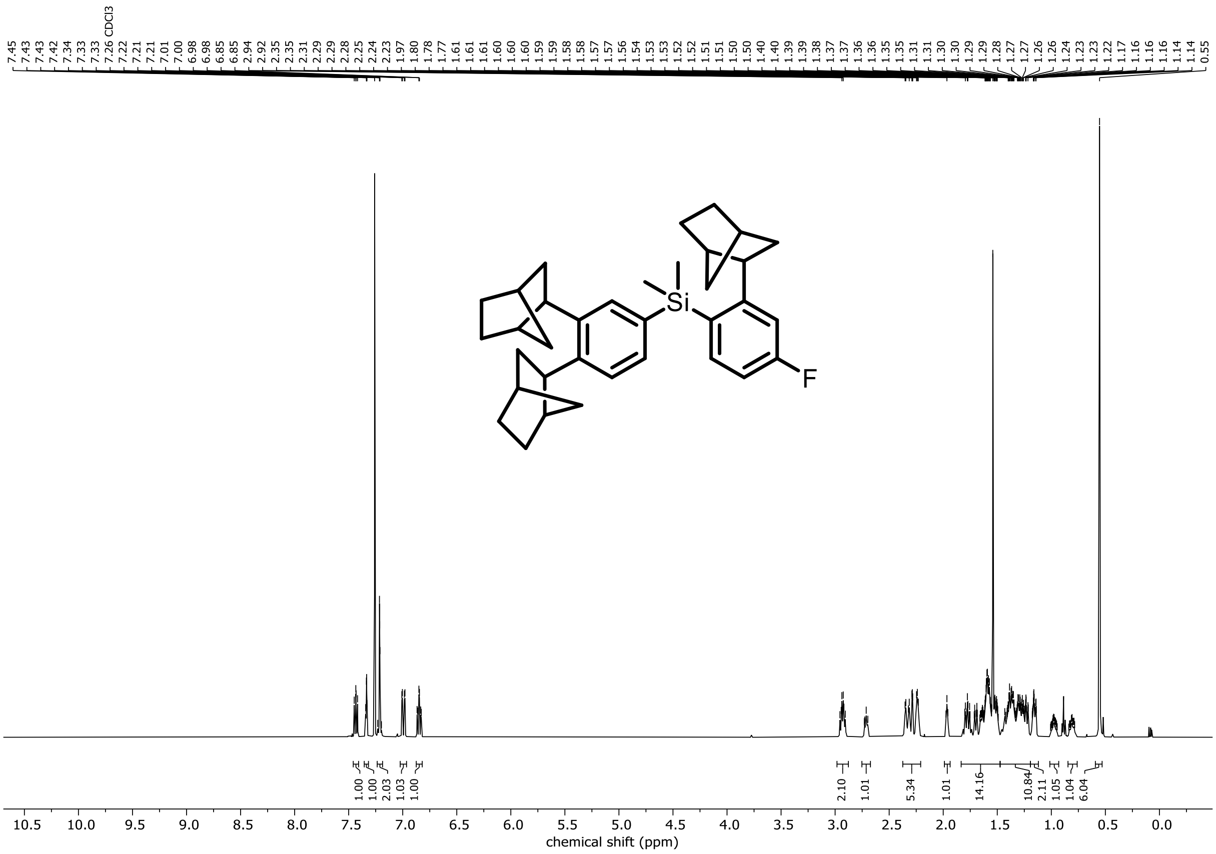

Tri-substituted 13 (tri-13) (^{13}C NMR, 126 MHz, $CDCl_3$)

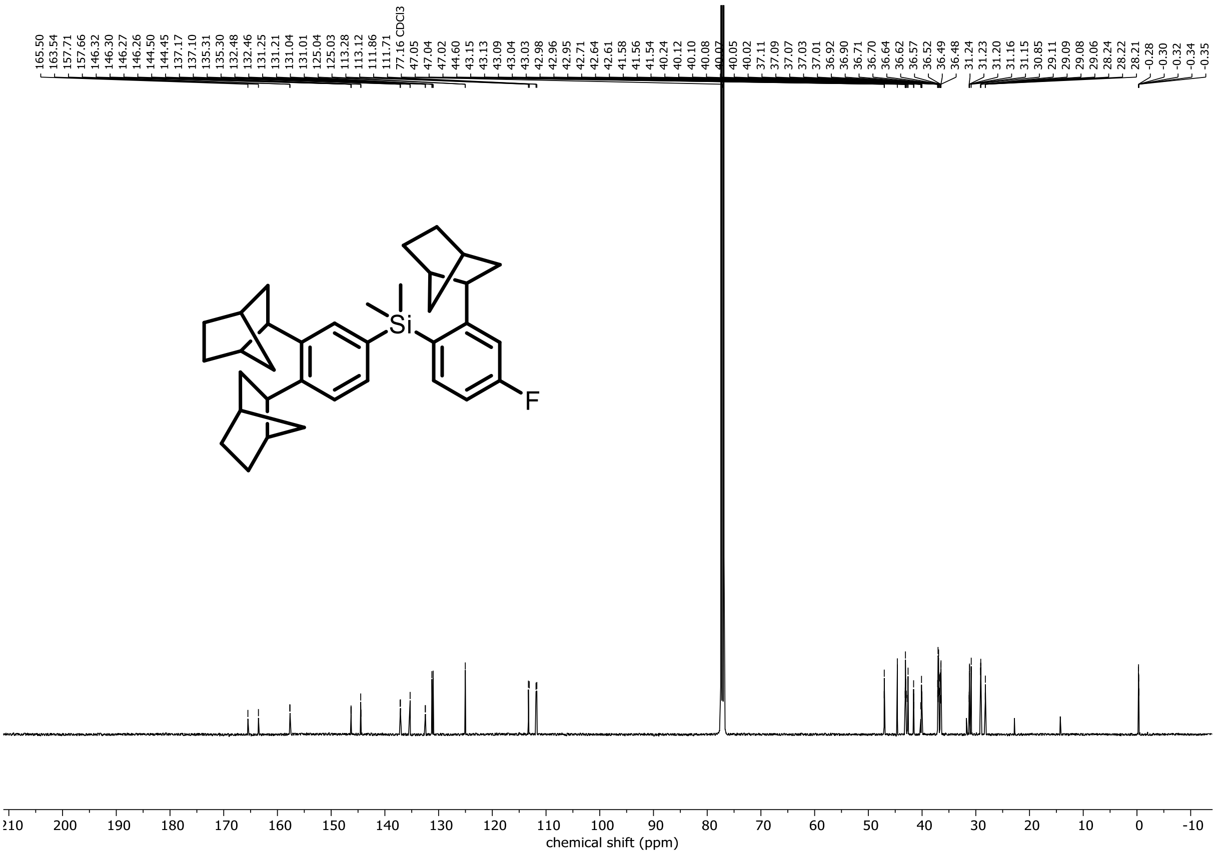

Tri-substituted 13 (tri-13) (^{19}F {^{1}H} NMR, 376 MHz, $CDCl_3$)

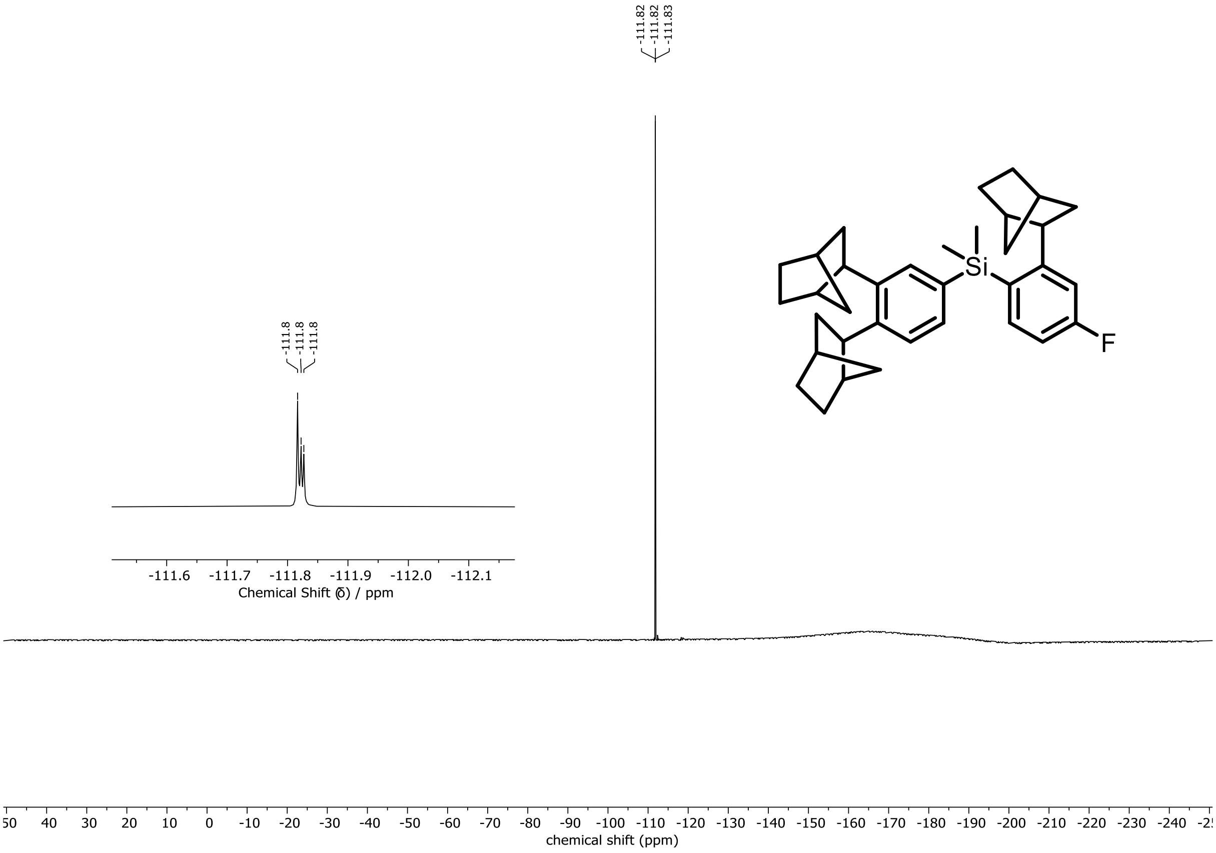

Tri-substituted 14 (tri-14) (^{1}H NMR, 400 MHz, $CDCl_3$)

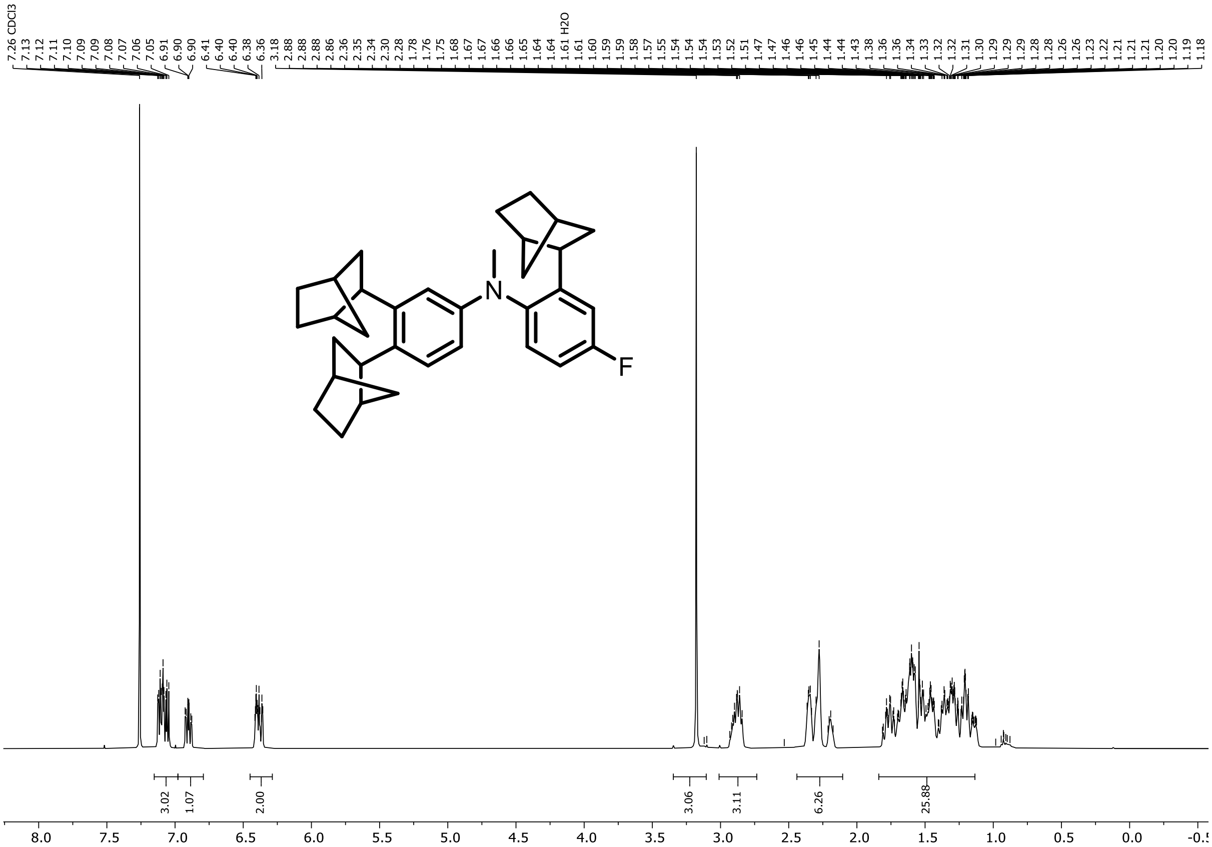

Tri-substituted 14 (tri-14) (^{13}C NMR, 101 MHz, $CDCl_3$)

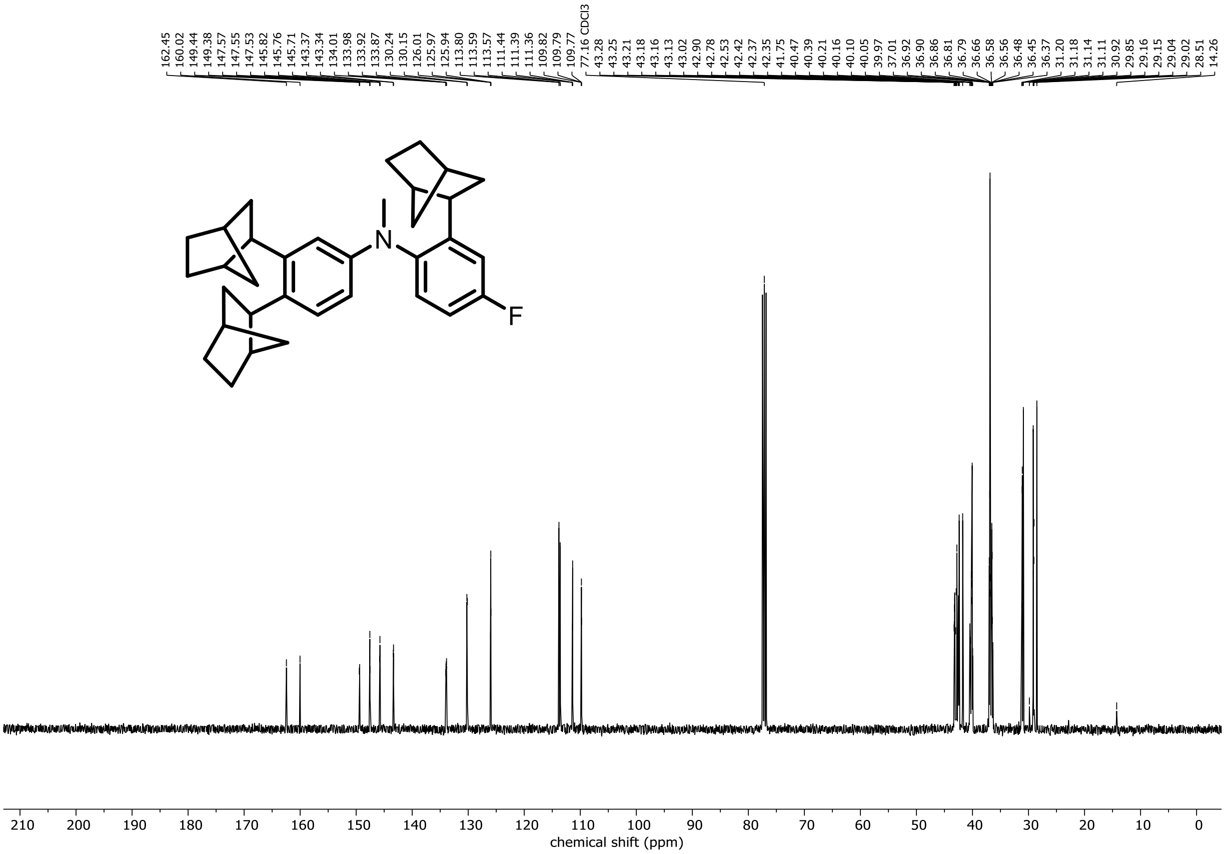

Tri-substituted 14 (tri-14) (^{19}F {^{1}H} NMR, 376 MHz, $CDCl_3$)

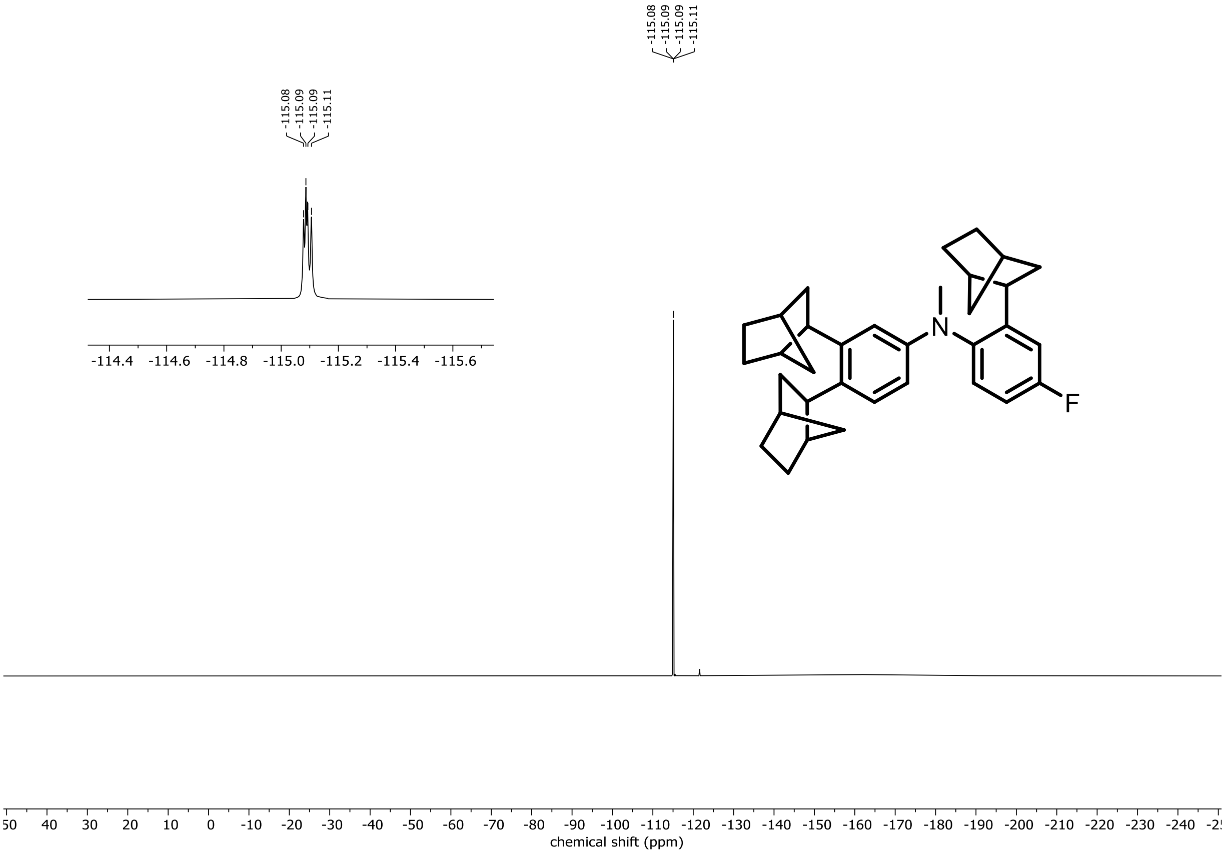

Tri-substituted 15 (tri-15) (^{1}H NMR, 500 MHz, $CDCl_3$)

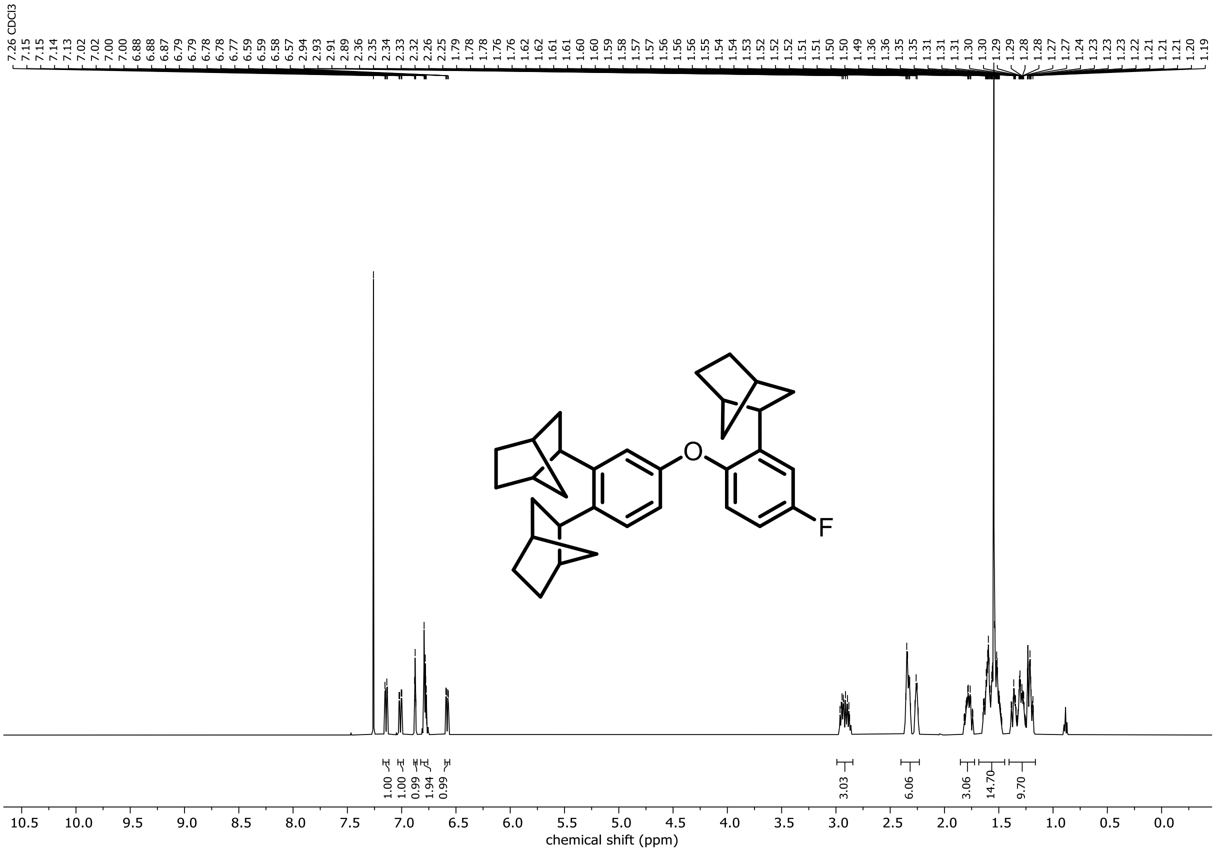

Tri-substituted 15 (tri-15) (^{13}C NMR, 126 MHz, $CDCl_3$)

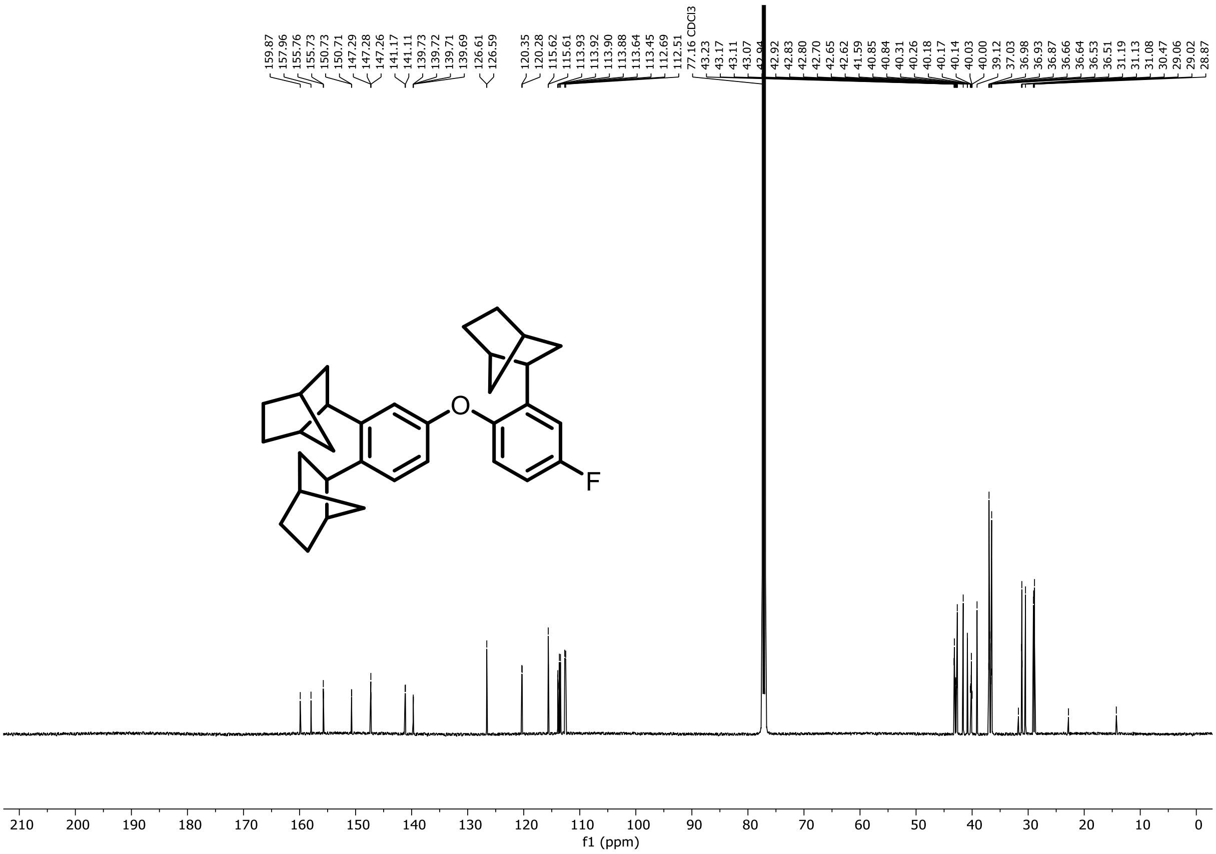

Tri-substituted 15 (tri-15) (^{19}F {^{1}H} NMR, 376 MHz, $CDCl_3$)

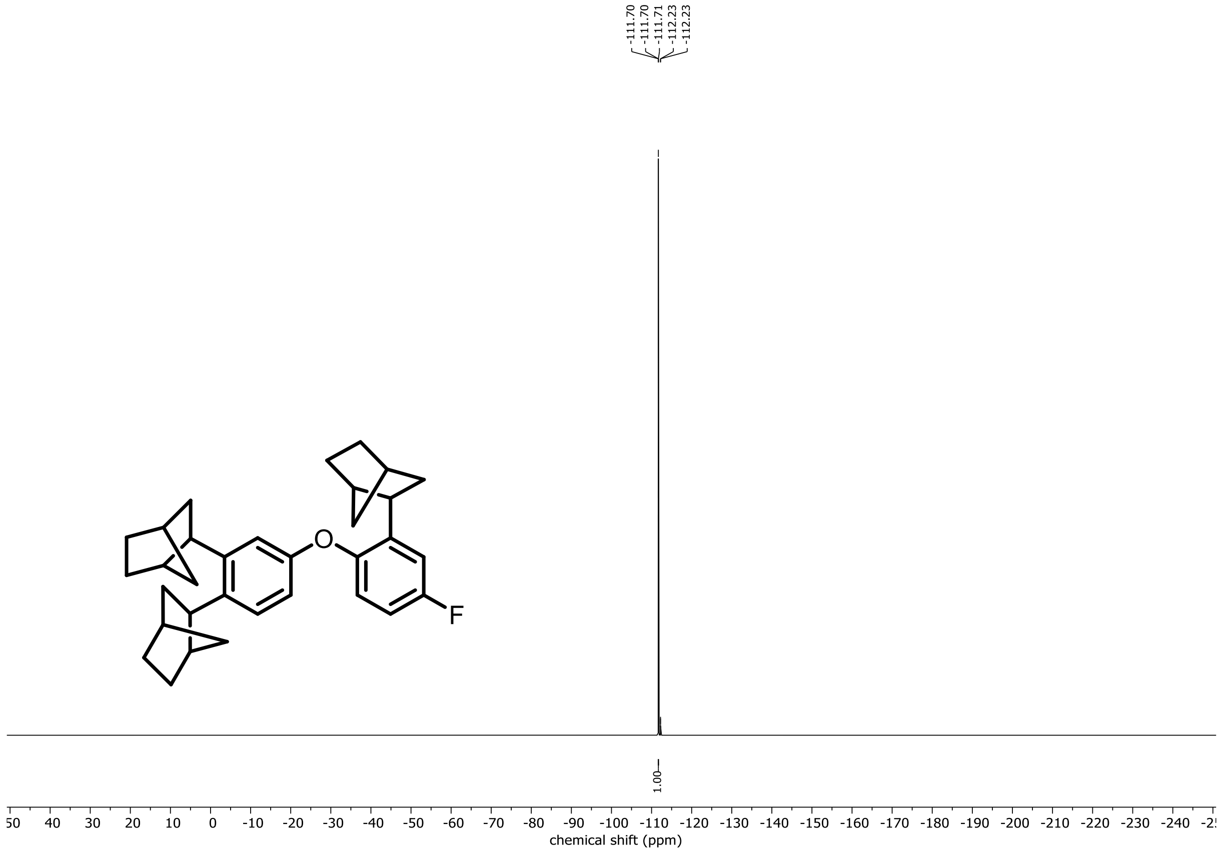

Di-substituted 15 (di-15) (^{1}H NMR, 500 MHz, $CDCl_3$)

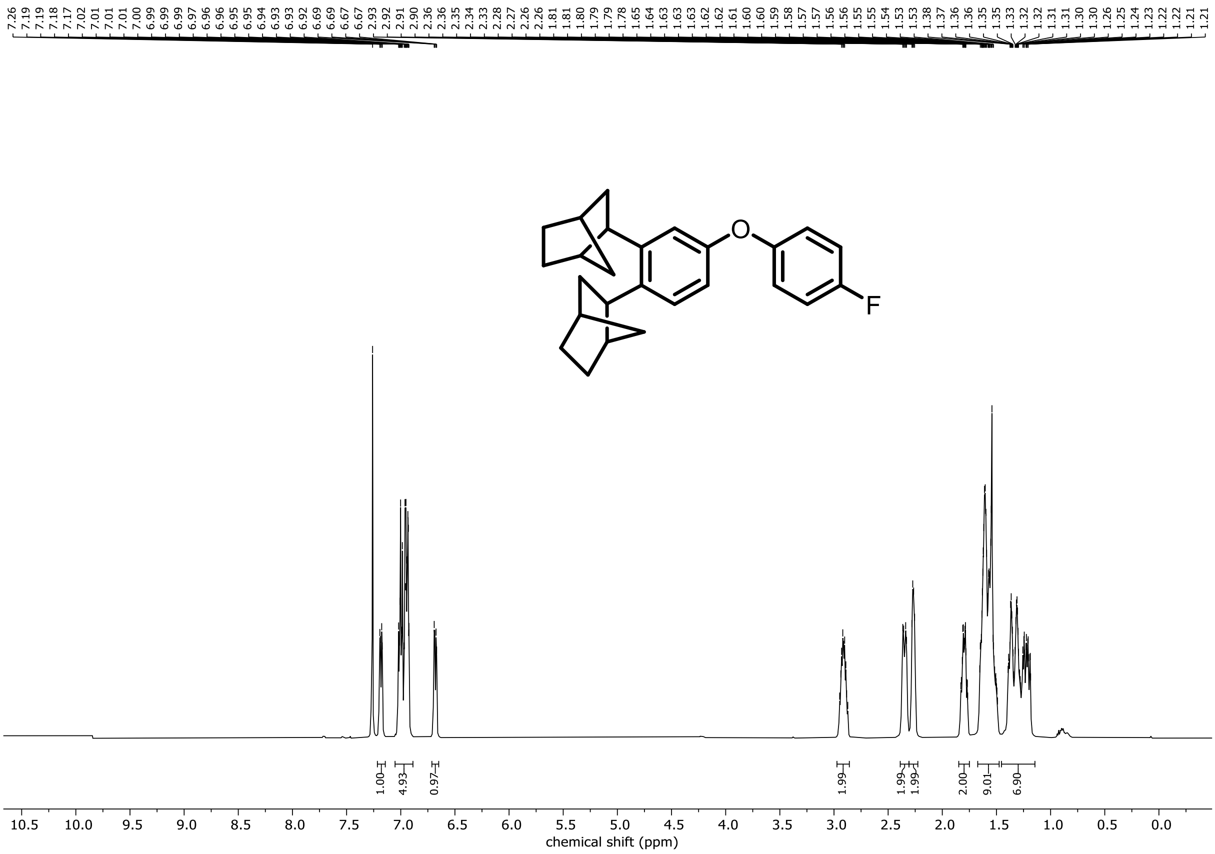

Di-substituted 15 (di-15) (^{1}H NMR, 500 MHz, $CDCl_3$)

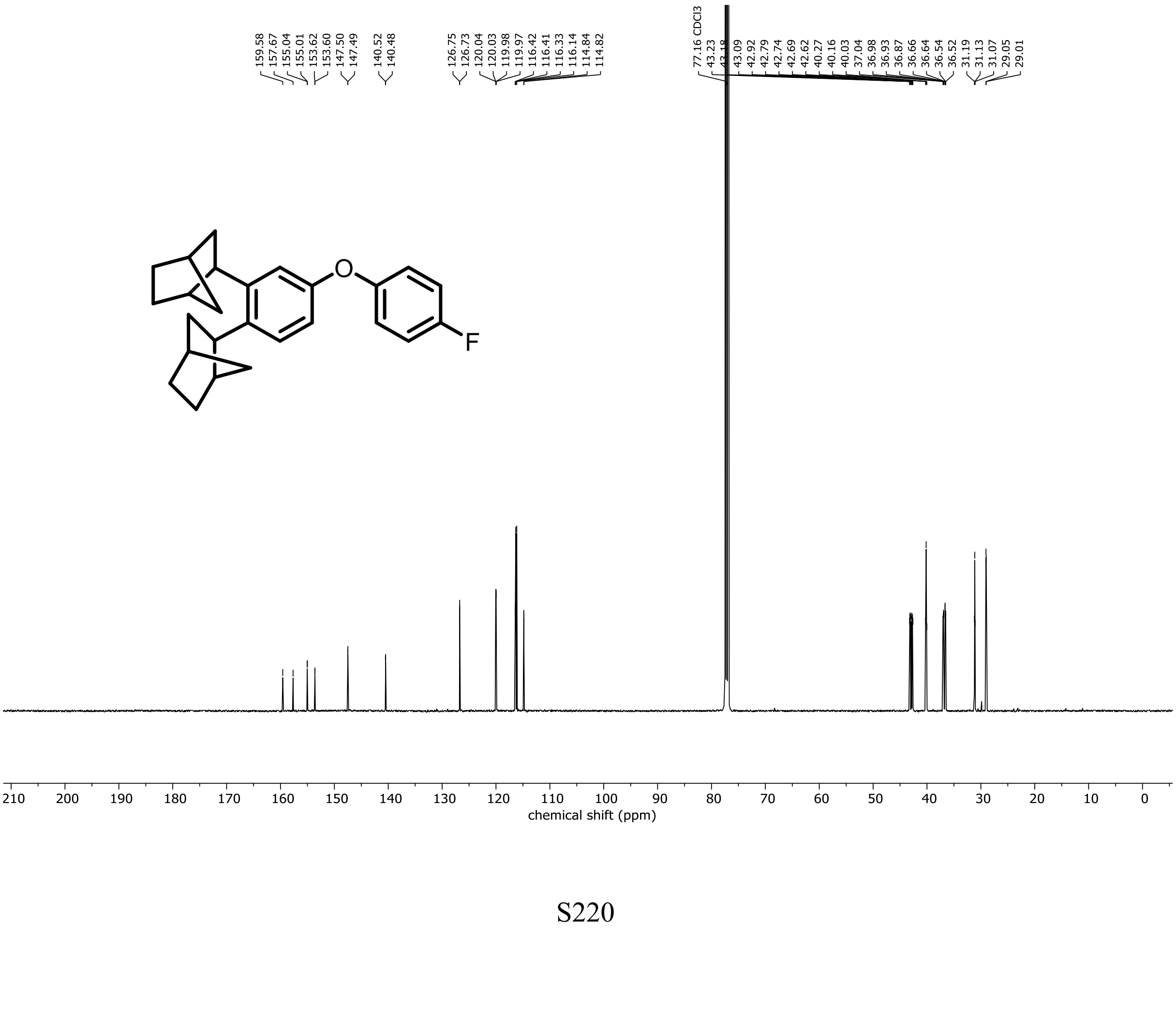

Di-substituted 15 (di-15) (^{19}F NMR, 376 MHz, $CDCl_3$)

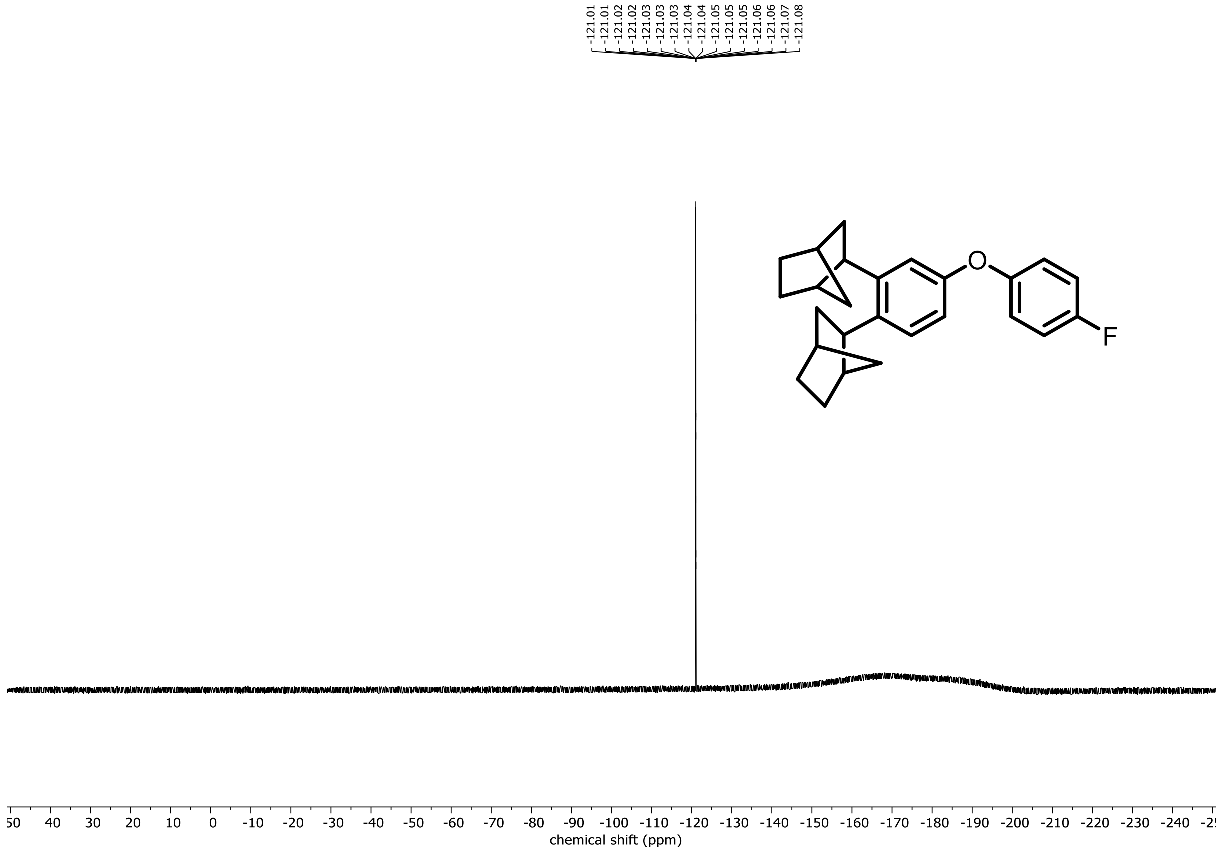

Tri-substituted D-7-1 (D-7-tri-1) (^{1}H NMR, 400 MHz, $CDCl_3$)

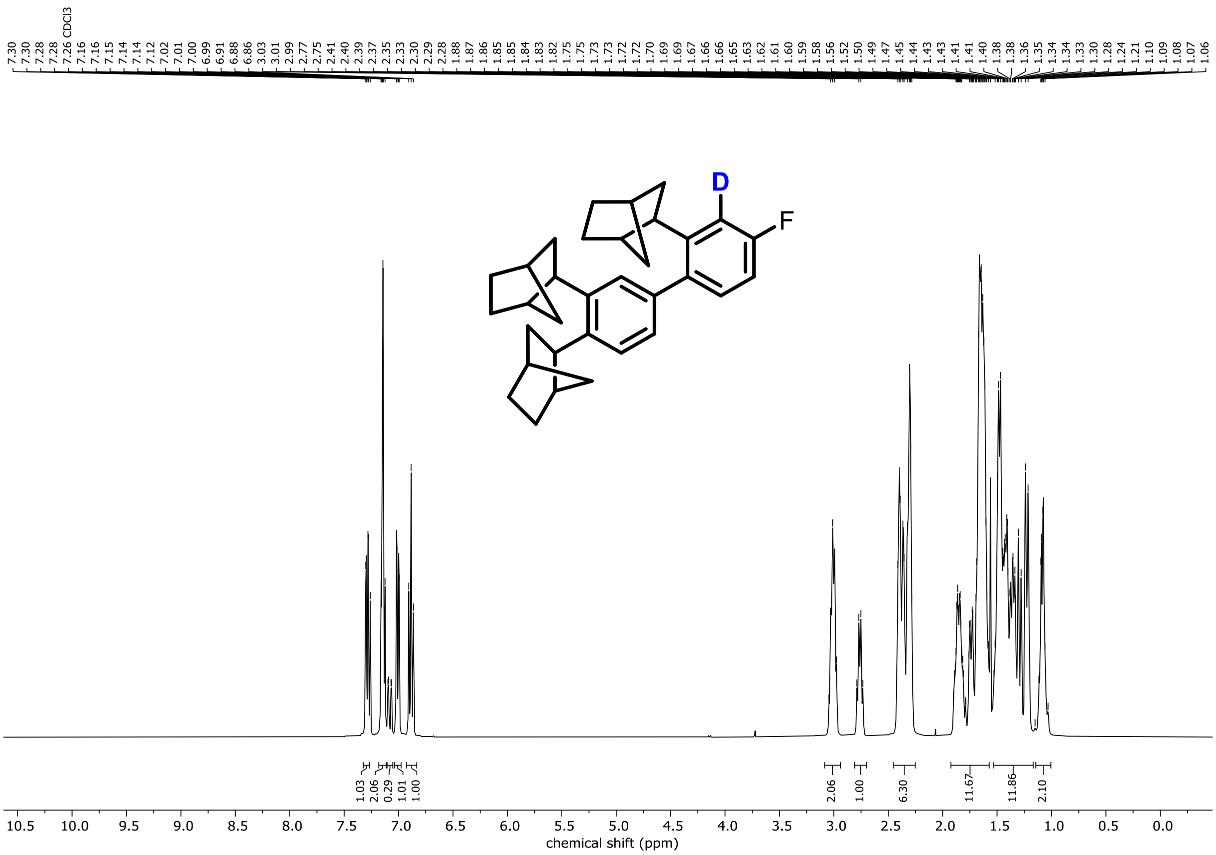

Tri-substituted D-7-1 (D-7-tri-1) (^{13}C NMR, 101 MHz, $CDCl_3$)

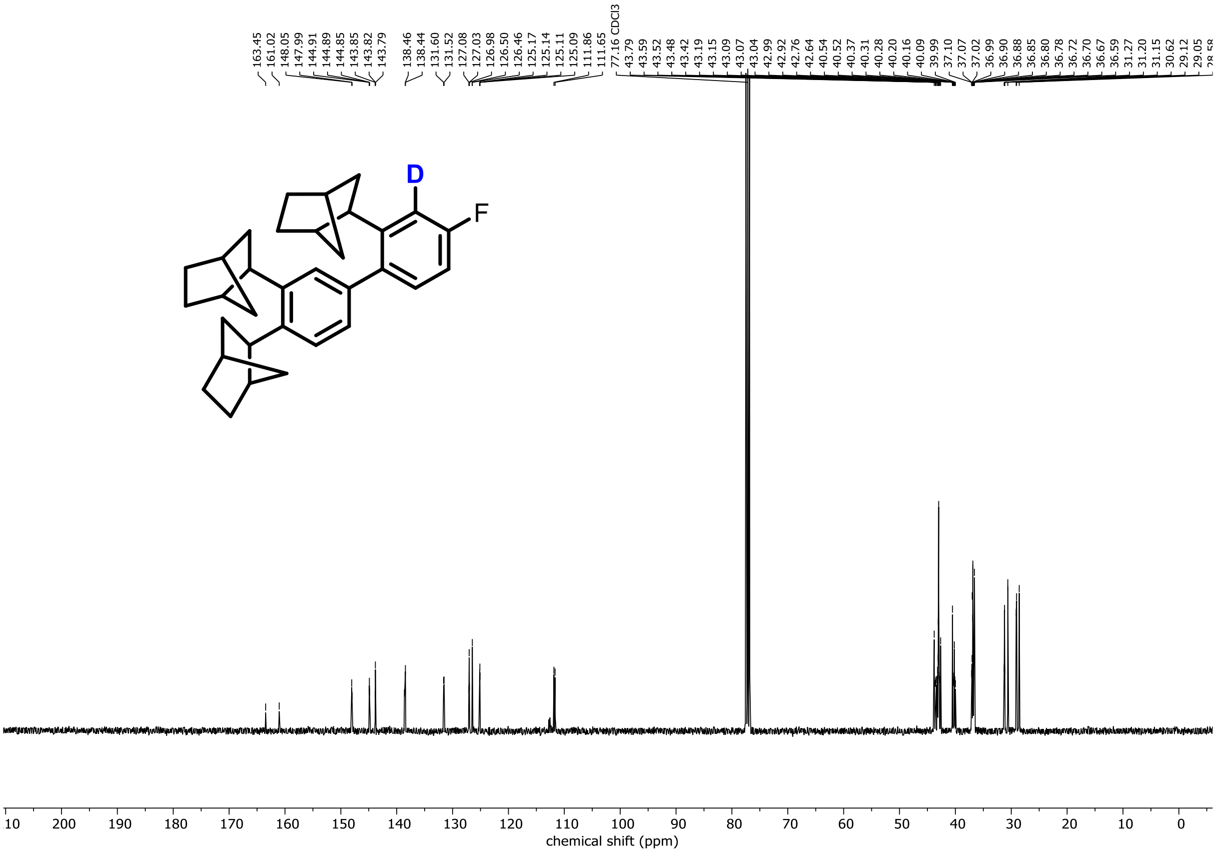

Tri-substituted D-7-1 (D-7-tri-1) (^{19}F {^{1}H} NMR, 376 MHz, $CDCl_3$)

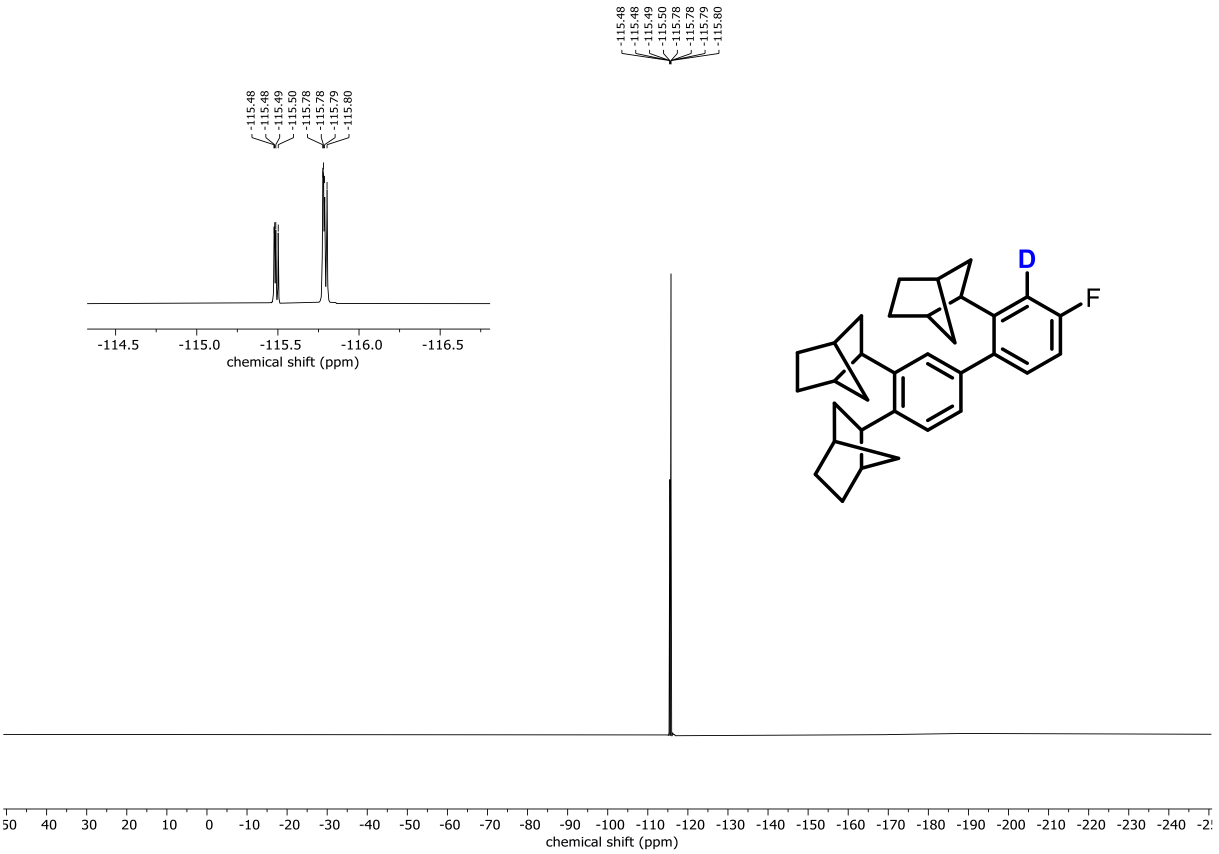

Tri-substituted (3,6)-D$_2$-1 (D$_2$-tri-1) (^{1}H NMR, 400 MHz, CDCl$_3$)

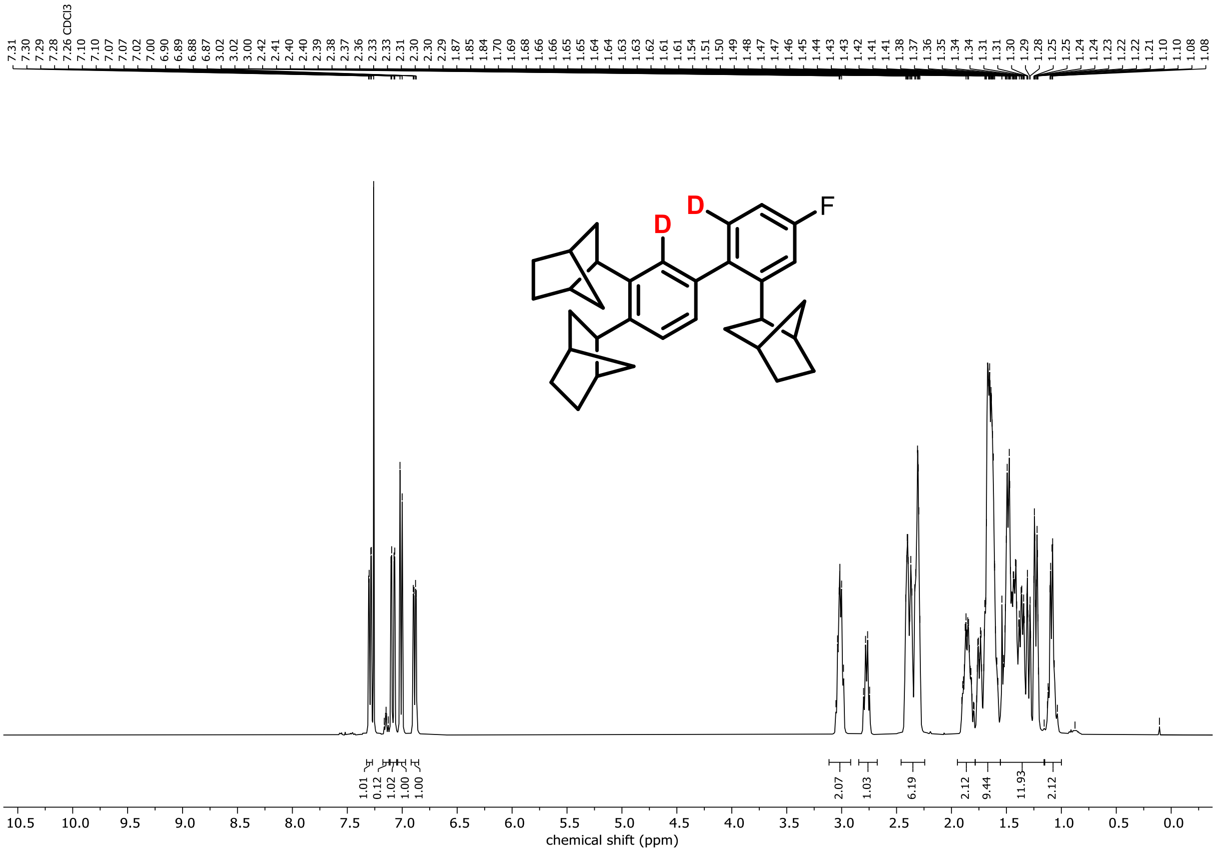

Tri-substituted (3,6)-D$_2$-1 (D$_2$-tri-1) (^{13}C NMR, 101 MHz, CDCl$_3$)

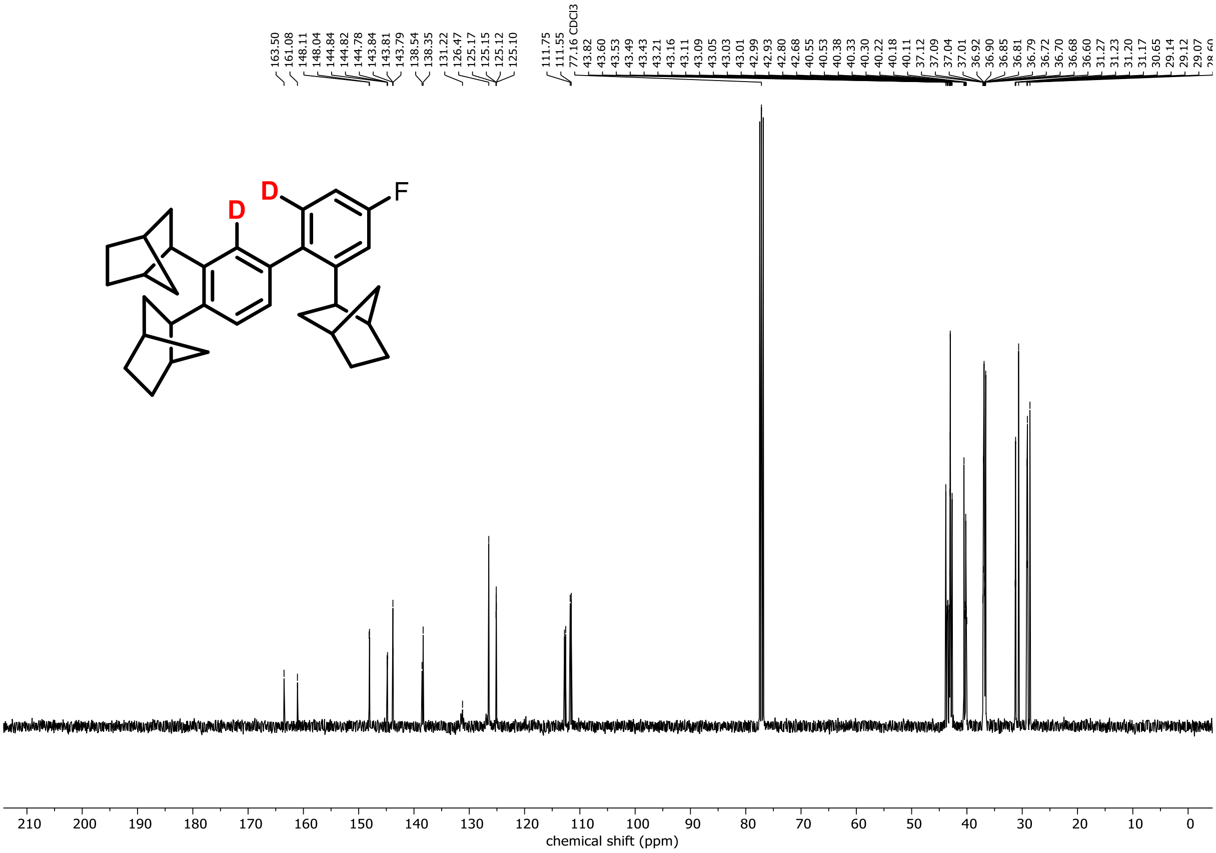

Tri-substituted (3,6)-D_2-1 (D_2-tri-1) (^{19}F {^{1}H} NMR, 376 MHz, $CDCl_3$)

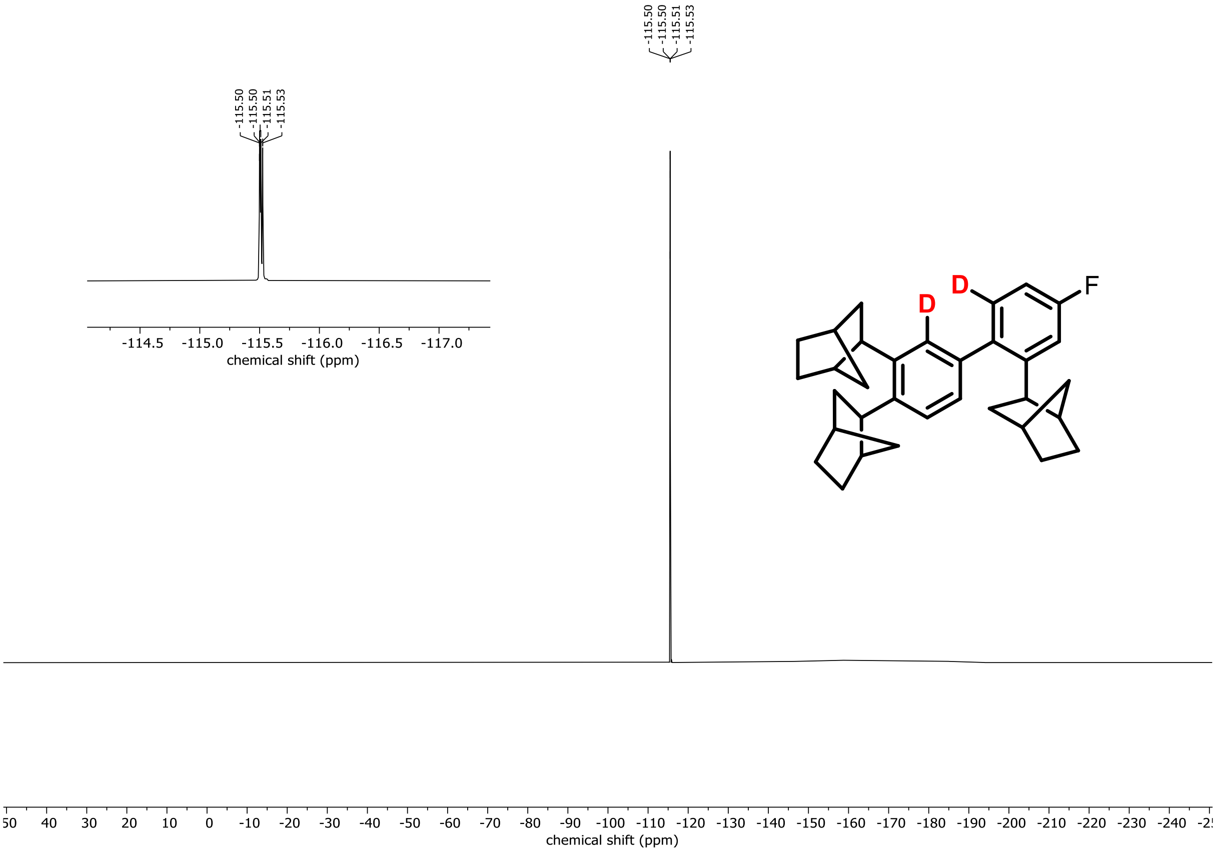

Combined hexa-substituted and tetra-substituted 16 (tetra-16 : hexa-16 = 1.0 : 6.7)

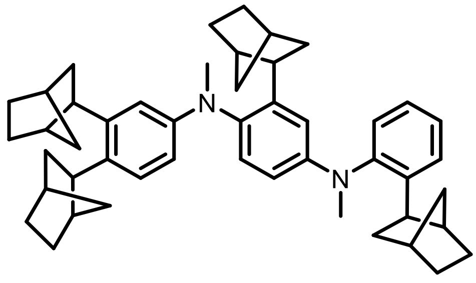

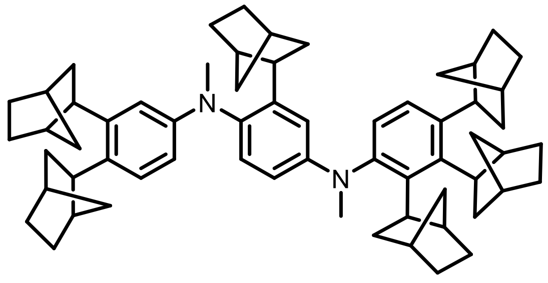

Combined hexa-substituted and tetra-substituted 16 (tetra-16 : hexa-16 = 1.0 : 6.7)

(^{1}H NMR, 500 MHz, C_6D_6)

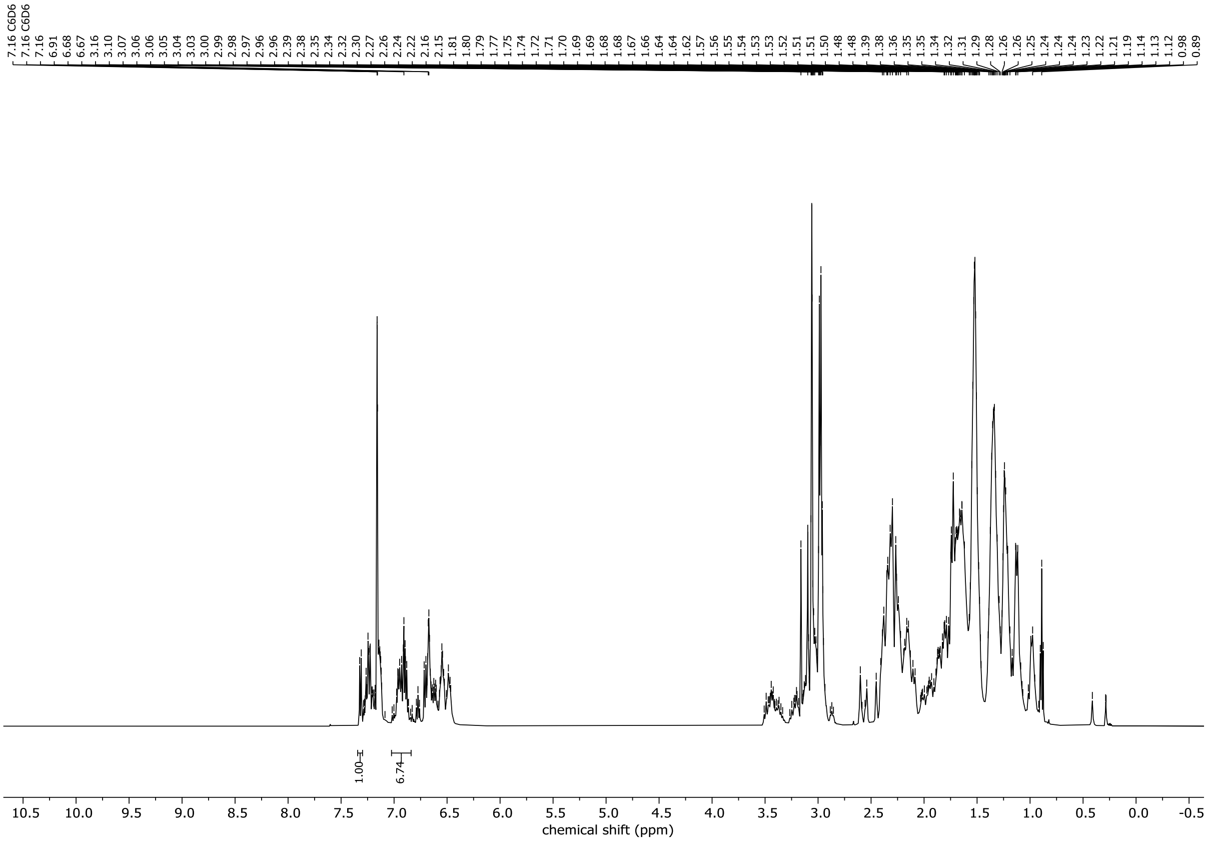

Combined hexa-substituted and tetra-substituted 16 (tetra-16 : hexa-16 = 1.0 : 6.7)

(HRMS (MALDI) *m/z*)

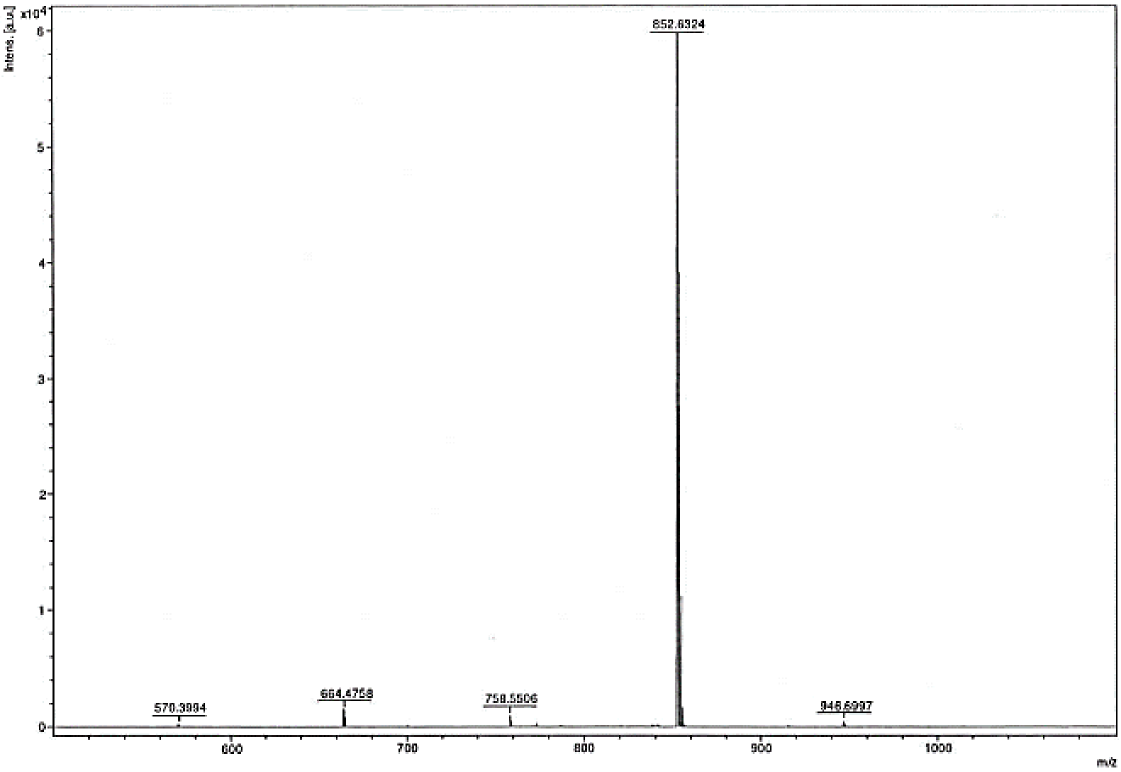

Observed m/z	Theoretical Mass	Error (ppm)	Formula	ID
664.4758	664.4751	1.05	$C_{48}H_{60}N_2$	$[M]^+$
852.6324	852.6316	0.94	$C_{62}H_{80}N_2$	$[M]^+$

Hexa-substituted 16 (hexa-16) (^{1}H NMR, 600 MHz, C_6D_6)

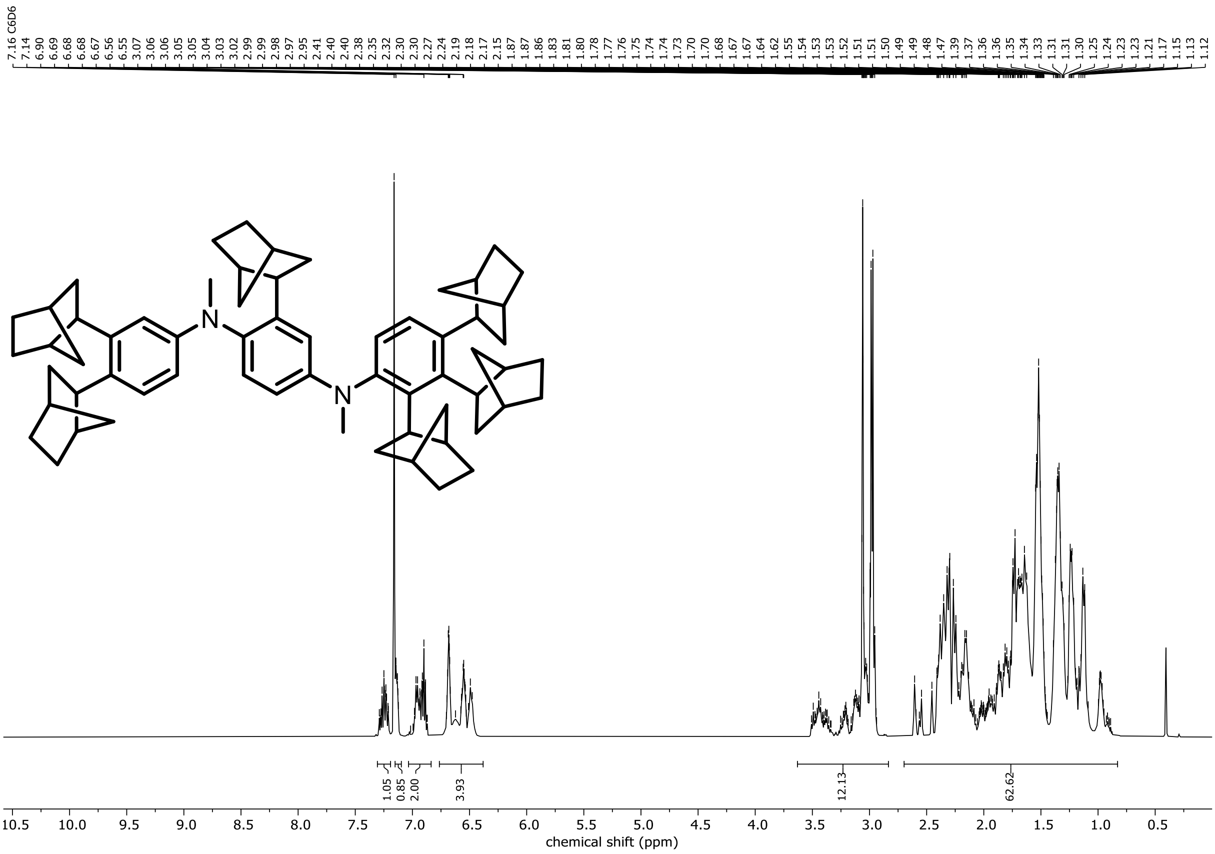

Hexa-substituted 16 (hexa-16) (^{13}C NMR, 151 MHz, C_6D_6)

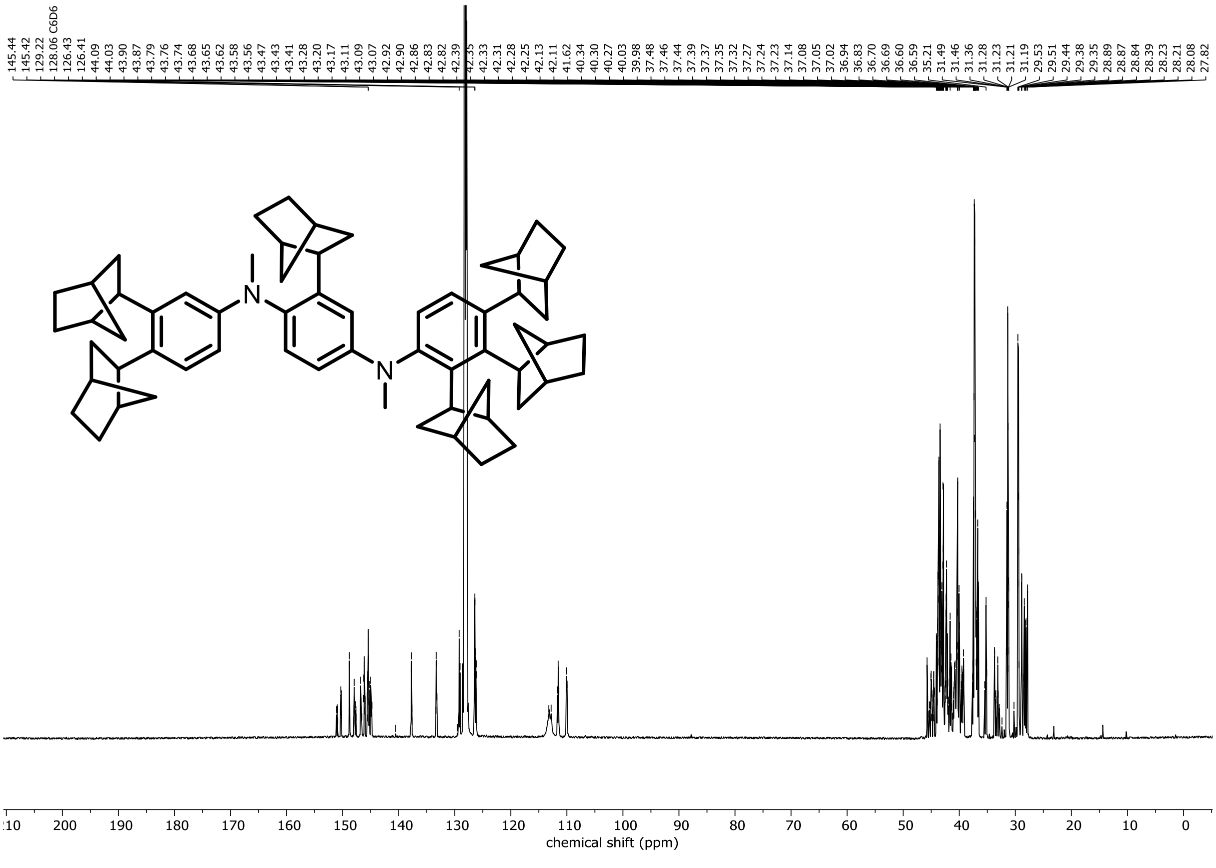

Hexa-substituted 16 (hexa-16) (HRMS (MALDI) *m/z*)

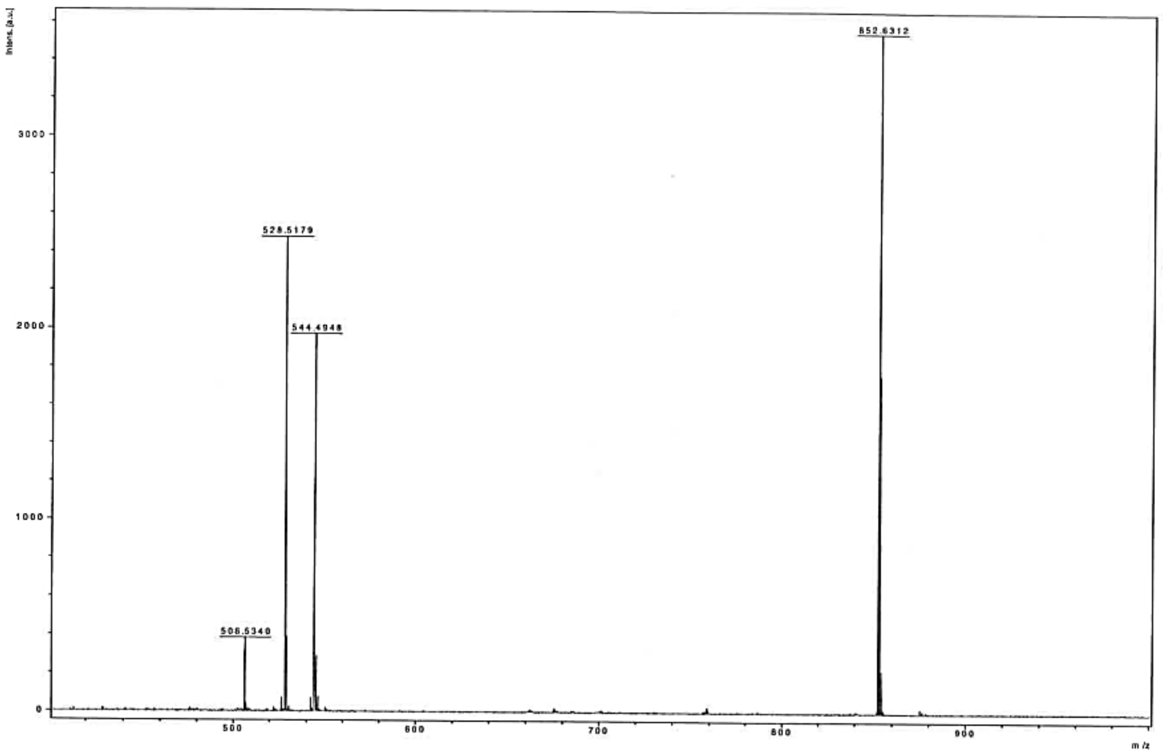

Tetra-substituted 16 (tetra-16) (^{1}H NMR, 600 MHz, C_6D_6; **tetra-16** : **hexa-16** = 5 : 1)

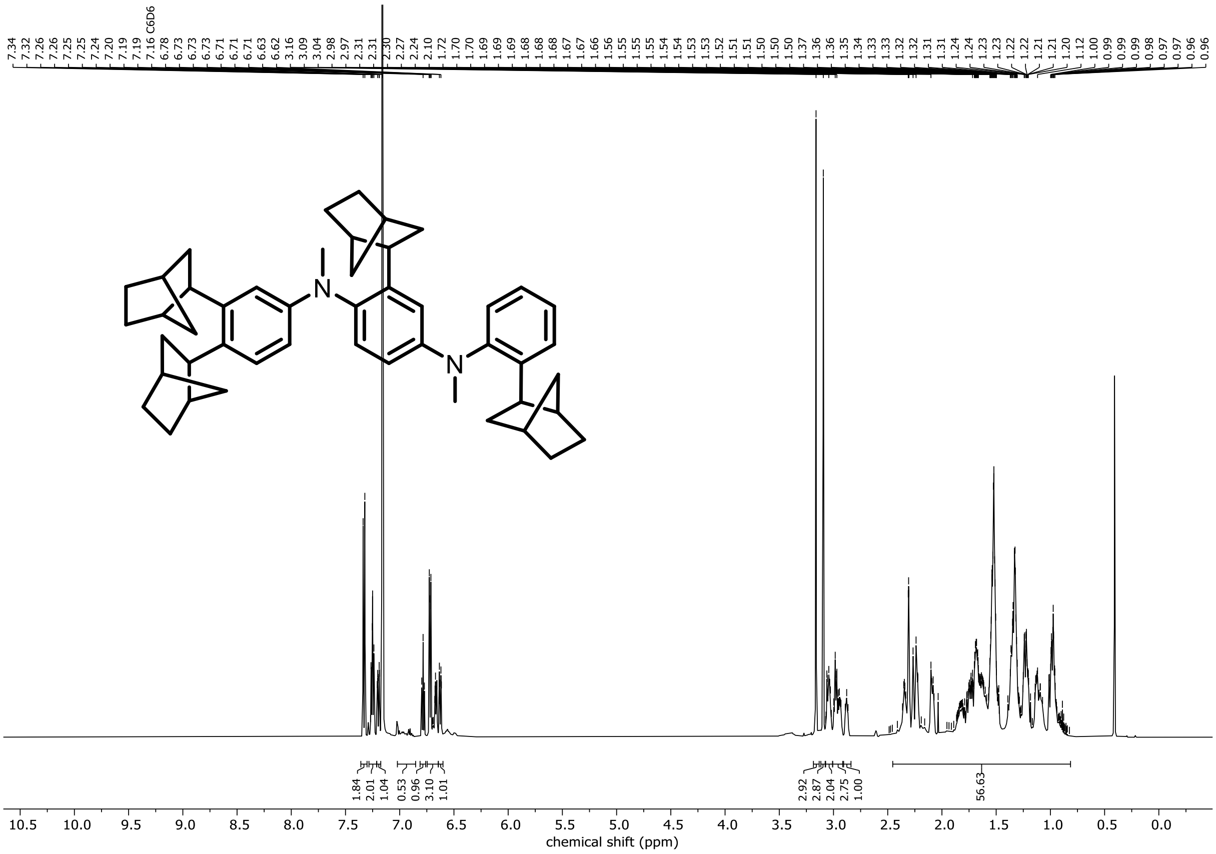

Tetra-substituted 16 (tetra-16) (^{13}C NMR, 151 MHz, C_6D_6; **tetra-16** : **hexa-16** = 5 : 1)

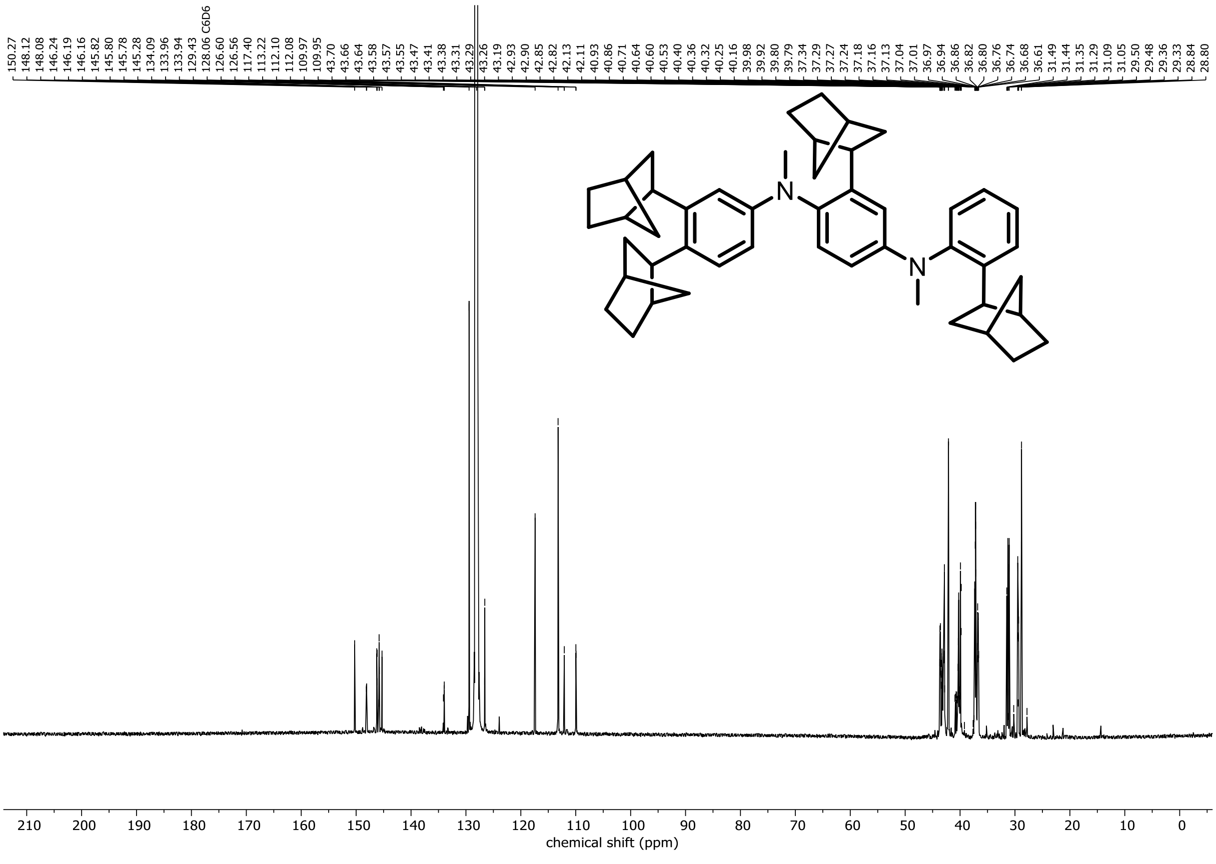

Tetra-substituted 16 (tetra-16) (HRMS (MALDI) *m/z*; **tetra-16** : **hexa-16** = 5 : 1)

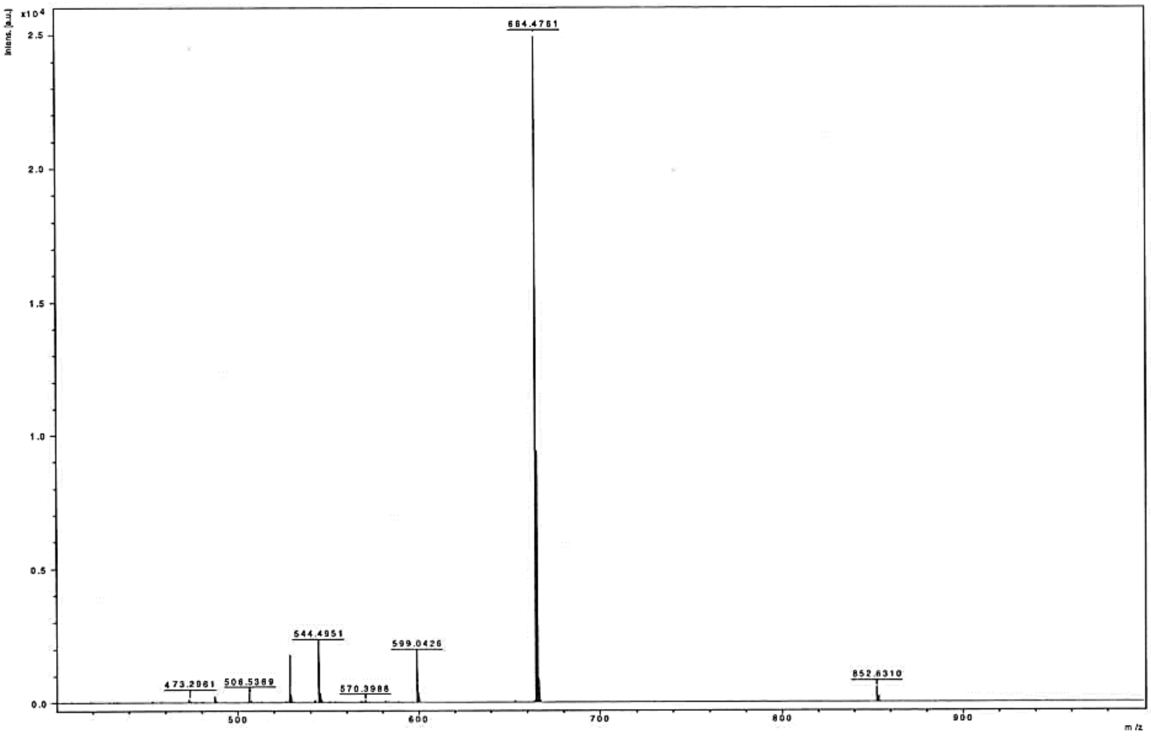

Crude reaction mixture of 17 subjected to optimized reaction conditions (^{1}H NMR, 400 MHz, C_6D_6)

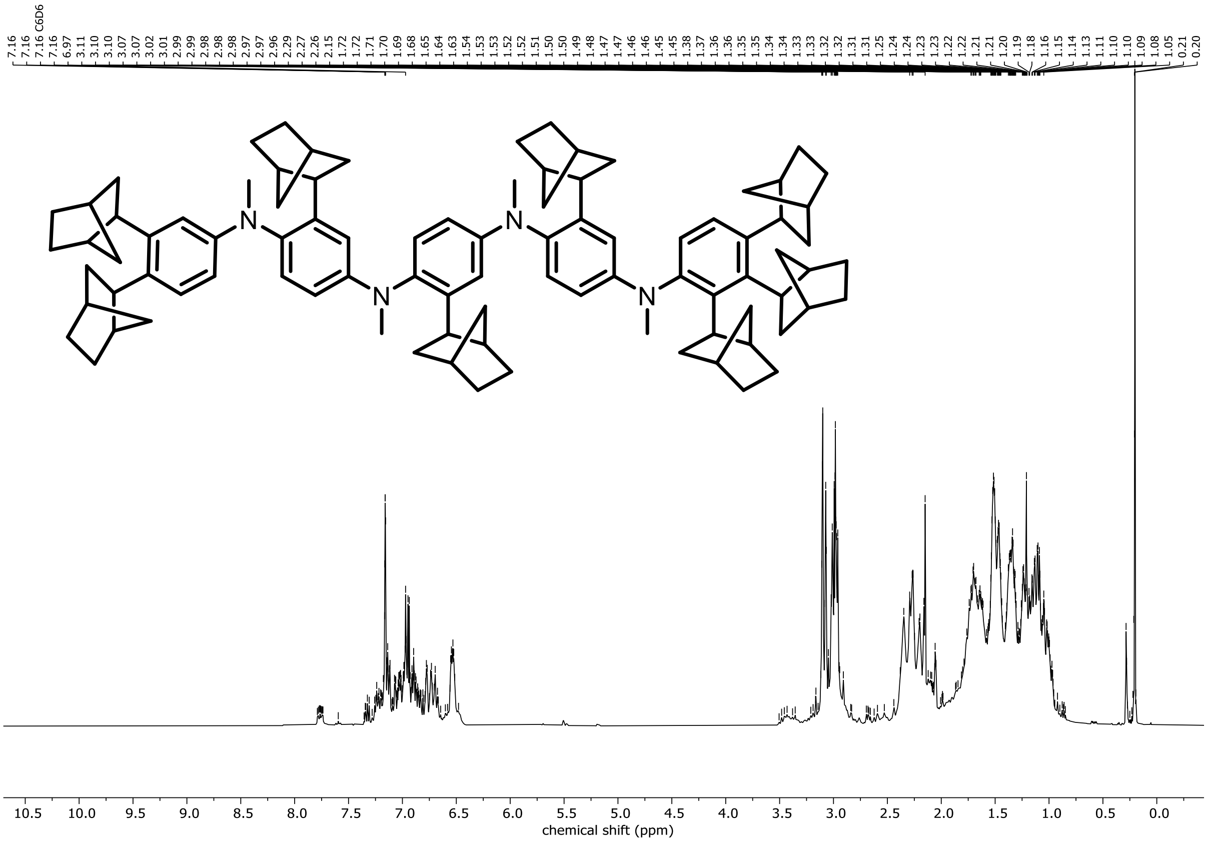

Crude reaction mixture of 17 subjected to optimized reaction conditions (HRMS (MALDI) *m/z*)

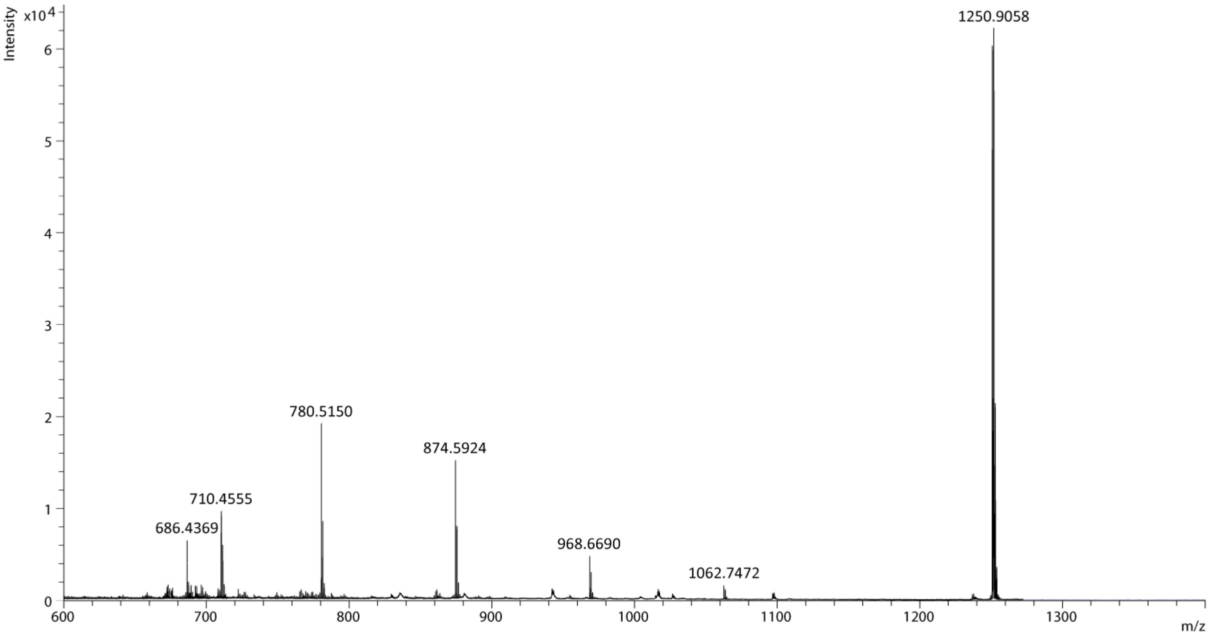

5) References

1. Ball, L. T., Lloyd-Jones, G. C., Russell, C. A. Gold-Catalyzed Oxidative Coupling of Arylsilanes and Arenes: Origin of Selectivity and Improved Precatalyst. *J. Am. Chem. Soc.* **136**, 254–264 (2014).
2. Beyer, C., Böhme, U., Pietzsch, C., Roewer, G. Preparation, characterization and properties of dipolar 1,2-N,N-dimethylaminomethylferrocenylsilanes, *J. Organomet. Chem.* **654**, 187–201 (2002).
3. Campeau, L., Stuart, D. R., Leclerc, J., Bertrand-Laperle, M., Villemure, E., Sun, H., Lasserre, S., Guimond, N., Lecavallier, M., Fagnou, K. Palladium-Catalyzed Direct Arylation of Azine and Azole N-Oxides: Reaction Development, Scope and Applications in Synthesis, *J. Am. Chem. Soc.* **131**, 3291–3306 (2009).
4. Nilsen, A., Miley, G. P., Forquer, I. P. , Mather, M. W., Katneni, K., Li, Y., Pou, S., Pershing, A. M., Stickles, A. M., Ryan, E., Kelly, J. X., Doggett, J. S., White, K. L., Hinrichs, D. J., Winter, R. W., Charman, S.A., Zakharov, L. N., Bathurst, I., Burrows, J.N., Vaidya, A. B., Riscoe, M. K., Discovery, Synthesis, and Optimization of Antimalarial 4(1H)-Quinolone-3-Diarylethers, *J. Med. Chem.*, **57**, 3818–3834 (2014).
5. Flatt, A. K., Tour, J. M., Synthesis of thiol substituted oligoanilines for molecular device candidates, *Tetrahedron Lett.* **44,** 6699–6702 (2003).
6. Sadighi, J. P., Singer, R. A., Buchwald, S. L., Palladium-Catalyzed Synthesis of Monodisperse, Controlled-Length, and Functionalized Oligoanilines, *J. Am. Chem. Soc.* **120**, 4960–4976 (1998).
7. Oguma, K., Miura, M., Satoh, T., Nomura, M. Merry-Go-Round Multiple Alkylation on Aromatic Rings via Rhodium Catalysis. *J. Am. Chem. Soc.* **122**, 10464–10465 (2000).
